# Palladium-Catalyzed Oxidative Allene–Allene Cross-Coupling

**DOI:** 10.1021/jacs.4c14607

**Published:** 2025-01-23

**Authors:** Haibo Wu, Qi Pan, Judith Grill, Magnus J. Johansson, Youai Qiu, Jan-E. Bäckvall

**Affiliations:** † Department of Organic Chemistry, Arrhenius Laboratory, Stockholm University, SE-10691 Stockholm, Sweden; ‡ Medicinal Chemistry, Research and Early Development, Cardiovascular, Renal and Metabolism (CVRM), BioPharmaceuticals R&D, AstraZeneca, Gothenburg, SE-43183 Mölndal, Sweden; § State Key Laboratory of Elemento-Organic Chemistry, Frontiers Science Center for New Organic Matter, Haihe Laboratory of Sustainable Chemical Transformations, College of Chemistry, Nankai University, Tianjin 300071, China

## Abstract

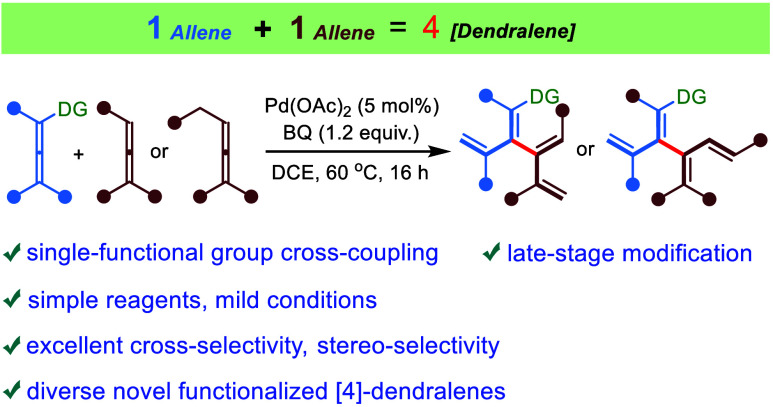

Direct cross-coupling reactions between two similar unactivated partners are challenging but constitute a powerful strategy for the creation of new carbon–carbon bonds in organic synthesis. [4]Dendralenes are a class of acyclic branched conjugated oligoenes with great synthetic potential for the rapid generation of structural complexity, yet the chemistry of [4]dendralenes remains an unexplored field due to their limited accessibility. Herein, we report a highly selective palladium-catalyzed oxidative cross-coupling of two allenes with the presence of a directing olefin in one of the allenes, enabling the facile synthesis of a broad range of functionalized [4]dendralenes in a convergent modular manner. Specifically, the selective allenic C–H activation of an allene with an allyl substituent as the assisting group gives rise to a vinylpalladium intermediate, which reacts with a less substituted allene in a carbopalladation, followed by a β-hydride elimination. The reaction sequence leads to a new C(sp^2^)–C(sp^2^) bond between two diene units. Remarkably, this protocol provides an unconventional strategy for the site-selective and stereoselective construction of C(vinyl)–C(vinyl) bonds without using any halogenated and organometallics olefin precursors. Furthermore, the practical transformations of the synthesized [4]dendralenes and late-stage modifications of biorelevant molecules demonstrate their potential in the total synthesis of natural products and drug discovery.

## Introduction

**Figure 1:**
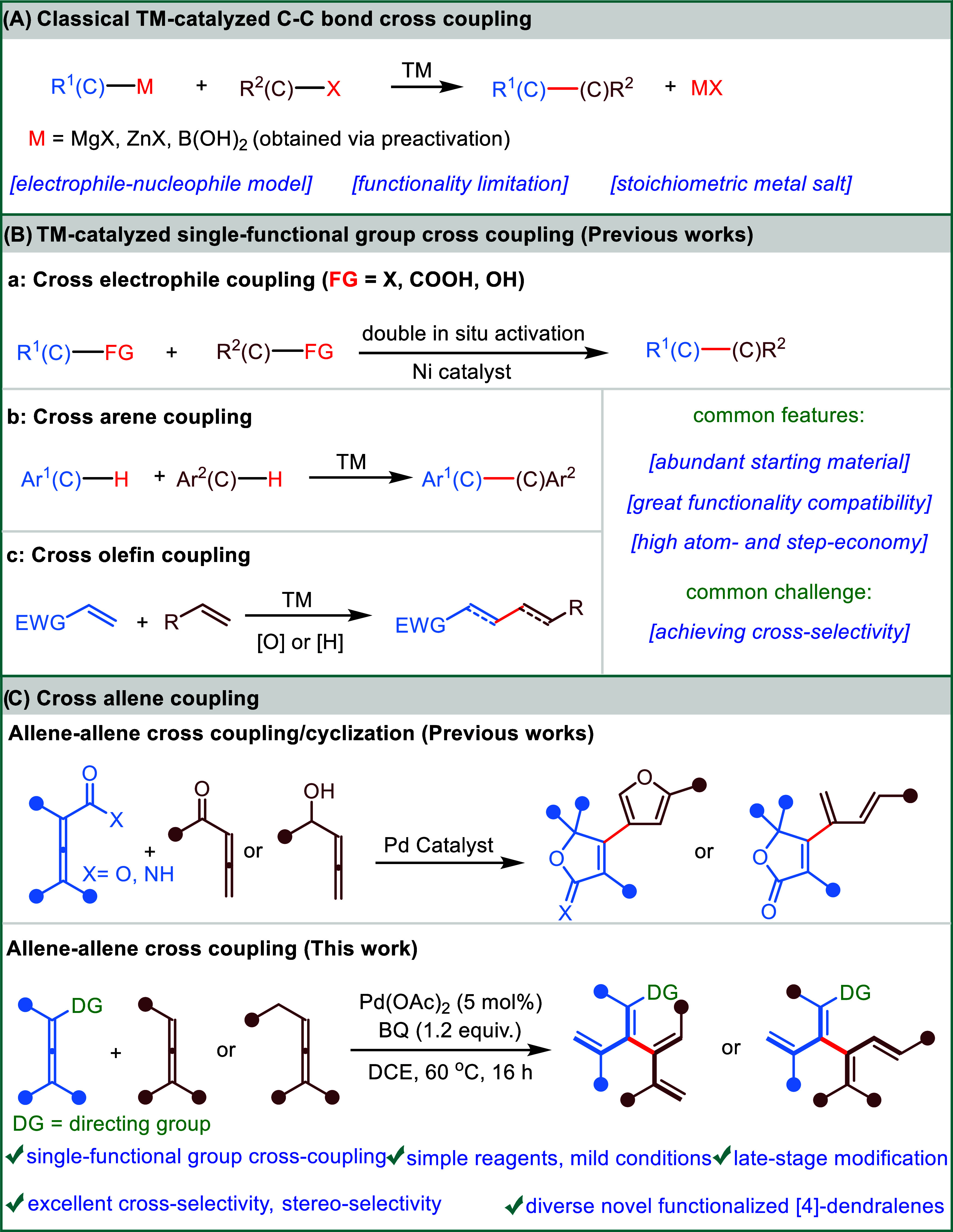
Classical transition-metal (TM)-catalyzed cross-coupling and single-functional group cross-coupling.

Transition-metal-catalyzed cross-coupling has proven to be one of the most important methods for the construction of C–C bonds.^1−7^ In a classical C–C cross-coupling reaction (Figure 1A), an organohalide electrophile and an organometallic nucleophile (such as an organomagnesium, an organozinc, or an organoboron) are usually applied as partners, and stoichiometric amounts of metal salt are generated as waste. The independent preparation of both the electrophile and nucleophile precursors requires extra steps and reagents, limiting the scope of substrates and functional group tolerance.

In the past few decades, the direct formations of C–C linkages from two coupling partners that contain a common functional group, also known as single-functional group cross-coupling,^8^ have attracted significant attention from the chemical community due to their intrinsic advantages in starting material availability, functional group compatibility, and atom- and step-economy.^9−11^ Nevertheless, the state-of-the-art in this field is heavily confined to a handful of functional group classes (Figure 1B) due to the great challenge of achieving cross-selectivity for two similar coupling partners. To harness the elaborate strategy of double in situ activation of two radical precursors, followed by radical sorting with nickel catalysis, the MacMillan and Baran groups have developed a series of cross-electrophile couplings of alkyl halides,^12^ carboxylic acids,^13−15^ and alcohols^8^ (Figure 1B, top), providing strategic disconnections for the construction of C(sp^3^)–C(sp^3^) bonds in organic synthesis. Moreover, recent advances in the development of electrochemically cross-electrophile coupling have also contributed to cross-alkyl halide coupling.^16,17^ Using the strategy involving a directing group for the arene C–H bond activation, the groups of Shi^18^ and Ackermann^19^ have successfully realized cross-arene coupling, offering straightforward approaches for the formation of C(sp^2^)–C(sp^2^) from two aromatic C–H bonds (Figure 1B, middle).

Olefins and polyenes are fundamental motifs in organic molecules, ranging from natural products and drugs to functional materials. Therefore, efficient cross-couplings for direct linkage between two different olefinic carbons are undoubtedly valuable and highly desired, as represented by the widely applied examples of olefin metathesis.^20^ Although considerable progress has been achieved recently in the cross-coupling of olefins with other types of partners,^21,22^ direct cross-olefin coupling remains elusive (Figure 1B, bottom). In 2009, the Loh group^23^ disclosed a direct cross-coupling of simple alkenes with acrylates via palladium-catalyzed dehydrogenative coupling. The difference in electron densities of the two olefins is crucial for achieving high cross-selectivity. Utilizing elaborate radical pathways, the Baran^24^ and Melchiorre^25^ groups reported two reductive olefin cross-couplings that enable rapid access to sp^3^-dense molecules. In a recent study, the Gevorgyan group demonstrated that electron-deficient and electron-rich alkenes could also be cross-coupled in a hydroalkenylation fashion.^26^ As illustrated by the aforementioned achievements in the field of single-functional group cross-coupling, unlocking new classes of functional groups as such would offer a paradigm shift in the way C–C bonds were created that were previously difficult or impossible to access.

The ever-increasing demands in the synthetic efficiency and sustainability of chemical production have motivated chemists to search for more straightforward methods to construct molecular complexity from abundant and simple starting materials.^27−31^ Allenes represent a class of readily accessible substrates that have been extensively studied and applied in numerous powerful organic reactions.^32−36^ In sharp contrast, so far, the cross-coupling of allenes remains largely unexploited, presumably due to the formidable challenge of overcoming the competing homodimerization^37−40^ or cyclization reactions (Figure 1C, top).^41−43^ [4]Dendralenes constitute a structural family of acyclic tetraenes with cross-conjugated connections between two diene units.^44^ Another sister family of the simplest category of dendralenes is [3]dendralenes, which have exhibited great potential in the synthesis of natural products and polymer materials,^45,46^ featuring rapid generation of fused and bridged multicyclic systems via unique diene-transmissive cycloadditions and electrocyclizations.^47^ In contrast, to date, the synthesis and application studies on [4]dendralenes are far less developed. Based on a variation of classical Suzuki-Miyaura cross-coupling, a recent work by the Sherburn group^48^ represents the only general approach for the synthesis of pure hydrocarbon [4]dendralenes (i.e., unfunctionalized [4]dendralenes).^49−51^ Continuing with our ongoing research in the field of allene chemistry^52−54^ and green oxidation reactions,^55^ we sought to develop an oxidative intermolecular allene cross-coupling that provides an efficient method for the synthesis of diverse functionalized [4]dendralenes in a highly convergent modular fashion. A critical challenge would be to precisely control the site-selective allenic C–H bond activation and carbopalladation in a sequential manner to suppress possible side reactions, such as homocoupling and cyclization. Here, we communicate the development of a palladium-catalyzed oxidative cross-coupling of two allenes with the presence of a directing group (DG) in one of the allenes (Figure 1C, bottom). A range of functionalities are well-tolerated on both allenes in the reactions, resulting in a diverse library of novel functionalized [4]dendralenes. We anticipate that our findings will promote the development of [4]dendralenes chemistry and pave the way toward concise construction of C(vinyl)-C(vinyl) bonds in the synthesis of complex molecules.

## Results and Discussion

**Figure 2:**
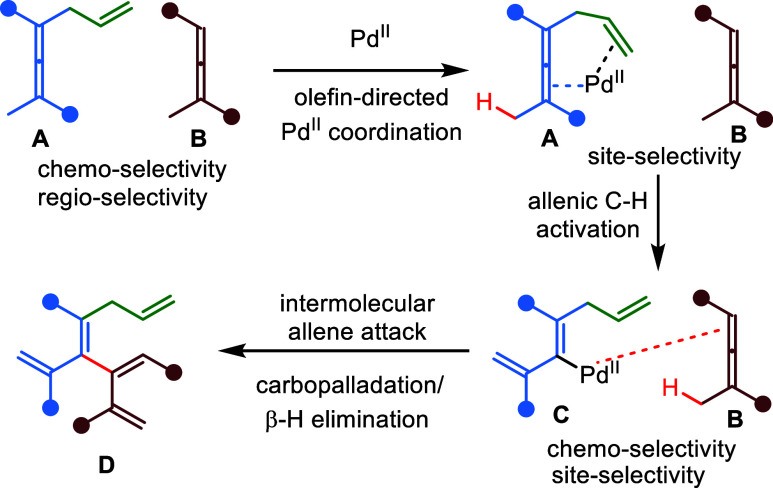
Envisioned strategy for allene–allene cross-coupling and potential challenges.

In our previous studies on palladium-catalyzed oxidative transformations of allenes, we discovered that a simple allylic substituent can serve as an effective directing group for the allenic C–H bond activation at the opposite end of the allene.^56,57^ Inspired by this finding, we envisioned an intermolecular allene cross-coupling strategy (Figure 2): a vinylpalladium intermediate **C** generated from an “activated” allene **A** (with a directing group) will be trapped by another “unactivated” but less sterically hindered allene **B** (without directing group) via a Heck-type pathway to afford the cross-coupling product **D**. However, the implementation of this proposal presents several potential selectivity challenges, as shown in Figure 2. First, the palladium catalyst must be selectively occupied by allene **A** in the presence of allene **B** in the initial step, where an intramolecular carbocyclization^58^ involving the directing olefin may be a possible side reaction. Second, once the vinylpalladium intermediate **C** has been formed as intended, allene **B** needs to be reactive enough to trap **C** over other competing pathways, such as homodimerization and intramolecular Heck-type reaction with the olefin. Third, the control of the overall geometry of product **D** in a single step would be a significant challenge since several possible stereoisomers of the newly generated olefins could be generated.

**Figure 3:**
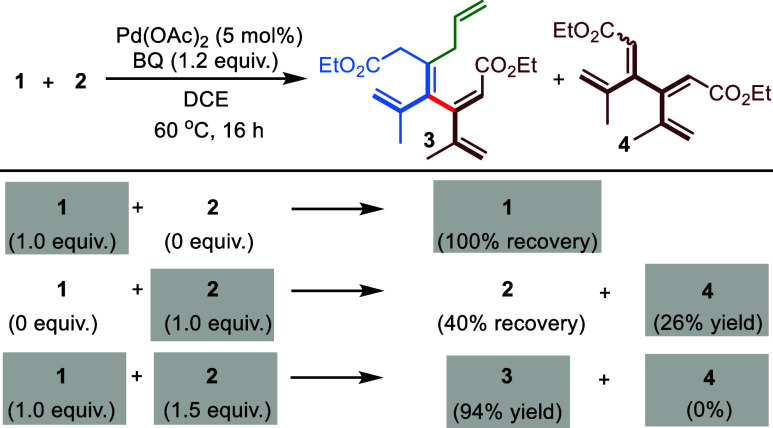
Cross-selectivity vs homodimerization.

Taking these potential challenges into account, we initiated the development of allene–allene cross-coupling using a tetrasubstituted allene **1** and a simple trisubstituted allene **2** as the model substrates. Under similar reaction conditions as developed in previous work,^57^ using Pd(OAc)_2_ as a catalyst and 1,4-benzoquinone (BQ) as a stoichiometric oxidant in toluene at 60 °C, the reaction afforded the desired cross-coupling product **3** as a single stereoisomer in 76% yield (Table 1, Entry 1). Screening of palladium(II) catalysts showed that Pd(OAc)_2_ was the optimal catalyst (Table 1, Entries 1–4). Changing the oxidant to 2,6-dimethyl-BQ (DMBQ) did not affect the yield (Table 1, Entry 5), while tetrafluoro-1,4-benzoquinone (F_4_–BQ) led to a reduction of yield (Table 1, Entry 6). Further screening of various solvents (Table 1, Entries 7–14) demonstrated that several commonly used solvents resulted in moderate to excellent yields (63–94%) of [4]dendralene **3**, with the highest yield obtained when DCE was used (Table 1, Entry 11). Strongly coordinating solvents such as dimethyl sulfoxide (DMSO) and dimethylformamide (DMF) led to a complex mixture with trace amounts of desired product **3** (Table 1, Entries 12–13). Notably, the present transformation is operationally straightforward, requiring only simple mixing of all of the reagents without necessitating precautions against air and moisture. Interestingly, a biomimetic aerobic version^55,59^ of the developed allene–allene cross-coupling using 5 mol % of Co(salophen) as an electron-transfer mediator (ETM) and catalytic amounts of BQ (20 mol %) under an atmosphere of O_2_ also gave high yield of the [4]dendralene product **3** (Table 1, Entry 15). As an alternative approach, the aerobic version is more sustainable as H_2_O is the only byproduct in this process. Additionally, control experiments (Figure 3) with a single allene under optimized reaction conditions indicated that homodimerization of tetrasubstituted allene **1** did not occur, whereas self-coupling of trisubstituted allene **2** resulted in a 26% yield. Remarkably, exclusive cross-selectivity was observed when a combination of allenes **1** and **2** was applied.

**Table 1:**
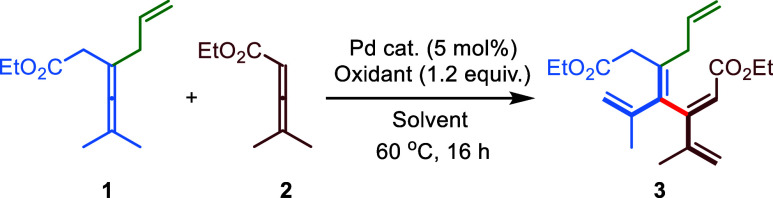
Optimization of the Reaction Conditions^a^

entry	Pd catalyst	solvent	oxidant	yield of **3** [%]^b^
1	Pd(OAc)_2_	toluene	BQ	76
2	Pd(TFA)_2_	toluene	BQ	71
3	Pd (PPh_3_)_2_Cl_2_	toluene	BQ	<5
4	PdCl_2_	toluene	BQ	<5
5	Pd(OAc)_2_	toluene	DMBQ	74
6	Pd(OAc)_2_	toluene	F_4_–BQ	69
7	Pd(OAc)_2_	THF	BQ	63
8	Pd(OAc)_2_	CH_3_CN	BQ	51
9	Pd(OAc)_2_	1,4-dioxane	BQ	84
10	Pd(OAc)_2_	CHCl_3_	BQ	71
11	Pd(OAc)_2_	DCE	BQ	94(93^c^)
12	Pd(OAc)_2_	DMF	BQ	<5
13	Pd(OAc)_2_	DMSO	BQ	<5
14	Pd(OAc)_2_	MeOH	BQ	80
15^d^	Pd(OAc)_2_	DCE	O_2_/ETM	93

a Reaction conditions: allene **1** (0.1 mmol), allene **2** (1.5 equiv), Pd catalyst (5 mol %), and oxidant (1.2 equiv) in 1.0 mL of solvent at 60 °C for 16 h.

b Yields were determined by ^1^H NMR using anisole as an internal standard.

c Isolated yield of **3**.

d Aerobic conditions: ETM = Co(salophen) (5 mol %), BQ (20 mol %), and O_2_ (balloon pressure).

**Scheme 1:**
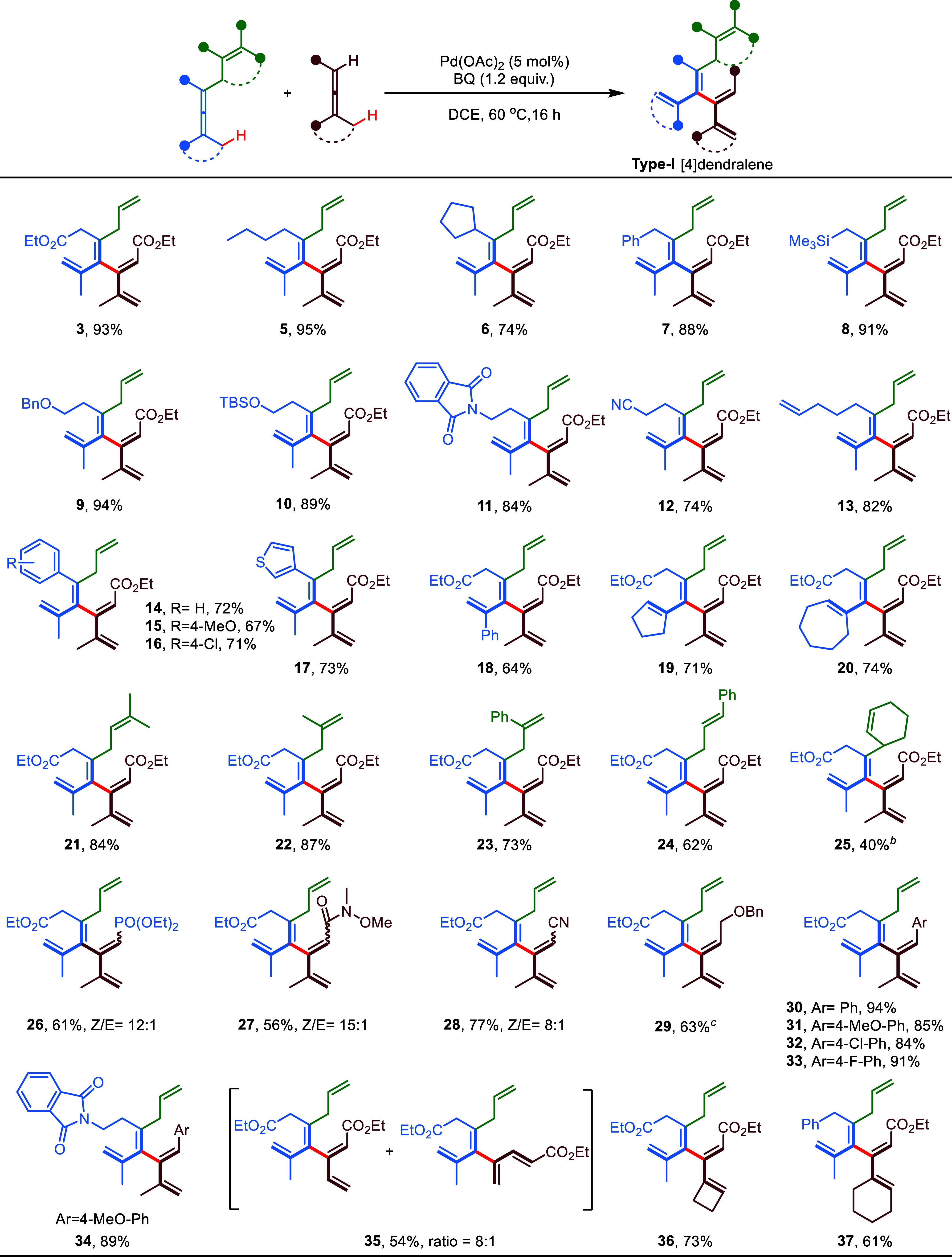
Reaction Scope: Formation of Type-I [4]Dendralenes Reaction conditions: enallene (0.2 mmol), directing group-free allene (1.5 equiv), Pd catalyst (5 mol %), and BQ (1.2 equiv) in 2.0 mL of DCE at 60 °C for 16 h, isolated yields. Reaction carried out at 70 °C. DMBQ was used instead of BQ.

With the proof of concept established, a variety of combinations of enallenes (bearing an allyl group) and directing group-free allenes were evaluated for cross-allene coupling (Scheme 1). Starting with a variation of enallenes, a wide range of functional groups including ester (**3**), alkyl (**5**, **6**, and **7**), silyl ether (**8**), protected alcohol (**9**, **10**), protected amine (**11**), cyano (**12**), alkene (**13**), arenes (**14**, **15**, and **16**), and heteroarene (**17**) on the adjacent position of the directing allyl group were well-tolerated, and the corresponding cross-coupling product with allene **2** was obtained in good to excellent yields (67–95%) with exclusive stereoselectivity. The geometry of the olefins in the obtained [4]dendralenes was determined by the use of nuclear Overhauser effect (NOE) studies (Supporting Information). Increasing the steric hindrance of the substituent (**6** and **14** versus **3** and **5**) seemingly affected the product yield. Interestingly, the presence of another olefin on a longer chain than the allyl group did not interfere with the cross-coupling, and product **13** was obtained in good yield (82%). Replacing one of the two methyl groups on model enallene **1** with a phenyl led to a lower yet acceptable yield (64%) of [4]dendralene **18**. Moreover, enallenes bearing cyclic moieties were also feasible in the present transformation, yielding products **19** and **20** with 71 and 74% yield, respectively. Considering the potential for further applications of the developed protocol in practical synthesis, the generality of the directing group would be highly important. To our delight, in addition to the simplest allyl, a series of ubiquitous allylic groups, including prenyl (**21**), isoprenyl (**22**), β-methylenephenethyl (**23**), and cinnamyl (**24**), could also serve as efficient directing groups, affording the desired cross-coupling product in 62–87% yields. A significant decrease in yield (40%) was observed when a cyclic directing group was applied (**25**), presumably due to the increased steric hindrance around the allene center. Next, we continued to investigate the scope of directing group-free allenes. Various functional groups, such as phosphonate (**26**), amide (**27**), cyano (**28**), and benzyl ether (**29**) attached to trisubstituted allenes, were compatible with the cross-coupling conditions, where the corresponding products were obtained in moderate to good yields (56–77%) with slightly lower Z/E ratios. Trisubstituted allenes bearing an aromatic substituent are also ideal coupling partners for enallenes, where good yields were obtained in all of the cases shown (**30**, **31**, **32**, **33**, and **34**) regardless of the electronic properties. Interestingly, the coupling of a disubstituted allene with the model enallene **1** generated an 8:1 mixture (**35**) of products connected to the middle and terminal allene carbon centers, respectively. Moreover, cyclic trisubstituted allenes can also be coupled with enallenes, yielding the [4]dendralene products **36** and **37** in 73 and 61% yields, respectively.

**Scheme 2:**
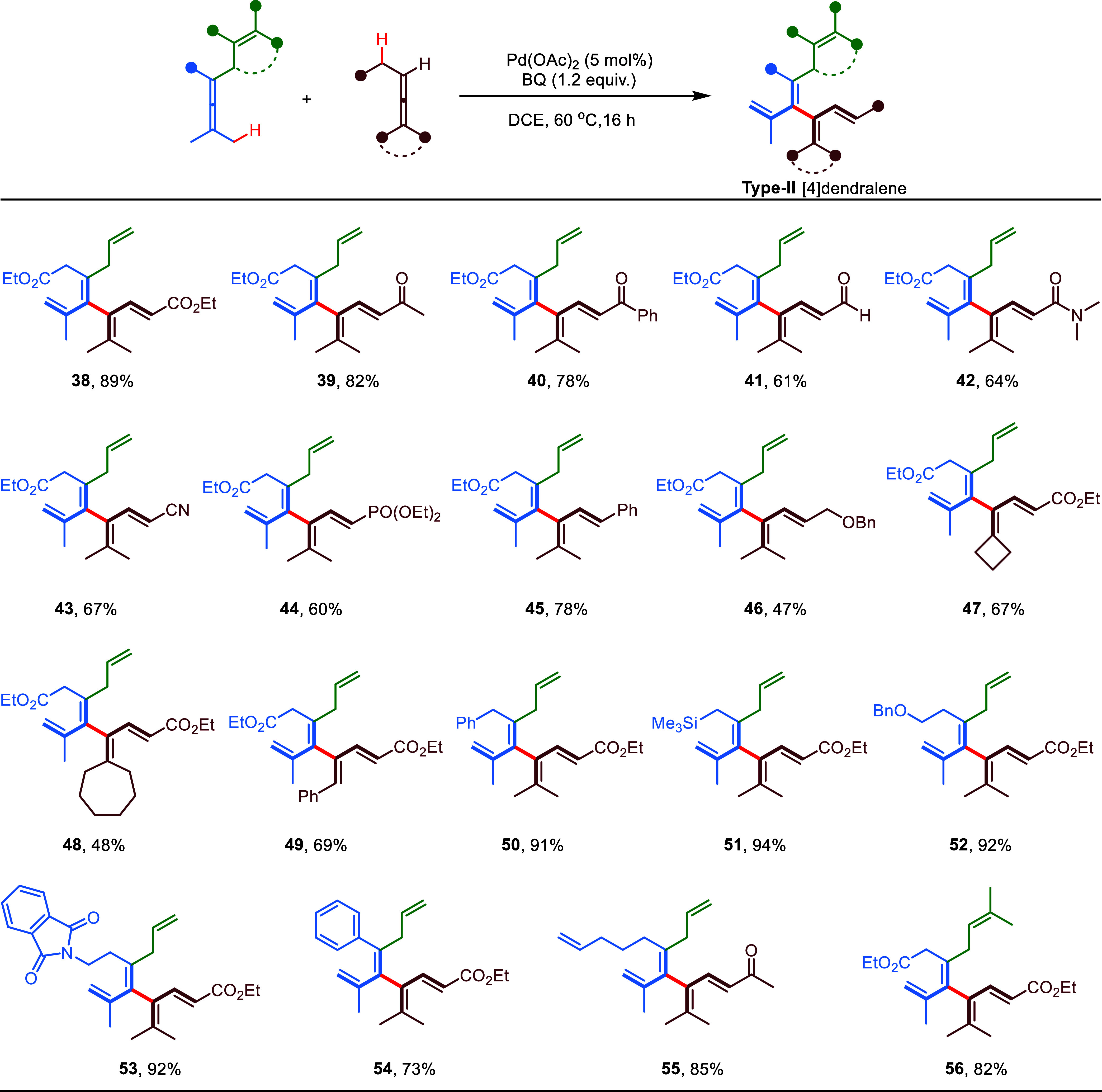
Reaction Scope: Formation of Type-II [4]Dendralenes Reaction conditions: enallene (0.2 mmol), directing group-free allene (1.5 equiv), Pd catalyst (5 mol %), and BQ (1.2 equiv) in 2.0 mL of DCE at 60 °C for 16 h, isolated yields.

We further expanded the scope of cross-coupling to various trisubstituted allenes bearing an allenic C–H bond adjacent to an electron-withdrawing group or phenyl (Scheme 2). Unlike the process of forming the aforementioned [4]dendralenes (**Type-I**) in Scheme 1, the generation of a new type of [4]dendralenes (**Type-II**) adopts a selective cleavage of the allenic C–H bond on the α carbon or benzylic carbon after intermolecular allene attack. Electron-withdrawing groups, including ester (**38**), ketone (**39**, **40**), aldehyde (**41**), amide (**42**), cyano (**43**), and phosphonate (**44**) were well-tolerated, affording the corresponding cross-coupling products in moderate to good yields (60–89%) with complete stereoselectivity. A high yield (78%) could also be obtained when an allene with a benzyl substituent was applied (**45**). Interestingly, two different types of [4]dendralenes (**46** versus **29**) were formed through the same enallene coupled with a pair of homologues of directing group-free allenes. Moreover, modulation of the two methyl groups on the trisubstituted allene to cycloalkyl (**47**, **48**) or phenyl (**49**) was feasible for the cross-allene coupling. Remarkably, good to excellent isolated yields (73–94%) of **Type-II** [4]dendralenes could be obtained when variations of substituents or directing olefin were applied to enallenes (**50**–**56**).

**Scheme 3:**
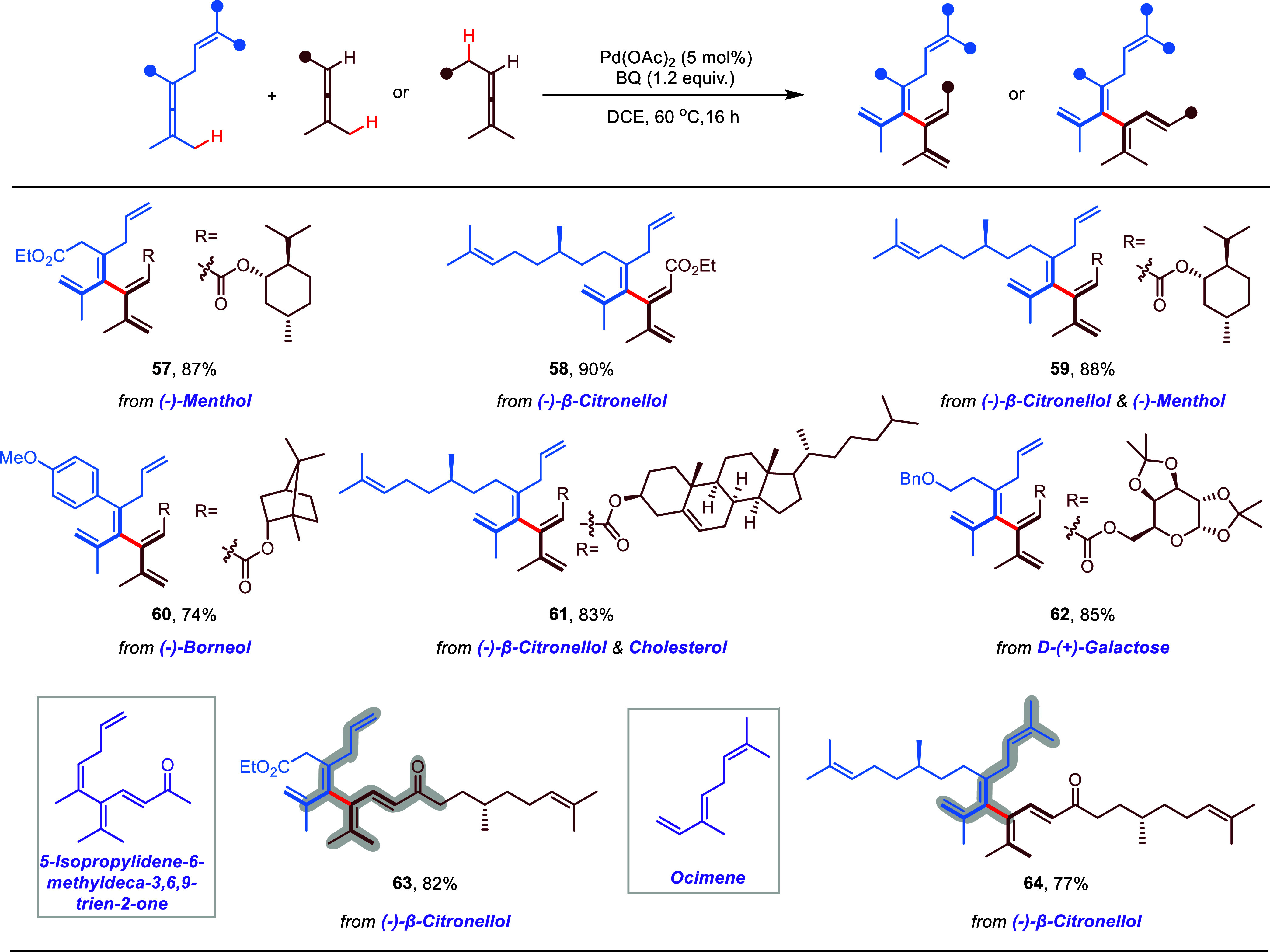
Free Combinations of Biorelevant Complex Allenes Reaction conditions: enallene (0.2 mmol), directing group-free allene (1.5 equiv), Pd catalyst (5 mol %), and BQ (1.2 equiv) in 2.0 mL of DCE at 60 °C for 16 h, isolated yields.

**Scheme 4:**
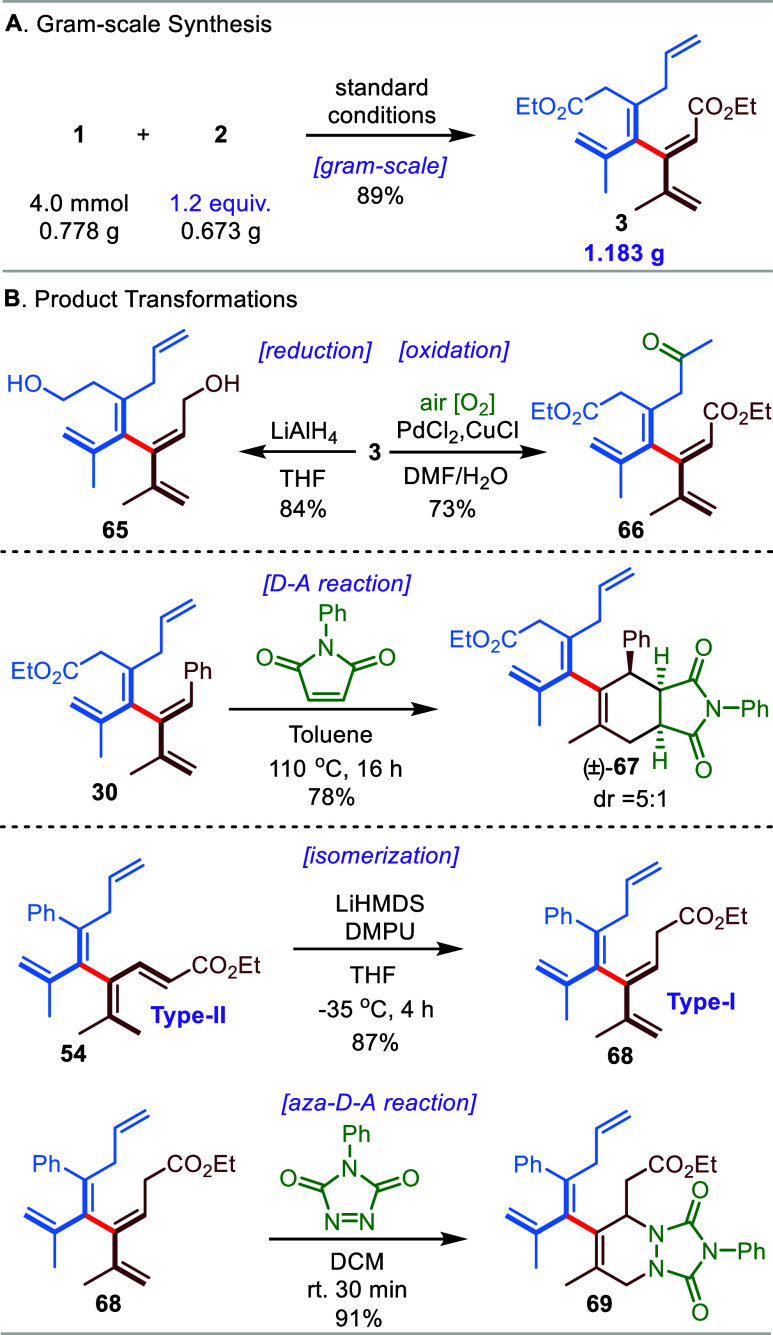
Gram-Scale Synthesis (A) and Product Transformations (B)

Late-stage C–H bond functionalization has served as a powerful strategy in drug discovery and chemical biology.^60−62^ To further evaluate the robustness and versatility of the newly developed single-functional group cross-coupling, late-stage functionalization of biorelevant molecules was performed (Scheme 3). We were delighted to find that a series of complex natural fragments including (−)-menthol (**57**, **59**), (−)-borneol (**60**), cholesterol (**61**), D-(+)-galactose (**62**), and (−)-β-citronellol (**58**, **59**, **61**, **63**, and **64**) could be incorporated into [4]dendralenes from either or both sides of coupled allenes in good yields (74–90%). Significantly, the core structures of both types of [4]dendralenes (**Type-I** and **Type-II**) synthesized via cross-allene coupling match with some interesting natural products, which further highlight the potential of the present methodology. For example, both [4]dendralenes **63** and **39** contain the structure of 5-isopropylidene-6-methyldeca-3,6,9-trien-2-one, a naturally occurring compound found in bioactive essential oil^63,64^ and honey;^65^ monoterpene ocimene structure can also be found in [4]dendralenes **64**, **56**, and **21**. These examples also demonstrate the advantage of using allyl as the internal assisting group for cross-coupling. The synthetic utility of the developed allene–allene cross-coupling was further demonstrated by a gram-scale reaction and product transformations (Scheme 4). First, diester [4]dendralene **3** was produced on a gram scale in 89% yield with a reduced excess of starting allene **2** (1.2 equiv) (Scheme 4A). Next, double reduction of diester **3** with lithium aluminum hydride occurred smoothly, affording diol [4]dendralene **65**.

Importantly, the directing allyl group inherited from the developed cross-coupling could be selectively converted to a ketone group via Wacker-Tsuji oxidation (from **3** to **66**, Scheme 4B, top). Moreover, the Diels–Alder addition of coupling product **30** with phenyl-substituted maleimide gave a high yield of adduct **67** with a 5:1 dr (diastereomeric ratio) (Scheme 4B, middle). Interestingly, a highly efficient base-mediated isomerization could be applied to transform **Type-II** [4]dendralene **54** to a new **Type-I** [4]dendralene **68**, which underwent a regioselective aza-Diels–Alder reaction with an aza dienophile at room temperature to give adduct **69** in a good yield (Scheme 4B, bottom).

**Scheme 5:**
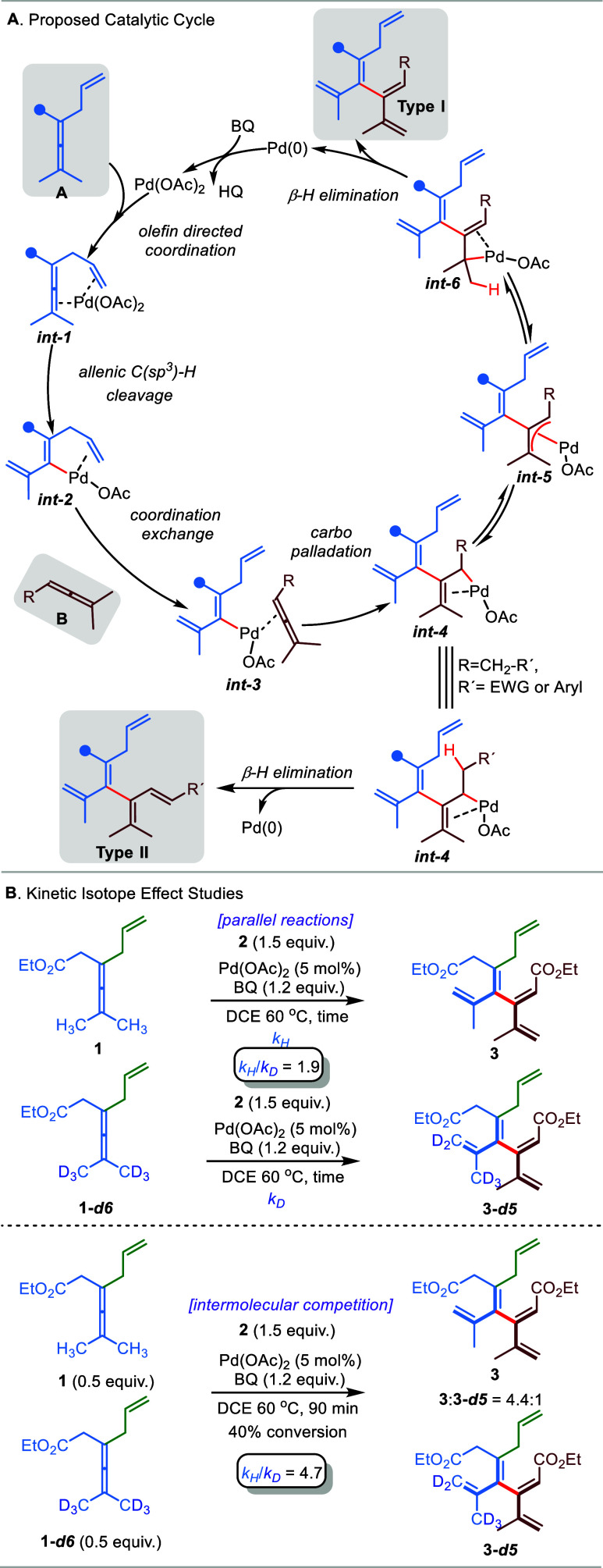
Proposed Catalytic Cycle (A) and Kinetic Isotope Effect Studies (B)

A plausible catalytic mechanism is proposed for the allene–allene cross-coupling in Scheme 5A. Olefin-directed coordination of the ′activated′ allene **A** (marked in blue) to Pd(OAc)_2_ forms **int-1**, which then undergoes allenic C(sp^3^)-H bond cleavage to generate vinylpalladium species **int-2**.^66^ Subsequently, the incoming directing group-free allene **B** (marked in brown) coordinates to Pd with the less sterically hindered double bond via ligand exchange with directing olefin to afford **int-3**, followed by carbopalladation, giving σ-allylpalladium intermediate **int-4**.^67^ When the R group does not provide protons for β-hydride elimination, a rearrangement occurs to give the π-allyl complex **int-5**, where the more stable configuration is formed with the R group *syn* to the newly formed C–C bond (marked in red). Formation of the other possible (σ-allyl)palladium complex (**int-6**) followed by β-hydride elimination will produce the **Type-I** [4]dendralene product observed, with the *Z* configuration of the trisubstituted double bond. A slightly less stereoselective formation of π-allyl complex **int-5** would lead to small amounts of another π-allyl isomer, where the R group is *anti* to the newly formed C–C bond. This minor π-allyl isomer would account for the minor isomer (*E*-configuration) in [4]dendralene products **26**, **27**, and **28** (Scheme 1). Finally, the generated Pd(0) is then reoxidized to Pd(II) by BQ, thus closing the catalytic cycle. In the case where the R group in σ-allyl complex **int-4** is CH_2_–EWG or CH_2_–Aryl, a β-hydride elimination takes place (lower part of Scheme 5A), which gives the **Type-II** [4]dendralene product.

Kinetic isotope effect (KIE) studies based on two parallel reactions as well as on an intermolecular competitive experiment were carried out for the formation of **Type-I** [4]dendralene product using **1** and **1-d6** (Scheme 5B; for details, see the Supporting Information). The corresponding absolute rate KIE value and competitive KIE value were determined as 1.9 and 4.7, respectively, which indicate that allenic C(sp^3^)-H cleavage of enallene is only partially rate-determining and that there is another step that comes into the overall rate. A similar level of KIE values (with an absolute rate KIE value of 2.0 and a competitive KIE value of 5.1) was obtained for the formation of **Type-II** [4]dendralene (see the Supporting Information for details).

## Conclusions

In summary, we have successfully developed an efficient oxidative allene–allene cross-coupling based on a directing group strategy, unlocking a new class of simple substrates for challenging cross-coupling. Utilizing simple reagents and mild reaction conditions, this approach provides unprecedented access to a diverse array of functionalized [4]dendralenes, which were previously difficult to synthesize. The practical transformations of the synthesized [4]dendralenes and subsequent late-stage modifications of biorelevant molecules underscore their potential in the total synthesis of natural products and drug discovery. Ongoing research in our laboratory focuses on further studies and applications of [4]dendralene chemistry, as well as gaining a deeper mechanistic understanding of the origin of the observed high cross-selectivity.

## Supporting Information Available

Experimental procedures; characterization data for all new compounds; NMR spectra; and additional relevant references (PDF)
