# Supplementary material for: Palladium-Catalyzed Oxidative Allene–Allene Cross-Coupling
Source: J Am Chem Soc. 2025 Jan 23;147(5):4338–48. doi: 10.1021/jacs.4c14607 (PMC11803718; doi:10.1021/jacs.4c14607)
Supplement: Supplementary file 1 — ja4c14607_si_001.pdf [file ja4c14607_si_001.pdf]

Supporting Information

Palladium-Catalyzed Oxidative Allene-Allene Cross-Coupling

Haibo Wu,[1] Qi Pan,[1] Judith Grill,[1] Magnus J. Johansson,[2] Youai Qiu,[3]* and Jan-E. Bäckvall[1]*

[1]Department of Organic Chemistry, Arrhenius Laboratory, Stockholm University, SE-106 91 Stockholm, Sweden.

[2]Medicinal Chemistry, Research and Early Development, Cardiovascular, Renal and Metabolism (CVRM), BioPharmaceuticals R&D, AstraZeneca Gothenburg, SE-43183 Mölndal, Sweden

[3]State Key Laboratory of Elemento-Organic Chemistry, Frontiers Science Center for New Organic Matter, Haihe Laboratory of Sustainable Chemical Transformations, College of Chemistry, Nankai University, Tianjin 300071, China

*Corresponding authors: Youai Qiu -Email: qiuyouai@nankai.edu.cn ; Jan-E. Bäckvall -Email: jeb@organ.su.se

Table of Contents

Table of Contents S1
General Information S2
1. Preparation of Starting Materials S3
   1.1 Preparation of Enallenes S3
   1.2 Preparation of Directing-group-free Allenes S11
2. Palladium-Catalyzed Allene-Allene Cross-Coupling S17
   2.1 General Procedure for Oxidative Allene-Allene Cross-Coupling S17
   2.2 General Procedure for Aerobic Version of Allene-Allene Cross-Coupling S17
   2.3 Characterization of **Type-I** [4]Dendralene Products S18
   2.4 Characterization of **Type-II** [4]Dendralene Products S36
   2.5 Negative Data S48
3. Gram-scale Synthesis and Products Transformations S49
   3.1 Gram-scale Synthesis S49
   3.2 Products Transformations S50
4. Kinetic Isotope Effect (KIE) Studies S54
   4.1 KIE Determined from Two Parallel Reactions S54
   4.2 Intermolecular Competition KIE Experiments S60
5. Determination of Stereochemistry of Compounds **3**, **30**, **38**, **49**, and **67** S63
6. NMR Spectra of New Compounds S75
7. References S167

General Information

All reagents were used directly from commercial suppliers, unless specified otherwise. The palladium-catalyzed cross allene coupling reactions were conducted without special precautions to exclude moisture and oxygen. Chromatographic separations were performed on Kieselgel 60 H silica gel (particle) size: 0.063-0.100 mm). Thin layer chromatography (TLC) was performed on aluminium plates coated with Kieselgel 60 (0.20 mm, UV254) and visualized under ultraviolet light (ν = 254 nm), or by staining with ethanolic phosphomolybdic acid and heating. ^{1}H NMR spectra were recorded at 400 MHz in Chloroform-*d* at 25°C and referenced internally to the residue $CHCl_3$ peak (7.26 ppm). ^{13}C NMR spectra were recorded at 101 MHz in Chloroform-*d* at 25 °C and referenced to the central peak of Chloroform-*d* (77.16 ppm). ^{19}F NMR spectra were recorded at 377 MHz in Chloroform-*d* at 25 °C. ^{31}P NMR spectra were recorded at 162 MHz in Chloroform-*d* at 25 °C. Chemical shifts are reported in ppm (δ scale). Coupling constants (*J*) are reported in Hertz (Hz). Multiplicity is denoted as: s (singlet), d (doublet), t (triplet), q (quartet), p (quintet), dd (doublet of doublets), br (broad) and m (multiplet). High-resolution mass spectra (HRMS) were obtained using electrospray ionization time-of-flight (ESI-TOF) methods.

1. *Preparation of Starting Materials*

1.1 Preparation of Enallenes

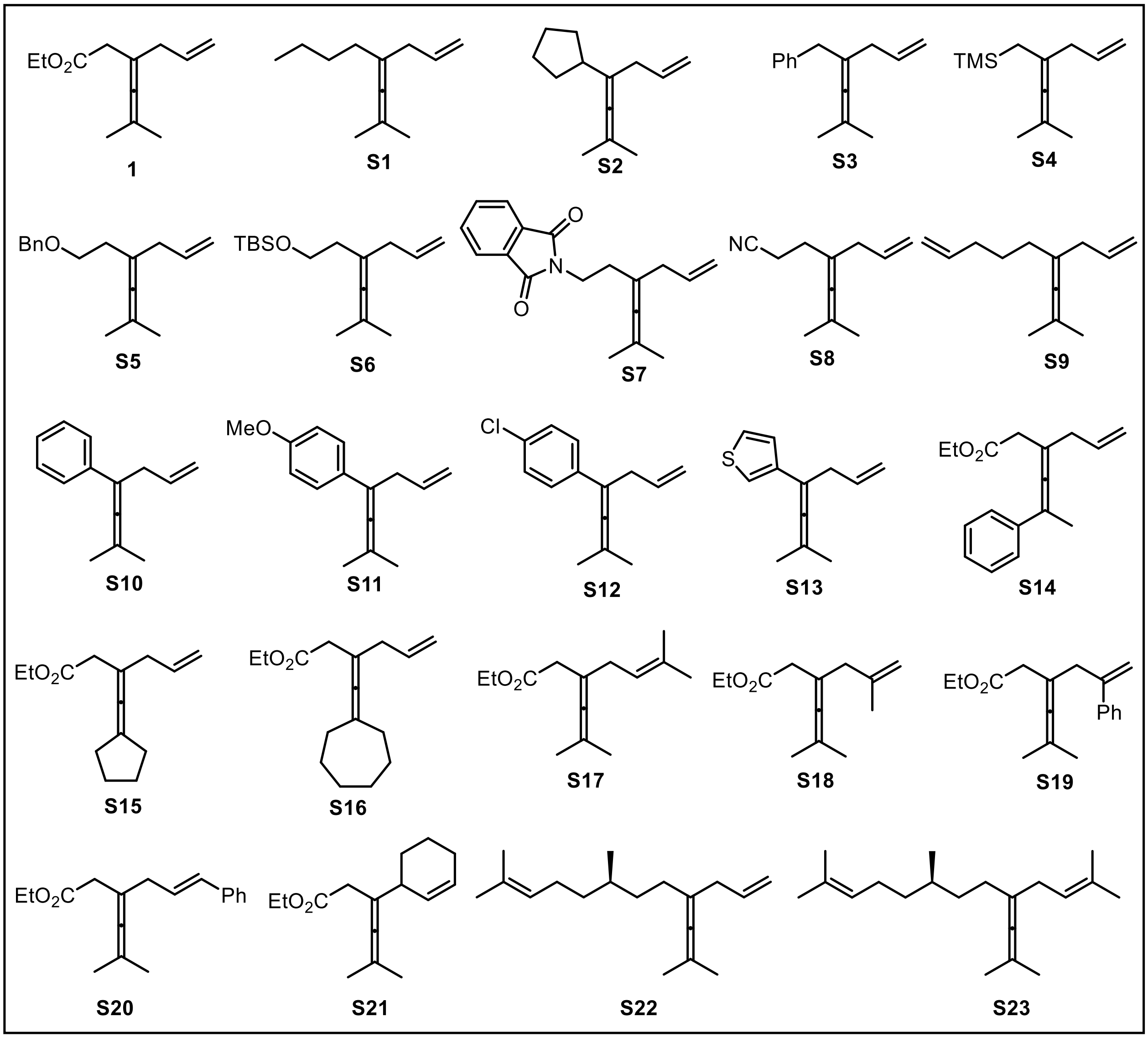

Figure S1. Eenallene substrates involved in this work.

Enallenes **1**,[1] **S1**,[2] **S3**,[2] **S5**,[1] **S10**,[3] **S14**,[4] **S15**,[5] **S16**[6] and **S18**[2] are known compounds, which were prepared according to reported procedures.

Enallenes **S2**, **S4**, **S9**, **S11**, **S12**, **S13**, **S22**, **S23** were prepared using the ***General procedure 1 (GP1)***.

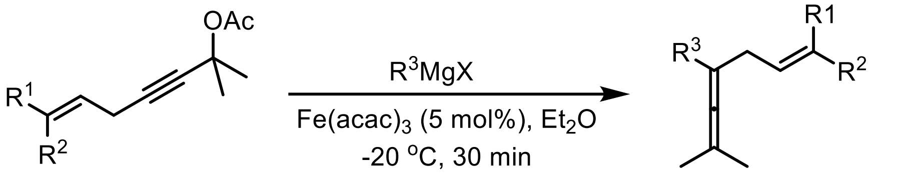

S2, **S4**, **S9**, **S11**, **S12**, **S13**, **S22**, **S23**

Scheme S1. General procedure for the preparation of enallenes **S2**, **S4**, **S9**, **S11**, **S12**, **S13**, **S22**, **S23**.

General procedure 1 (GP1): Iron-catalyzed synthesis of allenes from propargylic acetate and Grignard reagents was based on a previously reported procedure[7]: To a solution of $Fe(acac)_3$ (5 mol%) and propargylic acetate in dry Et_2O (0.2 M) was added Grignard reagent (1.5 equiv.) dropwise at -20 °C under nitrogen. The resulting mixture was stirred at -20 °C for 30 min before quenching with 10% citric acid solution (1 mL/mmol) and extraction with Et_2O three times. The combined organic extracts were dried over Na_2SO_4, filtered, and concentrated under reduced pressure. The residue was purified using column chromatography on silica gel to afford the desired enallene.

(6-Methylhepta-1,4,5-trien-4-yl) cyclopentane

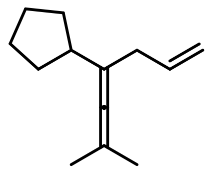

S2

Isolated yield: 51%, eluent: pentane (100%), colorless oil. ^{1}H NMR (400 MHz, Chloroform-*d*) δ 5.81 (ddt, *J* = 16.9, 10.1, 6.7 Hz, 1H), 5.08 – 4.92 (m, 2H), 2.70 (d, *J* = 6.7 Hz, 2H), 2.29 (p, *J* = 7.5 Hz, 1H), 1.78 – 1.69 (m, 2H), 1.66 (s, 6H), 1.64 – 1.49 (m, 4H), 1.44 – 1.33 (m, 2H). ^{13}C NMR (101 MHz, Chloroform-*d*) δ 198.16, 137.43, 114.85, 105.00, 96.56, 42.59, 37.42, 32.00, 25.03, 21.08. HRMS-ESI: Found [M+Na]$^+$ =199.1662; $C_{13}H_{20}Na$ requires 199.1659.

(2-Allyl-4-methylpenta-2,3-dien-1-yl)trimethylsilane

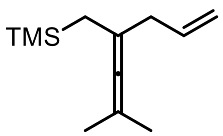

S4

Isolated yield: 93%, eluent: pentane (100%), colorless oil. ^{1}H NMR (400 MHz, Chloroform-*d*) δ 5.78 (ddt, *J* = 16.9, 10.1, 6.8 Hz, 1H), 5.07 – 4.93 (m, 2H), 2.63 (d, *J* = 6.8 Hz, 2H), 1.65 (s, 6H), 0.02 (s, 9H). ^{13}C NMR (101 MHz, Chloroform-*d*) δ 199.84, 137.09, 115.14, 97.35, 94.51, 40.65, 21.59, 21.31, -1.13. HRMS-ESI: Found [M+Na]$^+$ =217.1383; $C_{12}H_{22}SiNa$ requires 217.1387.

6-Allyl-8-methylnona-1,6,7-triene

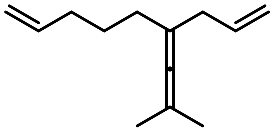

S9

Isolated yield: 93%, eluent: pentane (100%), colorless oil. ^{1}H NMR (400 MHz, Chloroform-*d*) δ 5.89 – 5.72 (m, 2H), 5.09 – 4.89 (m, 4H), 2.66 (dt, *J* = 6.7, 1.4 Hz, 2H), 2.11 – 2.03 (m, 2H), 1.91 (t, *J* = 7.4 Hz, 2H), 1.67 (s, 6H), 1.48 (p, *J* = 7.5 Hz, 2H). ^{13}C NMR (101 MHz, Chloroform-*d*) δ 199.26, 139.25, 115.13, 114.41, 100.09, 95.88, 38.19, 33.54, 31.99, 27.13, 21.09. HRMS-ESI: Found [M+Na]$^+$ =199.1463; $C_{13}H_{20}Na$ requires 199.1458.

1-Methoxy-4-(6-methylhepta-1,4,5-trien-4-yl)benzene

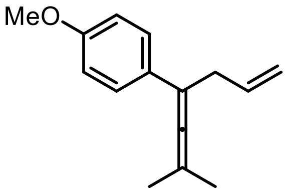

S11

Isolated yield: 66%, eluent: Et_2O/pentane (5: 95), pale yellow oil. ^{1}H NMR (400 MHz, Chloroform-*d*) δ 7.32 – 7.27 (m, 2H), 6.89 – 6.81 (m, 2H), 6.00 – 5.85 (m, 1H), 5.18 – 5.00 (m, 2H), 3.80 (s, 3H), 3.17 – 3.10 (m, 2H), 1.79 (s, 6H). ^{13}C NMR (101 MHz, Chloroform-*d*) δ 201.81, 158.30, 136.73, 130.41, 127.24, 115.59, 113.84, 101.36, 98.45, 55.45, 35.43, 20.66. HRMS-ESI: Found [M+Na]$^+$ =237.1247; $C_{15}H_{18}ONa$ requires 237.1250.

1-Chloro-4-(6-methylhepta-1,4,5-trien-4-yl)benzene

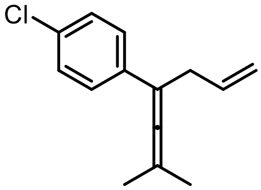

S12

Isolated yield: 73%, eluent: Et_2O/pentane (1: 99), pale yellow oil. ^{1}H NMR (400 MHz, Chloroform-*d*) δ 7.30 – 7.23 (m, 4H), 5.90 (ddt, *J* = 16.7, 10.1, 6.3 Hz, 1H), 5.18 – 5.01 (m, 2H), 3.13 (dt, *J* = 6.3, 1.5 Hz, 2H), 1.80 (s, 6H). ^{13}C NMR (101 MHz, Chloroform-*d*) δ 202.51, 136.58, 136.23, 131.91, 128.42, 127.43, 115.93, 101.08, 99.10, 35.18, 20.45. HRMS-ESI: Found [M+Na]$^+$ =241.0766; $C_{14}H_{15}ClNa$ requires 241.0762.

3-(6-Methylhepta-1,4,5-trien-4-yl)thiophene

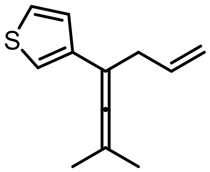

S13

Isolated yield: 60%, eluent: Et_2O/pentane (5: 95), pale yellow oil. ^{1}H NMR (400 MHz, Chloroform-*d*) δ 7.12 (dd, *J* = 5.1, 1.1 Hz, 1H), 6.94 (dd, *J* = 5.1, 3.6 Hz, 1H), 6.88 (dd, *J* = 3.6, 1.2 Hz, 1H), 5.92 (ddt, *J* = 16.7, 10.1, 6.5 Hz, 1H), 5.21 – 5.02 (m, 2H), 3.13 (dt, *J* = 6.5, 1.5 Hz, 2H), 1.80 (s, 6H). ^{13}C NMR (101 MHz, Chloroform-*d*) δ 201.29, 143.91, 136.04, 127.42, 123.91, 122.47, 116.01, 99.67, 98.02, 36.35, 20.53. HRMS-ESI: Found [M+H]$^+$ =191.0907; $C_{12}H_{15}S$ requires 191.0889.

(*R*)-4-Allyl-2,7,11-trimethyldodeca-2,3,10-triene

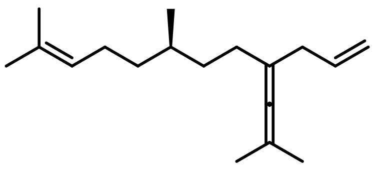

S22

Isolated yield: 72%, eluent: pentane (100%), colorless oil. ^{1}H NMR (400 MHz, Chloroform-*d*) δ 5.80 (ddt, *J* = 16.9, 10.0, 6.8 Hz, 1H), 5.16 – 4.93 (m, 3H), 2.66 (d, *J* = 6.5 Hz, 2H), 2.06 – 1.82 (m, 4H), 1.69 (s, 3H), 1.66 (s, 6H), 1.61 (s, 3H), 1.50 – 1.28 (m, 3H), 1.25 – 1.08 (m, 2H), 0.86 (d, *J* = 6.6 Hz, 3H). ^{13}C NMR (101 MHz, Chloroform-*d*) δ 199.25, 137.16, 131.09, 125.25, 115.06, 100.56, 95.67, 38.19, 37.32, 35.00, 32.09, 30.11, 25.82 (d, *J* = 12.3 Hz), 21.10, 19.66, 17.76. HRMS-ESI: Found [M+Na]$^+$ =269.2247; $C_{18}H_{30}Na$ requires 269.2240.

(*R*)-2,8,12-Trimethyl-5-(2-methylprop-1-en-1-ylidene)trideca-2,11-diene

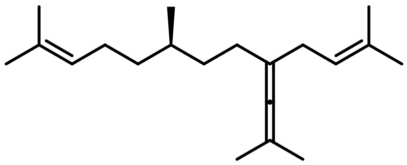

S23

Isolated yield: 75%, eluent: pentane (100%), colorless oil. ^{1}H NMR (400 MHz, Chloroform-*d*) δ 5.21 – 5.06 (m, 2H), 2.60 (d, *J* = 7.2 Hz, 2H), 1.70 (d, *J* = 1.4 Hz, 3H), 1.68 (d, *J* = 1.5 Hz, 3H), 1.65 (s, 6H), 1.61 (d, *J* = 1.3 Hz, 3H), 1.60 (d, *J* = 1.3 Hz, 3H), 1.49 – 1.26 (m, 3H), 1.24 – 1.07 (m, 2H), 0.86 (d, *J* = 6.4 Hz, 3H). ^{13}C NMR (101 MHz, Chloroform-*d*) δ 198.94, 132.29, 131.06, 125.27, 122.67, 101.49, 95.28, 37.35, 35.11, 32.42, 32.12, 30.15, 25.88, 25.77, 21.17, 19.65, 17.92, 17.76. HRMS-ESI: Found [M+Na]$^+$ =297.2249; $C_{20}H_{34}Na$ requires 297.2252.

Enallenes **S6**, **S7**, **S8** were prepared using the following procedures.

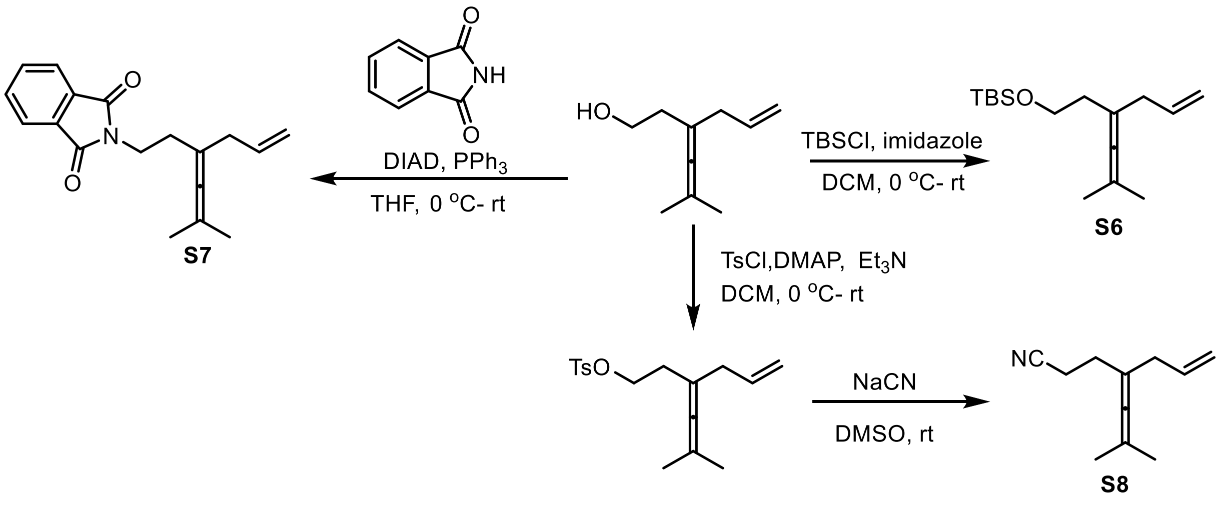

Scheme S2. Synthesis of enallenes **S6**, **S7**, **S8**.

Synthesis of enallenes S6:[1] To a solution of enallenol (152 mg, 1.0 mmol, 1.0 equiv.) and imidazaole (102 mg, 1.5 mmol, 1.5 equiv.) in 5 mL of DCM was added TBSCl (181 mg, 1.2 mmol, 1.2 equiv.) at 0 °C. The reaction mixture was stirred at room temperature overnight. After full consumption of starting material as monitored by TLC, the reaction mixture was concentrated *in vacuo* and purified via column chromatography on silica gel to afford the corresponding product enallene **S6.**

((3-Allyl-5-methylhexa-3,4-dien-1-yl)oxy)(tert-butyl)dimethylsilane

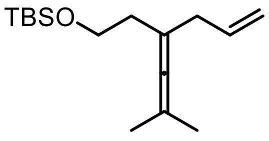

S6

Isolated yield: 95%, eluent: Et_2O/pentane (5: 95), colorless oil. ^{1}H NMR (400 MHz, Chloroform-*d*) δ 5.78 (ddt, *J* = 16.9, 10.0, 6.7 Hz, 1H), 5.09 – 4.94 (m, 2H), 3.65 (t, *J* = 7.3 Hz, 2H), 2.68 (d, *J* = 6.7 Hz, 2H), 2.14 (t, *J* = 7.3 Hz, 2H), 1.65 (s, 6H), 0.89 (s, 9H), 0.05 (s, 6H). ^{13}C NMR (101 MHz, Chloroform-*d*) δ 199.61, 136.83, 115.32, 97.09, 95.76, 62.50, 38.39, 36.01, 26.13, 21.07, 18.51, -5.10. Found $[M+Na]^+$ =213.1956; $C_{16}H_{30}OSiNa$ requires 289.1958.

Synthesis of enallenes S7: To a mixture of PPh_3 (393 mg, 1.5 mmol 1.5 equiv.), DIAD (di-isopropyl azodicarboxylate) (303 mg, 1.5 mmol, 1.5 equiv.), and phthalimide (221 mg, 1.5 mmol, 1.5 equiv.) was added a solution of enallenol (152 mg, 1.0 mmol, 1.0 equiv.) in THF (5 mL) at 0 °C. The resulting mixture was then stirred at room temperature overnight. After full consumption of starting material as monitored by TLC, the reaction mixture was concentrated *in vacuo* and purified via column chromatography on silica gel to afford the corresponding product enallene **S7**.

2-(3-Allyl-5-methylhexa-3,4-dien-1-yl)isoindoline-1,3-dione

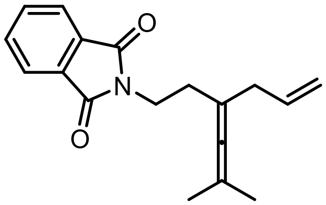

S7

Isolated yield: 90%, eluent: EtOAc/pentane (10: 95), white powder. ^{1}H NMR (400 MHz, Chloroform-*d*) δ 7.85 – 7.79 (m, 2H), 7.72 – 7.65 (m, 2H), 5.83 – 5.68 (m, 1H), 5.00 (dd, *J* = 25.7, 13.6 Hz, 2H), 3.74 (t, *J* = 7.2 Hz, 2H), 2.71 (d, *J* = 6.6 Hz, 2H), 2.28 (t, *J* = 7.3 Hz, 2H), 1.51 (s, 6H). ^{13}C NMR (101 MHz, Chloroform-*d*) δ 199.73, 168.37, 136.41, 133.91, 132.34, 123.18, 115.68, 96.79, 37.83, 36.63, 30.99, 20.86. HRMS-ESI: Found $[M+Na]^+$ =304.1308; $C_{18}H_{19}NO_2Na$ requires 304.1308.

Synthesis of enallenes S8: The tosylate is a known compound,[4] which was used for the synthesis of enallene **S8**. To a solution of the tosylate (307 mg, 1.0 mmol, 1.0 equiv.) in 2 mL of DMSO was

added NaCN (60 mg, 1.2 mmol, 1.2 equiv.). The resulting mixture was stirred at room temperature for 2 days and then diluted with 10 mL of H_2O and extracted with Et_2O (10 mL x 3). The combined organic extracts were dried over Na_2SO_4, filtered, and concentrated under reduced pressure. The residue was purified using column chromatography on silica gel to afford the desired enallene **S8**.

4-Allyl-6-methylhepta-4,5-dienenitrile

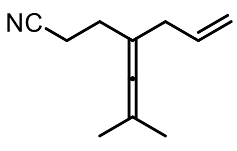

S8

Isolated yield: 96%, eluent: Et_2O/pentane (1: 99), colorless oil. ^{1}H NMR (400 MHz, Chloroform-*d*) δ 5.77 (ddt, *J* = 16.8, 10.1, 6.7 Hz, 1H), 5.12 – 4.98 (m, 2H), 2.70 (d, *J* = 6.7 Hz, 2H), 2.40 (t, *J* = 6.9 Hz, 2H), 2.22 (t, *J* = 7.0 Hz, 2H), 1.72 (s, 6H). ^{13}C NMR (101 MHz, Chloroform-*d*) δ 198.72, 135.95, 119.95, 116.08, 99.56, 98.04, 38.11, 27.91, 20.95, 15.86. HRMS-ESI: Found $[M+Na]^+$ =184.1102; $C_{11}H_{15}NNa$ requires 184.1097.

Enallenes **S17**, **S19**, **S20**, **S21** were prepared using the ***General procedure 2 (GP2)***.

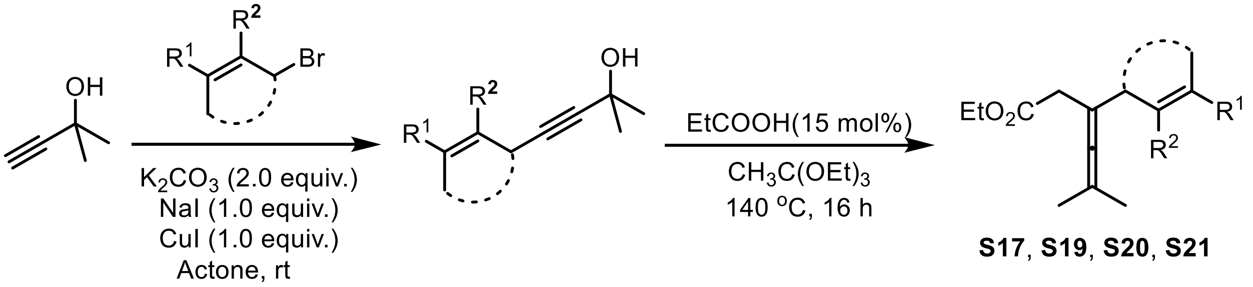

Scheme S3. General procedure for the preparation of enallenes **S17**, **S19**, **S20**, **S21**.

General procedure 2 (GP2): Step 1 (Allylation of propargyl alcohol): To a solution of 2-methylbut-3-yn-2-ol (1.68 g, 20 mmol) in 80 mL of acetone was added K_2CO_3 (5.50 g, 40 mmol, 2.0 equiv), NaI (3.0 g, 20 mmol, 1.0 equiv.), CuI (3.80 g, 20 mmol, 1.0 equiv.), and allyl bromide (24 mmol, 1.2 equiv.) sequentially. The reaction mixture was stirred at room temperature for 16 h, and then filtered to remove inorganic salts. After evaporation, 100 mL of Et_2O was added to the residue, and the mixture was filtered via silica gel (2 cm). The solvent was evaporated and the residue was purified using column chromatography on silica gel to afford the desired allylated propargyl alcohol.

Step 2 (Johnson–Claisen rearrangement): A dry round-bottomed flask containing crude propargylic alcohol (10 mmol) was equipped with a distillation receiver and a condenser. Triethyl

orthoacetate (20 ml) and propanoic acid (111 mg, 1.5 mmol, 0.15 equiv.) were added sequentially. The reaction was then refluxed at 140°C for 16 h before cooling down to 0 °C. Et_2O (100 mL) and HCl (aq., 1 M, 50 mL) were added and the layers were separated. The aqueous layer was extracted with Et_2O (50 mL x 2). The combined organic layers were dried over Na_2SO_4, filtered, evaporated. The residue was purified using column chromatography on silica gel to afford the desired enallene.

Ethyl 6-methyl-3-(2-methylprop-1-en-1-ylidene)hept-5-enoate

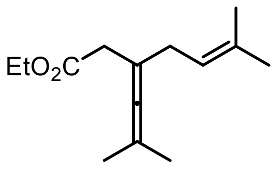

S17

Isolated yield: 52% over two steps, eluent: Et_2O/pentane (10: 90), colorless oil. ^{1}H NMR (400 MHz, Chloroform-*d*) δ 5.14 (tt, *J* = 7.3, 1.3 Hz, 1H), 4.12 (q, *J* = 7.1 Hz, 2H), 2.89 (s, 2H), 2.69 (d, *J* = 7.3 Hz, 2H), 1.70 (s, 3H), 1.66 (s, 6H), 1.61 (s, 3H), 1.25 (t, *J* = 7.1 Hz, 3H). ^{13}C NMR (101 MHz, Chloroform-*d*) δ 200.37, 171.99, 133.46, 121.64, 96.41, 95.22, 60.55, 39.03, 32.20, 25.86, 20.80, 17.87, 14.37. Found [M+Na]$^+$ =245.1512; $C_{14}H_{22}O_2Na$ requires 245.1512.

Ethyl 5-methyl-3-(2-phenylallyl)hexa-3,4-dienoate

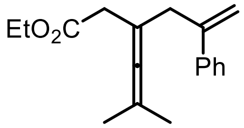

S19

Isolated yield: 58% over two steps, eluent: Et_2O/pentane (10: 90), colorless oil. ^{1}H NMR (400 MHz, Chloroform-*d*) δ 7.45 – 7.41 (m, 2H), 7.32 – 7.27 (m, 2H), 7.25 – 7.21 (m, 1H), 5.40 (d, *J* = 1.5 Hz, 1H), 5.12 (q, *J* = 1.3 Hz, 1H), 4.12 (q, *J* = 7.1 Hz, 2H), 3.26 (d, *J* = 1.1 Hz, 2H), 2.91 (s, 2H), 1.48 (s, 6H), 1.24 (t, *J* = 7.1 Hz, 3H). ^{13}C NMR (101 MHz, Chloroform-*d*) δ 201.95, 171.82, 145.49, 140.63, 128.18, 127.36, 126.39, 114.28, 96.66, 93.70, 60.59, 39.79, 38.63, 20.21, 14.34. HRMS-ESI: Found [M+Na]$^+$ =293.1524; $C_{18}H_{22}O_2Na$ requires 293.1512.

Ethyl 3-cinnamyl-5-methylhexa-3,4-dienoate

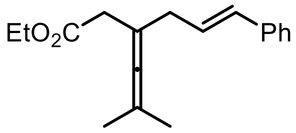

S20

Isolated yield: 43% over two steps, eluent: Et_2O/pentane (10: 90), pale yellow oil. ^{1}H NMR (400 MHz, Chloroform-*d*) δ 7.39 – 7.25 (m, 4H), 7.25 – 7.16 (m, 1H), 6.42 (dd, *J* = 15.8, 1.5 Hz, 1H), 6.17 (dt, *J* = 15.8, 7.0 Hz, 1H), 4.12 (q, *J* = 7.1 Hz, 2H), 2.96 (s, 2H), 2.92 (dd, *J* = 7.0, 1.4 Hz, 2H), 1.69 (s, 6H), 1.25 (t, *J* = 7.1 Hz, 3H). ^{13}C NMR (101 MHz, Chloroform-*d*) δ 200.89, 171.82, 137.81, 131.36, 128.61, 128.01, 127.14, 126.22, 96.91, 94.36, 60.64, 39.01, 37.11, 20.79, 14.36. HRMS-ESI: Found [M+Na]$^+$ =293.1509; $C_{18}H_{22}O_2Na$ requires 293.1512.

Ethyl 3-(cyclohex-2-en-1-yl)-5-methylhexa-3,4-dienoate

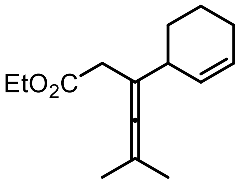

S21

Isolated yield: 48% over two steps, eluent: Et_2O/pentane (10: 90), colorless oil. ^{1}H NMR (400 MHz, Chloroform-*d*) δ 5.74 – 5.65 (m, 1H), 5.60 (dq, *J* = 10.1, 2.4 Hz, 1H), 4.12 (q, *J* = 7.1 Hz, 2H), 2.95 (s, 2H), 2.76 – 2.66 (m, 1H), 1.96 (tq, *J* = 5.6, 2.6 Hz, 2H), 1.84 – 1.69 (m, 2H), 1.67 (s, 3H), 1.66 (s, 3H), 1.60 – 1.42 (m, 2H), 1.25 (t, *J* = 7.1 Hz, 3H). ^{13}C NMR (101 MHz, Chloroform-*d*) δ 200.48, 172.12, 129.42, 127.65, 99.68, 97.59, 60.58, 38.96, 37.94, 28.13, 25.29, 21.07, 20.84, 20.66, 14.36. HRMS-ESI: Found $[M+Na]^+$ =257.1513; $C_{15}H_{22}O_2Na$ requires 257.1512.

1.2 Preparation of Directing-group-free Allenes

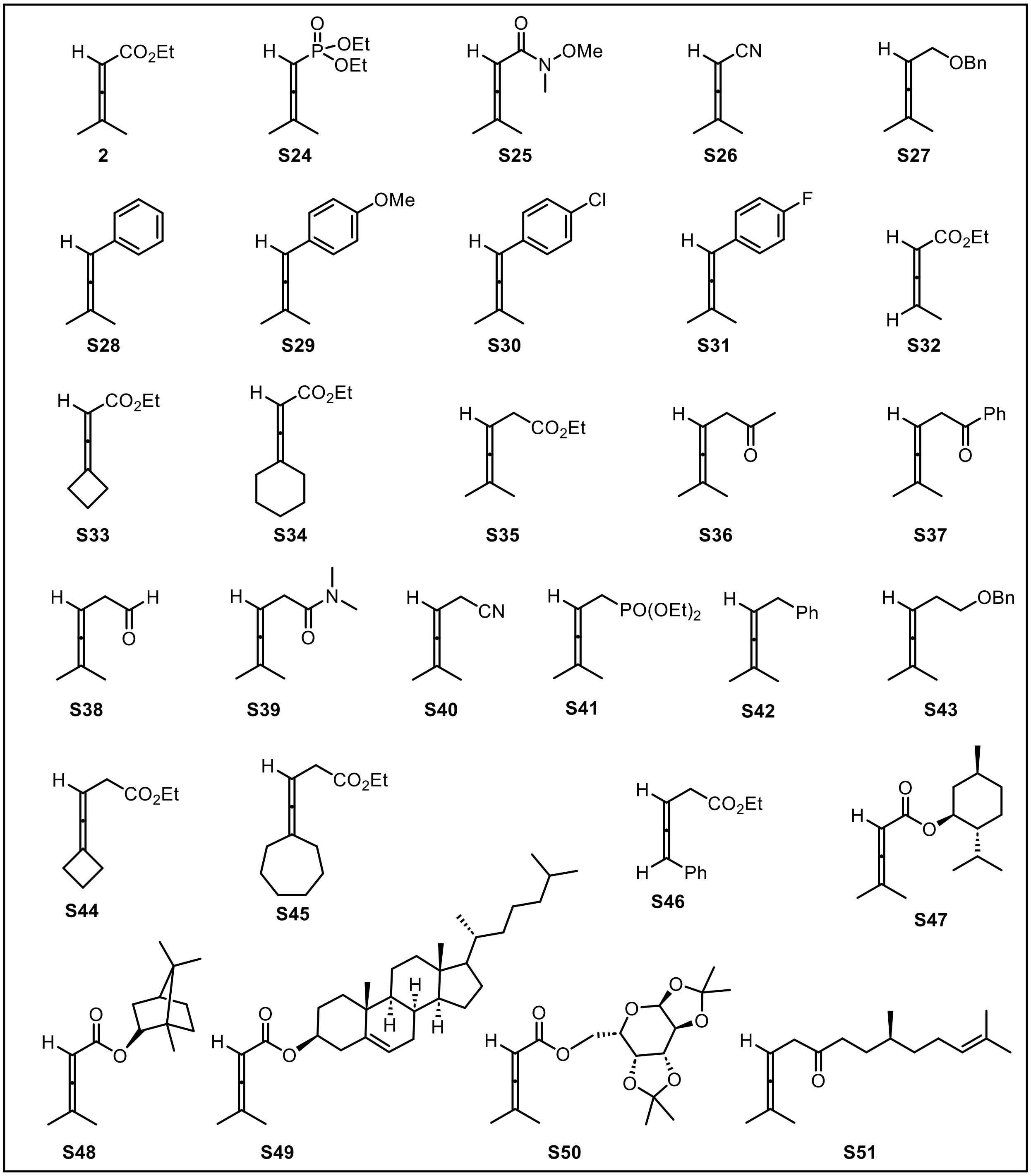

Figure S2. Directing-group-free allenes involved in this work.

Directing-group-free allenes **2**[8], **S24**[9], **S27**[10], **S28**[11], **S29**[11], **S30**[12], **S31**[11], **S32**[13], **S34**[14], **S35**[15], **S36**[16], **S37**[17], **S38**[18], **S39**[19], **S41**[20], **S42**[21], **S43**[22], **S45**[23] and **S46**[24] are known compounds, which were prepared using the reported procedures.

Directing-group-free allenes **S25**, **S26**, **S33**, **S47**, **S48**, **S49** and **S50** were prepared using the ***General procedure 3 (GP3)***.

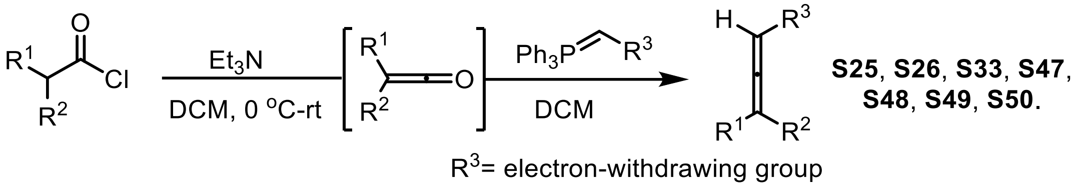

Scheme S3. General procedure for the preparation of allenes **S25**, **S26**, **S33**, **S47**, **S48**, **S49** and **S50.**

General procedure 3 (GP3) (allene formation via a Wittig reaction[8]): To a stirring solution of acyl chloride (10.5 mmol, 1.05 equiv.) in 90 mL of DCM under an atmosphere of nitrogen was added Et_3N (1.6 mL, 11.5 mmol, 1.15 equiv.) dropwise at 0 °C. The resulting mixture was stirred at room temperature for 30 min and then a solution of Wittig reagent (10 mmol, 1.0 equiv.) in 50 mL of DCM was added dropwise to the reaction at room temperature via an additional funnel and stirred overnight. The reaction mixture was concentrated under reduced pressure followed by the addition of 50 mL of Et_2O to form a slurry, which was filtered through a pad of silica gel and washed with 20 mL of Et_2O. The filtrate was concentrated under reduced pressure and the residue was purified using column chromatography on silica gel to afford the desired allene.

N-Methoxy-N,4-dimethylpenta-2,3-dienamide

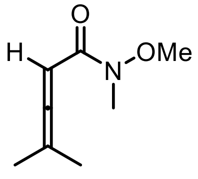

S25

Isolated yield: 42%, eluent: EtOAc/pentane (10: 90), colorless oil. ^{1}H NMR (400 MHz, Chloroform-*d*) δ 6.03 – 5.95 (m, 1H), 3.70 (s, 3H), 3.22 (s, 3H), 1.81 – 1.78 (m, 6H). ^{13}C NMR (101 MHz, Chloroform-*d*) δ 210.51, 166.68, 100.05, 84.33, 61.71, 32.77, 19.58. HRMS-ESI: Found $[M+Na]^+$ =178.0837; $C_8H_{13}NO_2Na$ requires 178.0838.

4-Methylpenta-2,3-dienenitrile

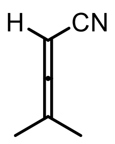

S26

Isolated yield: 55%, eluent: Et_2O/pentane (2: 98), colorless oil. ^{1}H NMR (400 MHz, Chloroform-*d*) δ 5.07 (hept, *J* = 3.0 Hz, 1H), 1.80 (d, *J* = 2.9 Hz, 6H). ^{13}C NMR (101 MHz, Chloroform-*d*) δ 213.79, 114.48, 102.29, 65.23, 19.28. HRMS-ESI: Found $[M+Na]^+$ =116.0478; C_6H_7NNa requires 116.0471.

Ethyl 3-cyclobutylideneacrylate

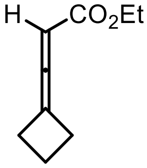

S33

Isolated yield: 37%, eluent: Et_2O/pentane (5: 95), colorless oil. ^{1}H NMR (400 MHz, Chloroform-*d*) δ 5.54 (p, *J* = 4.2 Hz, 1H), 4.18 (q, *J* = 7.1 Hz, 2H), 3.14 – 2.88 (m, 4H), 2.13 – 1.96 (m, 2H), 1.28 (t, *J* = 7.1 Hz, 3H). ^{13}C NMR (101 MHz, Chloroform-*d*) δ 203.68, 166.67, 105.34, 89.56, 60.81, 28.98, 17.83, 14.44. HRMS-ESI: Found $[M+Na]^+$ =175.0728; $C_9H_{12}O_2Na$ requires 175.0730.

(1*S*,2*R*,5*S*)-2-Isopropyl-5-methylcyclohexyl 4-methylpenta-2,3-dienoate

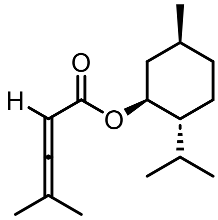

S47

Isolated yield: 62%, eluent: Et_2O/pentane (5: 95), colorless oil. ^{1}H NMR (400 MHz, Chloroform-*d*) δ 5.42 (hept, *J* = 2.8 Hz, 1H), 4.69 (td, *J* = 10.9, 4.4 Hz, 1H), 2.07 – 2.00 (m, 1H), 1.91 – 1.82 (m, 1H), 1.79 (dd, *J* = 6.1, 2.8 Hz, 6H), 1.72 – 1.63 (m, 2H), 1.55 – 1.44 (m, 1H), 1.43 – 1.34 (m, 1H), 1.12 – 0.93 (m, 2H), 0.89 (dd, *J* = 6.8, 5.0 Hz, 6H), 0.78 (d, *J* = 7.0 Hz, 3H). ^{13}C NMR (101 MHz, Chloroform-*d*) δ 210.78, 166.39, 100.06, 86.55, 74.56, 47.25, 41.08, 34.47, 31.51, 26.74, 24.07, 22.18, 20.74, 19.50, 19.45, 16.95. HRMS-ESI: Found $[M+Na]^+$ =273.1827; $C_{16}H_{26}O_2Na$ requires 273.1825.

(1*R*,2*R*,4*S*)-1,7,7-Trimethylbicyclo[2.2.1]heptan-2-yl 4-methylpenta-2,3-dienoate

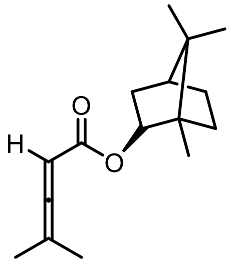

S48

Isolated yield: 62%, eluent: Et_2O/pentane (5: 95), colorless oil. ^{1}H NMR (400 MHz, Chloroform-*d*) δ 5.44 (hept, *J* = 2.8 Hz, 1H), 4.89 (ddd, *J* = 9.9, 3.5, 2.0 Hz, 1H), 2.42 – 2.29 (m, 1H), 1.94 (qt, *J* = 7.1, 3.2 Hz, 1H), 1.80 (dd, *J* = 2.8, 1.8 Hz, 6H), 1.77 – 1.65 (m, 2H), 1.33 – 1.18 (m, 2H), 1.00 (dd, *J* = 13.8, 3.5 Hz, 1H), 0.90 (s, 3H), 0.87 (s, 3H), 0.83 (s, 3H). ^{13}C NMR (101 MHz, Chloroform-*d*) δ 210.84, 167.14, 100.06, 86.53, 80.06, 49.01, 47.86, 45.10,

36.94, 28.15, 27.15, 19.85, 19.40, 19.02, 13.63. HRMS-ESI: Found $[M+Na]^+$ =271.1670; $C_{16}H_{24}O_2Na$ requires 271.1669.

4-Methylpenta-2,3-dienoate(3*S*,8*R*,9*S*,10*R*,13*R*,14*S*)-10,13-dimethyl-17-((R)-6-methylheptan-2-yl)-2,3,4,7,8,9,10,11,12,13,14,15,16,17-tetradecahydro-1H-cyclopenta[a]phenanthren-3-yl 4-methylpenta-2,3-dienoate

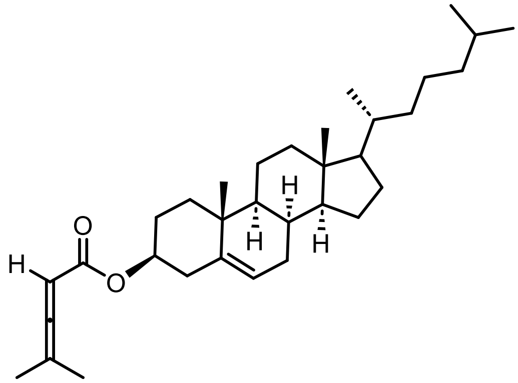

S49

Isolated yield: 40%, eluent: Et_2O/pentane (5: 95), white powder. ^{1}H NMR (400 MHz, Chloroform-*d*) δ 5.42 (hept, *J* = 2.8 Hz, 1H), 5.36 (dt, *J* = 5.5, 1.8 Hz, 1H), 4.70 – 4.57 (m, 1H), 2.41 – 2.25 (m, 2H), 2.05 – 1.79 (m, 5H), 1.79 (d, *J* = 2.9 Hz, 6H), 1.65 – 1.39 (m, 7H), 1.41 – 1.20 (m, 5H), 1.20 – 1.02 (m, 7H), 1.02 (s, 3H), 1.00 – 0.94 (m, 2H), 0.91 (d, *J* = 6.5 Hz, 3H), 0.86 (dd, *J* = 6.7, 1.8 Hz, 6H), 0.67 (s, 3H). ^{13}C NMR (101 MHz, Chloroform-*d*) δ 210.74, 166.11, 139.89, 122.65, 100.15, 86.56, 74.23, 56.82, 56.26, 50.15, 42.43, 39.87, 39.65, 38.28, 37.14, 36.72, 36.32, 35.93, 32.04, 31.99, 28.36, 28.13, 27.93, 24.41, 23.97, 22.95, 22.69, 21.17, 19.47, 19.45, 18.85, 11.98. HRMS-ESI: Found $[M+Na]^+$ =503.3871; $C_{33}H_{52}O_2Na$ requires 503.3867.

4-Methylpenta-2,3-dienoate((3a*S*,5*S*,5a*R*,8a*R*,8b*S*)-2,2,7,7-tetramethyltetrahydro-5H-bis([1,3]dioxolo)[4,5-b:4',5'-d]pyran-5-yl)methyl 4-methylpenta-2,3-dienoate

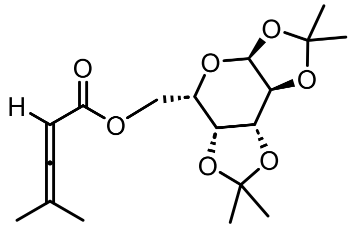

S50

Isolated yield: 62%, eluent: Et_2O/pentane (5: 95), colorless oil. ^{1}H NMR (400 MHz, Chloroform-*d*) δ 5.52 (d, *J* = 4.9 Hz, 1H), 5.48 (p, *J* = 2.8 Hz, 1H), 4.60 (dd, *J* = 7.9, 2.5 Hz, 1H), 4.34 – 4.27 (m, 2H), 4.26 – 4.19 (m, 2H), 4.07 – 3.99 (m, 1H), 1.77 (d, *J* = 2.9 Hz, 6H), 1.50 (s, 3H), 1.43 (s, 3H), 1.32 (d, *J* = 3.2 Hz, 6H). ^{13}C NMR (101 MHz, Chloroform-*d*) δ 211.18, 166.53, 109.71, 108.87, 100.39, 96.39, 85.93, 71.21, 70.80, 70.66, 66.23, 63.66, 26.13, 26.07, 25.13, 24.58, 19.37, 19.35. HRMS-ESI: Found $[M+Na]^+$ =377.1589; $C_{18}H_{26}O_7Na$ requires 377.1571.

Directing-group-free allenes **S43** and **S51** were prepared using the following procedures:

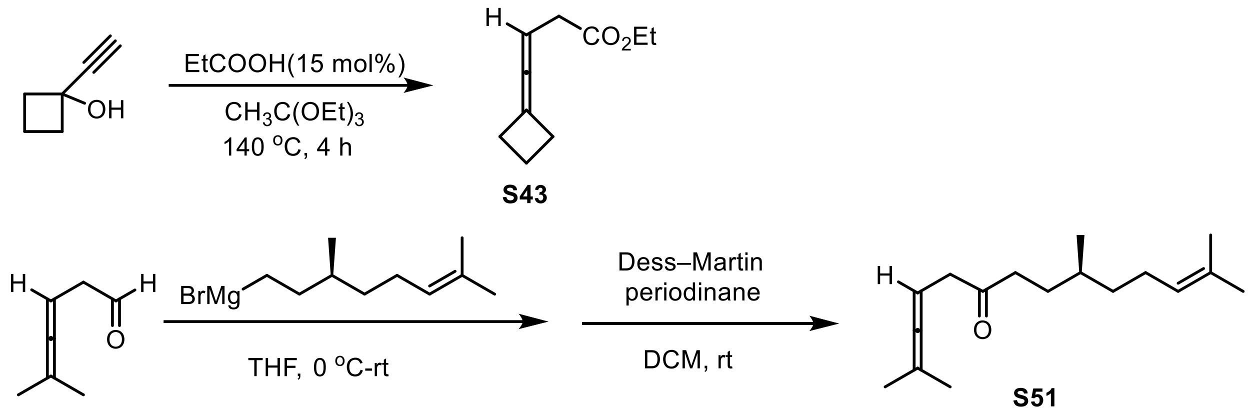

Scheme S4. Preparation of allenes **S43** and **S51**.

Synthesis of allene S43: (Johnson–Claisen rearrangement): A dry round-bottomed flask containing crude propargylic alcohol (10 mmol) was equipped with a distillation receiver and a condenser. Triethyl orthoacetate (20 ml) and propanoic acid (111 mg, 1.5 mmol, 0.15 equiv.) were added sequentially. The reaction was then refluxed at 140°C for 4 h before cooling down to 0 °C. Et_2O (100 mL) and HCl (aq., 1 M, 50 mL) were added and the layers were separated. The aqueous layer was extracted with Et_2O (50 mL x 2). The combined organic layers were dried over Na_2SO_4, filtered, evaporated. The residue was purified using column chromatography on silica gel to afford the desired allene **S43**.

Synthesis of allene S51: To a solution of starting aldehyde (1.1 g, 10 mmol, 1.0 equiv.) in 50 mL of dry THF was added the freshly prepared (*S*)-(+)-citronellyl magnesium bromide (0.7 M in THF) (17 mL, 12 mmol, 1.2 equiv.) under an atmosphere of nitrogen at 0 °C. The resulting mixture was stirred at room temperature for 1 h before quenching with saturated NH_4Cl (aq.) and extraction with Et_2O (20 mL x 3). The combined organic layers were dried over Na_2SO_4, filtered, evaporated to afford the alcohol, which was used for the next step without further purification.

The obtained alcohol was dissolved in 30 mL of DCM following by the addition of Dess-Martin periodinane (5.1 g, 12 mmol, 1.2 equiv.) at room temperature. The reaction mixture was stirred for 2 h before concentrating under reduced pressure. The residue was purified using column chromatography on silica gel to afford the desired allene **S51**.

Ethyl 4-cyclobutylidenebut-3-enoate

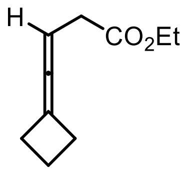

S43

Isolated yield: 58%, eluent: Et_2O/pentane (5: 95), colorless oil. ^1H NMR (400 MHz, Chloroform-*d*) δ 5.31 – 5.18 (m, 1H), 4.15 (q, *J* = 7.1 Hz, 2H), 3.01 (d, *J* = 7.1 Hz, 2H), 2.91 – 2.81 (m, 4H), 1.93 (p, *J* = 7.9 Hz, 2H), 1.26 (t, *J* = 7.1 Hz, 3H). ^{13}C NMR (101 MHz, Chloroform-*d*) δ 196.74, 171.86, 102.44, 86.48, 60.81, 35.78, 29.88, 17.64, 14.35. HRMS-ESI: Found $[M+Na]^+$ =189.0889; $C_{10}H_{14}O_2Na$ requires 189.0886.

(*S*)-2,9,13-Trimethyltetradeca-2,3,12-trien-6-one

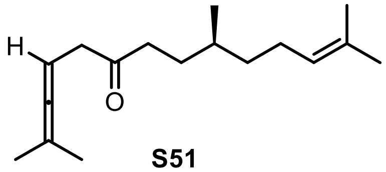

S51

Isolated yield: 64% over two steps, eluent: Et_2O/pentane (5: 95), colorless oil. ^1H NMR (400 MHz, Chloroform-*d*) δ 5.13 – 5.02 (m, 2H), 3.03 (d, *J* = 7.2 Hz, 2H), 2.55 – 2.36 (m, 2H), 2.07 – 1.86 (m, 2H), 1.69 (s, 3H), 1.68 (s, 3H), 1.68 (s, 3H), 1.59 (s, 3H), 1.46 – 1.27 (m, 4H), 1.20 – 1.10 (m, 1H), 0.87 (d, *J* = 6.2 Hz, 3H). ^{13}C NMR (101 MHz, Chloroform-*d*) δ 209.71, 203.44, 131.42, 124.83, 96.14, 82.22, 43.78, 39.88, 36.98, 32.27, 30.78, 25.86, 25.62, 20.54, 19.47, 17.80. HRMS-ESI: Found $[M+Na]^+$ =271.2019; $C_{17}H_{28}ONa$ requires 271.2032.

2. *Palladium-Catalyzed Allene-Allene Cross-Coupling*

2.1 *General Procedure for Oxidative Allene-Allene Cross-Coupling*

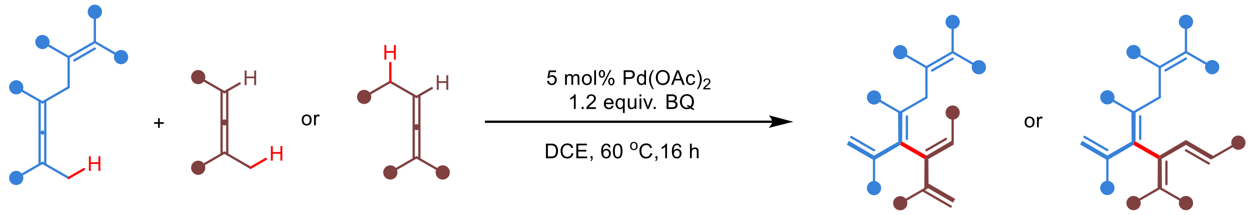

Scheme S5. Palladium-catalyzed allene-allene cross-coupling.

General procedure 4 (GP4): These reactions were conducted without special precautions to exclude moisture and oxygen. To a solution of enallene (0.2 mmol, 1.0 equiv.) and directing-group-free allene (0.3 mmol, 1.5 equiv.) in 2 mL of DCE was added $Pd(OAc)_2$ (2.2 mg, 0.01 mmol, 0.05 equiv.) and BQ (benzoquinone) (26 mg, 0.24 mmol, 1.2 equiv.) in one portion. The resulting mixture was stirred at 60 °C for 16 h before concentrating under reduced pressure. The residue was purified using column chromatography on silica gel to afford the [4]dendralene product.

2.2 *General Procedure for Aerobic Version of Allene-Allene Cross-Coupling*

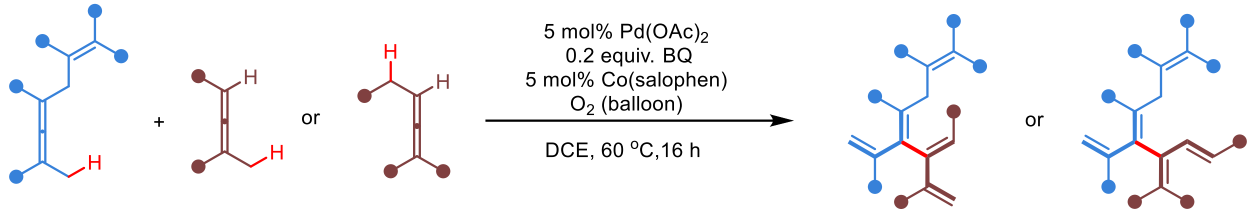

Scheme S6. Palladium-catalyzed allene-allene cross-coupling under aerobic conditions.

General procedure 5 (GP5): A Schlenk tube was charged with stirring bar, $Pd(OAc)_2$ (2.2 mg, 0.01 mmol, 0.05 equiv.), BQ (4.3 mg, 0.04 mmol, 0.2 equiv.) and Co(salophen)(Salcomine) (3.2 mg, 0.01 mmol, 0.05 equiv.) and then was sealed with a septum and subjected to three cycles of evacuation and filling with O_2 gas using a balloon. A solution of enallene (0.2 mmol, 1.0 equiv.) and directing-group-free allene (0.3 mmol, 1.5 equiv.) in 2 mL of DCE was added to the system. The resulting mixture was stirred at 60 °C for 16 h before concentrating under reduced pressure. The residue was purified using column chromatography on silica gel to afford the [4]dendralene product.

2.3 Characterization of Type-I [4]Dendralene Products

Diethyl (2*Z*,4*E*)-5-allyl-3,4-di(prop-1-en-2-yl)hepta-2,4-dienedioate

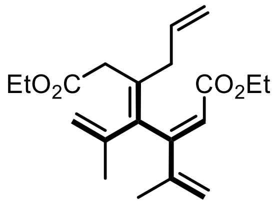

3

The reaction was carried out according to the general procedure ***GP4*** using eneallene **1** (39 mg, 0.2 mmol) and allene **2** (42 mg, 0.3 mmol), and the desired product was obtained after purification by silica gel column chromatography (eluent: Et_2O/pentane (5: 95)) as a colorless oil (62 mg, 93% yield). ^{1}H NMR (400 MHz, Chloroform-*d*) δ 5.98 (s, 1H), 5.70 (s, 1H), 5.70 – 5.58 (m, 1H), 5.35 (s, 1H), 5.05 – 4.92 (m, 4H), 4.18 – 4.06 (m, 4H), 3.60 (d, *J* = 16.3 Hz, 1H), 3.09 (d, *J* = 16.0 Hz, 1H), 2.71 (dd, *J* = 20.5, 6.4 Hz, 2H), 1.96 (s, 3H), 1.28 – 1.23 (m, 6H). ^{13}C NMR (101 MHz, Chloroform-*d*) δ 172.39, 166.12, 154.94, 143.49, 140.80, 138.02, 135.83, 128.23, 123.03, 117.42, 117.01, 116.75, 60.59, 60.06, 39.00, 37.06, 22.56, 20.48, 14.39, 14.35. HRMS-ESI: Found $[M+Na]^+$ =355.1887; $C_{20}H_{28}O_4Na$ requires 355.1880.

Ethyl (2*Z*,4*Z*)-5-allyl-3,4-di(prop-1-en-2-yl)nona-2,4-dienoate

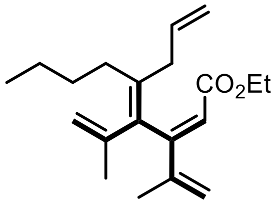

5

The reaction was carried out according to the general procedure ***GP4*** using eneallene **S1** (33 mg, 0.2 mmol) and allene **2** (42 mg, 0.3 mmol), and the desired product was obtained after purification by silica gel column chromatography (eluent: Et_2O/pentane (5: 95)) as a colorless oil (57 mg, 95% yield). ^{1}H NMR (400 MHz, Chloroform-*d*) δ 5.94 (s, 1H), 5.76 – 5.61 (m, 1H), 5.43 (d, *J* = 1.9 Hz, 1H), 5.32 – 5.26 (m, 1H), 5.03 – 4.89 (m, 3H), 4.87 (dd, *J* = 2.6, 0.9 Hz, 1H), 4.12 (q, *J* = 7.1 Hz, 2H), 2.67 (dt, *J* = 7.0, 1.4 Hz, 2H), 2.27 (s, 2H), 1.95 (s, 3H), 1.75 (s, 3H), 1.43 (s, 2H), 1.32 (q, *J* = 7.1 Hz, 2H), 1.26 (t, *J* = 7.1 Hz, 3H), 0.90 (t, *J* = 7.2 Hz, 3H). ^{13}C NMR (101 MHz, Chloroform-*d*) δ 166.29, 155.83, 144.00, 141.67, 136.88, 136.05, 133.71, 121.89, 117.34, 115.87, 115.76, 59.96, 37.79, 30.97, 30.71, 23.26, 23.18, 20.57, 14.46, 14.14. HRMS-ESI: Found $[M+Na]^+$ =325.2136; $C_{20}H_{30}O_2Na$ requires 325.2138.

Ethyl (2*Z*,4*E*)-5-cyclopentyl-3,4-di(prop-1-en-2-yl)octa-2,4,7-trienoate

The reaction was carried out according to the general procedure ***GP4*** using eneallene **S2** (35 mg, 0.2 mmol) and allene **2** (42 mg, 0.3 mmol), and the desired product was obtained after purification by silica gel column chromatography (eluent: Et_2O/pentane (5: 95)) as a colorless oil (46 mg, 74%

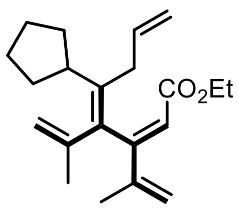

6

yield). ^{1}H NMR (400 MHz, Chloroform-*d*) δ 5.92 (s, 1H), 5.80 – 5.65 (m, 1H), 5.45 (d, *J* = 2.0 Hz, 1H), 5.29 (s, 1H), 4.99 – 4.93 (m, 1H), 4.93 – 4.86 (m, 2H), 4.80 (dq, *J* = 10.0, 1.7 Hz, 1H), 4.13 (q, *J* = 7.1 Hz, 2H), 3.24 – 3.13 (m, 1H), 2.69 (dd, *J* = 23.4, 6.7 Hz, 2H), 1.94 (s, 3H), 1.75 (s, 3H), 1.69 – 1.49 (m, 8H), 1.26 (t, *J* = 7.1 Hz, 3H). ^{13}C NMR (101 MHz, Chloroform-*d*) δ 166.33, 155.39, 144.42, 141.37, 138.95, 137.46, 135.32, 121.96, 117.75, 115.55, 114.46, 59.96, 43.72, 35.13, 31.97, 31.33, 25.81, 23.35, 20.68, 14.48. HRMS-ESI: Found $[M+Na]^+$ =337.2136; $C_{21}H_{30}O_2Na$ requires 337.2138.

Ethyl (2*Z*,4*E*)-5-benzyl-3,4-di(prop-1-en-2-yl)octa-2,4,7-trienoate

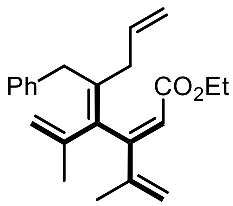

7

The reaction was carried out according to the general procedure ***GP4*** using eneallene **S3** (40 mg, 0.2 mmol) and allene **2** (42 mg, 0.3 mmol), and the desired product was obtained after purification by silica gel column chromatography (eluent: Et_2O/pentane (5: 95)) as a colorless oil (59 mg, 88% yield). ^{1}H NMR (400 MHz, Chloroform-*d*) δ 7.33 – 7.24 (m, 4H), 7.22 – 7.16 (m, 1H), 6.00 (s, 1H), 5.74 – 5.59 (m, 1H), 5.52 (d, *J* = 1.9 Hz, 1H), 5.34 (s, 1H), 5.08 – 5.00 (m, 2H), 4.97 – 4.89 (m, 1H), 4.90 – 4.79 (m, 1H), 4.15 (q, *J* = 7.1 Hz, 2H), 3.73 (d, *J* = 36.5 Hz, 2H), 2.57 (dt, *J* = 7.1, 1.4 Hz, 2H), 1.99 (s, 3H), 1.83 (t, *J* = 1.2 Hz, 3H), 1.26 (t, *J* = 7.1 Hz, 3H). ^{13}C NMR (101 MHz, Chloroform-*d*) δ 166.15, 155.30, 143.61, 141.68, 140.69, 136.34, 135.86, 133.91, 129.25, 128.36, 125.85, 122.09, 117.74, 116.41, 116.33, 60.05, 37.16, 37.13, 23.37, 20.65, 14.45. HRMS-ESI: Found $[M+Na]^+$ =359.1974; $C_{23}H_{28}O_2Na$ requires 359.1982.

Ethyl (2*Z*,4*E*)-3,4-di(prop-1-en-2-yl)-5-((trimethylsilyl)methyl)octa-2,4,7-trienoate

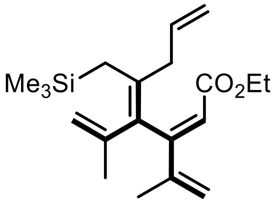

8

The reaction was carried out according to the general procedure ***GP4*** using eneallene **S4** (39 mg, 0.2 mmol) and allene **2** (42 mg, 0.3 mmol), and the desired product was obtained after purification by silica gel column chromatography (eluent: Et_2O/pentane (5: 95)) as a colorless oil (60 mg, 91% yield). ^{1}H NMR (400 MHz, Chloroform-*d*) δ 5.95 (s, 1H), 5.76 – 5.60 (m, 1H), 5.48 (d, *J* = 1.9 Hz, 1H), 5.29 (s, 1H), 5.03 – 4.86 (m, 4H), 4.11 (q, *J* = 7.2 Hz, 2H), 2.63 (s, 2H), 1.95 (s, 3H), 1.75 (s, 3H), 1.25 (t, *J* = 7.1 Hz, 3H), 0.08 (s, 9H). ^{13}C NMR (101 MHz, Chloroform-*d*) δ 166.15, 156.37, 144.44, 142.19, 137.14,

134.20, 131.06, 121.78, 117.57, 116.10, 116.05, 59.87, 40.52, 23.21, 22.09, 20.65, 14.46, 0.12. HRMS-ESI: Found $[M+Na]^+$ =355.2054; $C_{20}H_{32}O_2SiNa$ requires 355.2064.

Ethyl (2*Z*,4*E*)-5-(2-(benzyloxy)ethyl)-3,4-di(prop-1-en-2-yl)octa-2,4,7-trienoate

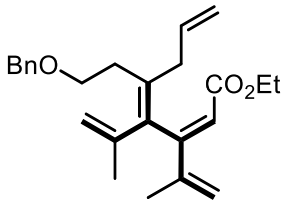

9

The reaction was carried out according to the general procedure ***GP4*** using eneallene **S5** (49 mg, 0.2 mmol) and allene **2** (42 mg, 0.3 mmol), and the desired product was obtained after purification by silica gel column chromatography (eluent: Et_2O/pentane (5: 95)) as a colorless oil (72 mg, 94% yield). ^1H NMR (400 MHz, Chloroform-*d*) δ 7.37 – 7.32 (m, 4H), 7.31 – 7.27 (m, 1H), 5.95 (s, 1H), 5.77 – 5.62 (m, 1H), 5.47 (s, 1H), 5.28 (s, 1H), 5.03 – 4.91 (m, 4H), 4.52 (s, 2H), 4.11 (q, *J* = 7.1 Hz, 2H), 3.62 (t, *J* = 7.3 Hz, 2H), 2.78 (s, 1H), 2.68 (d, *J* = 7.0 Hz, 2H), 2.59 (s, 1H), 1.95 (s, 3H), 1.74 (s, 3H), 1.25 (t, *J* = 7.1 Hz, 3H). ^{13}C NMR (101 MHz, Chloroform-*d*) δ 166.15, 155.62, 143.65, 141.23, 138.75, 136.54, 135.91, 132.12, 128.39, 127.69, 127.53, 122.32, 117.25, 116.31, 116.29, 72.85, 68.95, 59.95, 38.24, 31.42, 23.14, 20.48, 14.42. HRMS-ESI: Found $[M+Na]^+$ =403.2246; $C_{25}H_{32}O_3Na$ requires 403.2244.

Ethyl (2*Z*,4*E*)-5-(2-((tert-butyldimethylsilyl)oxy)ethyl)-3,4-di(prop-1-en-2-yl)octa-2,4,7-trienoate

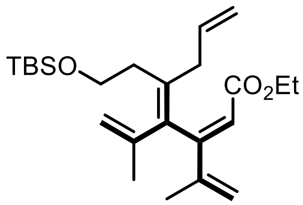

10

The reaction was carried out according to the general procedure ***GP4*** using eneallene **S6** (53 mg, 0.2 mmol) and allene **2** (42 mg, 0.3 mmol), and the desired product was obtained after purification by silica gel column chromatography (eluent: Et_2O/pentane (5: 95)) as a colorless oil (72 mg, 89% yield). ^1H NMR (400 MHz, Chloroform-*d*) δ 5.94 (s, 1H), 5.77 – 5.62 (m, 1H), 5.49 (d, *J* = 1.6 Hz, 1H), 5.29 (s, 1H), 5.04 – 4.92 (m, 4H), 4.12 (q, *J* = 7.1 Hz, 2H), 3.73 (t, *J* = 7.4 Hz, 2H), 2.68 (d, *J* = 7.0 Hz, 3H), 2.43 (s, 1H), 1.95 (s, 3H), 1.74 (s, 3H), 1.26 (t, *J* = 7.1 Hz, 3H), 0.89 (s, 9H), 0.05 (s, 6H). ^{13}C NMR (101 MHz, Chloroform-*d*) δ 166.19, 155.75, 143.71, 141.25, 136.73, 135.89, 132.39, 122.36, 117.24, 116.27, 116.17, 62.06, 59.97, 38.37, 34.65, 26.16, 23.21, 20.49, 18.52, 14.46, -5.09. HRMS-ESI: Found $[M+Na]^+$ =427.2639; $C_{24}H_{40}O_3SiNa$ requires 427.2639.

Ethyl (2*Z*,4*E*)-5-(2-(1,3-dioxoisoindolin-2-yl)ethyl)-3,4-di(prop-1-en-2-yl)octa-2,4,7-trienoate

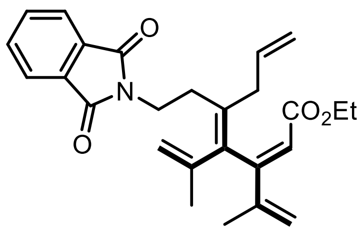

11

The reaction was carried out according to the general procedure ***GP4*** using eneallene **S7** (56 mg, 0.2 mmol) and allene **2** (42 mg, 0.3 mmol), and the desired product was obtained after purification by silica gel column chromatography (eluent: EtOAc/pentane (10: 90)) as a colorless oil (71 mg, 84% yield). ^{1}H NMR (400 MHz, Chloroform-*d*) δ 7.82 (dd, *J* = 5.4, 3.1 Hz, 2H), 7.68 (dd, *J* = 5.5, 3.0 Hz, 2H), 5.92 (s, 1H), 5.81 – 5.65 (m, 1H), 5.36 (d, *J* = 1.8 Hz, 1H), 5.26 (s, 1H), 5.14 (dd, *J* = 17.1, 1.7 Hz, 1H), 5.03 (dd, *J* = 11.7, 2.7 Hz, 2H), 4.87 (s, 1H), 4.03 (q, *J* = 7.1 Hz, 2H), 3.83 (s, 2H), 2.80 (dt, *J* = 7.1, 1.4 Hz, 2H), 2.73 (s, 2H), 1.92 (s, 3H), 1.72 (s, 3H), 1.21 (t, *J* = 7.1 Hz, 3H). ^{13}C NMR (101 MHz, Chloroform-*d*) δ 168.37, 165.87, 155.29, 143.45, 141.25, 136.85, 136.10, 133.83, 132.49, 131.70, 123.12, 121.99, 117.25, 116.96, 116.50, 59.92, 37.60, 36.62, 29.97, 22.99, 20.51, 14.43. HRMS-ESI: Found $[M+Na]^+$ =442.1990; $C_{26}H_{29}NO_4Na$ requires 442.1989.

Ethyl (2*Z*,4*Z*)-5-(2-cyanoethyl)-3,4-di(prop-1-en-2-yl)octa-2,4,7-trienoate

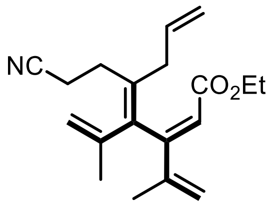

12

The reaction was carried out according to the general procedure ***GP4*** using eneallene **S8** (32 mg, 0.2 mmol) and allene **2** (42 mg, 0.3 mmol), and the desired product was obtained after purification by silica gel column chromatography (eluent: Et_2O/pentane (5: 95)) as a colorless oil (45 mg, 74% yield). ^{1}H NMR (400 MHz, Chloroform-*d*) δ 5.95 (s, 1H), 5.66 (ddt, *J* = 17.0, 10.0, 7.0 Hz, 1H), 5.49 (s, 1H), 5.34 (s, 1H), 5.09 – 5.00 (m, 3H), 4.95 (s, 1H), 4.12 (q, *J* = 7.1 Hz, 2H), 2.69 (t, *J* = 7.7 Hz, 4H), 2.49 (t, *J* = 8.0 Hz, 2H), 1.95 (s, 3H), 1.75 (s, 3H), 1.26 (t, *J* = 7.1 Hz, 3H). ^{13}C NMR (101 MHz, Chloroform-*d*) δ 165.91, 155.13, 143.33, 140.90, 137.46, 135.81, 131.50, 122.55, 120.10, 117.35, 117.21, 116.89, 60.08, 37.45, 27.00, 23.03, 20.49, 15.94, 14.45. HRMS-ESI: Found $[M+Na]^+$ =322.1778; $C_{19}H_{25}NO_2Na$ requires 322.1778.

Ethyl (2*Z*,4*Z*)-5-allyl-3,4-di(prop-1-en-2-yl)deca-2,4,9-trienoate

The reaction was carried out according to the general procedure ***GP4*** using eneallene **S9** (35 mg, 0.2 mmol) and allene **2** (42 mg, 0.3 mmol), and the desired product was obtained after purification by silica gel column chromatography (eluent: Et_2O/pentane (5: 95)) as a colorless oil (51 mg, 82% yield). ^{1}H NMR (400 MHz, Chloroform-*d*) δ 5.94 (s, 1H), 5.82 (ddt, *J* = 16.9, 10.2, 6.7 Hz, 1H), 5.68 (ddt, *J* = 17.0, 10.0, 7.0 Hz, 1H), 5.43 (s, 1H), 5.29 (s, 1H), 5.06 – 4.93 (m, 4H), 4.93 (s, 1H), 4.87 (s, 1H), 4.12 (q, *J* = 7.1 Hz, 2H), 2.68 (dt, *J* = 7.0, 1.5 Hz, 2H), 2.29 (s, 2H), 2.06 (q, *J* = 7.3

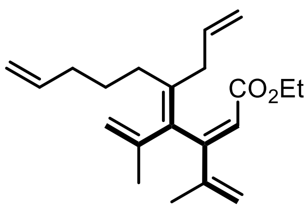

13

Hz, 2H), 1.95 (s, 3H), 1.75 (s, 3H), 1.55 (s, 2H), 1.26 (t, *J* = 7.1 Hz, 3H). ^{13}C NMR (101 MHz, Chloroform-*d*) δ 166.24, 155.74, 143.94, 141.63, 138.98, 136.78, 135.70, 134.06, 121.89, 117.35, 115.99, 115.86, 114.57, 59.97, 37.82, 34.23, 30.85, 27.84, 23.24, 20.56, 14.47. HRMS-ESI: Found $[M+Na]^+$ =337.2139; $C_{21}H_{30}O_2Na$ requires 337.2138.

Ethyl (2*Z*,4*E*)-5-phenyl-3,4-di(prop-1-en-2-yl)octa-2,4,7-trienoate

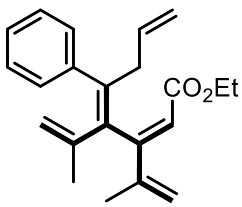

14

The reaction was carried out according to the general procedure ***GP4*** using eneallene **S10** (37 mg, 0.2 mmol) and allene **2** (42 mg, 0.3 mmol), and the desired product was obtained after purification by silica gel column chromatography (eluent: Et_2O/pentane (5: 95)) as a colorless oil (46 mg, 72% yield). ^{1}H NMR (400 MHz, Chloroform-*d*) δ 7.36 – 7.27 (m, 4H), 7.25 – 7.20 (m, 1H), 6.05 (s, 1H), 5.60 – 5.44 (m, 2H), 5.38 (s, 1H), 4.86 – 4.74 (m, 4H), 4.17 (q, *J* = 7.0 Hz, 2H), 3.01 (d, *J* = 6.4 Hz, 2H), 2.03 (s, 3H), 1.49 (dd, *J* = 1.3, 0.6 Hz, 3H), 1.29 (t, *J* = 7.1 Hz, 3H). ^{13}C NMR (101 MHz, Chloroform-*d*) δ 166.22, 156.60, 143.62, 142.43, 141.68, 136.35 (d, *J* = 1.6 Hz), 135.84, 129.06, 127.95, 126.79, 122.11, 117.97, 117.30, 115.73, 60.09, 41.31, 23.20, 20.54, 14.53. HRMS-ESI: Found $[M+Na]^+$ =345.1828; $C_{22}H_{26}O_2Na$ requires 345.1825.

Ethyl (2*Z*,4*E*)-5-(4-methoxyphenyl)-3,4-di(prop-1-en-2-yl)octa-2,4,7-trienoate

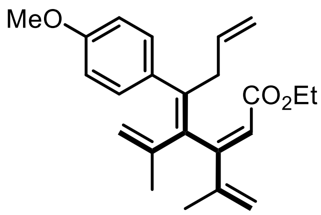

15

The reaction was carried out according to the general procedure ***GP4*** using eneallene **S11** (43 mg, 0.2 mmol) and allene **2** (42 mg, 0.3 mmol), and the desired product was obtained after purification by silica gel column chromatography (eluent: Et_2O/pentane (5: 95)) as a colorless oil (47 mg, 67% yield). ^{1}H NMR (400 MHz, Chloroform-*d*) δ 7.30 – 7.24 (m, 2H), 6.88 – 6.79 (m, 2H), 6.03 (s, 1H), 5.59 – 5.44 (m, 2H), 5.36 (s, 1H), 4.87 – 4.74 (m, 4H), 4.16 (q, *J* = 7.1 Hz, 2H), 3.81 (s, 3H), 2.98 (dd, *J* = 6.7, 1.5 Hz, 2H), 2.02 (s, 3H), 1.49 (s, 3H), 1.28 (t, *J* = 7.1 Hz, 3H). ^{13}C NMR (101 MHz, Chloroform-*d*) δ 166.25, 158.58, 156.94, 143.80, 141.75, 136.09, 135.99, 135.87, 134.66, 130.14, 122.02, 117.69, 117.15, 115.60, 113.36, 60.05, 55.26, 41.26, 23.24, 20.56, 14.53. HRMS-ESI: Found $[M+Na]^+$ =375.1930; $C_{23}H_{28}O_3Na$ requires 375.1931.

Ethyl (2*Z*,4*E*)-5-(4-chlorophenyl)-3,4-di(prop-1-en-2-yl)octa-2,4,7-trienoate

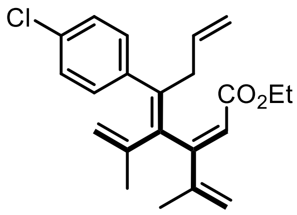

16

The reaction was carried out according to the general procedure ***GP4*** using eneallene **S12** (44 mg, 0.2 mmol) and allene **2** (42 mg, 0.3 mmol), and the desired product was obtained after purification by silica gel column chromatography (eluent: Et_2O/pentane (5: 95)) as a colorless oil (51 mg, 71% yield). ^{1}H NMR (400 MHz, Chloroform-*d*) δ 7.30 – 7.24 (m, 4H), 6.04 (s, 1H), 5.56 – 5.40 (m, 2H), 5.37 (s, 1H), 4.87 – 4.77 (m, 2H), 4.77 (dt, *J* = 3.6, 1.3 Hz, 2H), 4.16 (qd, *J* = 7.1, 1.2 Hz, 2H), 2.97 (dt, *J* = 6.9, 1.5 Hz, 2H), 2.02 (s, 3H), 1.51 (s, 3H), 1.29 (t, *J* = 7.1 Hz, 3H). ^{13}C NMR (101 MHz, Chloroform-*d*) δ 166.13, 156.36, 143.20, 141.51, 140.87, 137.16, 135.50, 135.14, 132.60, 130.47, 128.21, 122.17, 118.52, 117.37, 116.11, 60.12, 41.07, 23.18, 20.51, 14.52. HRMS-ESI: Found $[M+Na]^+$ =379.1435; $C_{22}H_{25}ClO_2Na$ requires 379.1436.

Ethyl (2*Z*,4*E*)-3,4-di(prop-1-en-2-yl)-5-(thiophen-3-yl)octa-2,4,7-trienoate

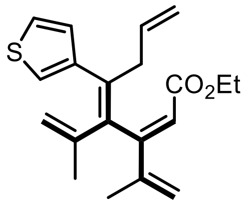

17

The reaction was carried out according to the general procedure ***GP4*** using eneallene **S13** (38 mg, 0.2 mmol) and allene **2** (42 mg, 0.3 mmol), and the desired product was obtained after purification by silica gel column chromatography (eluent: Et_2O/pentane (5: 95)) as a colorless oil (48 mg, 73% yield). ^{1}H NMR (400 MHz, Chloroform-*d*) δ 7.25 (d, *J* = 5.8 Hz, 1H), 7.07 (d, *J* = 3.5 Hz, 1H), 6.99 – 6.95 (m, 1H), 6.01 (s, 1H), 5.70 – 5.55 (m, 1H), 5.48 (s, 1H), 5.37 (s, 1H), 5.00 – 4.84 (m, 4H), 4.16 (q, *J* = 7.1 Hz, 2H), 3.00 (d, *J* = 6.7 Hz, 2H), 2.02 (s, 3H), 1.66 (s, 3H), 1.28 (t, *J* = 7.1 Hz, 3H). ^{13}C NMR (101 MHz, Chloroform-*d*) δ 166.21, 156.13, 143.94, 143.62, 141.52, 138.19, 135.77, 129.38, 127.31, 126.74, 125.23, 122.32, 118.24, 117.30, 116.24, 60.20, 41.74, 22.72, 20.63, 14.51. HRMS-ESI: Found $[M+Na]^+$ =351.1390; $C_{20}H_{24}O_2SNa$ requires 351.1389.

Diethyl (2*Z*,4*E*)-5-allyl-4-(1-phenylvinyl)-3-(prop-1-en-2-yl)hepta-2,4-dienedioate

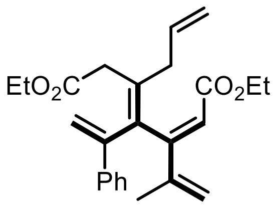

18

The reaction was carried out according to the general procedure ***GP4*** using eneallene **S14** (51 mg, 0.2 mmol) and allene **2** (42 mg, 0.3 mmol), and the desired product was obtained after purification by silica gel column chromatography (eluent: Et_2O/pentane (5: 95)) as a pale yellow oil (51 mg, 64% yield). ^{1}H NMR (400 MHz, Chloroform-*d*) δ 7.34 – 7.29 (m, 2H), 7.25 – 7.20 (m, 3H), 5.89 (s, 1H), 5.77 (ddt, *J* = 17.1, 10.0, 7.2 Hz, 1H), 5.67 (s, 1H), 5.34 – 5.24 (m, 3H), 5.09 – 4.97 (m, 2H), 4.07 (dq, *J* = 14.2, 7.1 Hz, 4H), 3.28 (s, 2H), 2.87 (d, *J* = 6.0 Hz, 2H), 1.89 (s, 3H), 1.28 – 1.17 (m, 6H). ^{13}C NMR (101 MHz, Chloroform-*d*) δ 171.90, 165.89, 155.31, 148.08, 141.42, 135.82 (d, *J* = 9.0 Hz), 131.39, 128.17, 127.83, 127.30, 122.09, 119.78, 117.68, 117.27, 60.58, 60.01, 39.81, 37.53, 20.70, 14.36, 14.30. HRMS-ESI: Found $[M+Na]^+$ =417.2041; $C_{25}H_{30}O_4Na$ requires 417.2036.

Diethyl (2*Z*,4*E*)-5-allyl-4-(cyclopent-1-en-1-yl)-3-(prop-1-en-2-yl)hepta-2,4-dienedioate

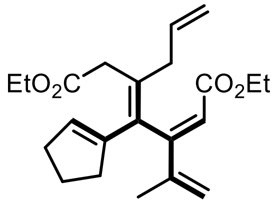

19

The reaction was carried out according to the general procedure ***GP4*** using eneallene **S15** (44 mg, 0.2 mmol) and allene **2** (42 mg, 0.3 mmol), and the desired product was obtained after purification by silica gel column chromatography (eluent: Et_2O/pentane (5: 95)) as a colorless oil (51 mg, 71% yield). ^{1}H NMR (400 MHz, Chloroform-*d*) δ 5.93 (s, 1H), 5.70 (s, 1H), 5.68 – 5.62 (m, 1H), 5.66 – 5.52 (m, 1H), 5.32 (s, 1H), 5.03 – 4.91 (m, 2H), 4.19 – 4.05 (m, 4H), 3.57 (d, *J* = 16.2 Hz, 1H), 3.07 (d, *J* = 16.2 Hz, 1H), 2.69 (qd, *J* = 14.3, 6.8 Hz, 2H), 2.39 – 2.21 (m, 4H), 1.95 (s, 3H), 1.78 (p, *J* = 7.5 Hz, 2H), 1.30 – 1.21 (m, 6H). ^{13}C NMR (101 MHz, Chloroform-*d*) δ 172.43, 166.20, 155.61, 141.82, 140.84, 135.87, 134.47, 130.57, 127.59, 123.03, 116.85, 116.69, 60.59, 59.98, 39.32, 37.22, 35.36, 33.01, 23.50, 20.30, 14.40, 14.38. HRMS-ESI: Found $[M+Na]^+$ =381.2036; $C_{22}H_{30}O_4Na$ requires 381.2036.

Diethyl (2*Z*,4*E*)-5-allyl-4-(cyclohept-1-en-1-yl)-3-(prop-1-en-2-yl)hepta-2,4-dienedioate

The reaction was carried out according to the general procedure ***GP4*** using eneallene **S16** (50 mg, 0.2 mmol) and allene **2** (42 mg, 0.3 mmol), and the desired product was obtained after purification by silica gel column chromatography (eluent: Et_2O/pentane (5: 95)) as a colorless oil (57 mg, 74% yield). ^{1}H NMR (400 MHz, Chloroform-*d*) δ 5.95 (s, 1H), 5.85 (t, *J* = 6.7 Hz, 1H), 5.72 – 5.56 (m, 2H), 5.31 (s, 1H), 5.02 – 4.92 (m, 2H), 4.22 – 4.06 (m, 4H), 3.54 (d, *J* = 16.2 Hz, 1H), 3.07 (d, *J* = 16.2 Hz, 1H), 2.79 – 2.63 (m, 2H), 2.19 – 2.08 (m, 4H), 1.94 (s, 3H), 1.74 – 1.64 (m, 2H), 1.39

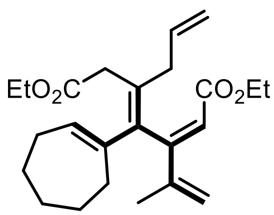

20

(ddt, *J* = 27.7, 11.9, 6.2 Hz, 4H), 1.25 (td, *J* = 7.1, 3.4 Hz, 6H). ^{13}C NMR (101 MHz, Chloroform-*d*) δ 172.53, 166.37, 155.70, 142.93, 141.32, 139.69, 136.12, 133.49, 126.92, 122.53, 117.13, 116.77, 60.49, 60.05, 39.06, 37.30, 32.81, 31.60, 28.75, 26.85, 26.80, 20.61, 14.41, 14.39. HRMS-ESI: Found $[M+Na]^+$ =409.2351; $C_{24}H_{34}O_4Na$ requires 409.2349.

Diethyl (2*Z*,4*E*)-5-(3-methylbut-2-en-1-yl)-3,4-di(prop-1-en-2-yl)hepta-2,4-dienedioate

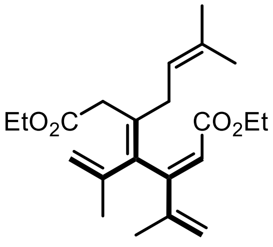

21

The reaction was carried out according to the general procedure ***GP4*** using eneallene **S17** (45 mg, 0.2 mmol) and allene **2** (42 mg, 0.3 mmol), and the desired product was obtained after purification by silica gel column chromatography (eluent: Et_2O/pentane (5: 95)) as a colorless oil (61 mg, 84% yield). ^{1}H NMR (400 MHz, Chloroform-*d*) δ 5.97 (s, 1H), 5.71 (s, 1H), 5.36 (s, 1H), 5.03 – 4.92 (m, 3H), 4.17 – 4.09 (m, 4H), 3.58 (d, *J* = 15.8 Hz, 1H), 3.03 (d, *J* = 15.9 Hz, 1H), 2.66 (t, *J* = 6.1 Hz, 3H), 1.96 (s, 3H), 1.71 (s, 3H), 1.65 (s, 3H), 1.55 (s, 3H), 1.26 (td, *J* = 7.1, 1.2 Hz, 6H). ^{13}C NMR (101 MHz, Chloroform-*d*) δ 172.60, 166.22, 155.23, 143.62, 140.87, 137.34, 133.28, 129.64, 123.01, 121.68, 117.33, 116.58, 60.56, 60.04, 37.32, 33.10, 25.95, 22.60, 20.48, 17.74, 14.40. HRMS-ESI: Found $[M+Na]^+$ =383.2190; $C_{22}H_{32}O_4Na$ requires 383.2193.

Diethyl (2*Z*,4*E*)-5-(2-methylallyl)-3,4-di(prop-1-en-2-yl)hepta-2,4-dienedioate

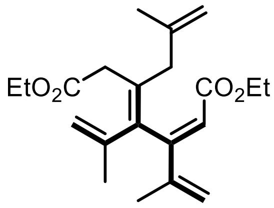

22

The reaction was carried out according to the general procedure ***GP4*** using eneallene **S18** (42 mg, 0.2 mmol) and allene **2** (42 mg, 0.3 mmol), and the desired product was obtained after purification by silica gel column chromatography (eluent: Et_2O/pentane (5: 95)) as a colorless oil (60 mg, 87% yield). ^{1}H NMR (400 MHz, Chloroform-*d*) δ 5.98 (s, 1H), 5.74 (s, 1H), 5.38 (s, 1H), 5.06 – 5.00 (m, 2H), 4.76 (s, 1H), 4.68 (s, 1H), 4.17 – 4.05 (m, 4H), 3.62 (d, *J* = 12.8 Hz, 1H), 3.10 (d, *J* = 13.8 Hz, 1H), 2.70 (s, 2H), 1.97 (s, 3H), 1.74 (s, 3H), 1.59 (s, 3H), 1.25 (t, *J* = 7.1 Hz, 6H). ^{13}C NMR (101 MHz, Chloroform-*d*) δ 172.36, 165.86, 155.00, 143.60, 140.72, 139.21, 128.41, 123.51, 117.58, 116.95, 113.36, 60.54, 60.04, 42.56, 36.87, 22.85, 22.75, 20.57, 14.43, 14.36. HRMS-ESI: Found $[M+Na]^+$ =369.2034; $C_{21}H_{30}O_4Na$ requires 369.2036.

Diethyl (2*Z*,4*E*)-5-(2-phenylallyl)-3,4-di(prop-1-en-2-yl)hepta-2,4-dienedioate

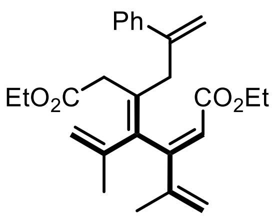

23

The reaction was carried out according to the general procedure ***GP4*** using eneallene **S19** (54 mg, 0.2 mmol) and allene **2** (42 mg, 0.3 mmol), and the desired product was obtained after purification by silica gel column chromatography (eluent: Et_2O/pentane (5: 95)) as a colorless oil (60 mg, 73% yield). ^{1}H NMR (400 MHz, Chloroform-*d*) δ 7.38 – 7.33 (m, 2H), 7.30 – 7.20 (m, 3H), 6.00 (s, 1H), 5.77 (s, 1H), 5.40 (s, 1H), 5.36 (s, 1H), 5.11 – 4.98 (m, 3H), 4.08 (dq, *J* = 11.2, 7.1 Hz, 4H), 3.65 (s, 1H), 3.21 (s, 2H), 3.15 (s, 1H), 1.99 (s, 3H), 1.76 (s, 3H), 1.22 (dt, *J* = 10.2, 7.1 Hz, 6H). ^{13}C NMR (101 MHz, Chloroform-*d*) δ 172.32, 165.81, 154.87, 145.35, 143.46, 141.76, 140.45, 139.56, 128.29, 128.22, 127.44, 126.26, 123.63, 117.69, 117.10, 115.40, 60.55, 60.04, 39.25, 36.99, 22.79, 20.63, 14.37, 14.28. HRMS-ESI: Found $[M+Na]^+$ =431.2194; $C_{26}H_{32}O_4Na$ requires 431.2193.

Diethyl (2*Z*,4*E*)-5-cinnamyl-3,4-di(prop-1-en-2-yl)hepta-2,4-dienedioate

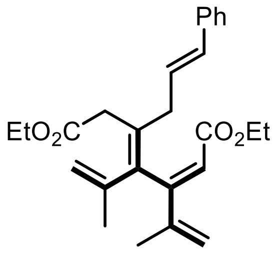

24

The reaction was carried out according to the general procedure ***GP4*** using eneallene **S20** (54 mg, 0.2 mmol) and allene **2** (42 mg, 0.3 mmol), and the desired product was obtained after purification by silica gel column chromatography (eluent: Et_2O/pentane (5: 95)) as a colorless oil (51 mg, 62% yield). ^{1}H NMR (400 MHz, Chloroform-*d*) δ 7.37 – 7.27 (m, 4H), 7.24 – 7.18 (m, 1H), 6.40 (d, *J* = 15.8 Hz, 1H), 6.15 – 6.03 (m, 1H), 5.74 (s, 1H), 5.05 (dt, *J* = 6.1, 1.8 Hz, 2H), 4.90 (s, 1H), 4.83 (s, 1H), 4.17 (q, *J* = 7.1 Hz, 2H), 4.09 (q, *J* = 7.1 Hz, 2H), 3.23 (s, 2H), 3.17 (dd, *J* = 7.0, 1.2 Hz, 2H), 1.93 (s, 3H), 1.77 (s, 3H), 1.28 (t, *J* = 7.1 Hz, 3H), 1.21 (t, *J* = 7.1 Hz, 3H). ^{13}C NMR (101 MHz, Chloroform-*d*) δ 171.83, 166.21, 156.54, 143.87, 142.68, 142.49, 137.45, 132.32, 128.77, 128.63, 127.72, 127.36, 126.23, 119.41, 116.70, 115.61, 60.72, 60.34, 37.53, 37.10, 22.65, 21.94, 14.30, 14.21. HRMS-ESI: Found $[M+Na]^+$ =431.2199; $C_{26}H_{32}O_4Na$ requires 431.2193.

Diethyl (2*Z*,4*Z*)-5-(cyclohex-2-en-1-yl)-3,4-di(prop-1-en-2-yl)hepta-2,4-dienedioate

The reaction was carried out according to the general procedure ***GP4*** using eneallene **S21** (47 mg, 0.2 mmol) and allene **2** (42 mg, 0.3 mmol) at 70 °C, and the desired product was obtained after purification by silica gel column chromatography (eluent: Et_2O/pentane (5: 95)) as a colorless oil

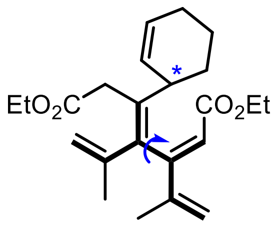

25

(30 mg, 40% yield). **[Note:** *atropisomerism was observed in [4]dendralene* ***25****, which results in two diastereomers present in NMR spectra***]** ^{1}H NMR (400 MHz, Chloroform-*d*) δ 6.00 (s, 1H), *[distinguishing peak for another diastereomer* 5.91 (s, 1H)*]* 5.94 (s, 1H), 5.77 – 5.68 (m, 1H), 5.42 (d, *J* = 10.4 Hz, 2H), 5.05 (d, *J* = 6.7 Hz, 1H), 4.96 (s, 1H), 4.12 (ddt, *J* = 12.5, 8.2, 4.7 Hz, 4H), 3.53 (dd, *J* = 22.7, 16.8 Hz, 1H), 2.95 (t, *J* = 17.9 Hz, 2H), 1.97 (s, 3H), 1.95 – 1.90 (m, 2H), 1.74 – 1.68 (m, 4H), 1.29 – 1.22 (m, 6H). One diastereomer: ^{13}C NMR (101 MHz, Chloroform-*d*) δ 173.28, 166.15, 155.34, 143.92, 141.47, 137.71, 132.70, 130.25, 129.27, 123.83, 117.17, 116.77, 60.58, 60.03, 42.12, 34.46, 27.40, 25.03, 22.65, 22.37, 20.58, 14.45, 14.33. Another diastereomer: ^{13}C NMR (101 MHz, Chloroform-*d*) δ 172.96, 166.15, 155.34, 143.77, 141.47, 137.71, 132.53, 129.49, 129.12, 123.73, 116.77, 116.56, 60.55, 60.03, 41.73, 34.42, 27.14, 24.92, 22.54, 22.26, 20.58, 14.45, 14.33. HRMS-ESI: Found $[M+Na]^+$ =395.2193; $C_{23}H_{32}O_4Na$ requires 395.2193.

Ethyl (3*E*,5*Z*)-3-allyl-5-((diethoxyphosphoryl)methylene)-6-methyl-4-(prop-1-en-2-yl)hepta-3,6-dienoate

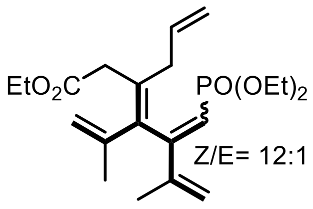

26

The reaction was carried out according to the general procedure ***GP4*** using eneallene **1** (39 mg, 0.2 mmol) and allene **S24** (61 mg, 0.3 mmol), and the desired product was obtained after purification by silica gel column chromatography (eluent: Et_2O/pentane (5: 95)) as a colorless oil (49 mg, 61% yield). ^{1}H NMR (400 MHz, Chloroform-*d*) δ 5.71 (ddt, *J* = 16.8, 10.3, 6.5 Hz, 1H), 5.49 (d, *J* = 19.3 Hz, 1H), 5.15 (s, 1H), 5.10 – 4.98 (m, 4H), 4.81 – 4.75 (m, 1H), 4.15 – 3.97 (m, 6H), 3.18 (s, 2H), 2.98 (dt, *J* = 6.6, 1.5 Hz, 2H), 1.85 (s, 3H), 1.71 (s, 3H), 1.29 (t, *J* = 7.1 Hz, 6H), 1.23 (t, *J* = 7.1 Hz, 3H). ^{31}P NMR (162 MHz, Chloroform-*d*) δ 16.06. ^{13}C NMR (101 MHz, Chloroform-*d*) δ 171.83, 161.76 (d, *J* = 5.4 Hz), 144.22 (d, *J* = 21.2 Hz), 142.47 (d, *J* = 5.7 Hz), 135.73, 128.78 (d, *J* = 1.1 Hz), 117.85 (d, *J* = 2.3 Hz), 117.50, 117.10, 116.44, 115.65, 61.76, 61.70, 60.68, 37.80, 37.37, 22.46, 21.20, 16.49, 16.42, 14.29. HRMS-ESI: Found $[M+Na]^+$ =419.1959; $C_{21}H_{33}O_5PNa$ requires 419.1958.

Ethyl (3*E*,5*Z*)-3-allyl-7-(methoxy(methyl)amino)-7-oxo-4,5-di(prop-1-en-2-yl)hepta-3,5-dienoate

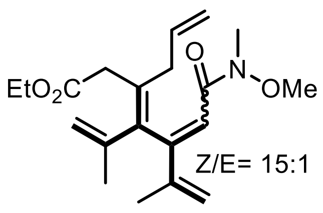

27

The reaction was carried out according to the general procedure ***GP4*** using eneallene **1** (39 mg, 0.2 mmol) and allene **S25** (46 mg, 0.3 mmol), and the desired product was obtained after purification by silica gel column chromatography (eluent: Et_2O/pentane (5: 95)) as a colorless oil (39 mg, 56% yield). ^{1}H NMR (400 MHz, Chloroform-*d*) δ 6.42 (s, 1H), 5.78 – 5.66 (m, 2H), 5.31 (s, 1H), 5.05 – 4.93 (m, 4H), 4.12 (q, *J* = 7.1 Hz, 2H), 3.67 (s, 3H), 3.16 (s, 3H), 2.75 (s, 2H), 1.98 (s, 3H), 1.73 (s, 3H), 1.25 (t, *J* = 7.1 Hz, 3H). ^{13}C NMR (101 MHz, Chloroform-*d*) δ 172.43, 152.02, 151.99, 143.93, 141.12, 138.06, 136.12, 128.10, 121.56, 116.81, 116.52, 116.07, 61.46, 60.38, 39.05, 36.87, 32.52, 22.49, 20.47, 14.22. HRMS-ESI: Found $[M+Na]^+$ =370.1991; $C_{20}H_{29}NO_4Na$ requires 370.1989.

Ethyl (3*E*,5*Z*)-3-allyl-5-(cyanomethylene)-6-methyl-4-(prop-1-en-2-yl)hepta-3,6-dienoate

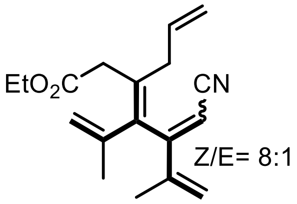

28

The reaction was carried out according to the general procedure ***GP4*** using eneallene **1** (39 mg, 0.2 mmol) and allene **S26** (28 mg, 0.3 mmol), and the desired product was obtained after purification by silica gel column chromatography (eluent: Et_2O/pentane (5: 95)) as a colorless oil (44 mg, 77% yield). ^{1}H NMR (400 MHz, Chloroform-*d*) δ 5.81 – 5.69 (m, 2H), 5.46 (s, 1H), 5.42 (s, 1H), 5.12 – 5.02 (m, 4H), 4.14 (q, *J* = 7.1 Hz, 2H), 3.35 (s, 2H), 2.75 (d, *J* = 7.0 Hz, 2H), 1.94 (s, 3H), 1.79 (s, 3H), 1.26 (t, *J* = 7.1 Hz, 3H). ^{13}C NMR (101 MHz, Chloroform-*d*) δ 171.78, 162.00, 142.48, 139.31, 138.09, 135.02, 131.69, 124.73, 117.96, 117.62, 96.55, 60.83, 38.70, 36.96, 22.71, 19.66, 14.32. HRMS-ESI: Found $[M+Na]^+$ =308.1627; $C_{18}H_{23}NO_2Na$ requires 308.1621.

Ethyl (3E,5Z)-3-allyl-7-(benzyloxy)-4,5-di(prop-1-en-2-yl)hepta-3,5-dienoate

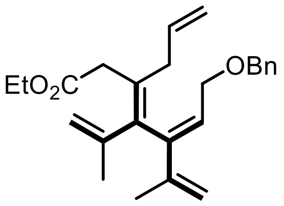

29

The reaction was carried out according to the general procedure ***GP4*** using eneallene **1** (39 mg, 0.2 mmol) and allene **S27** (56 mg, 0.3 mmol), 32 mg DMBQ was used instead of BQ and the desired product was obtained after purification by silica gel column chromatography (eluent: Et_2O/pentane (5: 95)) as a colorless oil (48 mg, 63% yield). ^{1}H NMR (400 MHz, Chloroform-*d*) δ 7.37 – 7.33 (m, 4H), 7.31 – 7.27 (m, 1H), 5.89 (t, *J* = 6.4 Hz, 1H), 5.63 (ddt, *J* = 17.2, 10.1, 7.1 Hz, 1H), 5.20 (s, 1H), 5.05 (s, 1H), 5.03 – 4.96 (m, 3H), 4.81 (s, 1H), 4.51 (s, 2H), 4.22 – 4.08 (m, 4H), 3.33 (d, *J* = 14.2 Hz, 2H), 2.75 (d, *J* = 7.1 Hz, 2H), 1.94 (s, 3H), 1.70

(s, 3H), 1.26 (t, J = 7.1 Hz, 3H). ^{13}C NMR (101 MHz, Chloroform-d) δ 172.15, 144.10, 141.97, 141.25, 139.22, 138.49, 135.89, 129.16, 128.50, 128.00, 127.75, 125.97, 117.07, 116.68, 115.68, 73.08, 68.96, 60.61, 38.42, 36.92, 22.54, 20.52, 14.39. HRMS-ESI: Found $[M+Na]^+$ =403.2242; $C_{25}H_{32}O_3Na$ requires 403.2244.

Ethyl (*E*)-3-allyl-5-((*Z*)-benzylidene)-6-methyl-4-(prop-1-en-2-yl)hepta-3,6-dienoate

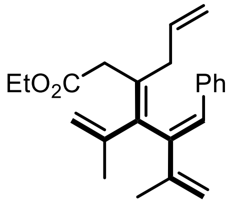

30

The reaction was carried out according to the general procedure ***GP4*** using eneallene **1** (39 mg, 0.2 mmol) and allene **S28** (43 mg, 0.3 mmol), and the desired product was obtained after purification by silica gel column chromatography (eluent: Et_2O/pentane (5: 95)) as a colorless oil (63 mg, 94% yield). ^{1}H NMR (400 MHz, Chloroform-d) δ 7.57 – 7.52 (m, 2H), 7.30 – 7.25 (m, 2H), 7.21 – 7.16 (m, 1H), 6.65 (s, 1H), 5.62 (ddt, J = 17.1, 10.0, 7.3 Hz, 1H), 5.32 (s, 1H), 5.14 (s, 1H), 5.05 – 4.88 (m, 3H), 4.80 (s, 1H), 4.18 (q, J = 7.1 Hz, 2H), 3.51 – 3.35 (m, 2H), 2.90 (t, J = 6.6 Hz, 2H), 2.08 (s, 3H), 1.64 (s, 3H), 1.29 (t, J = 7.1 Hz, 3H). ^{13}C NMR (101 MHz, Chloroform-d) δ 172.16, 143.20, 142.67, 140.89, 139.42, 137.26, 135.58, 130.07, 129.60, 128.12, 127.71, 127.27, 117.47, 117.01, 116.34, 60.63, 39.05, 37.28, 22.47, 21.16, 14.43. HRMS-ESI: Found $[M+Na]^+$ =359.1983; $C_{23}H_{28}O_2Na$ requires 359.1982.

Ethyl (*E*)-3-allyl-5-((*Z*)-4-methoxybenzylidene)-6-methyl-4-(prop-1-en-2-yl)hepta-3,6-dienoate

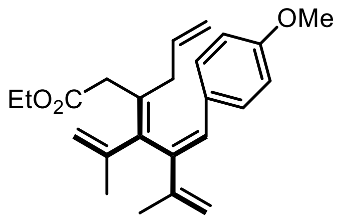

31

The reaction was carried out according to the general procedure ***GP4*** using eneallene **1** (39 mg, 0.2 mmol) and allene **S29** (52 mg, 0.3 mmol), and the desired product was obtained after purification by silica gel column chromatography (eluent: Et_2O/pentane (5: 95)) as a colorless oil (62 mg, 85% yield). ^{1}H NMR (400 MHz, Chloroform-d) δ 7.57 – 7.48 (m, 2H), 6.86 – 6.78 (m, 2H), 6.59 (s, 1H), 5.63 (ddt, J = 17.1, 9.9, 7.3 Hz, 1H), 5.25 (d, J = 1.8 Hz, 1H), 5.09 (s, 1H), 5.03 – 4.92 (m, 3H), 4.85 (d, J = 1.7 Hz, 1H), 4.18 (q, J = 7.1 Hz, 2H), 3.81 (s, 3H), 3.44 (d, J = 2.5 Hz, 2H), 2.95 – 2.83 (m, 3H), 2.06 (s, 3H), 1.65 (s, 3H), 1.29 (t, J = 7.1 Hz, 3H). ^{13}C NMR (101 MHz, Chloroform-d) δ 172.24, 158.87, 143.29, 142.73, 139.61, 138.77, 135.67, 131.02, 129.96, 129.95, 127.14, 117.39, 116.25, 116.11, 113.56, 60.62, 55.34, 38.99, 37.29, 22.46, 21.19, 14.44. HRMS-ESI: Found $[M+Na]^+$ =389.2086; $C_{24}H_{30}O_3Na$ requires 389.2087.

Ethyl (*E*)-3-allyl-5-((*Z*)-4-chlorobenzylidene)-6-methyl-4-(prop-1-en-2-yl)hepta-3,6-dienoate

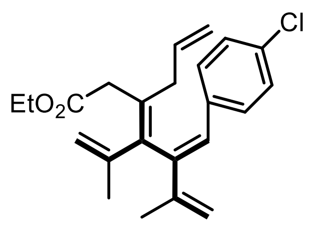

32

The reaction was carried out according to the general procedure ***GP4*** using eneallene **1** (39 mg, 0.2 mmol) and allene **S30** (53 mg, 0.3 mmol), and the desired product was obtained after purification by silica gel column chromatography (eluent: Et_2O/pentane (5: 95)) as a colorless oil (62 mg, 84% yield). ^{1}H NMR (400 MHz, Chloroform-*d*) δ 7.53 – 7.49 (m, 2H), 7.27 – 7.22 (m, 2H), 6.59 (s, 1H), 5.63 (ddt, *J* = 17.1, 10.0, 7.2 Hz, 1H), 5.31 (d, *J* = 1.8 Hz, 1H), 5.16 (s, 1H), 5.05 – 4.97 (m, 1H), 5.01 – 4.88 (m, 2H), 4.78 (dd, *J* = 2.3, 1.0 Hz, 1H), 4.18 (q, *J* = 7.1 Hz, 2H), 3.42 (d, *J* = 4.3 Hz, 2H), 2.96 – 2.80 (m, 2H), 2.06 (s, 3H), 1.63 (s, 3H), 1.29 (t, *J* = 7.1 Hz, 3H). ^{13}C NMR (101 MHz, Chloroform-*d*) δ 172.10, 142.90, 142.47, 141.49, 139.14, 135.67, 135.35, 132.88, 130.87, 130.40, 128.30, 126.37, 117.65, 117.48, 116.50, 60.69, 39.01, 37.18, 22.45, 21.07, 14.44. HRMS-ESI: Found $[M+Na]^+$ =393.1597; $C_{23}H_{27}ClO_2Na$ requires 393.1592.

Ethyl (*E*)-3-allyl-5-((*Z*)-4-fluorobenzylidene)-6-methyl-4-(prop-1-en-2-yl)hepta-3,6-dienoate

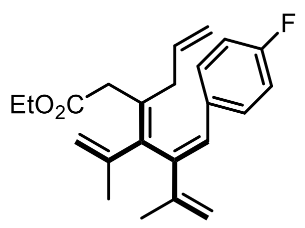

33

The reaction was carried out according to the general procedure ***GP4*** using eneallene **1** (39 mg, 0.2 mmol) and allene **S31** (48 mg, 0.3 mmol), and the desired product was obtained after purification by silica gel column chromatography (eluent: Et_2O/pentane (5: 95)) as a colorless oil (65 mg, 91% yield). ^{1}H NMR (400 MHz, Chloroform-*d*) δ 7.57 – 7.50 (m, 2H), 7.02 – 6.91 (m, 2H), 6.60 (s, 1H), 5.63 (ddt, *J* = 17.1, 10.0, 7.3 Hz, 1H), 5.29 (d, *J* = 1.9 Hz, 1H), 5.14 (s, 1H), 5.05 – 4.94 (m, 2H), 4.90 (dd, *J* = 2.3, 1.5 Hz, 1H), 4.77 (dd, *J* = 2.3, 0.9 Hz, 1H), 4.17 (q, *J* = 7.1 Hz, 2H), 3.42 (d, *J* = 2.3 Hz, 2H), 2.98 – 2.80 (m, 2H), 2.06 (s, 3H), 1.63 (s, 3H), 1.29 (t, *J* = 7.1 Hz, 3H). ^{13}C NMR (101 MHz, Chloroform-*d*) δ 172.14, 161.99 (d, *J* = 247.6 Hz), 143.03, 142.54, 140.58 (d, *J* = 2.0 Hz), 139.23, 135.43, 133.35 (d, *J* = 3.4 Hz), 131.29, 131.21, 130.36, 117.60, 117.05, 116.36, 115.12, 114.91, 60.67, 39.01, 37.18, 22.45, 21.10, 14.44. ^{19}F NMR (377 MHz, Chloroform-*d*) δ -114.62. HRMS-ESI: Found $[M+Na]^+$ =377.1890; $C_{23}H_{27}FO_2Na$ requires 377.1887.

2-((*E*)-3-Allyl-5-((*Z*)-4-methoxybenzylidene)-6-methyl-4-(prop-1-en-2-yl)hepta-3,6-dien-1-yl)isoindoline-1,3-dione

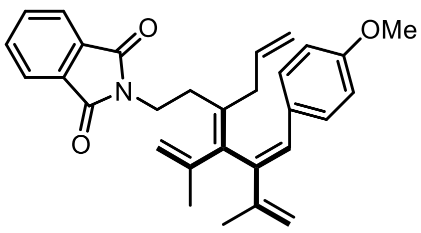

34

The reaction was carried out according to the general procedure ***GP4*** using eneallene **S7** (56 mg, 0.2 mmol) and allene **S29** (52 mg, 0.3 mmol), and the desired product was obtained after purification by silica gel column chromatography (eluent: Et_2O/pentane (5: 95)) as a colorless oil (80 mg, 89% yield). ^{1}H NMR (400 MHz, Chloroform-*d*) δ 7.84 (dd, *J* = 5.5, 3.0 Hz, 2H), 7.71 (dd, *J* = 5.5, 3.1 Hz, 2H), 7.47 – 7.38 (m, 2H), 6.83 – 6.75 (m, 2H), 6.57 (s, 1H), 5.69 (ddt, *J* = 17.1, 9.9, 7.2 Hz, 1H), 5.12 (dq, *J* = 17.1, 1.6 Hz, 1H), 5.10 – 4.94 (m, 4H), 4.81 (dd, *J* = 2.1, 0.9 Hz, 1H), 3.89 – 3.80 (m, 2H), 3.79 (s, 3H), 3.01 – 2.68 (m, 4H), 2.03 (s, 3H), 1.72 (s, 3H). ^{13}C NMR (101 MHz, Chloroform-*d*) δ 168.28, 158.82, 143.31, 142.92, 139.83, 138.60, 135.75, 134.01, 133.18, 132.36, 130.69, 130.13, 123.26, 117.34, 116.02, 115.69, 113.51, 55.32, 38.09, 36.63, 30.63, 22.97, 21.17. HRMS-ESI: Found $[M+Na]^+$ =476.2197; $C_{30}H_{31}NO_3Na$ requires 476.2196.

Diethyl (2*Z*,4*E*)-5-allyl-4-(prop-1-en-2-yl)-3-vinylhepta-2,4-dienedioate

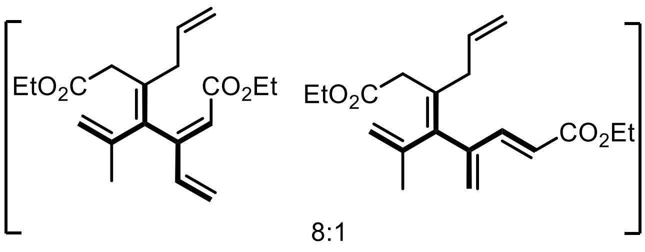

35

The reaction was carried out according to the general procedure ***GP4*** using eneallene **1** (39 mg, 0.2 mmol) and allene **S32** (38 mg, 0.3 mmol), and the desired product was obtained after purification by silica gel column chromatography (eluent: Et_2O/pentane (5: 95)) as a colorless oil (34 mg, 54% yield). ^{1}H NMR (400 MHz, Chloroform-*d*) δ 6.37 (dd, *J* = 17.2, 10.4 Hz, 1H), 5.87 (s, 1H), 5.83 (d, *J* = 17.2 Hz, 1H), 5.64 (ddt, *J* = 17.0, 10.0, 7.1 Hz, 1H), 5.46 (d, *J* = 10.4 Hz, 1H), 5.04 – 4.94 (m, 4H), 4.13 (q, *J* = 7.1 Hz, 4H), 3.60 (d, *J* = 15.5 Hz, 1H), 3.10 (d, *J* = 14.5 Hz, 1H), 2.82 – 2.61 (m, 2H), 1.72 (s, 3H), 1.25 (q, *J* = 7.2 Hz, 6H). Distinguishing peaks of minor isomer: ^{1}H NMR (400 MHz, Chloroform-*d*) δ 7.75 (dd, *J* = 17.5, 10.6 Hz, 1H), 5.53 (dt, *J* = 10.3, 1.6 Hz, 1H). ^{13}C NMR (101 MHz, Chloroform-*d*) δ 172.28, 165.71, 153.45, 143.19, 137.29, 136.53, 135.76, 128.62, 123.64, 121.13, 117.03, 116.87, 60.63, 60.09, 38.86, 36.95, 22.54, 14.37, 14.36. Minor isomer: ^{13}C NMR (101 MHz, Chloroform-*d*) δ 172.28, 165.71, 153.45, 143.19, 137.29, 136.53, 135.76, 128.62, 123.64, 121.13, 117.03, 116.87, 60.63, 60.09,

38.86, 36.95, 22.54, 14.37, 14.36. HRMS-ESI: Found $[M+Na]^+$ =341.1726; $C_{19}H_{26}O_4Na$ requires 341.1723.

Diethyl (2*Z*,4*E*)-5-allyl-3-(cyclobut-1-en-1-yl)-4-(prop-1-en-2-yl)hepta-2,4-dienedioate

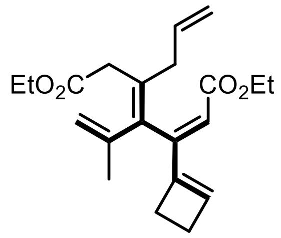

36

The reaction was carried out according to the general procedure ***GP4*** using eneallene **1** (39 mg, 0.2 mmol) and allene **S33** (45 mg, 0.3 mmol), and the desired product was obtained after purification by silica gel column chromatography (eluent: Et_2O/pentane (5: 95)) as a colorless oil (50 mg, 73% yield). ^{1}H NMR (400 MHz, Chloroform-*d*) δ 6.39 (s, 1H), 5.74 (s, 1H), 5.63 (ddt, *J* = 17.0, 9.9, 7.0 Hz, 1H), 5.05 – 4.93 (m, 4H), 4.17 – 4.07 (m, 4H), 3.52 (d, *J* = 16.1 Hz, 1H), 3.07 (d, *J* = 16.1 Hz, 1H), 2.80 (dd, *J* = 14.3, 6.4 Hz, 1H), 2.72 – 2.57 (m, 3H), 2.43 (s, 2H), 1.73 (s, 3H), 1.24 (td, *J* = 7.1, 4.5 Hz, 6H). ^{13}C NMR (101 MHz, Chloroform-*d*) δ 172.24, 166.09, 149.42, 146.14, 143.47, 139.60, 136.97, 136.05, 127.61, 116.82, 116.59, 115.79, 60.53, 60.01, 38.77, 36.88, 28.54, 26.40, 22.63, 14.38, 14.36. HRMS-ESI: Found $[M+Na]^+$ =367.1878; $C_{21}H_{28}O_4Na$ requires 367.1880.

Ethyl (2*Z*,4*E*)-5-benzyl-3-(cyclohex-1-en-1-yl)-4-(prop-1-en-2-yl)octa-2,4,7-trienoate

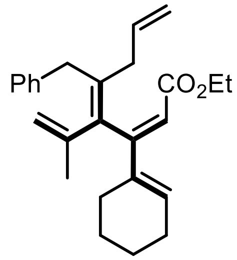

37

The reaction was carried out according to the general procedure ***GP4*** using eneallene **S3** (40 mg, 0.2 mmol) and allene **S34** (46 mg, 0.3 mmol), and the desired product was obtained after purification by silica gel column chromatography (eluent: Et_2O/pentane (5: 95)) as a colorless oil (46 mg, 61% yield). ^{1}H NMR (400 MHz, Chloroform-*d*) δ 7.31 – 7.27 (m, 4H), 7.22 – 7.17 (m, 1H), 6.32 (t, *J* = 5.2 Hz, 1H), 5.89 (s, 1H), 5.65 (ddt, *J* = 17.1, 10.0, 7.0 Hz, 1H), 5.02 (s, 2H), 4.96 – 4.88 (m, 1H), 4.82 (dq, *J* = 16.9, 1.7 Hz, 1H), 4.13 (q, *J* = 7.1 Hz, 2H), 3.73 (d, *J* = 10.0 Hz, 2H), 2.57 (dt, *J* = 7.0, 1.4 Hz, 2H), 2.24 (d, *J* = 35.5 Hz, 4H), 1.82 (s, 3H), 1.72 (s, 2H), 1.66 – 1.53 (m, 2H), 1.25 (t, *J* = 7.1 Hz, 3H). ^{13}C NMR (101 MHz, Chloroform-*d*) δ 166.50, 156.58, 143.86, 140.84, 136.44, 136.14, 135.25, 135.08, 133.51, 129.28, 128.33, 125.79, 116.27, 115.98, 114.24, 59.79, 37.17, 26.57, 25.93, 23.44, 22.94, 22.04, 14.50. HRMS-ESI: Found $[M+Na]^+$ =399.2298; $C_{26}H_{32}O_2Na$ requires 399.2295.

7-Ethyl 1-((1*S*,2*R*,5*S*)-2-isopropyl-5-methylcyclohexyl) (2*Z*,4*E*)-5-allyl-3,4-di(prop-1-en-2-yl)hepta-2,4-dienedioate

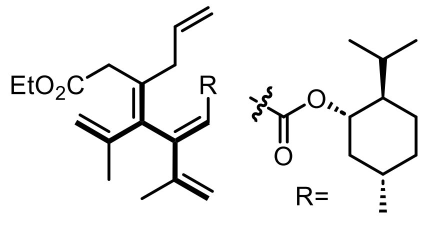

57

The reaction was carried out according to the general procedure ***GP4*** using eneallene **1** (39 mg, 0.2 mmol) and allene **S47** (74 mg, 0.3 mmol), and the desired product was obtained after purification by silica gel column chromatography (eluent: Et_2O/pentane (5: 95)) as a colorless oil (77 mg, 87% yield). ^1H NMR (400 MHz, Chloroform-*d*) δ 5.96 (s, 1H), 5.72 (s, 1H), 5.70 – 5.57 (m, 1H), 5.36 (s, 1H), 5.05 – 4.94 (m, 4H), 4.73 – 4.65 (m, 1H), 4.13 (q, *J* = 7.1 Hz, 2H), 3.61 (d, *J* = 16.1 Hz, 1H), 3.07 (d, *J* = 16.2 Hz, 1H), 2.71 (dd, *J* = 17.9, 7.0 Hz, 2H), 1.97 (s, 3H), 1.71 (s, 3H), 1.69 – 1.63 (m, 2H), 1.53 – 1.33 (m, 3H), 1.25 (t, *J* = 7.1 Hz, 3H), 1.21 – 0.92 (m, 3H), 0.91 – 0.86 (m, 9H), 0.77 – 0.69 (m, 3H). ^{13}C NMR (101 MHz, Chloroform-*d*) δ 172.52, 165.50, 155.23, 143.51, 140.81, 137.96, 136.02, 128.15, 123.00, 117.50, 117.01, 116.72, 73.64, 60.57, 47.20, 41.22, 39.14, 37.13, 34.43, 31.56, 26.18, 23.50, 22.55, 22.18, 20.95, 20.52, 16.33, 14.36. HRMS-ESI: Found $[M+Na]^+$ =465.2977; $C_{28}H_{42}O_4Na$ requires 465.2975.

Ethyl (*R*,2*Z*,4*Z*)-5-allyl-8,12-dimethyl-3,4-di(prop-1-en-2-yl)trideca-2,4,11-trienoate

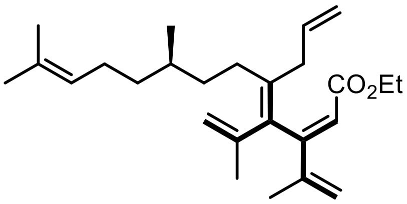

58

The reaction was carried out according to the general procedure ***GP4*** using eneallene **S22** (49 mg, 0.2 mmol) and allene **2** (42 mg, 0.3 mmol), and the desired product was obtained after purification by silica gel column chromatography (eluent: Et_2O/pentane (5: 95)) as a colorless oil (69 mg, 90% yield). ^1H NMR (400 MHz, Chloroform-*d*) δ 5.94 (s, 1H), 5.76 – 5.60 (m, 1H), 5.43 (d, *J* = 1.9 Hz, 1H), 5.29 (s, 1H), 5.10 (dtd, *J* = 7.1, 3.6, 2.7, 1.4 Hz, 1H), 5.03 – 4.90 (m, 3H), 4.88 (dd, *J* = 2.7, 1.0 Hz, 1H), 4.12 (q, *J* = 7.1 Hz, 2H), 2.67 (dt, *J* = 7.0, 1.4 Hz, 2H), 2.38 – 2.14 (m, 2H), 2.05 – 1.98 (m, 1H), 1.95 (s, 3H), 1.93 – 1.87 (m, 1H), 1.75 (s, 3H), 1.68 (s, 3H), 1.60 (s, 3H), 1.43 – 1.30 (m, 3H), 1.26 (t, *J* = 7.1 Hz, 3H), 1.20 – 1.09 (m, 2H), 0.88 (d, *J* = 6.4 Hz, 3H). ^{13}C NMR (101 MHz, Chloroform-*d*) δ 166.29, 155.79, 144.00, 141.67, 136.87, 136.18, 133.71, 131.13, 125.16, 121.87, 117.36, 115.94, 115.74, 59.96, 37.86, 36.98, 35.50, 33.07, 28.73, 25.88, 25.74, 23.22, 20.56, 19.70, 17.79, 14.47. HRMS-ESI: Found $[M+Na]^+$ =407.2921; $C_{26}H_{40}O_2Na$ requires 407.2921.

(1*S*,2*R*,5*S*)-2-Isopropyl-5-methylcyclohexyl (*R*,2*Z*,4*Z*)-5-allyl-8,12-dimethyl-3,4-di(prop-1-en-2-yl)trideca-2,4,11-trienoate

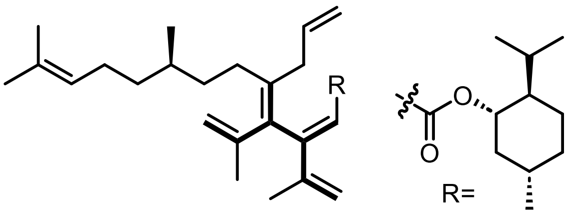

59

The reaction was carried out according to the general procedure ***GP4*** using eneallene **S22** (49 mg, 0.2 mmol) and allene **47** (75 mg, 0.3 mmol), and the desired product was obtained after purification by silica gel column chromatography (eluent: Et_2O/pentane (5: 95)) as a colorless oil (89 mg, 88% yield). ^{1}H NMR (400 MHz, Chloroform-*d*) δ 5.92 (s, 1H), 5.77 – 5.62 (m, 1H), 5.44 (s, 1H), 5.28 (s, 1H), 5.14 – 5.05 (m, 1H), 5.03 – 4.86 (m, 4H), 4.70 (td, *J* = 10.9, 4.3 Hz, 1H), 2.67 (d, *J* = 7.1 Hz, 2H), 2.39 – 2.14 (m, 2H), 2.00 (d, *J* = 10.3 Hz, 2H), 1.95 (s, 3H), 1.93 – 1.85 (m, 2H), 1.75 (s, 3H), 1.68 (s, 3H), 1.67 – 1.63 (m, 1H), 1.60 (s, 3H), 1.52 – 1.29 (m, 7H), 1.21 – 0.94 (m, 4H), 0.92 – 0.87 (m, 9H), 0.86 – 0.82 (m, 1H), 0.74 (s, 3H). ^{13}C NMR (101 MHz, Chloroform-*d*) δ 165.57, 144.01, 141.70, 137.06, 136.08, 133.69, 131.05, 125.19, 121.74, 117.52, 115.91, 115.73, 73.42, 47.25, 41.27, 37.90, 35.45, 34.48, 33.07, 31.56, 28.77, 26.15, 25.87, 25.73, 23.50, 23.22, 22.21, 20.99, 20.58, 19.70, 17.79, 16.36. HRMS-ESI: Found $[M+Na]^+$ =517.4016; $C_{34}H_{54}O_2Na$ requires 517.4016.

(2*R*,4*S*)-1,7,7-Trimethylbicyclo[2.2.1]heptan-2-yl (2*Z*,4*E*)-5-(4-methoxyphenyl)-3,4-di(prop-1-en-2-yl)octa-2,4,7-trienoate

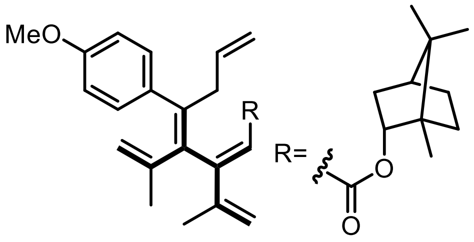

60

The reaction was carried out according to the general procedure ***GP4*** using eneallene **S11** (43 mg, 0.2 mmol) and allene **S48** (74 mg, 0.3 mmol), and the desired product was obtained after purification by silica gel column chromatography (eluent: Et_2O/pentane (5: 95)) as a colorless oil (68 mg, 74% yield). ^{1}H NMR (400 MHz, Chloroform-*d*) δ 7.29 – 7.25 (m, 2H), 6.85 – 6.81 (m, 2H), 6.05 (s, 1H), 5.60 – 5.46 (m, 2H), 5.35 (s, 1H), 4.97 – 4.87 (m, 1H), 4.86 – 4.73 (m, 4H), 3.80 (s, 3H), 2.98 (dt, *J* = 6.9, 1.5 Hz, 2H), 2.38 (dt, *J* = 13.4, 6.9 Hz, 1H), 2.05 (s, 3H), 2.03 – 1.97 (m, 1H), 1.83 – 1.69 (m, 1H), 1.67 (q, *J* = 4.2 Hz, 1H), 1.49 (s, 3H), 1.35 – 1.23 (m, 3H), 1.00 (dd, *J* = 13.7, 3.6 Hz, 1H), 0.91 (s, 3H), 0.88 (s, 3H), 0.85 (d, *J* = 2.8 Hz, 3H). ^{13}C NMR (101 MHz, Chloroform-*d*) δ 166.33, 158.55, 156.77,

143.83, 141.84, 136.26, 135.85, 134.78, 121.80, 117.63, 117.45, 115.76, 113.31, 79.30, 55.27, 48.97, 47.98, 45.11, 41.33, 37.16, 36.87, 28.22, 27.38, 23.31, 20.60, 19.91, 19.05, 13.69. HRMS-ESI: Found $[M+Na]^+$ =483.2870; $C_{31}H_{40}O_3Na$ requires 483.2870.

(3*S*,8*R*,9*S*,10*R*,13*R*,14*S*)-10,13-Dimethyl-17-((*R*)-6-methylheptan-2-yl)-2,3,4,7,8,9,10,11,12,13,14,15,16,17-tetradecahydro-1H-cyclopenta[a]phenanthren-3-yl (2*Z*,4*Z*,8*R*)-5-allyl-8,12-dimethyl-3,4-di(prop-1-en-2-yl)trideca-2,4,11-trienoate

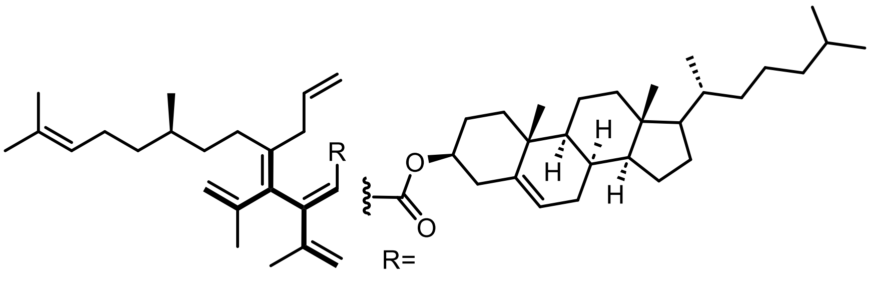

61

The reaction was carried out according to the general procedure ***GP4*** using eneallene **S22** (49 mg, 0.2 mmol) and allene **S49** (144 mg, 0.3 mmol), and the desired product was obtained after purification by silica gel column chromatography (eluent: Et_2O/pentane (5: 95)) as a colorless oil (120 mg, 83% yield). ^{1}H NMR (400 MHz, Chloroform-*d*) δ 5.93 (s, 1H), 5.70 (ddt, *J* = 17.0, 10.0, 7.0 Hz, 1H), 5.42 (s, 1H), 5.37 (d, *J* = 3.6 Hz, 2H), 5.28 (s, 1H), 5.15 – 5.05 (m, 1H), 5.03 – 4.90 (m, 3H), 4.89 (d, *J* = 2.6 Hz, 1H), 4.67 – 4.56 (m, 1H), 2.68 (d, *J* = 7.0 Hz, 2H), 2.32 (d, *J* = 7.6 Hz, 2H), 2.27 – 1.96 (m, 4H), 1.95 (s, 3H), 1.91 – 1.78 (m, 3H), 1.76 (s, 3H), 1.68 (s, 3H), 1.60 (s, 3H), 1.58 – 1.03 (m, 25H), 1.02 (s, 3H), 0.99 – 0.94 (m, 2H), 0.92 (d, *J* = 6.5 Hz, 3H), 0.90 – 0.87 (m, 6H), 0.86 (d, *J* = 1.7 Hz, 3H), 0.68 (s, 3H). ^{13}C NMR (101 MHz, Chloroform-*d*) δ 165.58, 155.52, 143.99, 141.67, 139.90, 136.94, 136.07, 133.76, 131.06, 125.18, 122.69, 121.72, 117.73, 115.95, 115.74, 73.56, 56.83, 56.30, 50.21, 42.46, 39.89, 39.67, 38.41, 37.89, 37.21, 36.99, 36.76, 36.34, 35.95, 35.50, 33.08, 32.04 (d, *J* = 2.6 Hz), 28.82, 28.39, 28.16, 28.04, 25.88, 25.74, 24.44, 23.99, 23.26, 22.97, 22.72, 21.18, 20.57, 19.72, 19.49, 18.87, 17.81, 12.01. HRMS-ESI: Found $[M+Na]^+$ =747.6063; $C_{51}H_{80}O_2Na$ requires 747.6059.

((3a*S*,5*S*,5a*R*,8a*R*,8b*S*)-2,2,7,7-Tetramethyltetrahydro-5H-bis([1,3]dioxolo)[4,5-b:4',5'-d]pyran-5-yl)methyl (2*Z*,4*E*)-5-(2-(benzyloxy)ethyl)-3,4-di(prop-1-en-2-yl)octa-2,4,7-trienoate

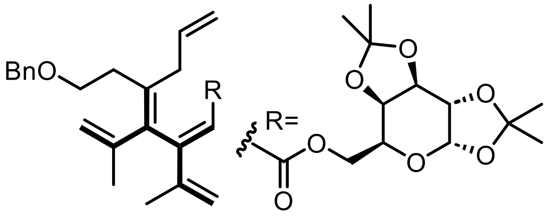

62

The reaction was carried out according to the general procedure ***GP4*** using eneallene **S5** (48 mg, 0.2 mmol) and allene **S50** (106 mg, 0.3 mmol), and the desired product was obtained after purification by silica gel column chromatography (eluent: Et_2O/pentane (5: 95)) as a colorless oil (101 mg, 85% yield). ^{1}H NMR (400 MHz, Chloroform-*d*) δ 7.35 – 7.31 (m, 4H), 7.29 – 7.24 (m, 1H), 6.00 (s, 1H), 5.74 – 5.59 (m, 1H), 5.53 (d, *J* = 5.0 Hz, 1H), 5.48 (s, 1H), 5.28 (s, 1H), 5.00 – 4.90 (m, 4H), 4.61 (dd, *J* = 7.9, 2.5 Hz, 1H), 4.55 – 4.46 (m, 2H), 4.35 – 4.24 (m, 2H), 4.24 (dd, *J* = 7.9, 1.8 Hz, 1H), 4.17 (s, 1H), 4.06 – 3.98 (m, 1H), 3.62 (d, *J* = 6.3 Hz, 2H), 2.75 (s, 1H), 2.65 (d, *J* = 7.0 Hz, 2H), 2.58 (s, 1H), 1.94 (s, 3H), 1.72 (s, 3H), 1.50 (s, 3H), 1.45 (s, 3H), 1.33 (d, *J* = 5.3 Hz, 6H). ^{13}C NMR (101 MHz, Chloroform-*d*) δ 165.62, 143.55, 141.30, 138.80, 136.61, 135.79, 132.16, 128.40, 127.70, 127.53, 122.65, 116.67, 116.43, 116.31, 109.65, 108.83, 96.44, 72.85, 71.20, 70.81, 70.58, 68.98, 66.09, 62.95, 38.25, 31.41, 26.18, 26.11, 25.09, 24.64, 23.18, 20.51. HRMS-ESI: Found $[M+Na]^+$ =617.3091; $C_{35}H_{46}O_8Na$ requires 617.3091.

2.4 Characterization of Type-II [4]Dendralene Products

Diethyl (2*E*,5*E*)-6-allyl-5-(prop-1-en-2-yl)-4-(propan-2-ylidene)octa-2,5-dienedioate

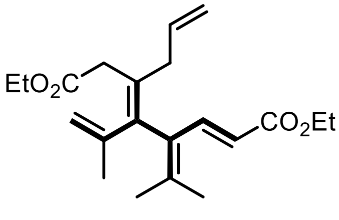

38

The reaction was carried out according to the general procedure ***GP4*** using eneallene **1** (39 mg, 0.2 mmol) and allene **S35** (46 mg, 0.3 mmol), and the desired product was obtained after purification by silica gel column chromatography (eluent: Et_2O/pentane (5: 95)) as a colorless oil (62 mg, 89% yield). ^{1}H NMR (400 MHz, Chloroform-*d*) δ 7.78 (d, *J* = 15.4 Hz, 1H), 5.84 (d, *J* = 15.4 Hz, 1H), 5.69 – 5.54 (m, 1H), 4.99 (s, 2H), 4.96 (d, *J* = 7.2 Hz, 1H), 4.86 (s, 1H), 4.22 – 4.10 (m, 4H), 3.36 (d, *J* = 5.6 Hz, 2H), 2.67 (d, *J* = 7.0 Hz, 2H), 2.00 (s, 3H), 1.85 (s, 3H), 1.66 (s, 3H), 1.28 (td, *J* = 7.1, 3.8 Hz, 6H). ^{13}C NMR (101 MHz, Chloroform-*d*) δ 172.09, 168.21, 144.11, 143.97, 141.27, 135.59, 131.52, 129.78, 118.70, 117.15,

115.82, 60.71, 60.27, 38.31, 37.18, 23.92, 22.46, 20.52, 14.45, 14.31. HRMS-ESI: Found $[M+Na]^+$ =369.2037; $C_{21}H_{30}O_4Na$ requires 369.2036.

Ethyl (3*E*,6*E*)-3-allyl-8-oxo-4-(prop-1-en-2-yl)-5-(propan-2-ylidene)nona-3,6-dienoate

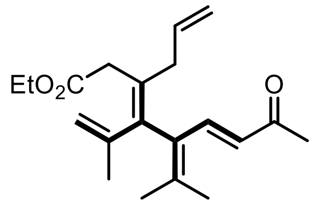

39

The reaction was carried out according to the general procedure ***GP4*** using eneallene **1** (39 mg, 0.2 mmol) and allene **S36** (37 mg, 0.3 mmol), and the desired product was obtained after purification by silica gel column chromatography (eluent: Et_2O/pentane (5: 95)) as a colorless oil (52 mg, 82% yield). ^1H NMR (400 MHz, Chloroform-*d*) δ 7.63 (d, *J* = 15.6 Hz, 1H), 6.16 (d, *J* = 15.6 Hz, 1H), 5.61 (ddt, *J* = 16.2, 10.7, 7.1 Hz, 1H), 5.00 (dt, *J* = 2.8, 1.0 Hz, 2H), 5.00 – 4.92 (m, 1H), 4.86 (dd, *J* = 2.2, 0.8 Hz, 1H), 4.14 (q, *J* = 7.1 Hz, 2H), 3.44 – 3.28 (m, 2H), 2.66 (dt, *J* = 7.2, 1.3 Hz, 2H), 2.27 (s, 3H), 2.02 (s, 3H), 1.88 (s, 3H), 1.66 – 1.65 (m, 3H), 1.27 (t, *J* = 7.1 Hz, 3H). ^{13}C NMR (101 MHz, Chloroform-*d*) δ 199.17, 172.11, 145.37, 143.91, 139.79, 139.32, 135.53, 131.82, 129.93, 127.98, 117.21, 115.92, 60.70, 38.30, 37.05, 28.11, 24.12, 22.44, 20.61, 14.37. HRMS-ESI: Found $[M+Na]^+$ =339.1934; $C_{20}H_{28}O_3Na$ requires 339.1931.

Ethyl (3*E*,6*E*)-3-allyl-8-oxo-8-phenyl-4-(prop-1-en-2-yl)-5-(propan-2-ylidene)octa-3,6-dienoate

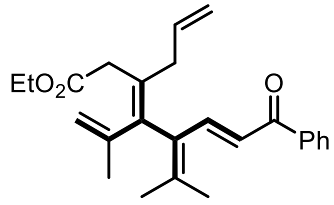

40

The reaction was carried out according to the general procedure ***GP4*** using eneallene **1** (39 mg, 0.2 mmol) and allene **S37** (56 mg, 0.3 mmol), and the desired product was obtained after purification by silica gel column chromatography (eluent: Et_2O/pentane (5: 95)) as a colorless oil (59 mg, 78% yield). ^1H NMR (400 MHz, Chloroform-*d*) δ 8.06 – 7.98 (m, 2H), 7.97 (d, *J* = 15.0 Hz, 1H), 7.58 – 7.49 (m, 1H), 7.51 – 7.42 (m, 2H), 7.13 (d, *J* = 15.1 Hz, 1H), 5.65 (ddt, *J* = 16.3, 10.8, 7.1 Hz, 1H), 5.07 – 4.89 (m, 4H), 4.16 (q, *J* = 7.1 Hz, 2H), 3.54 (d, *J* = 16.1 Hz, 1H), 3.34 (d, *J* = 16.1 Hz, 1H), 2.79 – 2.67 (m, 2H), 2.07 (s, 3H), 1.90 (s, 3H), 1.71 (dd, *J* = 1.4, 0.8 Hz, 3H), 1.25 (t, *J* = 7.1 Hz, 3H). ^{13}C NMR (101 MHz, Chloroform-*d*) δ 191.12, 172.27, 146.08, 144.22, 141.18, 139.83, 138.76, 135.56, 132.58, 129.92,

128.64, 128.60, 122.99, 117.16, 115.80, 60.70, 38.38, 36.97, 24.28, 22.56, 20.73, 14.39. HRMS-ESI: Found $[M+Na]^+$ =401.2087; $C_{25}H_{30}O_3Na$ requires 401.2087.

Ethyl (3*E*,6*E*)-3-allyl-8-oxo-4-(prop-1-en-2-yl)-5-(propan-2-ylidene)octa-3,6-dienoate

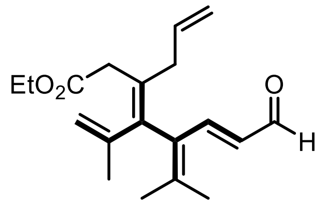

41

The reaction was carried out according to the general procedure ***GP4*** using eneallene **1** (39 mg, 0.2 mmol) and allene **S38** (33 mg, 0.3 mmol), and the desired product was obtained after purification by silica gel column chromatography (eluent: Et_2O/pentane (5: 95)) as a colorless oil (37 mg, 61% yield). ^{1}H NMR (400 MHz, Chloroform-*d*) δ 9.61 (d, *J* = 8.0 Hz, 1H), 7.58 (d, *J* = 15.3 Hz, 1H), 6.16 (dd, *J* = 15.3, 8.0 Hz, 1H), 5.59 (ddt, *J* = 16.6, 10.4, 7.1 Hz, 1H), 5.00 (ddt, *J* = 7.9, 3.1, 1.6 Hz, 2H), 5.00 – 4.92 (m, 1H), 4.87 (dd, *J* = 2.2, 0.9 Hz, 1H), 4.14 (q, *J* = 7.1 Hz, 2H), 3.43 – 3.28 (m, 2H), 2.66 (dt, *J* = 7.1, 1.3 Hz, 2H), 2.06 (s, 3H), 1.92 (s, 3H), 1.65 (s, 3H), 1.27 (t, *J* = 7.1 Hz, 3H). ^{13}C NMR (101 MHz, Chloroform-*d*) δ 194.44, 171.94, 149.32, 147.13, 143.66, 139.01, 135.27, 132.22, 130.34, 129.85, 117.37, 116.19, 60.79, 38.30, 37.11, 24.24, 22.38, 20.68, 14.34. HRMS-ESI: Found $[M+Na]^+$ =325.1772; $C_{19}H_{26}O_3Na$ requires 325.1774.

Ethyl (3*E*,6*E*)-3-allyl-8-(dimethylamino)-8-oxo-4-(prop-1-en-2-yl)-5-(propan-2-ylidene)octa-3,6-dienoate

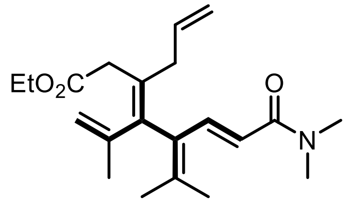

42

The reaction was carried out according to the general procedure ***GP4*** using eneallene **1** (39 mg, 0.2 mmol) and allene **S39** (46 mg, 0.3 mmol), and the desired product was obtained after purification by silica gel column chromatography (eluent: Et_2O/pentane (5: 95)) as a colorless oil (44 mg, 64% yield). ^{1}H NMR (400 MHz, Chloroform-*d*) δ 7.72 (d, *J* = 14.8 Hz, 1H), 6.44 (d, *J* = 14.9 Hz, 1H), 5.69 – 5.54 (m, 1H), 4.99 (dq, *J* = 2.4, 1.3 Hz, 2H), 4.98 – 4.91 (m, 1H), 4.83 (dd, *J* = 2.5, 0.9 Hz, 1H), 4.11 (q, *J* = 7.1 Hz, 2H), 3.51 (d, *J* = 16.2 Hz, 1H), 3.24 (d, *J* = 16.2 Hz, 1H), 3.07 (s, 3H), 3.00 (s, 3H), 2.76 – 2.60 (m, 2H), 1.99 (s, 3H), 1.81 (s, 3H), 1.66 (s, 3H), 1.25 (t, *J* = 7.1 Hz, 3H). ^{13}C NMR (101 MHz, Chloroform-*d*) δ 172.41, 168.06, 144.40, 141.69, 140.30, 138.62, 135.79, 131.74, 129.43, 118.54, 116.91, 115.44,

60.57, 38.33, 37.40, 36.78, 35.93, 23.91, 22.54, 20.38, 14.38. HRMS-ESI: Found $[M+Na]^+$ =368.2194; $C_{21}H_{31}NO_3Na$ requires 368.2196.

Ethyl (*E*)-3-allyl-5-((*E*)-2-cyanovinyl)-6-methyl-4-(prop-1-en-2-yl)hepta-3,5-dienoate

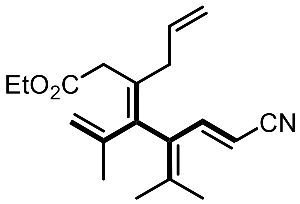

43

The reaction was carried out according to the general procedure ***GP4*** using eneallene **1** (39 mg, 0.2 mmol) and allene **S40** (32 mg, 0.3 mmol), and the desired product was obtained after purification by silica gel column chromatography (eluent: Et_2O/pentane (5: 95)) as a colorless oil (40 mg, 67% yield). ^{1}H NMR (400 MHz, Chloroform-*d*) δ 7.47 (d, *J* = 16.1 Hz, 1H), 5.67 – 5.52 (m, 1H), 5.46 (d, *J* = 16.1 Hz, 1H), 5.05 – 4.93 (m, 3H), 4.82 (s, 1H), 4.13 (q, *J* = 7.1 Hz, 2H), 3.46 (d, *J* = 16.2 Hz, 1H), 3.26 (d, *J* = 16.2 Hz, 1H), 2.70 – 2.57 (m, 2H), 1.97 (s, 3H), 1.86 (s, 3H), 1.65 (s, 3H), 1.26 (t, *J* = 7.1 Hz, 3H). ^{13}C NMR (101 MHz, Chloroform-*d*) δ 171.95, 146.70, 145.11, 143.49, 138.16, 135.05, 131.45, 131.19, 119.69, 117.50, 116.31, 96.54, 60.86, 38.32, 36.95, 24.08, 22.35, 20.46, 14.36. HRMS-ESI: Found $[M+Na]^+$ =322.1777; $C_{19}H_{25}NO_2Na$ requires 322.1778.

Ethyl (*E*)-3-allyl-5-((*E*)-2-(diethoxyphosphoryl)vinyl)-6-methyl-4-(prop-1-en-2-yl)hepta-3,5-dienoate

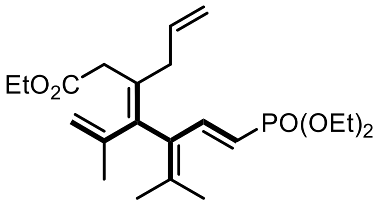

44

The reaction was carried out according to the general procedure ***GP4*** using eneallene **1** (39 mg, 0.2 mmol) and allene **S41** (65 mg, 0.3 mmol), and the desired product was obtained after purification by silica gel column chromatography (eluent: EtOAc/pentane (10: 90)) as a colorless oil (49 mg, 60% yield). ^{1}H NMR (400 MHz, Chloroform-*d*) δ 7.56 (dd, *J* = 21.8, 16.9 Hz, 1H), 5.73 – 5.55 (m, 2H), 5.03 – 4.94 (m, 3H), 4.83 (s, 1H), 4.13 (q, *J* = 7.1 Hz, 2H), 4.08 – 3.98 (m, 4H), 3.42 – 3.30 (m, 2H), 2.66 (d, *J* = 7.1 Hz, 2H), 1.98 (s, 3H), 1.84 (s, 3H), 1.65 (s, 3H), 1.33 – 1.23 (m, 9H). ^{31}P NMR (162 MHz, Chloroform-*d*) δ 21.81. ^{13}C NMR (101 MHz, Chloroform-*d*) δ 172.02, 145.13 (d, *J* = 8.6 Hz), 143.93, 143.07, 139.20, 135.50, 131.97 (d, *J* = 24.3 Hz), 130.13, 117.17, 115.72, 114.09 (d, *J* = 189.0 Hz), 61.56, 61.52, 60.66, 38.20, 37.04, 23.86 (d, *J* = 2.3 Hz), 22.42, 20.38, 16.51, 16.45, 14.29. HRMS-ESI: Found $[M+Na]^+$ =433.2123; $C_{22}H_{35}O_5PNa$ requires 433.2114.

Ethyl (*E*)-3-allyl-6-methyl-4-(prop-1-en-2-yl)-5-((*E*)-styryl)hepta-3,5-dienoate

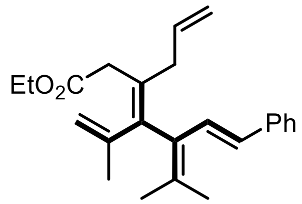

45

The reaction was carried out according to the general procedure ***GP4*** using eneallene **1** (39 mg, 0.2 mmol) and allene **S42** (47 mg, 0.3 mmol), and the desired product was obtained after purification by silica gel column chromatography (eluent: Et_2O/pentane (5: 95)) as a colorless oil (55 mg, 78% yield). ^{1}H NMR (400 MHz, Chloroform-*d*) δ 7.45 – 7.38 (m, 2H), 7.34 – 7.26 (m, 2H), 7.23 – 7.14 (m, 1H), 7.17 (d, *J* = 15.9 Hz, 1H), 6.54 (d, *J* = 15.9 Hz, 1H), 5.69 (ddt, *J* = 17.1, 10.1, 7.2 Hz, 1H), 5.05 – 4.95 (m, 3H), 4.90 (dd, *J* = 2.5, 1.0 Hz, 1H), 4.18 (q, *J* = 7.2 Hz, 2H), 3.42 (d, *J* = 1.2 Hz, 2H), 2.83 – 2.74 (m, 2H), 1.98 (s, 3H), 1.84 (s, 3H), 1.74 (s, 3H), 1.29 (t, *J* = 7.1 Hz, 3H). ^{13}C NMR (101 MHz, Chloroform-*d*) δ 172.44, 144.76, 140.55, 138.55, 136.11, 134.54, 132.57, 129.67, 128.95, 128.65, 127.01, 126.37, 125.84, 116.88, 115.20, 60.58, 38.29, 37.05, 23.50, 22.72, 20.08, 14.45. HRMS-ESI: Found $[M+Na]^+$ =373.2137; $C_{24}H_{30}O_2Na$ requires 373.2138.

Ethyl (3*E*,6*E*)-3-allyl-8-(benzyloxy)-4-(prop-1-en-2-yl)-5-(propan-2-ylidene)octa-3,6-dienoate

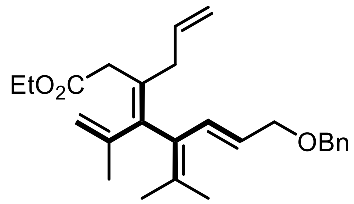

46

The reaction was carried out according to the general procedure ***GP4*** using eneallene **1** (39 mg, 0.2 mmol) and allene **S43** (60 mg, 0.3 mmol), and the desired product was obtained after purification by silica gel column chromatography (eluent: Et_2O/pentane (5: 95)) as a colorless oil (37 mg, 47% yield). ^{1}H NMR (400 MHz, Chloroform-*d*) δ 7.37 – 7.25 (m, 5H), 6.62 (d, *J* = 15.5 Hz, 1H), 5.81 – 5.59 (m, 2H), 5.05 – 4.94 (m, 3H), 4.84 (dd, *J* = 2.6, 1.0 Hz, 1H), 4.50 (s, 2H), 4.18 – 4.07 (m, 4H), 3.36 (d, *J* = 7.2 Hz, 2H), 2.74 (dt, *J* = 7.3, 1.3 Hz, 2H), 1.87 (s, 3H), 1.78 (s, 3H), 1.71 (s, 3H), 1.25 (t, *J* = 7.1 Hz, 3H). ^{13}C NMR (101 MHz, Chloroform-*d*) δ 172.40, 144.68, 140.61, 138.69, 136.21, 133.90, 131.62, 129.52, 128.69, 128.46, 127.91, 127.62, 126.90, 116.83, 115.20, 71.75, 71.25, 60.57, 38.25, 37.03, 23.26, 22.71, 19.89, 14.36. HRMS-ESI: Found $[M+Na]^+$ =417.2405; $C_{26}H_{34}O_3Na$ requires 417.2405.

Diethyl (2*E*,5*E*)-6-allyl-4-cyclobutylidene-5-(prop-1-en-2-yl)octa-2,5-dienedioate

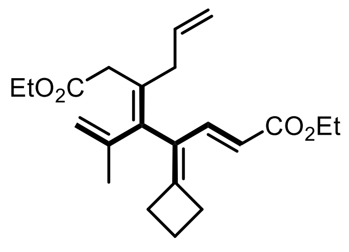

47

The reaction was carried out according to the general procedure ***GP4*** using eneallene **1** (39 mg, 0.2 mmol) and allene **S44** (50 mg, 0.3 mmol), and the desired product was obtained after purification by silica gel column chromatography (eluent: Et_2O/pentane (5: 95)) as a colorless oil (48 mg, 67% yield). ^{1}H NMR (400 MHz, Chloroform-*d*) δ 7.35 (d, *J* = 15.5 Hz, 1H), 5.79 (d, *J* = 15.6 Hz, 1H), 5.72 – 5.57 (m, 1H), 5.06 – 4.98 (m, 1H), 5.02 – 4.94 (m, 2H), 4.78 (dd, *J* = 2.4, 1.0 Hz, 1H),4.18 (q, *J* = 7.1 Hz, 2H), 4.13 (q, *J* = 7.1 Hz, 2H), 3.31 (s, 2H), 2.96 (td, *J* = 7.9, 2.6 Hz, 2H), 2.77 – 2.66 (m, 4H), 2.01 (p, *J* = 7.9 Hz, 2H), 1.69 (s, 3H), 1.28 (td, *J* = 7.1, 2.3 Hz, 6H). ^{13}C NMR (101 MHz, Chloroform-*d*) δ 171.97, 168.07, 155.59, 143.92, 140.91, 137.56, 135.94, 116.90, 116.85, 115.22, 60.70, 60.26, 38.13, 37.18, 31.88, 30.88, 22.35, 16.50, 14.46, 14.32. HRMS-ESI: Found $[M+Na]^+$ =381.2038; $C_{22}H_{30}O_4Na$ requires 381.2036.

Diethyl (2E,5*E*)-6-allyl-4-cycloheptylidene-5-(prop-1-en-2-yl)octa-2,5-dienedioate

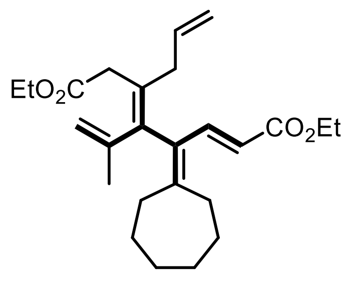

48

The reaction was carried out according to the general procedure ***GP4*** using eneallene **1** (39 mg, 0.2 mmol) and allene **S45** (62 mg, 0.3 mmol), and the desired product was obtained after purification by silica gel column chromatography (eluent: Et_2O/pentane (5: 95)) as a colorless oil (39 mg, 48% yield). ^{1}H NMR (400 MHz, Chloroform-*d*) δ 7.83 (d, *J* = 15.4 Hz, 1H), 5.88 (d, *J* = 15.4 Hz, 1H), 5.70 – 5.54 (m, 1H), 5.04 – 4.94 (m, 3H), 4.86 (dd, *J* = 2.4, 1.0 Hz, 1H), 4.24 – 4.10 (m, 4H), 3.43 (d, *J* = 15.9 Hz, 1H), 3.33 (d, *J* = 16.0 Hz, 1H), 2.71 (dq, *J* = 7.1, 1.4 Hz, 2H), 2.64 – 2.56 (m, 2H), 2.51 – 2.37 (m, 2H), 1.67 (s, 3H), 1.64 – 1.42 (m, 8H), 1.28 (td, *J* = 7.1, 4.1 Hz, 6H). ^{13}C NMR (101 MHz, Chloroform-*d*) δ 172.11, 168.29, 153.91, 144.13, 141.28, 139.45, 135.78, 131.13, 129.55, 118.77, 117.36, 115.83, 60.69, 60.26, 38.51, 37.18, 34.59, 31.48, 30.08, 28.43, 28.06, 26.92, 22.67, 14.48, 14.34. HRMS-ESI: Found $[M+Na]^+$ =423.2504; $C_{25}H_{36}O_4Na$ requires 423.2506.

Diethyl (2*E*,5*E*)-6-allyl-4-((*E*)-benzylidene)-5-(prop-1-en-2-yl)octa-2,5-dienedioate

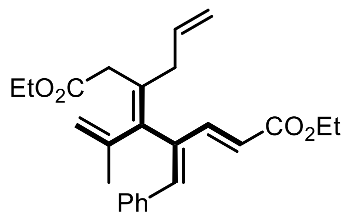

49

The reaction was carried out according to the general procedure ***GP4*** using eneallene **1** (39 mg, 0.2 mmol) and allene **S46** (60 mg, 0.3 mmol), and the desired product was obtained after purification by silica gel column chromatography (eluent: Et_2O/pentane (5: 95)) as a colorless oil (54 mg, 69% yield). ^{1}H NMR (400 MHz, Chloroform-*d*) δ 7.67 – 7.60 (m, 2H), 7.49 (d, *J* = 15.4 Hz, 1H), 7.36 – 7.25 (m, 3H), 6.86 (s, 1H), 6.01 (d, *J* = 15.4 Hz, 1H), 5.63 (ddt, *J* = 17.3, 10.1, 7.3 Hz, 1H), 5.05 – 4.94 (m, 3H), 4.95 – 4.89 (m, 1H), 4.26 – 4.18 (m, 4H), 3.47 (d, *J* = 1.5 Hz, 2H), 2.87 (d, *J* = 7.3 Hz, 2H), 1.64 (s, 3H), 1.32 (t, *J* = 7.1 Hz, 6H). ^{13}C NMR (101 MHz, Chloroform-*d*) δ 171.79, 167.56, 148.56, 142.05, 139.58, 137.25, 137.20, 135.85, 134.88, 131.35, 130.29, 128.87, 128.39, 119.88, 117.96, 117.14, 60.85, 60.50, 38.94, 37.34, 22.36, 14.46, 14.41. HRMS-ESI: Found $[M+Na]^+$ =417.2049; $C_{25}H_{30}O_4Na$ requires 417.2046.

Ethyl (2*E*,5*E*)-6-benzyl-5-(prop-1-en-2-yl)-4-(propan-2-ylidene)nona-2,5,8-trienoate

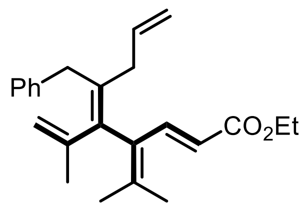

50

The reaction was carried out according to the general procedure ***GP4*** using eneallene **S3** (40 mg, 0.2 mmol) and allene **S35** (46 mg, 0.3 mmol), and the desired product was obtained after purification by silica gel column chromatography (eluent: Et_2O/pentane (5: 95)) as a colorless oil (64 mg, 91% yield). ^{1}H NMR (400 MHz, Chloroform-*d*) δ 7.81 (d, *J* = 15.4 Hz, 1H), 7.34 – 7.25 (m, 2H), 7.24 – 7.15 (m, 3H), 5.80 (d, *J* = 15.5 Hz, 1H), 5.62 (ddt, *J* = 17.0, 10.0, 7.0 Hz, 1H), 5.09 – 5.02 (m, 1H), 4.99 – 4.91 (m, 1H), 4.94 – 4.89 (m, 1H), 4.90 – 4.80 (m, 1H), 4.20 (q, *J* = 7.1 Hz, 2H), 3.74 (q, *J* = 14.7 Hz, 2H), 2.57 – 2.45 (m, 2H), 2.01 (s, 3H), 1.85 (s, 3H), 1.76 (s, 3H), 1.31 (t, *J* = 7.1 Hz, 3H). ^{13}C NMR (101 MHz, Chloroform-*d*) δ 168.16, 143.88, 143.76, 141.61, 140.65, 137.09, 136.23, 135.74, 132.13, 129.08, 128.48, 125.98, 118.62, 116.52, 115.79, 60.27, 37.12, 36.61, 24.09, 23.01, 20.60, 14.48. HRMS-ESI: Found $[M+Na]^+$ =373.2137; $C_{24}H_{30}O_2Na$ requires 373.2138.

Ethyl (2*E*,5*E*)-5-(prop-1-en-2-yl)-4-(propan-2-ylidene)-6-((trimethylsilyl)methyl)nona-2,5,8-trienoate

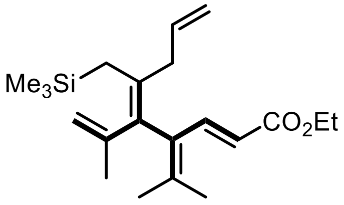

51

The reaction was carried out according to the general procedure ***GP4*** using eneallene **S4** (39 mg, 0.2 mmol) and allene **S35** (46 mg, 0.3 mmol), and the desired product was obtained after purification by silica gel column chromatography (eluent: Et_2O/pentane (5: 95)) as a colorless oil (65 mg, 94% yield). ^{1}H NMR (400 MHz, Chloroform-*d*) δ 7.79 (d, *J* = 15.3 Hz, 1H), 5.78 (d, *J* = 15.4 Hz, 1H), 5.71 – 5.56 (m, 1H), 5.04 – 4.88 (m, 3H), 4.81 (dd, *J* = 2.4, 0.9 Hz, 1H), 4.18 (qd, *J* = 7.1, 2.4 Hz, 2H), 2.64 – 2.51 (m, 2H), 1.98 (s, 3H), 1.94 (d, *J* = 1.7 Hz, 2H), 1.81 (s, 3H), 1.67 (s, 3H), 1.28 (t, *J* = 7.1 Hz, 3H). 0.08 (s, 9H). ^{13}C NMR (101 MHz, Chloroform-*d*) δ 168.28, 144.60, 143.49, 142.06, 136.92, 135.31, 133.10, 132.38, 118.58, 116.25, 115.38, 60.16, 39.88, 24.18, 22.98, 21.91, 20.56, 14.47, 0.10. HRMS-ESI: Found $[M+Na]^+$ =369.2221; $C_{21}H_{34}O_2SiNa$ requires 369.2220.

Ethyl (2*E*,5*E*)-6-(2-(benzyloxy)ethyl)-5-(prop-1-en-2-yl)-4-(propan-2-ylidene)nona-2,5,8-trienoate

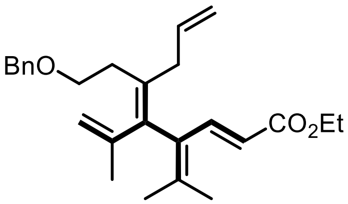

52

The reaction was carried out according to the general procedure ***GP4*** using eneallene **S5** (48 mg, 0.2 mmol) and allene **S35** (46 mg, 0.3 mmol), and the desired product was obtained after purification by silica gel column chromatography (eluent: Et_2O/pentane (5: 95)) as a colorless oil (72 mg, 92% yield). ^{1}H NMR (400 MHz, Chloroform-*d*) δ 7.78 (d, *J* = 15.4 Hz, 1H), 7.38 – 7.24 (m, 5H), 5.79 (d, *J* = 15.4 Hz, 1H), 5.64 (ddt, *J* = 17.1, 10.3, 7.0 Hz, 1H), 5.00 – 4.90 (m, 3H), 4.82 (d, *J* = 3.1 Hz, 1H), 4.52 (s, 2H), 4.18 (q, *J* = 7.1 Hz, 2H), 3.57 (t, *J* = 7.0 Hz, 2H), 2.74 – 2.65 (m, 2H), 2.63 (d, *J* = 7.0 Hz, 2H), 1.99 (s, 3H), 1.80 (s, 3H), 1.67 (s, 3H), 1.28 (t, *J* = 7.1 Hz, 3H). ^{13}C NMR (101 MHz, Chloroform-*d*) δ 168.28, 144.08, 143.62, 141.52, 138.68, 137.51, 136.47, 133.72, 132.36, 128.41, 127.78, 127.58, 118.55, 116.32, 115.47, 72.93, 68.86, 60.24, 37.46, 31.41, 24.06, 22.86, 20.48, 14.48. HRMS-ESI: Found $[M+Na]^+$ =417.2377; $C_{26}H_{34}O_3Na$ requires 417.2400.

Ethyl (2*E*,5*E*)-6-(2-(1,3-dioxoisoindolin-2-yl)ethyl)-5-(prop-1-en-2-yl)-4-(propan-2-ylidene)nona-2,5,8-trienoate

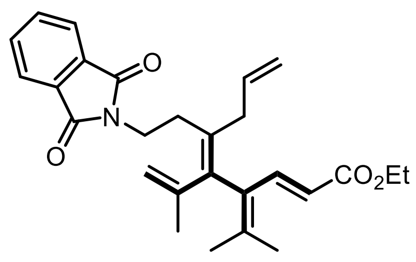

53

The reaction was carried out according to the general procedure ***GP4*** using eneallene **S7** (56 mg, 0.2 mmol) and allene **S35** (46 mg, 0.3 mmol), and the desired product was obtained after purification by silica gel column chromatography (eluent: Et_2O/pentane (5: 95)) as a colorless oil (79 mg, 92% yield). ^1H NMR (400 MHz, Chloroform-*d*) δ 7.81 (dd, *J* = 5.4, 3.1 Hz, 2H), 7.74 (d, *J* = 15.4 Hz, 1H), 7.68 (dd, *J* = 5.5, 3.0 Hz, 2H), 5.74 – 5.64 (m, 1H), 5.64 (d, *J* = 15.5 Hz, 1H), 5.14 – 4.96 (m, 3H), 4.77 (dd, *J* = 2.2, 1.0 Hz, 1H), 4.16 (q, *J* = 7.1 Hz, 2H), 3.87 – 3.72 (m, 2H), 2.81 – 2.71 (m, 3H), 2.69 – 2.60 (m, 1H), 1.96 (s, 3H), 1.72 (s, 3H), 1.62 (s, 3H), 1.27 (t, *J* = 7.1 Hz, 3H). ^{13}C NMR (101 MHz, Chloroform-*d*) δ 168.21, 167.99, 143.58, 143.49, 141.28, 138.48, 135.88, 133.96, 133.14, 132.27, 131.98, 123.22, 118.42, 117.06, 115.71, 60.22, 37.08, 36.87, 30.08, 24.01, 22.82, 20.47, 14.46. HRMS-ESI: Found $[M+Na]^+$ =456.2142; $C_{27}H_{31}NO_4Na$ requires 456.2145.

Ethyl (2*E*,5*E*)-6-phenyl-5-(prop-1-en-2-yl)-4-(propan-2-ylidene)nona-2,5,8-trienoate

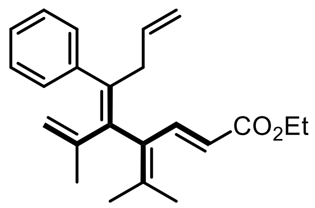

54

The reaction was carried out according to the general procedure ***GP4*** using eneallene **S10** (37 mg, 0.2 mmol) and allene **S35** (46 mg, 0.3 mmol), and the desired product was obtained after purification by silica gel column chromatography (eluent: Et_2O/pentane (5: 95)) as a colorless oil (49 mg, 73% yield). ^1H NMR (400 MHz, Chloroform-*d*) δ 7.87 (d, *J* = 15.4 Hz, 1H), 7.32 – 7.27 (m, 2H), 7.25 – 7.21 (m, 3H), 5.81 (d, *J* = 15.4 Hz, 1H), 5.55 (ddt, *J* = 17.0, 10.2, 7.0 Hz, 1H), 4.86 – 4.76 (m, 3H), 4.67 – 4.64 (m, 1H), 4.25 – 4.18 (m, 2H), 3.02 – 2.96 (m, 2H), 2.07 (s, 3H), 1.93 (s, 3H), 1.49 (s, 3H), 1.31 (t, *J* = 7.1 Hz, 3H). ^{13}C NMR (101 MHz, Chloroform-*d*) δ 168.19, 143.70, 143.32, 142.57, 141.66, 138.75, 137.20, 135.53, 133.66, 128.94, 128.01, 126.81, 118.25, 117.81, 116.15, 60.35, 41.14, 24.20, 23.18, 20.44, 14.52. HRMS-ESI: Found $[M+Na]^+$ =359.1988; $C_{23}H_{28}O_2Na$ requires 359.1989.

(3*E*,6*Z*)-7-Allyl-6-(prop-1-en-2-yl)-5-(propan-2-ylidene)dodeca-3,6,11-trien-2-one

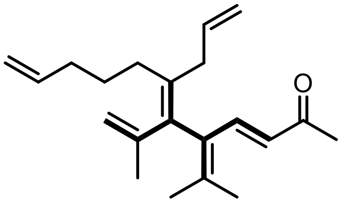

55

The reaction was carried out according to the general procedure ***GP4*** using eneallene **S9** (35 mg, 0.2 mmol) and allene **S36** (37 mg, 0.3 mmol), and the desired product was obtained after purification by silica gel column chromatography (eluent: Et_2O/pentane (5: 95)) as a colorless oil (51 mg, 85% yield). ^{1}H NMR (400 MHz, Chloroform-*d*) δ 7.63 (d, *J* = 15.6 Hz, 1H), 6.02 (d, *J* = 15.6 Hz, 1H), 5.81 (ddt, *J* = 16.9, 10.2, 6.7 Hz, 1H), 5.69 – 5.58 (m, 1H), 5.05 – 4.92 (m, 5H), 4.77 (dd, *J* = 2.4, 0.9 Hz, 1H), 2.65 – 2.58 (m, 2H), 2.34 – 2.28 (m, 2H), 2.26 (s, 3H), 2.09 – 2.02 (m, 2H), 2.01 (s, 3H), 1.83 (s, 3H), 1.67 (s, 3H), 1.58 – 1.47 (m, 2H). ^{13}C NMR (101 MHz, Chloroform-*d*) δ 199.24, 144.75, 144.10, 140.50, 138.77, 137.36, 136.44, 135.41, 132.83, 127.88, 116.19, 115.23, 114.72, 37.23, 34.20, 28.29, 27.83, 24.30, 22.95, 20.57. HRMS-ESI: Found $[M+Na]^+$ =321.2198; $C_{21}H_{30}ONa$ requires 321.2189.

Diethyl (2*E*,5*E*)-6-(3-methylbut-2-en-1-yl)-5-(prop-1-en-2-yl)-4-(propan-2-ylidene)octa-2,5-dienedioate

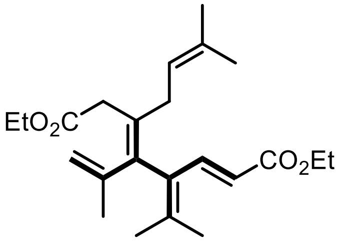

56

The reaction was carried out according to the general procedure ***GP4*** using eneallene **S17** (45 mg, 0.2 mmol) and allene **S35** (46 mg, 0.3 mmol), and the desired product was obtained after purification by silica gel column chromatography (eluent: Et_2O/pentane (5: 95)) as a colorless oil (61 mg, 82% yield). ^{1}H NMR (400 MHz, Chloroform-*d*) δ 7.77 (d, *J* = 15.4 Hz, 1H), 5.86 (d, *J* = 15.4 Hz, 1H), 4.98 (s, 1H), 4.96 – 4.91 (m, 1H), 4.85 (s, 1H), 4.23 – 4.10 (m, 4H), 3.32 (d, *J* = 7.5 Hz, 2H), 2.61 (t, *J* = 6.9 Hz, 2H), 1.99 (s, 3H), 1.85 (s, 3H), 1.65 (s, 3H), 1.65 (s, 3H), 1.53 (s, 3H), 1.28 (td, *J* = 7.1, 3.4 Hz, 6H). ^{13}C NMR (101 MHz, Chloroform-*d*) δ 172.27, 168.28, 144.09, 143.89, 141.27, 138.56, 133.38, 131.75, 131.17, 121.37, 118.67, 115.62, 60.66, 60.21, 37.40, 32.42, 25.93, 23.86, 22.53, 20.49, 17.75, 14.46, 14.34. HRMS-ESI: Found $[M+Na]^+$ =397.2357; $C_{23}H_{34}O_4Na$ requires 397.2357.

Ethyl (*S*,3*E*,6*E*)-3-allyl-11,15-dimethyl-8-oxo-4-(prop-1-en-2-yl)-5-(propan-2-ylidene) hexadeca-3,6,14-trienoate

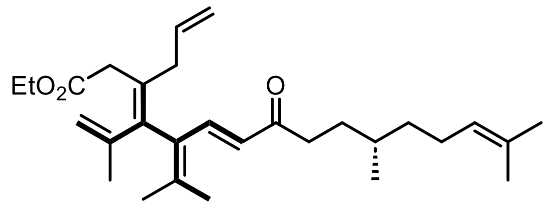

63

The reaction was carried out according to the general procedure ***GP4*** using eneallene **1** (39 mg, 0.2 mmol) and allene **S51** (75 mg, 0.3 mmol), and the desired product was obtained after purification by silica gel column chromatography (eluent: Et_2O/pentane (5: 95)) as a colorless oil (88 mg, 82% yield). ^{1}H NMR (400 MHz, Chloroform-*d*) δ 7.66 (d, *J* = 15.5 Hz, 1H), 6.20 (d, *J* = 15.5 Hz, 1H), 5.61 (ddt, *J* = 15.8, 11.2, 7.1 Hz, 1H), 5.11 – 5.04 (m, 1H), 5.02 – 4.93 (m, 3H), 4.87 – 4.84 (m, 1H), 4.13 (q, *J* = 7.1 Hz, 2H), 3.36 (s, 2H), 2.66 (d, *J* = 7.1 Hz, 2H), 2.59 – 2.44 (m, 2H), 2.01 (s, 3H), 1.98 – 1.89 (m, 2H), 1.86 (s, 3H), 1.66 (d, *J* = 1.0 Hz, 6H), 1.58 (s, 3H), 1.44 (s, 4H), 1.26 (t, *J* = 7.1 Hz, 3H), 1.20 – 1.10 (m, 1H), 0.88 (d, *J* = 6.3 Hz, 3H). ^{13}C NMR (101 MHz, Chloroform-*d*) δ 201.76, 172.10, 145.10, 143.99, 139.49, 138.68, 135.57, 131.84, 131.26, 129.77, 126.95, 126.93, 124.88, 117.12, 115.77, 60.64, 39.55, 38.27, 37.01, 32.36, 31.63, 25.82, 25.61, 24.07, 22.45, 20.58, 19.47, 19.45, 17.75, 14.37. HRMS-ESI: Found $[M+Na]^+$ =463.3183; $C_{29}H_{44}O_3Na$ requires 463.3183.

(6*S*,10*E*,13*Z*,17*R*)-2,6,17,21-Tetramethyl-14-(3-methylbut-2-en-1-yl)-13-(prop-1-en-2-yl)-12-(propan-2-ylidene)docosa-2,10,13,20-tetraen-9-one

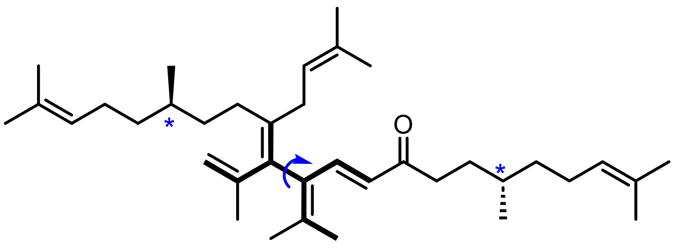

64

The reaction was carried out according to the general procedure ***GP4*** using eneallene **S23** (55 mg, 0.2 mmol) and allene **S51** (75 mg, 0.3 mmol), and the desired product was obtained after purification by silica gel column chromatography (eluent: Et_2O/pentane (5: 95)) as a colorless oil (80 mg, 77% yield). **[Note:** *atropisomerism was observed in [4]dendralene* ***64****, which results in two diastereomers present in NMR spectra***]** ^{1}H NMR (400 MHz, Chloroform-*d*) δ 7.67 (dd, *J* = 15.4, 2.8 Hz, 1H), 6.08 (d, *J* = 15.4 Hz, 1H), 5.14 – 5.03 (m, 2H), 4.96 (dt, *J* = 8.3, 2.0 Hz, 2H), 4.77 (d, *J* = 2.5 Hz, 1H), 2.60 – 2.43 (m, 4H), 2.34 – 2.19 (m, 2H), 1.99 (s, 3H), 2.05 – 1.88 (m, 4H), 1.81 (s, 3H), 1.69 – 1.66 (m, 9H), 1.64 (s, 3H), 1.59 (d, *J* = 5.3 Hz, 6H), 1.53 (s, 3H), 1.48 – 1.10 (m, 10H), 0.89 (dd, *J* = 6.0, 2.7 Hz, 6H). ^{13}C NMR (101

MHz, Chloroform-*d*) δ 201.81, 144.46, 144.37, 139.41, 138.99, 134.48, 133.13, 131.76, 131.29, 131.14, 126.64, 125.07, 124.87, 122.46, 114.84, 39.40, 39.34, 37.04, 36.28, 33.06, 33.00, 32.40, 31.81, 31.45, 29.05, 25.95, 25.83, 25.70, 25.63, 24.16, 23.01, 20.53, 19.69, 19.50, 17.89, 17.77. Distinguishing peaks for another atropisomer: ^{13}C NMR (101 MHz, Chloroform-*d*) δ 201.79, 144.39, 139.38, 138.98, 133.11, 126.59, 122.44, 114.80, 37.04, 36.30, 32.41, 31.48, 25.85, 25.71, 24.17, 23.03, 20.54, 19.50, 17.77. HRMS-ESI: Found $[M+Na]^+$ =543.4538; $C_{37}H_{60}ONa$ requires 543.4536.

2.5 Negative Data

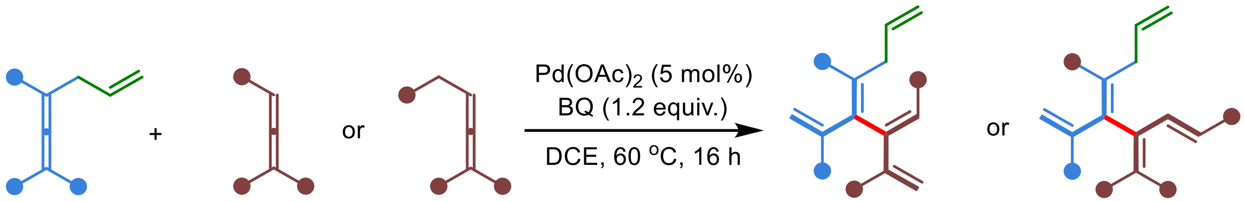

While most substrates matching the features indicated in the above scheme underwent the developed allene-allene cross-coupling smoothly, the following substrates failed to deliver the desired products.

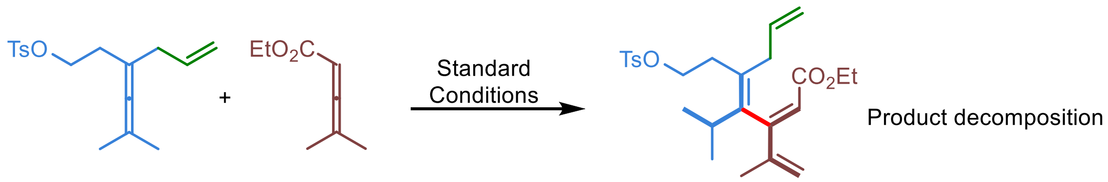

S-a1 **2**

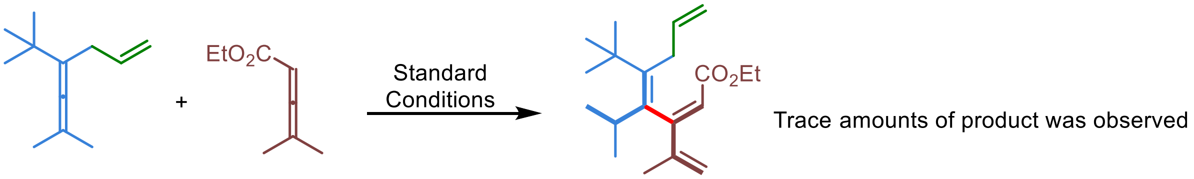

S-a2 **2**

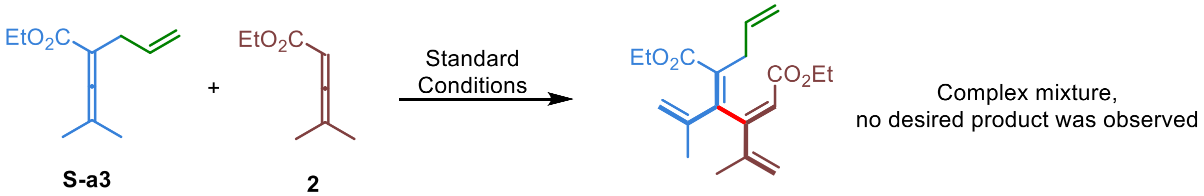

S-a3 **2**

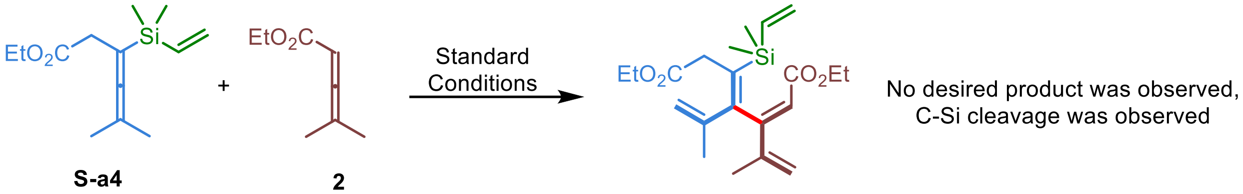

S-a4 **2**

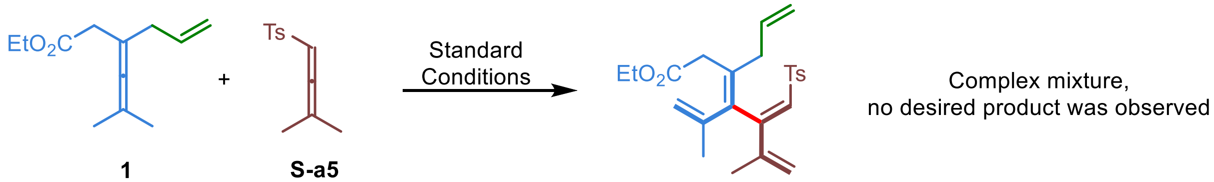

1 **S-a5**

A tosylate (Ts)-containing allene (**S-a1**) was tested with the model substrate **2**, a significant amount of a suspected product was observed on TLC; however, the target spot was unstable during

purification using silica gel column chromatography. A substrate (**S-a2**) bearing a *tert*-butyl group was also tested with the model substrate **2**, but only trace amounts of the desired product were observed, likely due to the steric hindrance caused by the *tert*-butyl group. The substrate (**S-a3**), which bears an ester group directly connected to the allene moiety, was unsuccessful in the desired cross-coupling reaction. Both chelating and electronic effects are possible factors contributing to this incompatibility. A fully-substituted alkenylsilane (**S-a4**) was tested as a potential directing group, but no formation of the desired product was detected. Instead, cleavage of the C–Si bond was observed in the ^{1}H NMR spectrum of the isolated reaction mixture. Finally, a tosyl-containing, directing-group-free allene (**S-a5**) was found to be incompatible, likely due to the strong chelating effect of the tosyl group to the metal center of the catalyst.

3. Gram-scale Synthesis and Products Transformations

3.1 Gram-scale Synthesis

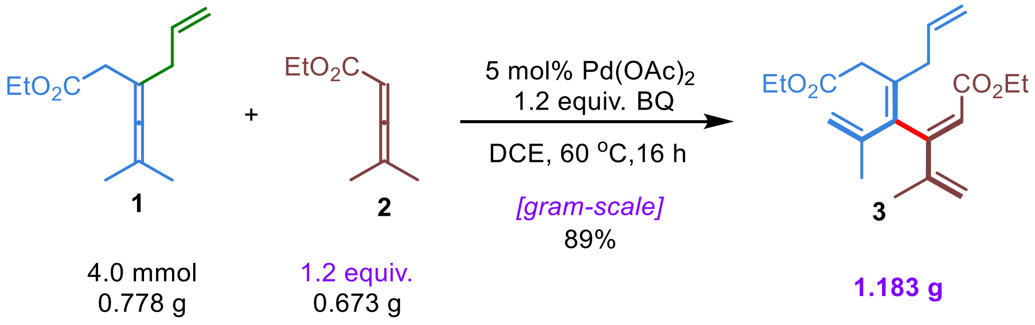

Scheme S7. Gram-scale Synthesis.

To a solution of enallene **1** (0.778 g, 4.0 mmol, 1.0 equiv.) and directing-group-free allene **2** (0.673g, 4.8 mmol, 1.2 equiv.) in 20 mL of DCE was added $Pd(OAc)_2$ (44 mg, 0.2 mmol, 0.05 equiv.) and BQ (benzoquinone) (520 mg, 4.8 mmol, 1.2 equiv.) in one portion. The resulting mixture was stirred at 60 °C for 16 h before concentrating under reduced pressure. The residue was purified using column chromatography on silica gel to afford the [4]dendralene **3** (1.183 g, 89%).

3.2 Products Transformations

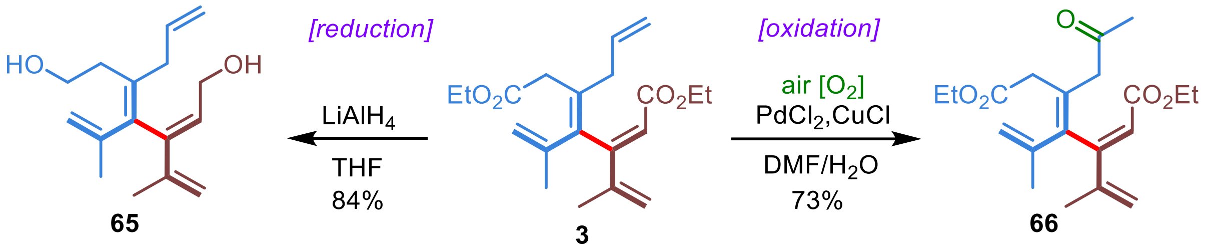

Scheme S8. Double Reduction and Regioselective Wacker-Tsuji Oxidation of [4]dendralene **3**.

Double reduction: To a solution of diester [4]dendralene **3** (67 mg, 0.2 mmol, 1.0 equiv.) in 2 mL THF was added $LiAlH_4$ (15 mg, 0.4 mmol, 2.0 equiv.) at 0 °C. The resulting mixture was then warmed to ambient temperature and stirred for 4 h before $Na_2SO_4{\cdot}10H_2O$ was added. After the addition the reaction mixture was further stirred for 30 min. The whole solution was then loaded directly on silica gel for column chromatography (eluent: EtOAc/pentane (50: 50)) to afford the diol **65** as a colorless oil (42 mg, 84%).

(2*Z*,4*E*)-5-Allyl-3,4-di(prop-1-en-2-yl)hepta-2,4-diene-1,7-diol

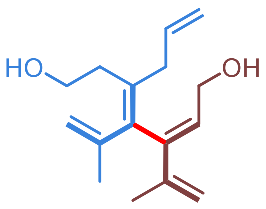

65

^{1}H NMR (400 MHz, Chloroform-*d*) δ 5.83 (t, *J* = 6.8 Hz, 1H), 5.79 – 5.63 (m, 1H), 5.09 (s, 1H), 5.07 – 4.97 (m, 4H), 4.84 (s, 1H), 4.17 (t, *J* = 6.5 Hz, 2H), 3.73 (td, *J* = 6.9, 1.3 Hz, 2H), 2.72 (d, *J* = 6.8 Hz, 2H), 2.61 (d, *J* = 7.7 Hz, 2H), 1.93 (s, 3H), 1.73 (s, 3H), 1.65 (br, 2H). ^{13}C NMR (101 MHz, Chloroform-*d*) δ 144.82, 142.98, 141.42, 137.94, 136.93, 132.70, 127.12, 116.60, 116.30, 115.35, 61.39, 61.05, 37.54, 34.36, 23.12, 20.55. HRMS-ESI: Found $[M+Na]^+$ =271.1670; $C_{16}H_{24}O_2Na$ requires 271.1669.

Wacker-Tsuji Oxidation:[25] A 4.0 mL vial with magnetic stir bar was charged successively with $PdCl_2$ (5.4 mg, 0.03 mmol, 0.015 equiv.), CuCl (14.9 mg, 0.3 mmol, 0.15 equiv.), DMF/H2O (7:1, 1.0 mL), [4]dendralene **3** (67 mg, 0.2 mmol, 1.0 equiv.). The resulting mixture was stirred at room temperature under air atmosphere overnight. The reaction mixture was then diluted with 5 mL H_2O and extracted with Et_2O (10 mL x 3). The combined organic layer was dried over Na_2SO_4, filtered, and concentrated under reduced pressure. The residue was purified using column chromatography on silica gel to afford **66** as a colorless oil (51 mg, 73%).

Diethyl (2*Z*,4*E*)-5-(2-oxopropyl)-3,4-di(prop-1-en-2-yl)hepta-2,4-dienedioate

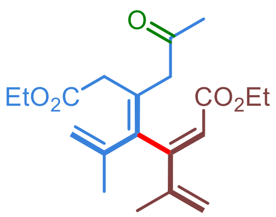

66

^{1}H NMR (400 MHz, Chloroform-*d*) δ 5.98 (s, 1H), 5.60 (s, 1H), 5.34 (s, 1H), 5.05 (t, *J* = 1.8 Hz, 1H), 4.96 (d, *J* = 2.2 Hz, 1H), 4.17 – 4.06 (m, 4H), 3.60 (s, 1H), 3.38 (s, 1H), 3.17 (d, *J* = 18.5 Hz, 2H), 2.06 (s, 3H), 1.95 (s, 3H), 1.71 (s, 3H), 1.27 – 1.22 (m, 6H). ^{13}C NMR (101 MHz, Chloroform-*d*) δ 206.65, 172.09, 165.98, 153.72, 142.87, 140.51, 140.35, 124.78, 123.32, 118.14, 117.33, 60.69, 60.37, 47.83, 37.92, 30.47, 22.57, 20.50, 14.32. HRMS-ESI: Found [M+Na]$^+$ =371.1830; $C_{20}H_{28}O_5Na$ requires 371.1829.

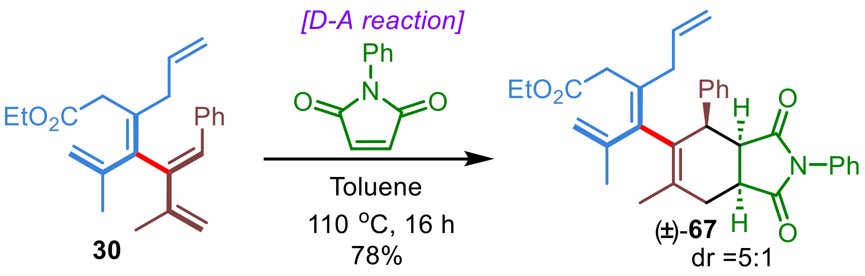

Scheme S9. Diels-Alder Reaction of [4]dendralene **30** with N-Phenylmaleimide.

Diels-Alder Reaction: A solution of [4]dendralene **30** (67 mg, 0.2 mmol, 1.0 equiv.) and N-Phenylmaleimide (70 mg, 0.4 mmol, 2.0 equiv.) in 1 mL toluene was stirred at 110 °C for 16 h. The reaction mixture was cooled to room temperature and then loaded directly on silica gel for column chromatography (eluent: EtOAc/pentane (25: 75)) to afford the adduct **67** as a pale yellow oil (80 mg, 78%).

Ethyl (*E*)-3-allyl-5-methyl-4-(6-methyl-1,3-dioxo-2,4-diphenyl-2,3,3a,4,7,7a-hexahydro-1H-isoindol-5-yl)hexa-3,5-dienoate

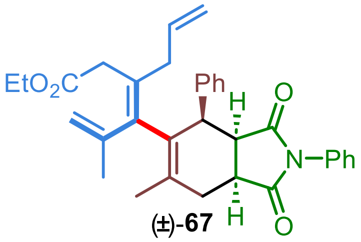

(±)-**67**
dr =5:1

Major isomer: ^{1}H NMR (400 MHz, Chloroform-*d*) δ 7.42 – 7.12 (m, 10H), 5.16 – 5.00 (m, 2H), 4.90 – 4.82 (m, 1H), 4.81 – 4.45 (m, 2H), 4.19 (s, 1H), 4.03 (q, *J* = 7.1 Hz, 2H), 3.53 – 3.39 (m, 2H), 3.24 (d, *J* = 16.1 Hz, 1H), 2.99 (d, *J* = 16.2 Hz, 1H), 2.74 – 2.67 (m, 2H), 2.39 – 2.21 (m, 2H), 1.70 (s, 3H), 1.52 (s, 3H), 1.16 (t, *J* = 7.1 Hz, 3H). ^{13}C NMR (101 MHz, Chloroform-*d*) δ 179.25, 177.84, 172.02, 142.88, 142.39, 141.32, 135.56, 132.35, 132.26, 131.05, 129.17, 128.73, 128.54, 128.26,

127.76, 126.71, 126.16, 117.33, 116.78, 60.52, 45.47, 43.08, 39.47, 37.68, 36.79, 30.10, 22.15, 21.79, 14.34. HRMS-ESI: Found $[M+Na]^+$ =532.2458; $C_{33}H_{35}NO_4Na$ requires 532.2458.

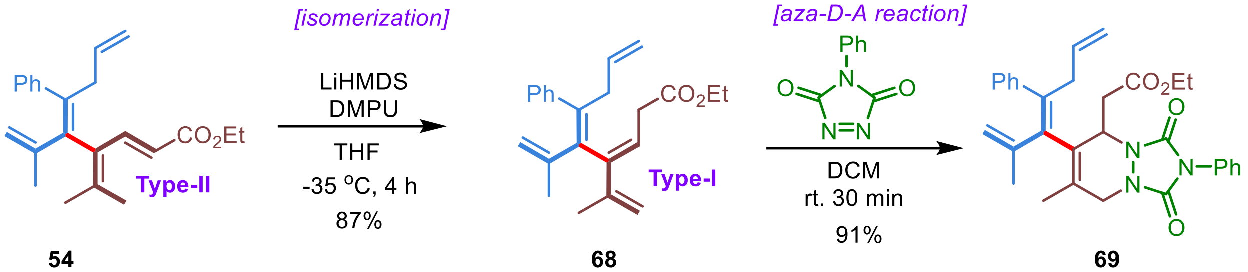

Scheme S10. Isomerization of **Type-II** [4]Dendralene **54** to **Type-I** [4]Dendralene **68** and Aza-Diels-Alder Reaction.

Isomerization:[26] To a solution of [4]dendralene **54** (68 mg, 0.2 mmol, 1.0 equiv.) in 1 mL dry THF was added 0.3 mL of LiHMDS solution (1.0 M in THF, 0.3 mmol, 1.5 equiv.) and DMPU (39 mg, 0.3 mmol, 1.5 equiv.) at −35 °C. The resulting mixture was stirred for 4 h at that temperature. For work up, the dark orange mixture was poured into aq. HOAc solution (10 % w/w, 5 mL). The aqueous phase was extracted with Et_2O (10 mL x 3), the combined extracts were washed with water (10 mL), dried over Na_2SO_4, filtered and concentrated under reduced pressure. The residue was purified using column chromatography on silica gel to afford [4]dendralene **68** as a colorless oil (59 mg, 87%).

Ethyl (3*Z*,5*E*)-6-phenyl-4,5-di(prop-1-en-2-yl)nona-3,5,8-trienoate

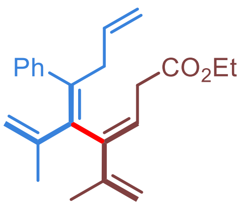

68

^1H NMR (400 MHz, Chloroform-*d*) δ 7.34 – 7.20 (m, 5H), 5.98 (t, *J* = 7.0 Hz, 1H), 5.63 – 5.48 (m, 1H), 5.15 (s, 1H), 5.06 (s, 1H), 4.88 – 4.69 (m, 4H), 4.17 (q, *J* = 7.1 Hz, 2H), 3.25 (d, *J* = 7.0 Hz, 2H), 3.04 (d, *J* = 6.7 Hz, 2H), 2.02 (s, 3H), 1.49 (s, 3H), 1.28 (t, *J* = 7.1 Hz, 3H). ^{13}C NMR (101 MHz, Chloroform-*d*) δ 172.09, 144.52, 143.67, 142.43, 141.93, 138.47, 136.96, 135.66, 128.95, 127.99, 126.79, 120.37, 117.76, 116.14, 115.60, 60.75, 40.97, 35.57, 23.14, 20.60, 14.37. HRMS-ESI: Found $[M+Na]^+$ =358.1981; $C_{23}H_{28}O_2Na$ requires 358.1982.

Aza-Diels-Alder Reaction: To a solution of the obtained [4]dendralene **68** (51 mg, 0.15 mmol, 1.0 equiv.) in 1 mL CH_2Cl_2 was added PTAD (4-Phenyl-1,2,4-triazoline-3,5-dione) (32 mg, 0.18 mmol, 1.2 equiv.) at room temperature. The resulting mixture was stirred at room temperature for 30 min. The reaction mixture was then loaded directly on silica gel for column chromatography (eluent: EtOAc/pentane (20: 80)) to afford the adduct **69** as a pale yellow oil (70 mg, 91%).

Ethyl (*E*)-2-(7-methyl-6-(2-methyl-4-phenylhepta-1,3,6-trien-3-yl)-1,3-dioxo-2-phenyl-2,3,5,8-tetrahydro-1*H*-[1,2,4]triazolo[1,2-a]pyridazin-5-yl)acetate

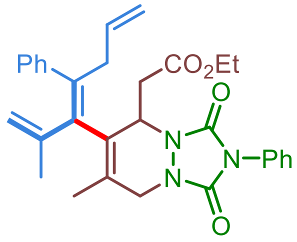

69

^{1}H NMR (400 MHz, Chloroform-*d*) δ 7.58 – 7.51 (m, 2H), 7.48 (t, *J* = 7.7 Hz, 2H), 7.41 – 7.34 (m, 1H), 7.33 – 7.16 (m, 5H), 5.64 – 5.49 (m, 1H), 4.98 – 4.85 (m, 4H), 4.71 (s, 1H), 4.35 (d, *J* = 16.3 Hz, 1H), 4.15 – 3.97 (m, 3H), 3.26 (d, *J* = 6.7 Hz, 2H), 2.89 (dd, *J* = 13.8, 4.5 Hz, 1H), 2.63 (dd, *J* = 13.8, 6.1 Hz, 1H), 1.94 (s, 3H), 1.54 (s, 3H), 1.19 (t, *J* = 7.1 Hz, 3H). ^{13}C NMR (101 MHz, Chloroform-*d*) δ 170.17, 153.41, 151.40, 142.97, 141.29, 141.21, 135.43, 134.58, 131.43, 129.58, 129.27, 128.73, 128.28, 128.03, 127.10, 126.16, 125.67, 118.90, 117.09, 61.31, 51.95, 47.87, 40.18, 35.84, 22.84, 18.31, 14.13. HRMS-ESI: Found $[M+Na]^+$ =534.2363; $C_{31}H_{33}N_3O_4Na$ requires 534.2363.

4. *Kinetic Isotope Effect (KIE) Studies*

4.1 KIE Determined from Two Parallel Reactions

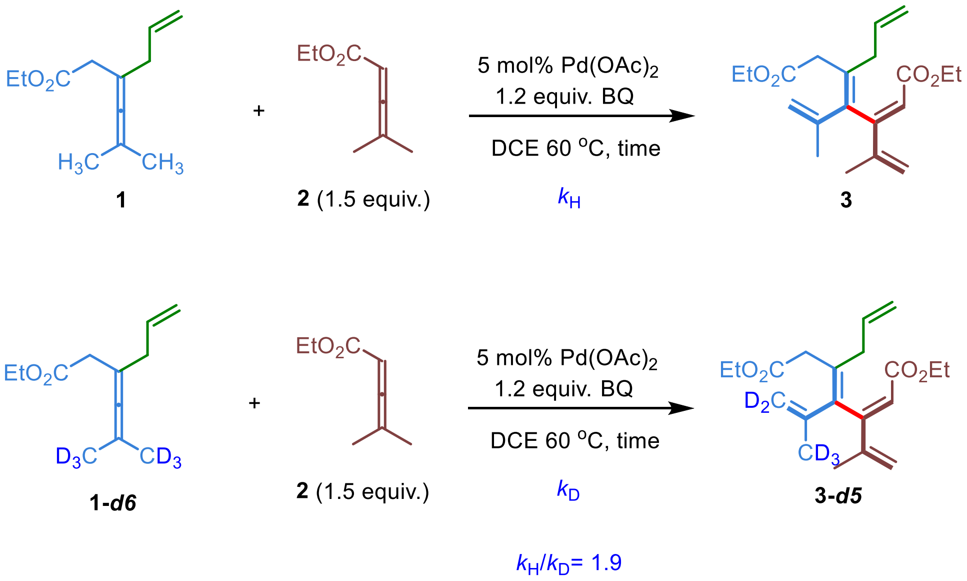

Scheme S11. KIE Determined from Two Parallel Reactions for the Formation of **Type-I** [4]Dendralene.

To a solution of enallene **1** (194 mg, 1.0 mmol, 1.0 equiv.) or **1-*d6*** (200 mg, 1.0 mmol, 1.0 equiv.), directing-group-free allene **2** (210 mg, 1.5 mmol, 1.5 equiv.) and 1,3,5-trimethoxybenzene (56 mg, 0.33 mmol, internal standard for NMR yield) in 10 mL of DCE was added $Pd(OAc)_2$ (11 mg, 0.05 mmol, 0.05 equiv.) and BQ (benzoquinone) (130 mg, 1.2 mmol, 1.2 equiv.) in one portion. The resulting mixture was stirred at 60 °C, and 0.5 mL of the reaction sample was collected via syringe at different reaction time. The reaction sample was passed through a pad of silica gel in a short glass pipette and washed with 5 mL Et_2O, and then the eluent was concentrated under reduced pressure. The yields of **3** and **3-*d5*** were determined by ^{1}H NMR of the residue. The reaction rates were obtained by plotting the product formation curve over time. The KIE determined from two parallel reactions is determined as KIE=k_H/k_D=1.9.

The detailed kinetic data and reaction progress curves as following:

Time/min	0	10	20	30	40	50	60	70	80	100	120
Yield of **3**/%	0	0	1.5	3.2	5.2	7.7	10.5	13.1	16.0	23.2	29.4
Yield of **3-d5**/%	0	0	0.7	1.2	3.4	4.8	6.9	8.2	9.9	12.6	17.9

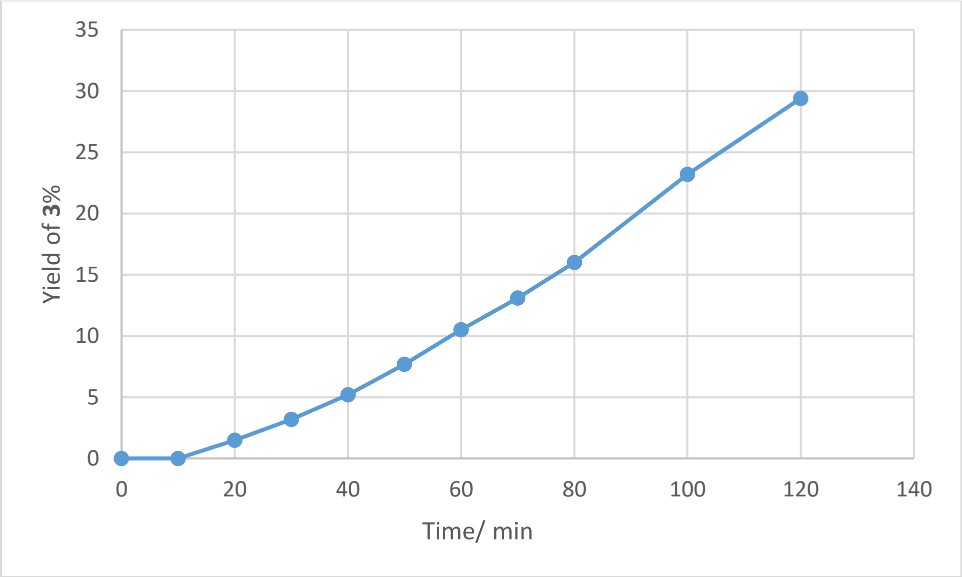

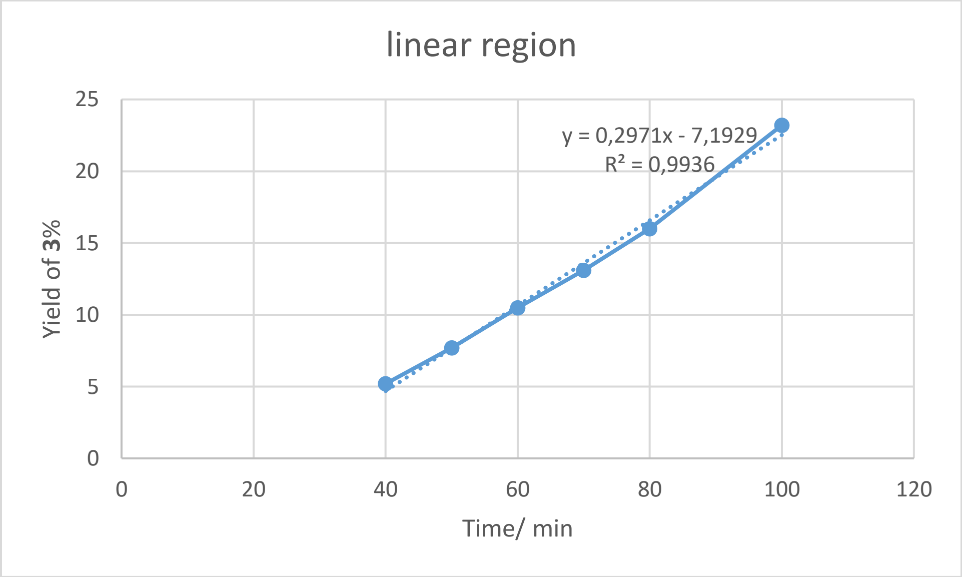

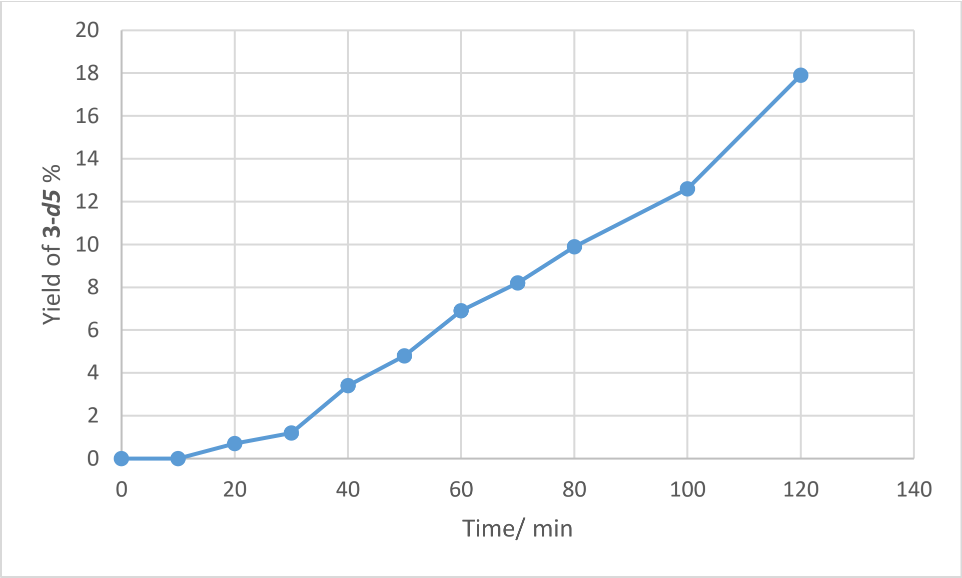

Yield of 3-d5 %
Time/ min

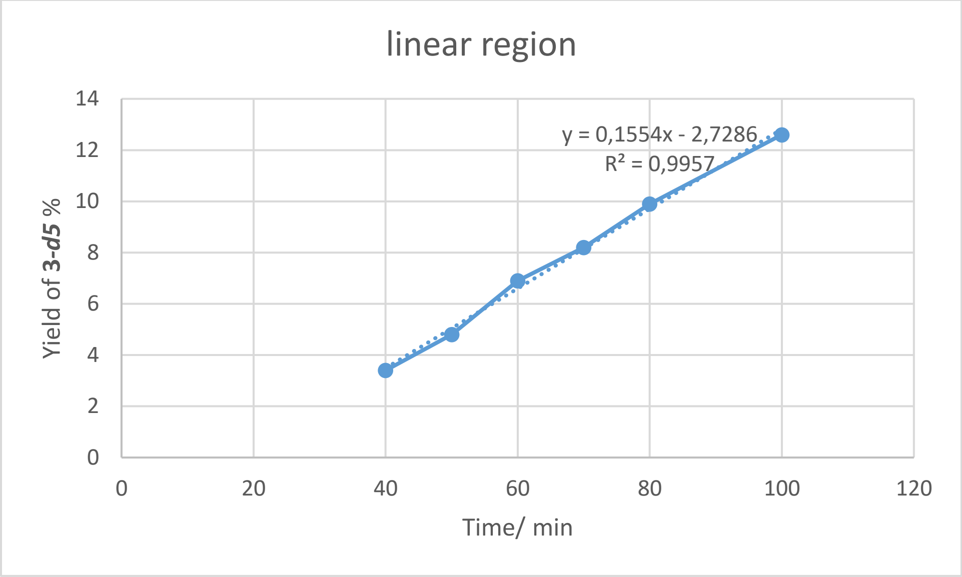

linear region
y = 0,1554x - 2,7286
R² = 0,9957
Yield of 3-d5 %
Time/ min

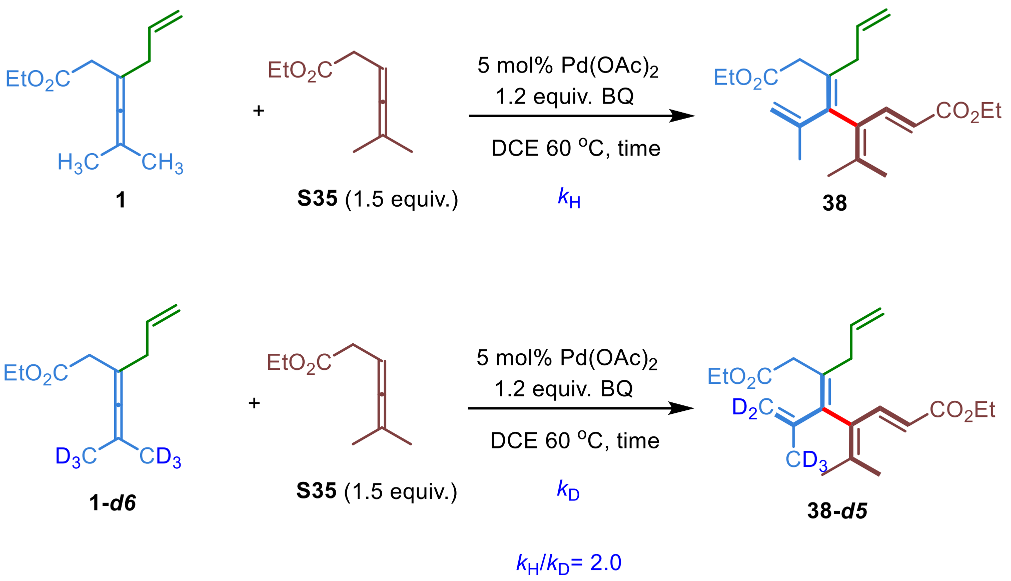

Scheme S12. KIE Determined from Two Parallel Reactions for the Formation of **Type-II** [4]Dendralene.

To a solution of enallene **1** (194 mg, 1.0 mmol, 1.0 equiv.) or **1-*d6*** (200 mg, 1.0 mmol, 1.0 equiv.), directing-group-free allene **S35** (231 mg, 1.5 mmol, 1.5 equiv.) and 1,3,5-trimethoxybenzene (56 mg, 0.33 mmol, internal standard for NMR yield) in 10 mL of DCE was added $Pd(OAc)_2$ (11 mg, 0.05 mmol, 0.05 equiv.) and BQ (benzoquinone) (130 mg, 1.2 mmol, 1.2 equiv.) in one portion. The resulting mixture was stirred at 60 °C, and 0.5 mL of the reaction sample was collected via syringe at different reaction time. The reaction sample was passed through a pad of silica gel in a short glass pipette and washed with 5 mL Et_2O, and then the eluent was concentrated under reduced pressure. The yields of **38** and **38-*d5*** were determined by ^{1}H NMR of the residue. The reaction rates were obtained by plotting the product formation curve over time. The KIE determined from two parallel reactions is determined as KIE=k_H/k_D=2.0

The detailed kinetic data and reaction progress curves as following:

Time/min	0	10	20	30	40	50	60	70	80	100	120
Yield of **38**/%	0	2.9	5.0	9.5	12.2	17.1	22.2	27.5	32.7	45.0	59.9
Yield of **38-*d5***/%	0	1.1	1.9	5.1	7.9	10.8	13.4	15.7	18.4	24.7	35.8

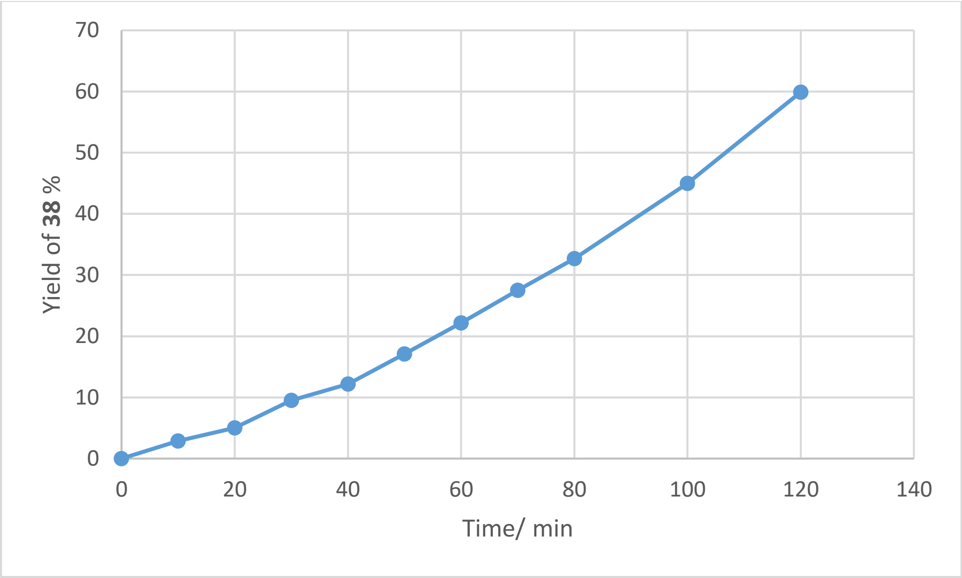

Yield of 38 %
Time/ min
0
10
20
30
40
50
60
70
0
20
40
60
80
100
120
140

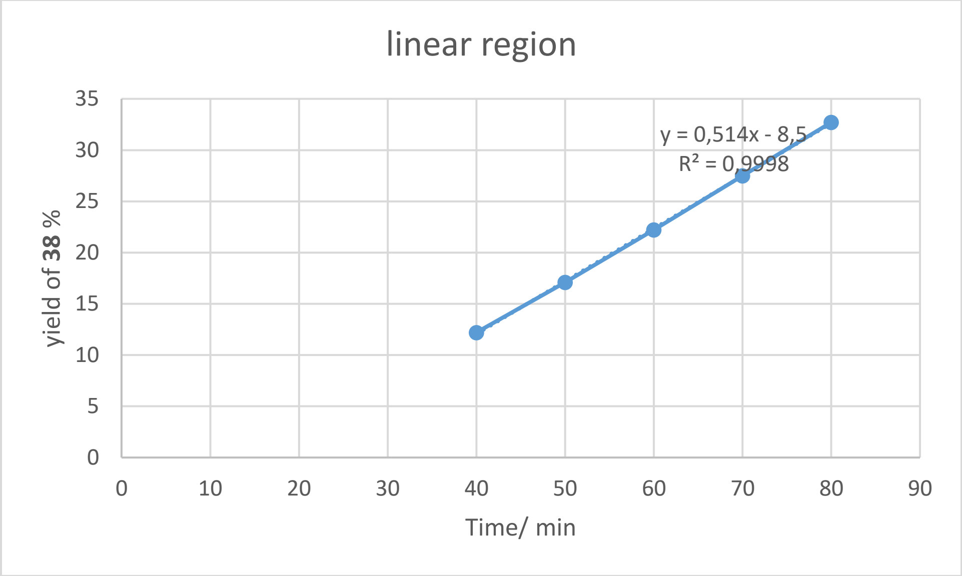

linear region
y = 0,514x - 8,5
R² = 0,9998
yield of 38 %
Time/ min
0
5
10
15
20
25
30
35
0
10
20
30
40
50
60
70
80
90

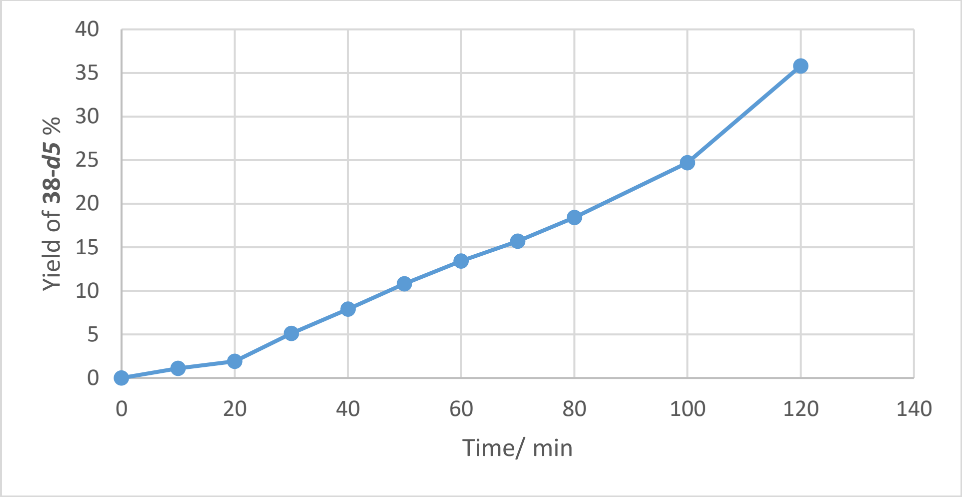

Yield of **38-*d5*** %
Time/ min

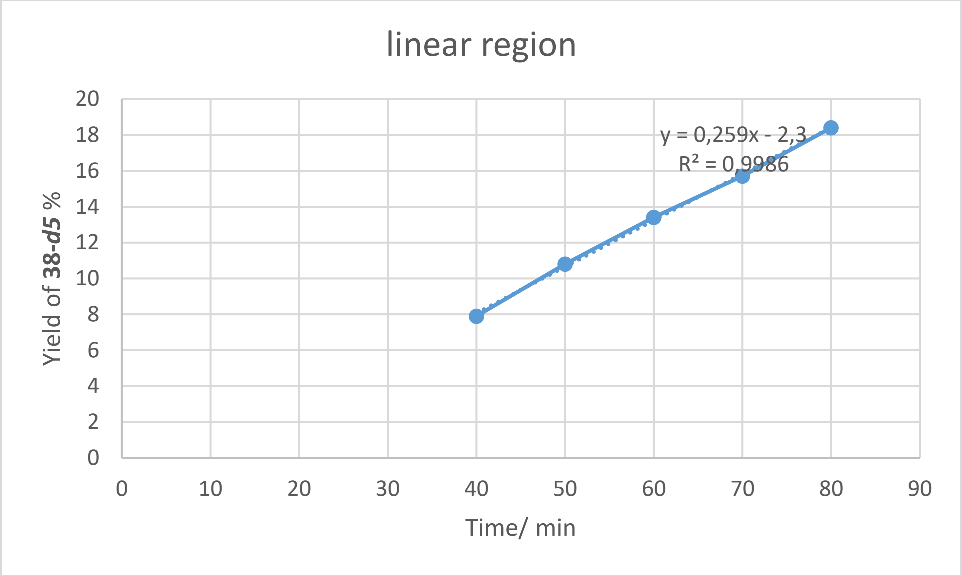

linear region
y = 0,259x - 2,3
R² = 0,9986
Yield of **38-*d5*** %
Time/ min

4.2 Intermolecular Competition KIE Experiments

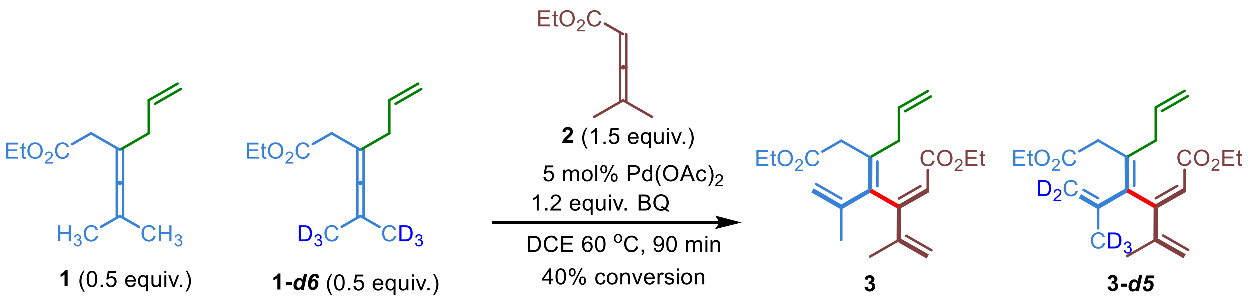

1:**1-*d6*** = 1:1

3:**3-*d5*** = 4.4:1

Scheme S13. Kinetic isotope effect (KIE) experiment for the formation of **Type-I** [4]dendralene.

To a solution of enallene **1** (19.4 mg, 0.1 mmol, 0.5 equiv.), enallene **1-*d6*** (20.0 mg, 0.1 mmol, 0.5 equiv.) and directing-group-free allene **2** (42 mg, 0.3 mmol, 1.5 equiv.) in 2 mL of DCE was added $Pd(OAc)_2$ (2.2 mg, 0.01 mmol, 0.05 equiv.) and BQ (benzoquinone) (26 mg, 0.24 mmol, 1.2 equiv.) in one portion. The resulting mixture was stirred at 60 °C for 90 minutes before

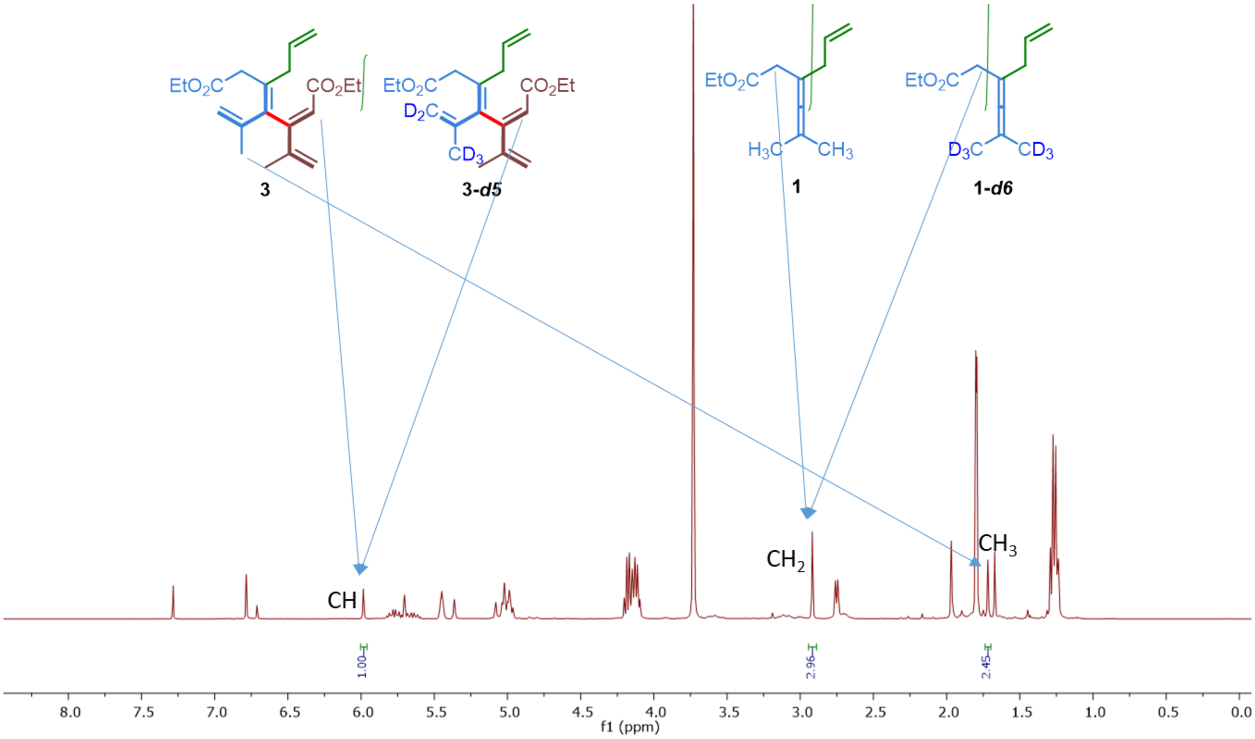

Figure S3. ^{1}H NMR Spectrum of KIE Experiment for the Formation of **Type-I** [4]Dendralene.

concentrating under reduced pressure. The conversion and the ratio of **3** and **3-*d5*** were determined by ^{1}H NMR of the residue.

As shown in **Figure S3**, the ratio of starting material (**1** and **1-*d6***) and product (**3** and **3-*d5***) was calculated to be 1.48 : 1 ((2.96/2) : 1), thus, the corresponding conversion was 40%; the ratio of **3** and **3-*d5*** was calculated to be 4.4 : 1 (2.45/(3-2.45)). Therefore, the kinetic isotope effect value was determined to be *KIE* = 4.7 according to Sih's equation.[27]

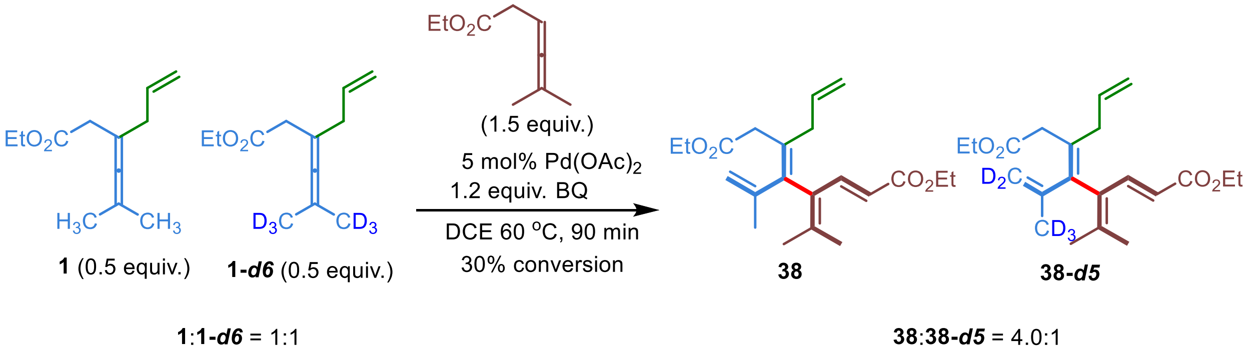

Scheme S14. Kinetic Isotope Effect (KIE) experiment for the Formation of **Type-II** [4]Dendralene.

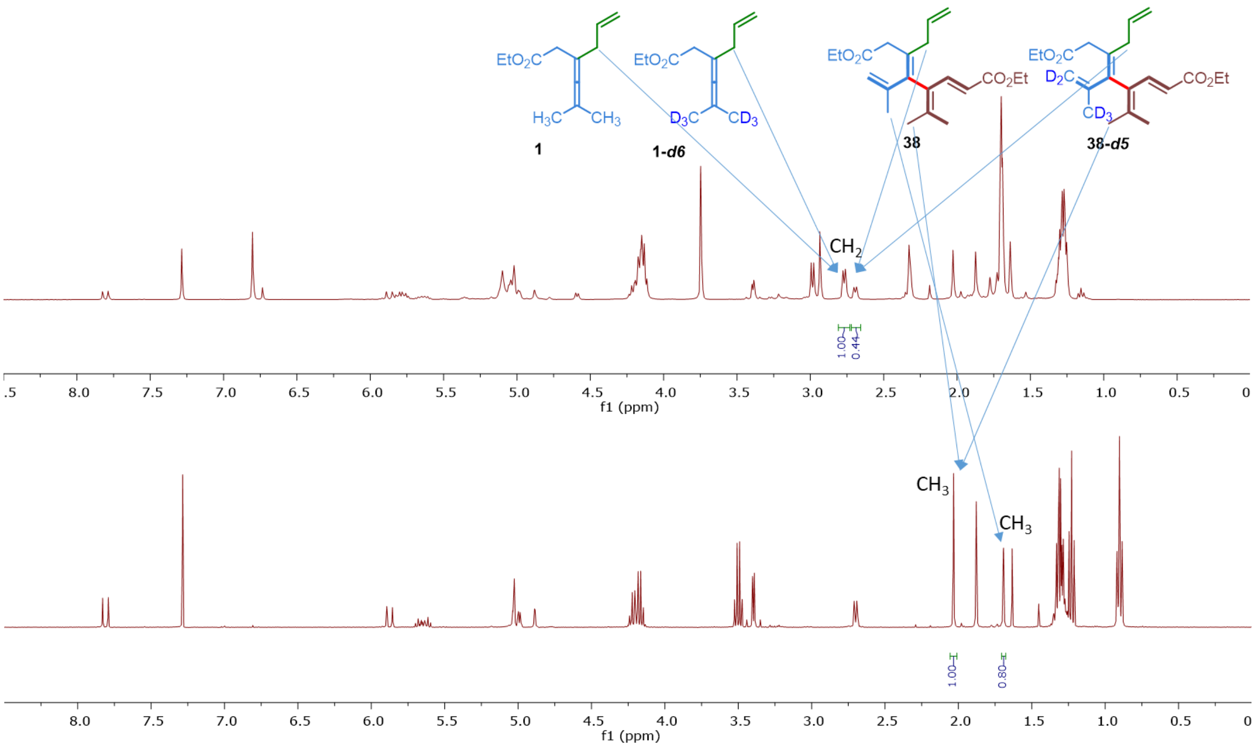

Figure S4. ^{1}H NMR Spectrum of KIE Experiment for the Formation of **Type-II** [4]Dendralene.

Similar experiment was carried out for the formation of **Type-II** [4]dendralene **38** (**Scheme S14**). The conversion was determined by crude ^{1}H NMR of the reaction (**Figure S4**, upper part, conversion = 0.44/(1+0.44) = 30%). However, the ratio of **38** and **38-*d5*** could not be calculated directly from the crude spectrum due to the overlap of the target peak. Isolation of product (**38** and **38-*d5***) by column chromatography was therefore performed, and the corresponding ratio of **38** and **38-*d5*** was calculated to be 4 : 1 (**Figure S4**, bottom, **38**:**38-*d5*** = 0.8 : (1-0.8)=4:1). Therefore, the kinetic isotope effect value in this case was determined to be *KIE* = 5.1 according to Sih's equation.

5. *Determination of Stereochemistry of Compounds 3, 30, 38, 49, and 67*

Confirmation of Stereochemistry of [4]Dendralene 3 (Type-I):

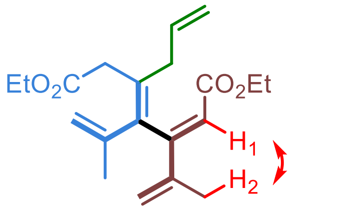

3

significant NOESY correlation

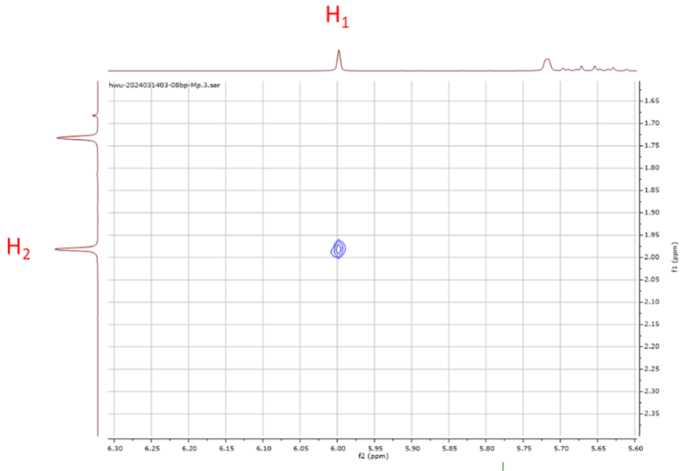

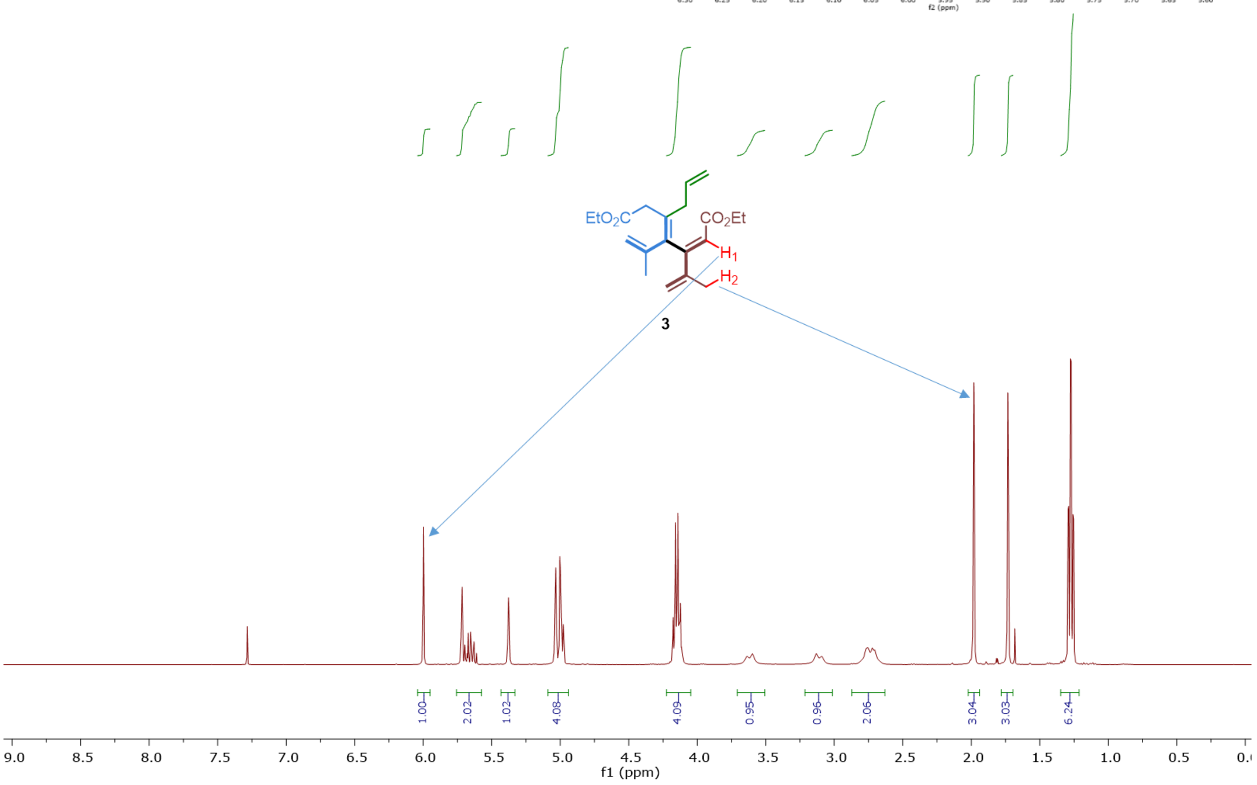

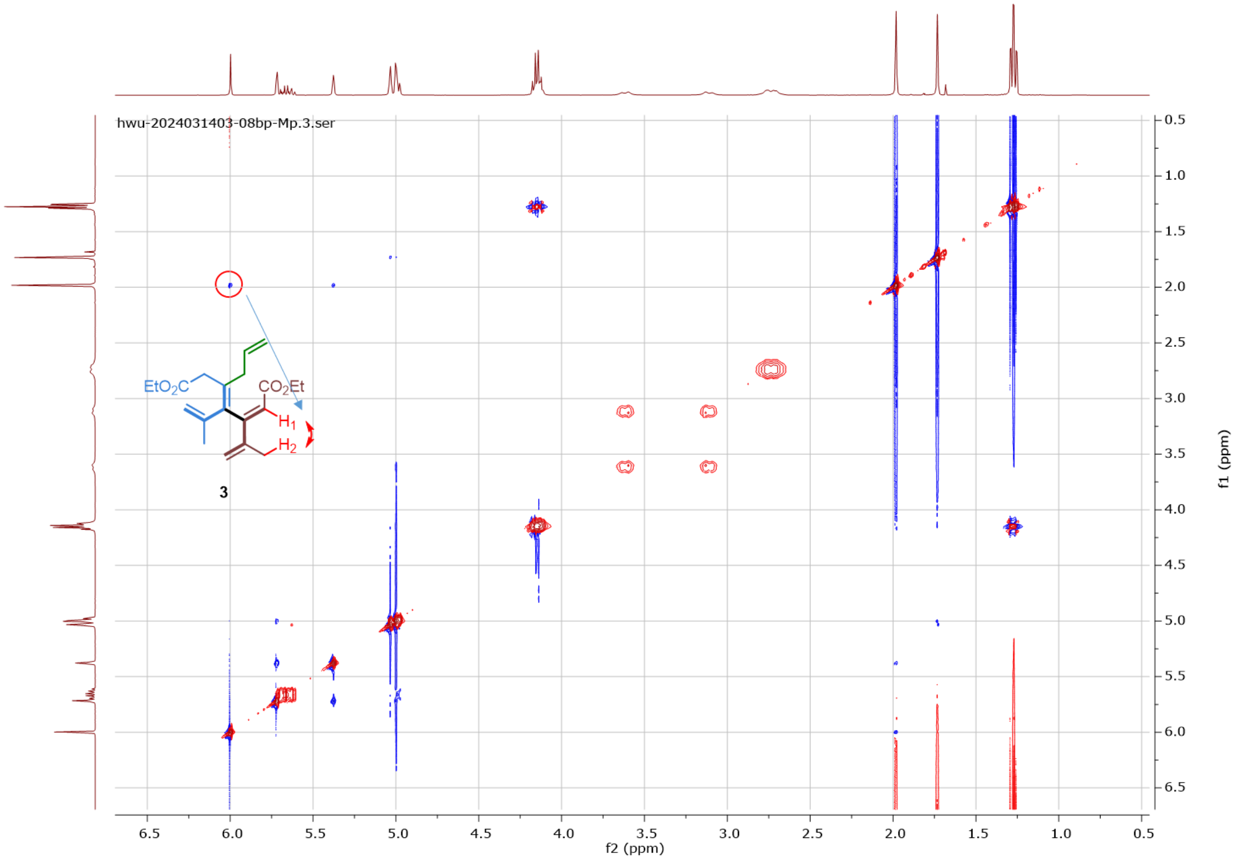

Confirmation of Stereochemistry of [4]Dendralene 30 (Type-I):

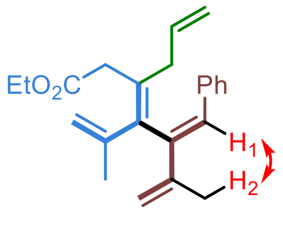

30

significant NOESY correlation

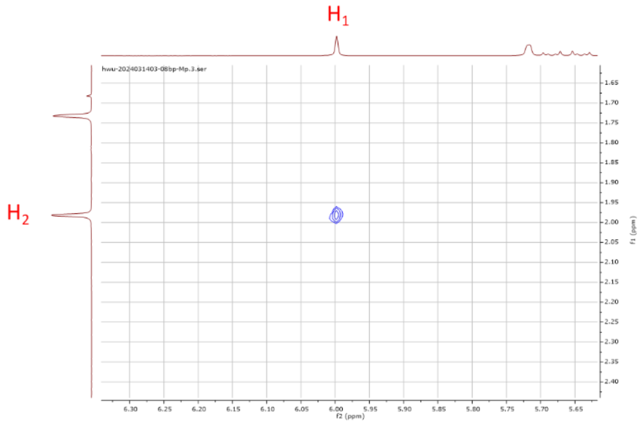

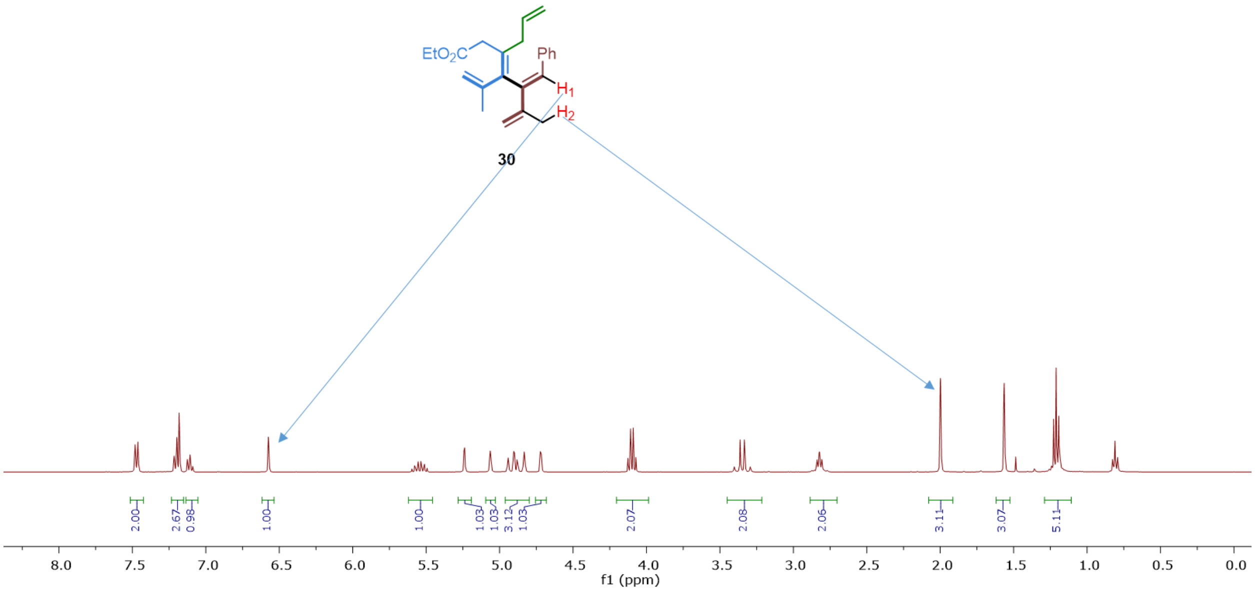
EtO2C
Ph
H1
H2
30
2.00
2.67
0.98
1.00
1.00
1.03
1.03
3.12
1.03
2.07
2.08
2.06
3.11
3.07
5.11
f1 (ppm)

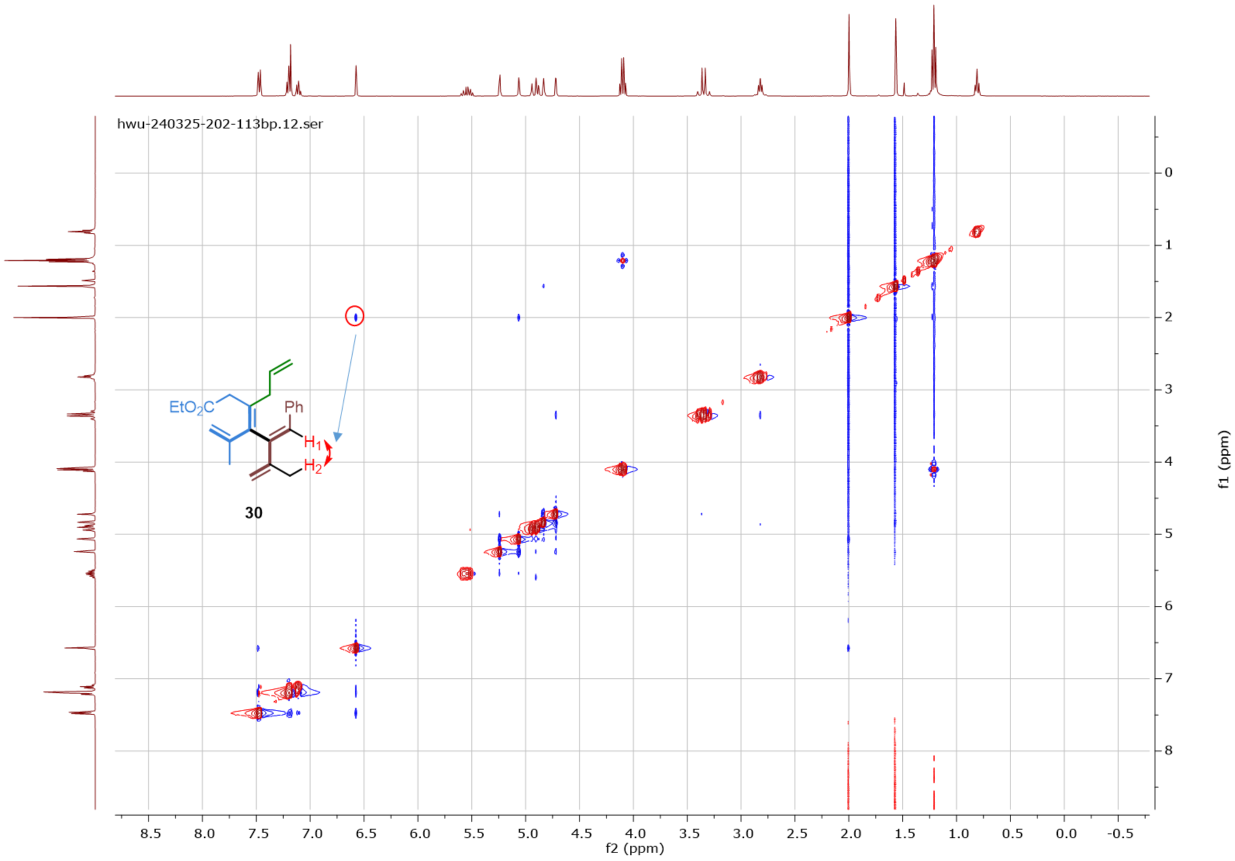
hwu-240325-202-113bp.12.ser
EtO2C
Ph
H1
H2
30
f2 (ppm)
f1 (ppm)

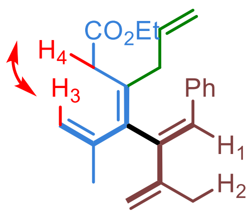

30

significant NOESY correlation

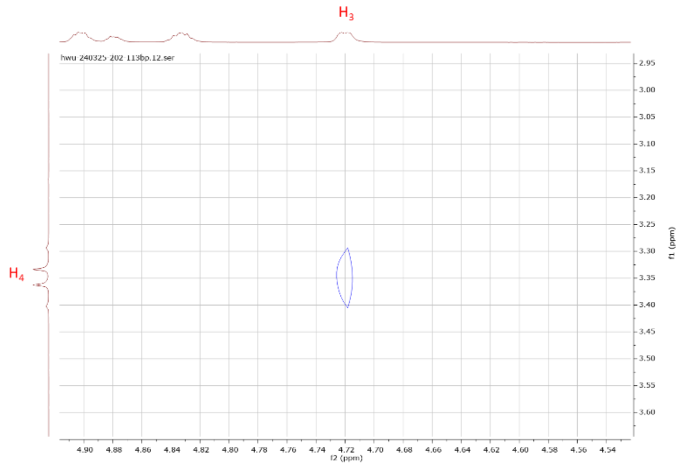

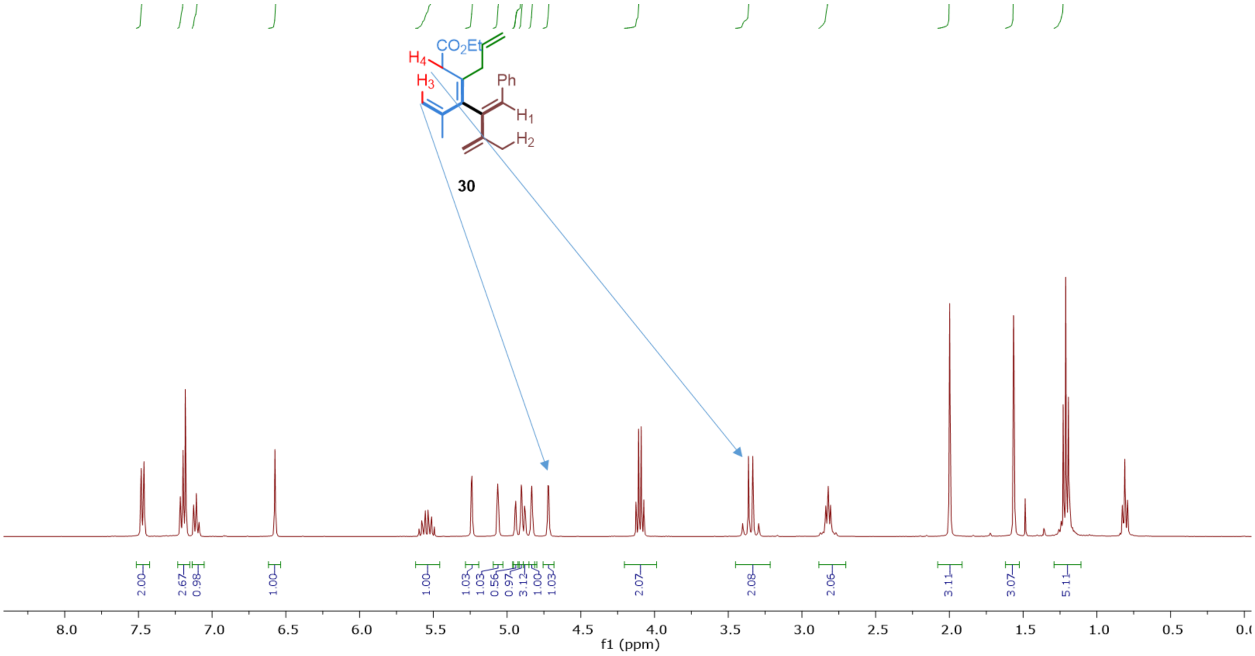

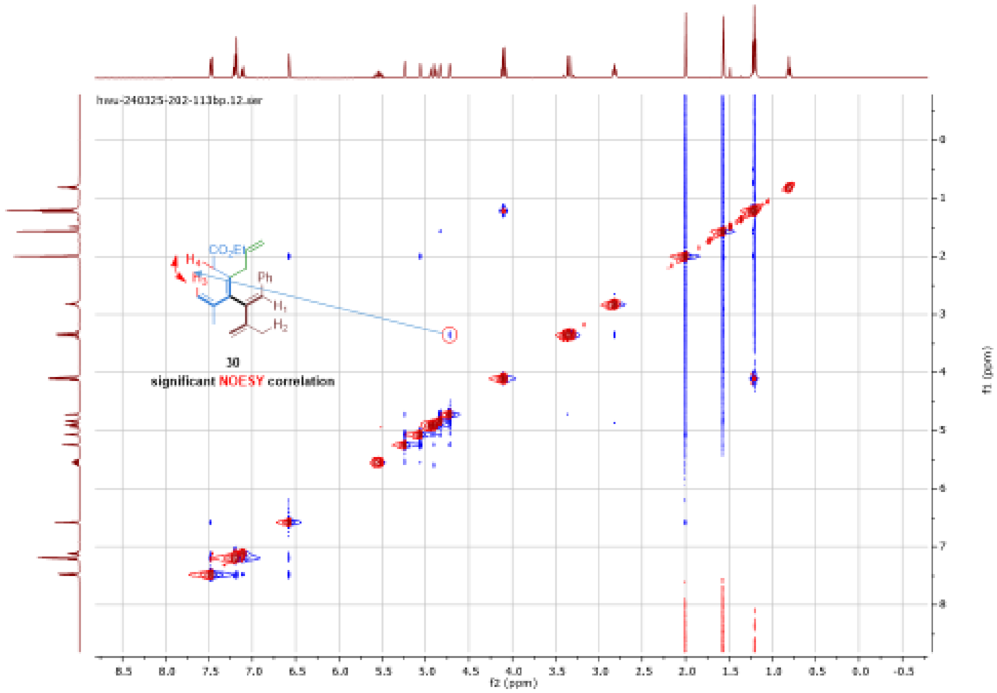

Confirmation of Stereochemistry of [4]Dendralene 38 (Type-II):

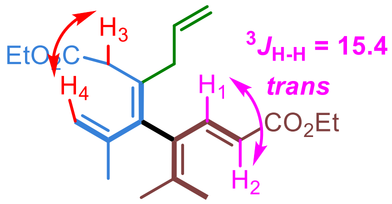

38

significant NOESY correlation

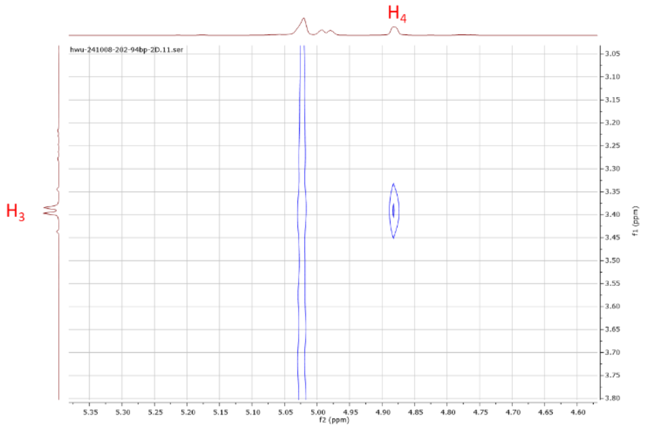

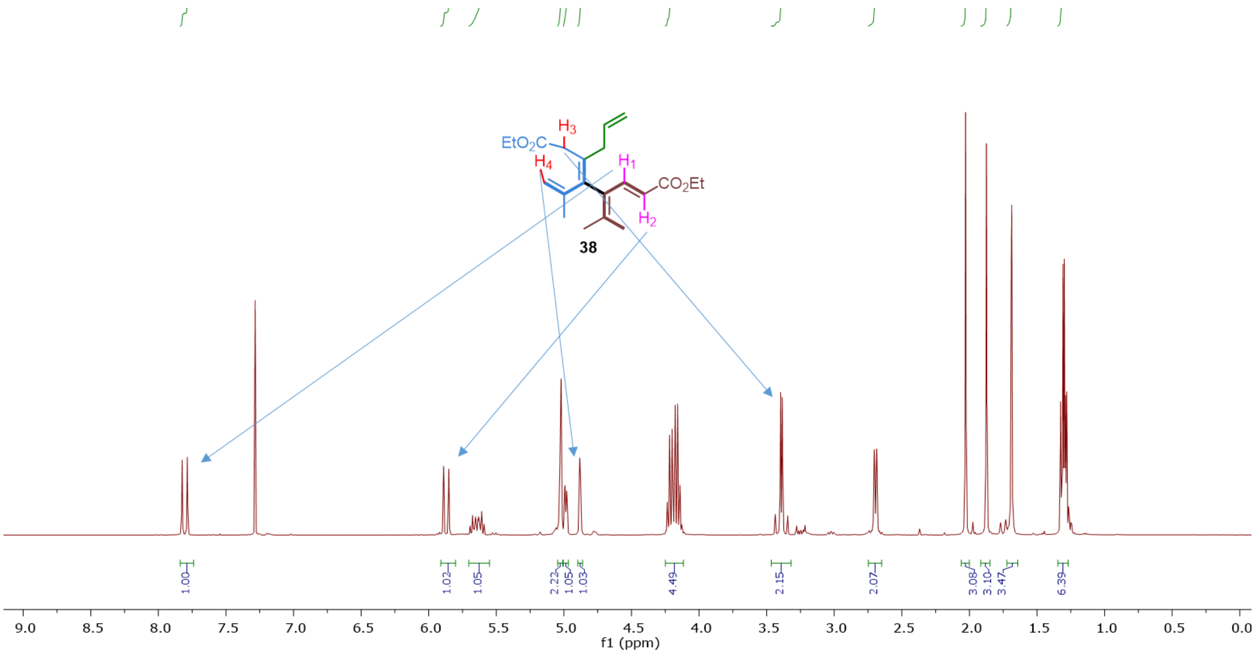
EtO2C
H3
H4
H1
CO2Et
H2
38
1.00
1.02
1.05
2.22
1.05
1.03
4.49
2.15
2.07
3.08
3.10
3.47
6.39
f1 (ppm)

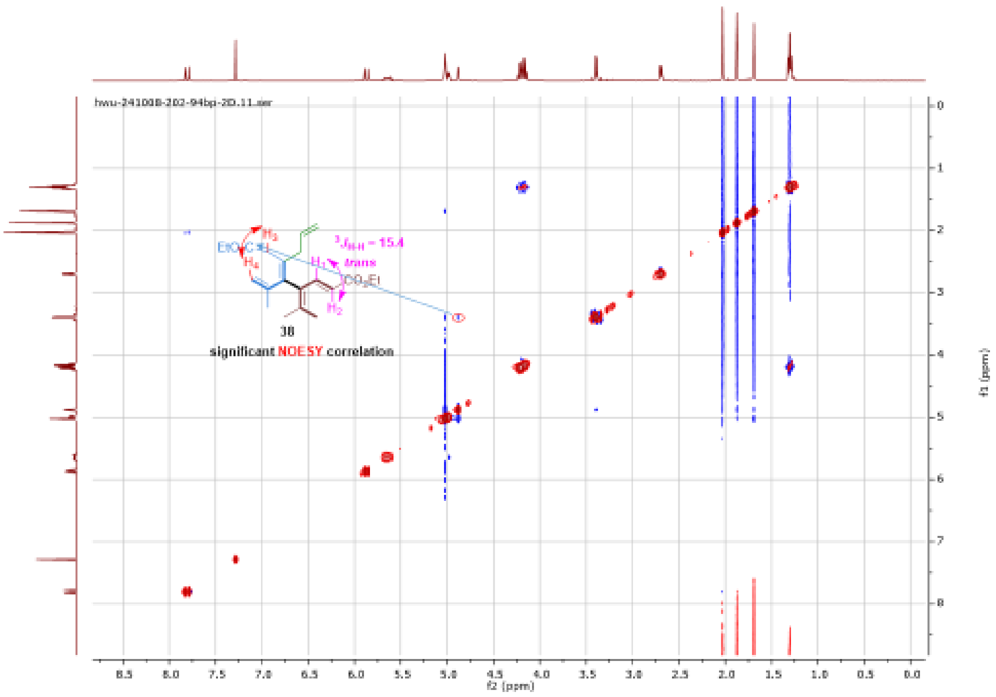
hwu-241008-202-94bp-2D.11.ser
EtO2C
H3
H4
H1
$^{3}J_{H-H}$ = 15.4
trans
CO2Et
H2
38
significant NOESY correlation
f2 (ppm)
f1 (ppm)

Confirmation of Stereochemistry of [4]Dendralene 49 (Type-II):

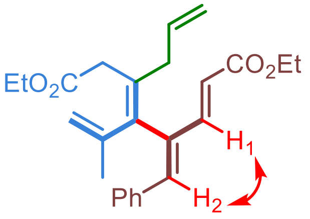

49

significant NOESY correlation

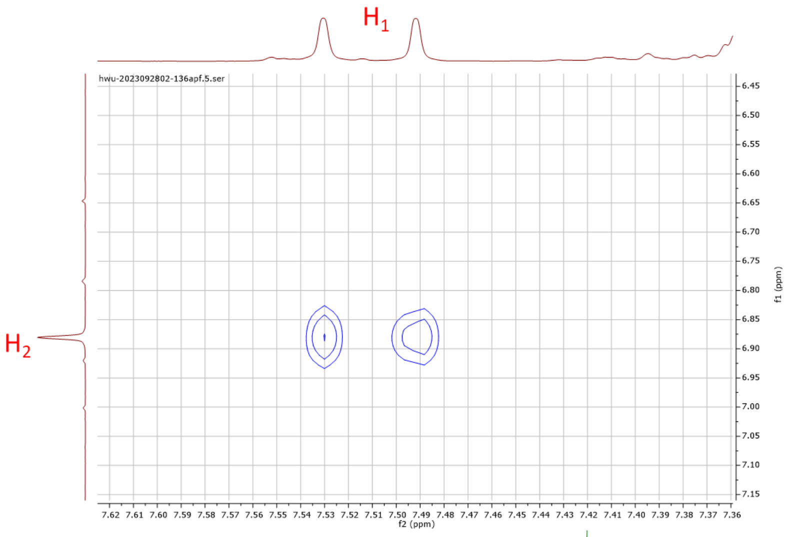

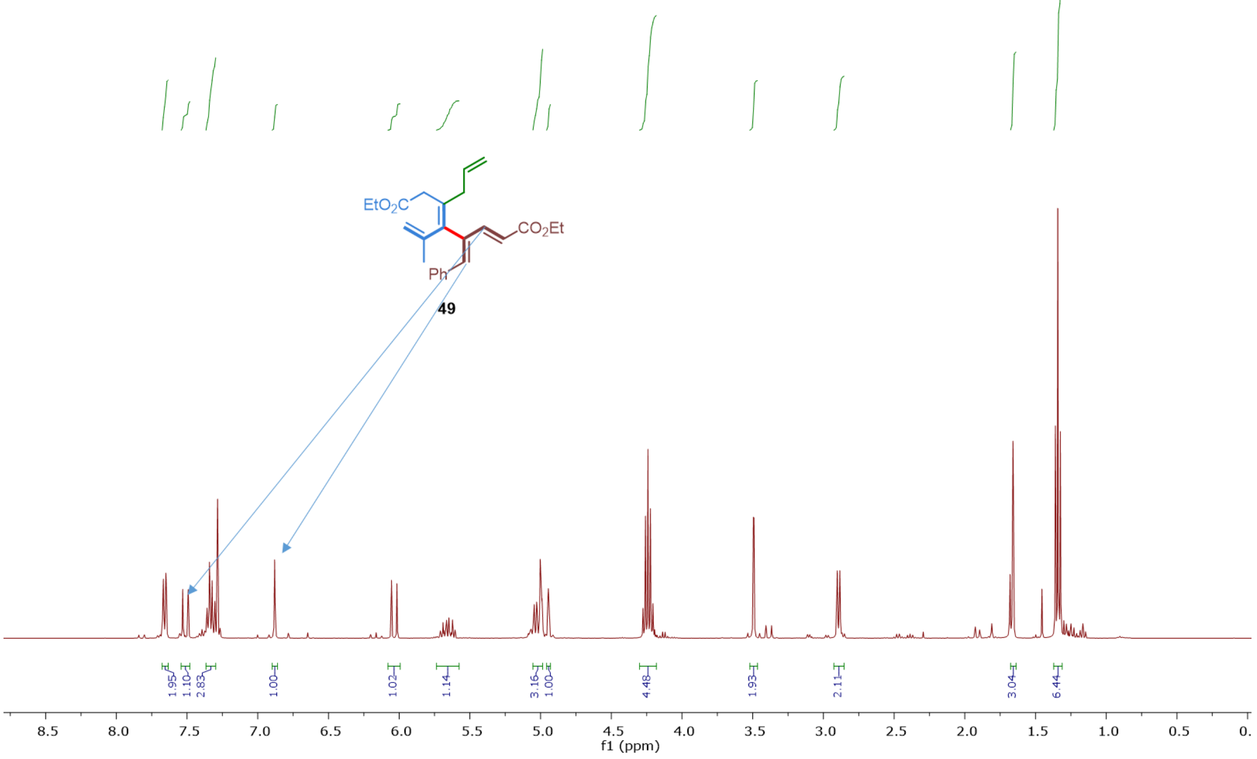

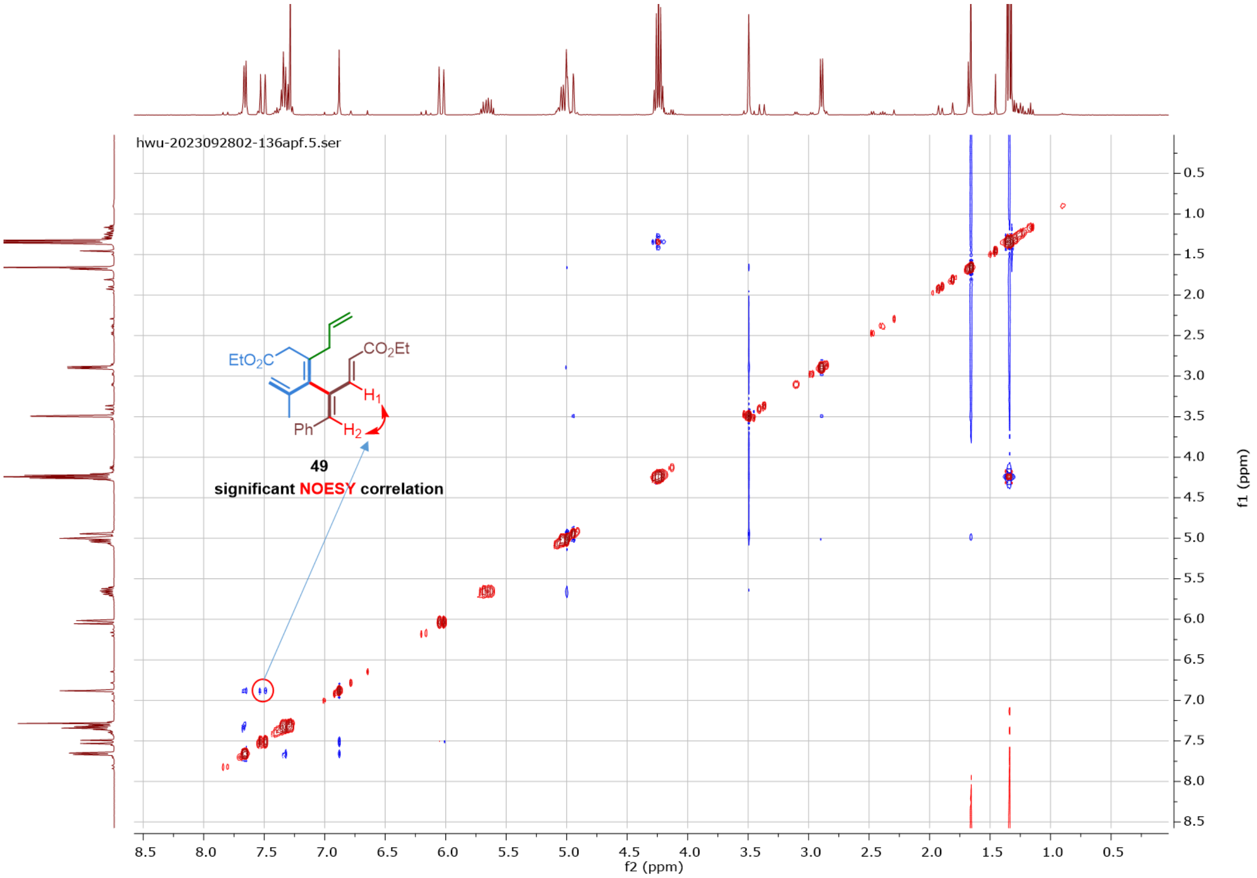

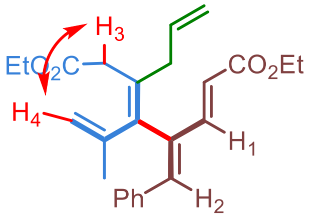

49

significant NOESY correlation

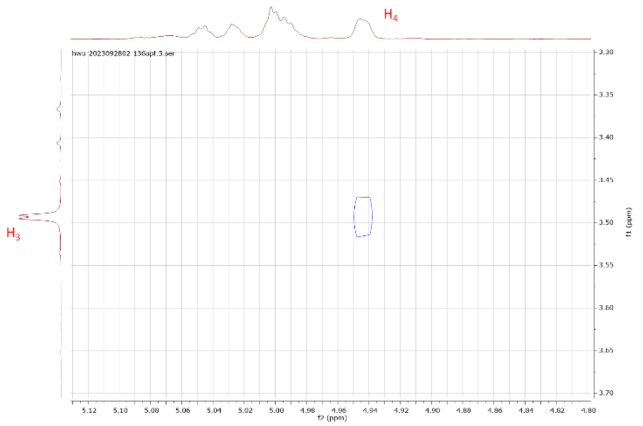

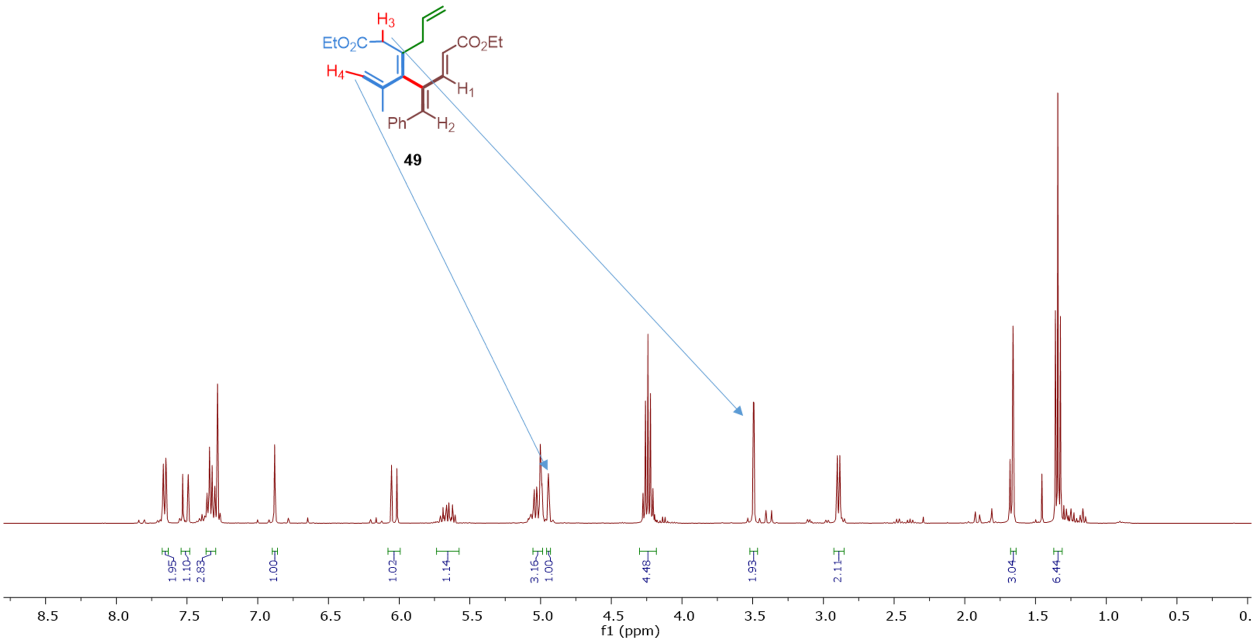

H_3
EtO_2C
CO_2Et
H_4
H_1
Ph
H_2
49
1.95
1.10
2.83
1.00
1.02
1.14
3.16
1.00
4.48
1.93
2.11
3.04
6.44
f1 (ppm)

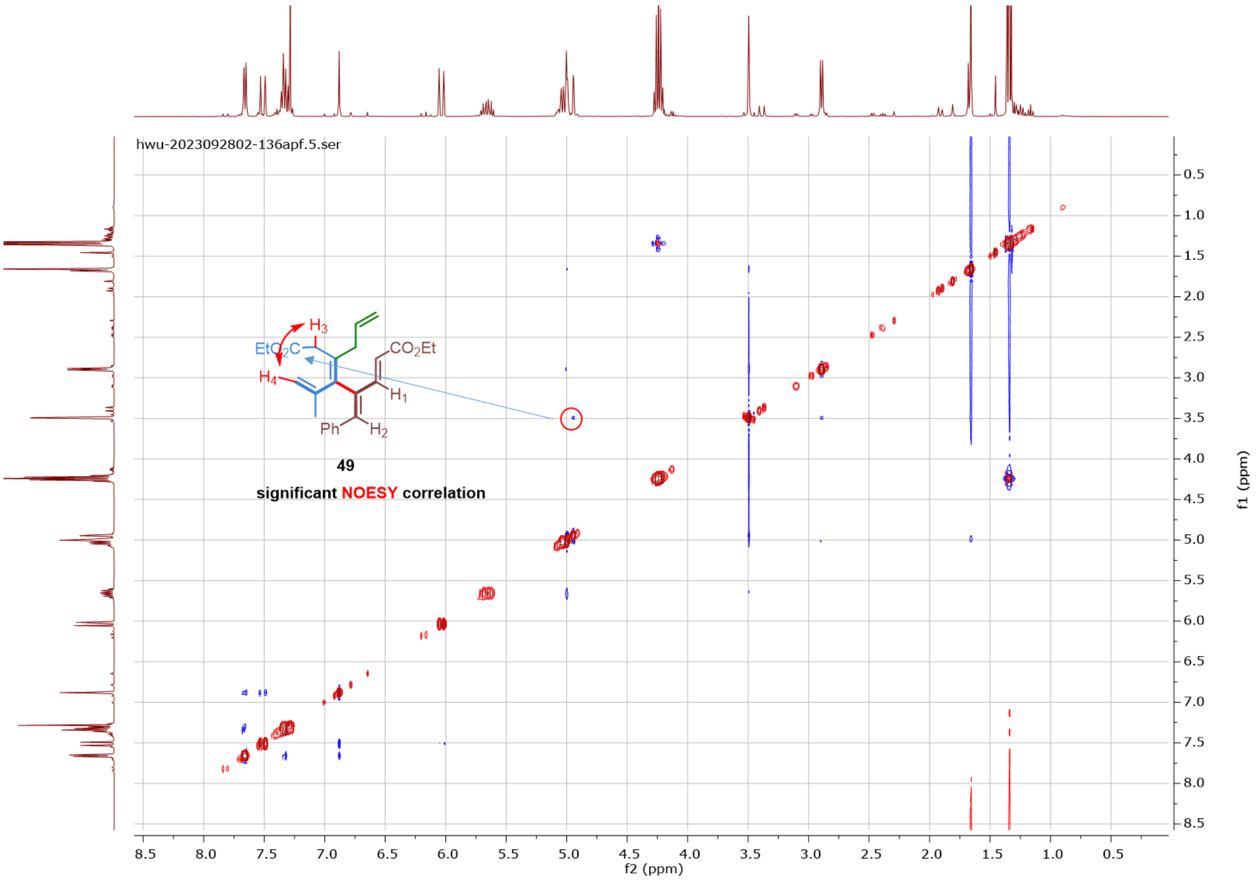

hwu-2023092802-136apf.5.ser
H_3
EtO_2C
CO_2Et
H_4
H_1
Ph
H_2
49
significant NOESY correlation
f2 (ppm)
f1 (ppm)

Confirmation of Relative Stereochemistry of Compound (±) 67:

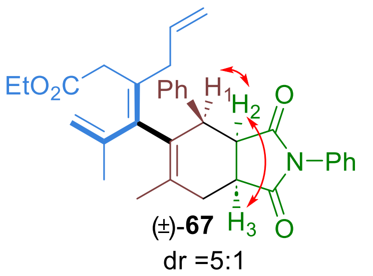

significant NOESY correlation

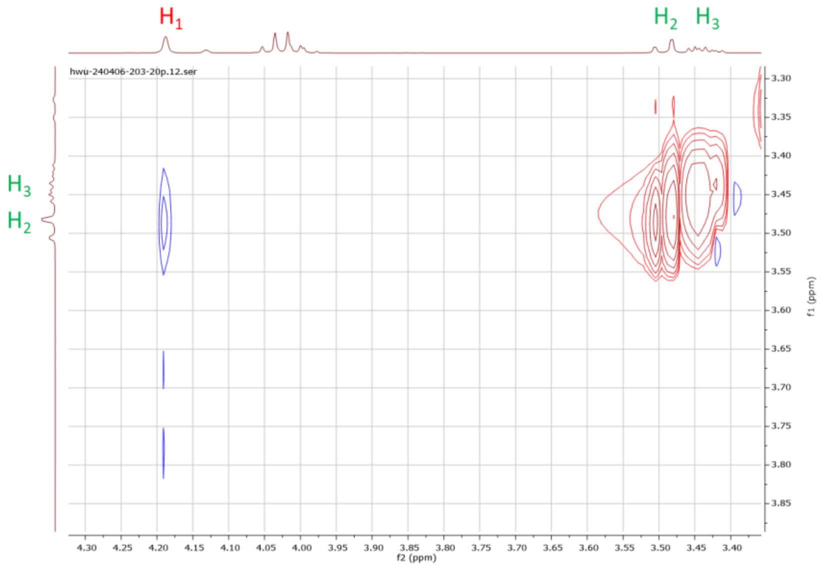

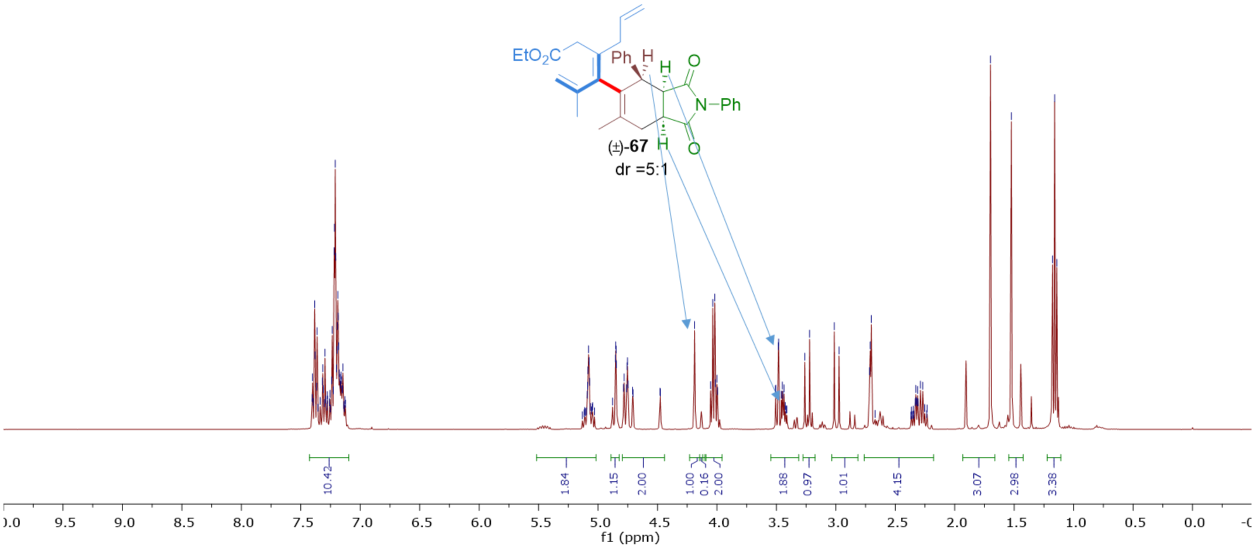

The assignment of benzylic proton (H_1) was confirmed by the following HSQC spectrum:

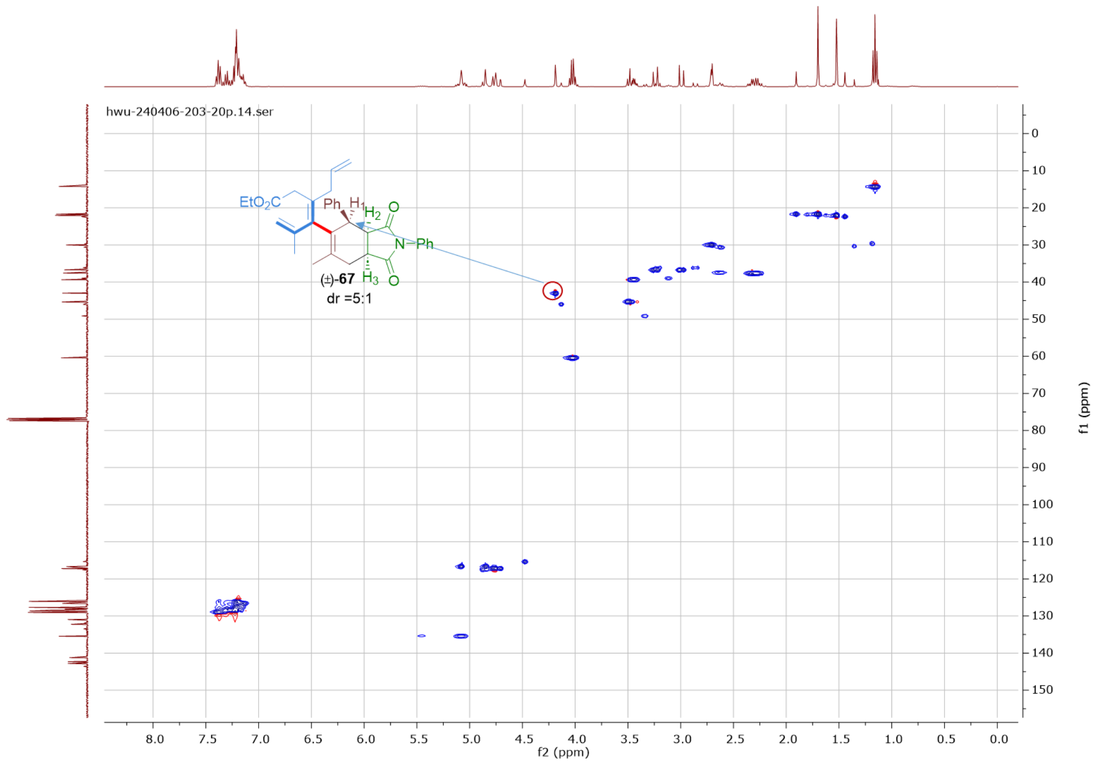

The assignments of protons H_2 and H_3 were confirmed by the following COSY spectrum:

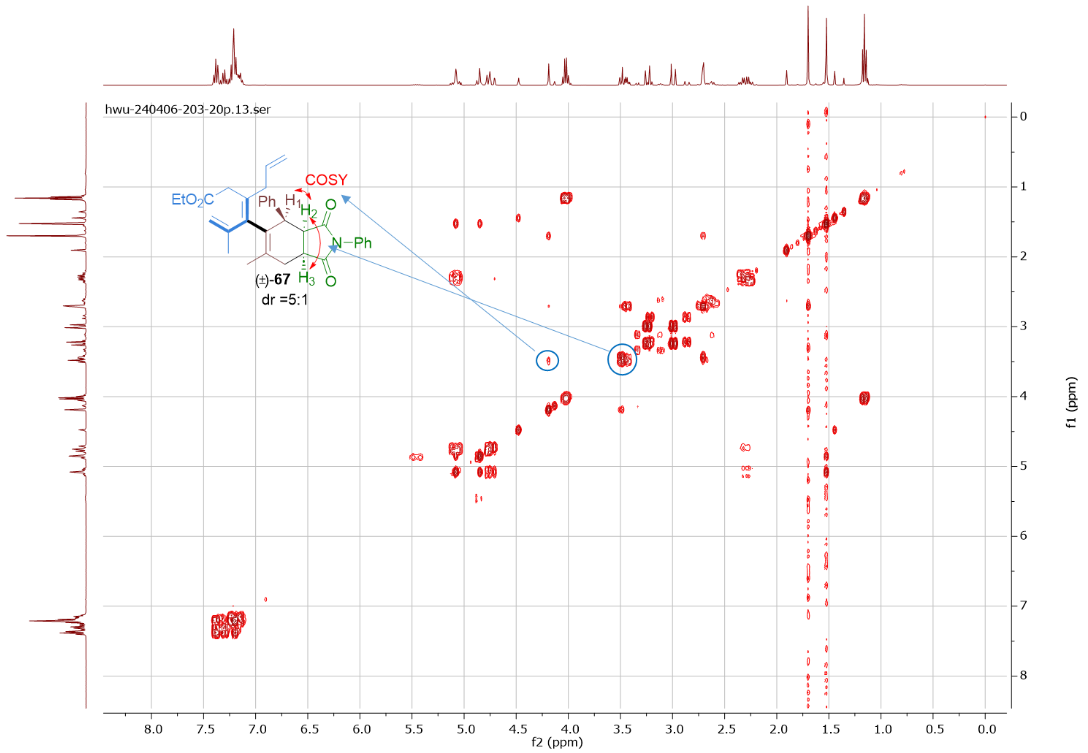

The relative stereochemistry of compound **(±) 67** was confirmed by the following NOESY spectrum:

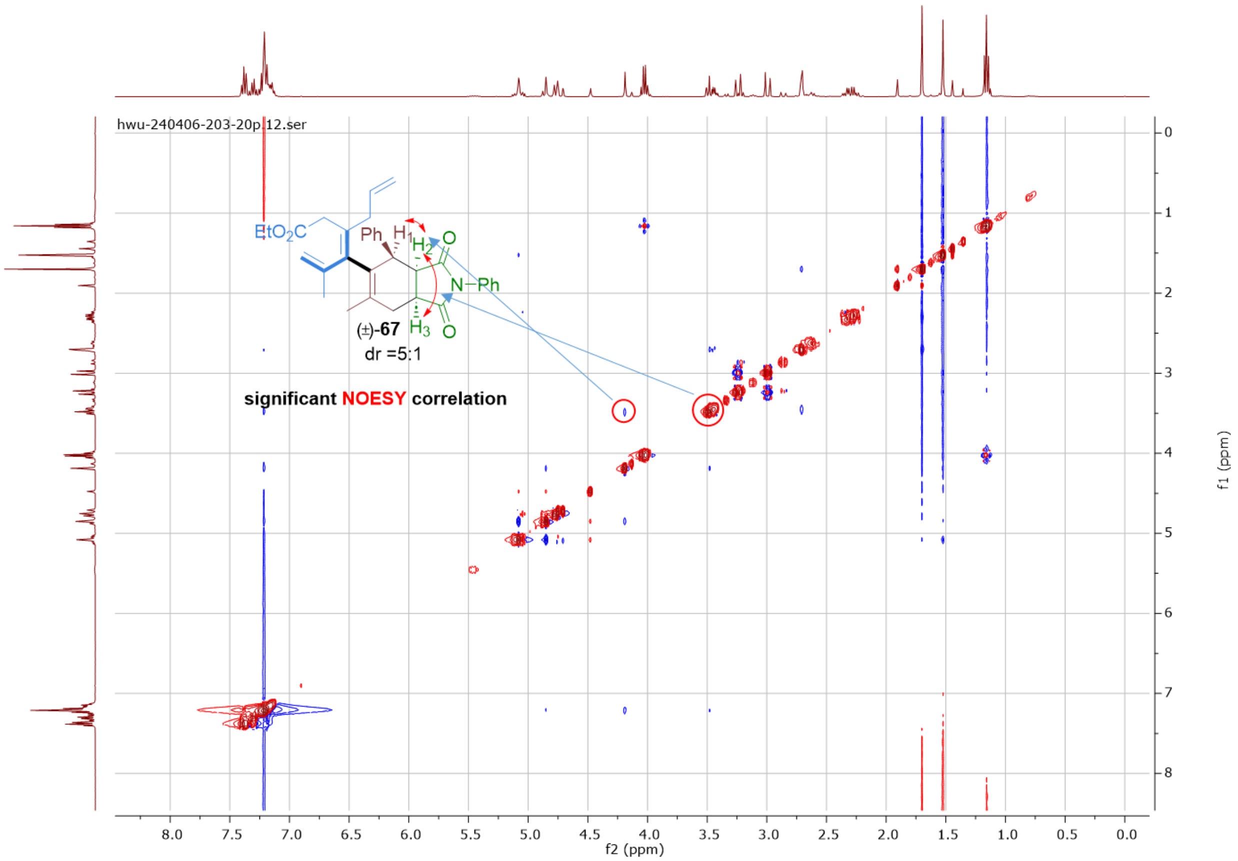

6. NMR Spectra of New Compounds

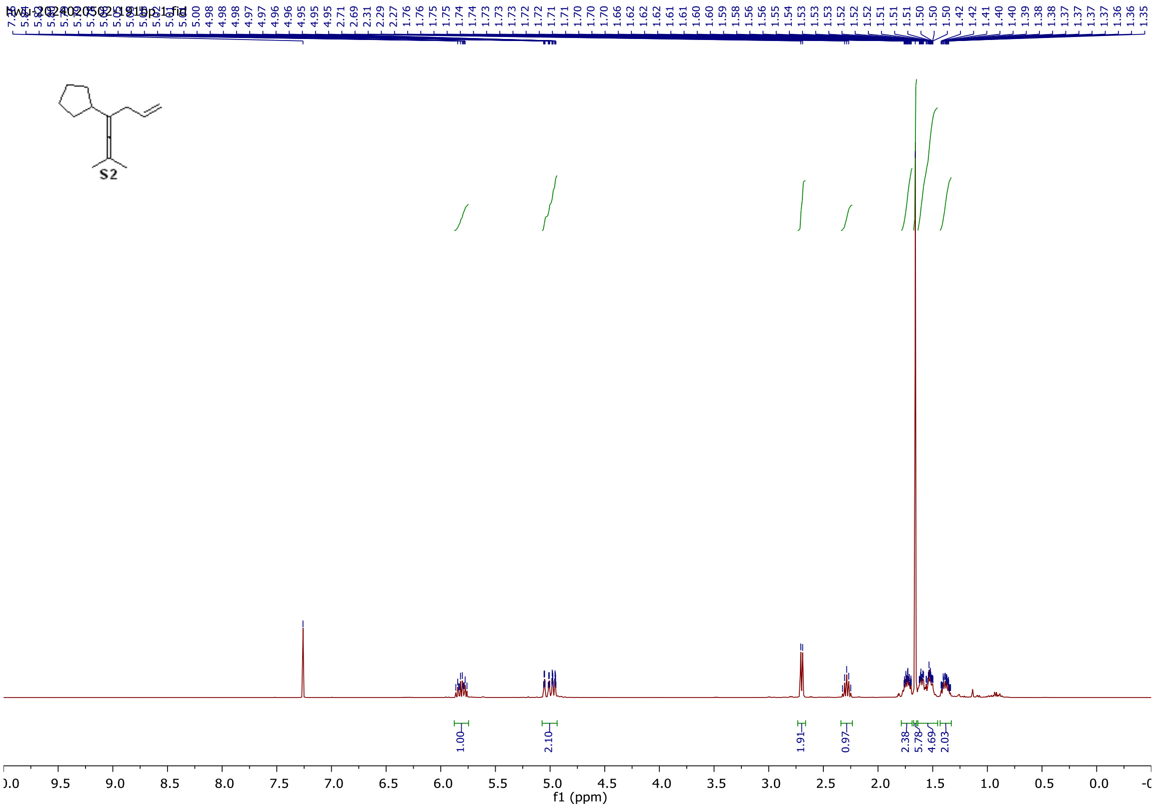

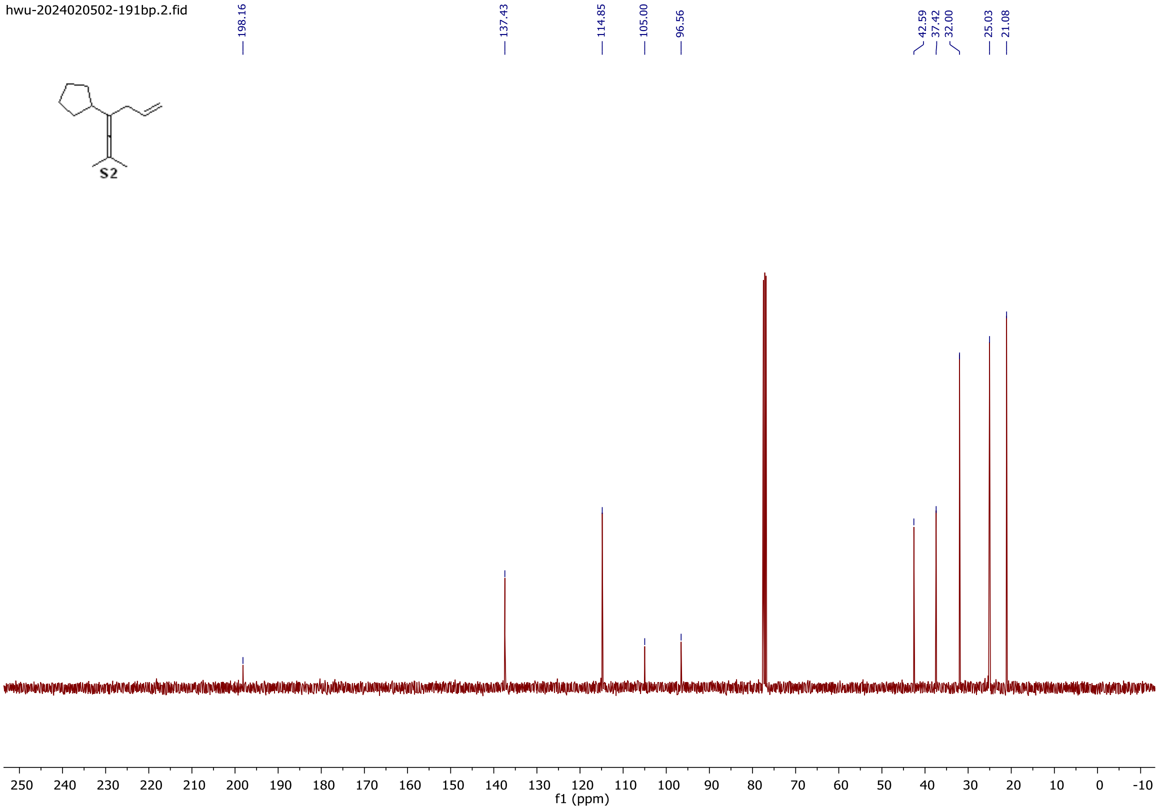

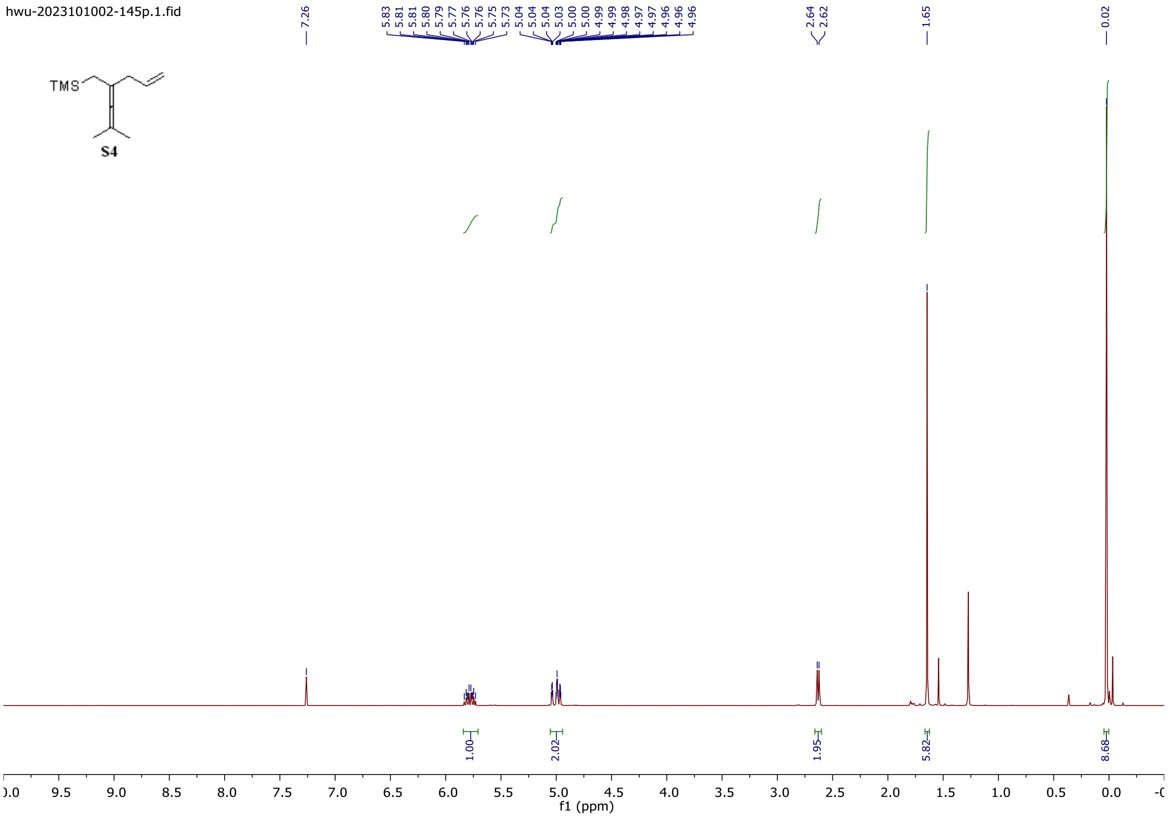
hwu-2023101002-145p.1.fid
TMS
S4
7.26
5.83 5.81 5.81 5.80 5.79 5.77 5.76 5.76 5.75 5.73 5.04 5.04 5.04 5.03 5.00 5.00 4.99 4.99 4.98 4.97 4.97 4.96 4.96 4.96
2.64 2.62
1.65
0.02
1.00
2.02
1.95
5.82
8.68
f1 (ppm)

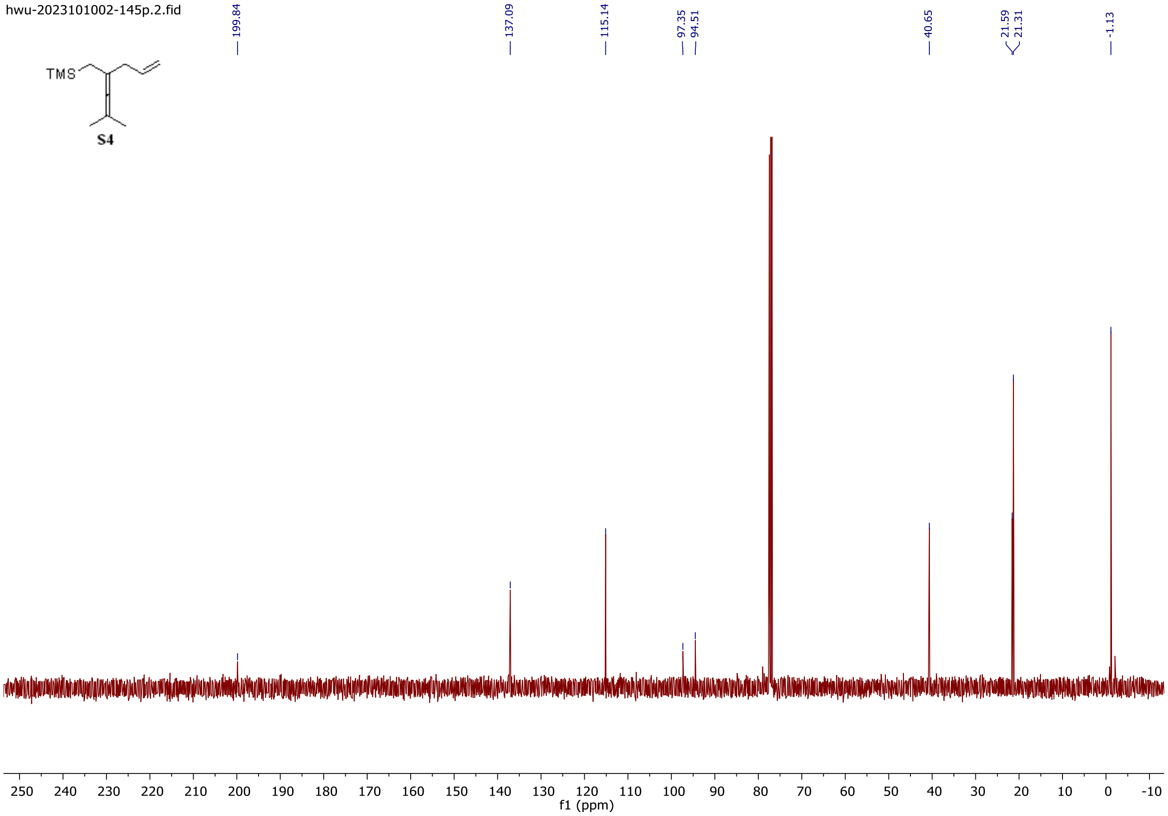
hwu-2023101002-145p.2.fid
199.84
137.09
115.14
97.35
94.51
40.65
21.59
21.31
-1.13
TMS
S4
f1 (ppm)

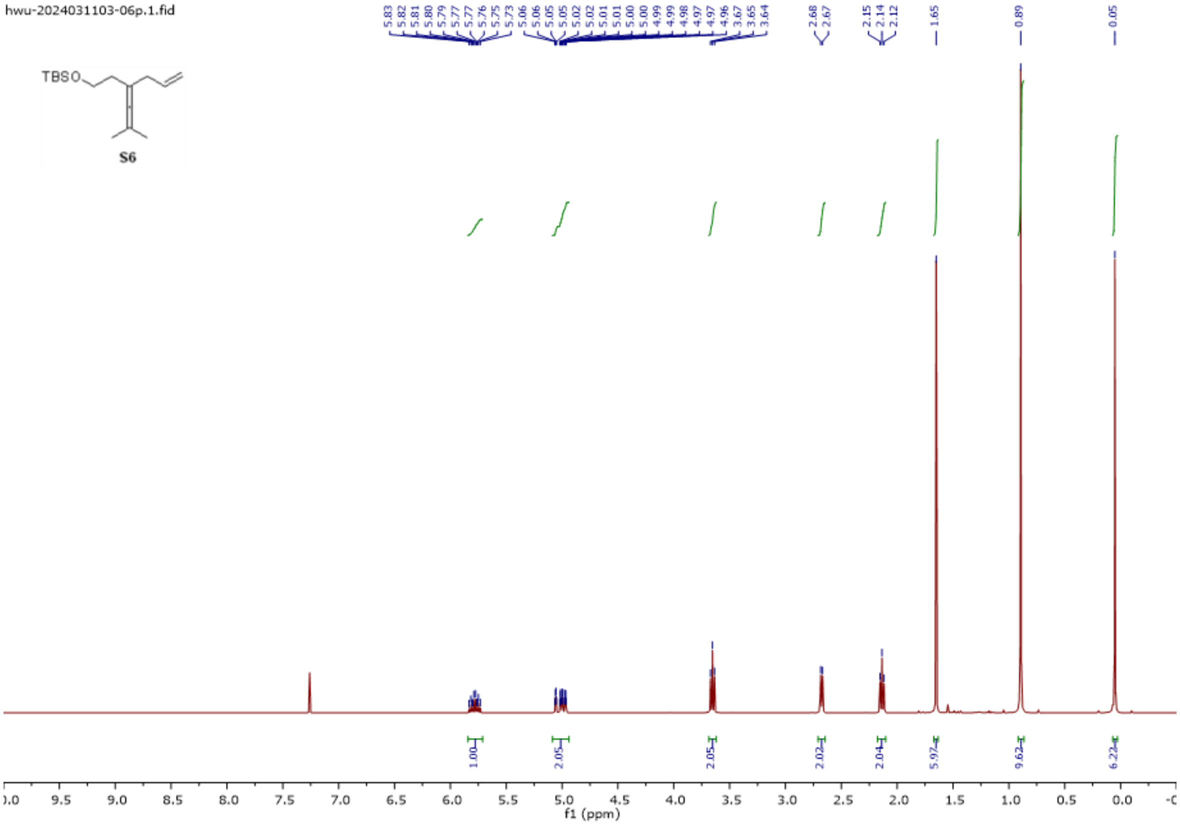
hwu-2024031103-06p.1.fid
5.83 5.82 5.81 5.80 5.79 5.77 5.77 5.76 5.75 5.73 5.06 5.06 5.05 5.05 5.02 5.02 5.01 5.01 5.00 5.00 4.99 4.99 4.98 4.97 4.97 4.96 3.67 3.65 3.64
2.68 2.67
2.15 2.14 2.12
1.65
0.89
0.05
TBSO
S6
1.00
2.05
2.05
2.02
2.04
5.97
9.62
6.23
f1 (ppm)

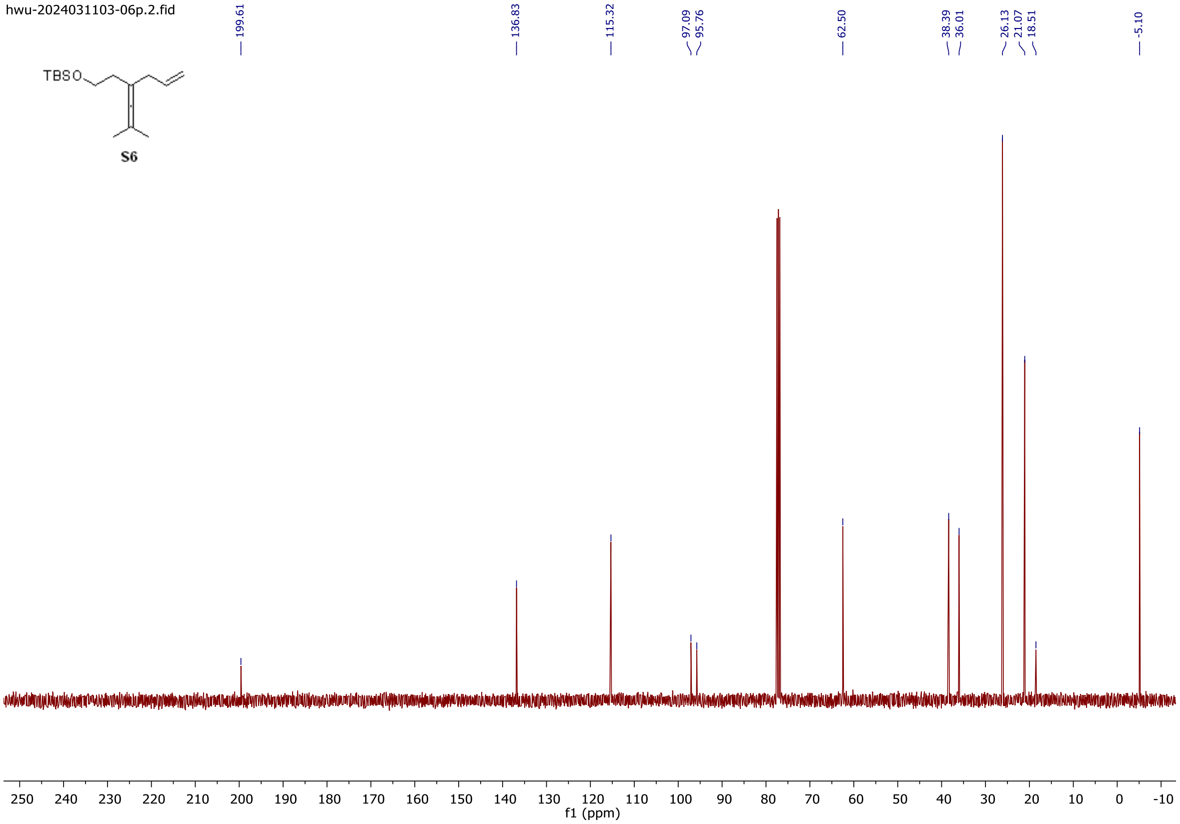
hwu-2024031103-06p.2.fid
199.61
136.83
115.32
97.09
95.76
62.50
38.39
36.01
26.13
21.07
18.51
-5.10
TBSO
S6
f1 (ppm)

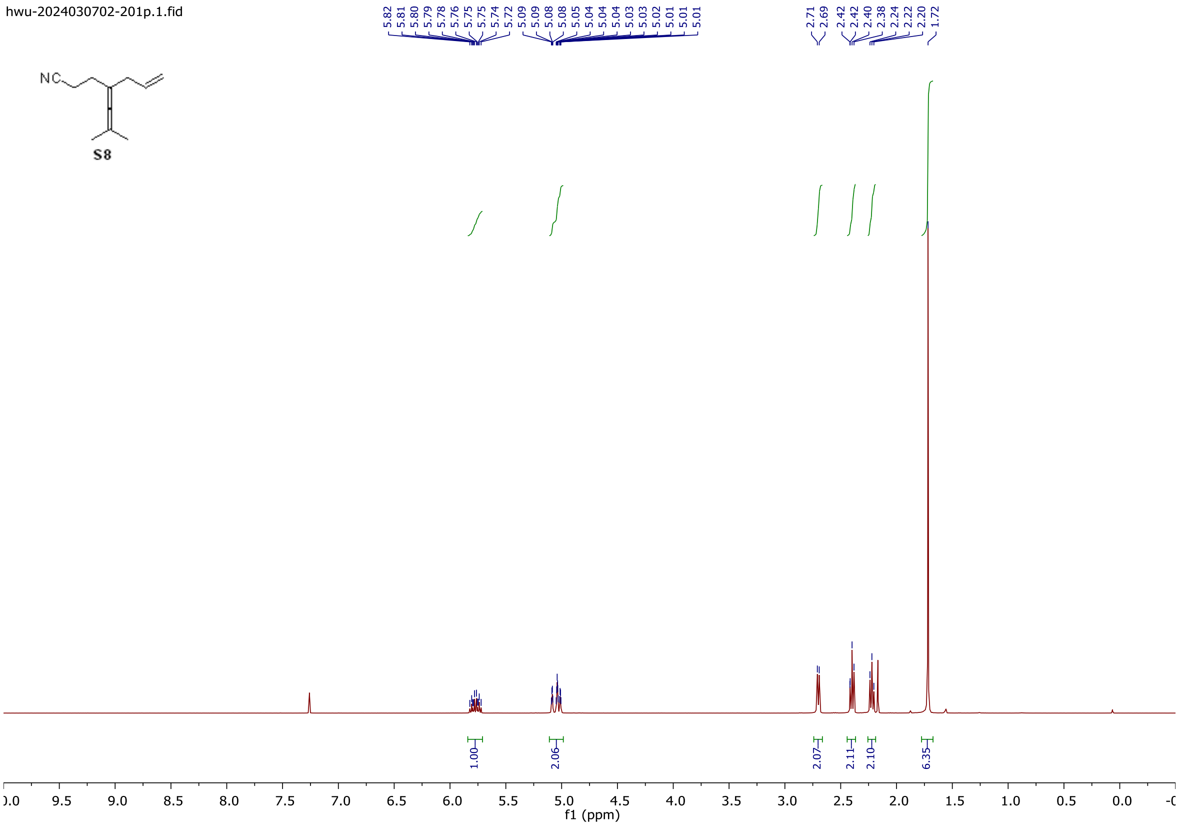
hwu-2024030702-201p.1.fid
NC
S8
5.82
5.81
5.80
5.79
5.78
5.76
5.75
5.75
5.74
5.72
5.09
5.09
5.08
5.08
5.05
5.04
5.04
5.04
5.03
5.03
5.02
5.01
5.01
5.01
2.71
2.69
2.42
2.42
2.40
2.38
2.24
2.22
2.20
1.72
1.00
2.06
2.07
2.11
2.10
6.35
f1 (ppm)

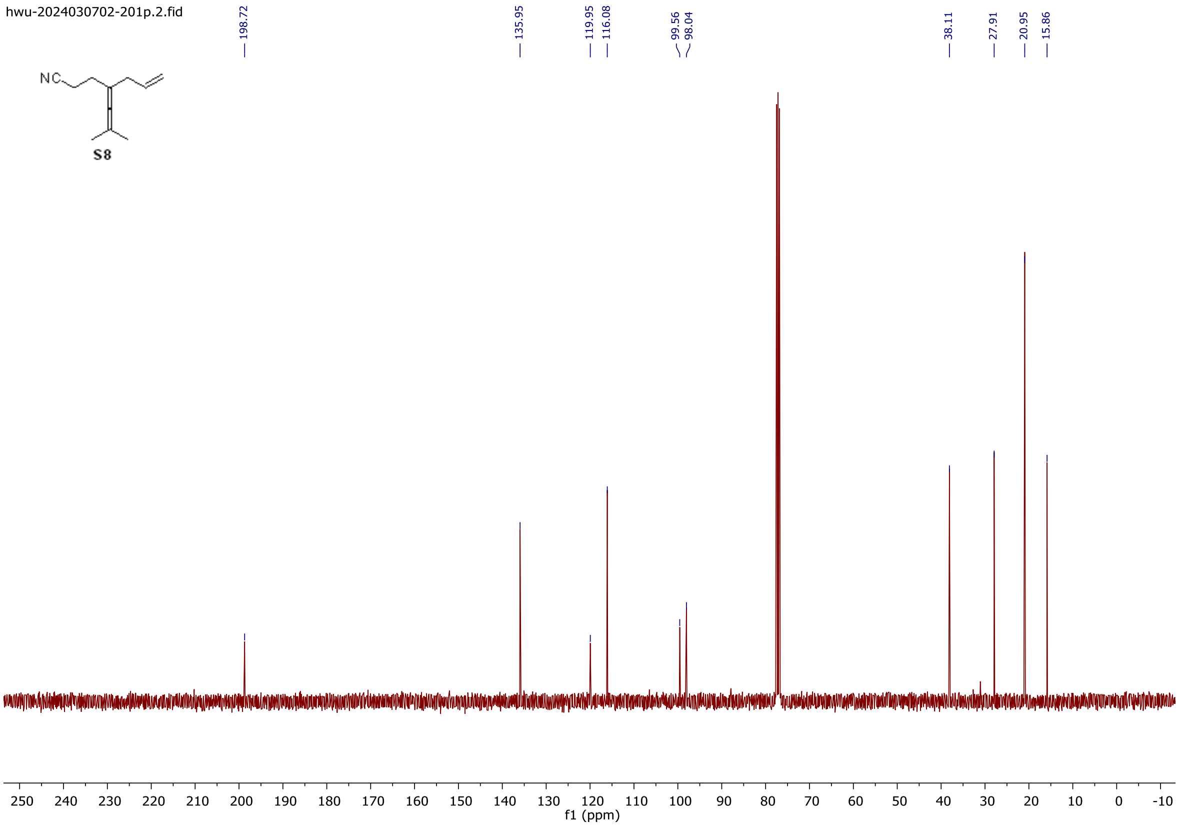
hwu-2024030702-201p.2.fid
NC
S8
198.72
135.95
119.95
116.08
99.56
98.04
38.11
27.91
20.95
15.86
f1 (ppm)

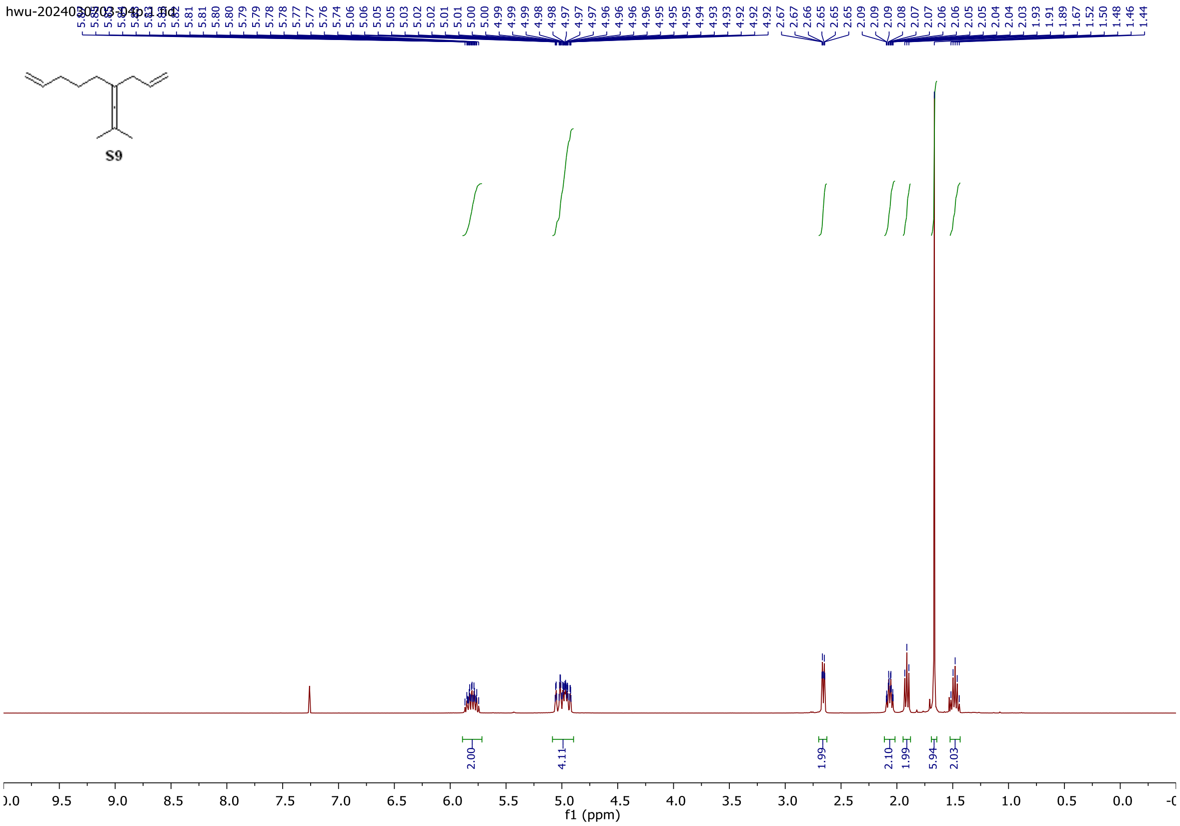
hwu-2024030703-04p.1.fid
5.81 5.81 5.80 5.79 5.79 5.78 5.78 5.77 5.77 5.76 5.74 5.06 5.06 5.05 5.05 5.03 5.02 5.02 5.01 5.01 5.00 5.00 4.99 4.99 4.99 4.98 4.98 4.97 4.97 4.97 4.96 4.96 4.96 4.96 4.95 4.95 4.95 4.94 4.93 4.93 4.92 4.92 4.92 2.67 2.67 2.66 2.65 2.65 2.65 2.09 2.09 2.09 2.08 2.07 2.07 2.06 2.06 2.05 2.05 2.04 2.04 2.03 1.93 1.91 1.89 1.67 1.52 1.50 1.48 1.46 1.44
S9
2.00
4.11
1.99
2.10
1.99
5.94
2.03
f1 (ppm)

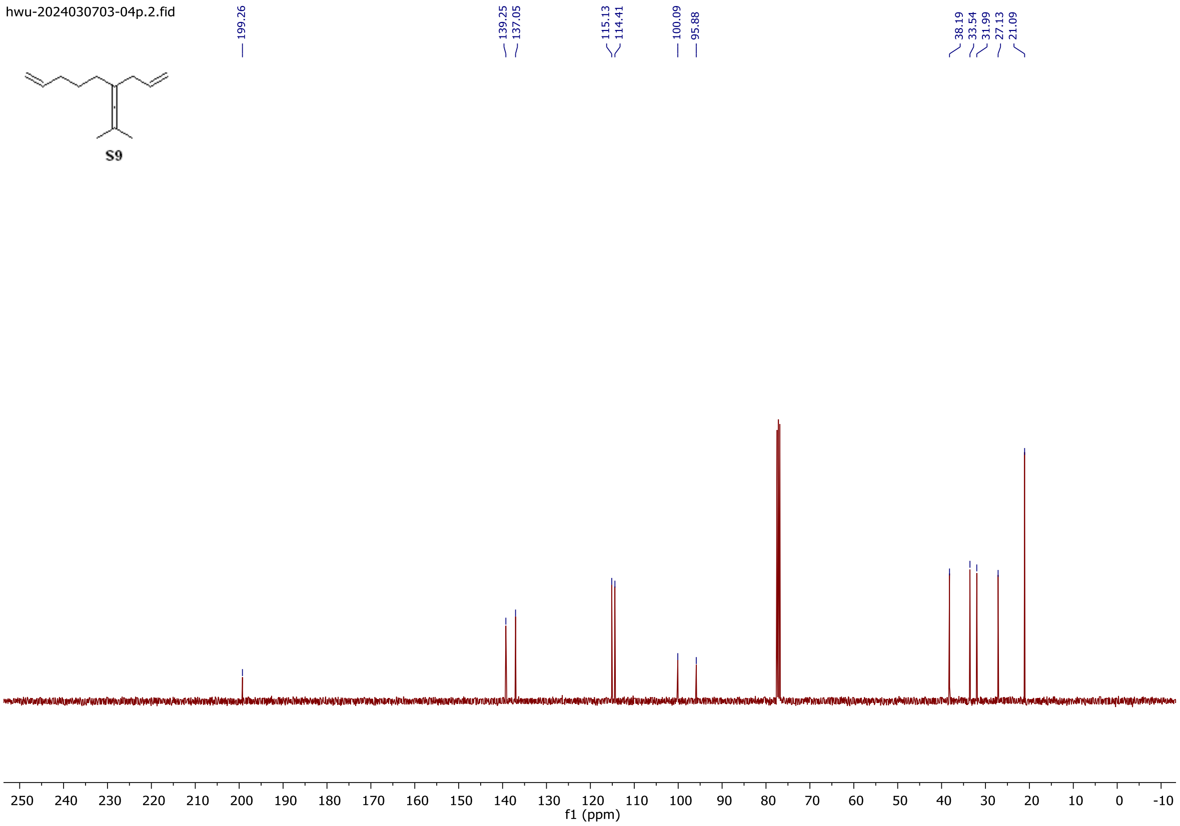
hwu-2024030703-04p.2.fid
199.26
139.25
137.05
115.13
114.41
100.09
95.88
38.19
33.54
31.99
27.13
21.09
S9
f1 (ppm)

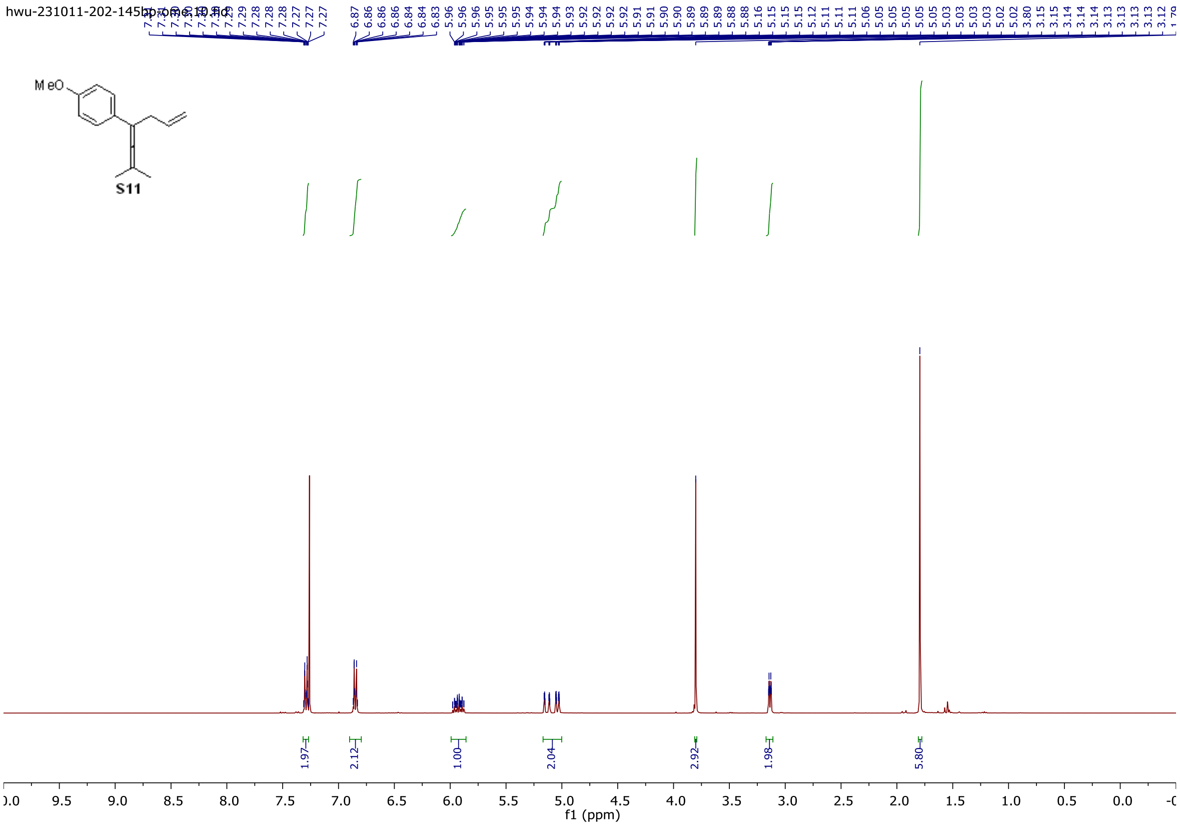
hwu-231011-202-145bp-ome.10.fid
MeO
S11
7.29
7.28
7.28
7.28
7.27
7.27
7.27
6.87
6.86
6.86
6.86
6.84
6.84
6.83
5.96
5.96
5.96
5.95
5.95
5.95
5.94
5.94
5.94
5.93
5.92
5.92
5.92
5.92
5.91
5.91
5.90
5.89
5.89
5.89
5.88
5.16
5.15
5.15
5.12
5.11
5.11
5.11
5.06
5.05
5.05
5.05
5.05
5.05
5.03
5.03
5.03
5.03
5.02
3.80
3.15
3.15
3.14
3.14
3.13
3.13
3.13
3.13
3.12
1.97
2.12
1.00
2.04
2.92
1.98
5.80
f1 (ppm)

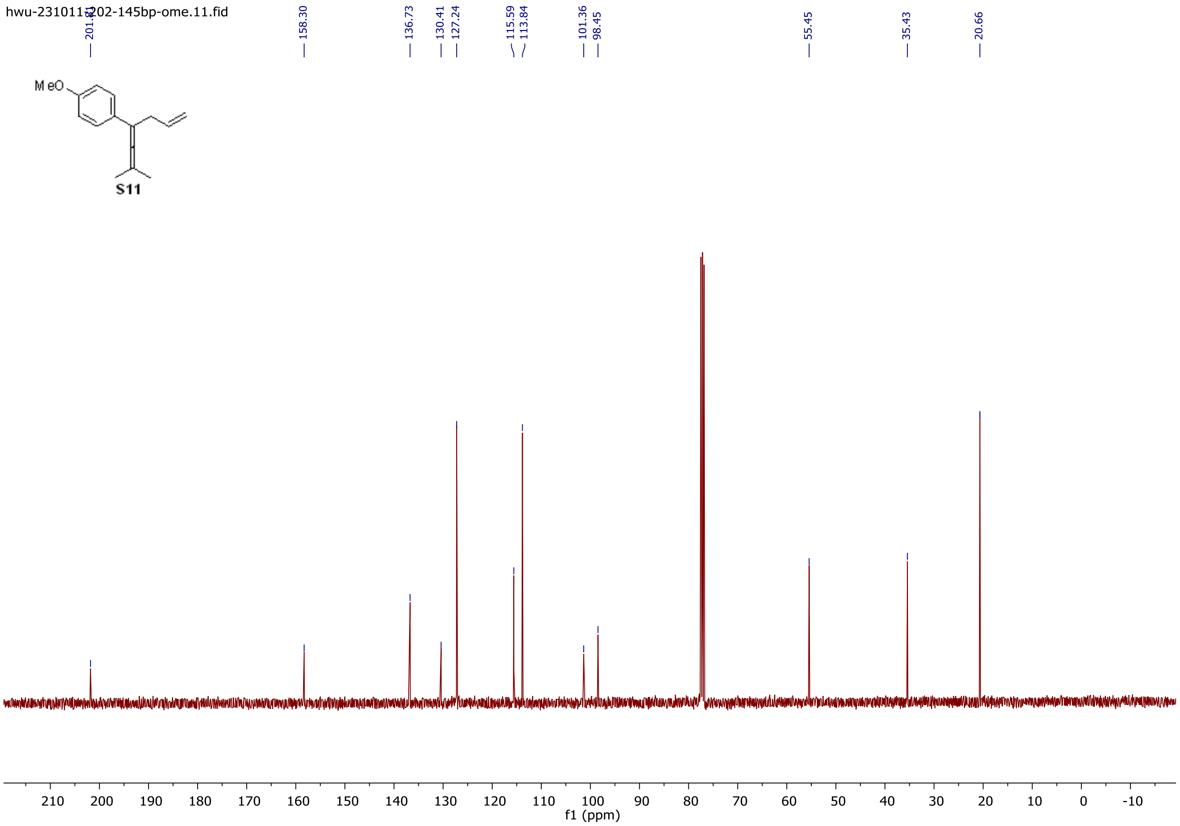
hwu-231011-202-145bp-ome.11.fid
201.81
158.30
136.73
130.41
127.24
115.59
113.84
101.36
98.45
55.45
35.43
20.66
MeO
S11
f1 (ppm)

hwu-2023101202-145cp.1.fid

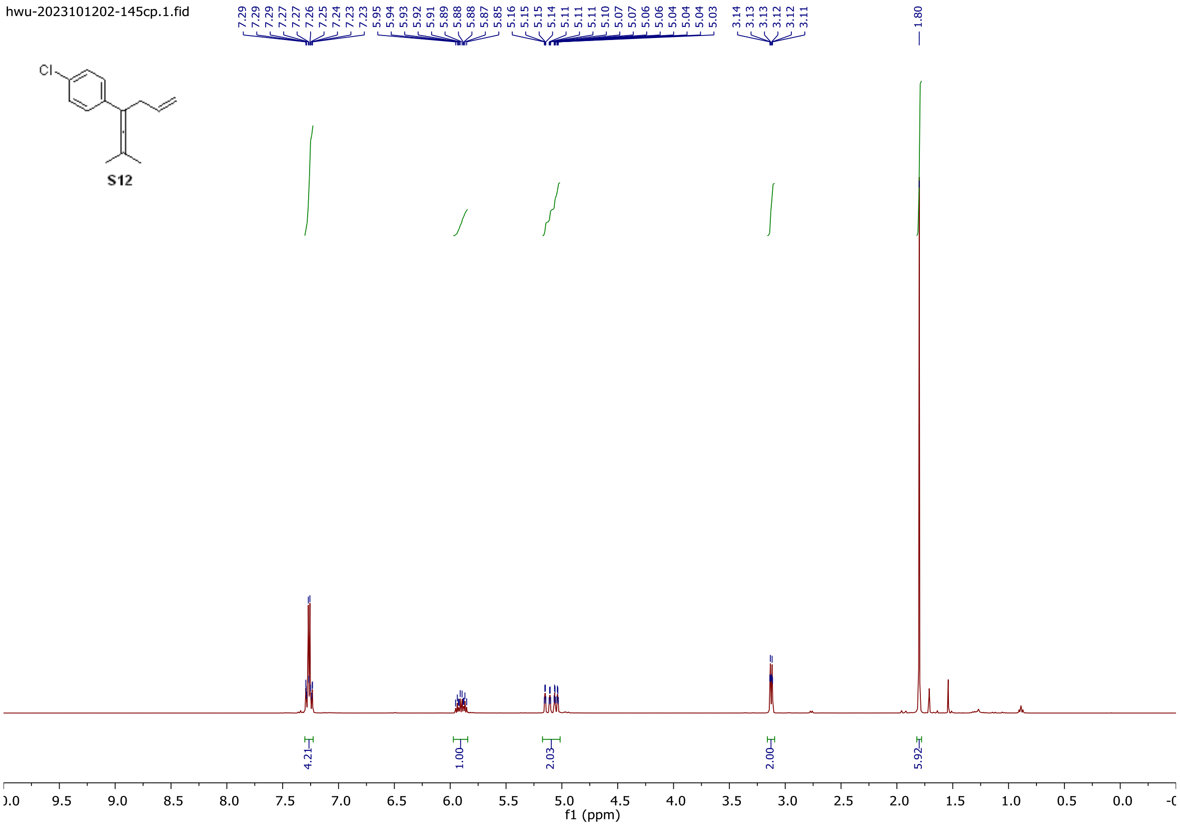

hwu-2023101202-145cp.2.fid

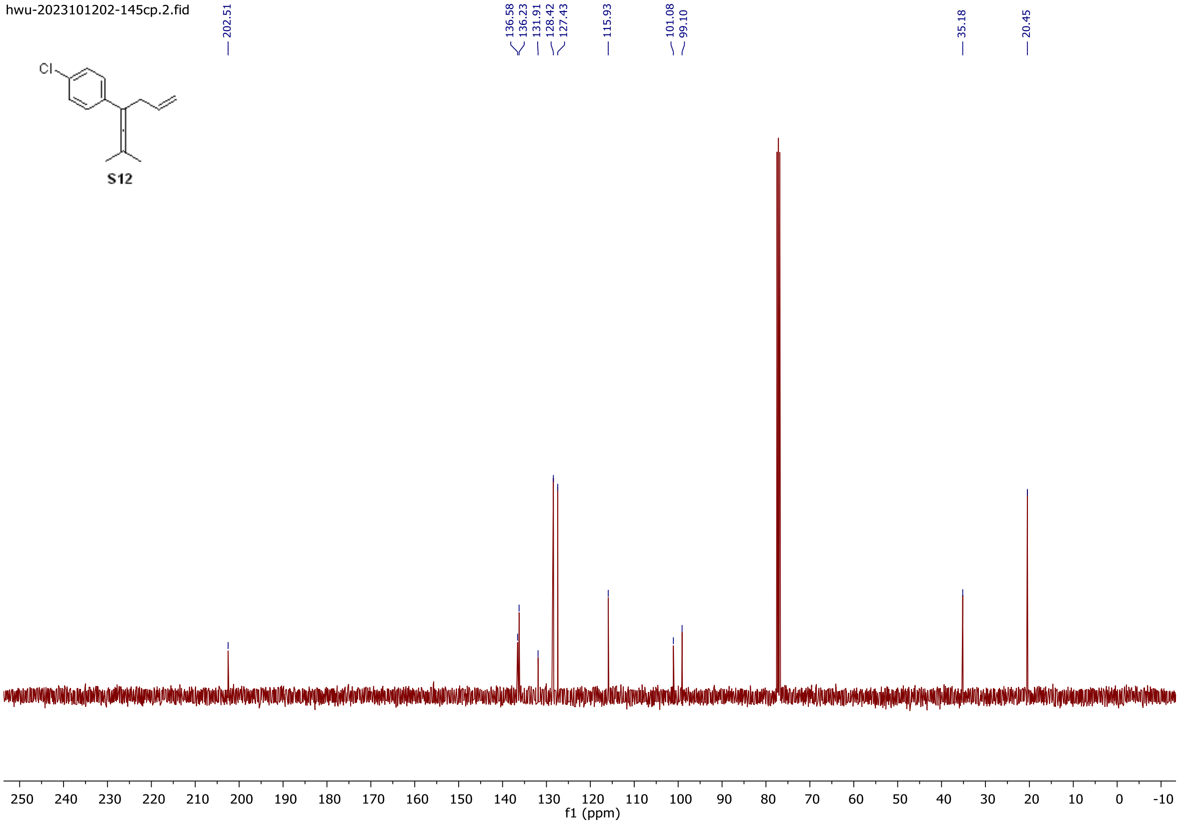

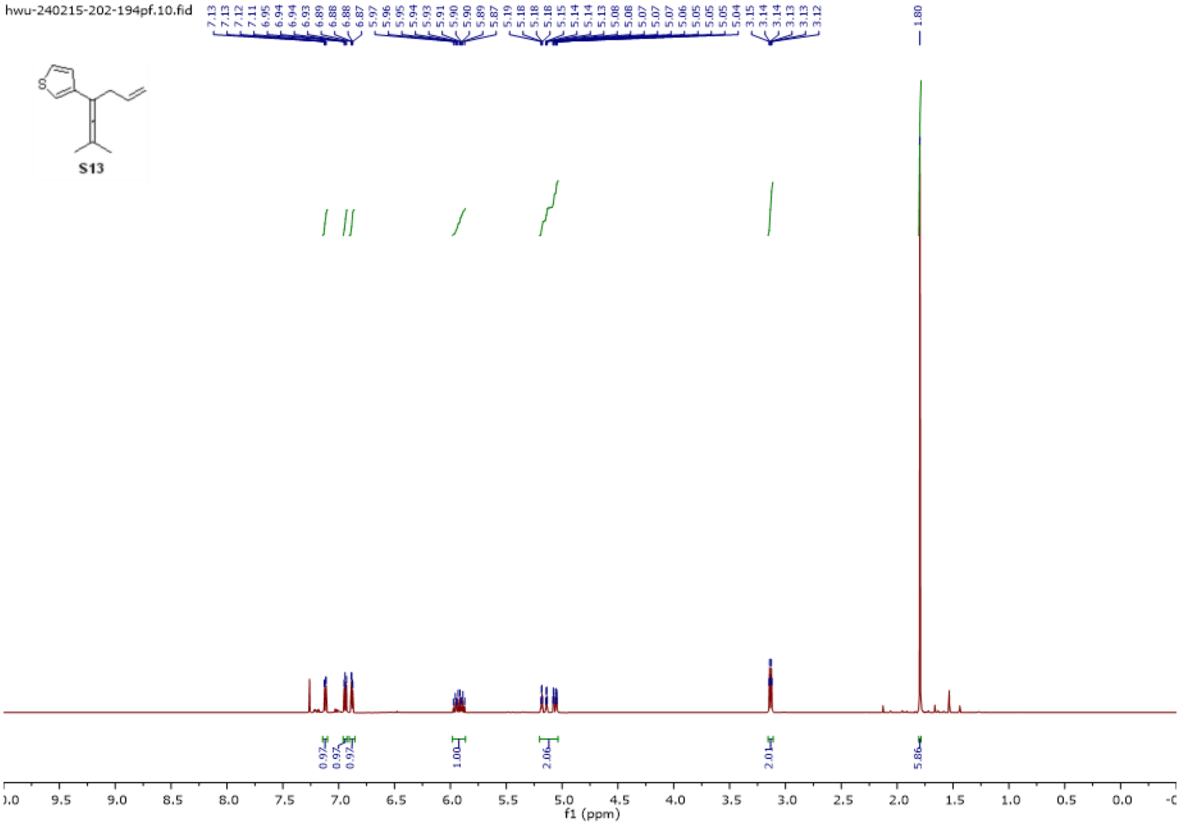
hwu-240215-202-194pf.10.fid
S13
1.80
f1 (ppm)

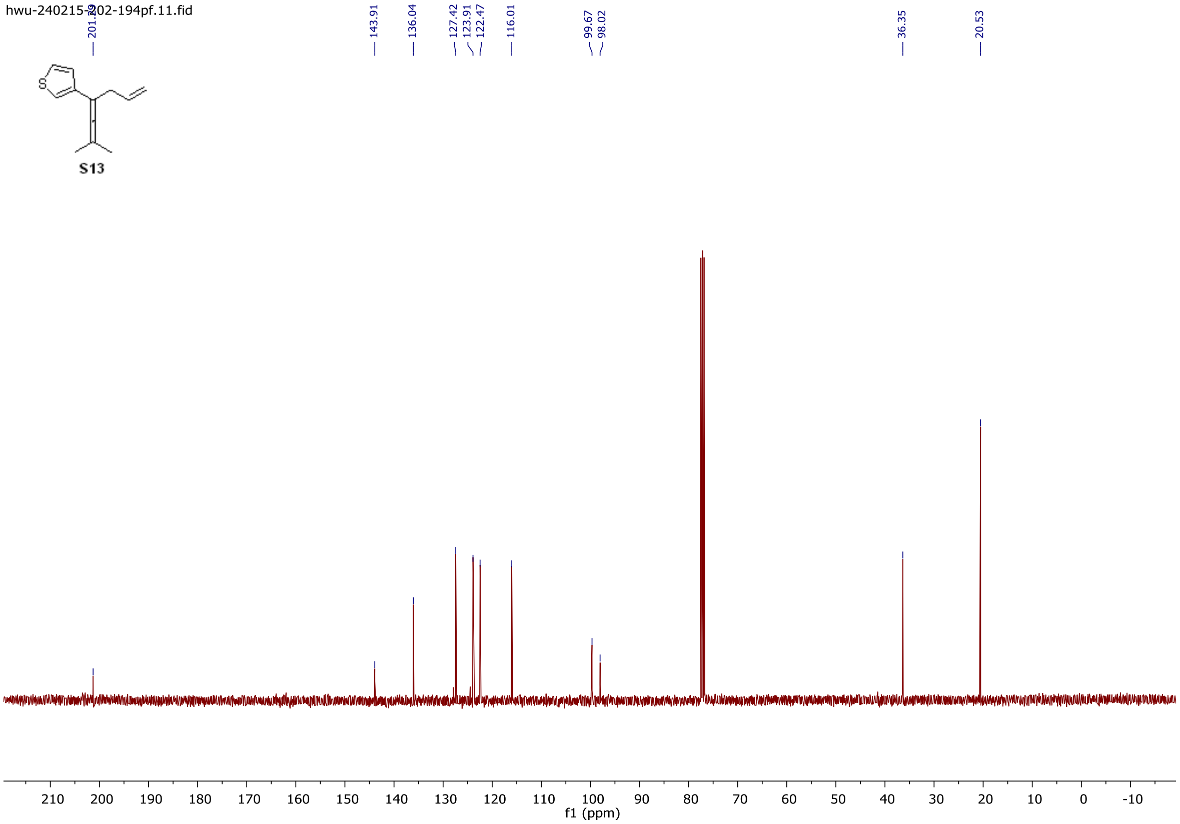
hwu-240215-202-194pf.11.fid
S13
143.91
136.04
127.42
123.91
122.47
116.01
99.67
98.02
36.35
20.53
f1 (ppm)

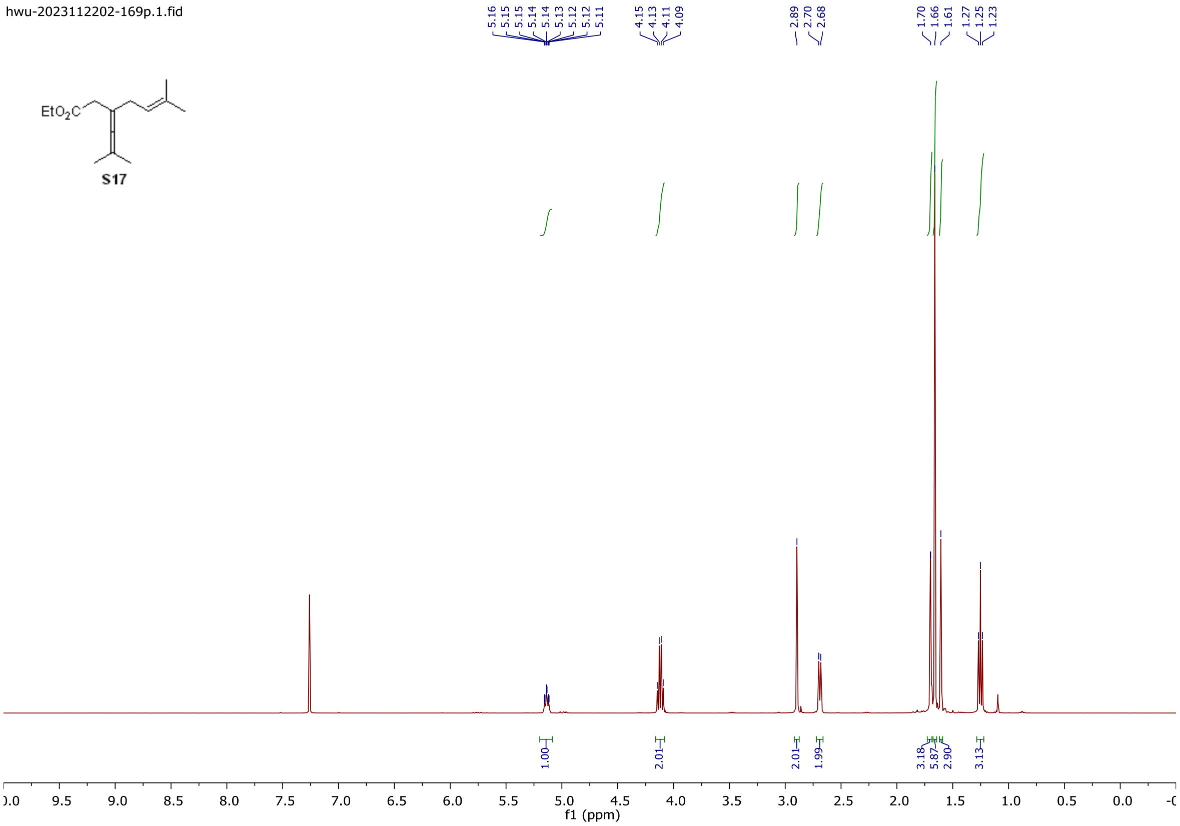
hwu-2023112202-169p.1.fid
EtO2C
S17
5.16
5.15
5.15
5.14
5.14
5.13
5.12
5.12
5.11
4.15
4.13
4.11
4.09
2.89
2.70
2.68
1.70
1.66
1.61
1.27
1.25
1.23
1.00
2.01
2.01
1.99
3.18
5.87
2.90
3.13
f1 (ppm)

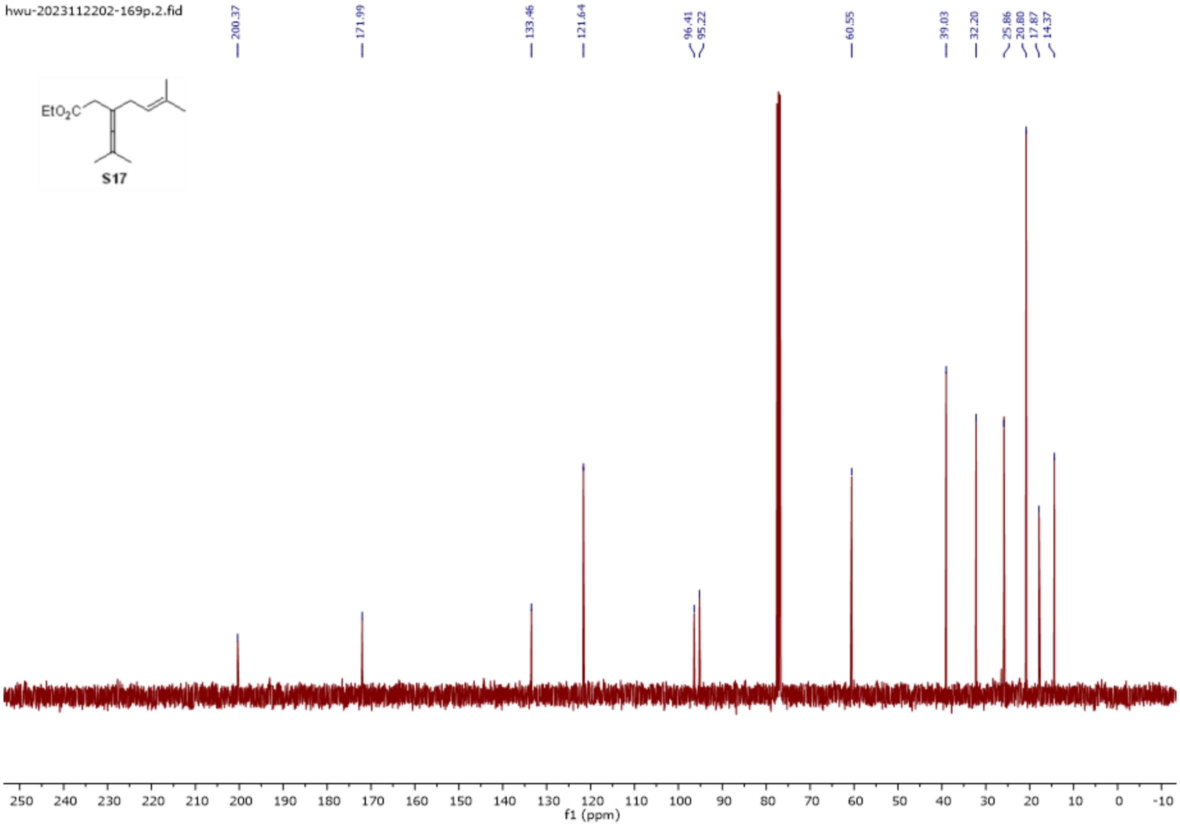
hwu-2023112202-169p.2.fid
EtO2C
S17
200.37
171.99
133.46
121.64
96.41
95.22
60.55
39.03
32.20
25.86
20.80
17.87
14.37
f1 (ppm)

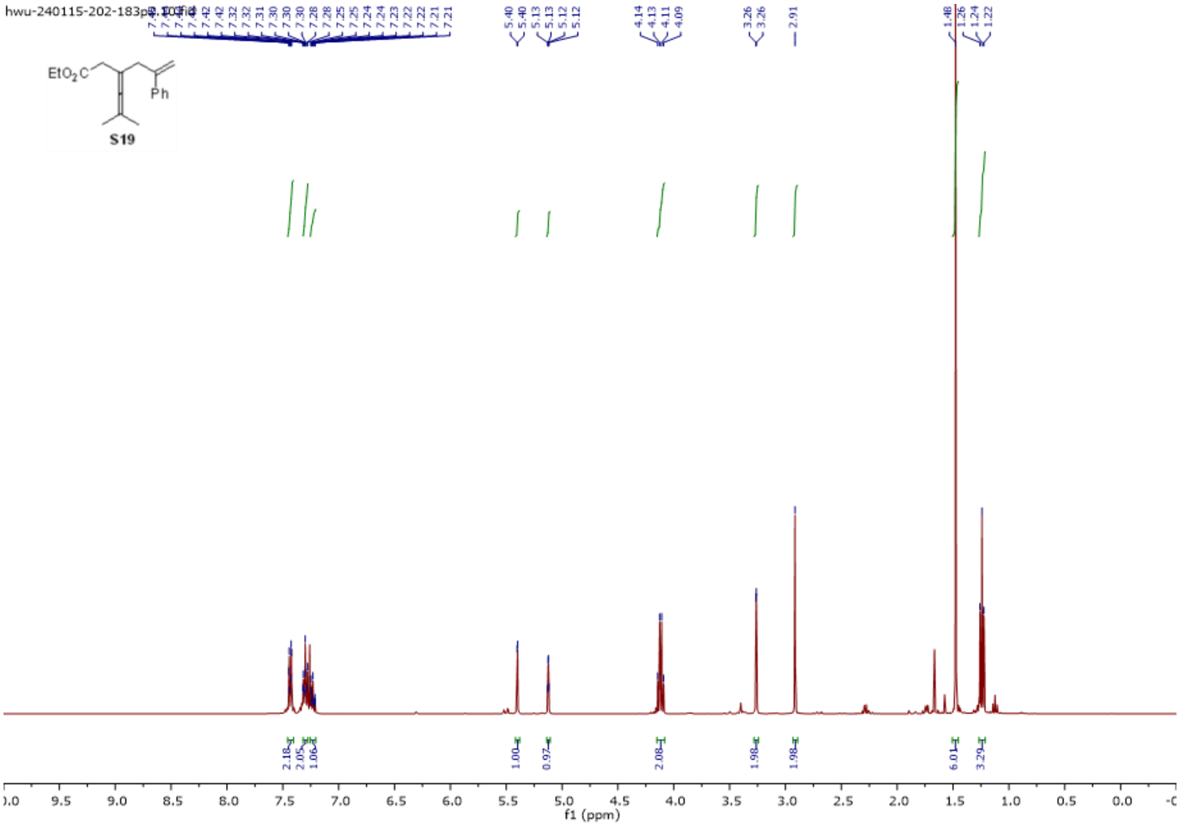

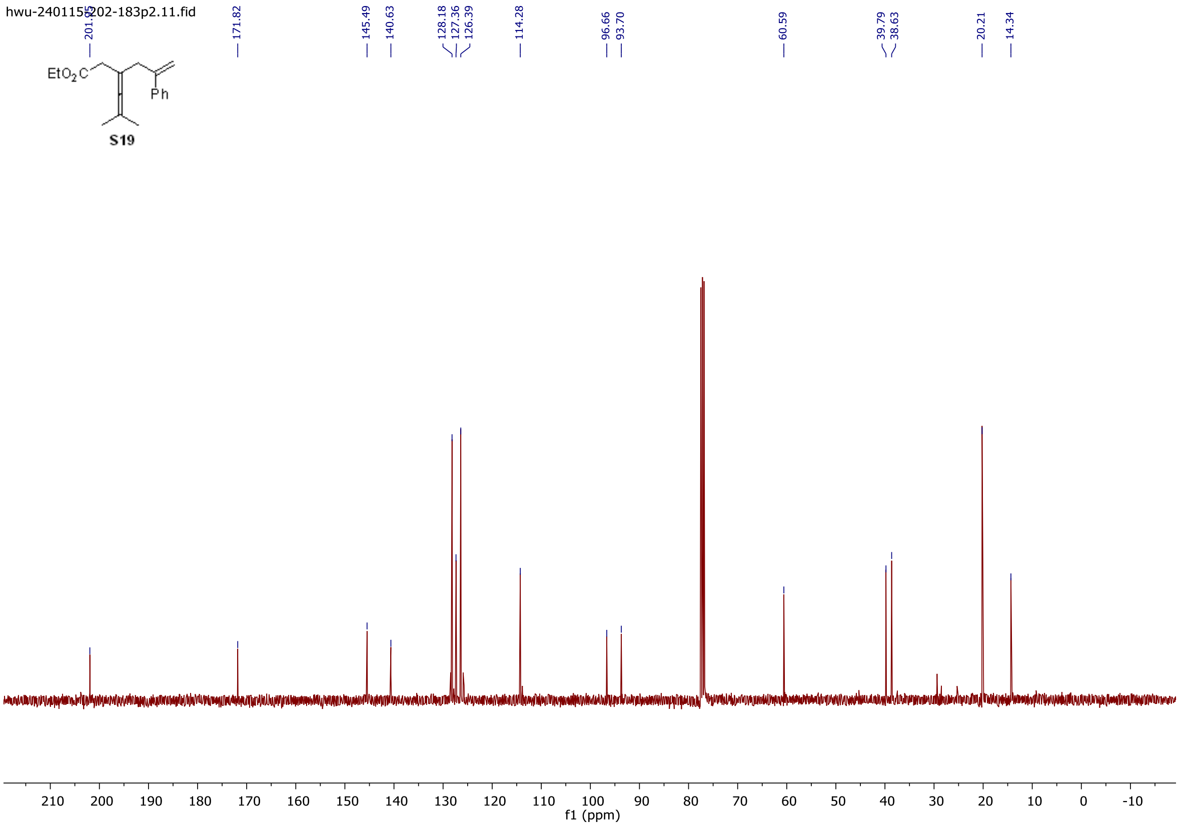

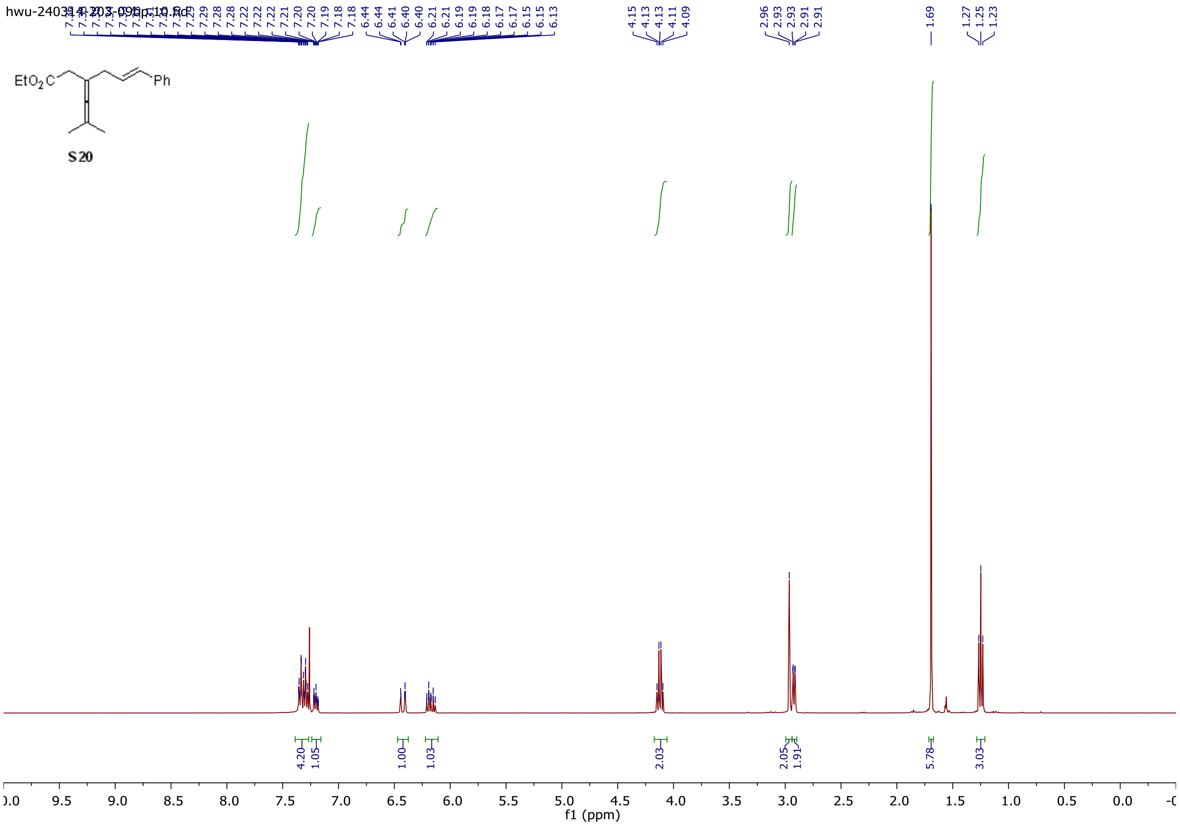
hwu-240314-203-09bp.10.fid
7.29
7.29
7.28
7.28
7.22
7.22
7.22
7.21
7.20
7.20
7.19
7.18
7.18
6.44
6.41
6.40
6.40
6.21
6.21
6.19
6.19
6.18
6.17
6.17
6.15
6.15
6.13
4.15
4.13
4.13
4.11
4.09
2.96
2.93
2.93
2.91
2.91
1.69
1.27
1.25
1.23
EtO2C
Ph
S20
4.20
1.05
1.00
1.03
2.03
2.05
1.91
5.78
3.03
f1 (ppm)

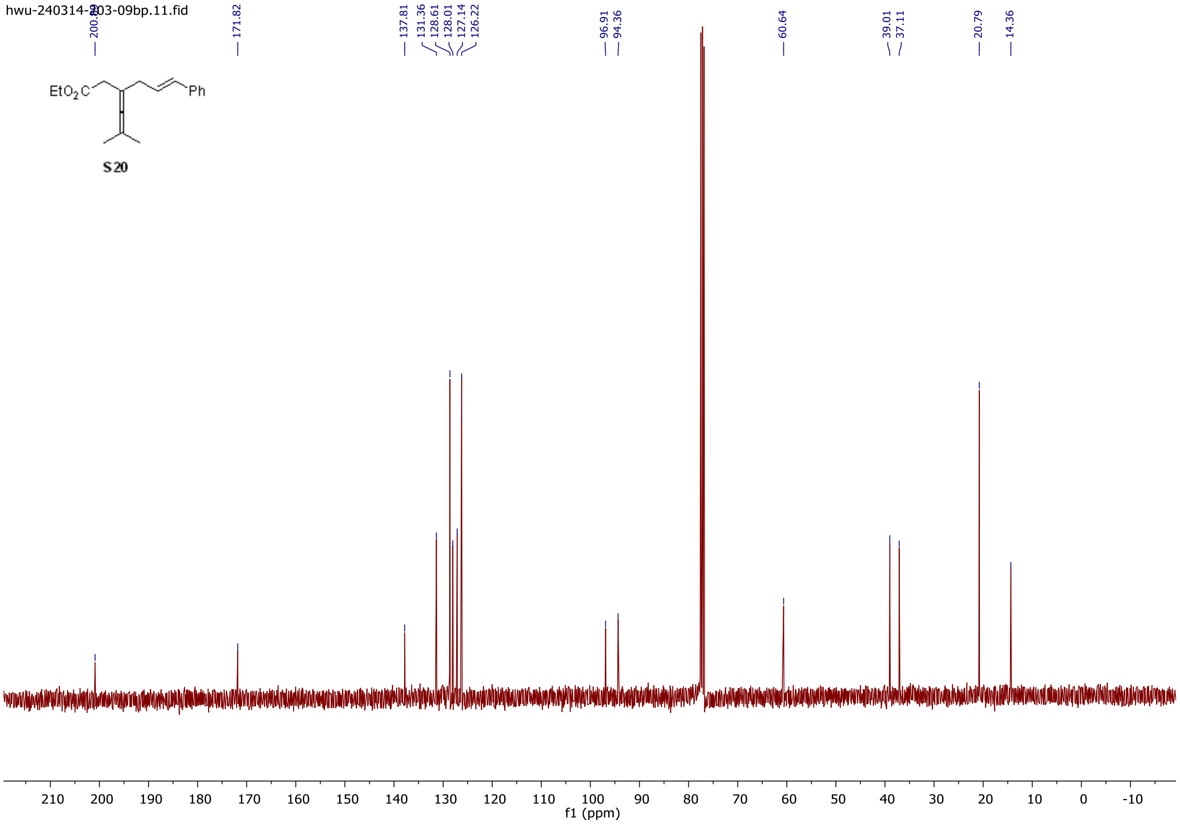
hwu-240314-203-09bp.11.fid
200.9
171.82
137.81
131.36
128.61
128.01
127.14
126.22
96.91
94.36
60.64
39.01
37.11
20.79
14.36
EtO2C
Ph
S20
f1 (ppm)

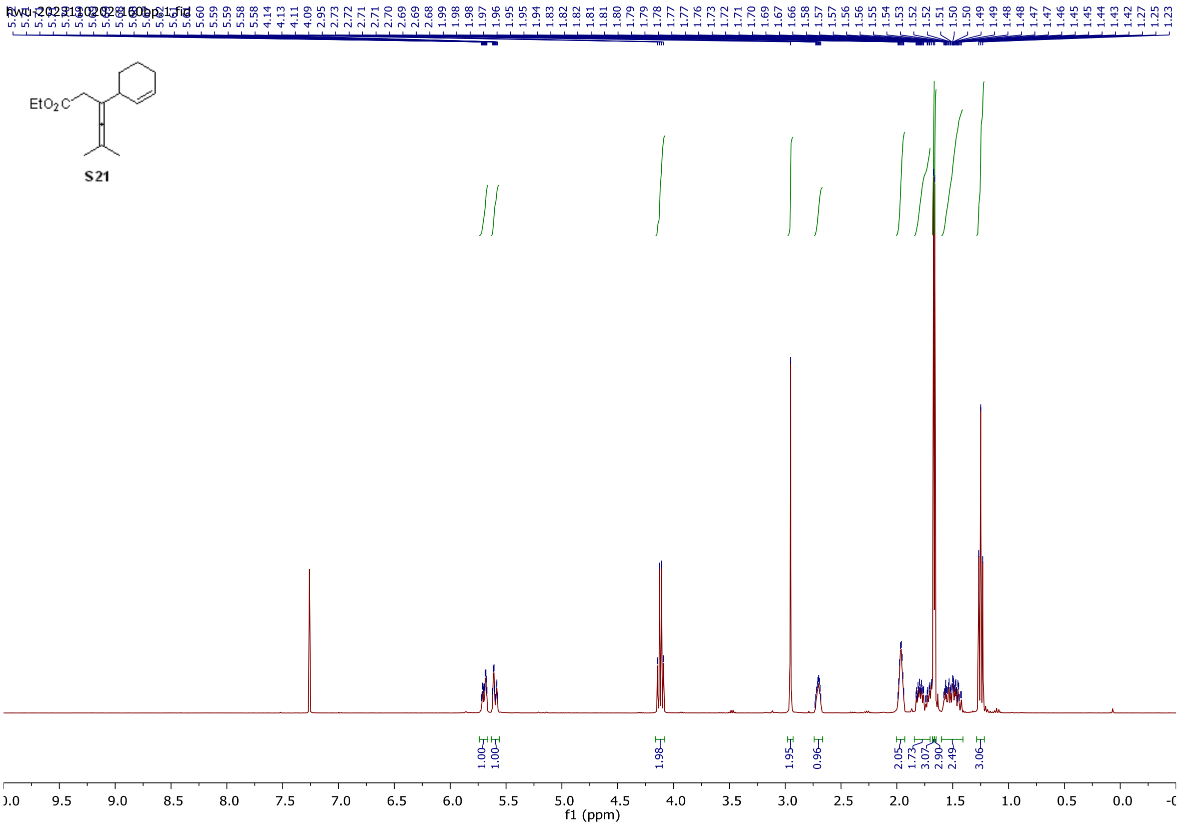
EtO2C
S21
1.00
1.00
1.98
1.95
0.96
2.05
1.73
3.07
2.90
2.49
3.06
f1 (ppm)

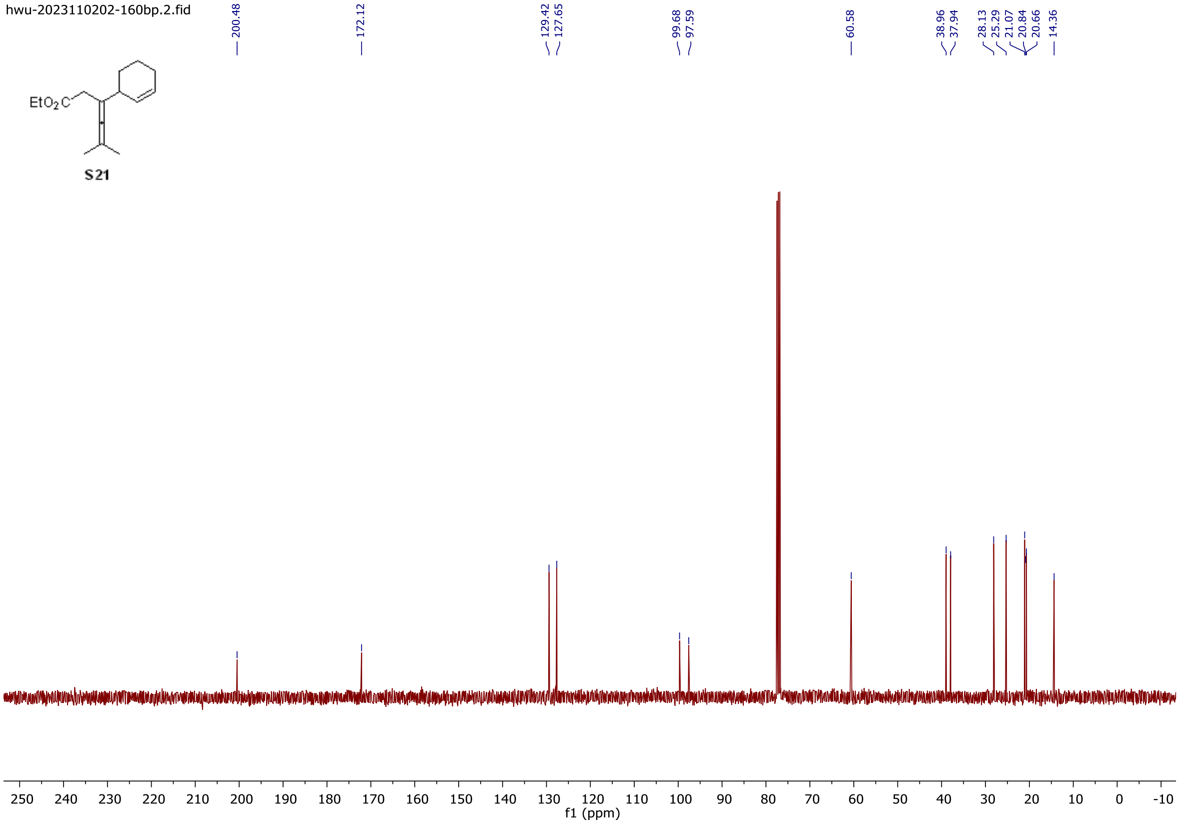
hwu-2023110202-160bp.2.fid
200.48
172.12
129.42
127.65
99.68
97.59
60.58
38.96
37.94
28.13
25.29
21.07
20.84
20.66
14.36
EtO2C
S21
f1 (ppm)

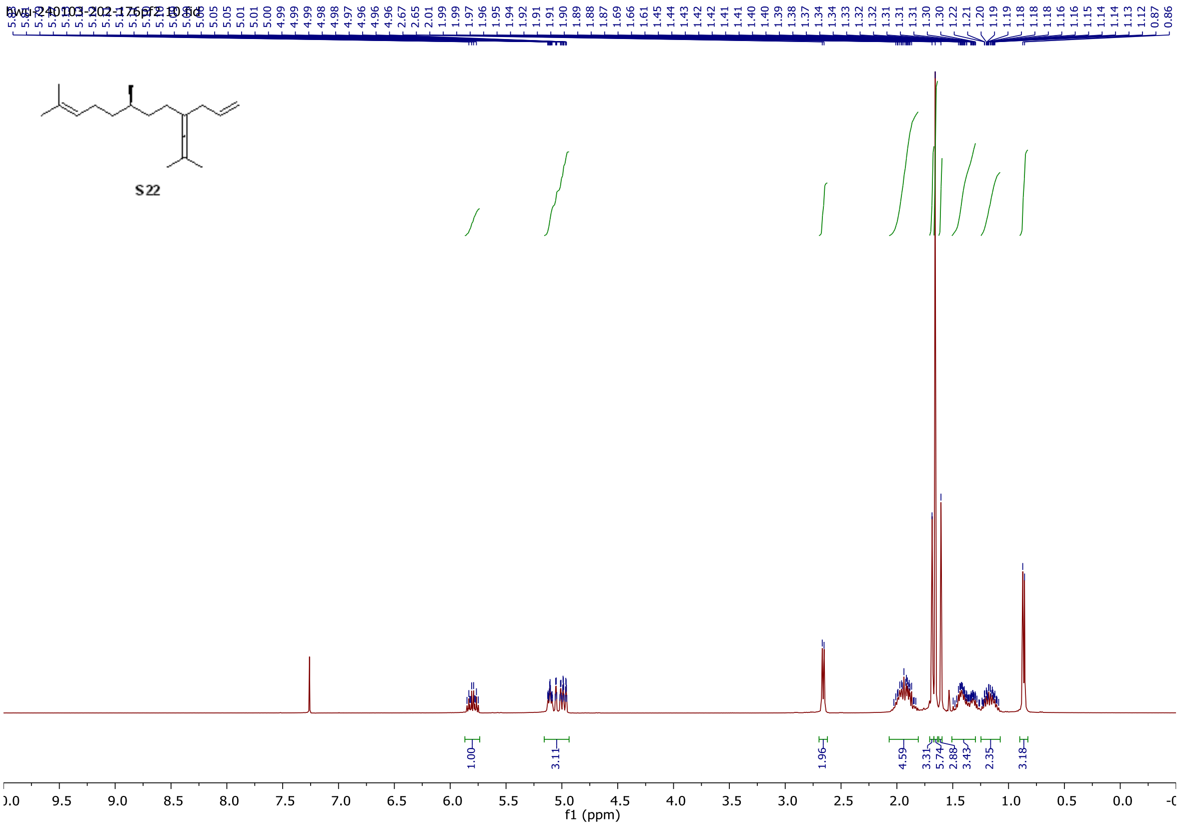
5.05 5.05 5.01 5.00 4.99 4.99 4.99 4.98 4.98 4.97 4.96 4.96 4.96 2.67 2.65 2.01 1.99 1.99 1.97 1.96 1.95 1.94 1.92 1.91 1.91 1.90 1.89 1.88 1.87 1.69 1.66 1.61 1.45 1.44 1.43 1.42 1.42 1.41 1.41 1.40 1.40 1.39 1.38 1.37 1.34 1.34 1.33 1.32 1.32 1.31 1.31 1.31 1.30 1.30 1.22 1.21 1.20 1.19 1.19 1.18 1.18 1.18 1.16 1.16 1.15 1.14 1.14 1.13 1.12 0.87 0.86
S22
1.00
3.11
1.96
4.59
3.31
5.74
2.88
3.43
2.35
3.18
f1 (ppm)

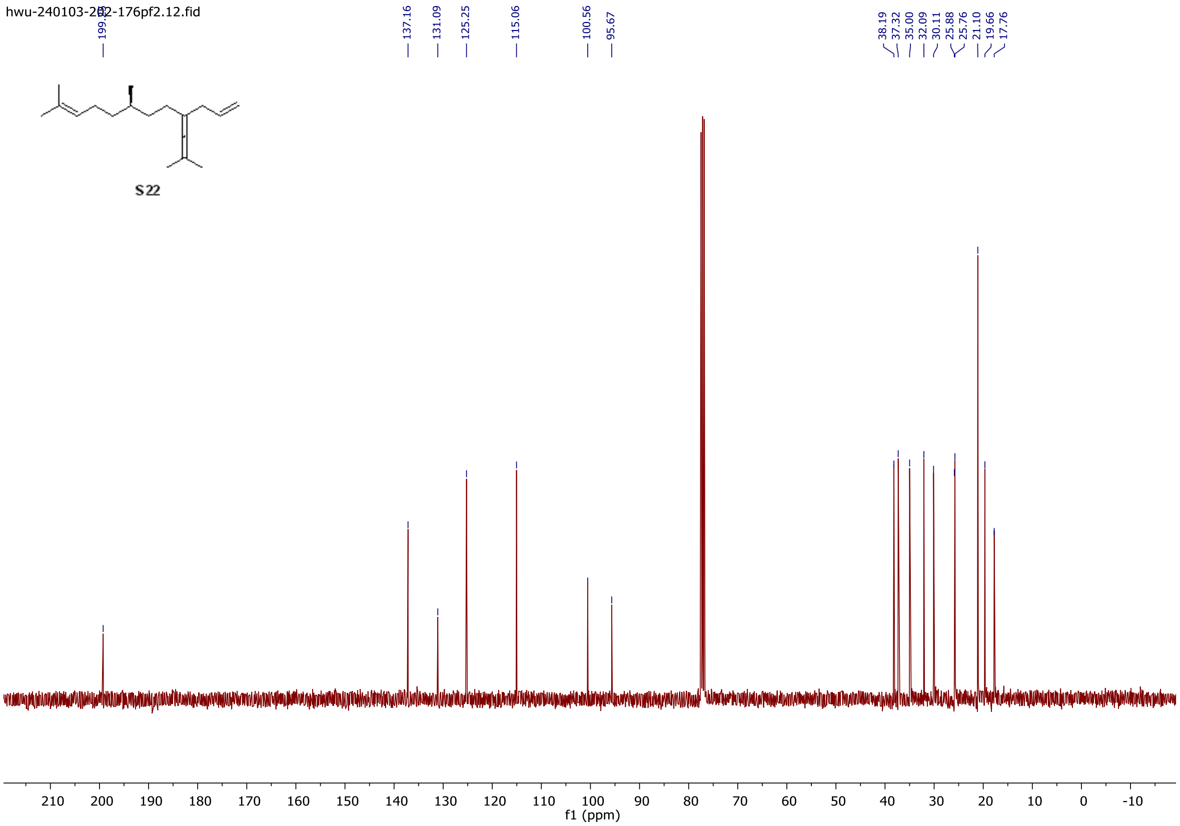
hwu-240103-202-176pf2.12.fid
199.
137.16
131.09
125.25
115.06
100.56
95.67
38.19
37.32
35.00
32.09
30.11
25.88
25.76
21.10
19.66
17.76
S22
f1 (ppm)

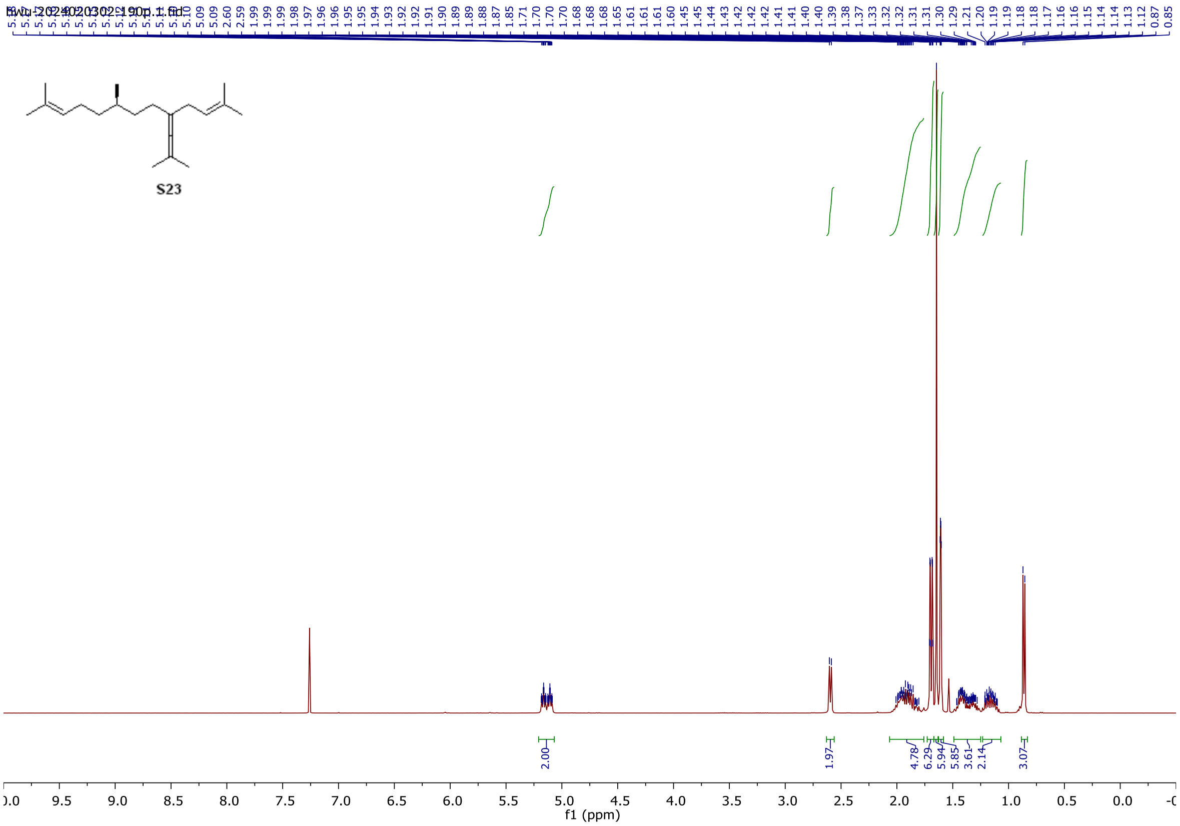
hwu-2024020302-190p.1.fid
S23
2.00
1.97
4.78
6.29
5.94
5.85
3.61
2.14
3.07
f1 (ppm)

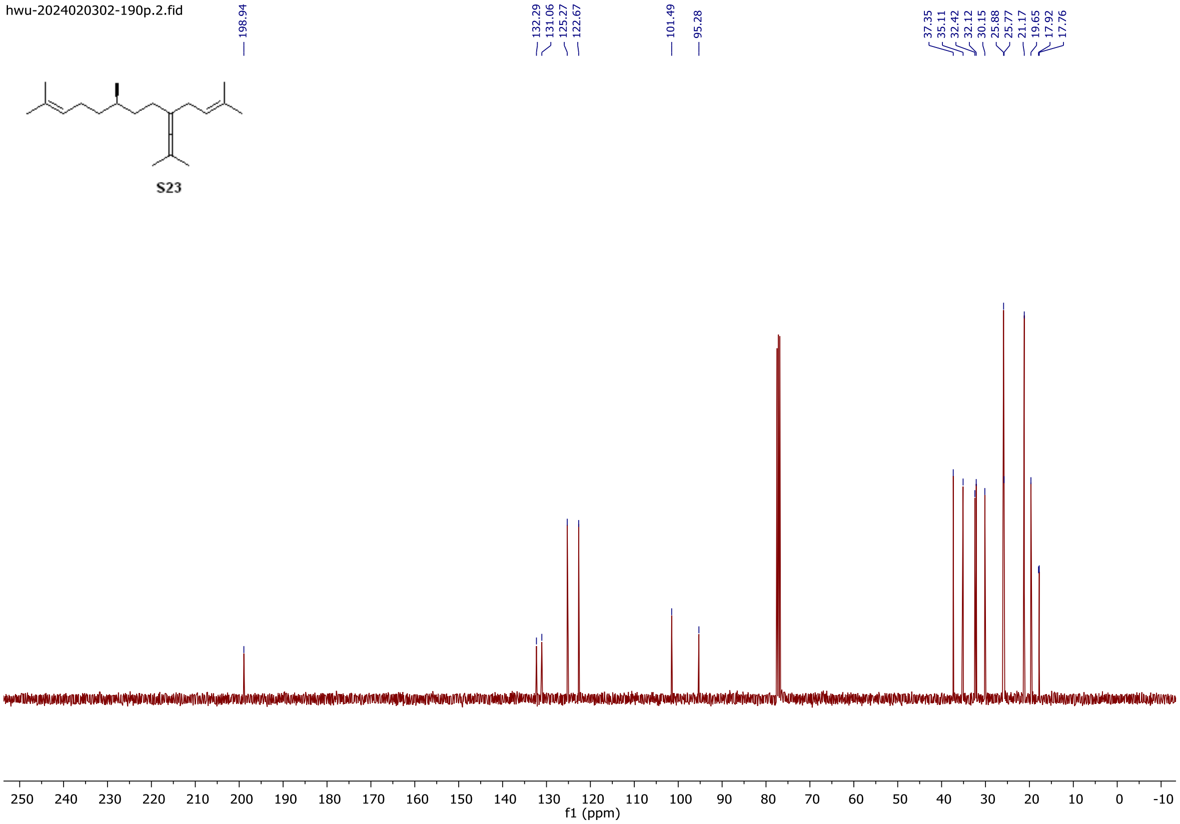
hwu-2024020302-190p.2.fid
198.94
132.29
131.06
125.27
122.67
101.49
95.28
37.35
35.11
32.42
32.12
30.15
25.88
25.77
21.17
19.65
17.92
17.76
S23
f1 (ppm)

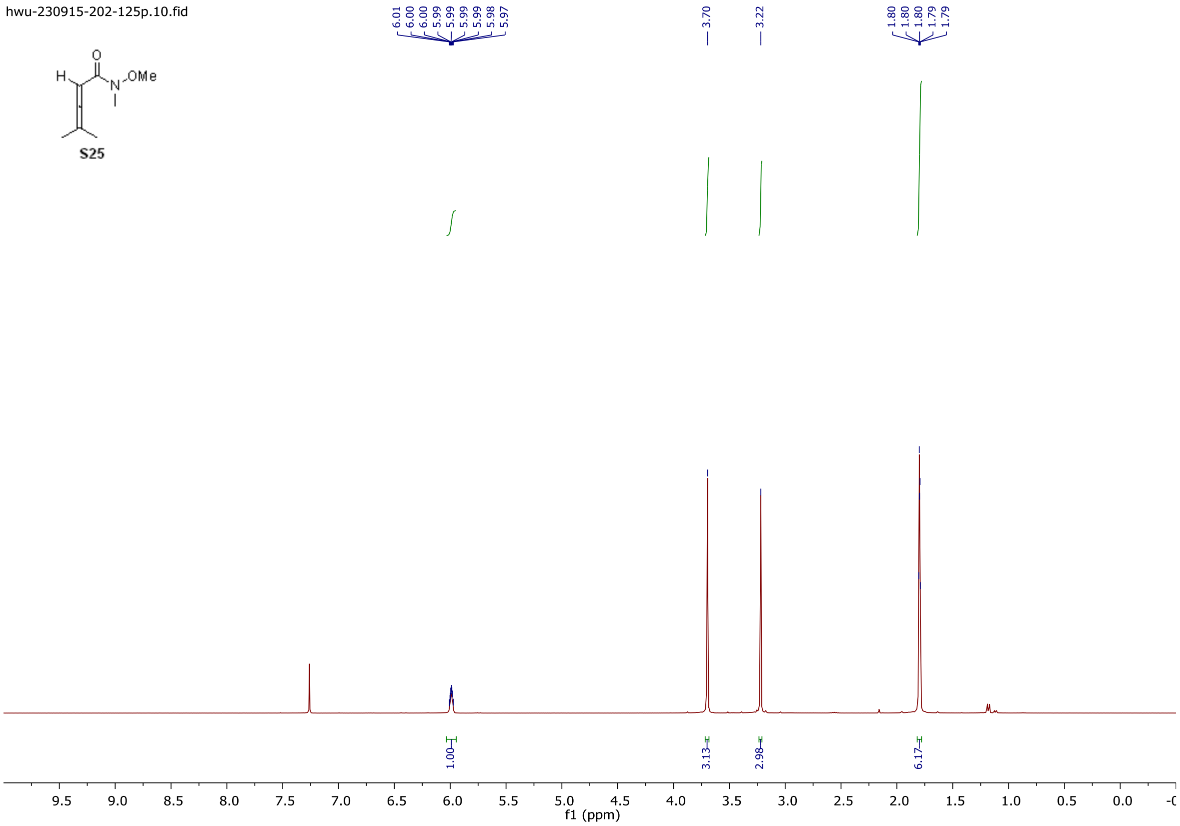
hwu-230915-202-125p.10.fid
O
H
N
OMe
S25
6.01
6.00
6.00
5.99
5.99
5.99
5.99
5.98
5.97
3.70
3.22
1.80
1.80
1.80
1.79
1.79
1.00
3.13
2.98
6.17
9.5
9.0
8.5
8.0
7.5
7.0
6.5
6.0
5.5
5.0
4.5
4.0
3.5
3.0
2.5
2.0
1.5
1.0
0.5
0.0
f1 (ppm)

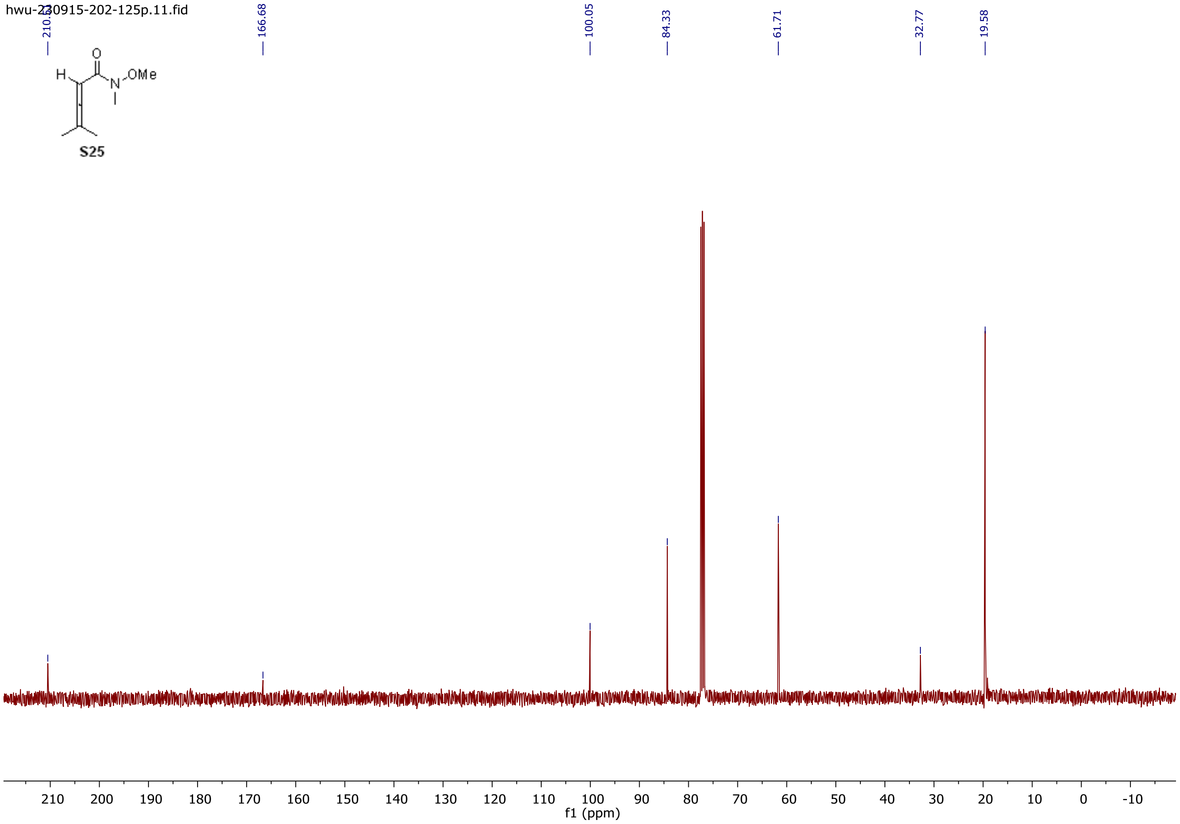
hwu-230915-202-125p.11.fid
166.68
100.05
84.33
61.71
32.77
19.58
O
H
N
OMe
S25
210
200
190
180
170
160
150
140
130
120
110
100
90
80
70
60
50
40
30
20
10
0
-10
f1 (ppm)

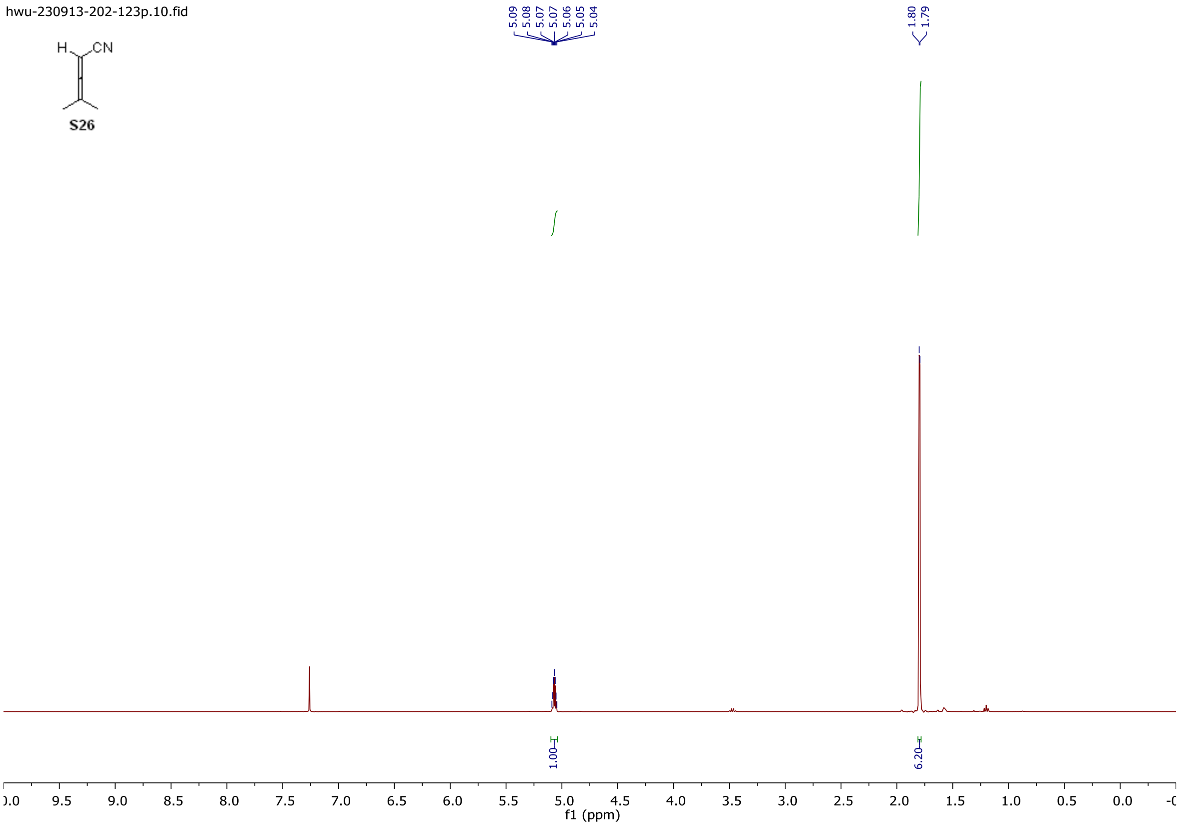
hwu-230913-202-123p.10.fid
H
CN
S26
5.09
5.08
5.07
5.07
5.06
5.05
5.04
1.80
1.79
1.00
6.20
f1 (ppm)

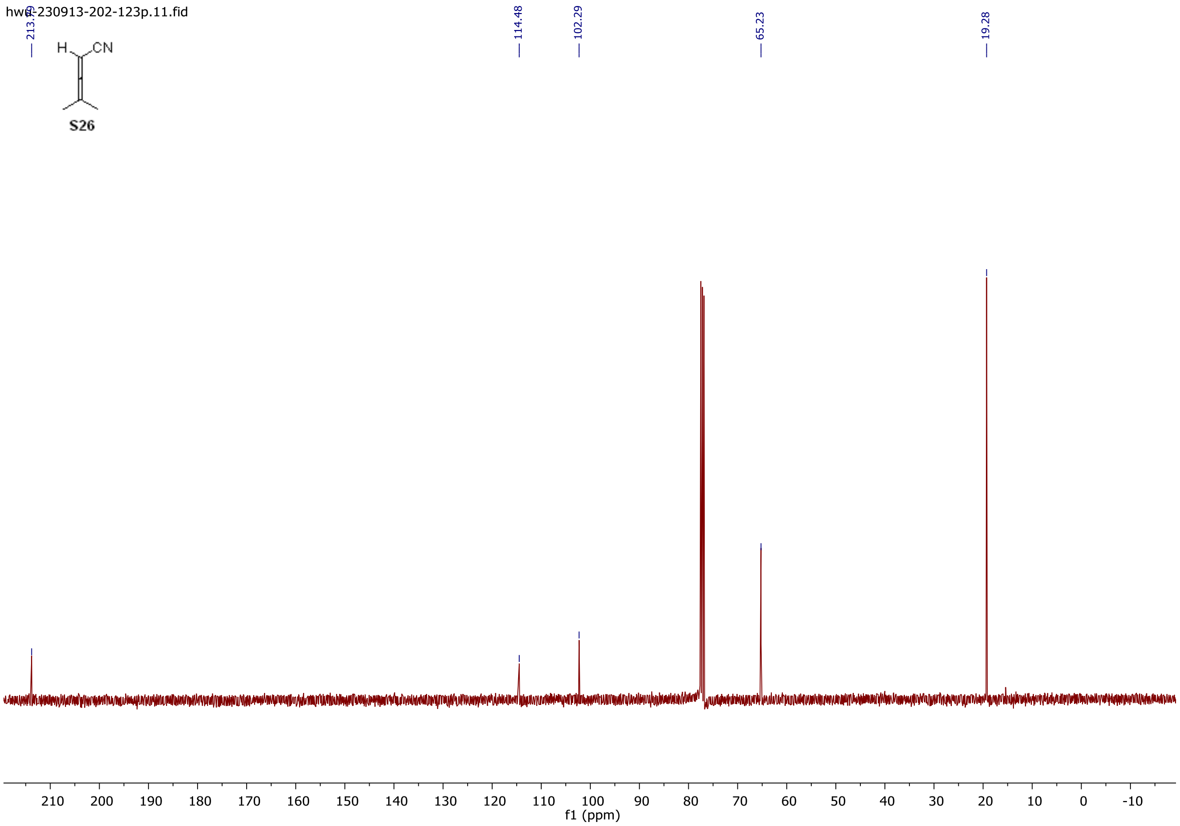
hwu-230913-202-123p.11.fid
H
CN
S26
114.48
102.29
65.23
19.28
f1 (ppm)

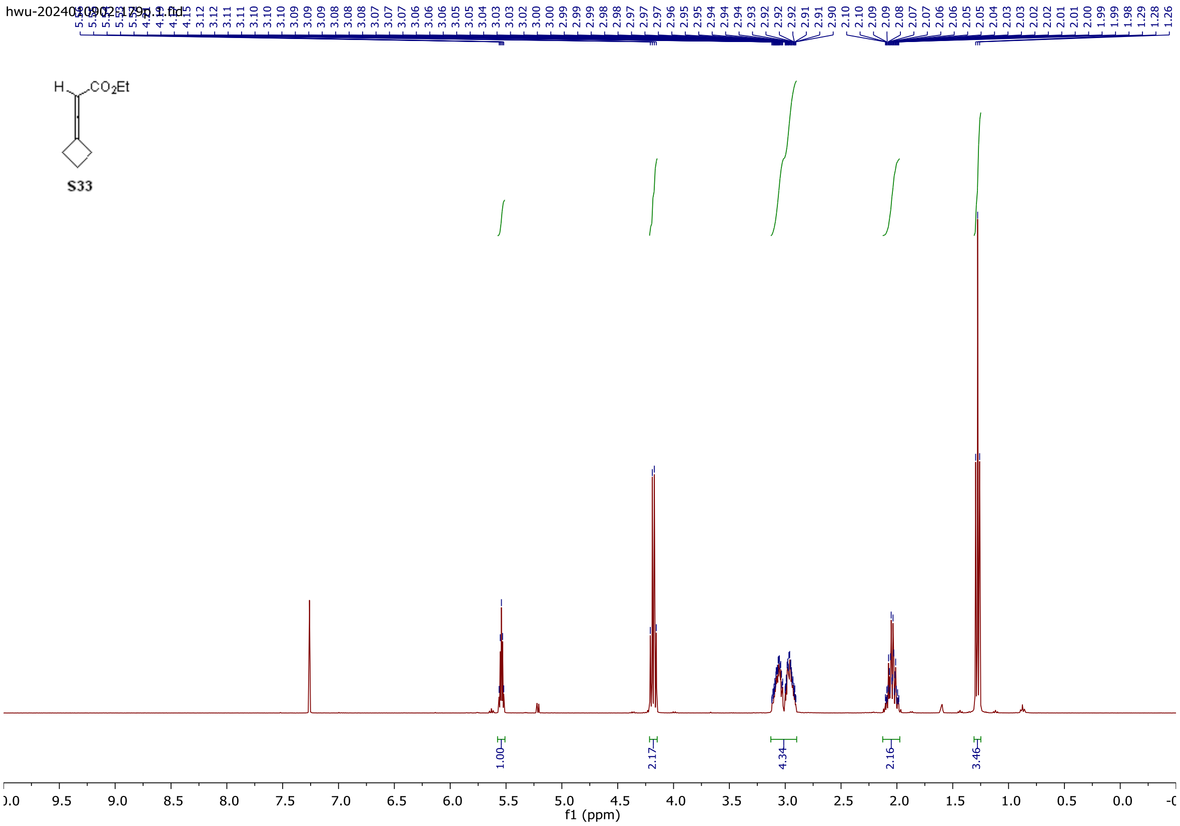
hwu-2024010902-179p.1.fid
CO2Et
H
S33
1.00
2.17
4.34
2.16
3.46
f1 (ppm)

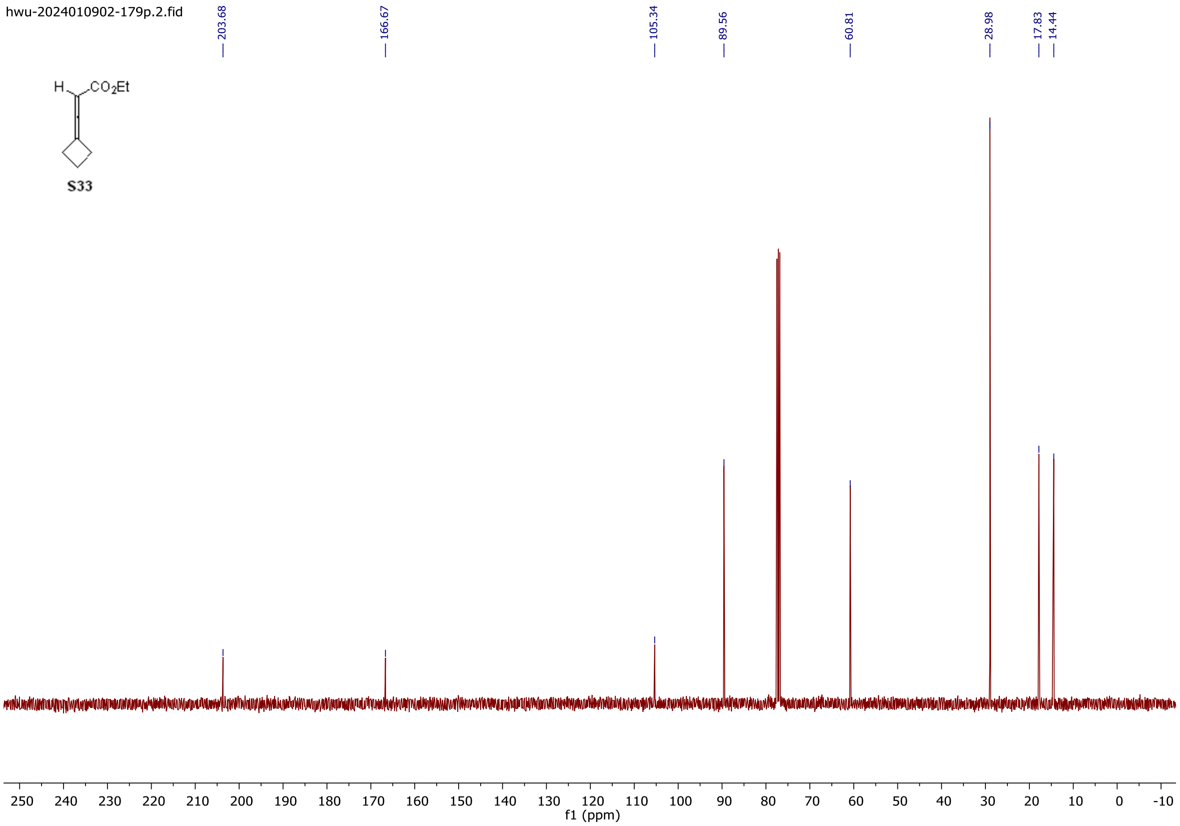
hwu-2024010902-179p.2.fid
203.68
166.67
105.34
89.56
60.81
28.98
17.83
14.44
CO2Et
H
S33
f1 (ppm)

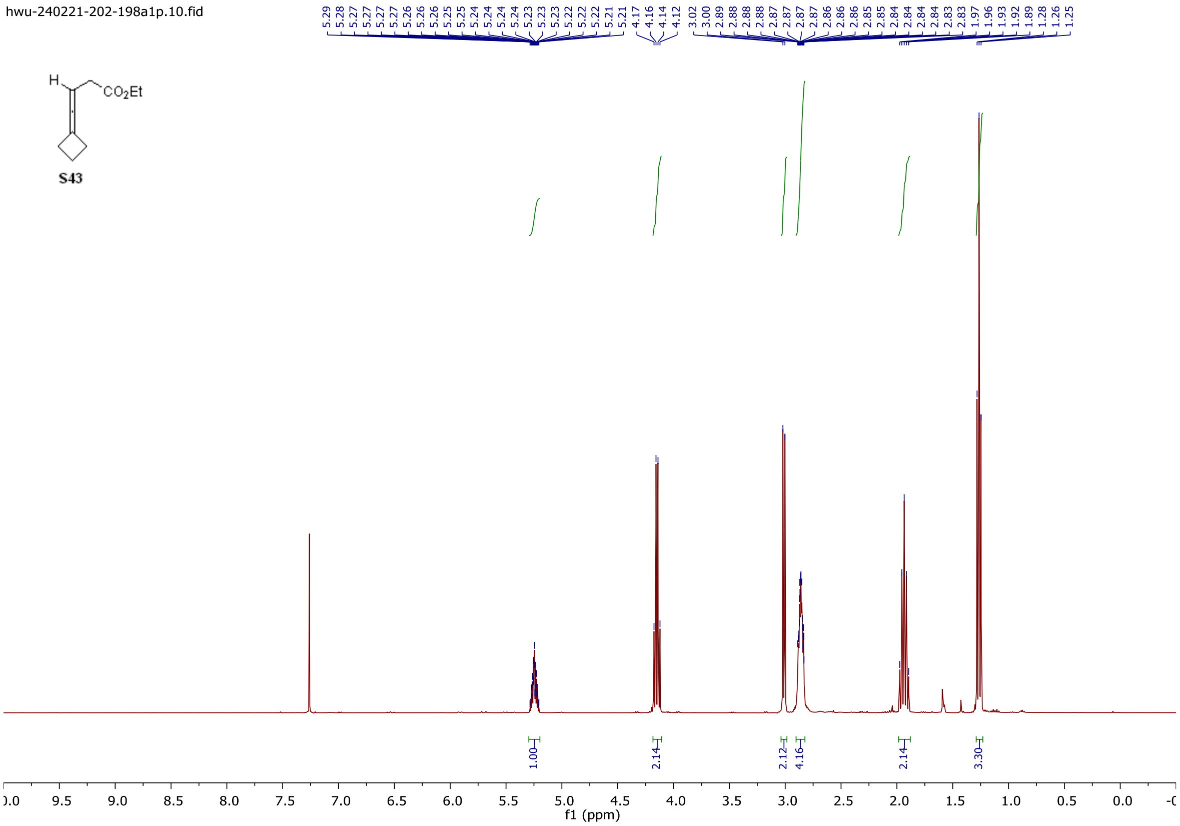
hwu-240221-202-198a1p.10.fid
CO2Et
S43
f1 (ppm)

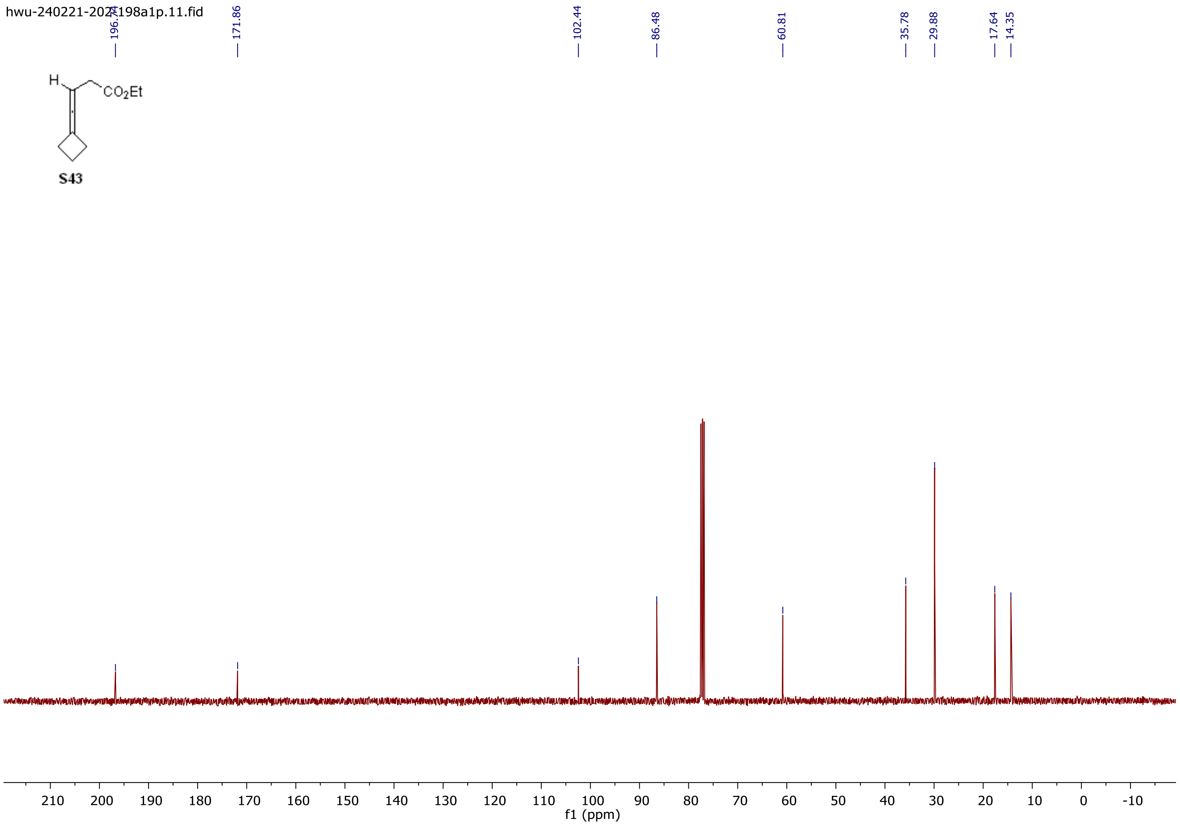
hwu-240221-202-198a1p.11.fid
196.74
171.86
102.44
86.48
60.81
35.78
29.88
17.64
14.35
CO2Et
S43
f1 (ppm)

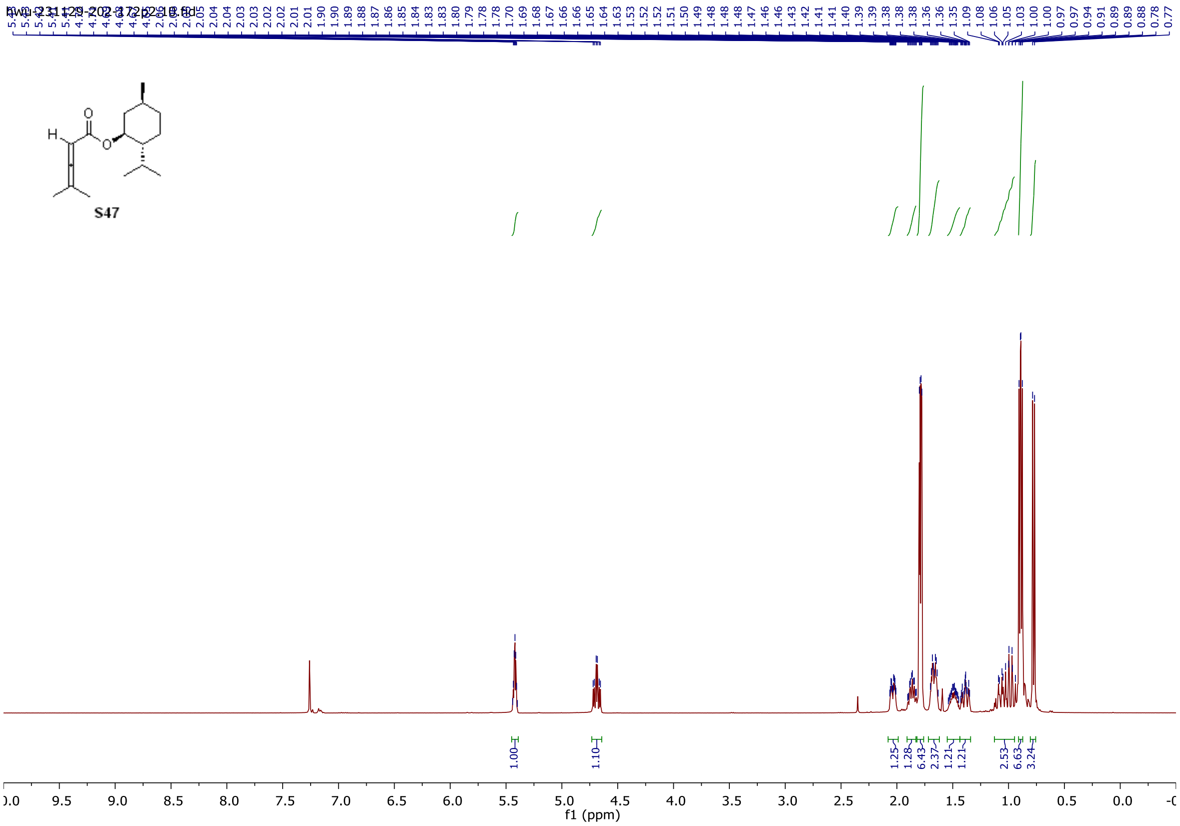

S47
1.00
1.10
1.25
1.28
6.43
2.37
1.21
1.21
2.53
6.63
3.24
f1 (ppm)

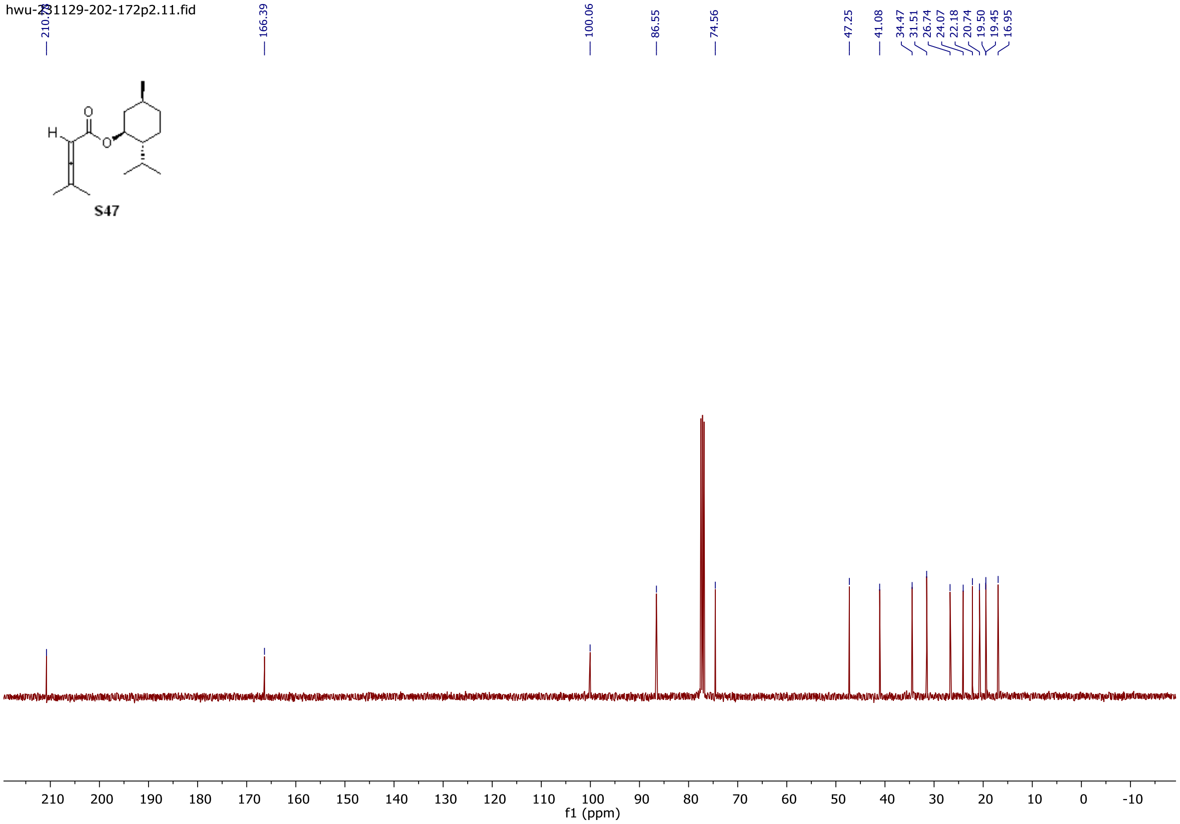

hwu-231129-202-172p2.11.fid
166.39
100.06
86.55
74.56
47.25
41.08
34.47
31.51
26.74
24.07
22.18
20.74
19.50
19.45
16.95
S47
f1 (ppm)

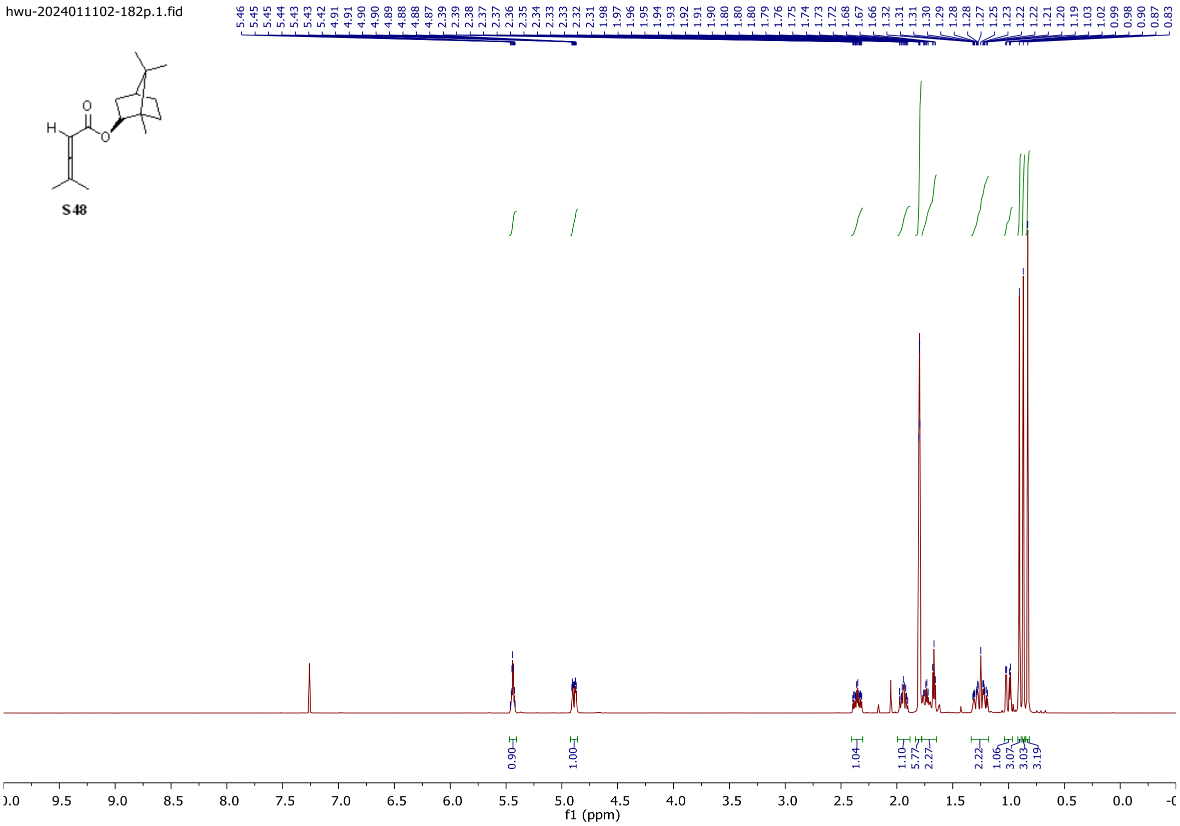
hwu-2024011102-182p.1.fid
5.46 5.45 5.45 5.44 5.43 5.43 5.42 4.91 4.91 4.90 4.90 4.89 4.88 4.88 4.87 2.39 2.39 2.38 2.37 2.37 2.36 2.35 2.34 2.33 2.33 2.32 2.31 1.98 1.97 1.96 1.95 1.94 1.93 1.92 1.91 1.90 1.80 1.80 1.80 1.79 1.76 1.75 1.74 1.73 1.72 1.68 1.67 1.66 1.32 1.31 1.31 1.30 1.29 1.28 1.28 1.27 1.25 1.23 1.22 1.22 1.21 1.20 1.19 1.03 1.02 0.99 0.98 0.90 0.87 0.83
H
O
O
S48
0.90
1.00
1.04
1.10
5.77
2.27
2.22
1.06
3.07
3.03
3.19
f1 (ppm)

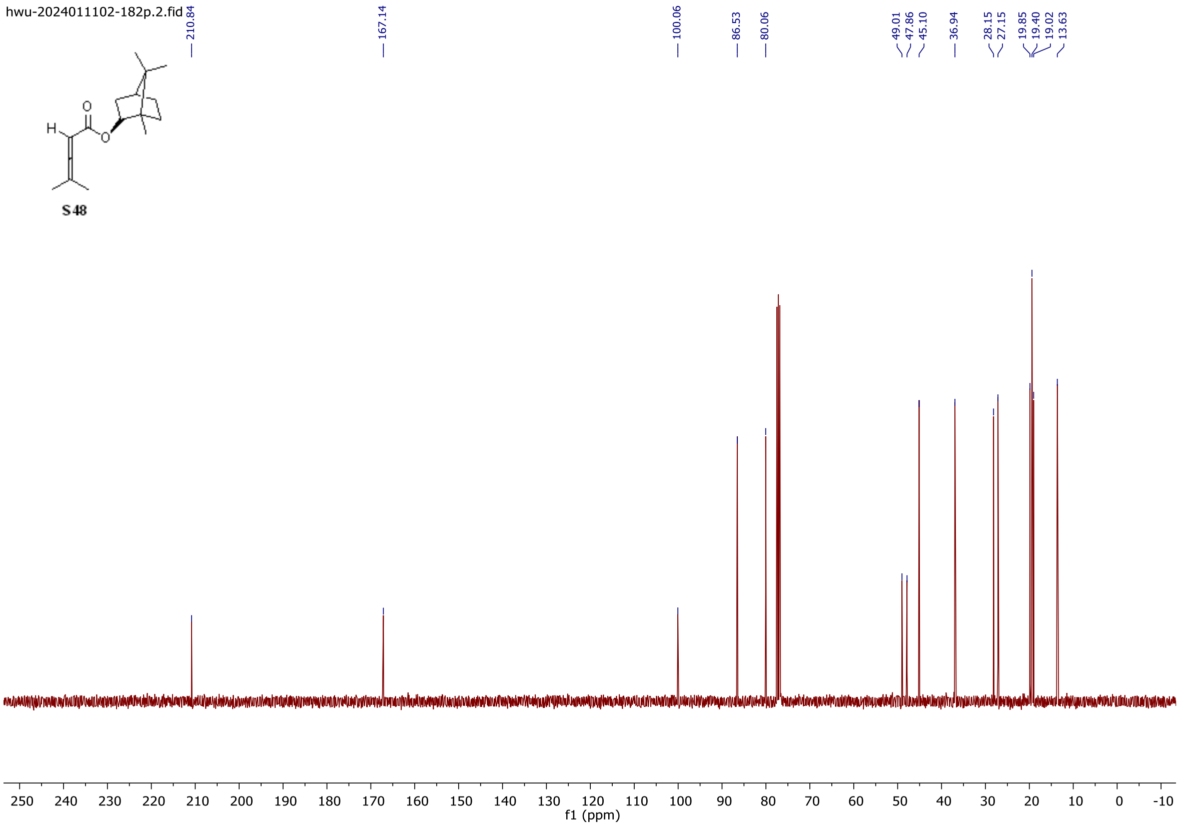
hwu-2024011102-182p.2.fid
210.84
167.14
100.06
86.53
80.06
49.01 47.86 45.10
36.94
28.15 27.15
19.85 19.40 19.02 13.63
H
O
O
S48
f1 (ppm)

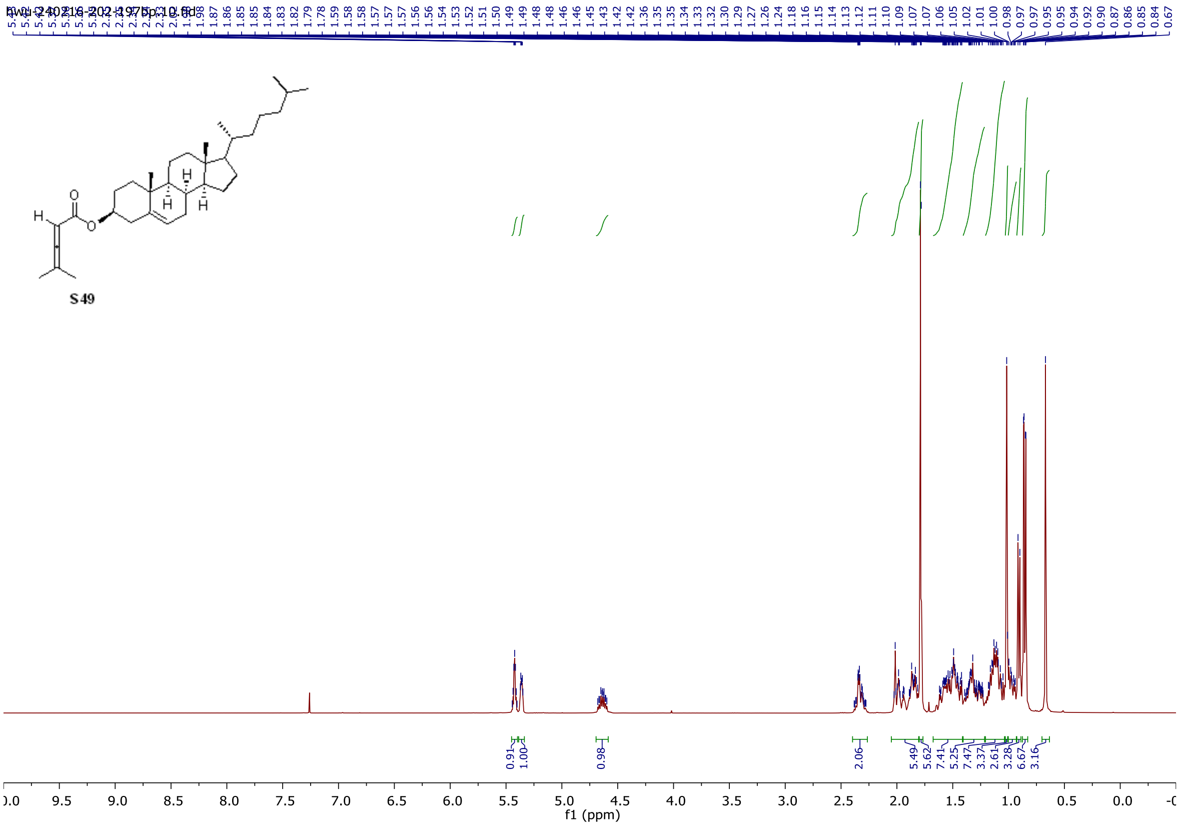
hwu-240216-202-197bp.10.fid
S49
f1 (ppm)

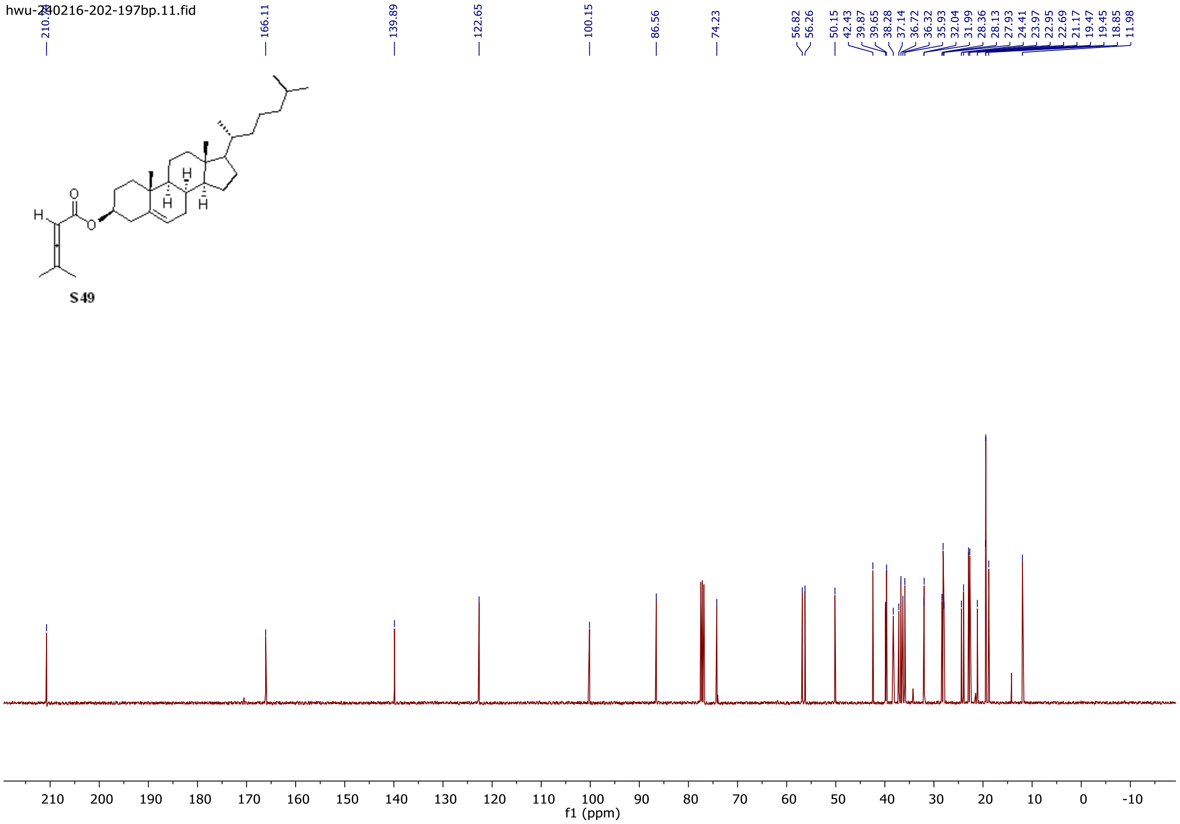
hwu-240216-202-197bp.11.fid
S49
f1 (ppm)

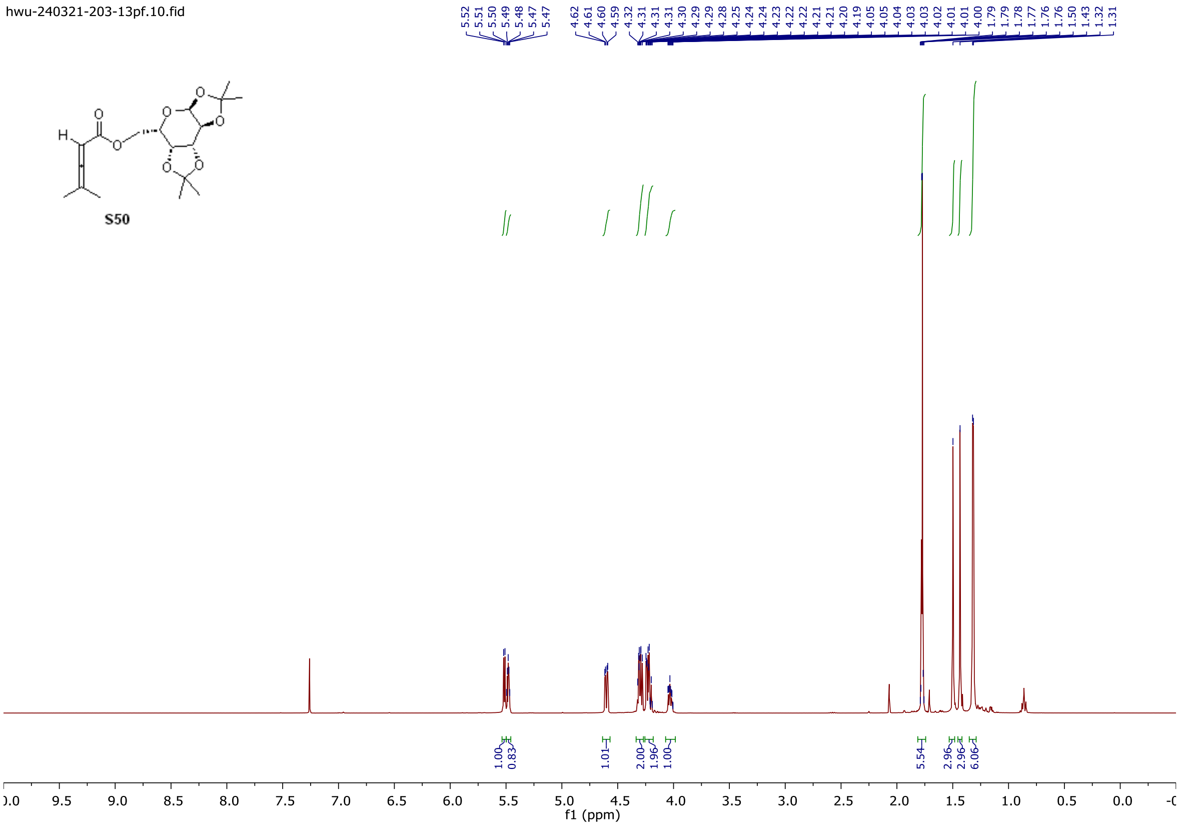
hwu-240321-203-13pf.10.fid
5.52 5.51 5.50 5.49 5.48 5.47 5.47
4.62 4.61 4.60 4.59 4.32 4.31 4.31 4.31 4.30 4.29 4.29 4.28 4.25 4.24 4.24 4.23 4.22 4.22 4.21 4.21 4.20 4.19 4.05 4.05 4.04 4.03 4.03 4.02 4.01 4.01 4.00
1.79 1.79 1.78 1.77 1.76 1.76 1.50 1.43 1.32 1.31
S50
1.00 0.83 1.01 2.00 1.96 1.00 5.54 2.96 2.96 6.06
f1 (ppm)

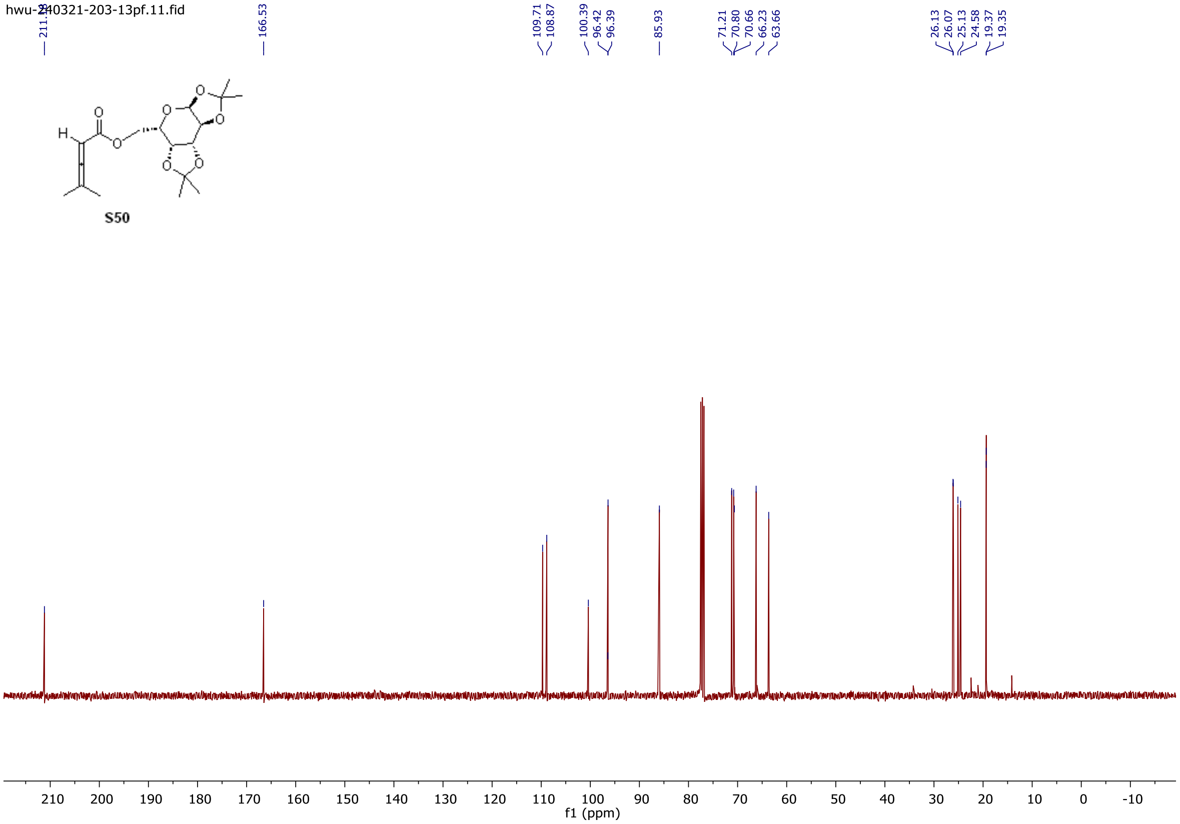
hwu-240321-203-13pf.11.fid
166.53
109.71 108.87 100.39 96.42 96.39 85.93
71.21 70.80 70.66 66.23 63.66
26.13 26.07 25.13 24.58 19.37 19.35
S50
f1 (ppm)

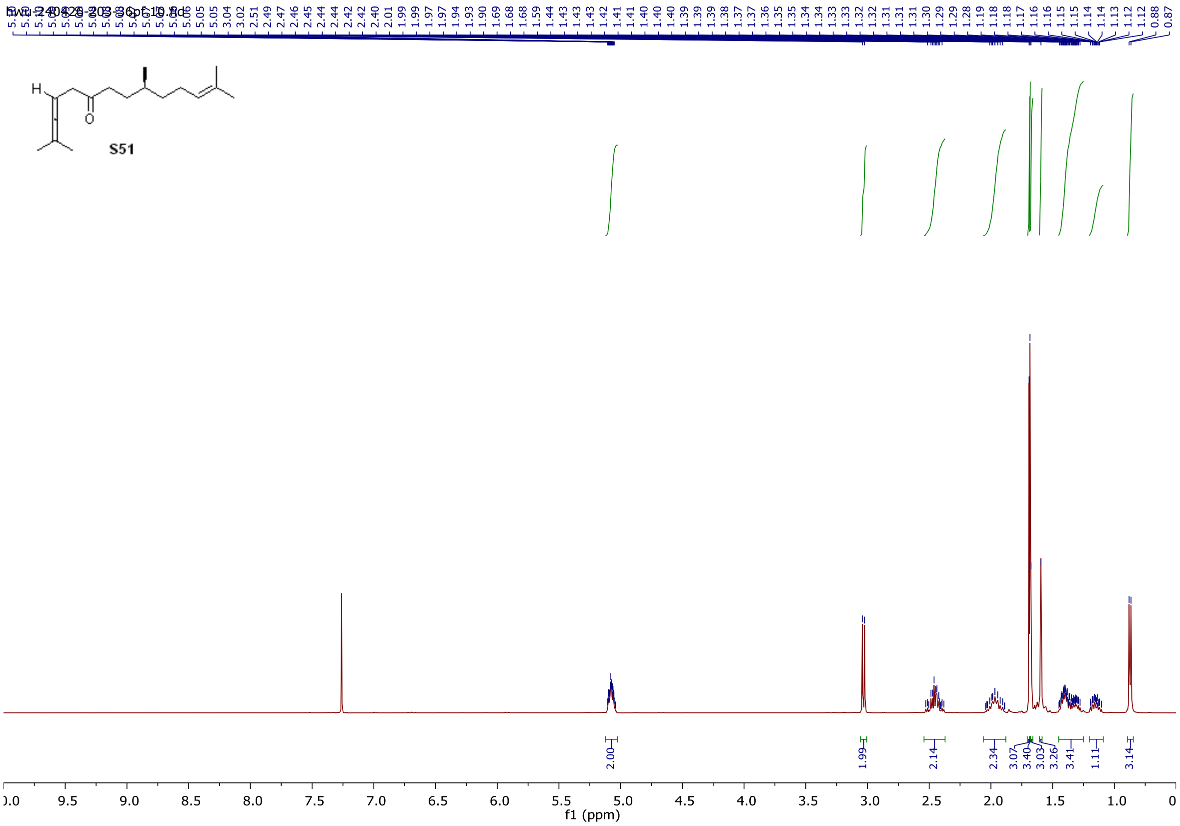
S51
f1 (ppm)

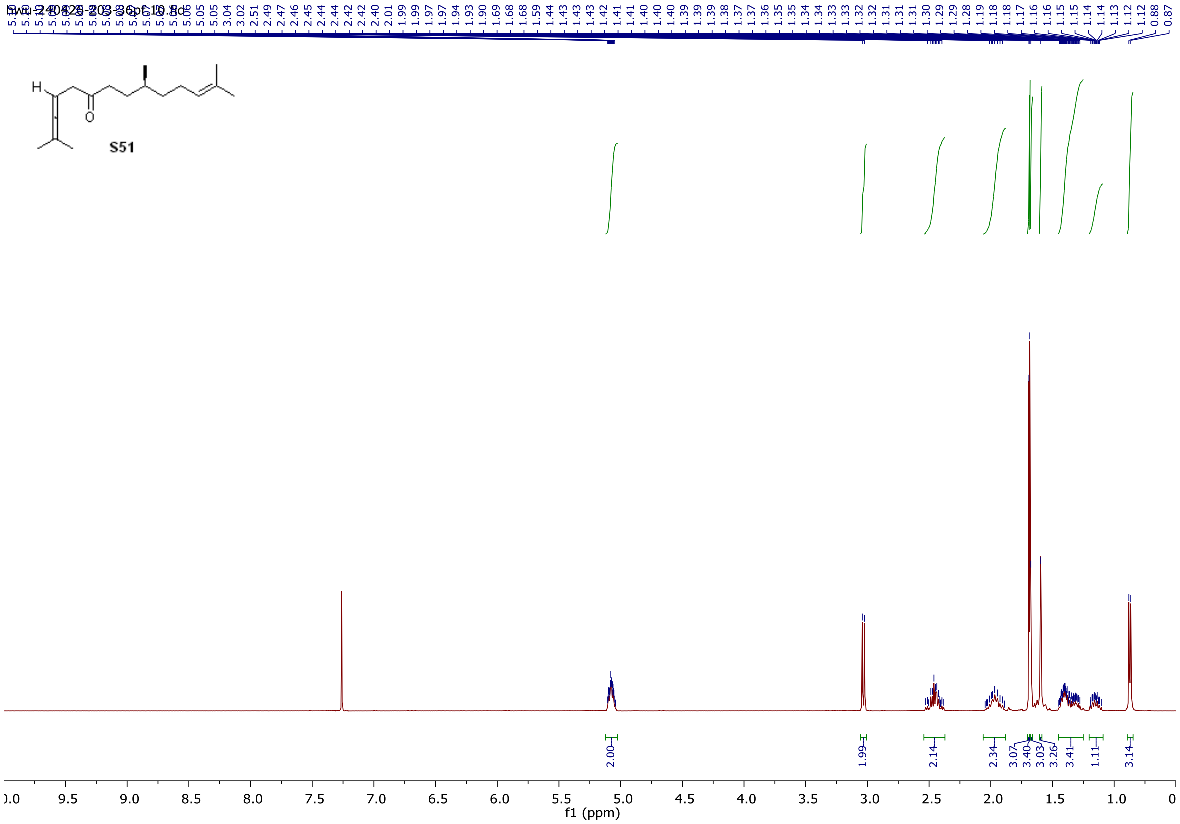
S51
f1 (ppm)

hwu-230608-202-79ap.10.fid

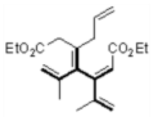

3

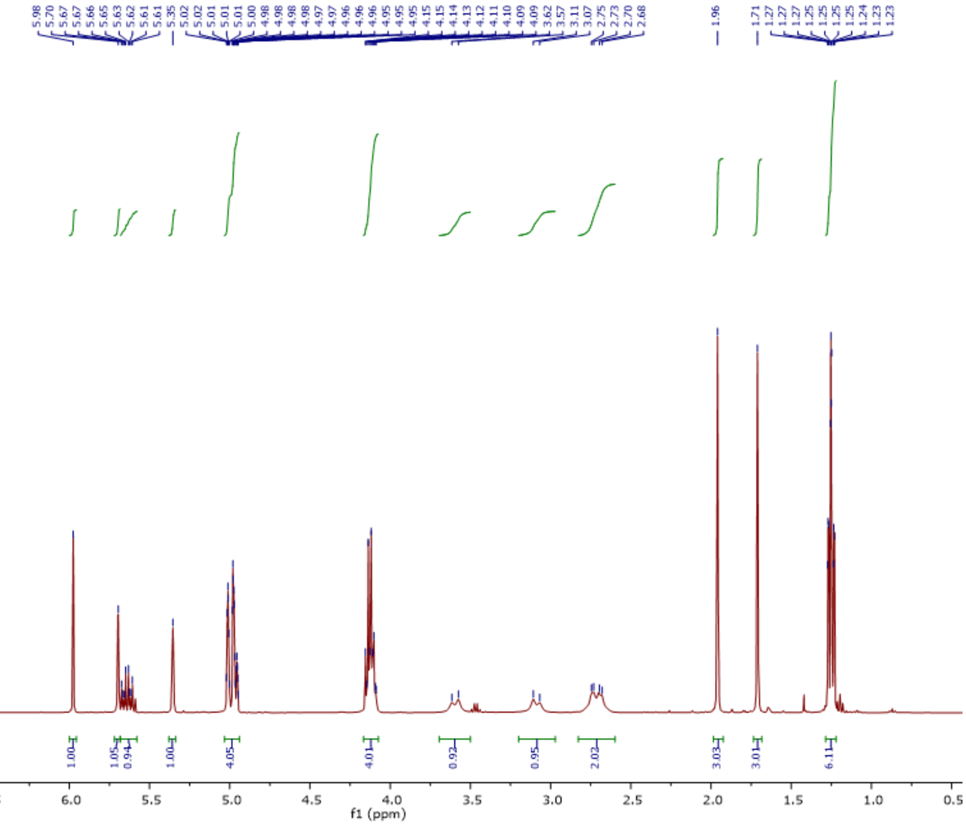

hwu-230608-202-79ap.11.fid

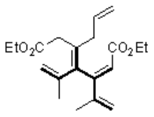

3

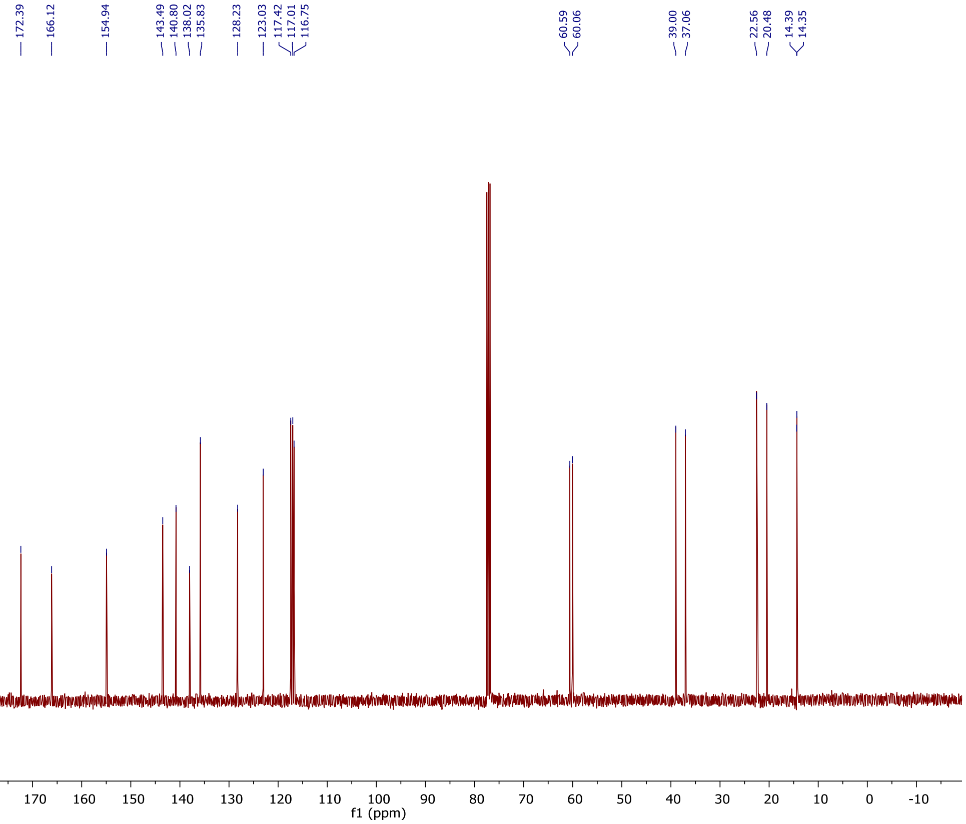

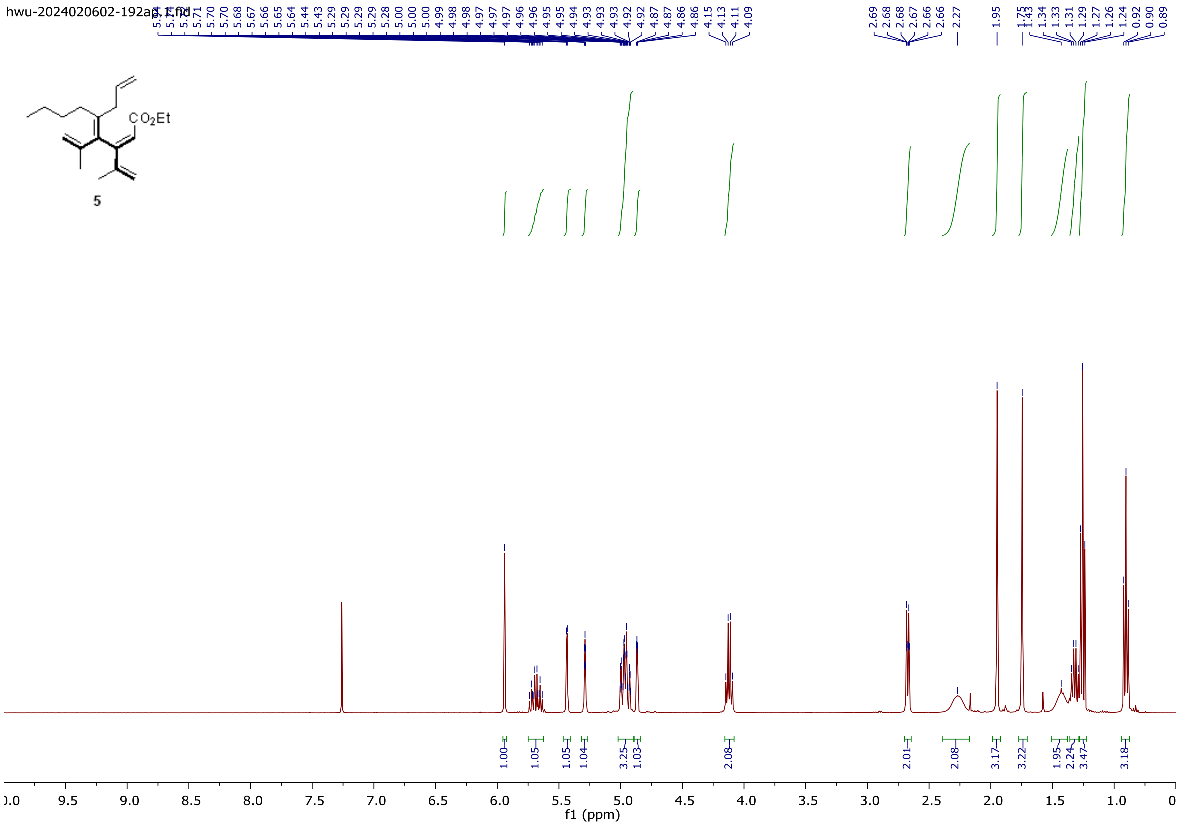
hwu-2024020602-192ap.1.fid
CO2Et
5
f1 (ppm)

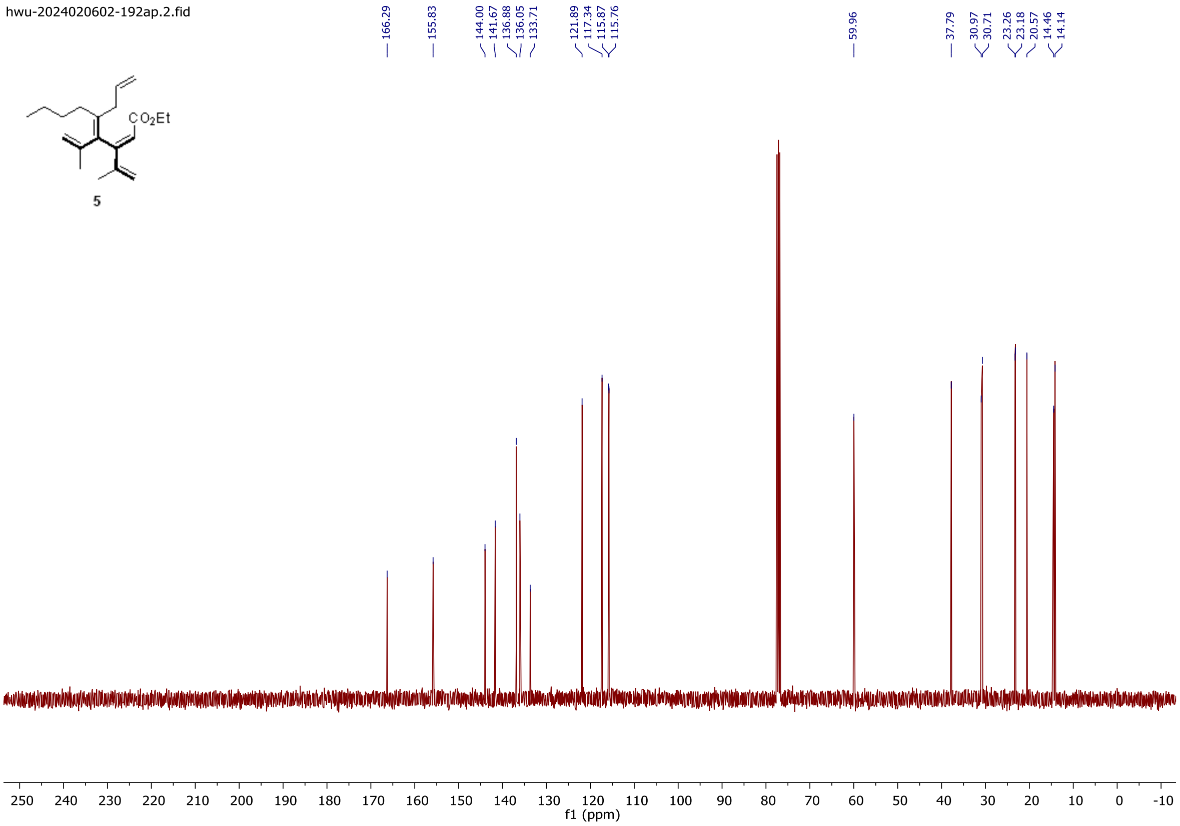
hwu-2024020602-192ap.2.fid
166.29
155.83
144.00
141.67
136.88
136.05
133.71
121.89
117.34
115.87
115.76
59.96
37.79
30.97
30.71
23.26
23.18
20.57
14.46
14.14
CO2Et
5
f1 (ppm)

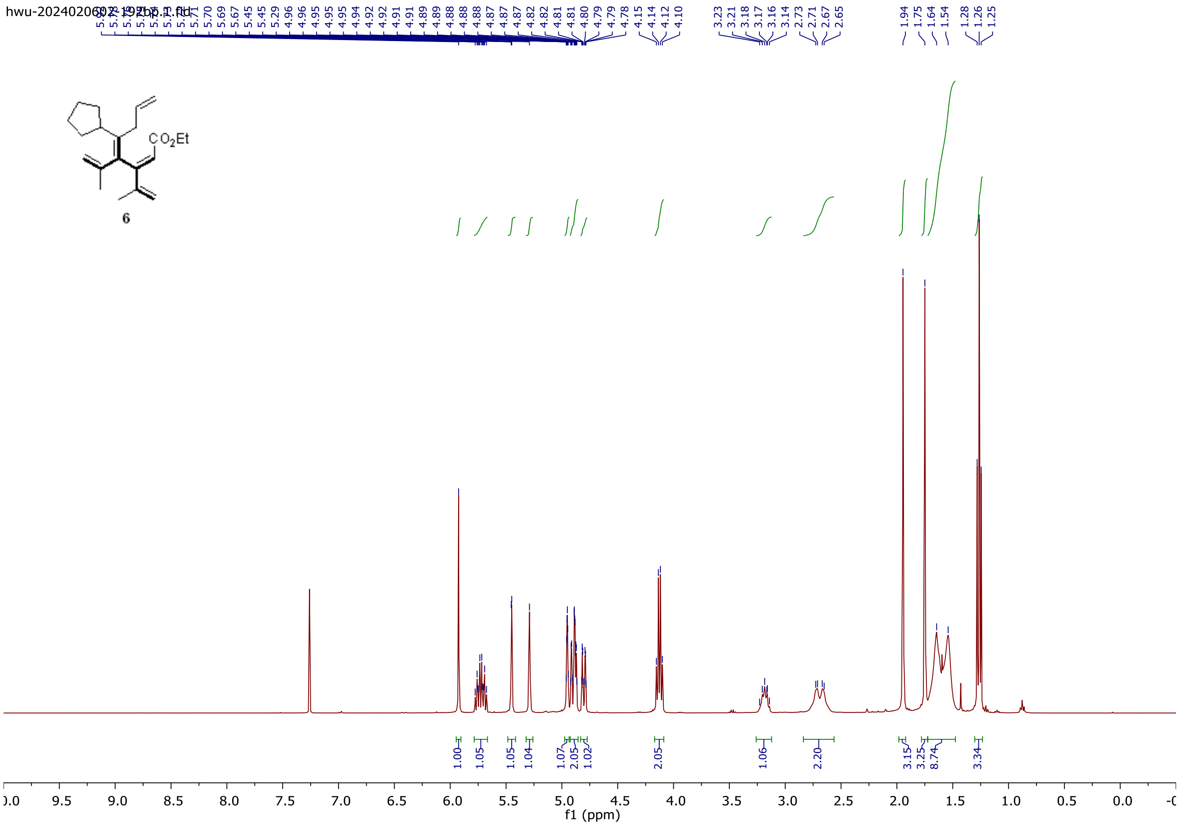
hwu-2024020602-192bp.1.fid
CO2Et
6
1.00
1.05
1.05
1.04
1.07
2.05
1.02
2.05
1.06
2.20
3.15
3.25
8.74
3.34
f1 (ppm)

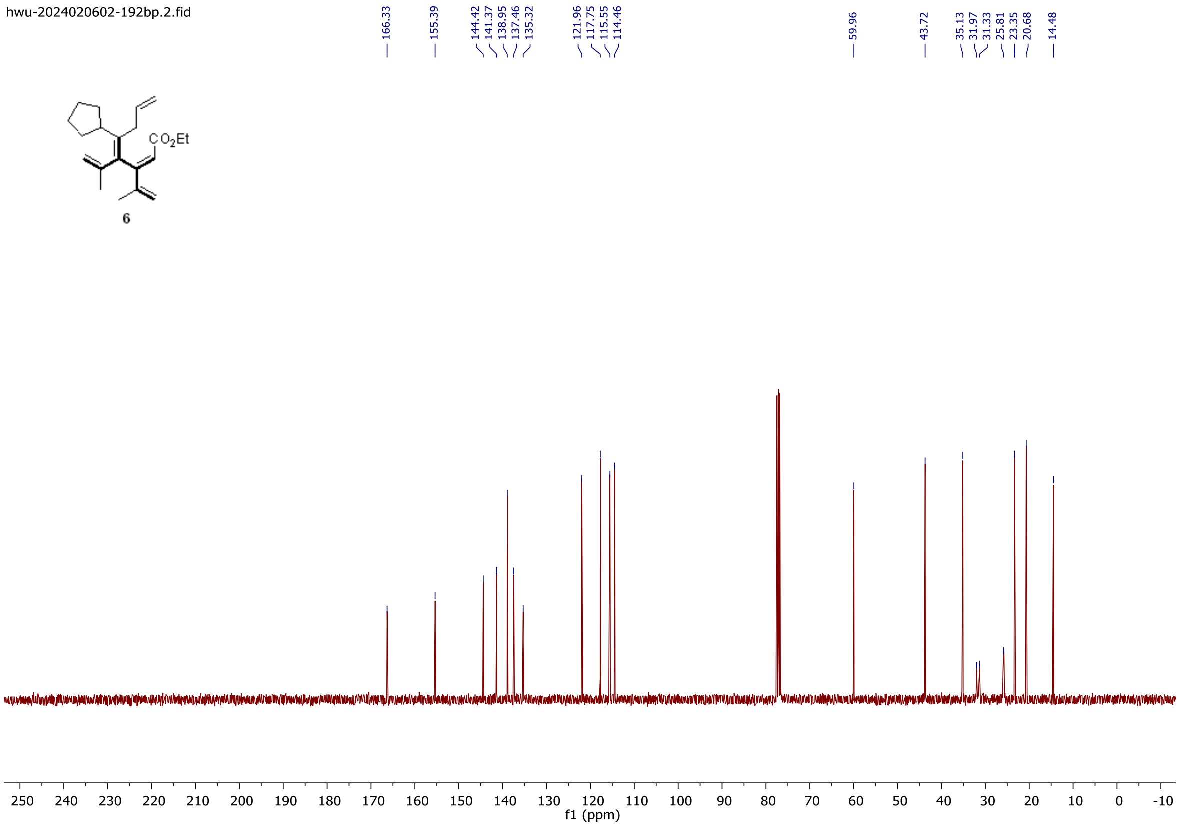
hwu-2024020602-192bp.2.fid
166.33
155.39
144.42
141.37
138.95
137.46
135.32
121.96
117.75
115.55
114.46
59.96
43.72
35.13
31.97
31.33
25.81
23.35
20.68
14.48
CO2Et
6
f1 (ppm)

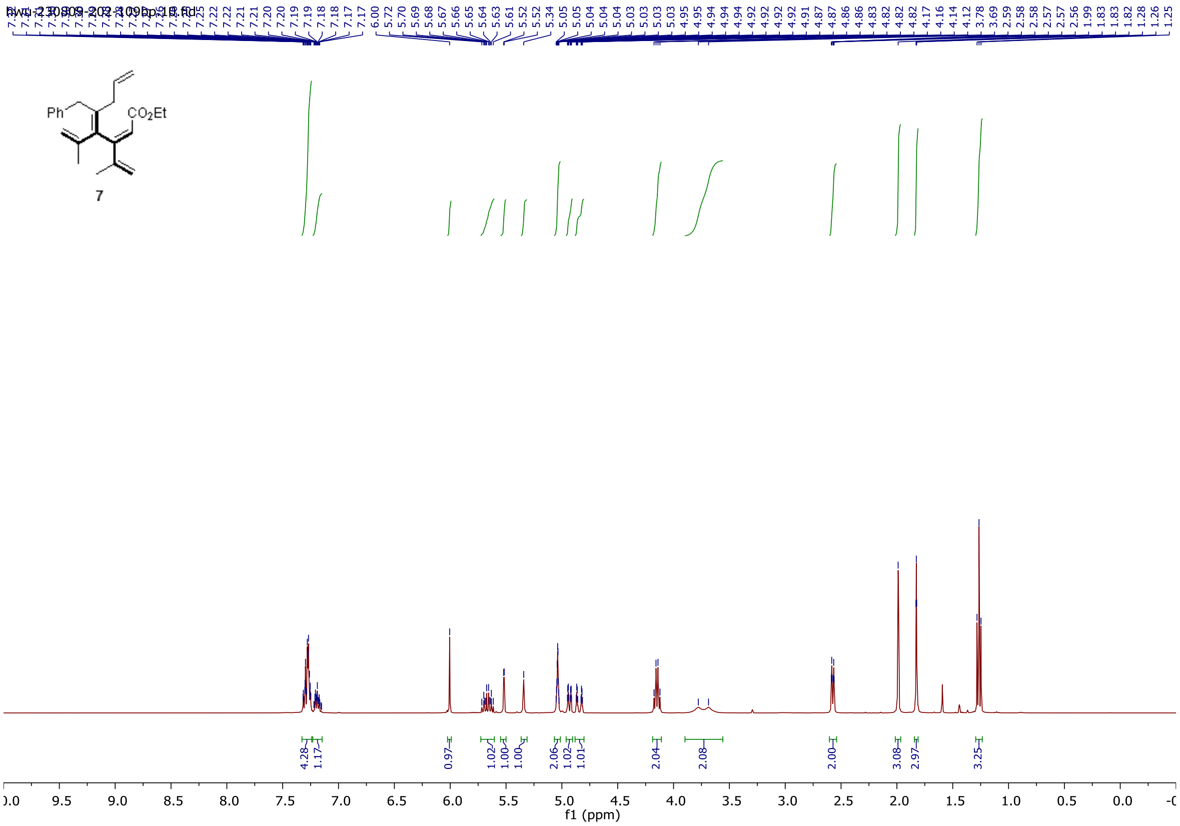

hwu-230809-202-109bp.10.fid
7.22 7.22 7.21 7.20 7.19 7.19 7.18 7.18 7.17 6.00 5.72 5.70 5.69 5.68 5.67 5.66 5.65 5.64 5.63 5.61 5.52 5.52 5.34 5.05 5.05 5.04 5.04 5.04 5.03 5.03 5.03 5.03 4.95 4.95 4.94 4.94 4.94 4.92 4.92 4.92 4.92 4.91 4.87 4.87 4.86 4.86 4.83 4.82 4.82 4.82 4.17 4.16 4.14 4.12 3.78 3.69 2.59 2.58 2.58 2.57 2.57 2.56 1.99 1.83 1.83 1.82 1.82 1.28 1.26 1.25
Ph
CO2Et
7
4.28
1.17
0.97
1.02
1.00
1.00
2.06
1.02
1.01
2.04
2.08
2.00
3.08
2.97
3.25
10.0 9.5 9.0 8.5 8.0 7.5 7.0 6.5 6.0 5.5 5.0 4.5 4.0 3.5 3.0 2.5 2.0 1.5 1.0 0.5 0.0
f1 (ppm)

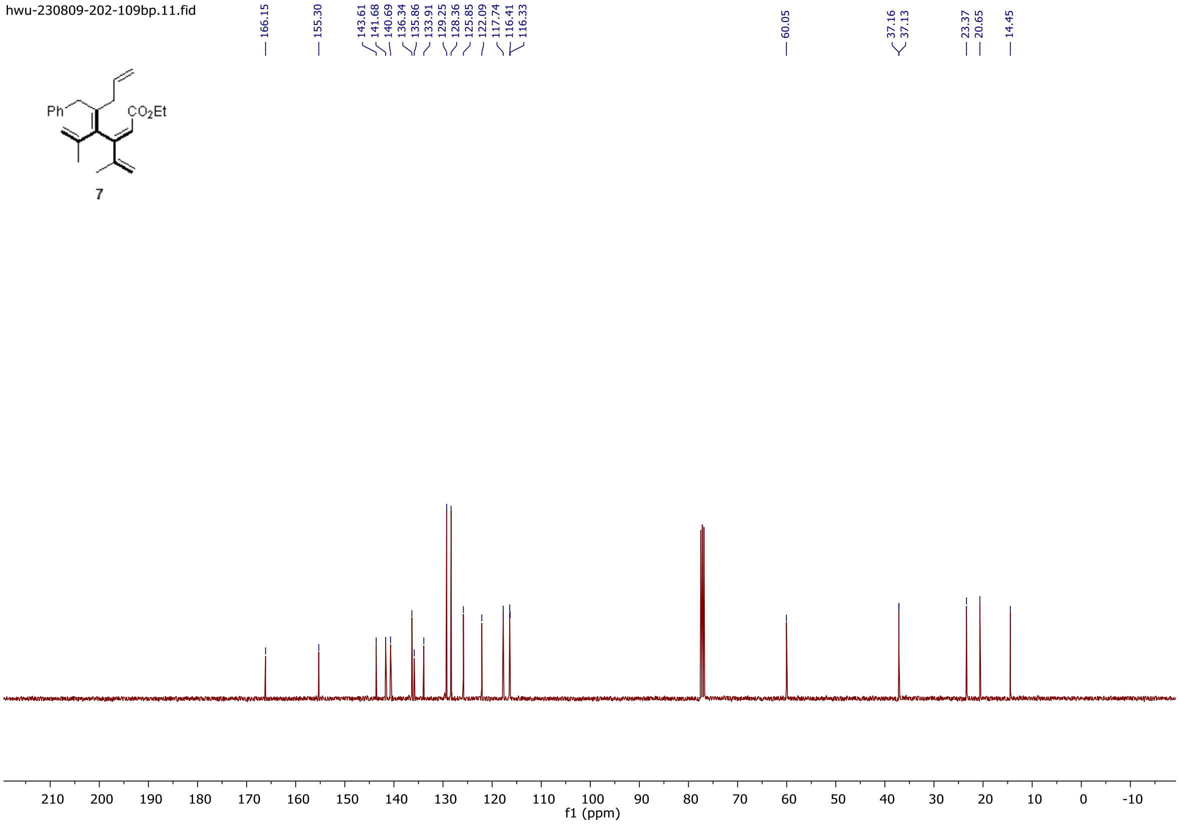

hwu-230809-202-109bp.11.fid
166.15
155.30
143.61 141.68 140.69 136.34 135.86 133.91 129.25 128.36 125.85 122.09 117.74 116.41 116.33
60.05
37.16 37.13
23.37 20.65
14.45
Ph
CO2Et
7
210 200 190 180 170 160 150 140 130 120 110 100 90 80 70 60 50 40 30 20 10 0 -10
f1 (ppm)

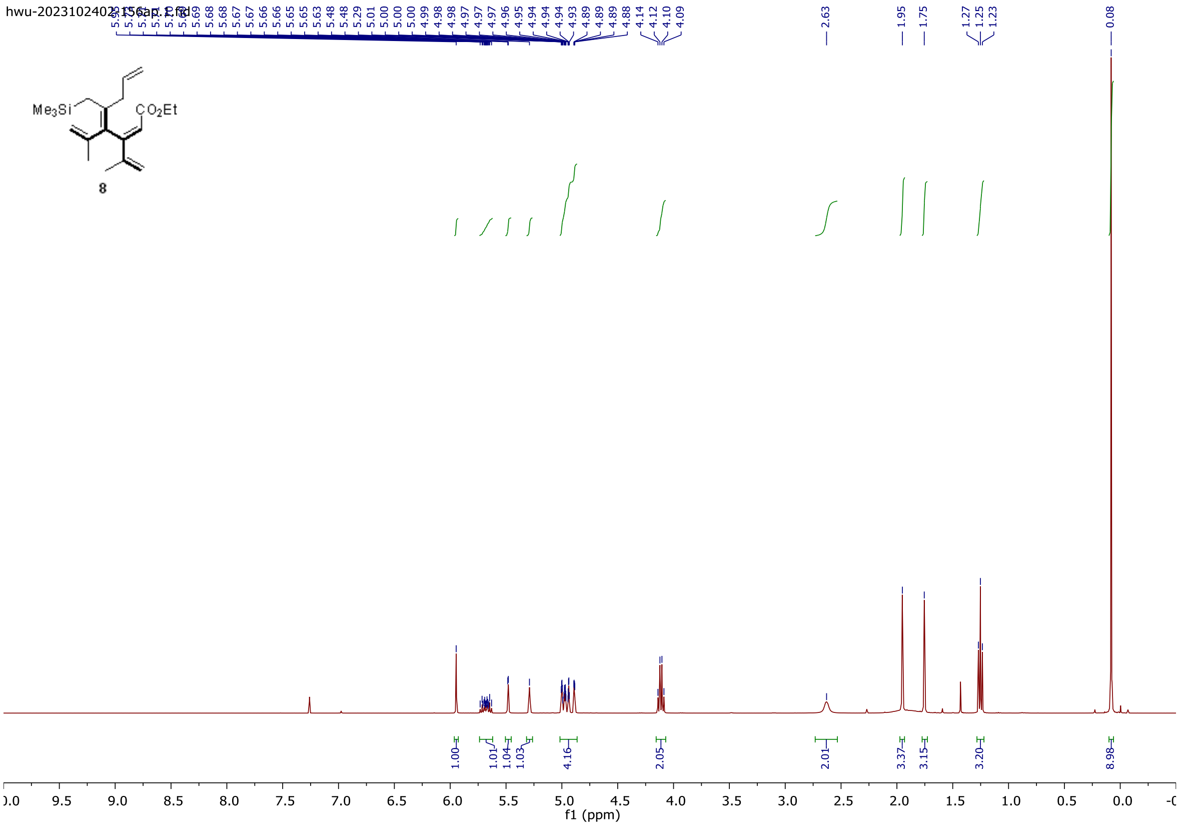
hwu-2023102402-156ap.1.fid
Me3Si
CO2Et
8
5.69 5.68 5.68 5.67 5.66 5.66 5.65 5.65 5.63 5.48 5.48 5.29 5.01 5.00 5.00 5.00 4.99 4.99 4.98 4.98 4.97 4.97 4.97 4.96 4.95 4.94 4.94 4.94 4.93 4.89 4.89 4.89 4.88 4.14 4.12 4.10 4.09
2.63
1.95 1.75
1.27 1.25 1.23
0.08
1.00 1.01 1.04 1.03 4.16 2.05 2.01 3.37 3.15 3.20 8.98
f1 (ppm)

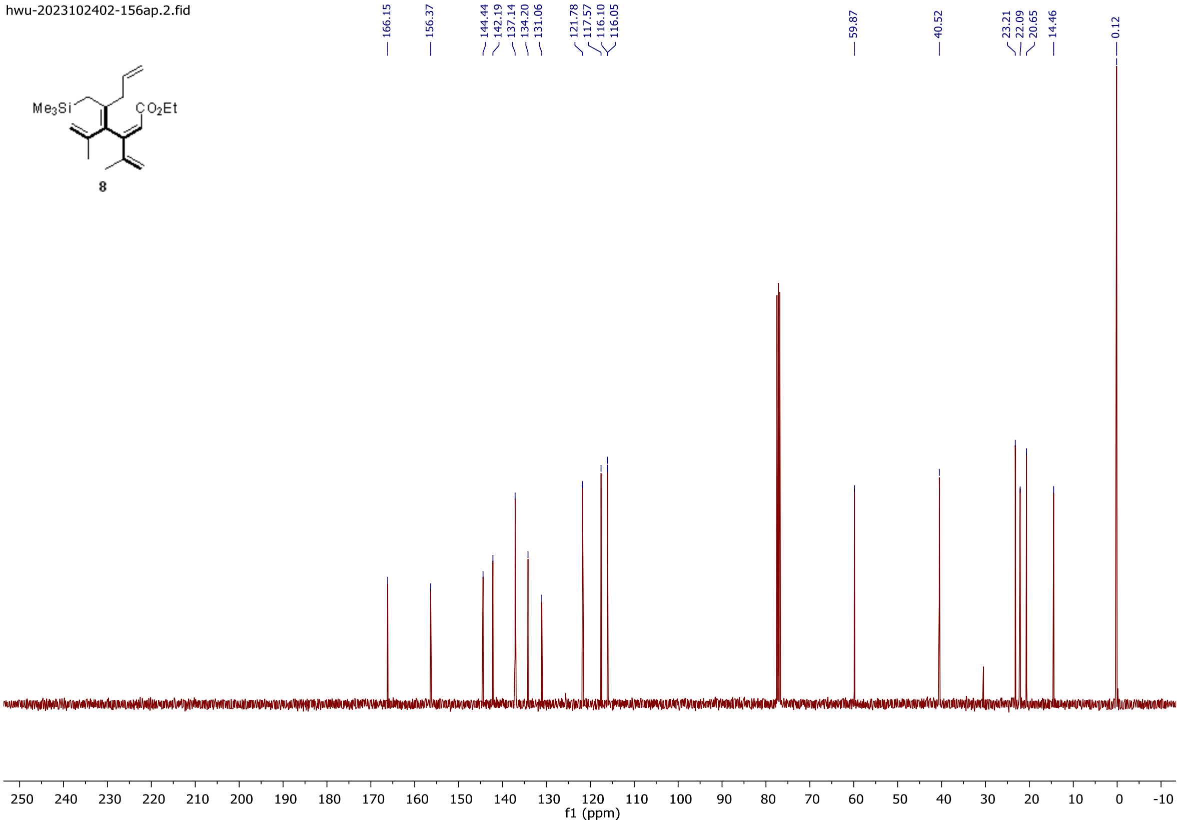
hwu-2023102402-156ap.2.fid
Me3Si
CO2Et
8
166.15
156.37
144.44 142.19 137.14 134.20 131.06
121.78 117.57 116.10 116.05
59.87
40.52
23.21 22.09 20.65
14.46
0.12
f1 (ppm)

hwu-2024030503-01cp.1.fid

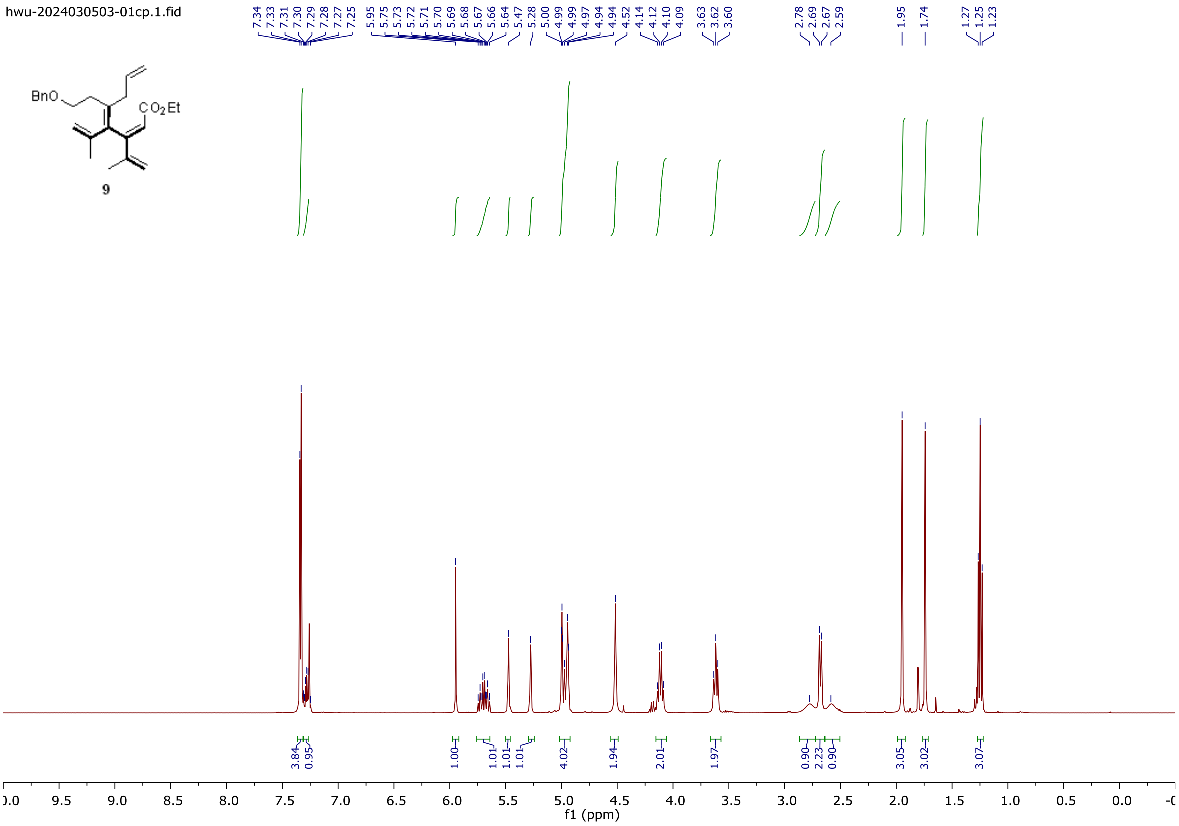

hwu-2024030503-01cp.2.fid

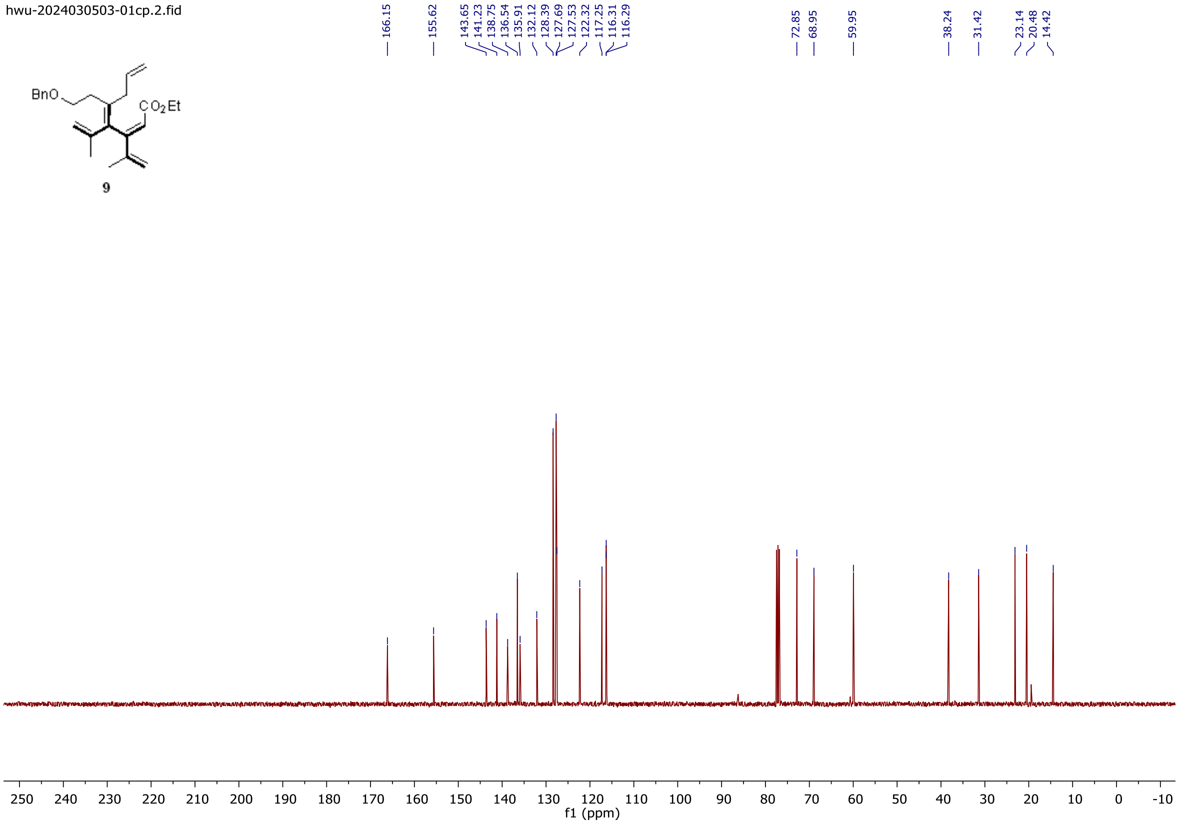

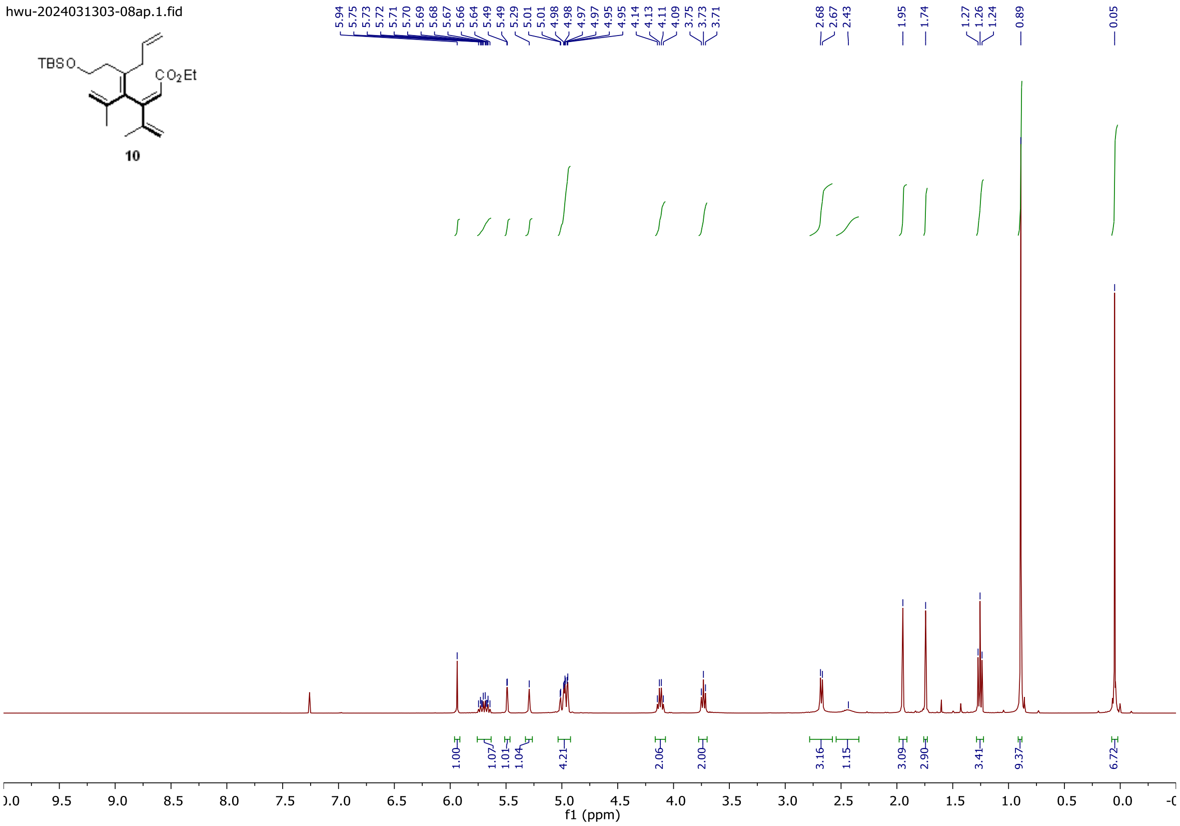
hwu-2024031303-08ap.1.fid
TBSO
CO2Et
10
f1 (ppm)

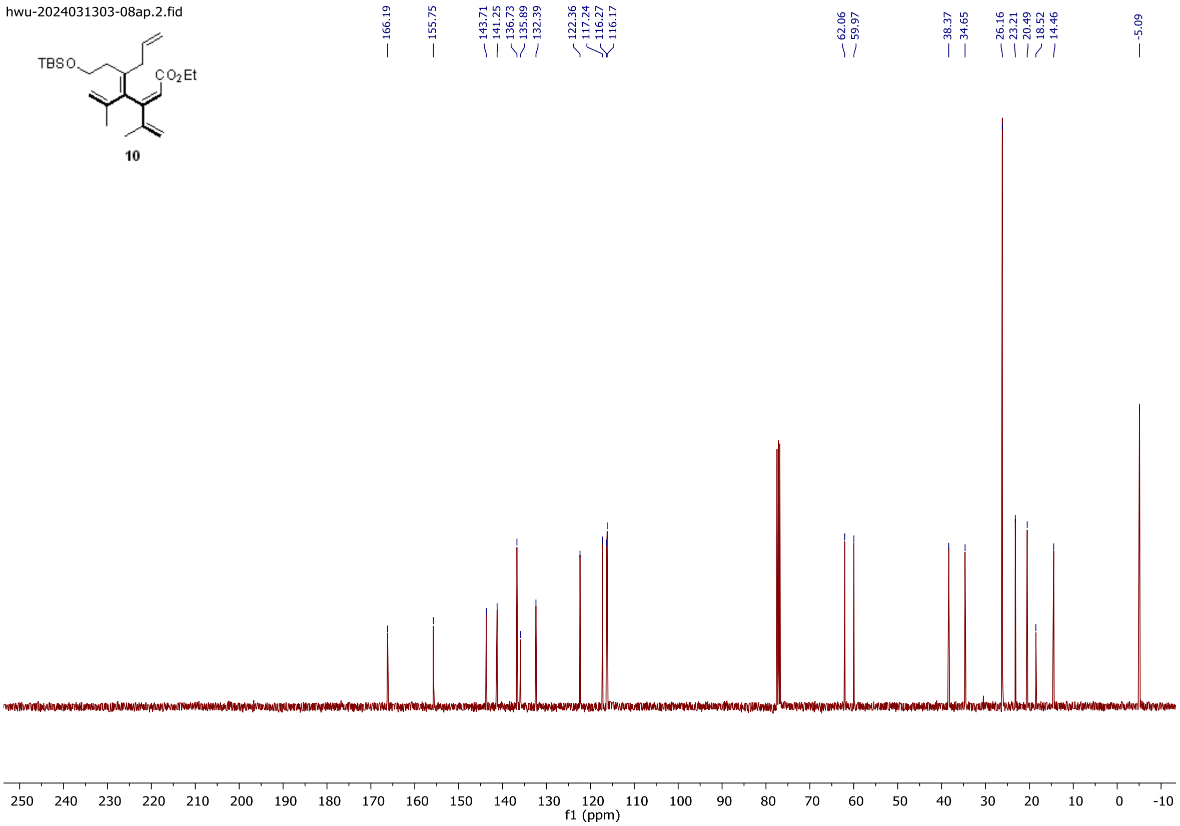
hwu-2024031303-08ap.2.fid
TBSO
CO2Et
10
f1 (ppm)

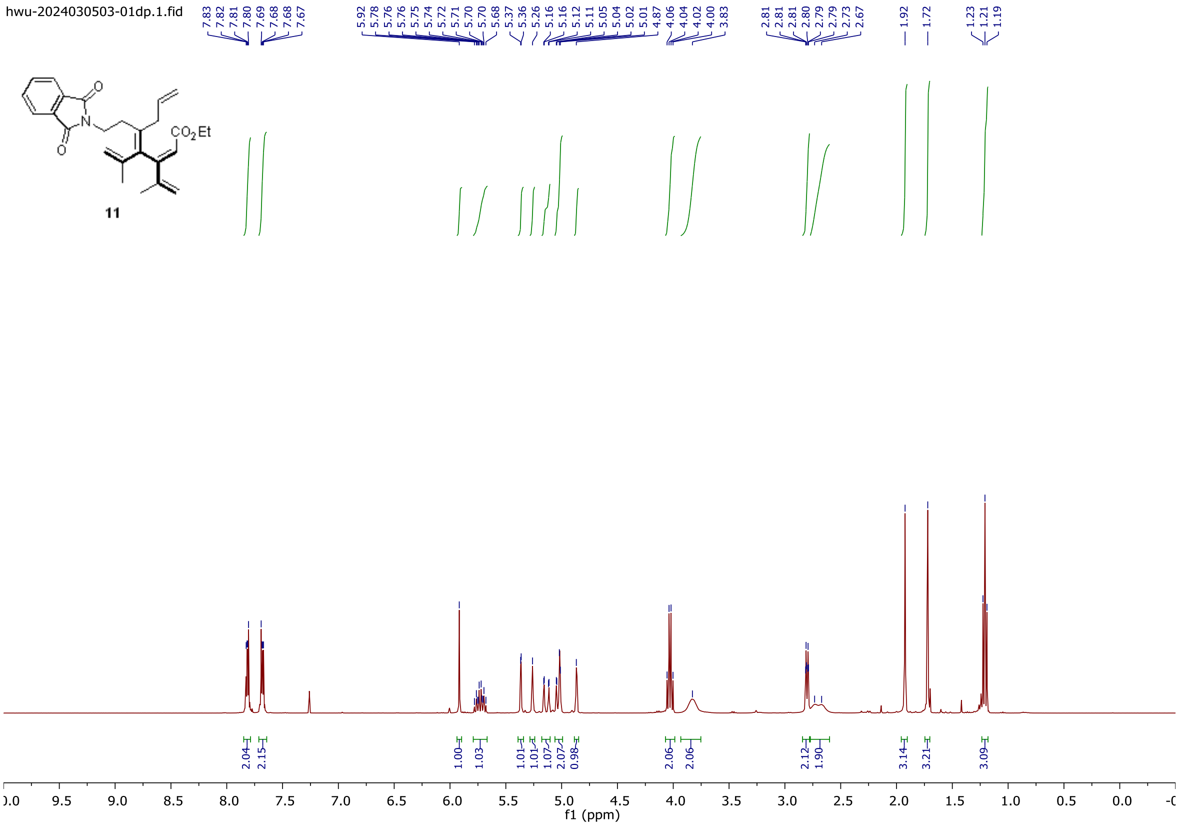
hwu-2024030503-01dp.1.fid
7.83 7.82 7.81 7.80 7.69 7.68 7.68 7.67
5.92 5.78 5.76 5.76 5.75 5.74 5.72 5.71 5.70 5.70 5.68 5.37 5.36 5.26 5.16 5.16 5.12 5.11 5.05 5.04 5.02 5.01 4.87 4.06 4.04 4.02 4.00 3.83
2.81 2.81 2.81 2.80 2.79 2.79 2.73 2.67
1.92 1.72 1.23 1.21 1.19
CO_2Et
11
2.04 2.15 1.00 1.03 1.01 1.01 1.07 2.07 0.98 2.06 2.06 2.12 1.90 3.14 3.21 3.09
f1 (ppm)

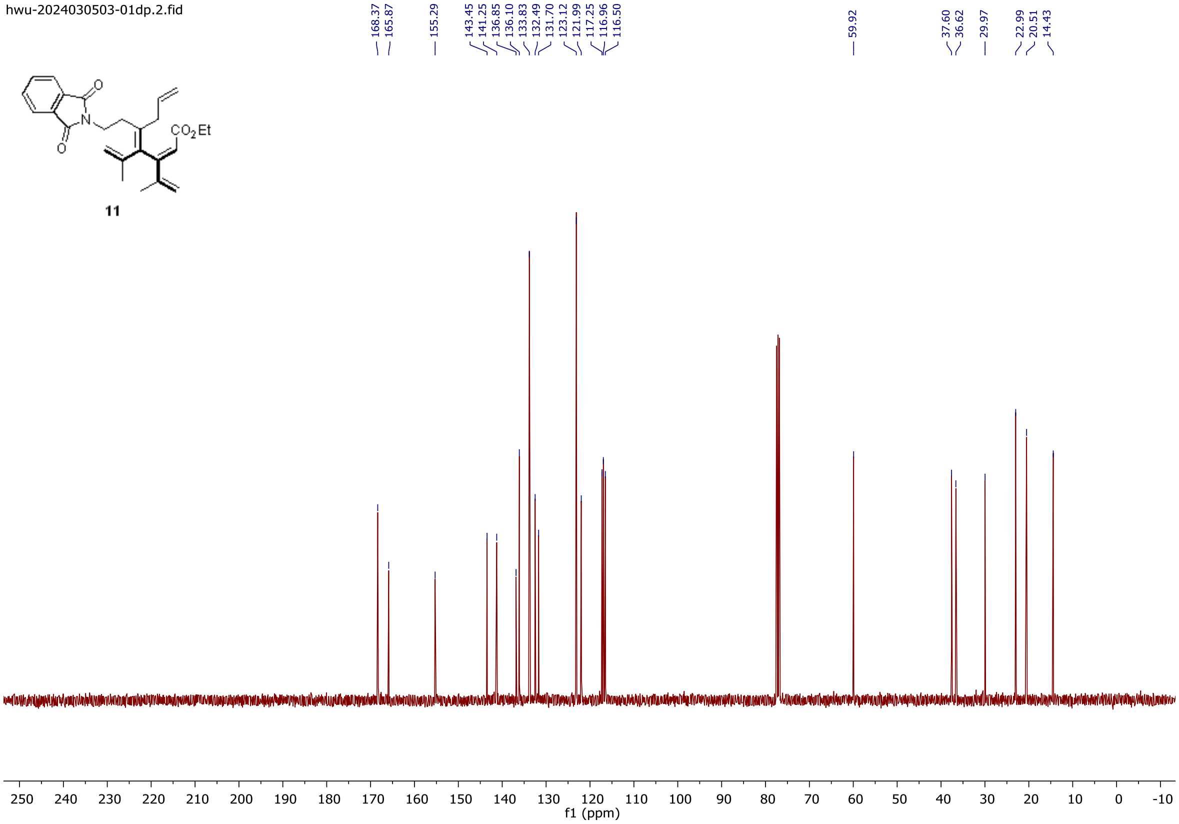
hwu-2024030503-01dp.2.fid
168.37 165.87 155.29 143.45 141.25 136.85 136.10 133.83 132.49 131.70 123.12 121.99 117.25 116.96 116.50 59.92 37.60 36.62 29.97 22.99 20.51 14.43
CO_2Et
11
f1 (ppm)

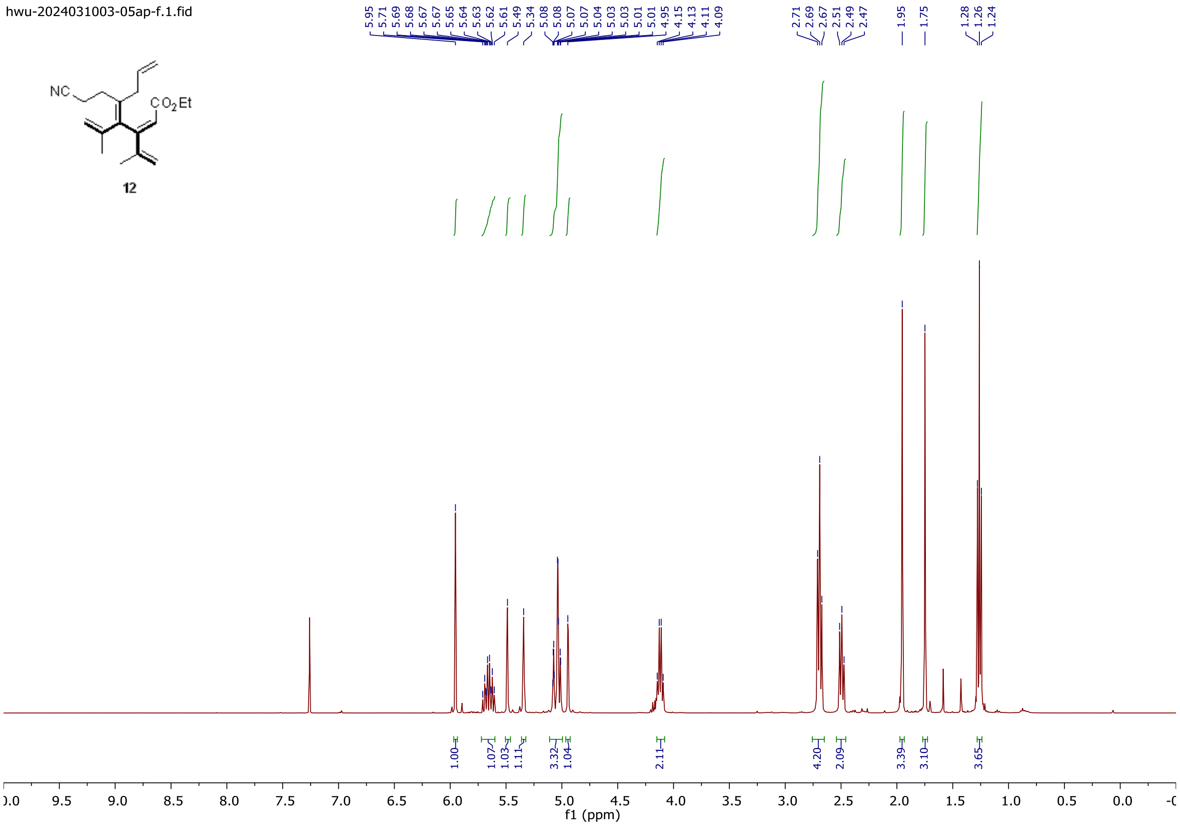
hwu-2024031003-05ap-f.1.fid
NC
CO2Et
12
5.95 5.71 5.69 5.68 5.67 5.67 5.65 5.64 5.63 5.62 5.61 5.49 5.34 5.08 5.08 5.07 5.04 5.03 5.03 5.01 5.01 4.95 4.15 4.13 4.11 4.09
2.71 2.69 2.67 2.51 2.49 2.47
1.95
1.75
1.28 1.26 1.24
1.00 1.07 1.03 1.11 3.32 1.04 2.11 4.20 2.09 3.39 3.10 3.65
f1 (ppm)

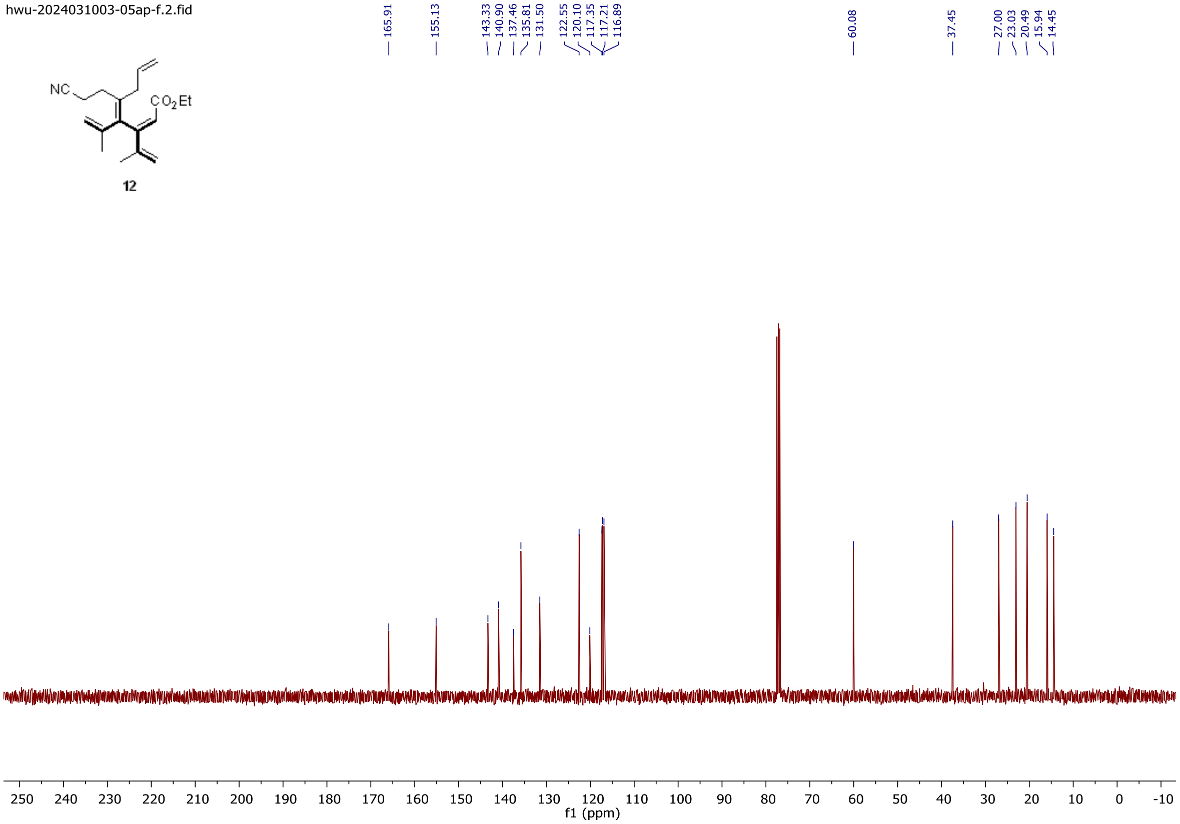
hwu-2024031003-05ap-f.2.fid
NC
CO2Et
12
165.91
155.13
143.33 140.90 137.46 135.81 131.50
122.55 120.10 117.35 117.21 116.89
60.08
37.45
27.00 23.03 20.49 15.94 14.45
f1 (ppm)

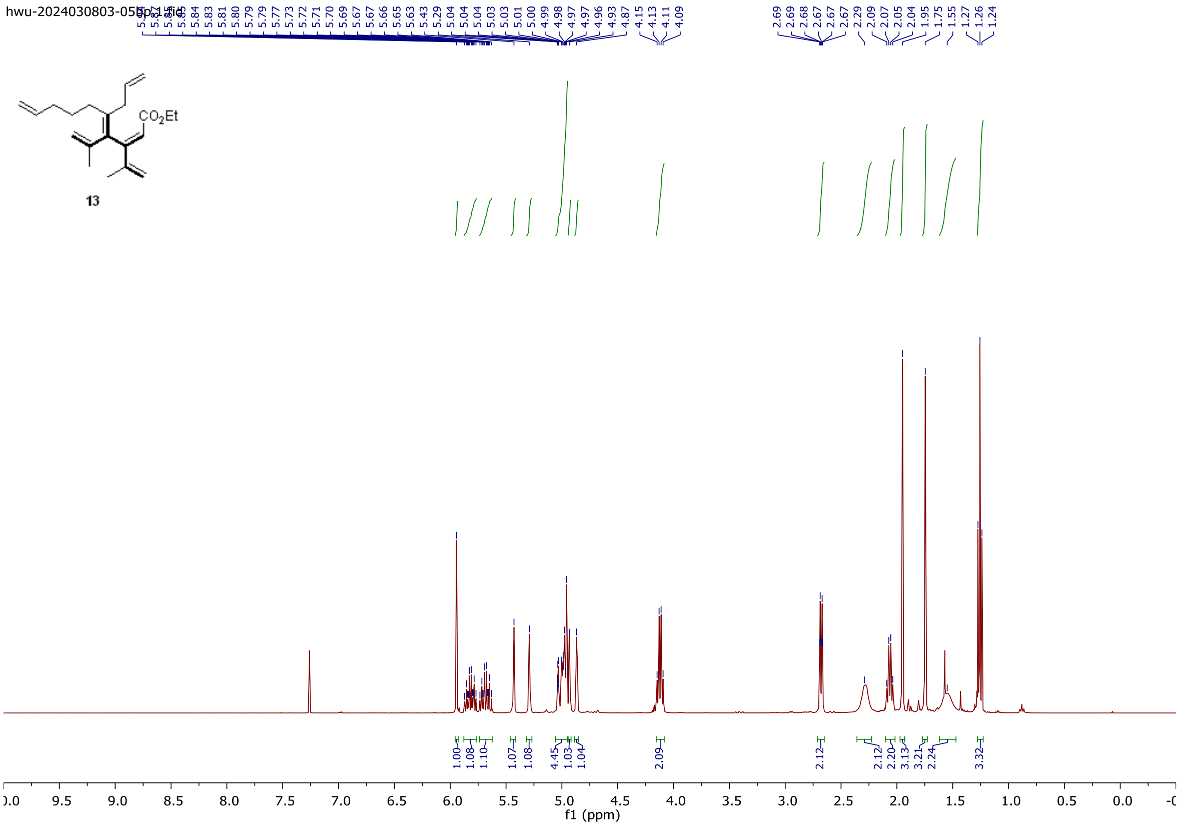
hwu-2024030803-05bp.1.fid
5.84
5.83
5.81
5.80
5.79
5.79
5.77
5.73
5.72
5.71
5.70
5.69
5.67
5.67
5.66
5.65
5.63
5.43
5.29
5.04
5.04
5.04
5.03
5.01
5.00
4.99
4.98
4.97
4.97
4.96
4.93
4.87
4.15
4.13
4.11
4.09
2.69
2.69
2.68
2.67
2.67
2.67
2.29
2.09
2.07
2.05
2.04
1.95
1.75
1.55
1.27
1.26
1.24
CO2Et
13
1.00
1.08
1.10
1.07
1.08
4.45
1.03
1.04
2.09
2.12
2.12
2.20
3.13
3.21
2.24
3.32
f1 (ppm)

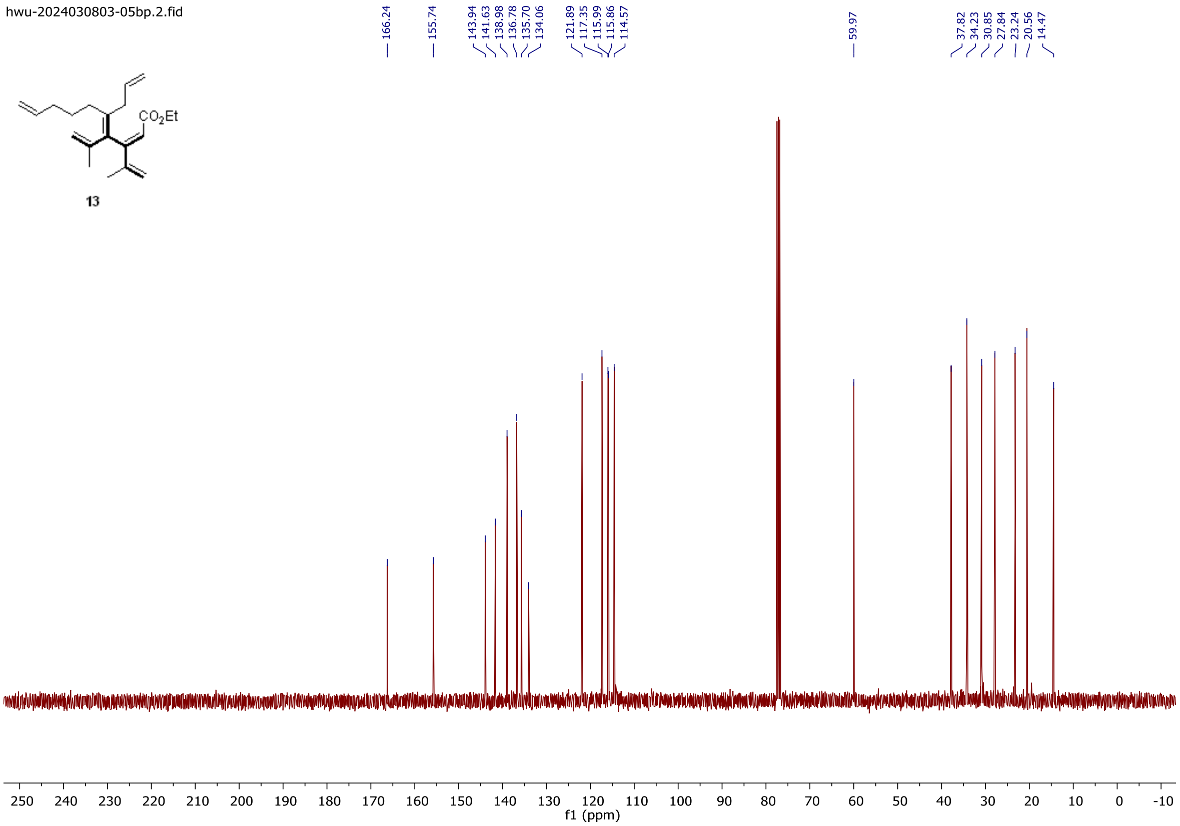
hwu-2024030803-05bp.2.fid
166.24
155.74
143.94
141.63
138.98
136.78
135.70
134.06
121.89
117.35
115.99
115.86
114.57
59.97
37.82
34.23
30.85
27.84
23.24
20.56
14.47
CO2Et
13
f1 (ppm)

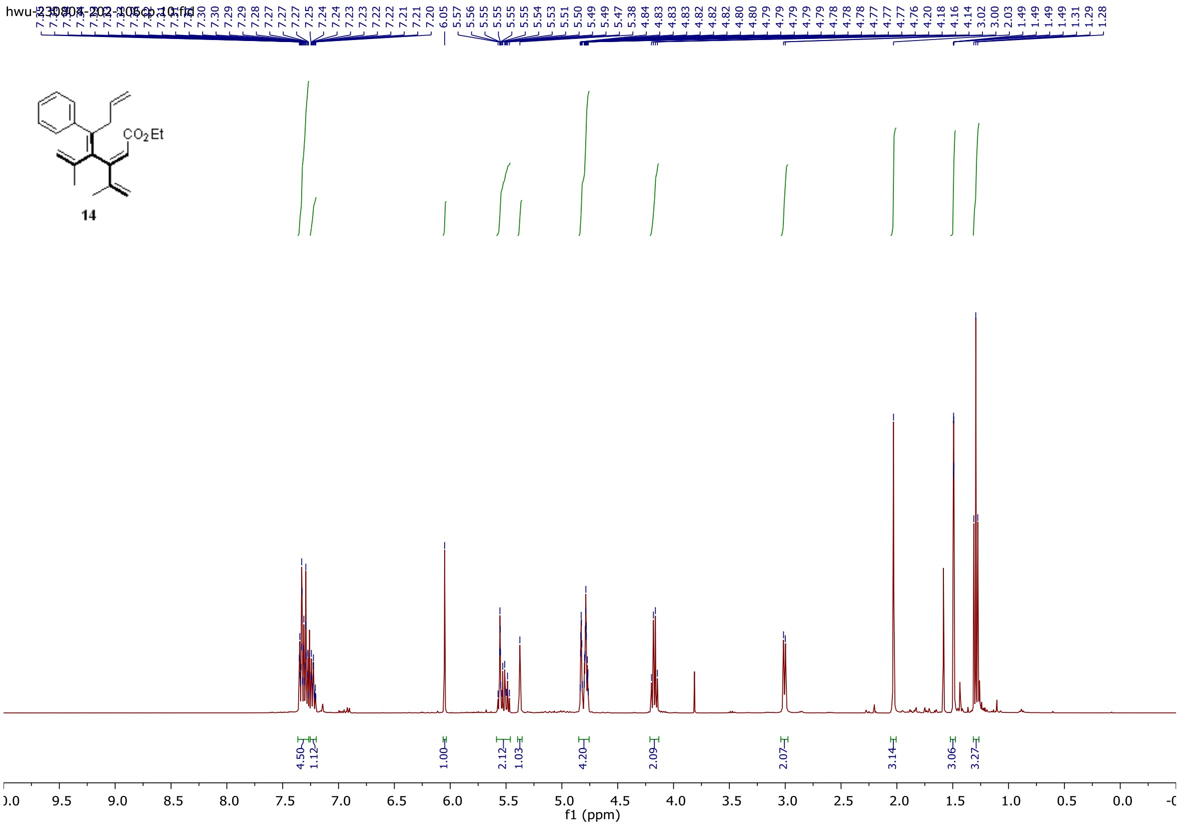
hwu-230804-202-106cp.10.fid
7.30 7.30 7.29 7.29 7.28 7.27 7.27 7.25 7.24 7.24 7.23 7.23 7.22 7.22 7.21 7.20 6.05 5.57 5.56 5.55 5.55 5.55 5.55 5.54 5.53 5.51 5.50 5.49 5.49 5.47 5.38 4.84 4.83 4.83 4.83 4.82 4.82 4.80 4.80 4.79 4.79 4.79 4.79 4.79 4.78 4.78 4.77 4.77 4.77 4.76 4.20 4.18 4.16 4.14 3.02 3.00 2.03 1.49 1.49 1.49 1.49 1.31 1.29 1.28
CO2Et
14
4.50 1.12 1.00 2.12 1.03 4.20 2.09 2.07 3.14 3.06 3.27
9.5 9.0 8.5 8.0 7.5 7.0 6.5 6.0 5.5 5.0 4.5 4.0 3.5 3.0 2.5 2.0 1.5 1.0 0.5 0.0
f1 (ppm)

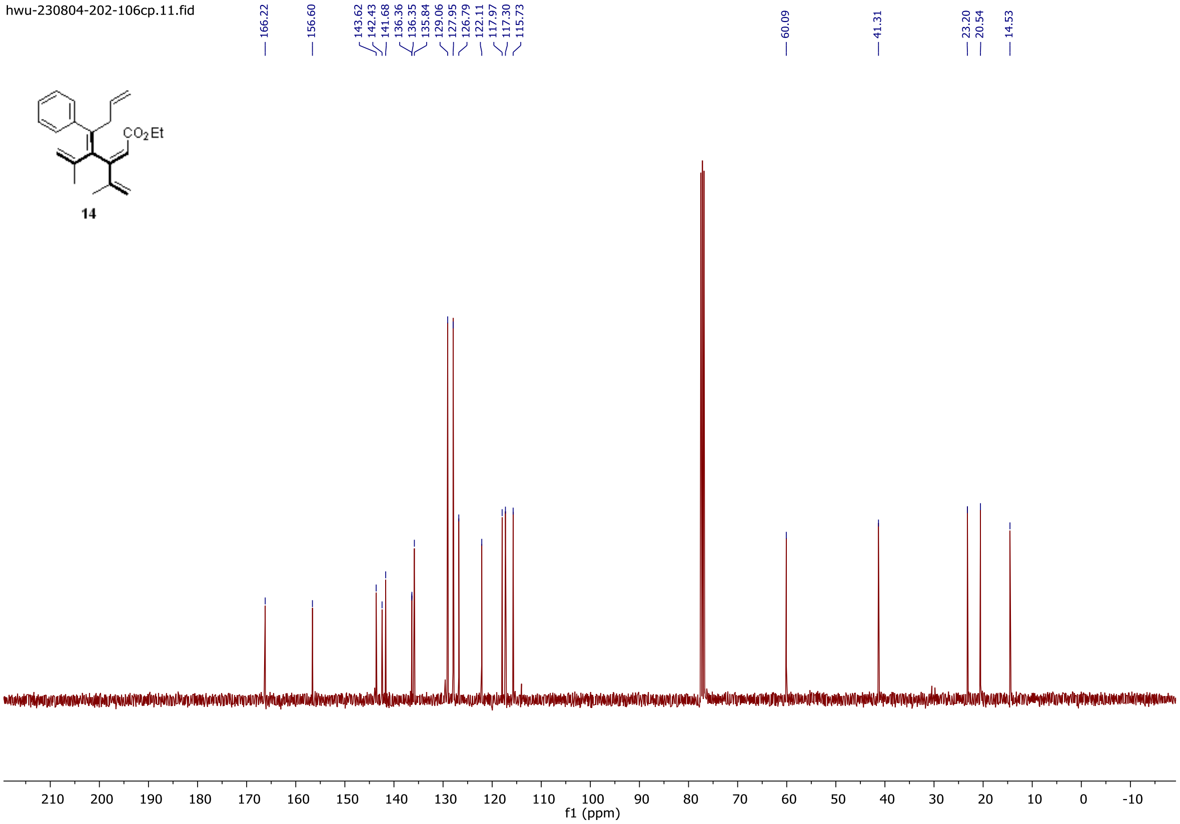
hwu-230804-202-106cp.11.fid
166.22 156.60 143.62 142.43 141.68 136.36 136.35 135.84 129.06 127.95 126.79 122.11 117.97 117.30 115.73 60.09 41.31 23.20 20.54 14.53
CO2Et
14
210 200 190 180 170 160 150 140 130 120 110 100 90 80 70 60 50 40 30 20 10 0 -10
f1 (ppm)

hwu-2023102502-156cf.1.fid

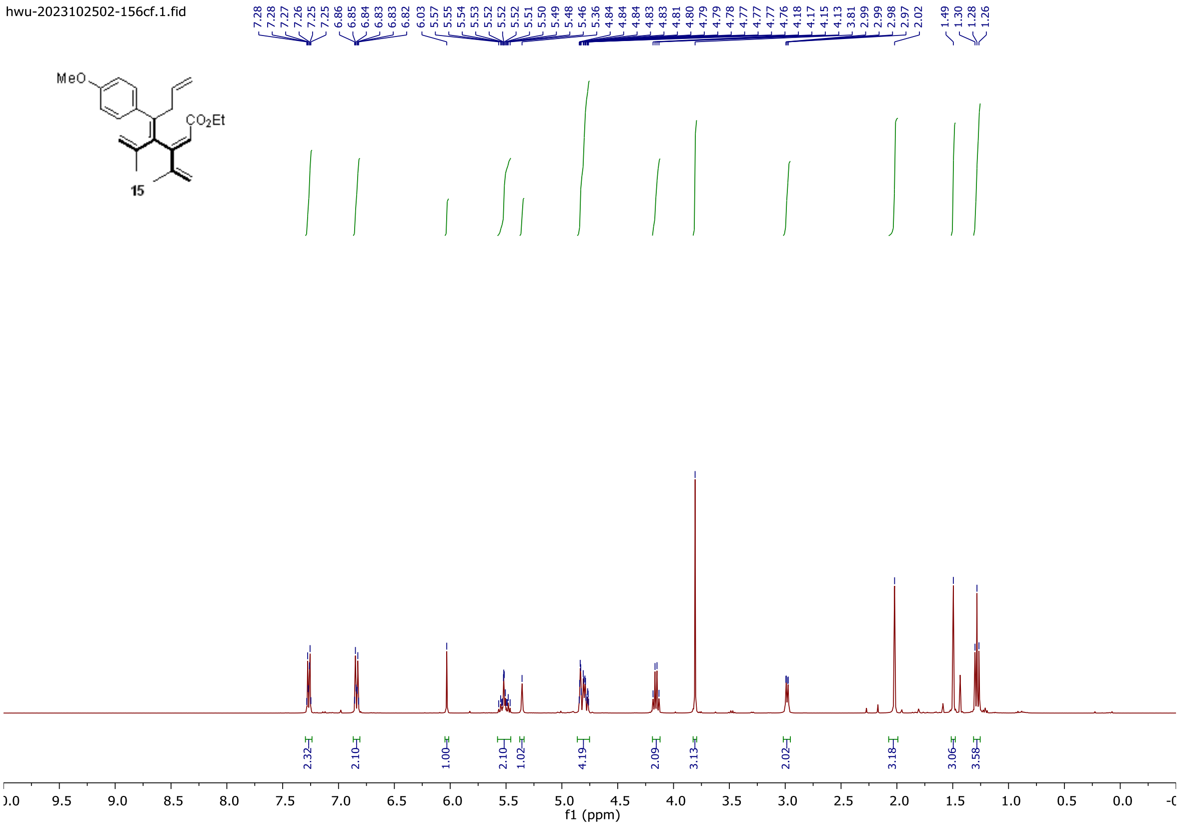

hwu-2023102502-156cf.3.fid

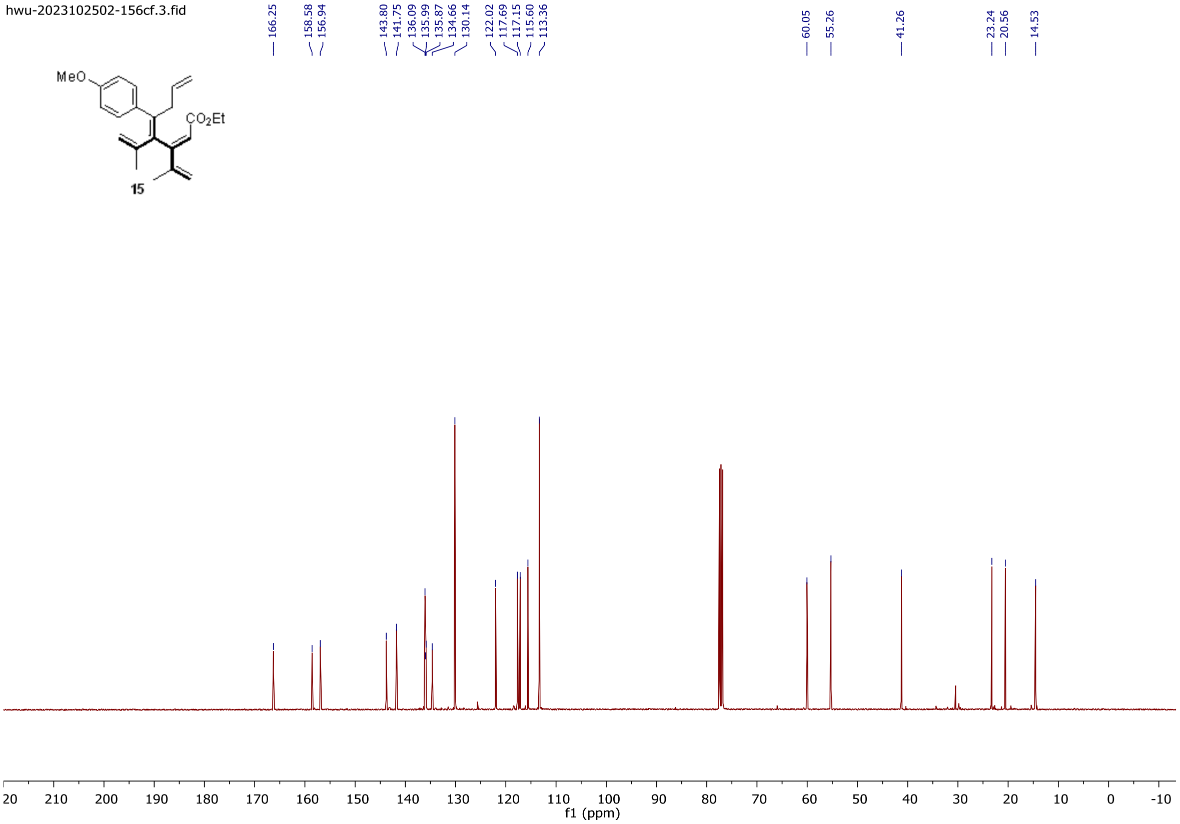

hwu-231024-202-156bp.10.fid

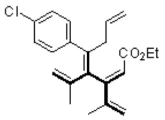

16

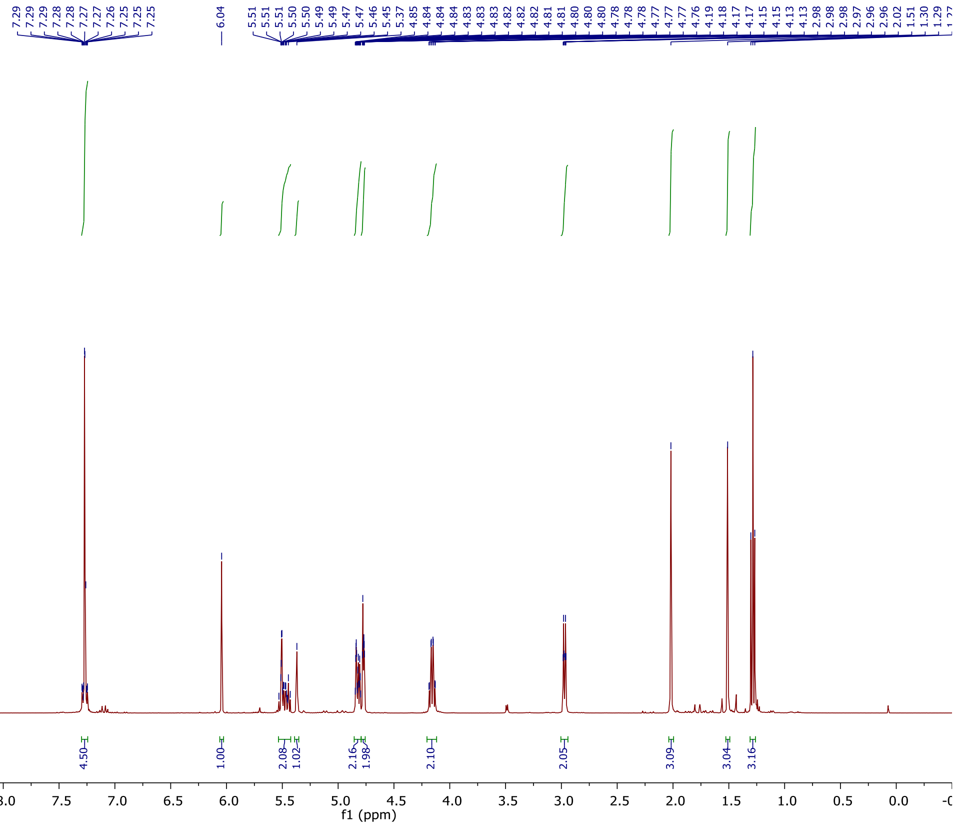

hwu-231024-202-156bp.11.fid

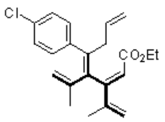

16

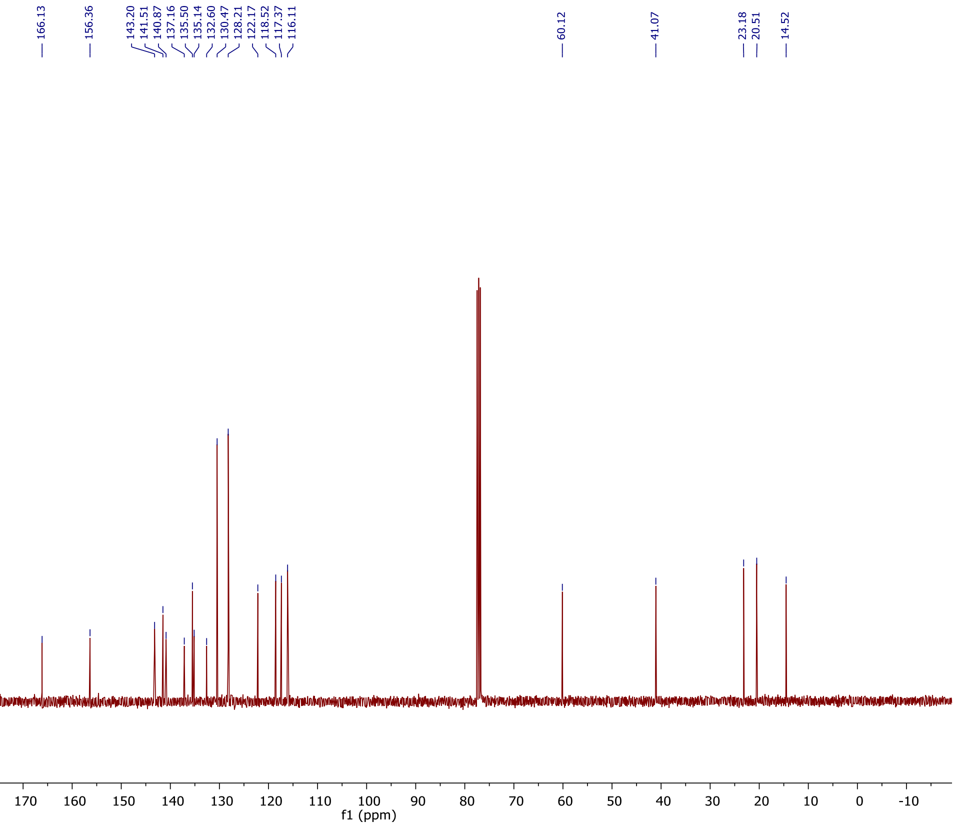

hwu-2024022002-199apf.1.fid

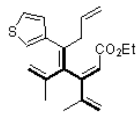

17

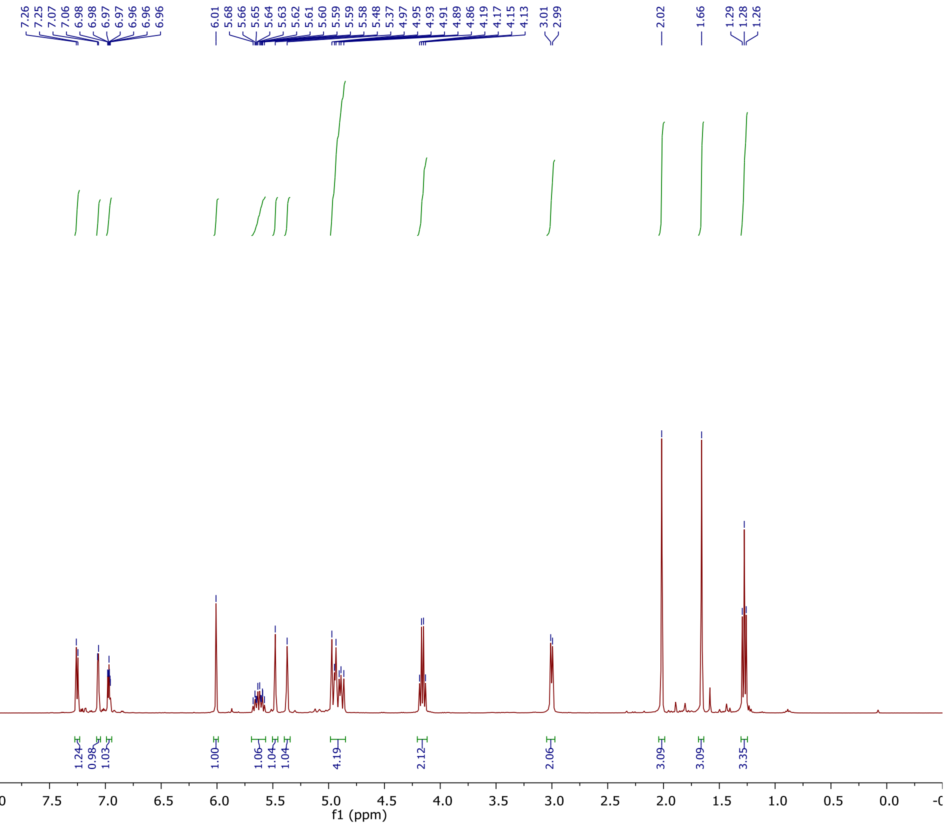

hwu-2024022002-199apf.2.fid

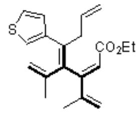

17

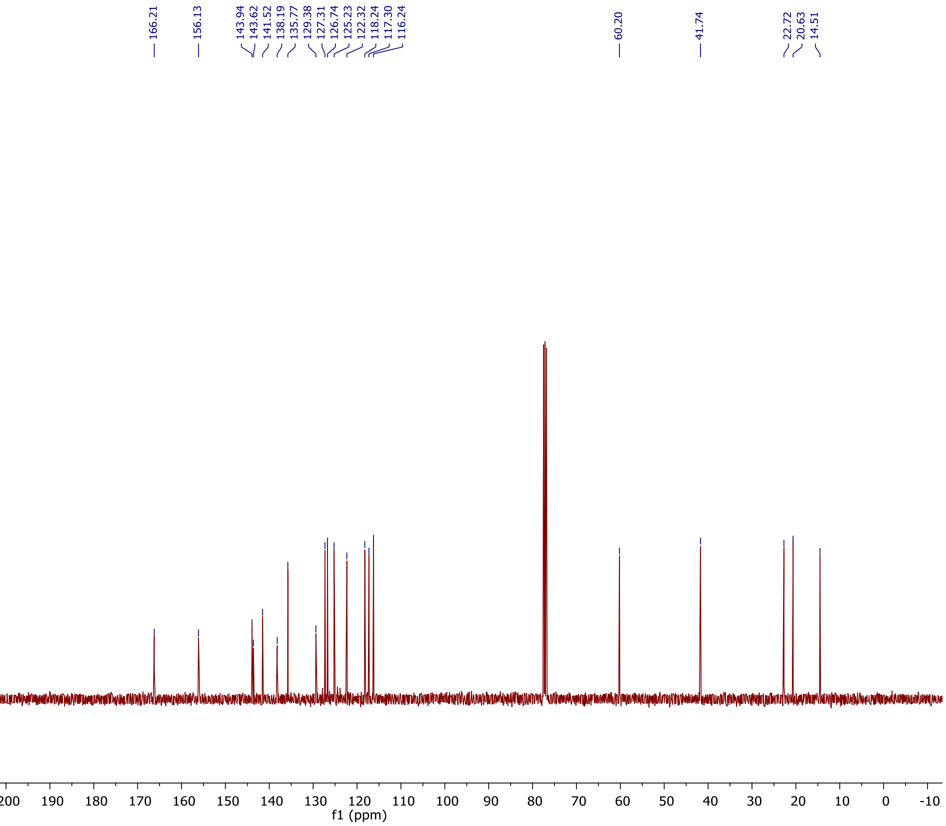

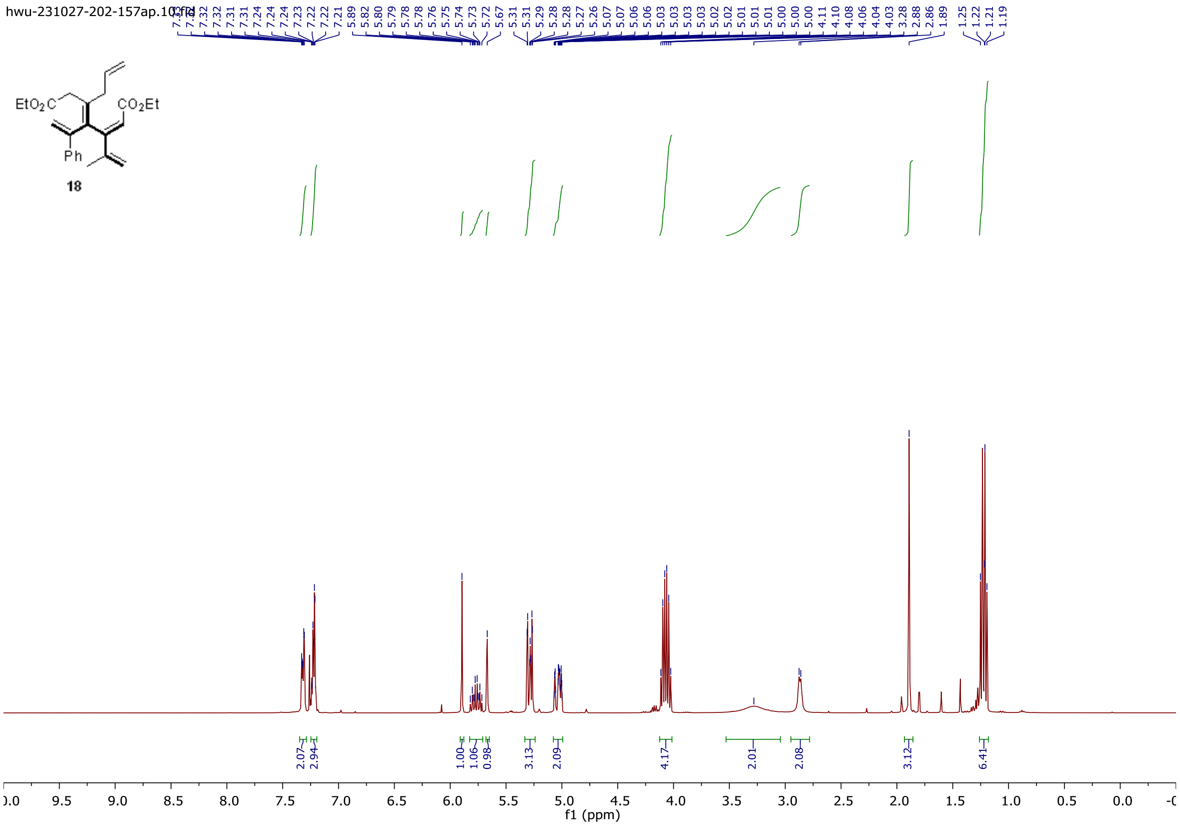
hwu-231027-202-157ap.10.fid
7.32 7.32 7.31 7.31 7.24 7.24 7.23 7.22 7.22 7.21 5.89 5.82 5.80 5.79 5.78 5.78 5.76 5.75 5.74 5.73 5.72 5.67 5.31 5.29 5.28 5.28 5.27 5.26 5.07 5.07 5.06 5.06 5.03 5.03 5.03 5.03 5.02 5.02 5.01 5.01 5.01 5.00 5.00 5.00 4.11 4.10 4.08 4.06 4.04 4.03 3.28 2.88 2.86 1.89 1.25 1.22 1.21 1.19
EtO2C
CO2Et
Ph
18
2.07 2.94 1.00 1.06 0.98 3.13 2.09 4.17 2.01 2.08 3.12 6.41
f1 (ppm)

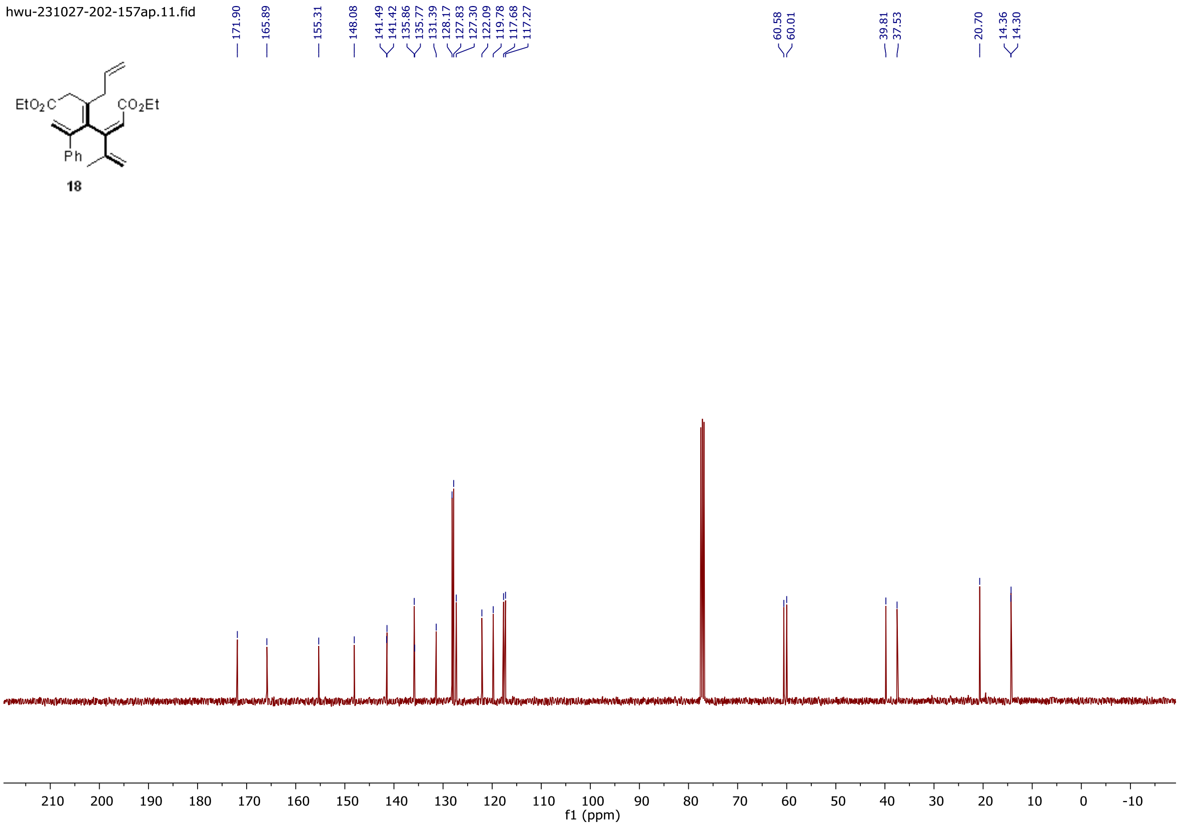
hwu-231027-202-157ap.11.fid
171.90 165.89 155.31 148.08 141.49 141.42 135.86 135.77 131.39 128.17 127.83 127.30 122.09 119.78 117.68 117.27 60.58 60.01 39.81 37.53 20.70 14.36 14.30
EtO2C
CO2Et
Ph
18
f1 (ppm)

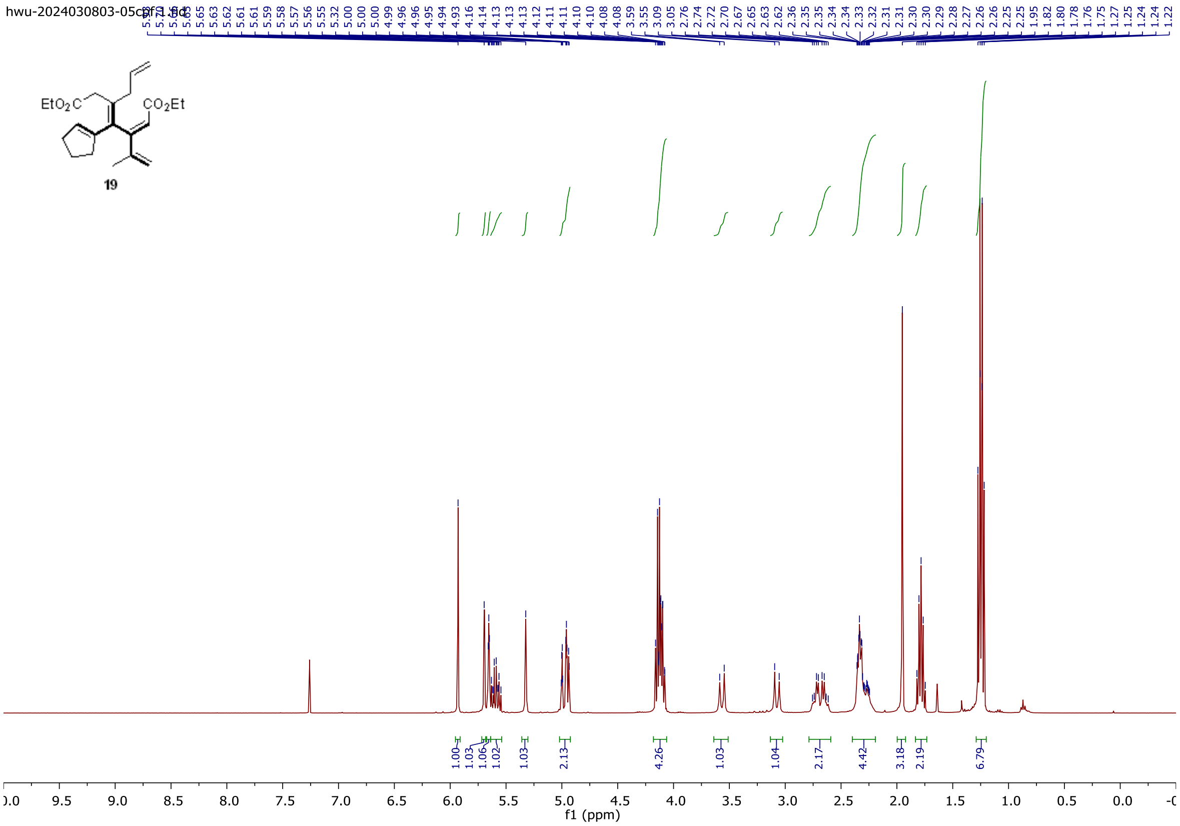
hwu-2024030803-05cpf.1.fid
5.65 5.63 5.62 5.61 5.59 5.58 5.57 5.56 5.55 5.32 5.00 5.00 5.00 4.99 4.96 4.96 4.95 4.94 4.93 4.16 4.14 4.13 4.13 4.13 4.12 4.11 4.11 4.10 4.10 4.08 4.08 3.59 3.55 3.09 3.05 2.76 2.74 2.72 2.70 2.67 2.65 2.63 2.62 2.36 2.35 2.35 2.34 2.34 2.33 2.33 2.32 2.31 2.31 2.30 2.30 2.29 2.28 2.27 2.26 2.26 2.25 2.25 1.95 1.82 1.80 1.78 1.76 1.75 1.27 1.25 1.24 1.24 1.22
EtO2C
CO2Et
19
1.00 1.03 1.06 1.02 1.03 2.13 4.26 1.03 1.04 2.17 4.42 3.18 2.19 6.79
f1 (ppm)

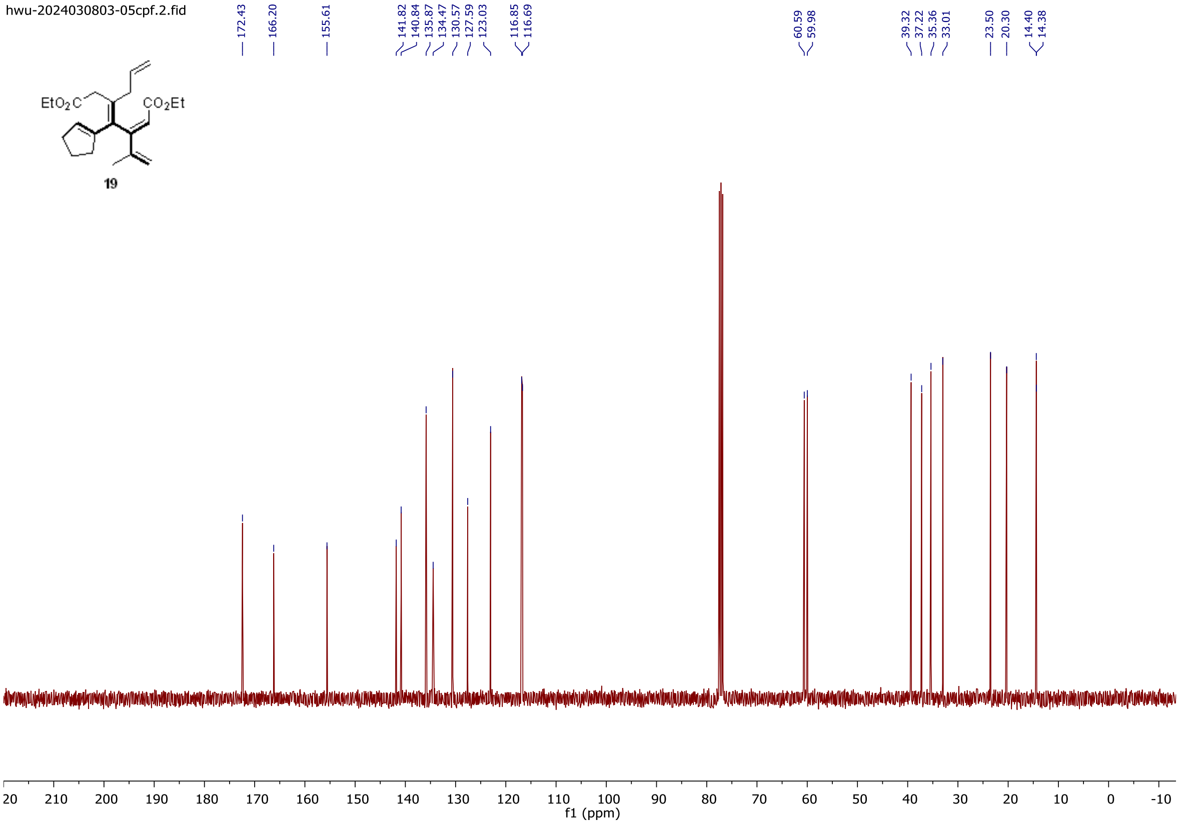
hwu-2024030803-05cpf.2.fid
172.43
166.20
155.61
141.82 140.84 135.87 134.47 130.57 127.59 123.03
116.85 116.69
60.59 59.98
39.32 37.22 35.36 33.01
23.50 20.30
14.40 14.38
EtO2C
CO2Et
19
f1 (ppm)

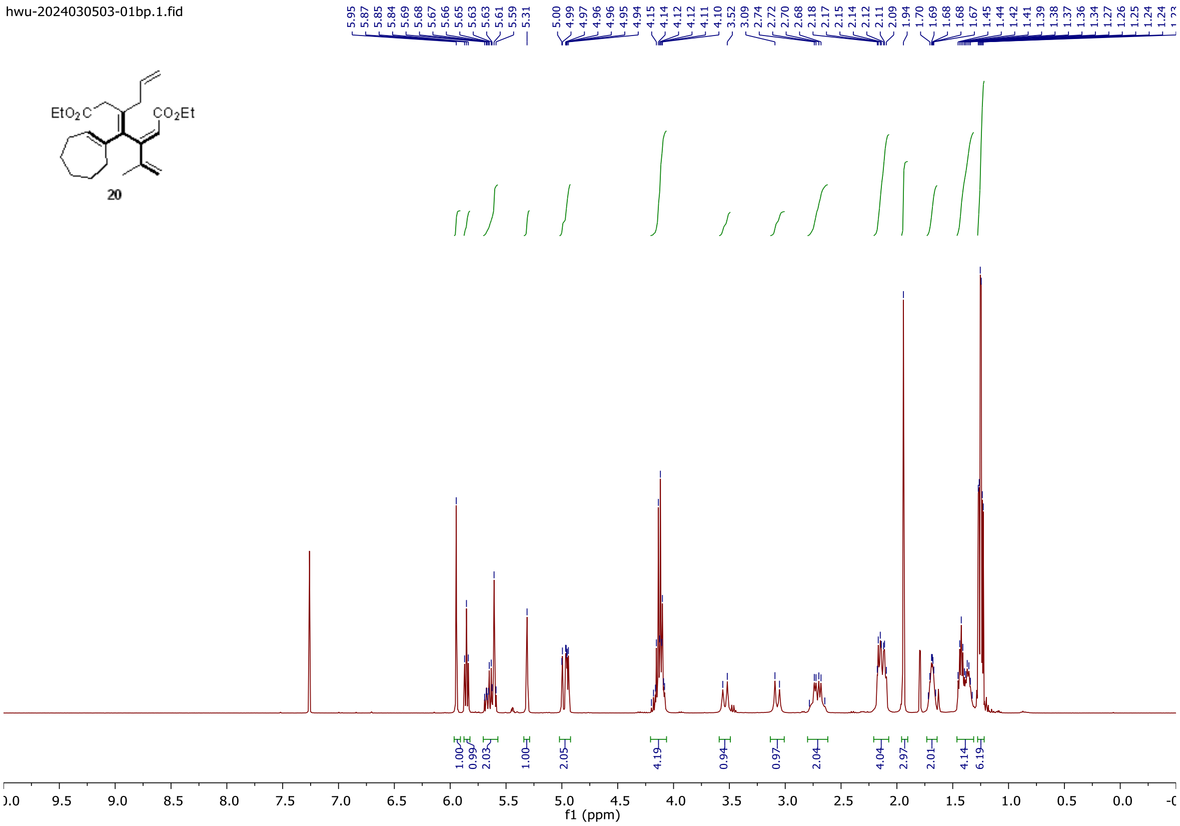
hwu-2024030503-01bp.1.fid
5.95 5.87 5.85 5.84 5.69 5.68 5.67 5.66 5.65 5.63 5.63 5.61 5.59 5.31 5.00 4.99 4.97 4.96 4.96 4.95 4.94 4.15 4.14 4.12 4.12 4.11 4.10 3.52 3.09 2.74 2.72 2.70 2.68 2.18 2.17 2.15 2.14 2.12 2.11 2.09 1.94 1.70 1.69 1.68 1.68 1.67 1.45 1.44 1.42 1.41 1.39 1.38 1.37 1.36 1.34 1.27 1.26 1.25 1.24
EtO_2C
CO_2Et
20
1.00
0.99
2.03
1.00
2.05
4.19
0.94
0.97
2.04
4.04
2.97
2.01
4.14
6.19
9.5 9.0 8.5 8.0 7.5 7.0 6.5 6.0 5.5 5.0 4.5 4.0 3.5 3.0 2.5 2.0 1.5 1.0 0.5 0.0
f1 (ppm)

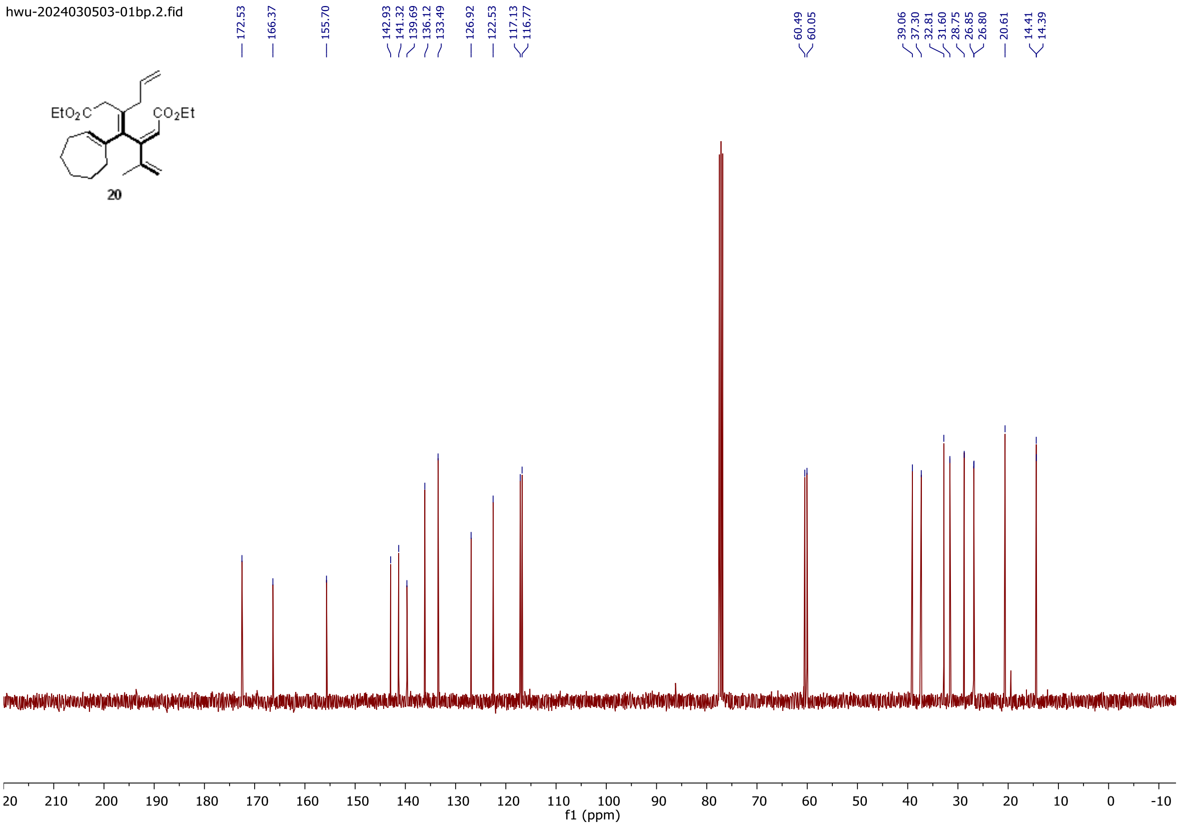
hwu-2024030503-01bp.2.fid
172.53 166.37 155.70 142.93 141.32 139.69 136.12 133.49 126.92 122.53 117.13 116.77 60.49 60.05 39.06 37.30 32.81 31.60 28.75 26.85 26.80 20.61 14.41 14.39
EtO_2C
CO_2Et
20
210 200 190 180 170 160 150 140 130 120 110 100 90 80 70 60 50 40 30 20 10 0 -10
f1 (ppm)

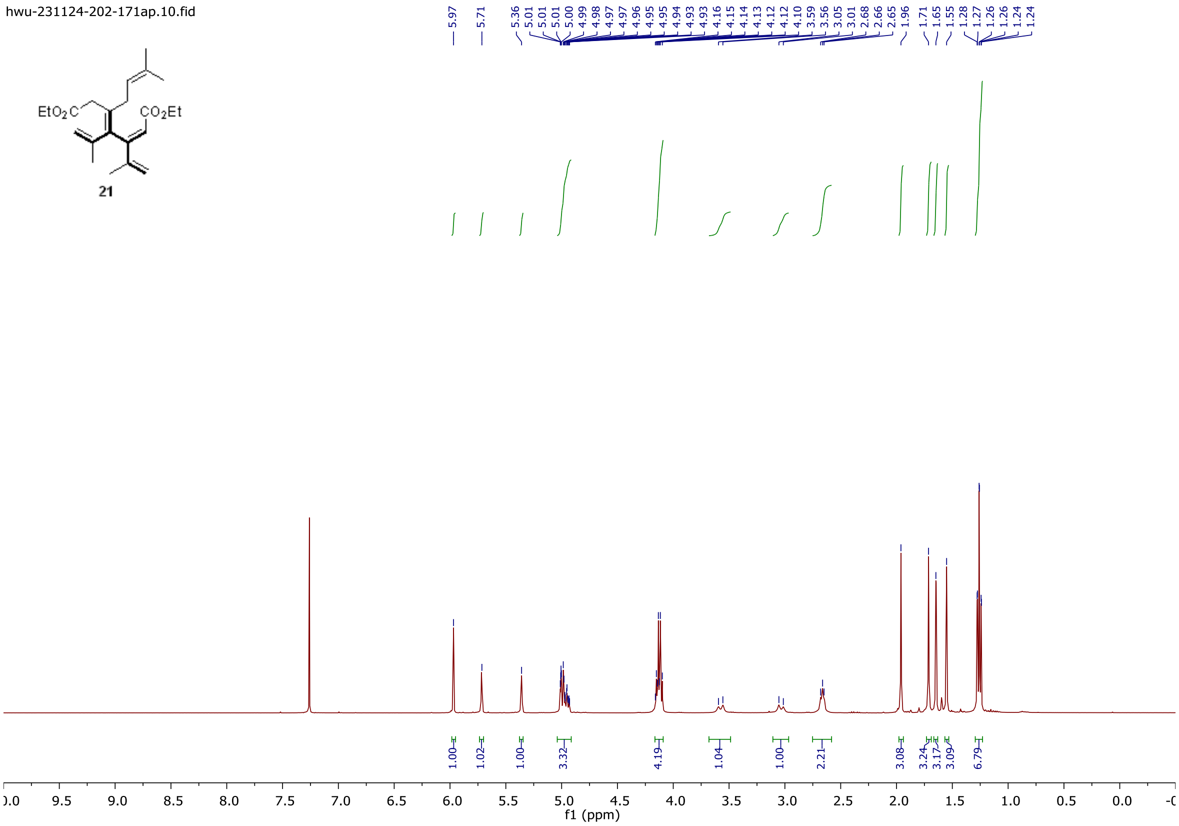
hwu-231124-202-171ap.10.fid
EtO2C
CO2Et
21
5.97
5.71
5.36
5.01
5.01
5.01
5.00
4.99
4.98
4.97
4.97
4.96
4.95
4.95
4.94
4.93
4.93
4.16
4.15
4.14
4.13
4.12
4.10
3.59
3.56
3.05
3.01
2.68
2.66
2.65
1.96
1.71
1.65
1.55
1.28
1.27
1.26
1.26
1.24
1.24
1.00
1.02
1.00
3.32
4.19
1.04
1.00
2.21
3.08
3.24
3.17
3.09
6.79
f1 (ppm)

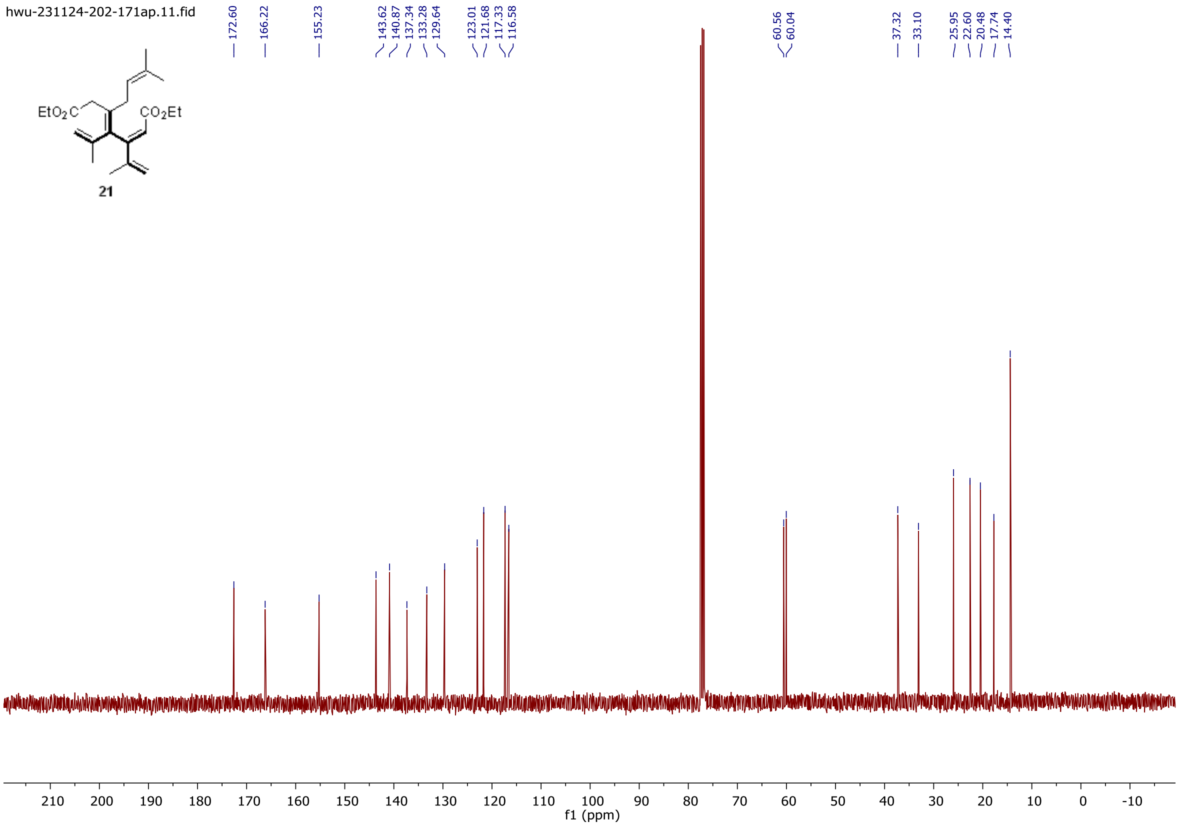
hwu-231124-202-171ap.11.fid
EtO2C
CO2Et
21
172.60
166.22
155.23
143.62
140.87
137.34
133.28
129.64
123.01
121.68
117.33
116.58
60.56
60.04
37.32
33.10
25.95
22.60
20.48
17.74
14.40
f1 (ppm)

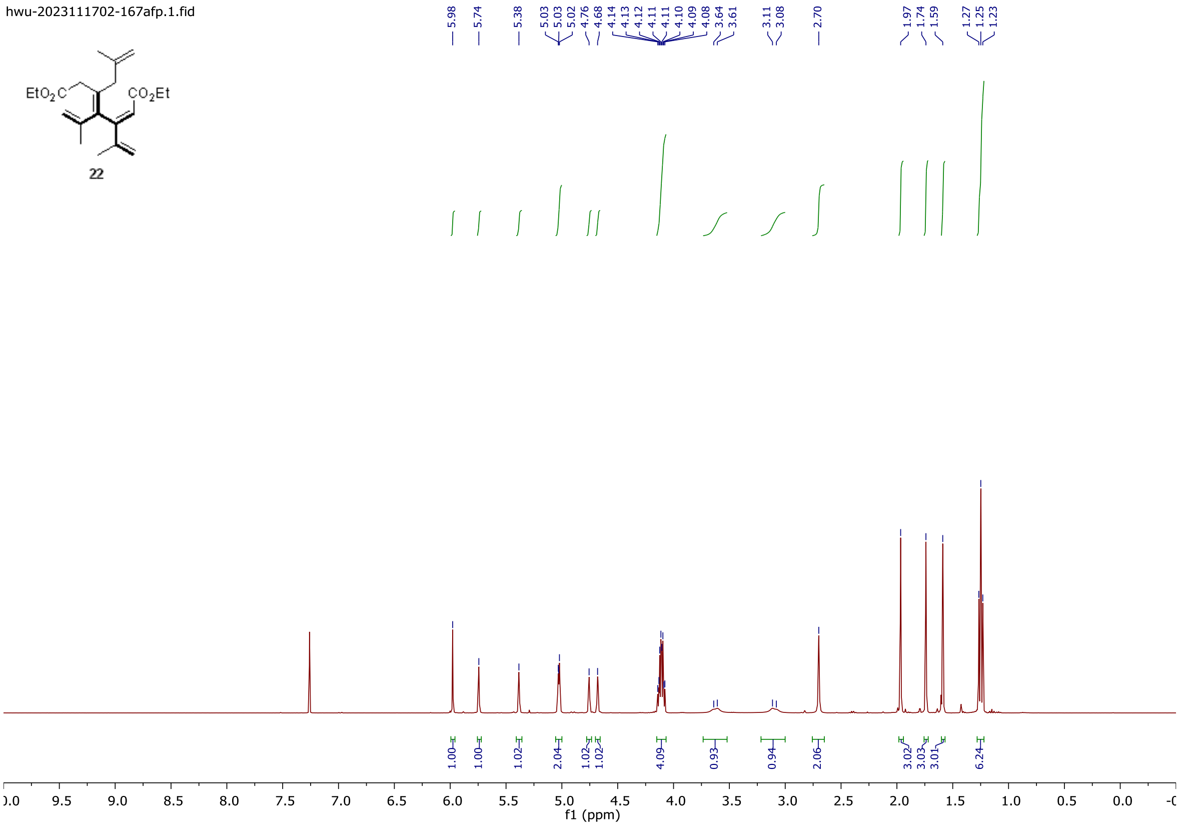
hwu-2023111702-167afp.1.fid
EtO2C
CO2Et
22
5.98
5.74
5.38
5.03
5.03
5.02
4.76
4.68
4.14
4.13
4.12
4.11
4.11
4.11
4.10
4.09
4.08
3.64
3.61
3.11
3.08
2.70
1.97
1.74
1.59
1.27
1.25
1.23
1.00
1.00
1.02
2.04
1.02
1.02
4.09
0.93
0.94
2.06
3.02
3.03
3.01
6.24
f1 (ppm)

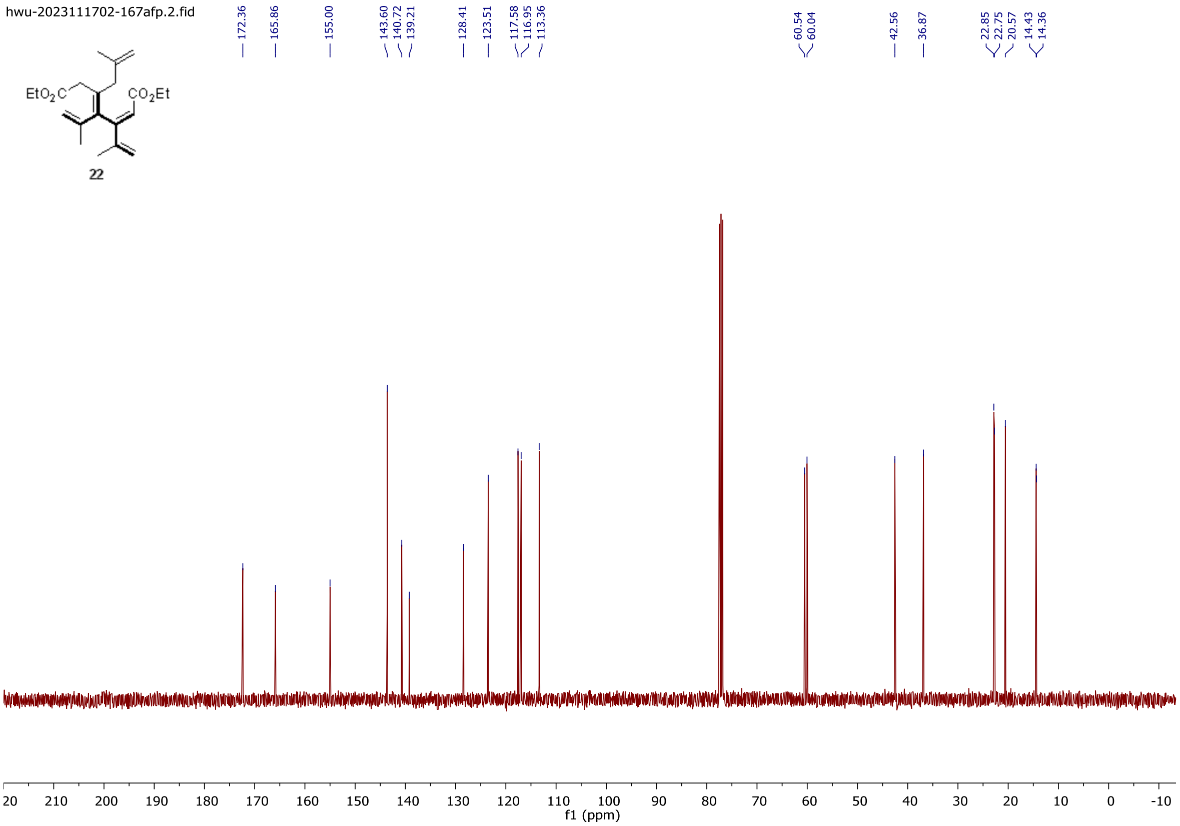
hwu-2023111702-167afp.2.fid
EtO2C
CO2Et
22
172.36
165.86
155.00
143.60
140.72
139.21
128.41
123.51
117.58
116.95
113.36
60.54
60.04
42.56
36.87
22.85
22.75
20.57
14.43
14.36
f1 (ppm)

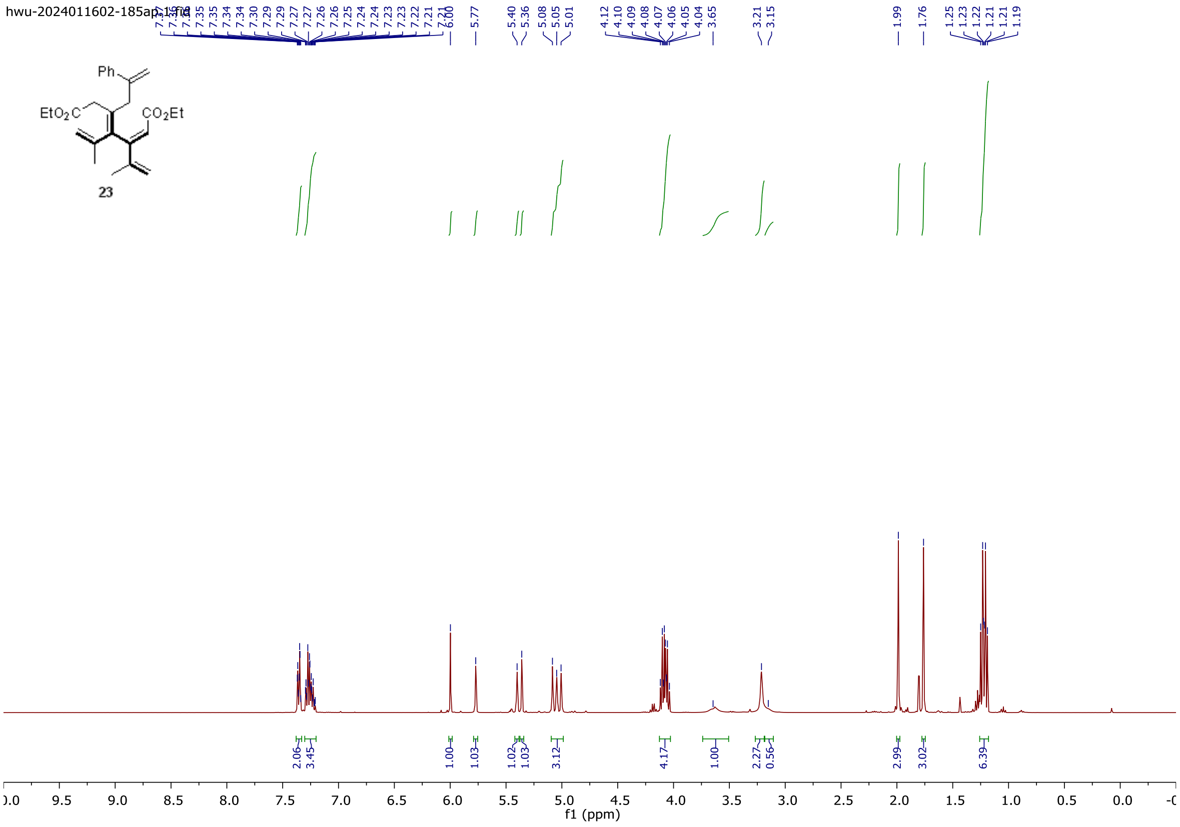

23

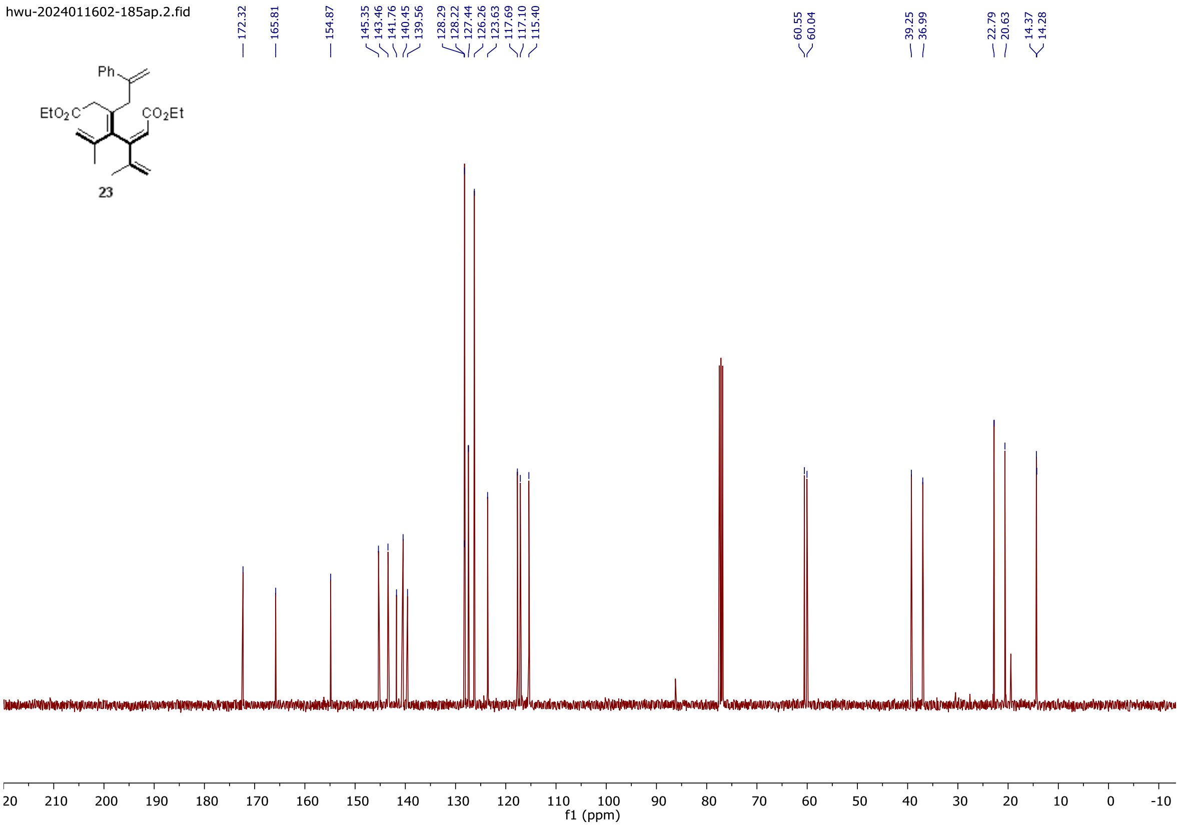

23

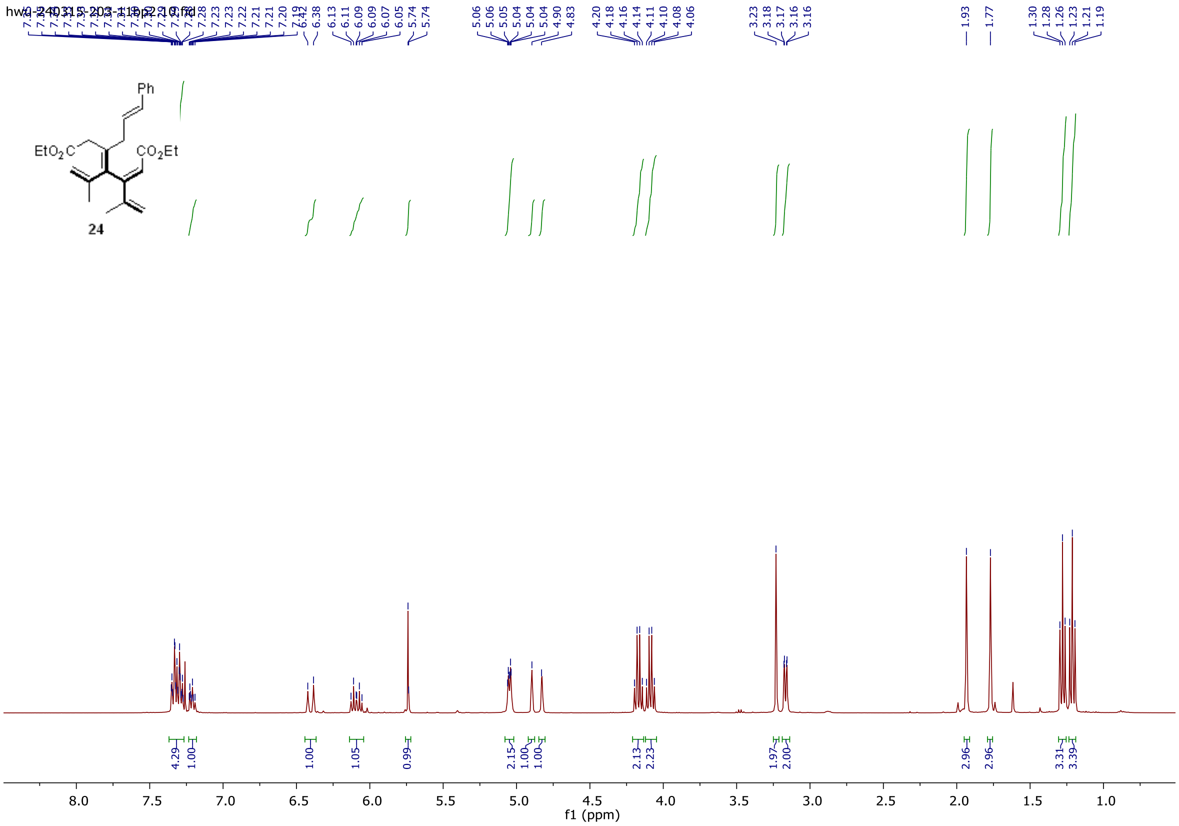

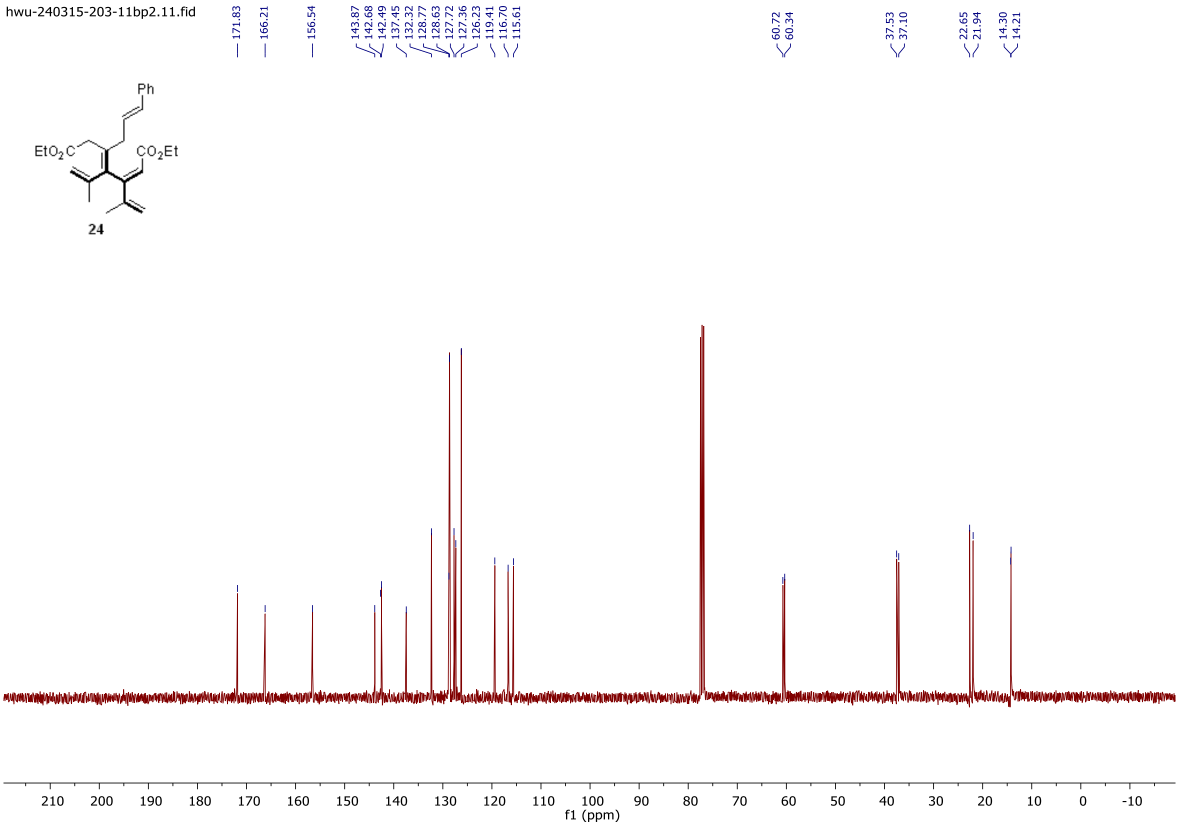

hwu-2023110802-162pf1.1.fid

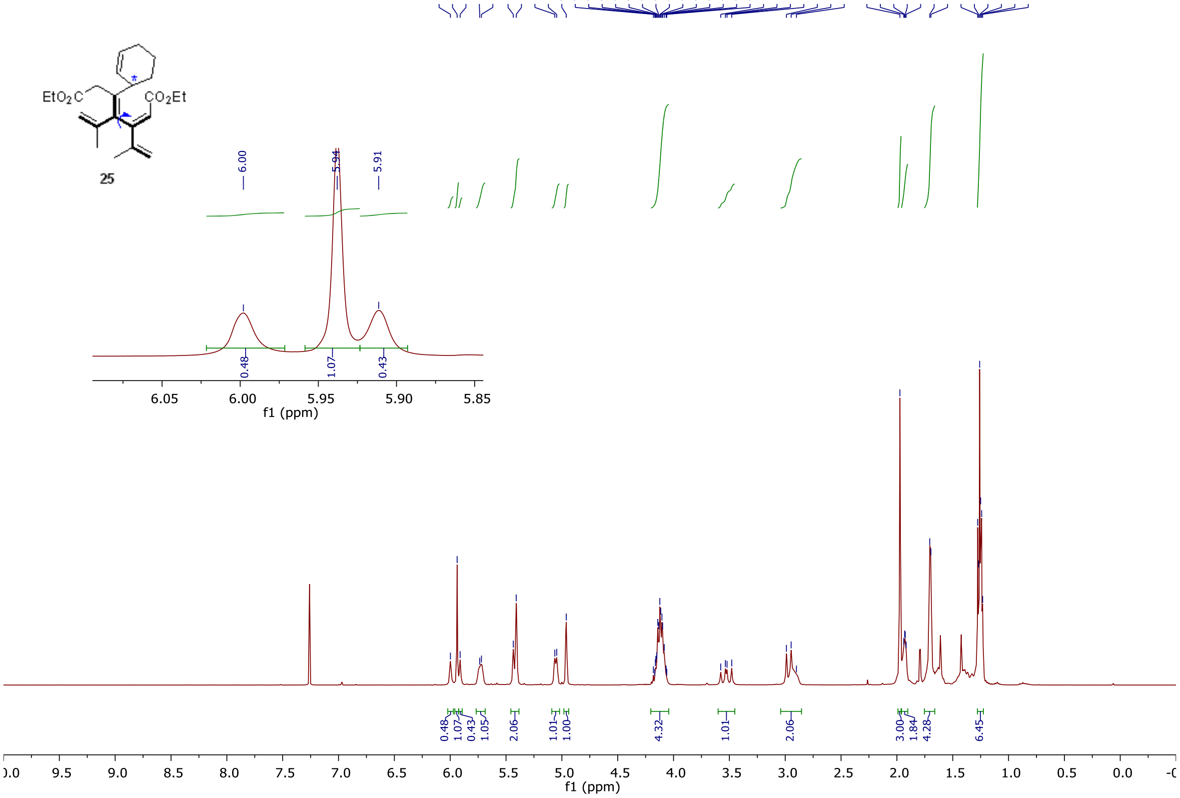

25

hwu-2023110802-162pf1.3.fid

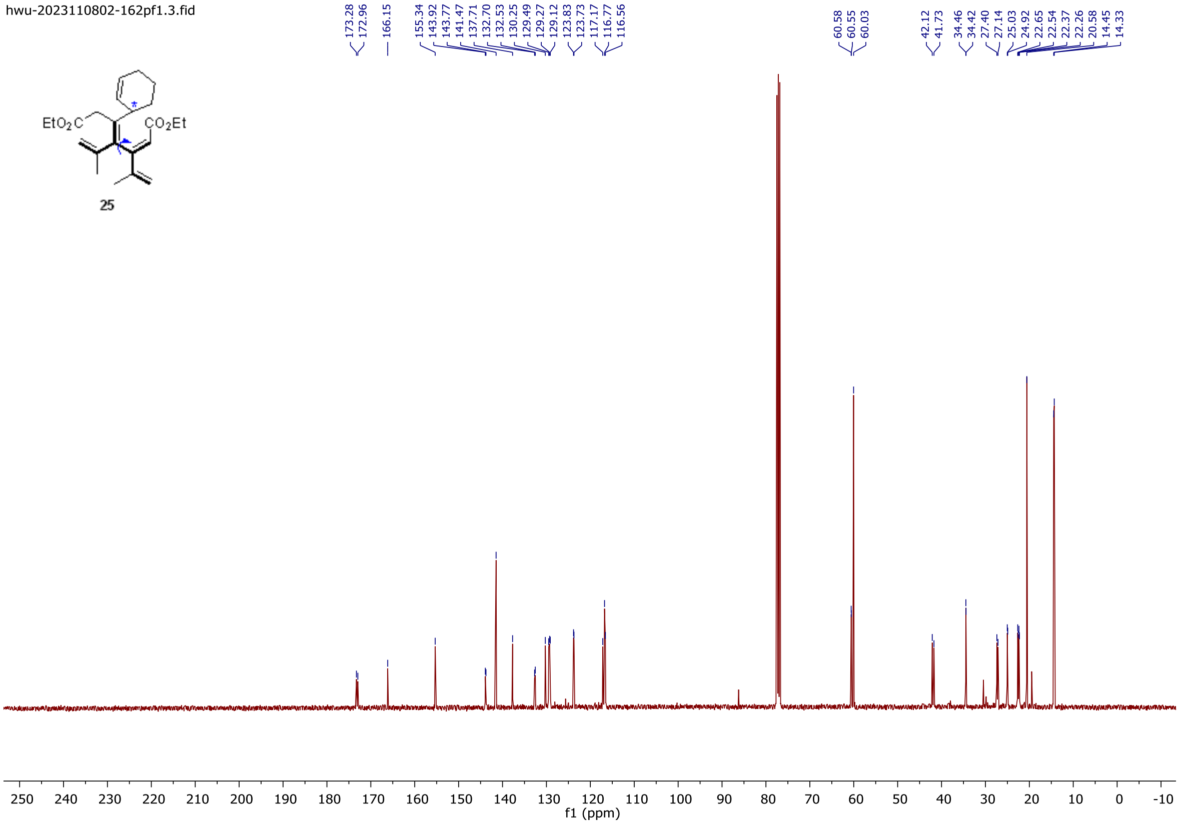

25

hwu-230629-202-91p1.10.fid

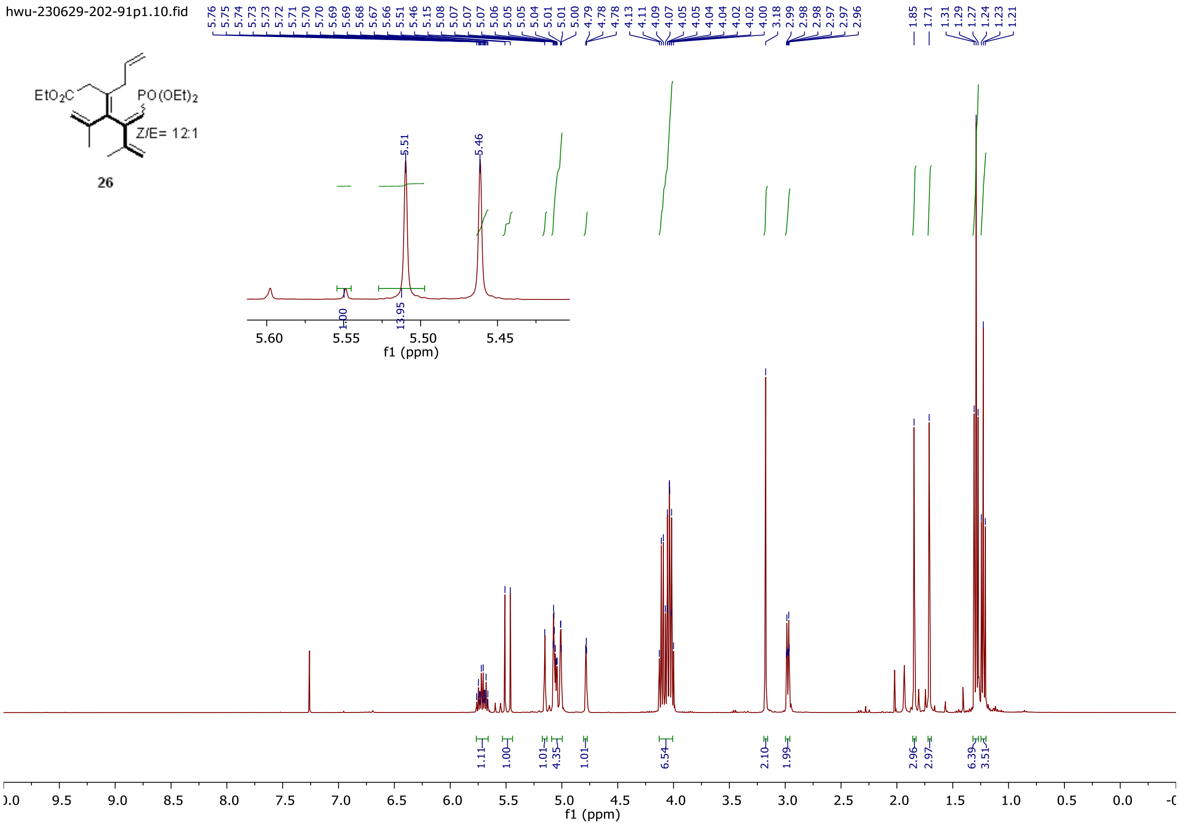

hwu-230629-202-91p1.11.fid

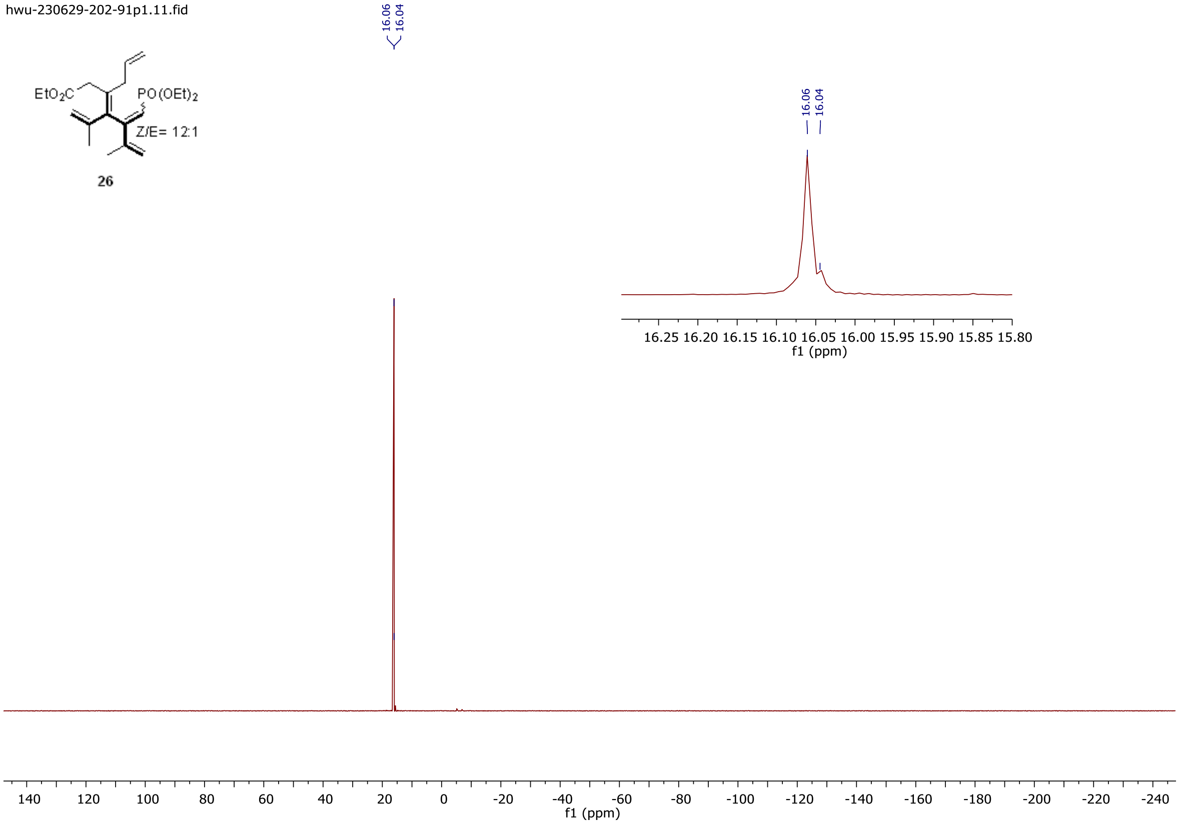

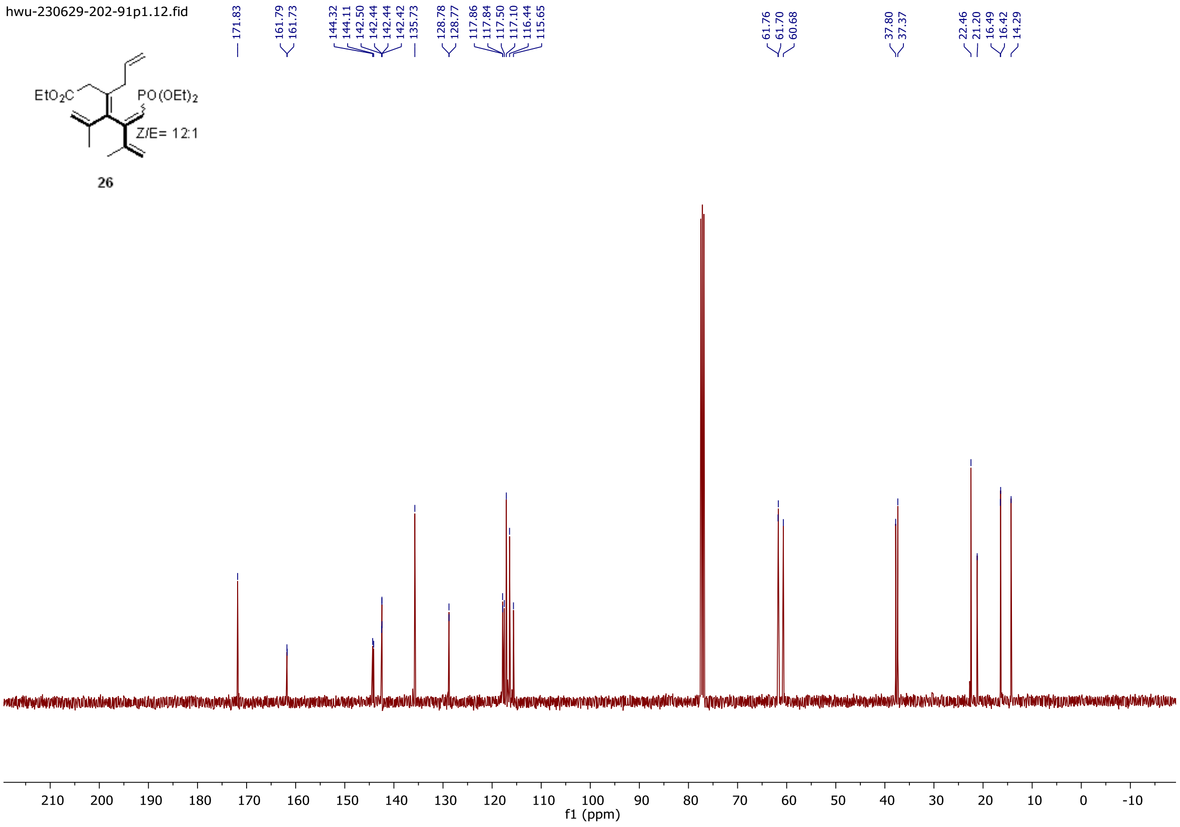
hwu-230629-202-91p1.12.fid
171.83
161.79
161.73
144.32
144.11
142.50
142.44
142.44
142.42
135.73
128.78
128.77
117.86
117.84
117.50
117.10
116.44
115.65
61.76
61.70
60.68
37.80
37.37
22.46
21.20
16.49
16.42
14.29
EtO_2C
$PO(OEt)_2$
Z/E= 12:1
26
f1 (ppm)

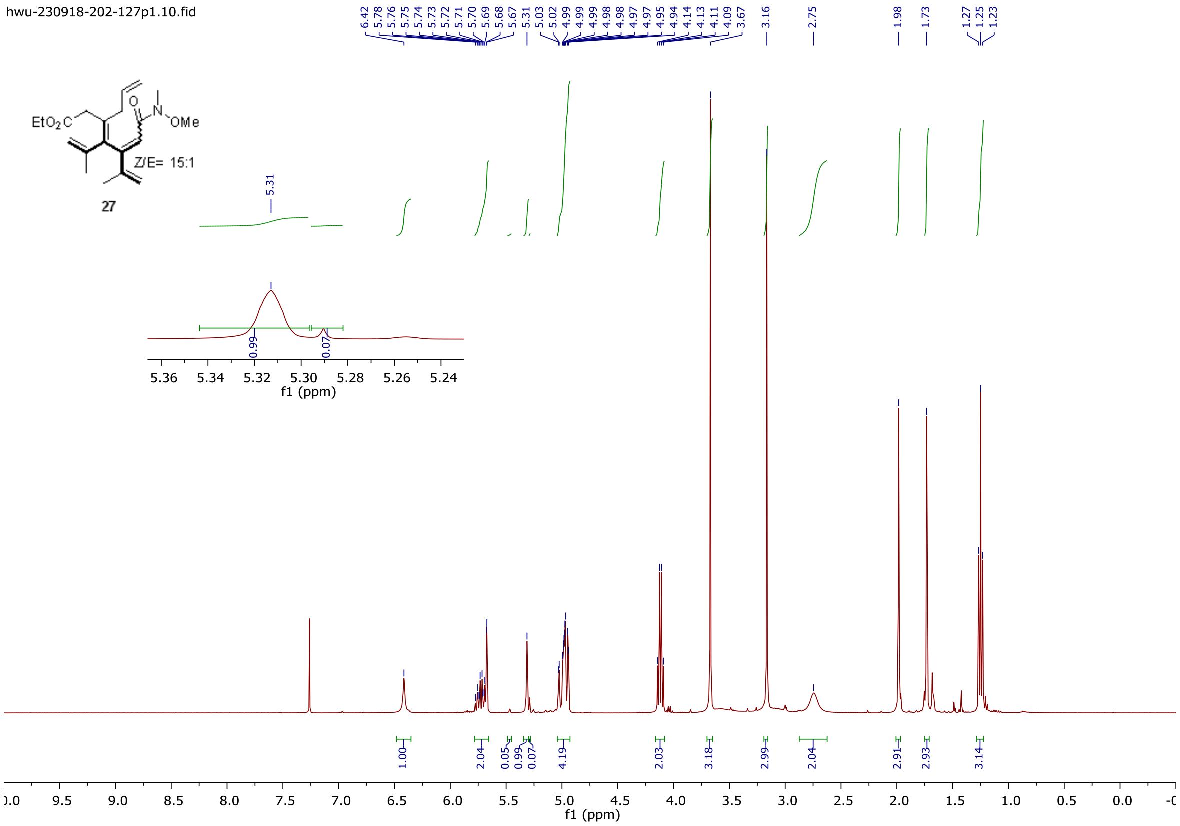
hwu-230918-202-127p1.10.fid
6.42 5.78 5.76 5.75 5.74 5.73 5.72 5.71 5.70 5.69 5.68 5.67 5.31 5.03 5.02 4.99 4.99 4.99 4.98 4.98 4.97 4.97 4.95 4.94 4.14 4.13 4.11 4.09 3.67 3.16 2.75 1.98 1.73 1.27 1.25 1.23
EtO2C
O
N
OMe
Z/E= 15:1
27
5.31
0.99
0.07
f1 (ppm)
1.00
2.04
0.05
0.99
0.07
4.19
2.03
3.18
2.99
2.04
2.91
2.93
3.14
f1 (ppm)

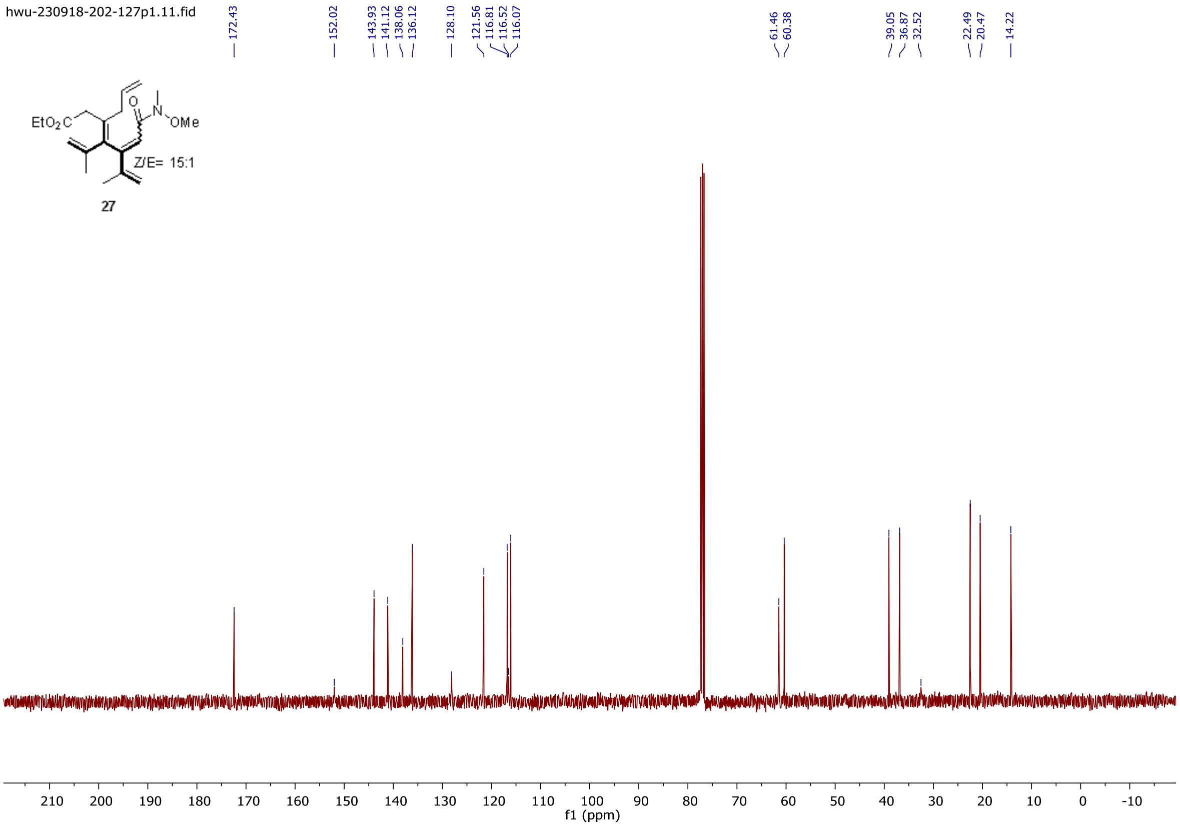
hwu-230918-202-127p1.11.fid
172.43 152.02 143.93 141.12 138.06 136.12 128.10 121.56 116.81 116.52 116.07 61.46 60.38 39.05 36.87 32.52 22.49 20.47 14.22
EtO2C
O
N
OMe
Z/E= 15:1
27
f1 (ppm)

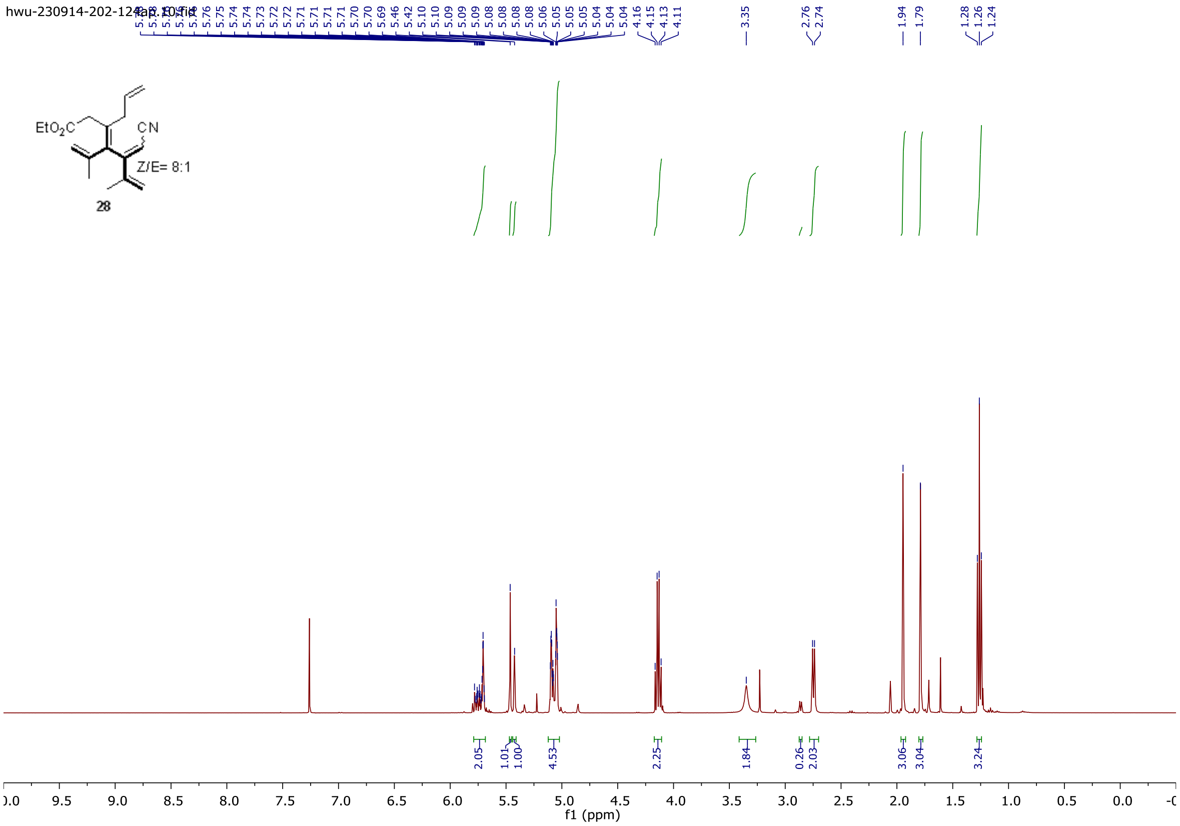
EtO2C
CN
Z/E= 8:1
28
f1 (ppm)

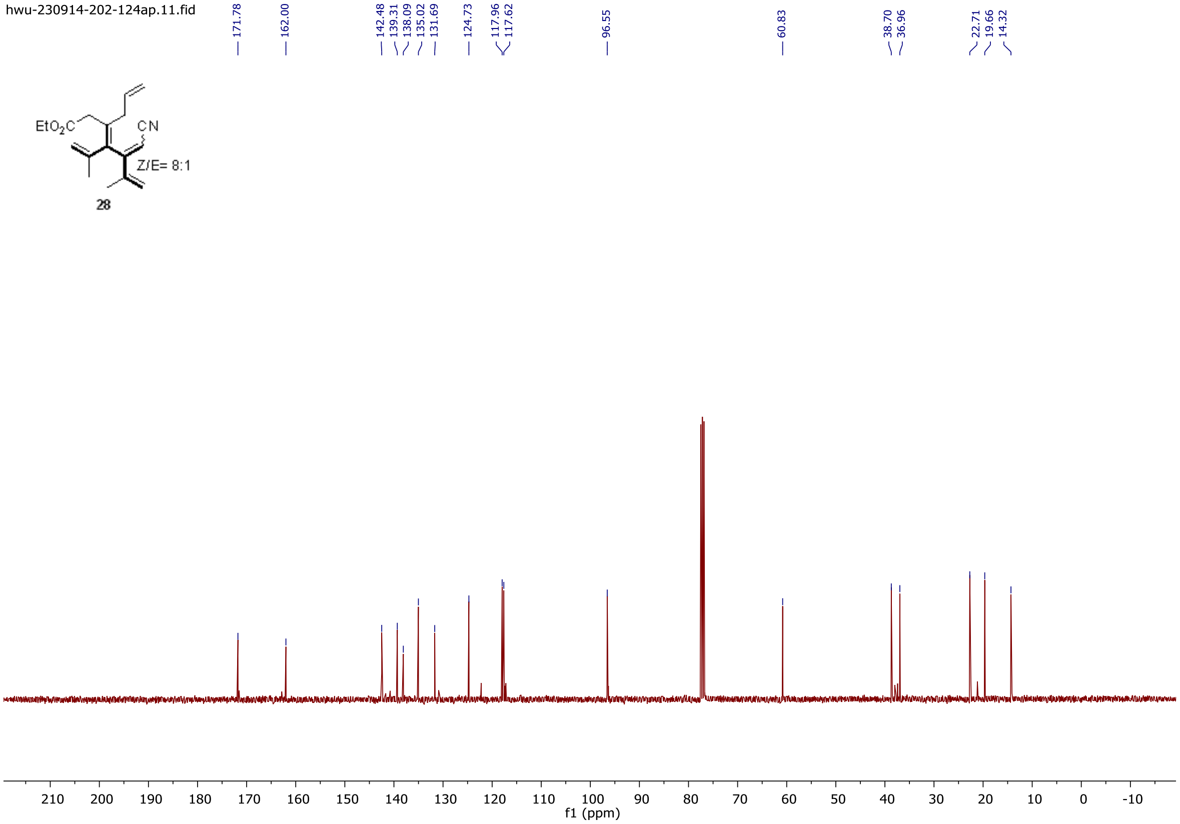
hwu-230914-202-124ap.11.fid
171.78
162.00
142.48
139.31
138.09
135.02
131.69
124.73
117.96
117.62
96.55
60.83
38.70
36.96
22.71
19.66
14.32
EtO2C
CN
Z/E= 8:1
28
f1 (ppm)

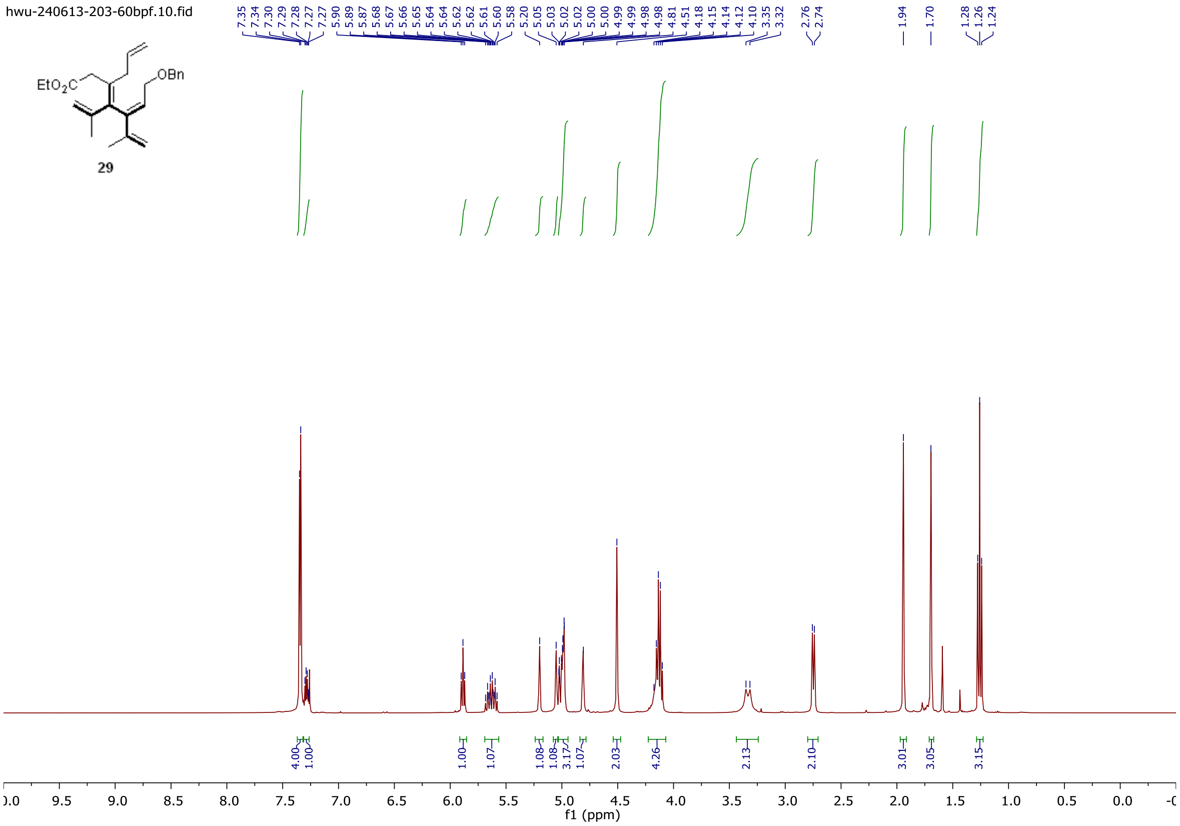
hwu-240613-203-60bpf.10.fid
7.35 7.34 7.30 7.29 7.28 7.27 7.27 5.90 5.89 5.87 5.68 5.67 5.66 5.65 5.64 5.64 5.62 5.62 5.61 5.60 5.58 5.20 5.05 5.03 5.02 5.02 5.02 5.00 5.00 4.99 4.99 4.98 4.98 4.98 4.81 4.51 4.18 4.15 4.14 4.12 4.10 3.35 3.32 2.76 2.74 1.94 1.70 1.28 1.26 1.24
EtO2C
OBn
29
4.00 1.00 1.00 1.07 1.08 1.08 3.17 1.07 2.03 4.26 2.13 2.10 3.01 3.05 3.15
f1 (ppm)

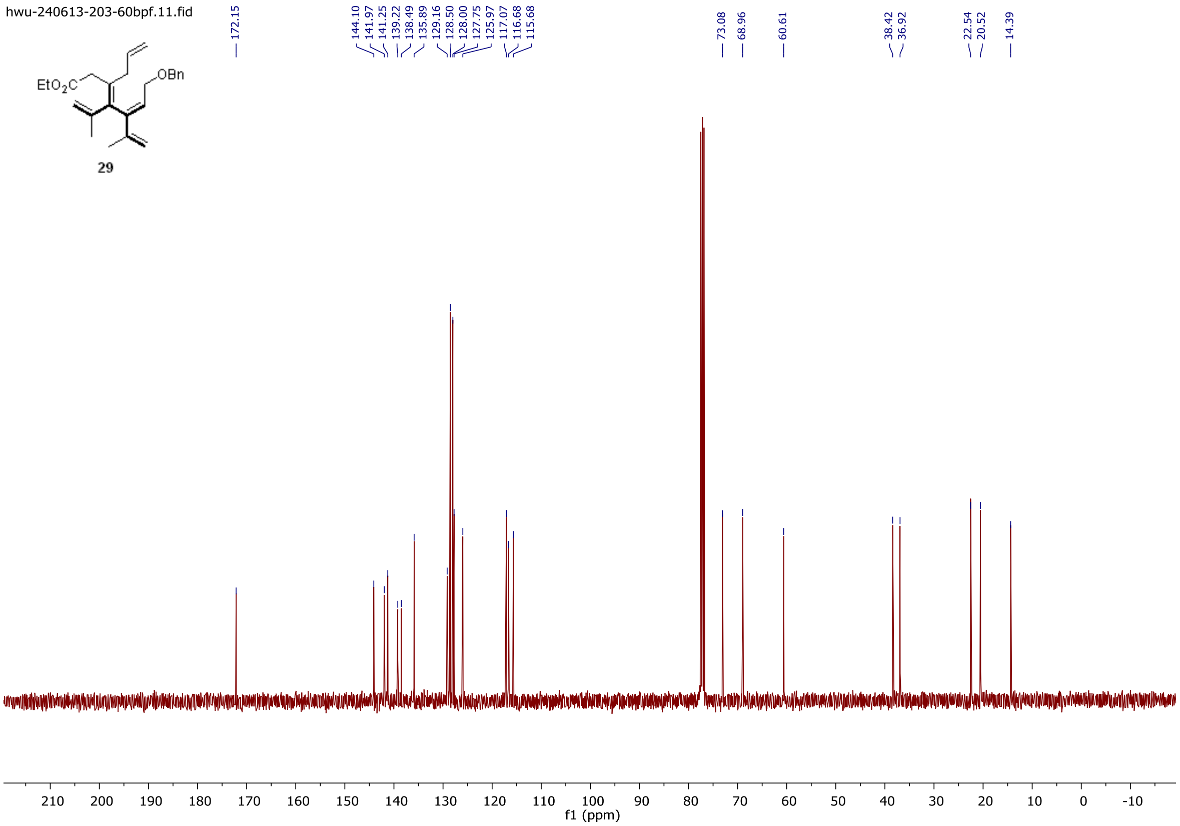
hwu-240613-203-60bpf.11.fid
172.15 144.10 141.97 141.25 139.22 138.49 135.89 129.16 128.50 128.00 127.75 125.97 117.07 116.68 115.68 73.08 68.96 60.61 38.42 36.92 22.54 20.52 14.39
EtO2C
OBn
29
f1 (ppm)

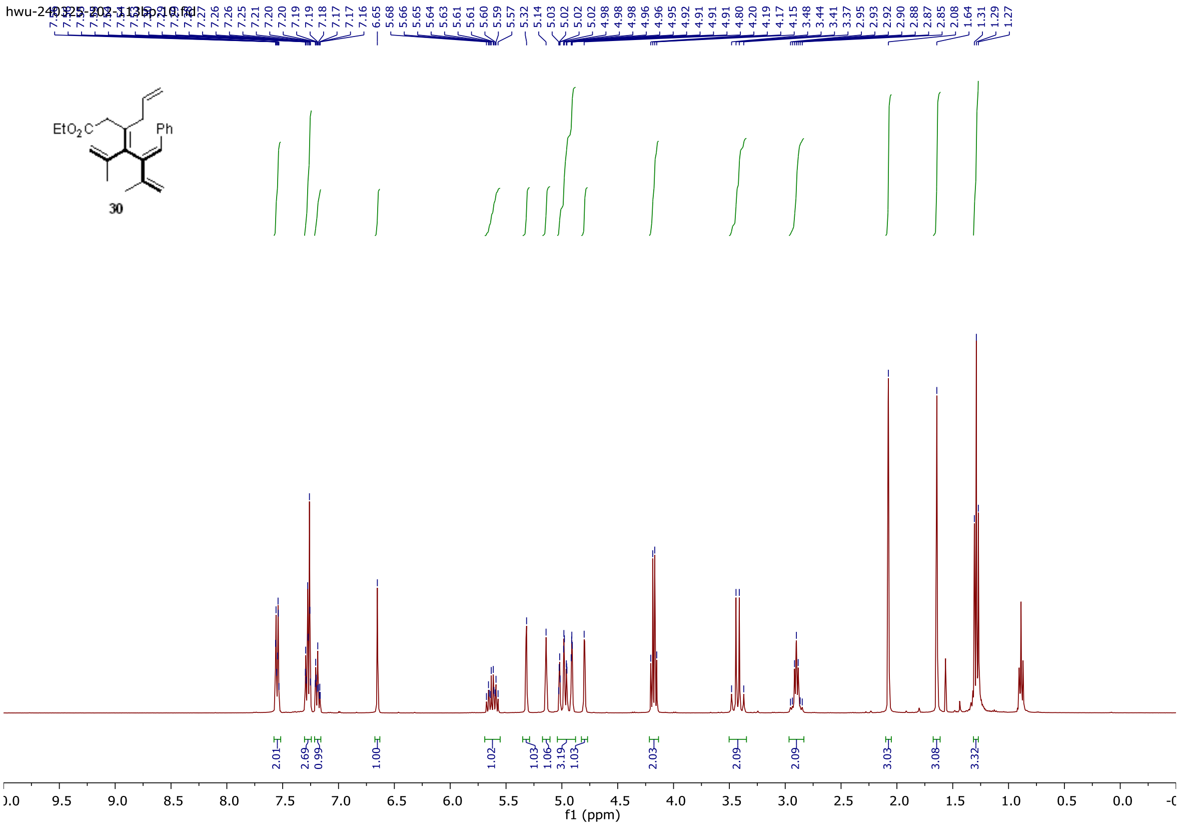
hwu-240325-202-113bp.10.fid
7.26 7.25 7.21 7.20 7.19 7.19 7.18 7.17 7.17 7.16 6.65 5.68 5.66 5.65 5.64 5.63 5.61 5.61 5.60 5.59 5.57 5.32 5.14 5.03 5.02 5.02 5.02 4.98 4.98 4.98 4.96 4.96 4.95 4.92 4.91 4.91 4.91 4.80 4.20 4.19 4.17 4.15 3.48 3.44 3.41 3.37 2.95 2.93 2.92 2.90 2.88 2.87 2.85 2.08 1.64 1.31 1.29 1.27
EtO2C
Ph
30
2.01 2.69 0.99 1.00 1.02 1.03 1.06 3.19 1.03 2.03 2.09 2.09 3.03 3.08 3.32
f1 (ppm)

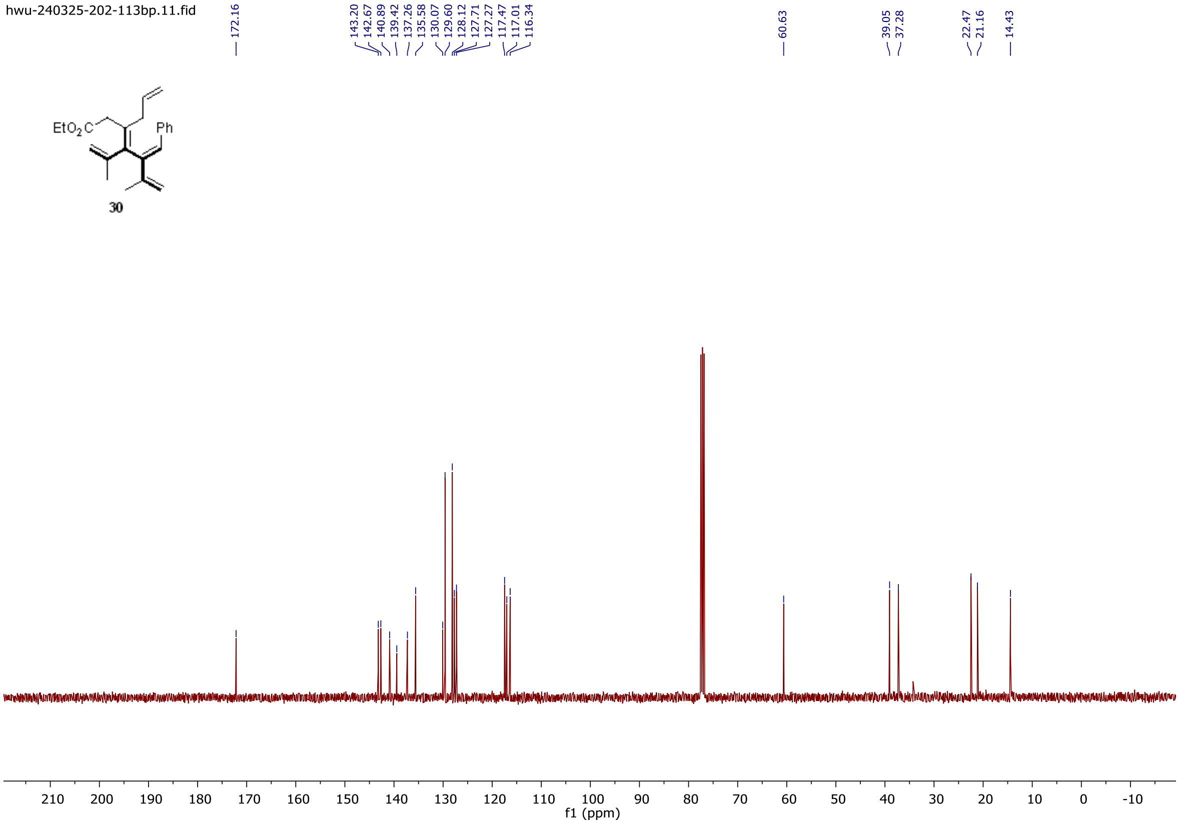
hwu-240325-202-113bp.11.fid
172.16
143.20 142.67 140.89 139.42 137.26 135.58 130.07 129.60 128.12 127.71 127.27 117.47 117.01 116.34
60.63
39.05 37.28
22.47 21.16
14.43
EtO2C
Ph
30
f1 (ppm)

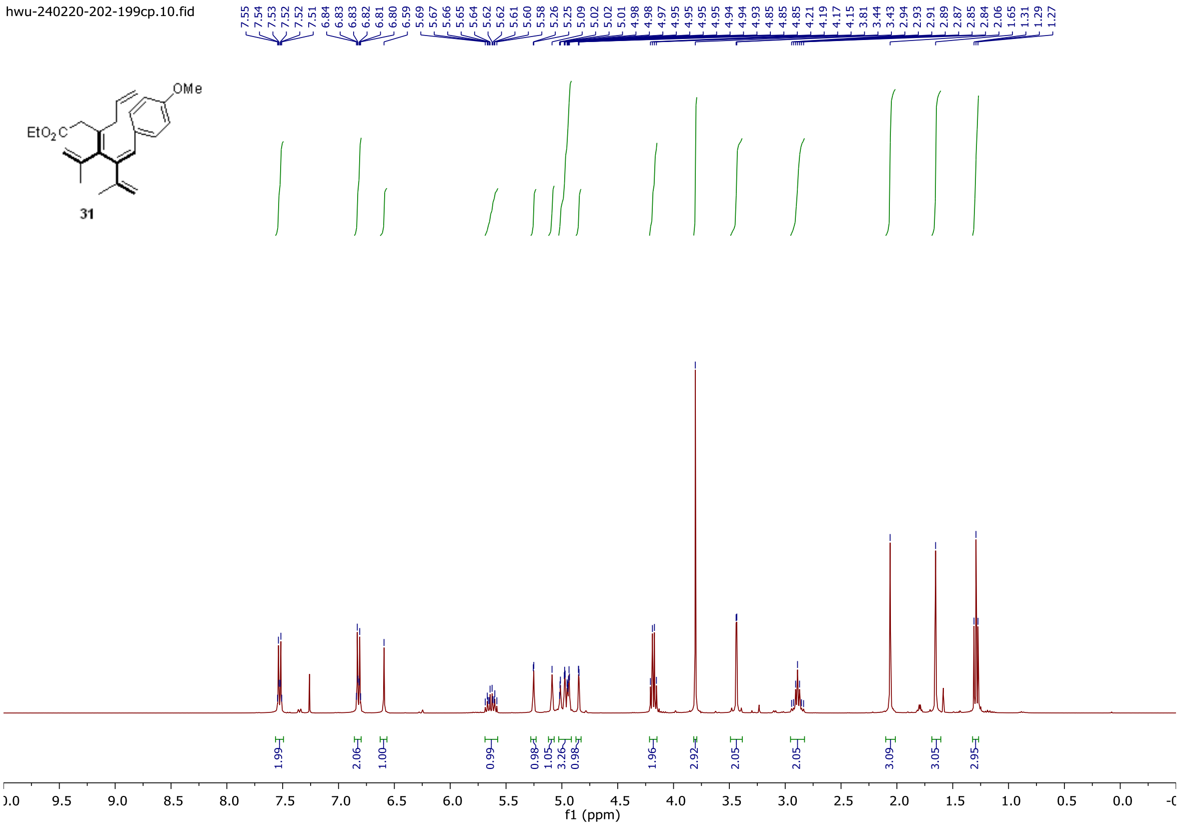
hwu-240220-202-199cp.10.fid
7.55 7.54 7.53 7.52 7.52 7.51 6.84 6.83 6.83 6.82 6.81 6.80 6.59 5.69 5.67 5.66 5.65 5.64 5.62 5.62 5.61 5.60 5.58 5.26 5.25 5.09 5.02 5.01 4.98 4.98 4.97 4.95 4.95 4.95 4.94 4.94 4.93 4.85 4.85 4.85 4.21 4.19 4.17 4.15 3.81 3.44 3.43 2.94 2.93 2.91 2.89 2.87 2.85 2.84 2.06 1.65 1.31 1.29 1.27
OMe
EtO_2C
31
1.99 2.06 1.00 0.99 0.98 1.05 3.26 0.98 1.96 2.92 2.05 2.05 3.09 3.05 2.95
f1 (ppm)

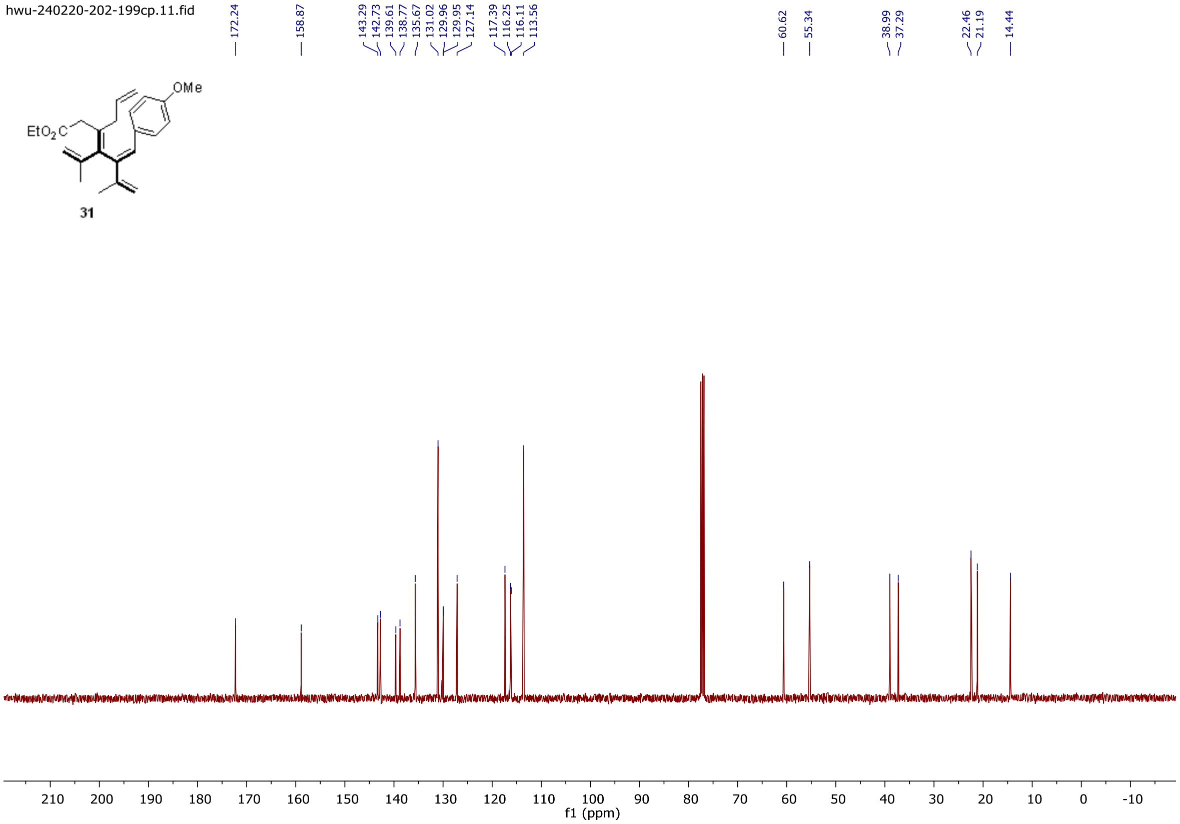
hwu-240220-202-199cp.11.fid
172.24 158.87 143.29 142.73 139.61 138.77 135.67 131.02 129.96 129.95 127.14 117.39 116.25 116.11 113.56 60.62 55.34 38.99 37.29 22.46 21.19 14.44
OMe
EtO_2C
31
f1 (ppm)

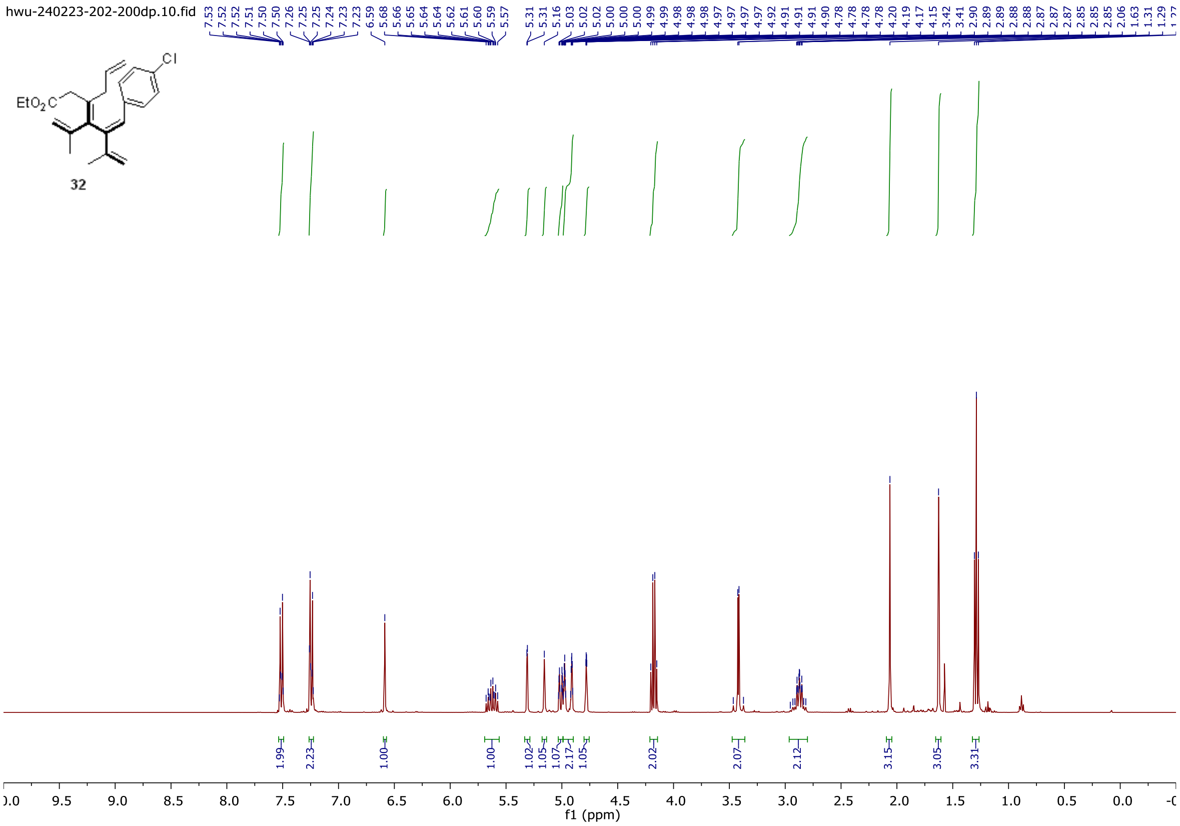
hwu-240223-202-200dp.10.fid
7.53 7.52 7.52 7.51 7.50 7.50 7.26 7.25 7.25 7.24 7.23 7.23 6.59 5.68 5.66 5.65 5.64 5.64 5.62 5.61 5.60 5.59 5.57 5.31 5.31 5.16 5.03 5.02 5.02 5.00 5.00 5.00 4.99 4.99 4.98 4.98 4.98 4.97 4.97 4.97 4.97 4.92 4.91 4.91 4.91 4.90 4.78 4.78 4.78 4.78 4.20 4.19 4.17 4.15 3.42 3.41 2.90 2.89 2.89 2.88 2.88 2.87 2.87 2.87 2.85 2.85 2.85 2.06 1.63 1.31 1.29 [illegible]
EtO2C
Cl
32
1.99 2.23 1.00 1.00 1.02 1.05 1.07 2.17 1.05 2.02 2.07 2.12 3.15 3.05 3.31
f1 (ppm)

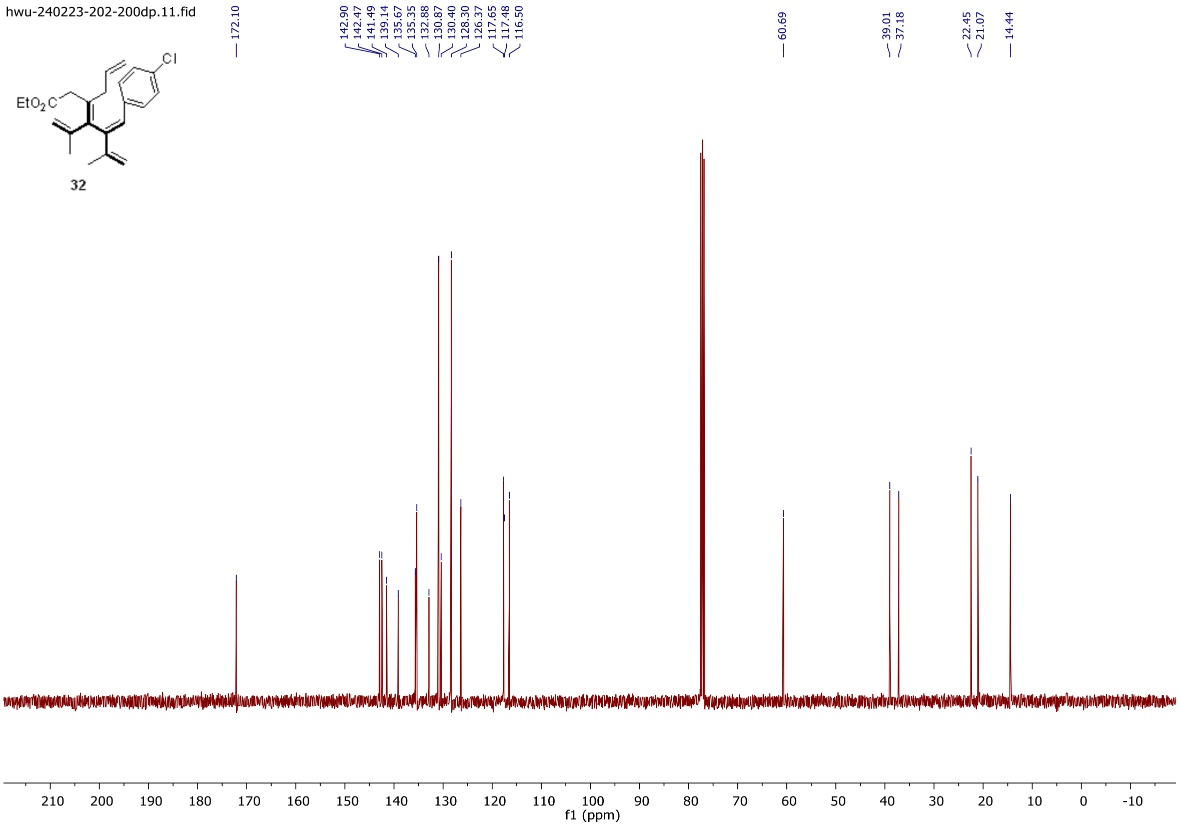
hwu-240223-202-200dp.11.fid
172.10
142.90 142.47 141.49 139.14 135.67 135.35 132.88 130.87 130.40 128.30 126.37 117.65 117.48 116.50
60.69
39.01 37.18
22.45 21.07
14.44
EtO2C
Cl
32
f1 (ppm)

hwu-240223-202-200cp.10.fid

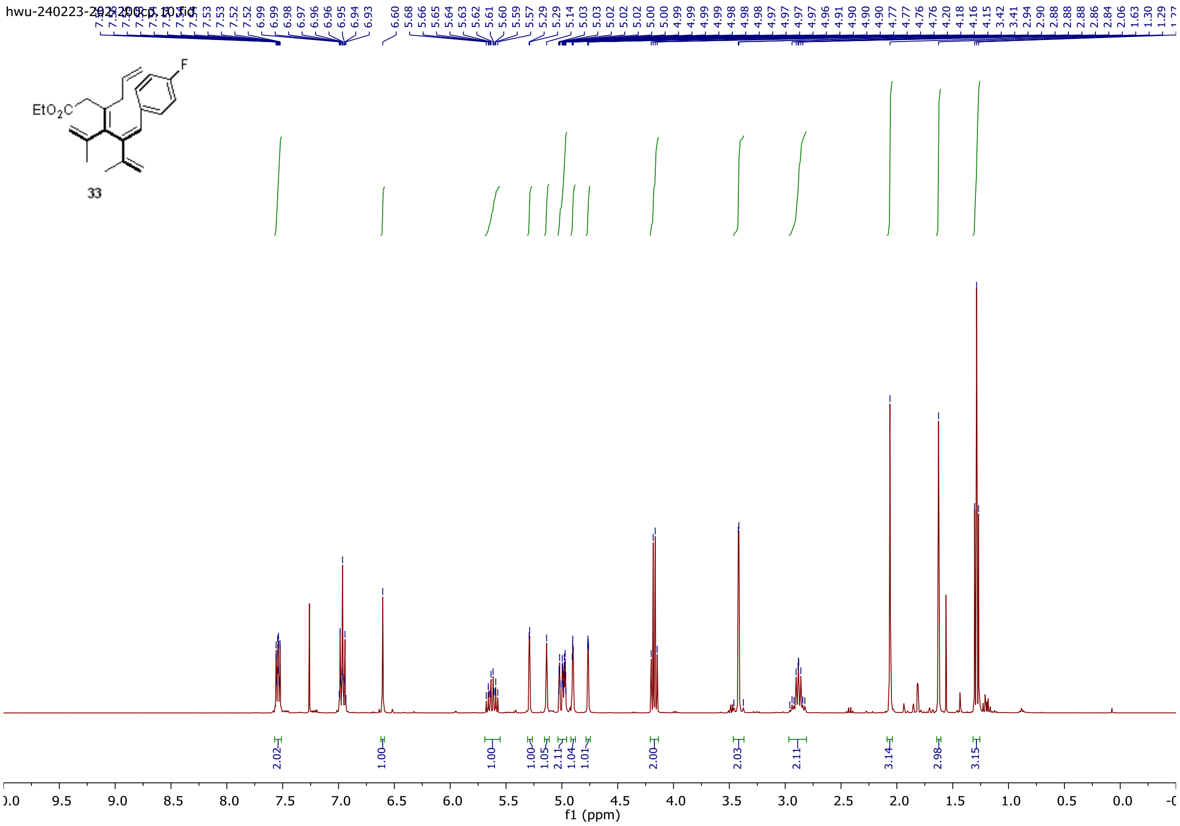

hwu-240223-202-200cp.11.fid

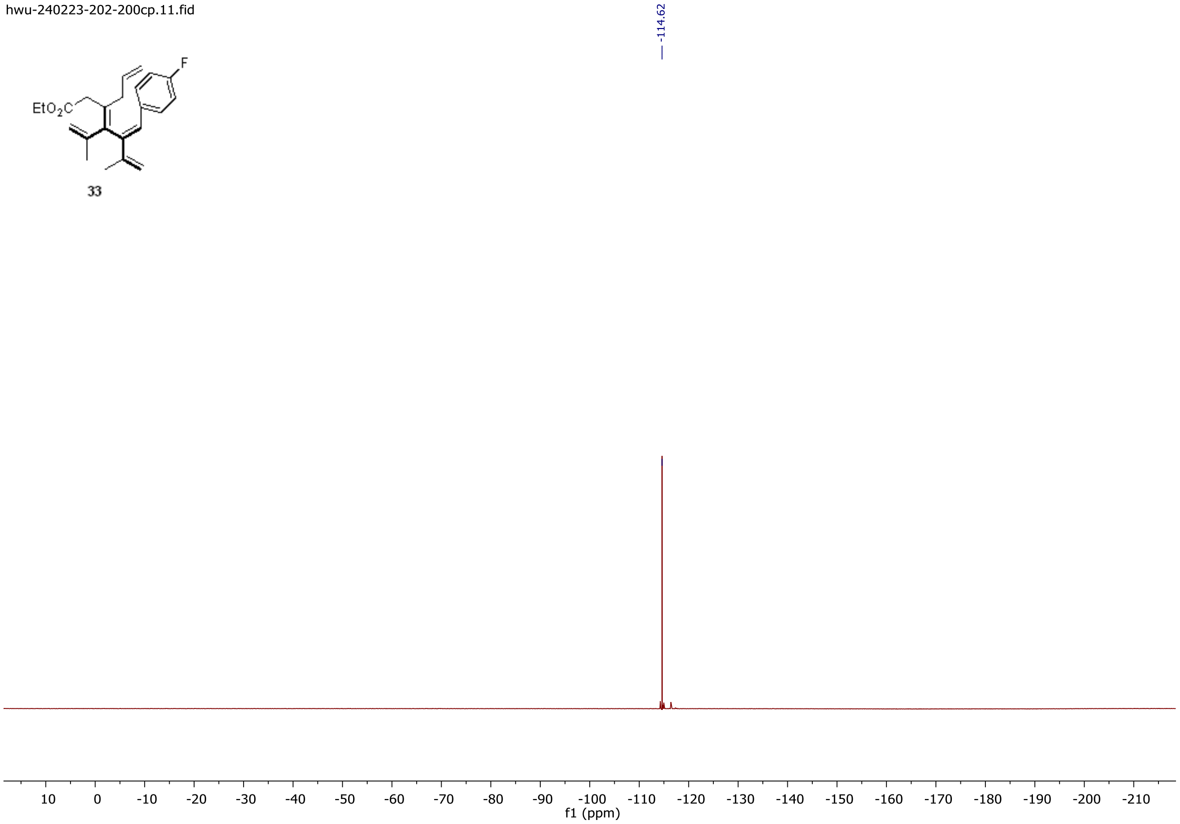

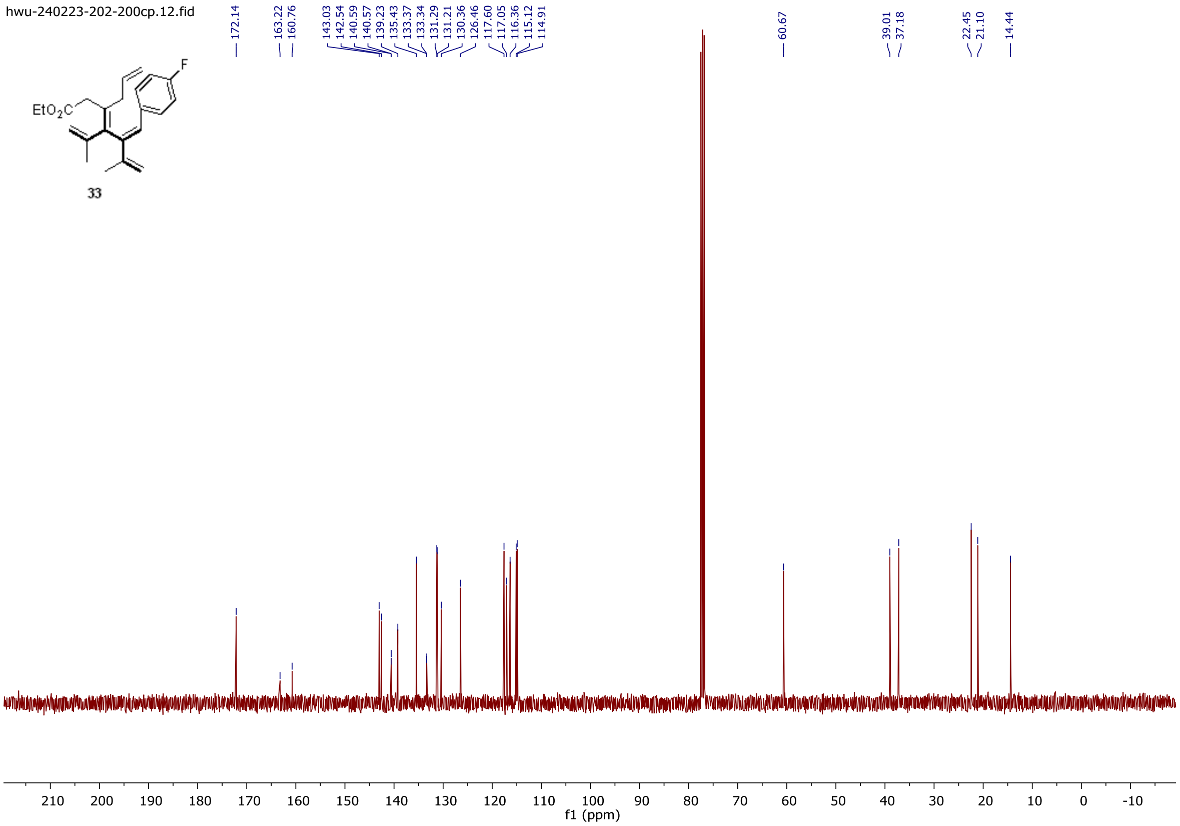
hwu-240223-202-200cp.12.fid
172.14
163.22
160.76
143.03
142.54
140.59
140.57
139.23
135.43
133.37
133.34
131.29
131.21
130.36
126.46
117.60
117.05
116.36
115.12
114.91
60.67
39.01
37.18
22.45
21.10
14.44
EtO2C
F
33
210 200 190 180 170 160 150 140 130 120 110 100 90 80 70 60 50 40 30 20 10 0 -10
f1 (ppm)

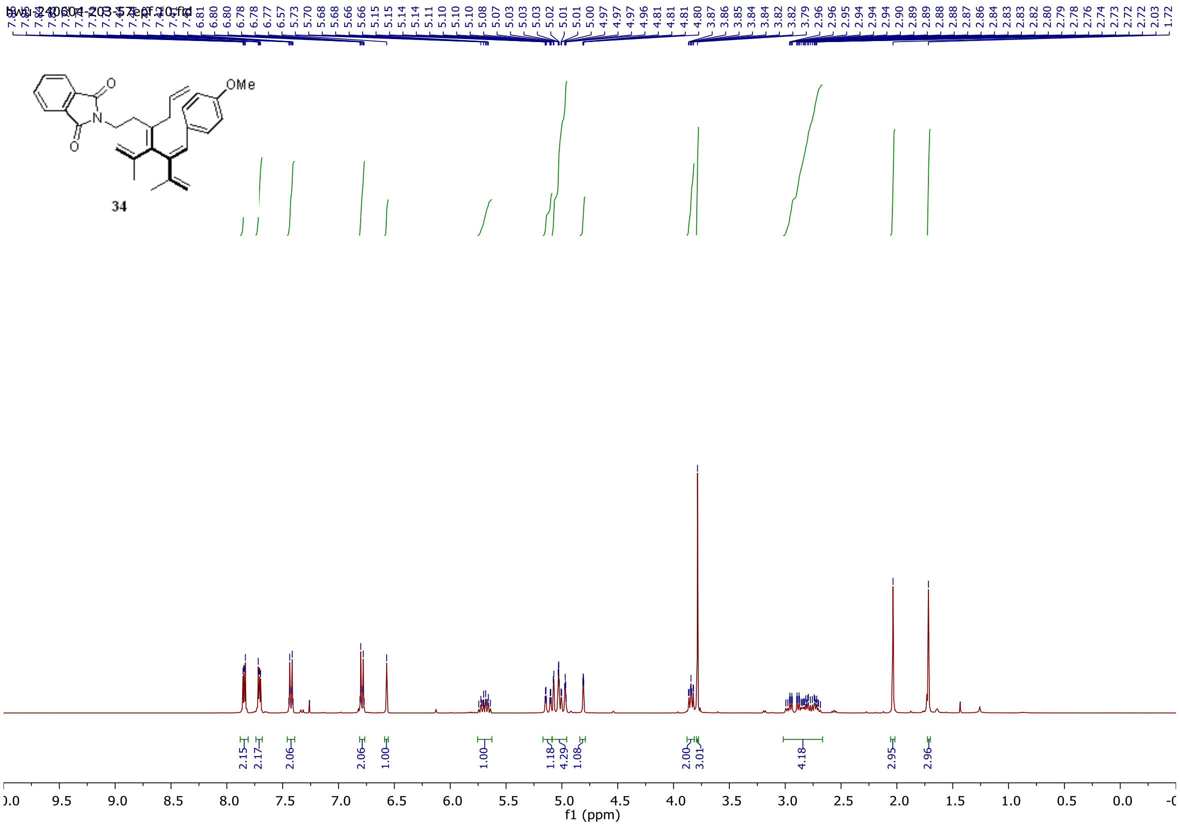
hwu-240604-203-57epf.10.fid
OMe
N
O
O
34
2.15
2.17
2.06
2.06
1.00
1.00
1.18
4.29
1.08
2.00
3.01
4.18
2.95
2.96
f1 (ppm)

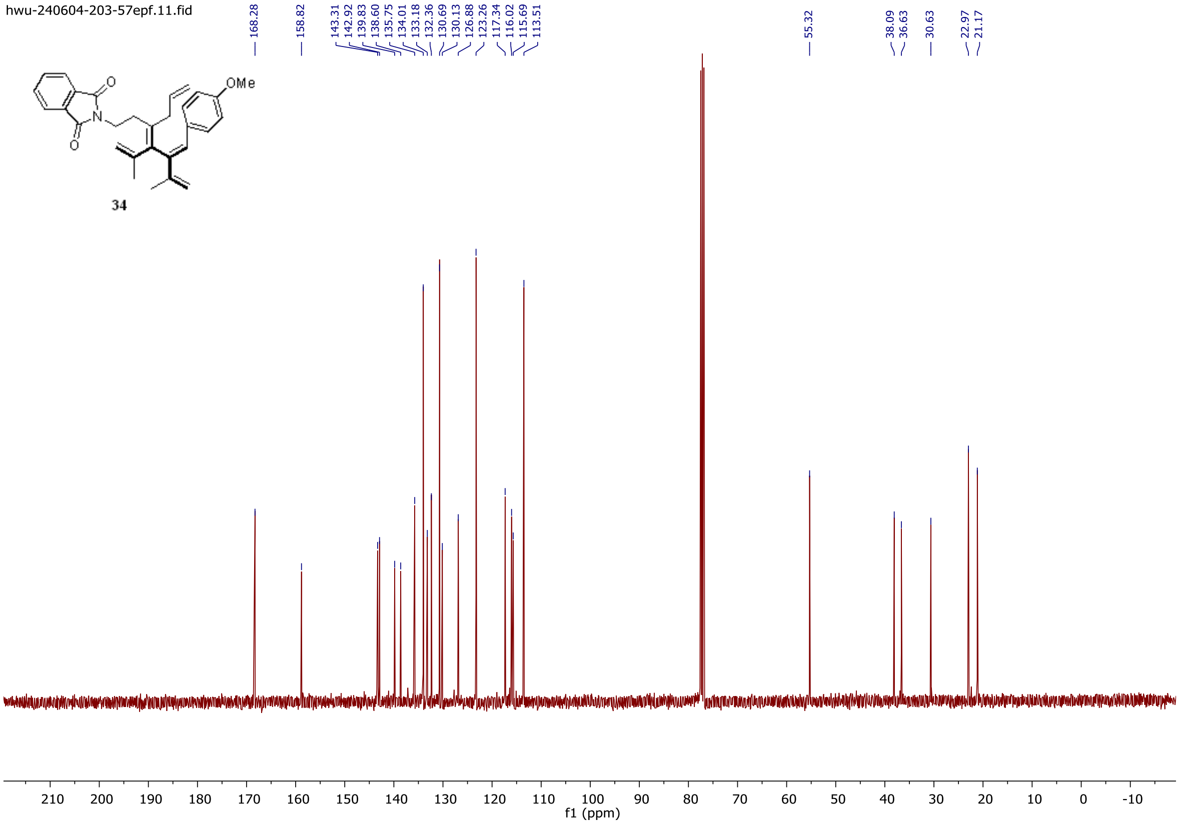
hwu-240604-203-57epf.11.fid
168.28
158.82
143.31
142.92
139.83
138.60
135.75
134.01
133.18
132.36
130.69
130.13
126.88
123.26
117.34
116.02
115.69
113.51
55.32
38.09
36.63
30.63
22.97
21.17
OMe
N
O
O
34
f1 (ppm)

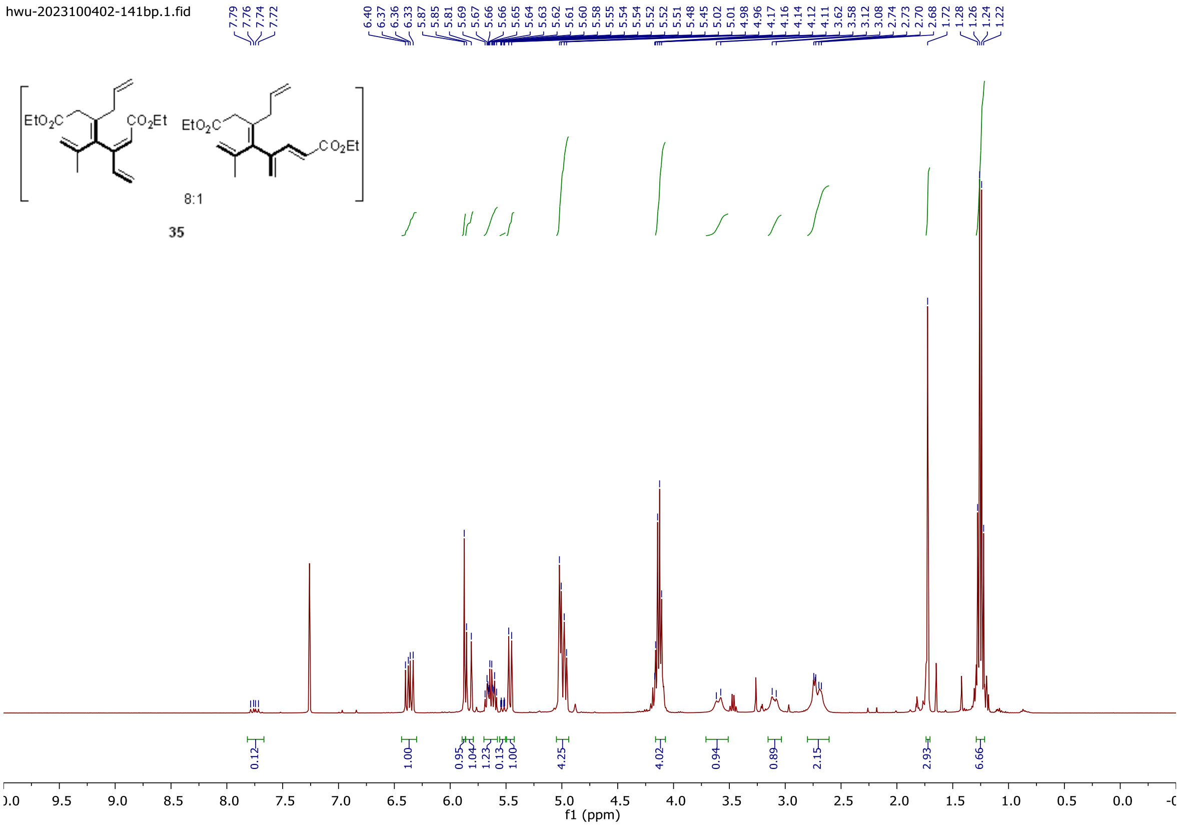
hwu-2023100402-141bp.1.fid
7.79 7.76 7.74 7.72
6.40 6.37 6.36 6.33 5.87 5.85 5.81 5.69 5.67 5.66 5.66 5.65 5.64 5.63 5.62 5.61 5.60 5.58 5.55 5.54 5.54 5.52 5.52 5.51 5.48 5.45 5.02 5.01 4.98 4.96 4.17 4.16 4.14 4.12 4.11 3.62 3.58 3.12 3.08 2.74 2.73 2.70 2.68 1.72 1.28 1.26 1.24 1.22
EtO_2C
CO_2Et
8:1
35
0.12 1.00 0.95 1.04 1.23 0.13 1.00 4.25 4.02 0.94 0.89 2.15 2.93 6.66
f1 (ppm)

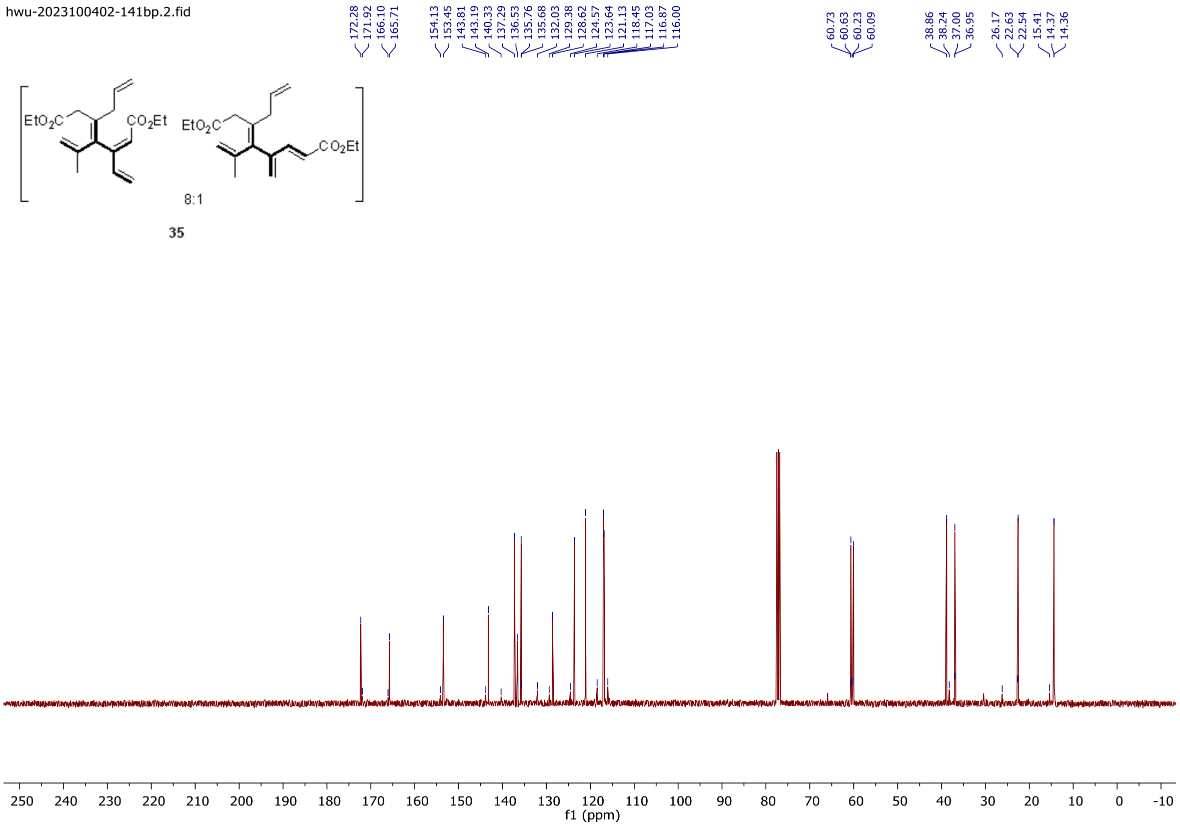
hwu-2023100402-141bp.2.fid
172.28 171.92 166.10 165.71 154.13 153.45 143.81 143.19 140.33 137.29 136.53 135.76 135.68 132.03 129.38 128.62 124.57 123.64 121.13 118.45 117.03 116.87 116.00
60.73 60.63 60.23 60.09
38.86 38.24 37.00 36.95 26.17 22.63 22.54 15.41 14.37 14.36
EtO_2C
CO_2Et
8:1
35
f1 (ppm)

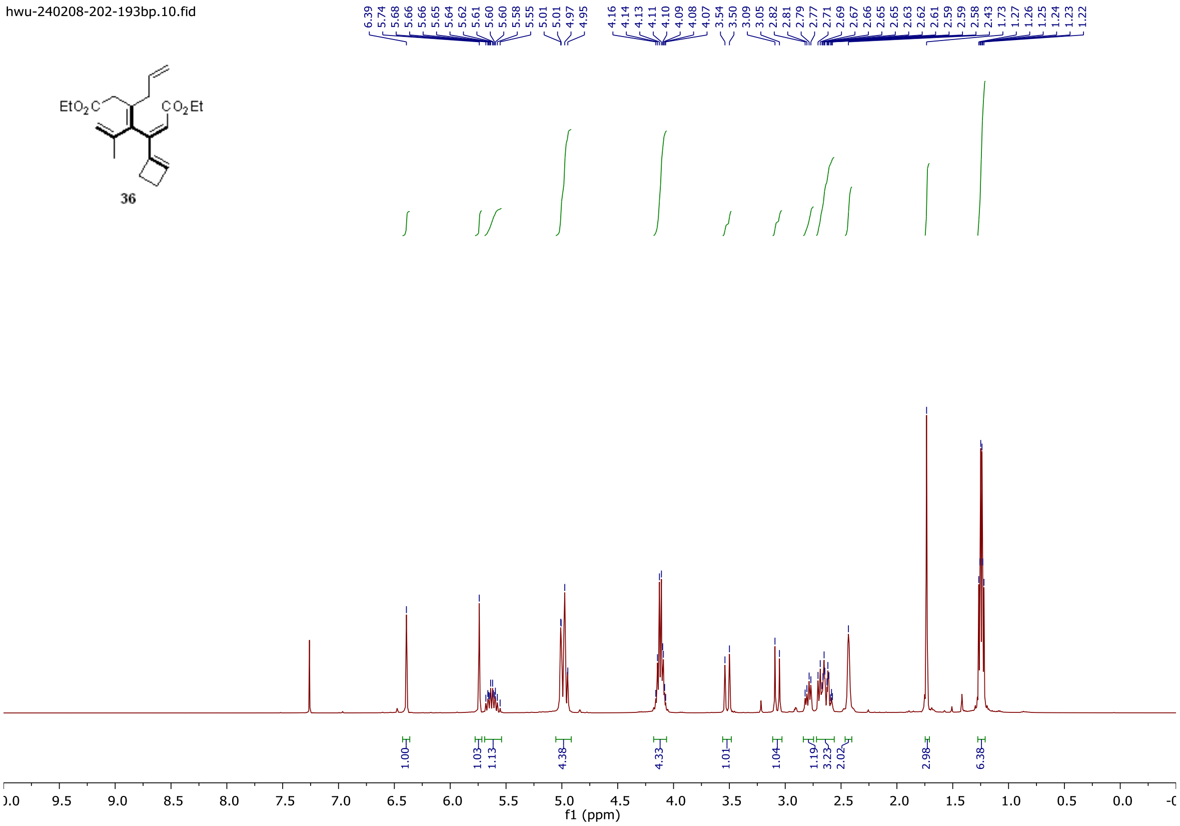
hwu-240208-202-193bp.10.fid
6.39 5.74 5.68 5.66 5.66 5.65 5.64 5.62 5.61 5.60 5.60 5.58 5.55 5.01 5.01 4.97 4.95 4.16 4.14 4.13 4.11 4.10 4.09 4.08 4.07 3.54 3.50 3.09 3.05 2.82 2.81 2.79 2.77 2.71 2.69 2.67 2.66 2.65 2.65 2.63 2.62 2.61 2.59 2.59 2.58 2.43 1.73 1.27 1.26 1.25 1.24 1.23 1.22
EtO2C
CO2Et
36
1.00 1.03 1.13 4.38 4.33 1.01 1.04 1.19 3.23 2.02 2.98 6.38
f1 (ppm)

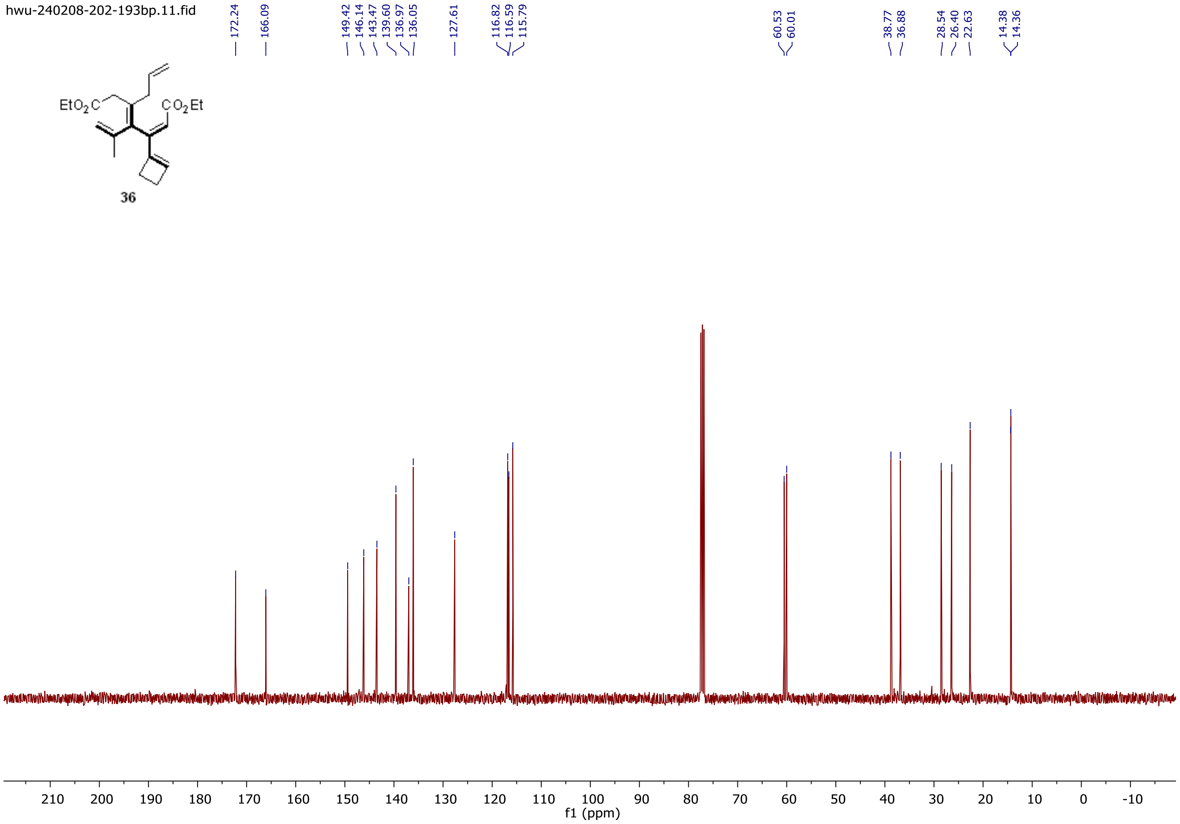
hwu-240208-202-193bp.11.fid
172.24 166.09 149.42 146.14 143.47 139.60 136.97 136.05 127.61 116.82 116.59 115.79 60.53 60.01 38.77 36.88 28.54 26.40 22.63 14.38 14.36
EtO2C
CO2Et
36
f1 (ppm)

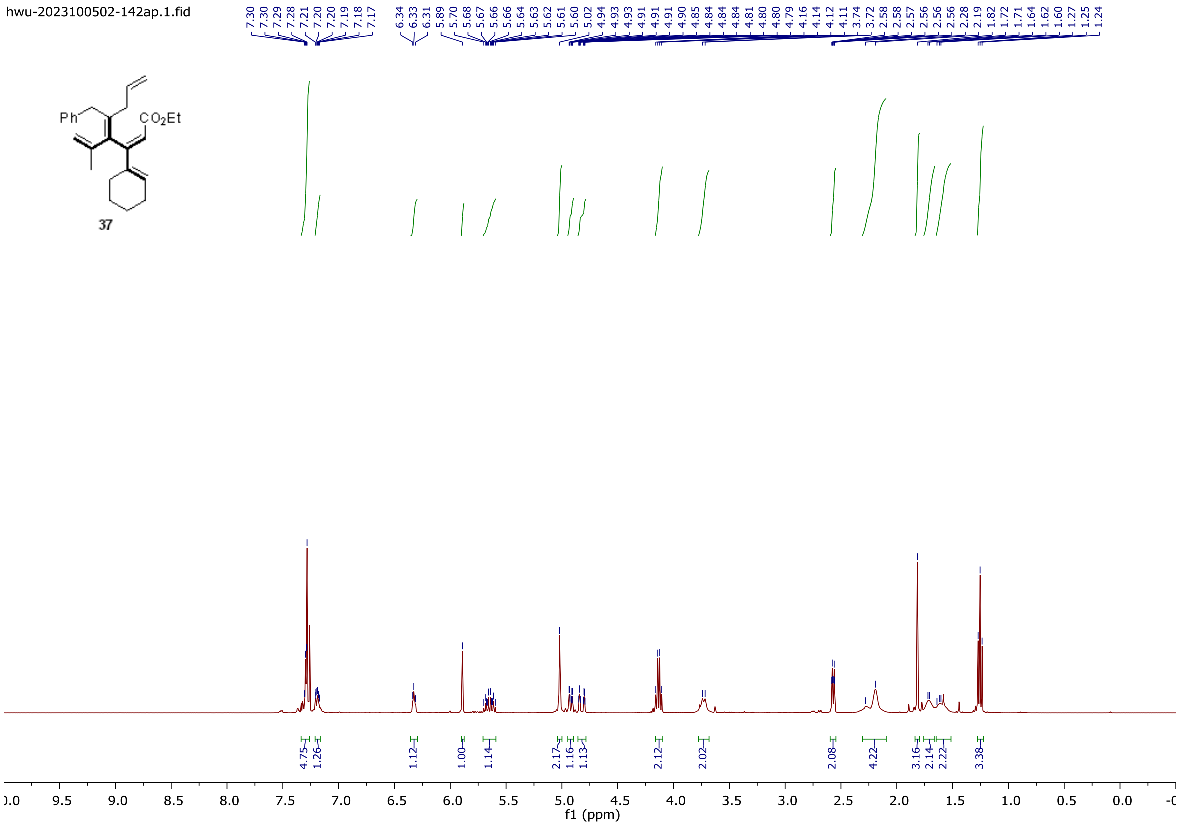
hwu-2023100502-142ap.1.fid
7.30 7.30 7.29 7.28 7.21 7.20 7.20 7.19 7.18 7.17
6.34 6.33 6.31 5.89 5.70 5.68 5.67 5.66 5.66 5.64 5.63 5.62 5.61 5.60 5.02 4.94 4.93 4.93 4.91 4.91 4.91 4.90 4.85 4.84 4.84 4.84 4.81 4.80 4.80 4.79 4.16 4.14 4.12 4.11 3.74 3.72 2.58 2.58 2.57 2.56 2.56 2.56 2.28 2.19 1.82 1.72 1.71 1.64 1.62 1.60 1.27 1.25 1.24
Ph
CO2Et
37
4.75 1.26 1.12 1.00 1.14 2.17 1.16 1.13 2.12 2.02 2.08 4.22 3.16 2.14 2.22 3.38
f1 (ppm)

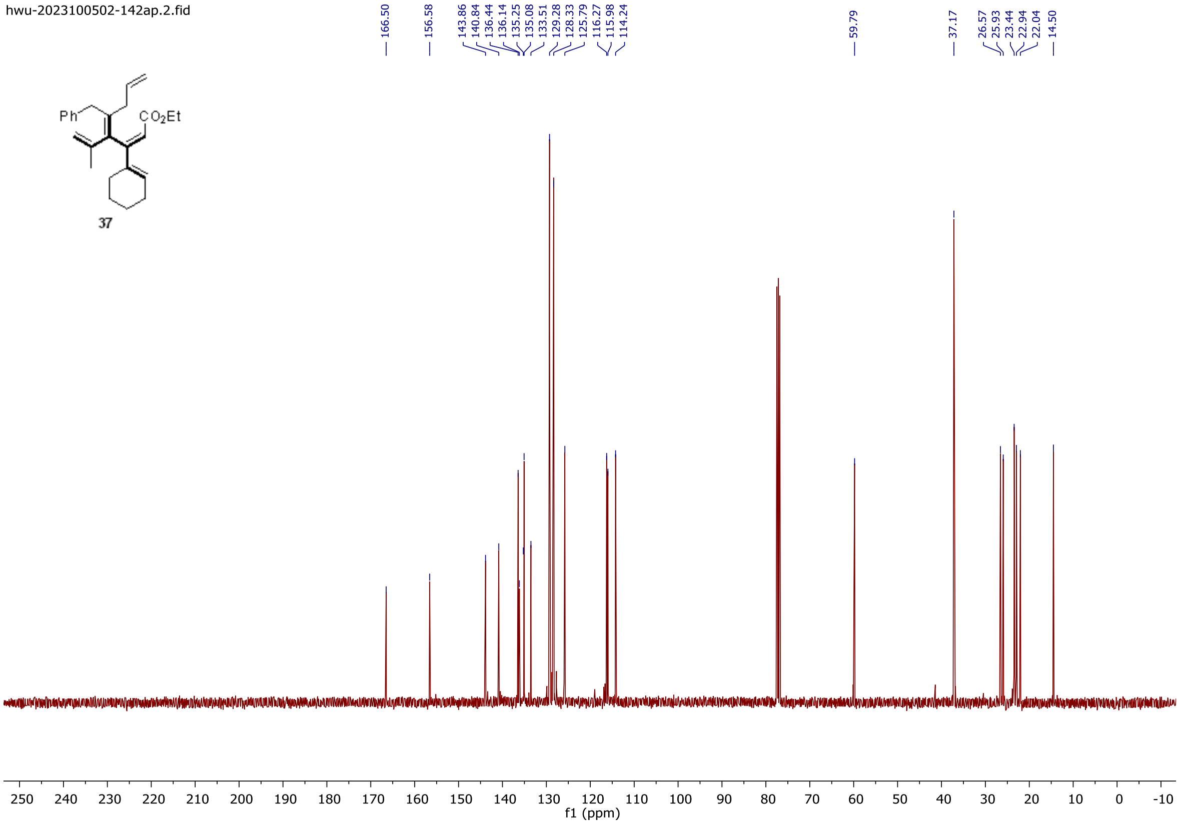
hwu-2023100502-142ap.2.fid
166.50
156.58
143.86 140.84 136.44 136.14 135.25 135.08 133.51 129.28 128.33 125.79 116.27 115.98 114.24
59.79
37.17
26.57 25.93 23.44 22.94 22.04 14.50
Ph
CO2Et
37
f1 (ppm)

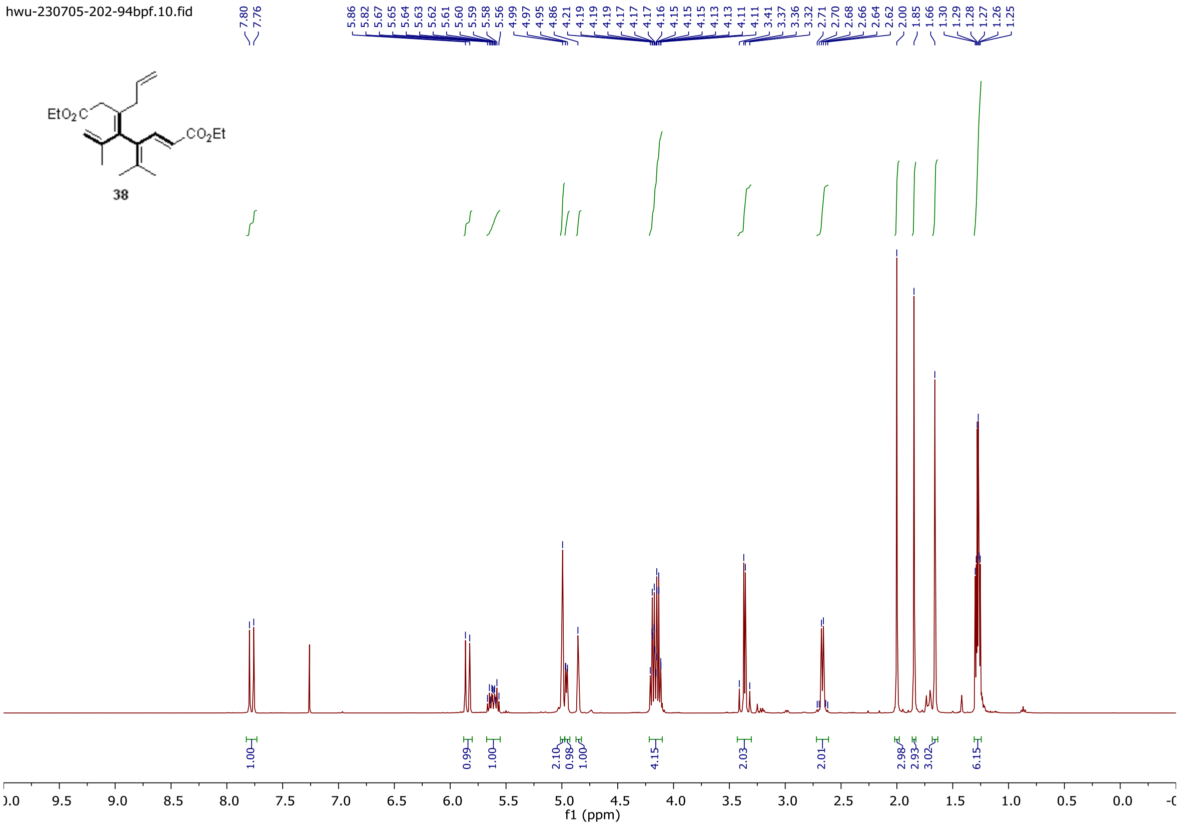
hwu-230705-202-94bpf.10.fid
7.80 7.76 5.86 5.82 5.67 5.65 5.64 5.63 5.62 5.61 5.60 5.59 5.58 5.56 4.99 4.97 4.95 4.86 4.21 4.19 4.19 4.19 4.17 4.17 4.17 4.16 4.15 4.15 4.15 4.13 4.13 4.11 4.11 3.41 3.37 3.36 3.32 2.71 2.70 2.68 2.66 2.64 2.62 2.00 1.85 1.66 1.30 1.29 1.28 1.27 1.26 1.25
EtO2C
CO2Et
1.00 0.99 1.00 2.10 0.98 1.00 4.15 2.03 2.01 2.98 2.93 3.02 6.15
f1 (ppm)

38

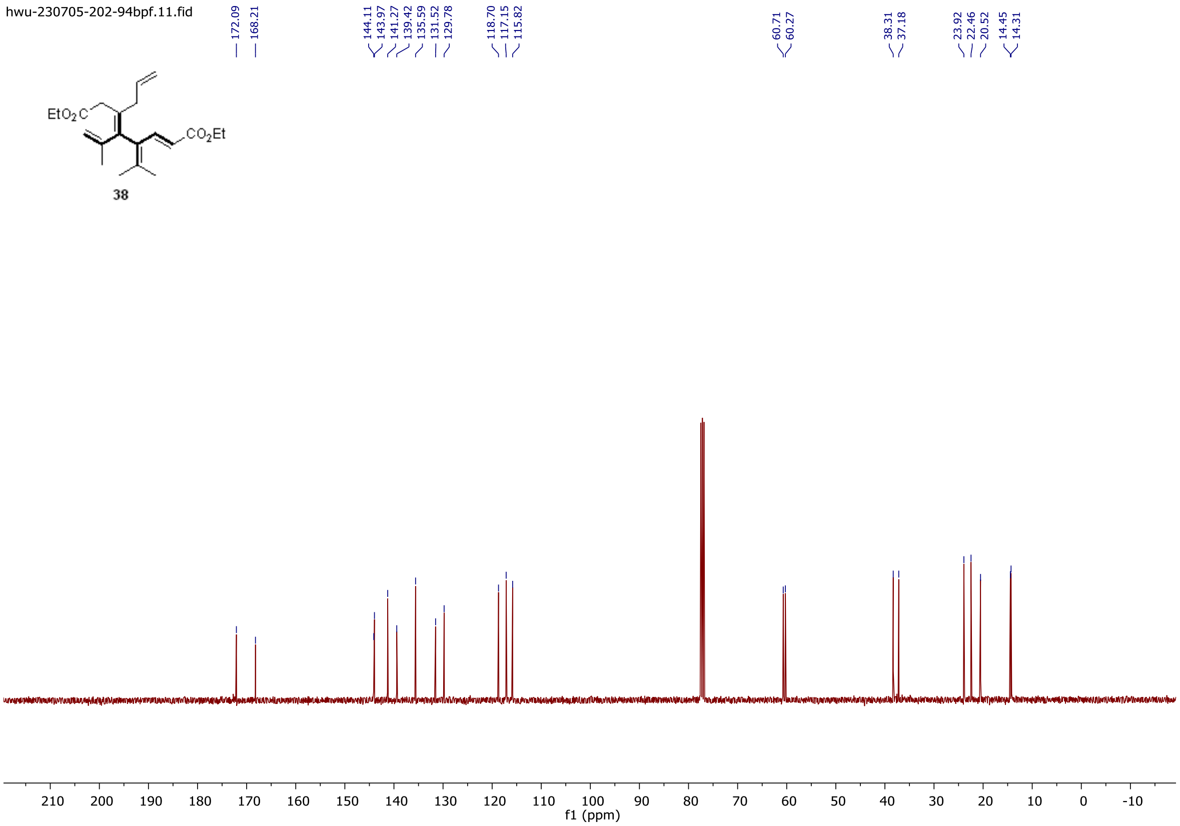
hwu-230705-202-94bpf.11.fid
172.09 168.21 144.11 143.97 141.27 139.42 135.59 131.52 129.78 118.70 117.15 115.82 60.71 60.27 38.31 37.18 23.92 22.46 20.52 14.45 14.31
EtO2C
CO2Et
f1 (ppm)

38

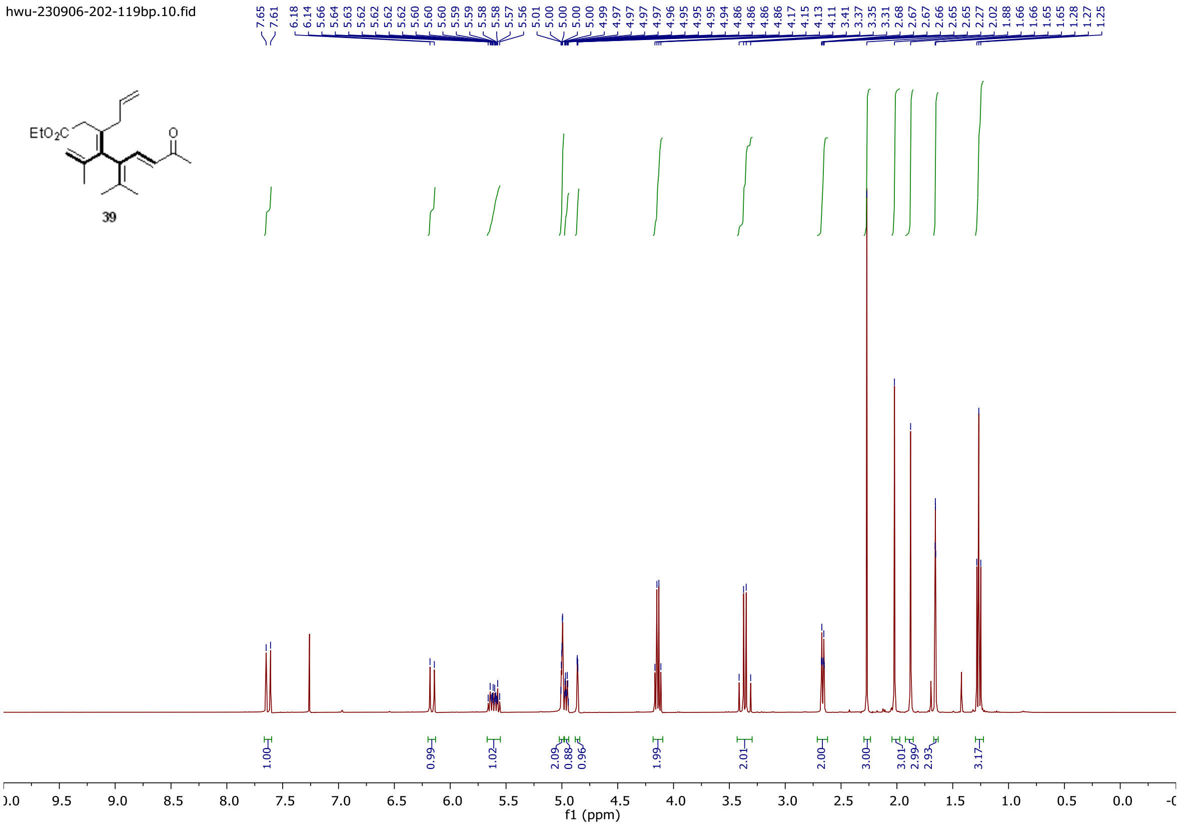

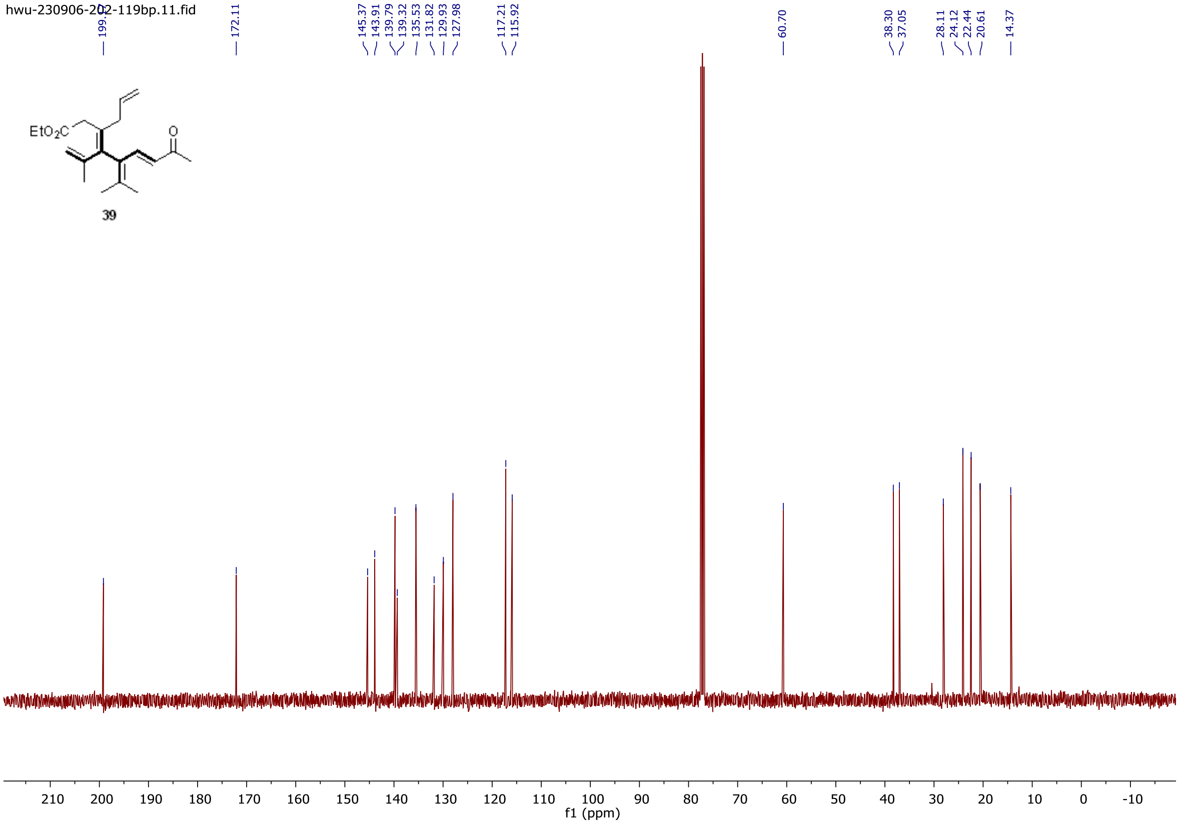

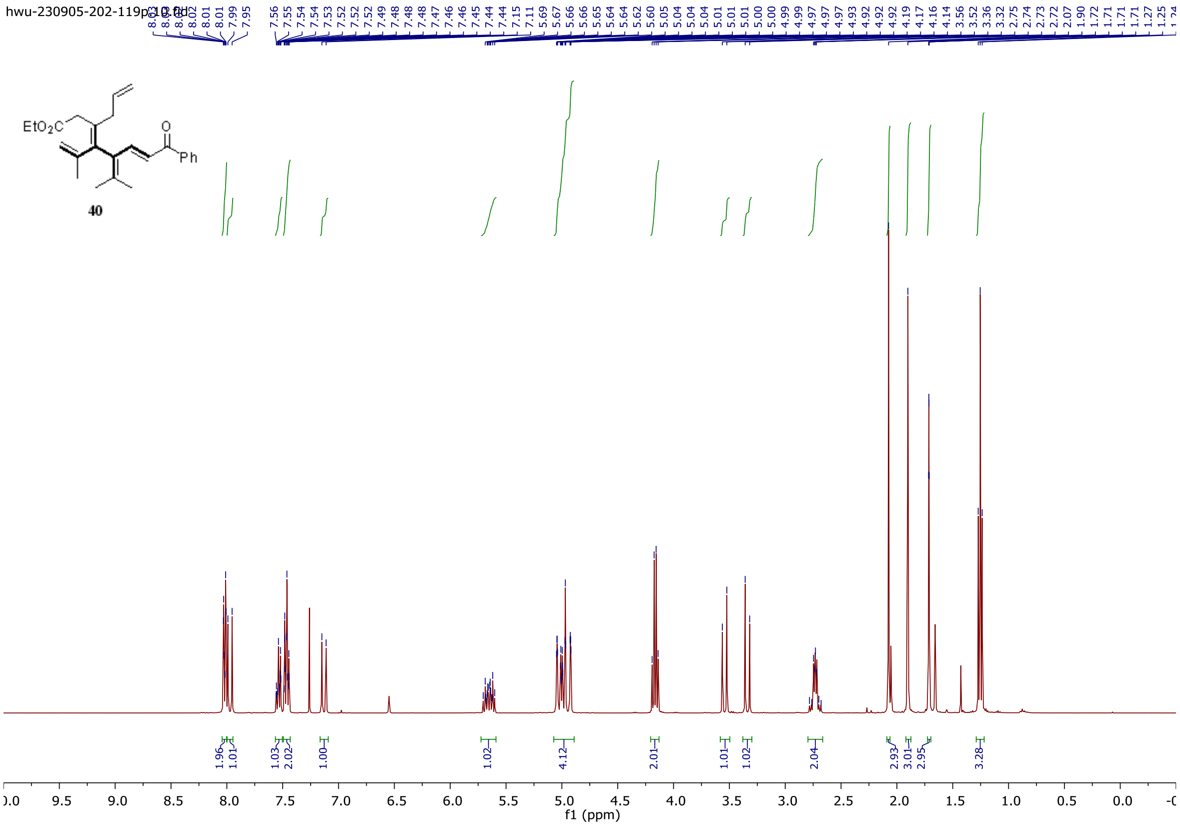
hwu-230905-202-119p.10.fid
EtO2C
O
Ph
40
1.96
1.01
1.03
2.02
1.00
1.02
4.12
2.01
1.01
1.02
2.04
2.93
3.01
2.95
3.28
f1 (ppm)

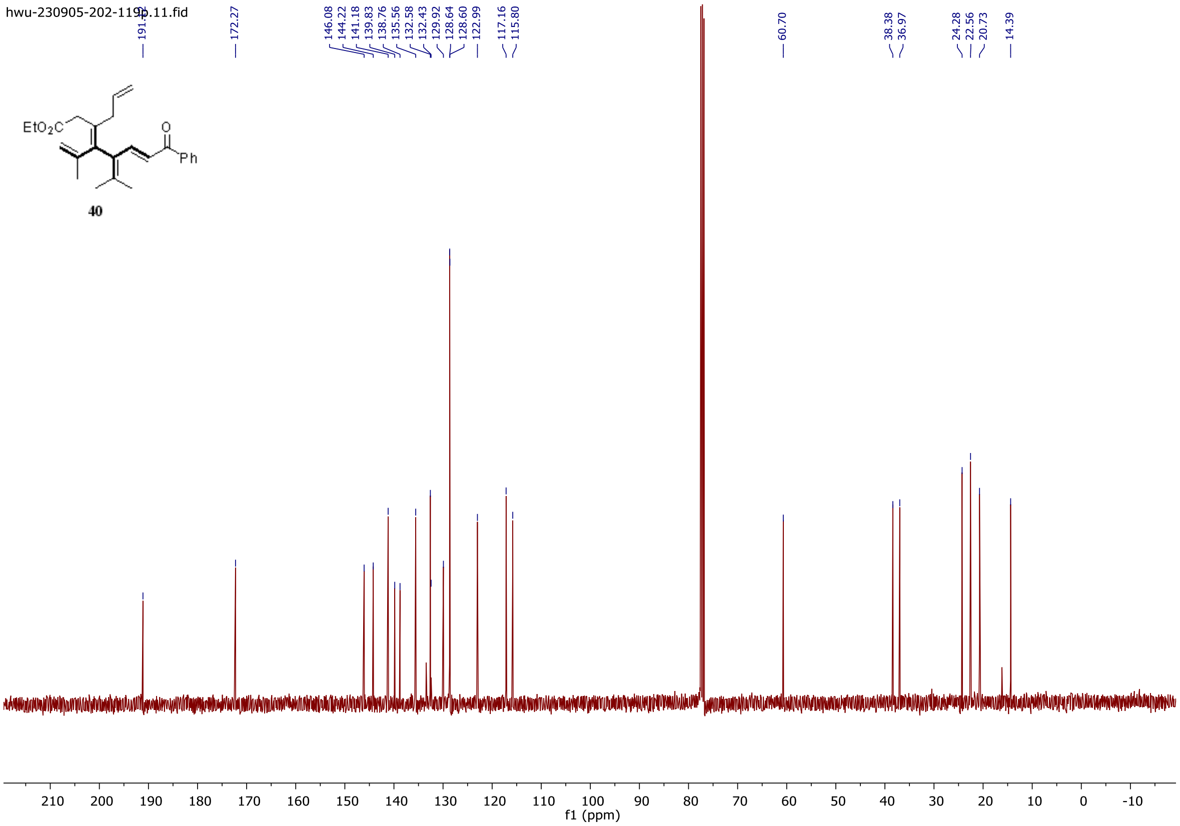
hwu-230905-202-119p.11.fid
191.12
172.27
146.08
144.22
141.18
139.83
138.76
135.56
132.58
132.43
129.92
128.64
128.60
122.99
117.16
115.80
60.70
38.38
36.97
24.28
22.56
20.73
14.39
EtO2C
O
Ph
40
f1 (ppm)

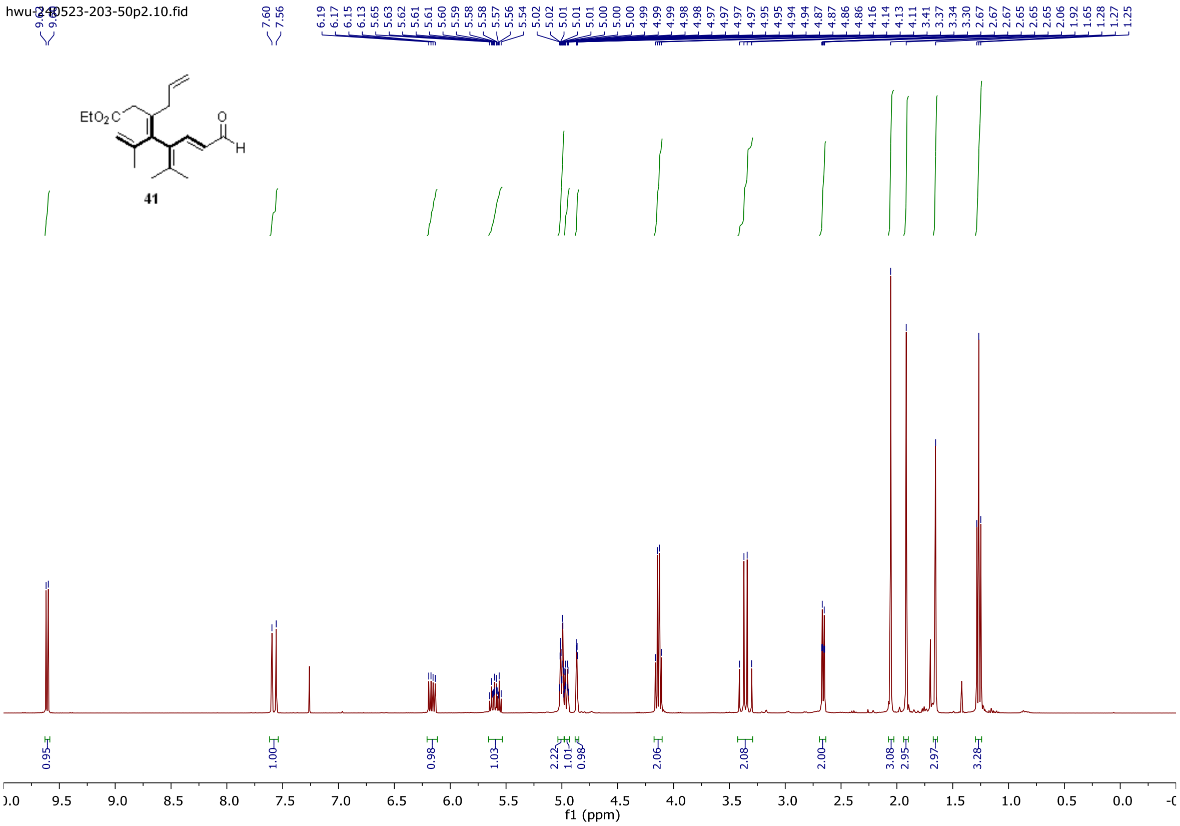
hwu-240523-203-50p2.10.fid
7.60
7.56
6.19
6.17
6.15
6.13
5.65
5.63
5.62
5.61
5.61
5.60
5.59
5.58
5.58
5.57
5.56
5.56
5.54
5.02
5.01
5.01
5.01
5.00
5.00
5.00
4.99
4.99
4.99
4.98
4.98
4.97
4.97
4.97
4.95
4.95
4.94
4.94
4.87
4.87
4.86
4.86
4.16
4.14
4.13
4.11
3.41
3.37
3.34
3.30
2.67
2.67
2.67
2.65
2.65
2.65
2.06
1.92
1.65
1.28
1.27
1.25
EtO2C
O
H
41
0.95
1.00
0.98
1.03
2.22
1.01
0.98
2.06
2.08
2.00
3.08
2.95
2.97
3.28
f1 (ppm)

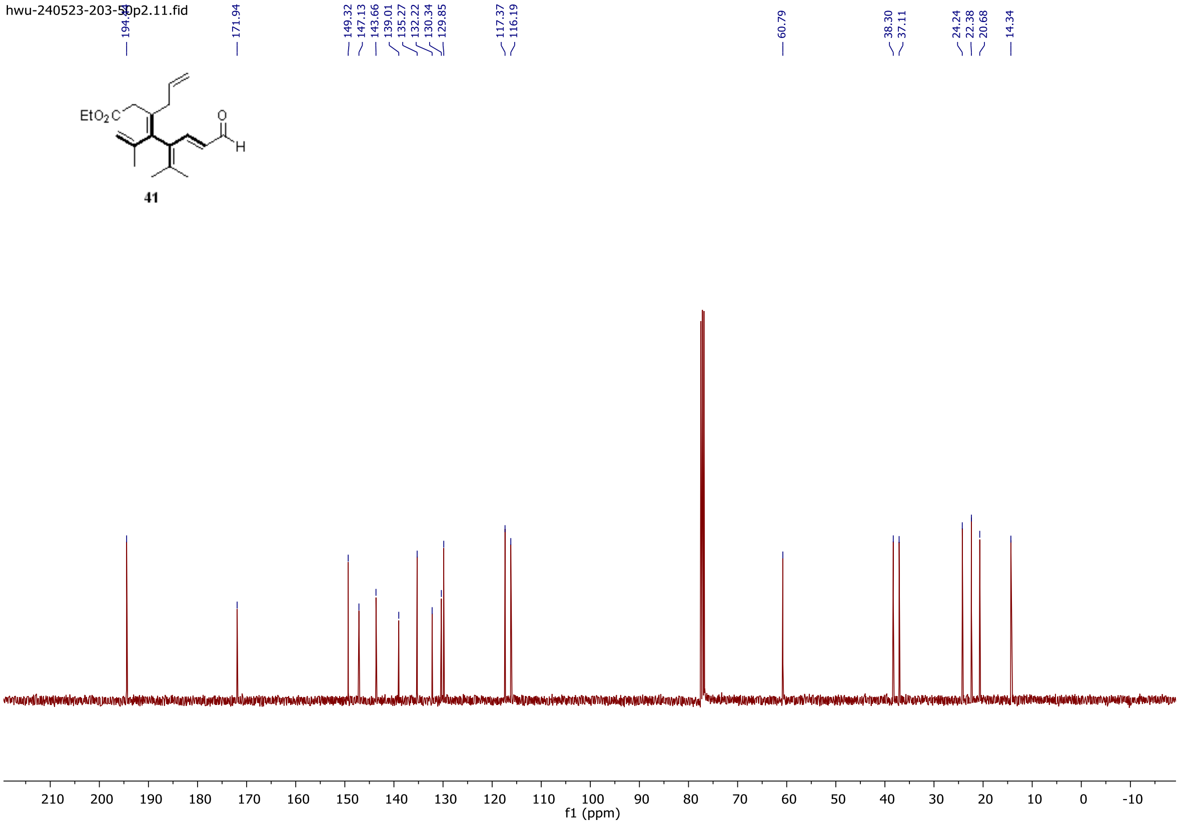
hwu-240523-203-50p2.11.fid
171.94
149.32
147.13
143.66
139.01
135.27
132.22
130.34
129.85
117.37
116.19
60.79
38.30
37.11
24.24
22.38
20.68
14.34
EtO2C
O
H
41
f1 (ppm)

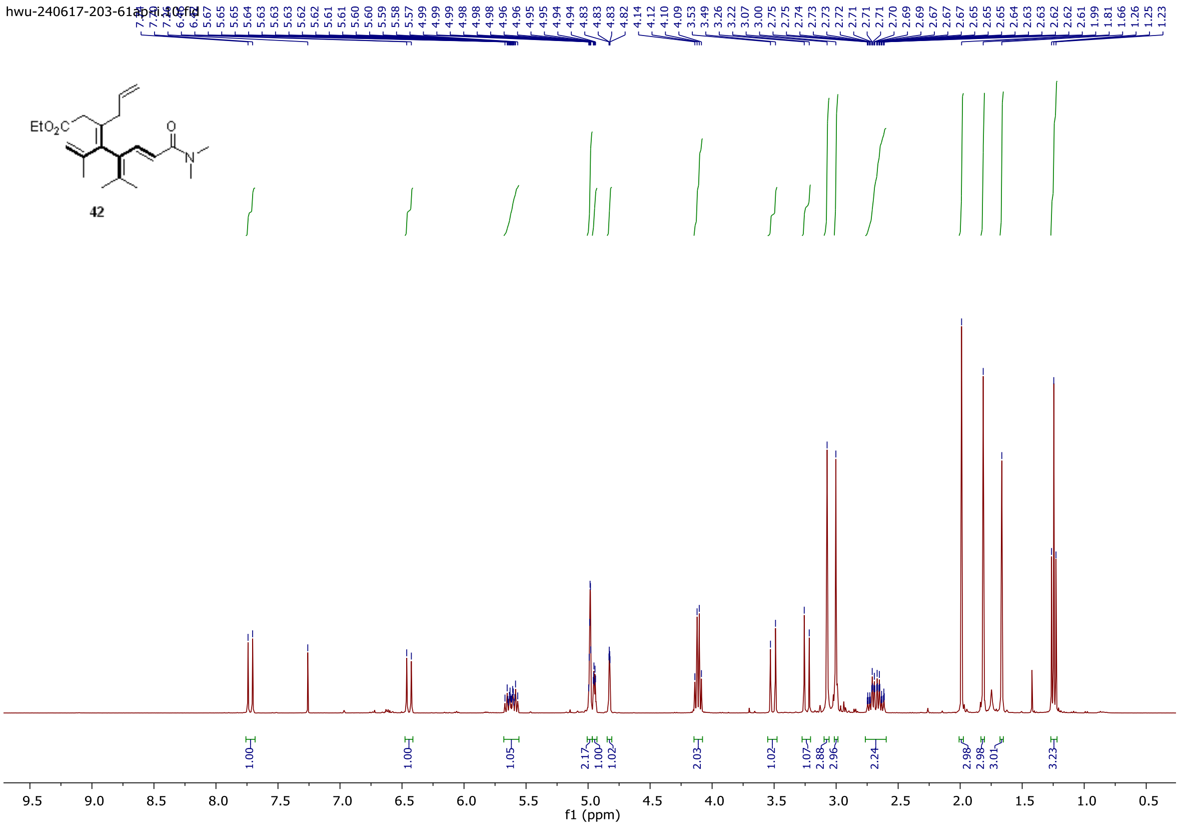
hwu-240617-203-61ap

EtO2C
O
N
42
1.00
1.00
1.05
2.17
1.00
1.02
2.03
1.02
1.07
2.88
2.96
2.24
2.98
2.98
3.01
3.23
f1 (ppm)

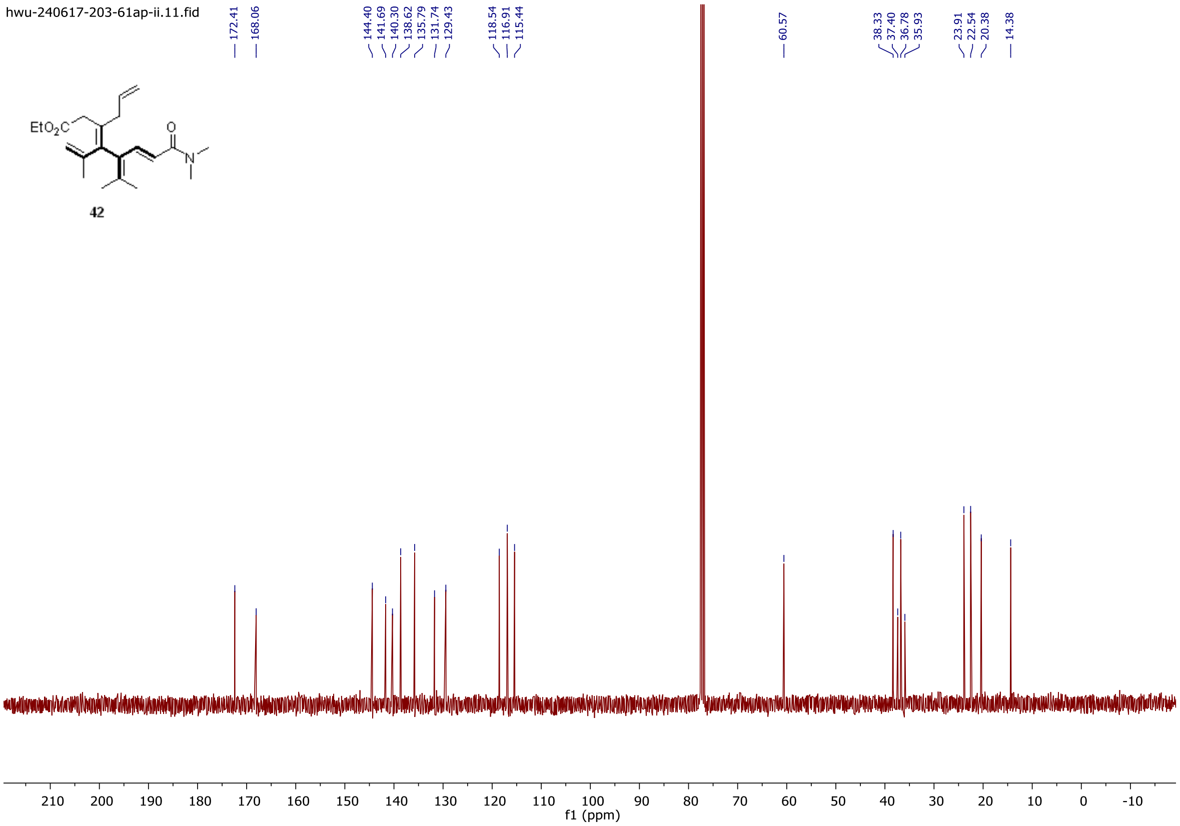
hwu-240617-203-61ap-ii.11.fid
172.41
168.06
144.40
141.69
140.30
138.62
135.79
131.74
129.43
118.54
116.91
115.44
60.57
38.33
37.40
36.78
35.93
23.91
22.54
20.38
14.38
EtO2C
O
N
42
f1 (ppm)

pq-1-93.1.fid

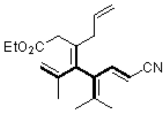

43

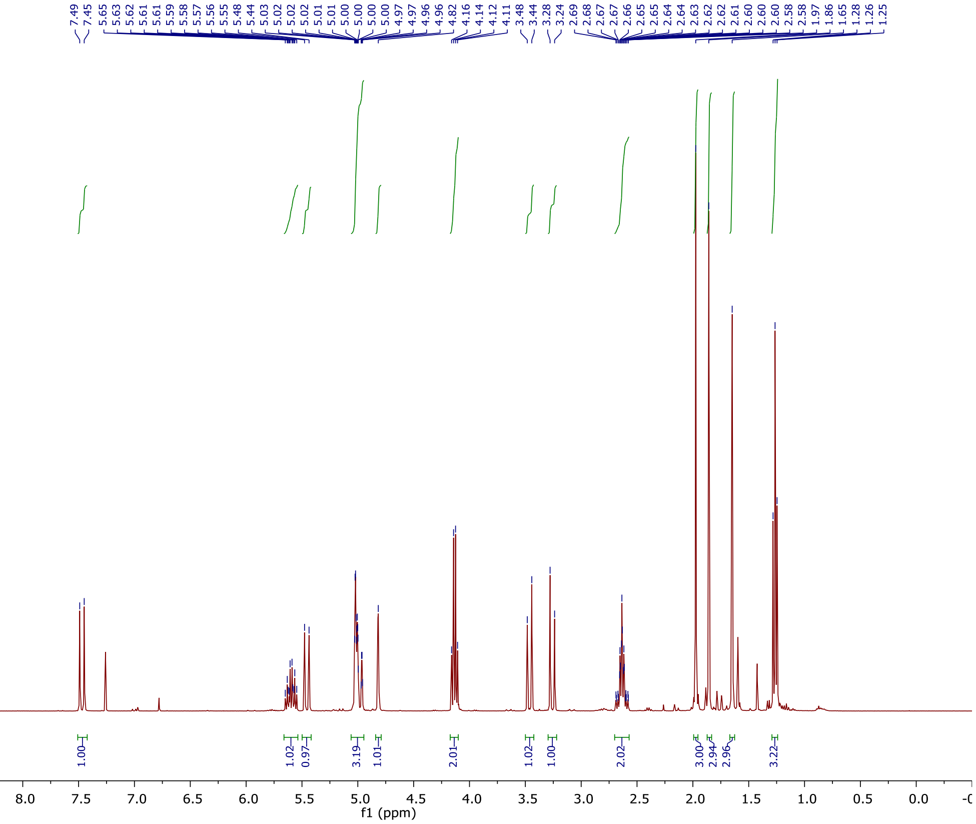

pq-1-93.2.fid

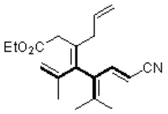

43

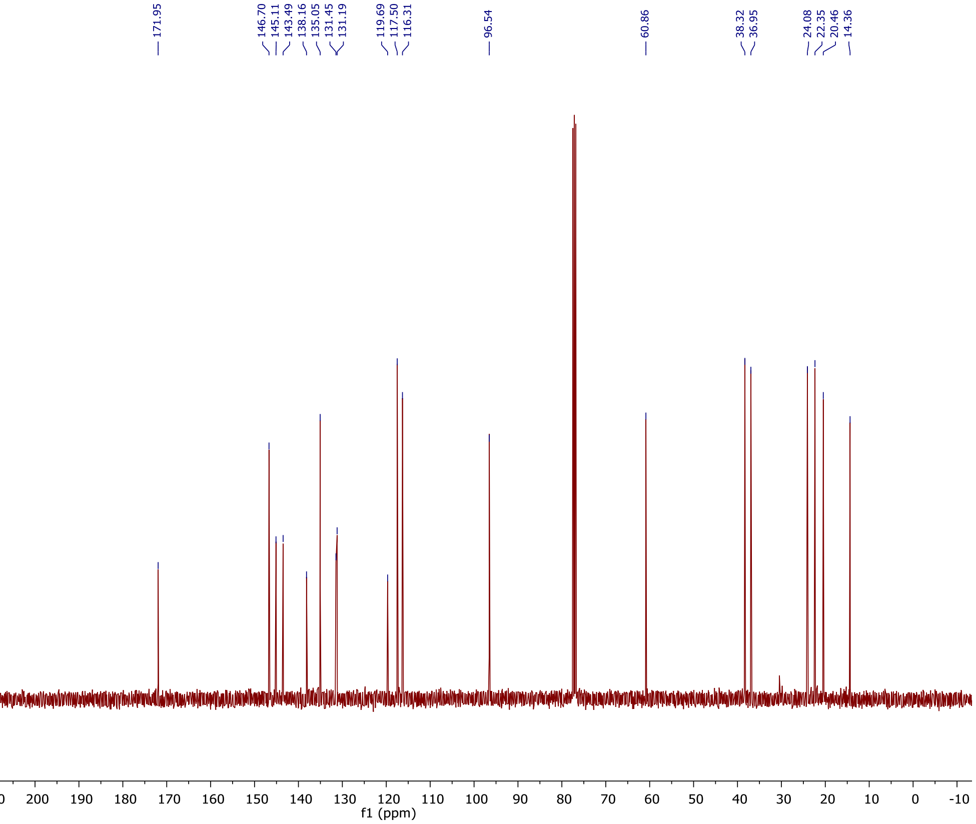

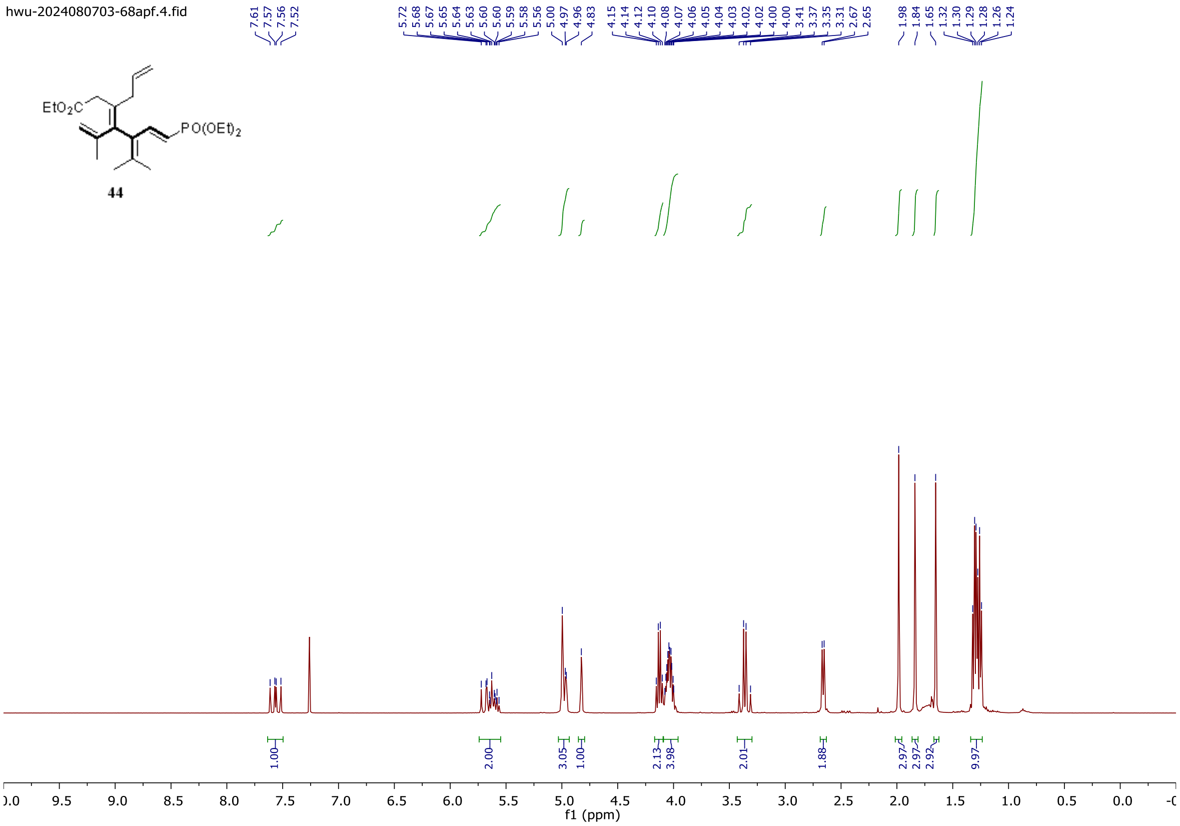
hwu-2024080703-68apf.4.fid
7.61 7.57 7.56 7.52
5.72 5.68 5.67 5.65 5.64 5.63 5.60 5.59 5.58 5.56 5.00 4.97 4.96 4.83
4.15 4.14 4.12 4.10 4.08 4.07 4.06 4.05 4.04 4.03 4.02 4.02 4.00 4.00 3.41 3.37 3.35 3.31 2.67 2.65
1.98 1.84 1.65 1.32 1.30 1.29 1.28 1.26 1.24
EtO_2C
$PO(OEt)_2$
44
1.00 2.00 3.05 1.00 2.13 3.98 2.01 1.88 2.97 2.97 2.92 9.97
9.5 9.0 8.5 8.0 7.5 7.0 6.5 6.0 5.5 5.0 4.5 4.0 3.5 3.0 2.5 2.0 1.5 1.0 0.5 0.0
f1 (ppm)

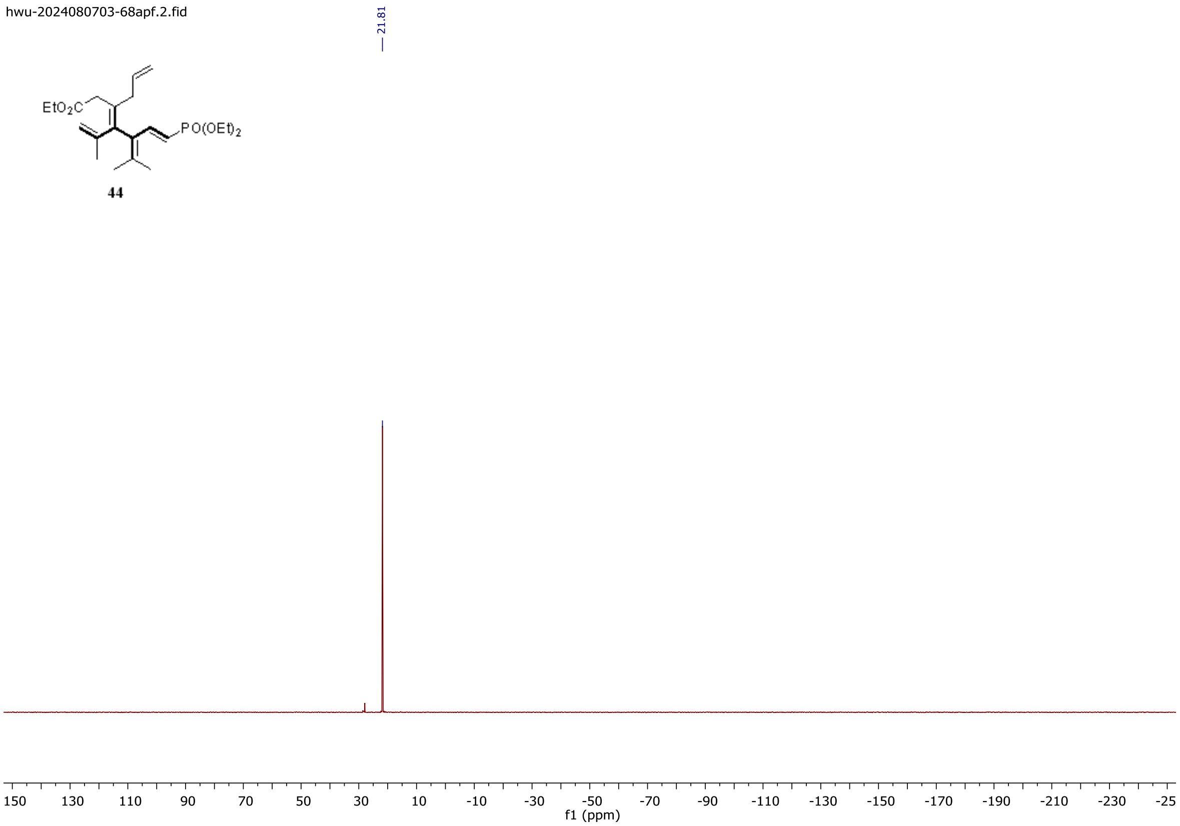
hwu-2024080703-68apf.2.fid
21.81
EtO_2C
$PO(OEt)_2$
44
150 130 110 90 70 50 30 10 -10 -30 -50 -70 -90 -110 -130 -150 -170 -190 -210 -230
f1 (ppm)

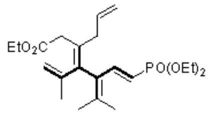
EtO2C
PO(OEt)2

44

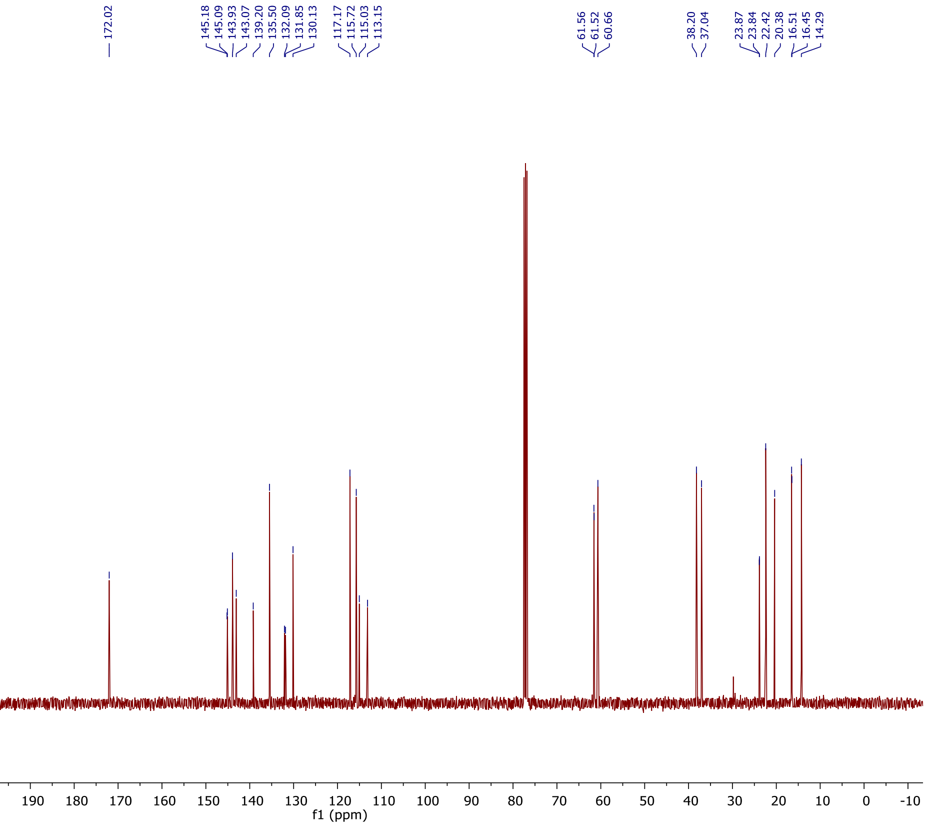
hwu-2024080703-68apf.3.fid
172.02
145.18
145.09
143.93
143.07
139.20
135.50
132.09
131.85
130.13
117.17
115.72
115.03
113.15
61.56
61.52
60.66
38.20
37.04
23.87
23.84
22.42
20.38
16.51
16.45
14.29
250 240 230 220 210 200 190 180 170 160 150 140 130 120 110 100 90 80 70 60 50 40 30 20 10 0 -10
f1 (ppm)

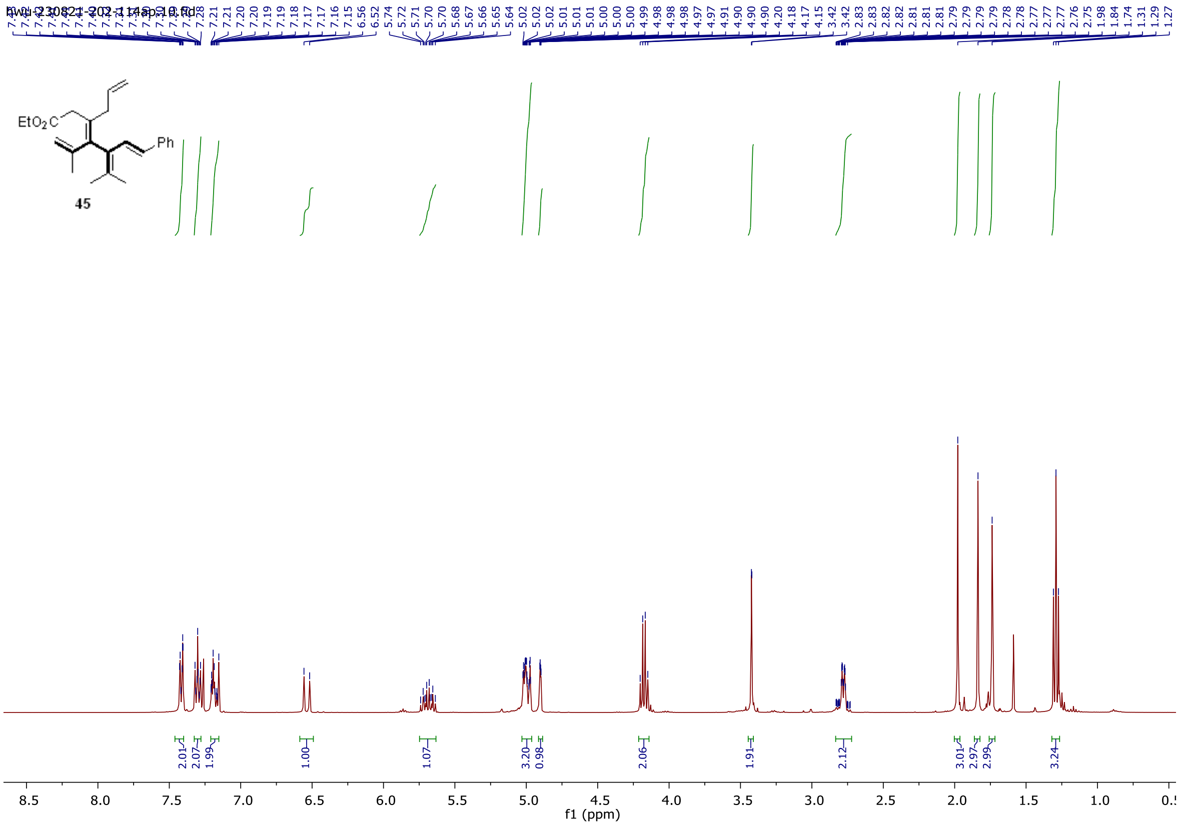
hwu-230821-202-114ap.10.fid
EtO2C
Ph
45
2.01
2.07
1.99
1.00
1.07
3.20
0.98
2.06
1.91
2.12
3.01
2.97
2.99
3.24
f1 (ppm)

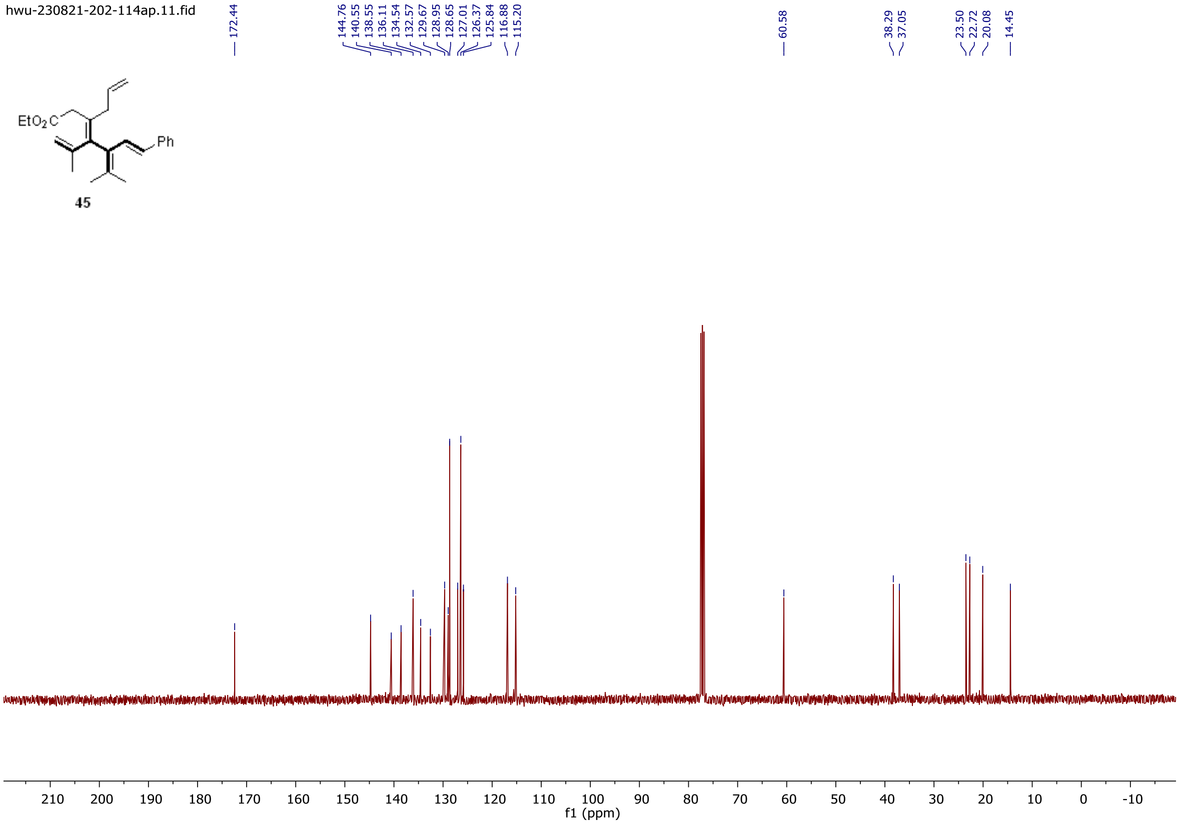
hwu-230821-202-114ap.11.fid
172.44
144.76
140.55
138.55
136.11
134.54
132.57
129.67
128.95
128.65
127.01
126.37
125.84
116.88
115.20
60.58
38.29
37.05
23.50
22.72
20.08
14.45
EtO2C
Ph
45
f1 (ppm)

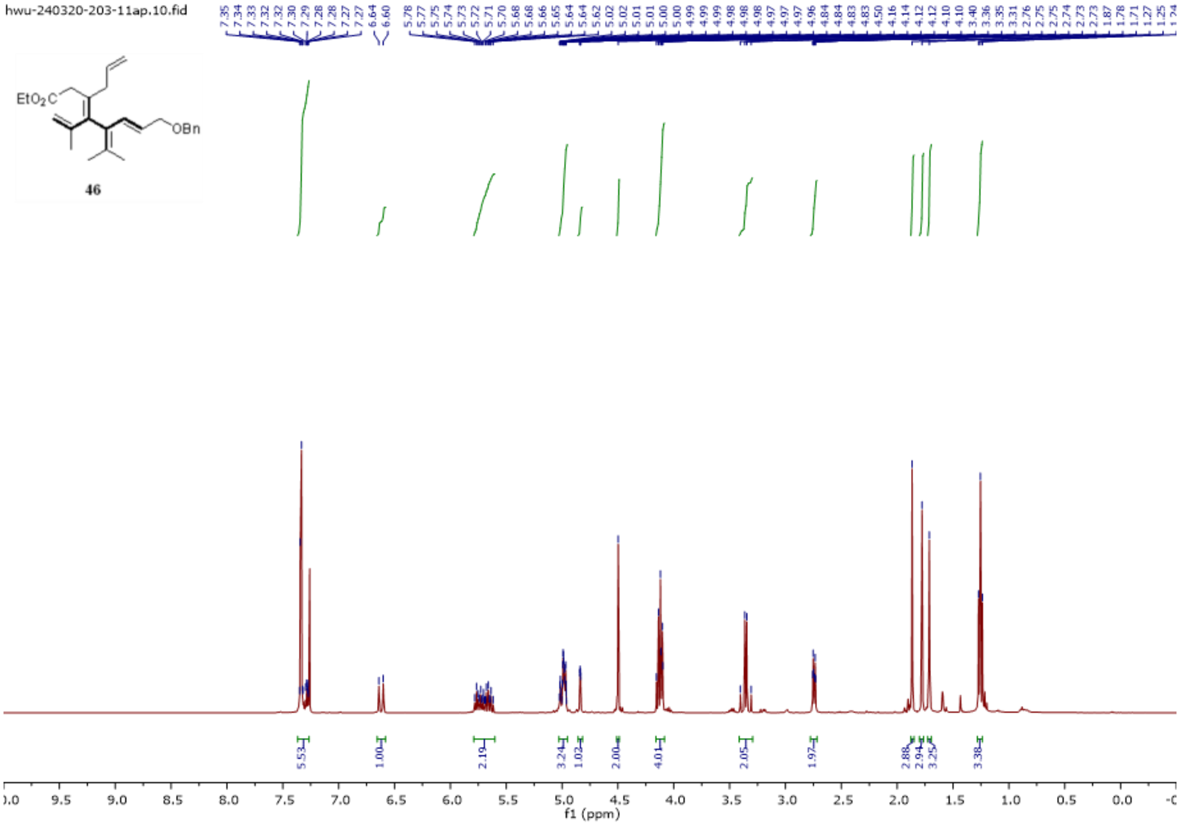
hwu-240320-203-11ap.10.fid
EtO2C
OBn
46
7.35
7.34
7.33
7.32
7.30
7.29
7.28
7.28
7.27
7.27
6.64
6.60
5.78
5.77
5.75
5.74
5.73
5.72
5.71
5.70
5.68
5.68
5.66
5.65
5.64
5.64
5.62
5.02
5.02
5.01
5.01
5.00
5.00
4.99
4.99
4.99
4.98
4.98
4.98
4.97
4.97
4.97
4.96
4.84
4.84
4.83
4.83
4.50
4.16
4.14
4.12
4.12
4.10
4.10
3.40
3.36
3.35
3.31
2.76
2.75
2.75
2.74
2.73
2.73
1.87
1.78
1.71
1.27
1.25
1.24
5.53
1.00
2.19
3.24
1.02
2.00
4.01
2.05
1.97
2.88
2.94
3.25
3.38
f1 (ppm)

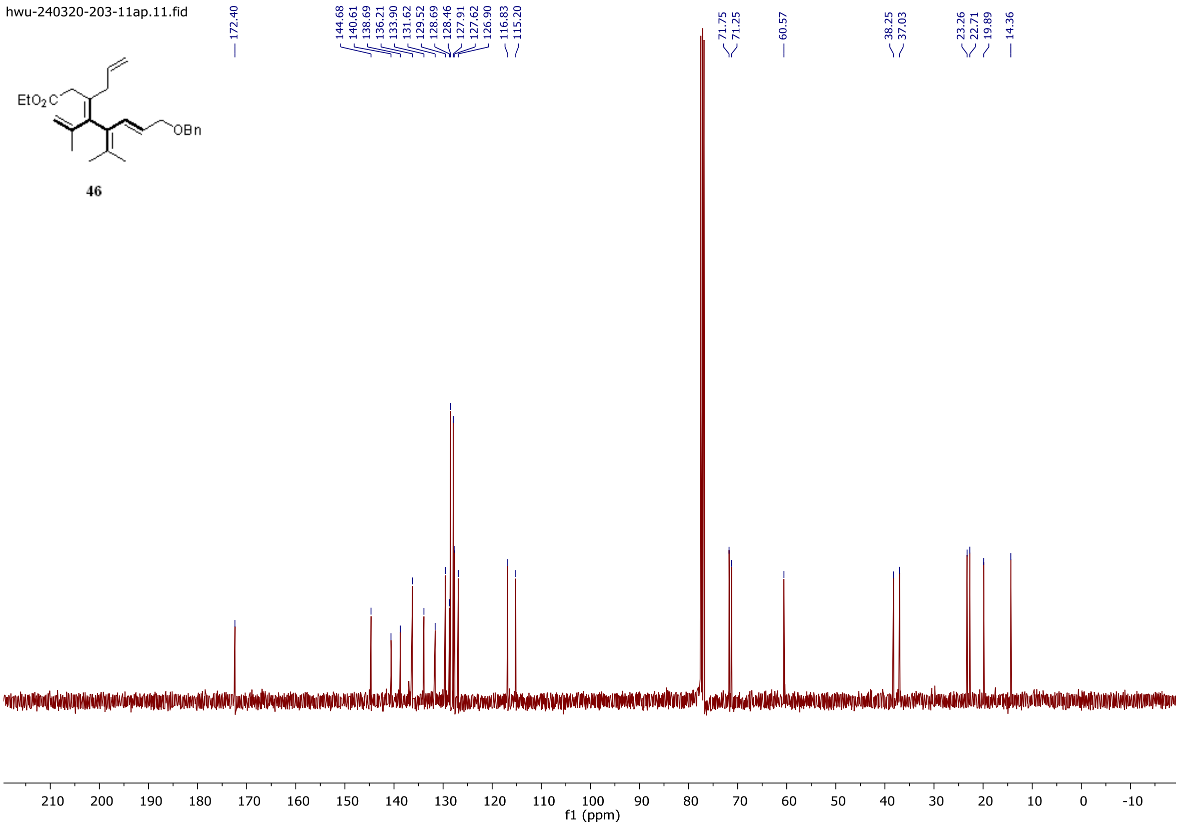
hwu-240320-203-11ap.11.fid
EtO2C
OBn
46
172.40
144.68
140.61
138.69
136.21
133.90
131.62
129.52
128.69
128.46
127.91
127.62
126.90
116.83
115.20
71.75
71.25
60.57
38.25
37.03
23.26
22.71
19.89
14.36
f1 (ppm)

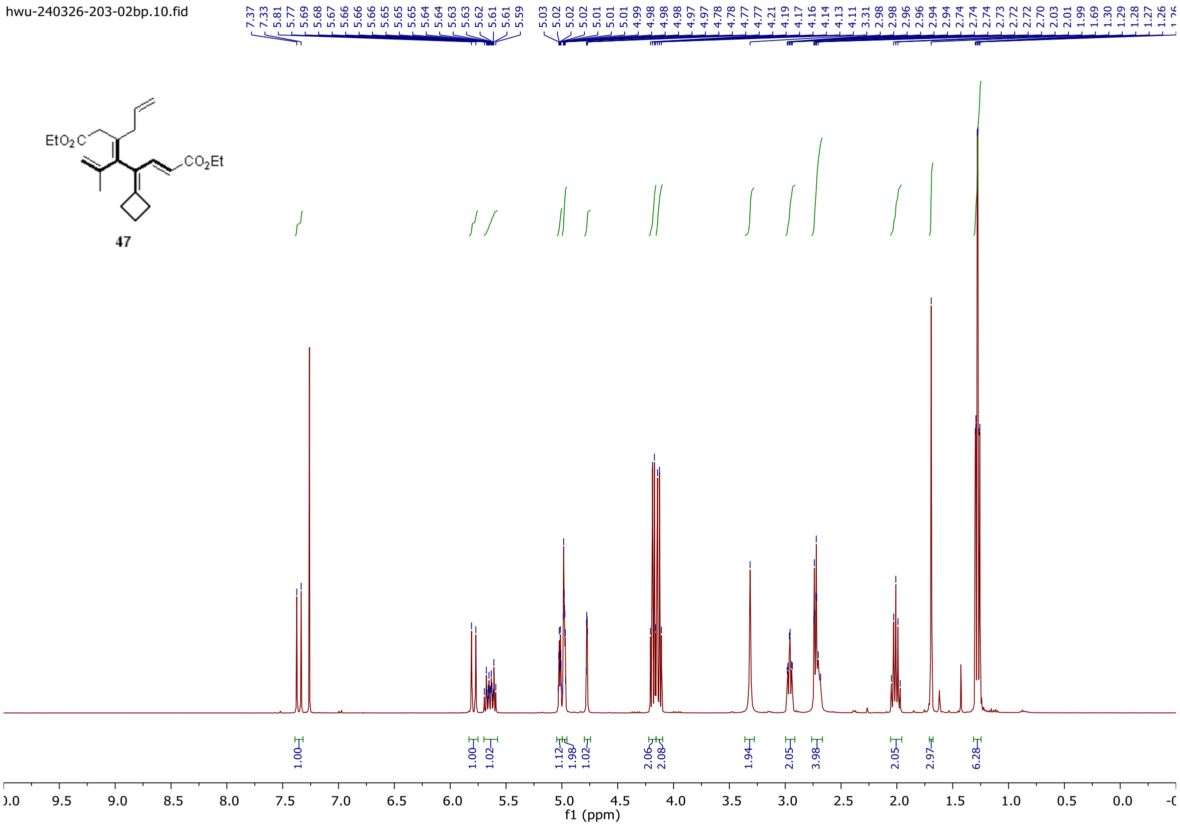
hwu-240326-203-02bp.10.fid
7.37 7.33 5.81 5.77 5.69 5.68 5.67 5.66 5.66 5.65 5.65 5.65 5.64 5.64 5.63 5.63 5.62 5.61 5.61 5.59 5.03 5.02 5.02 5.02 5.01 5.01 5.01 4.99 4.98 4.98 4.98 4.97 4.97 4.78 4.78 4.77 4.77 4.21 4.19 4.17 4.16 4.14 4.13 4.11 3.31 2.98 2.98 2.96 2.96 2.94 2.94 2.74 2.74 2.74 2.73 2.72 2.72 2.70 2.03 2.01 1.99 1.69 1.30 1.29 1.28 1.27 1.26
EtO_2C
CO_2Et
47
1.00 1.00 1.02 1.12 1.98 1.02 2.06 2.08 1.94 2.05 3.98 2.05 2.97 6.28
9.5 9.0 8.5 8.0 7.5 7.0 6.5 6.0 5.5 5.0 4.5 4.0 3.5 3.0 2.5 2.0 1.5 1.0 0.5 0.0
f1 (ppm)

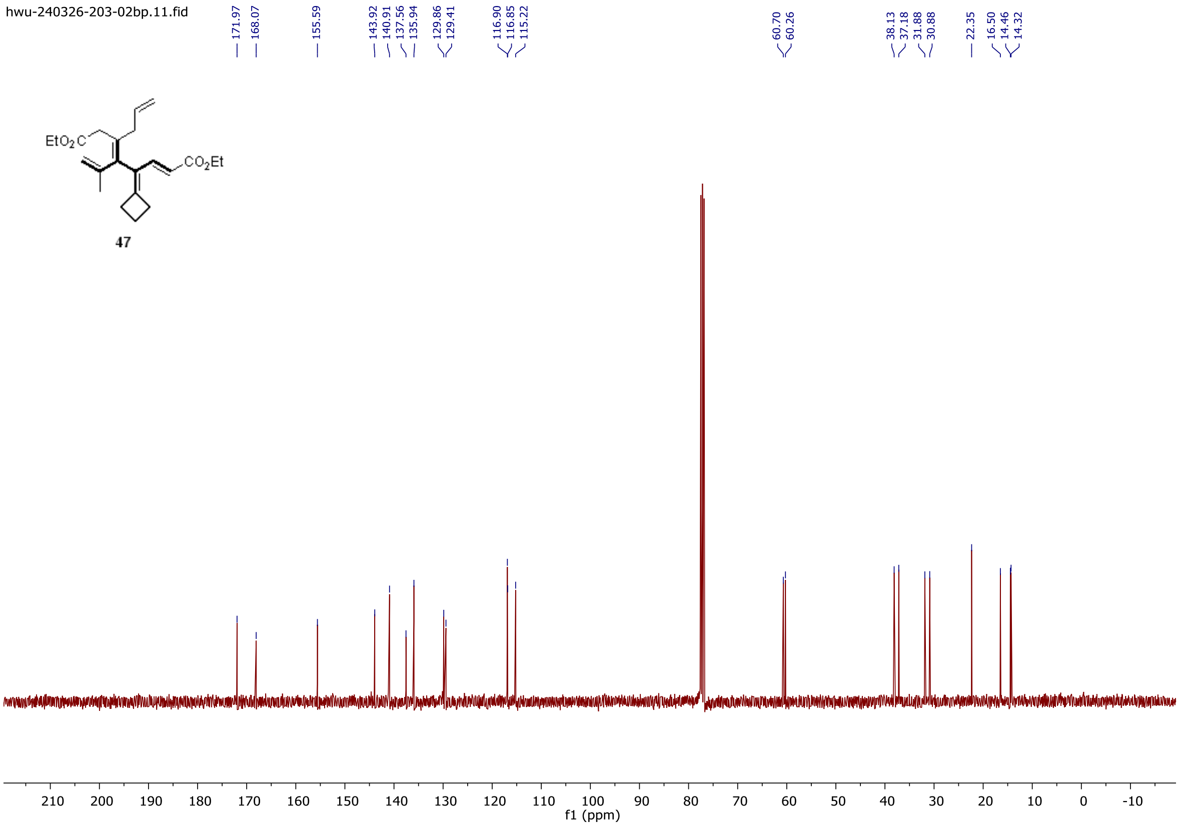
hwu-240326-203-02bp.11.fid
171.97 168.07 155.59 143.92 140.91 137.56 135.94 129.86 129.41 116.90 116.85 115.22 60.70 60.26 38.13 37.18 31.88 30.88 22.35 16.50 14.46 14.32
EtO_2C
CO_2Et
47
210 200 190 180 170 160 150 140 130 120 110 100 90 80 70 60 50 40 30 20 10 0 -10
f1 (ppm)

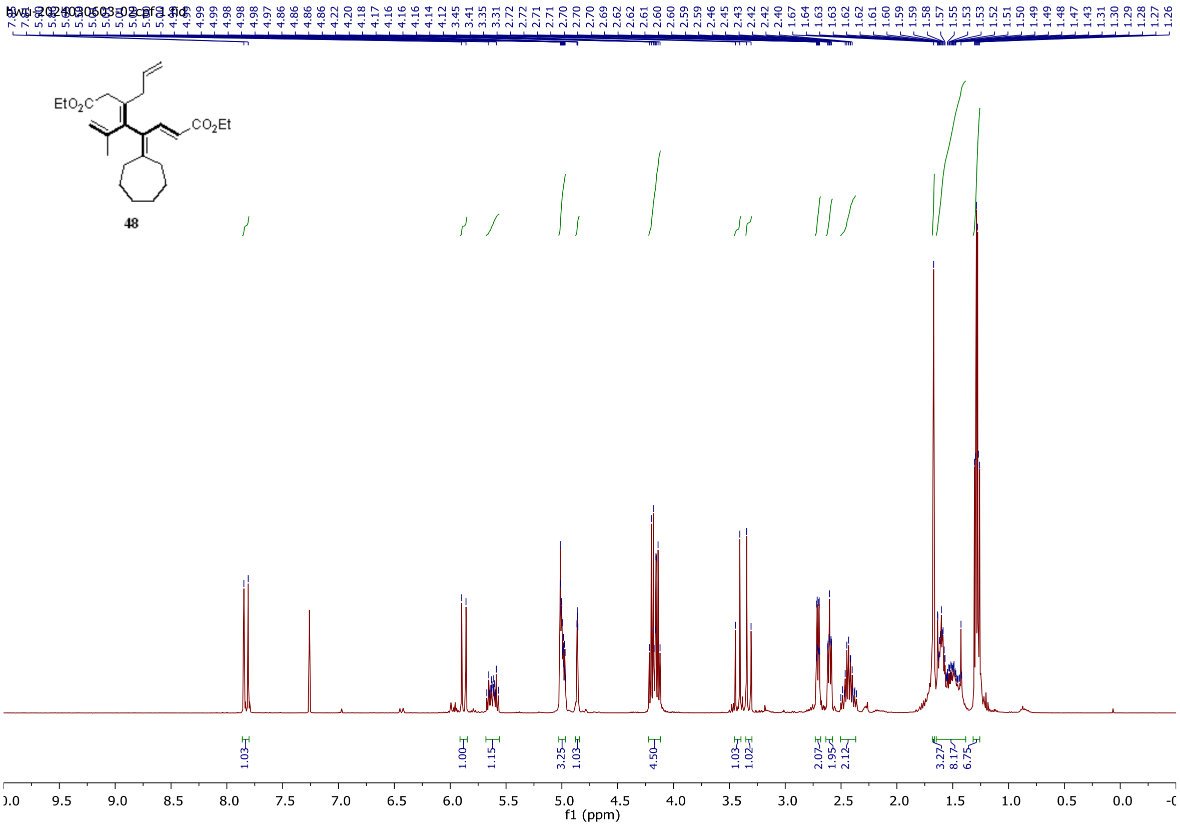
hwu-2024030603-02cpf.1.fid
EtO2C
CO2Et
48
f1 (ppm)

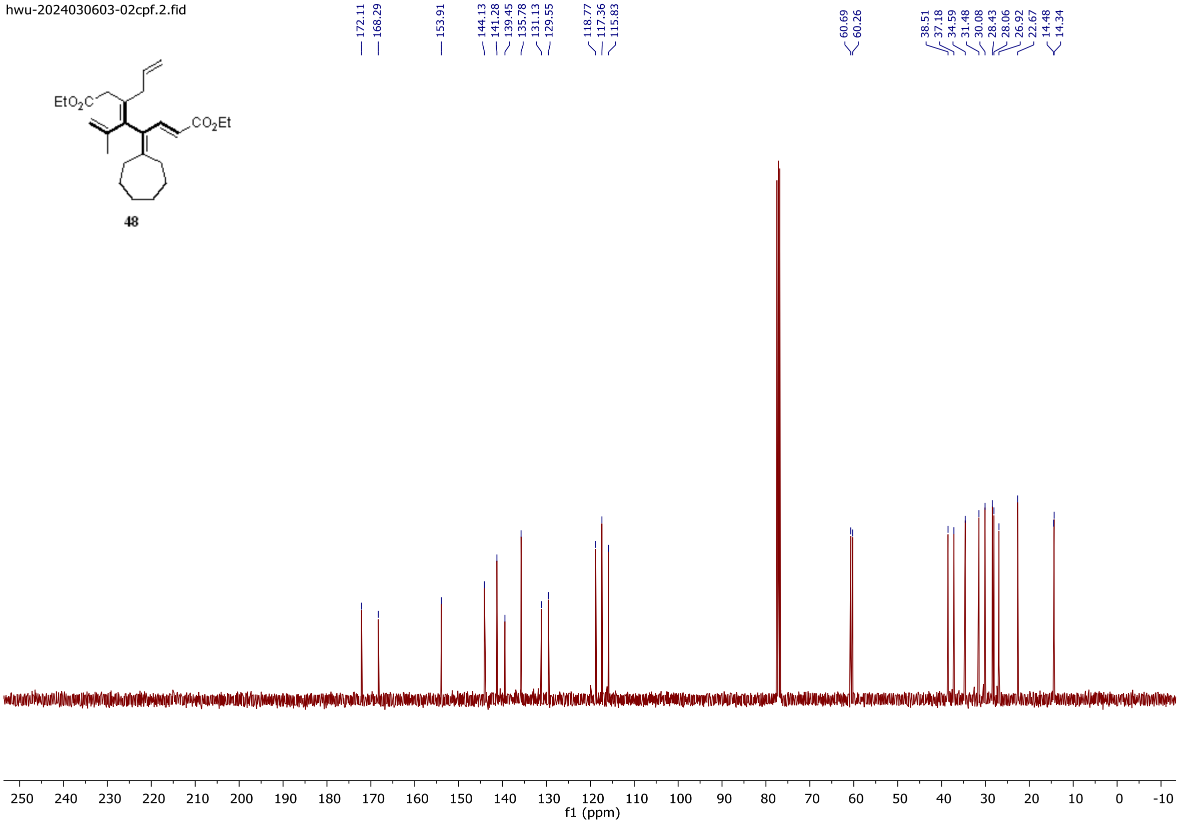
hwu-2024030603-02cpf.2.fid
172.11
168.29
153.91
144.13
141.28
139.45
135.78
131.13
129.55
118.77
117.36
115.83
60.69
60.26
38.51
37.18
34.59
31.48
30.08
28.43
28.06
26.92
22.67
14.48
14.34
EtO2C
CO2Et
48
f1 (ppm)

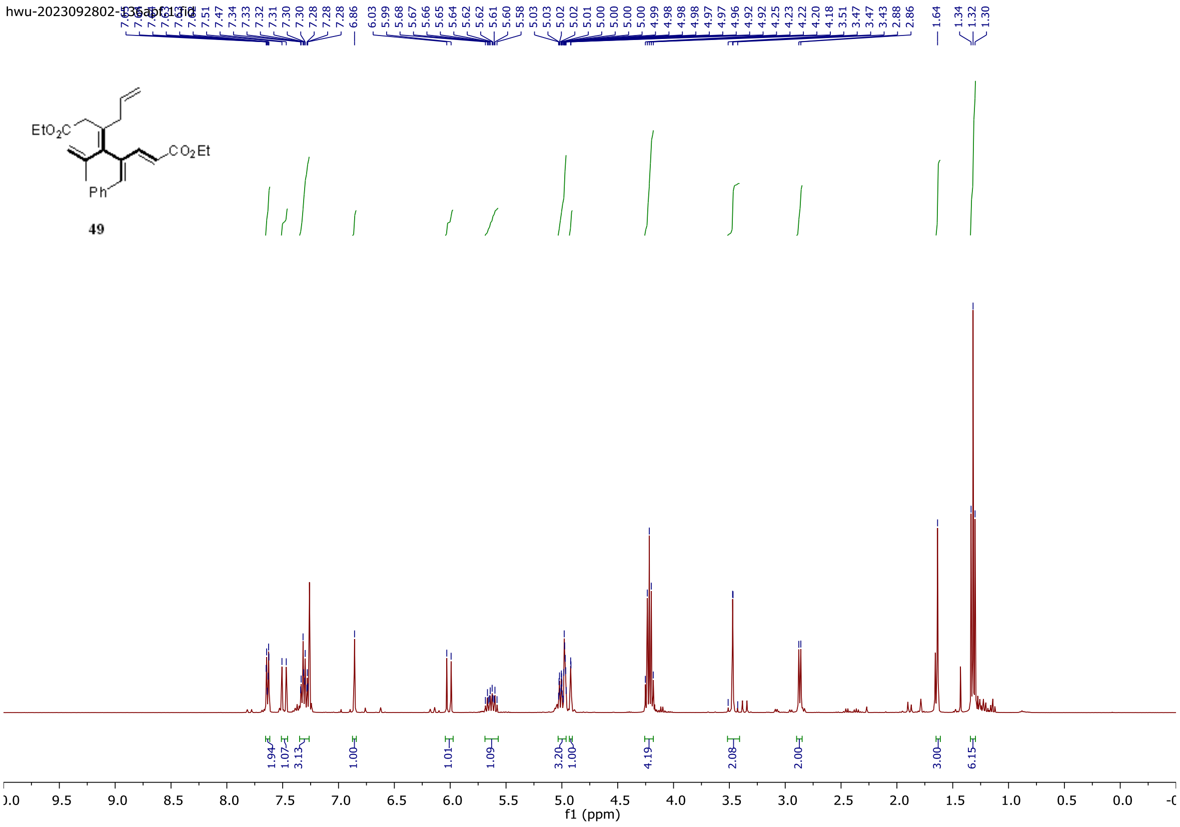

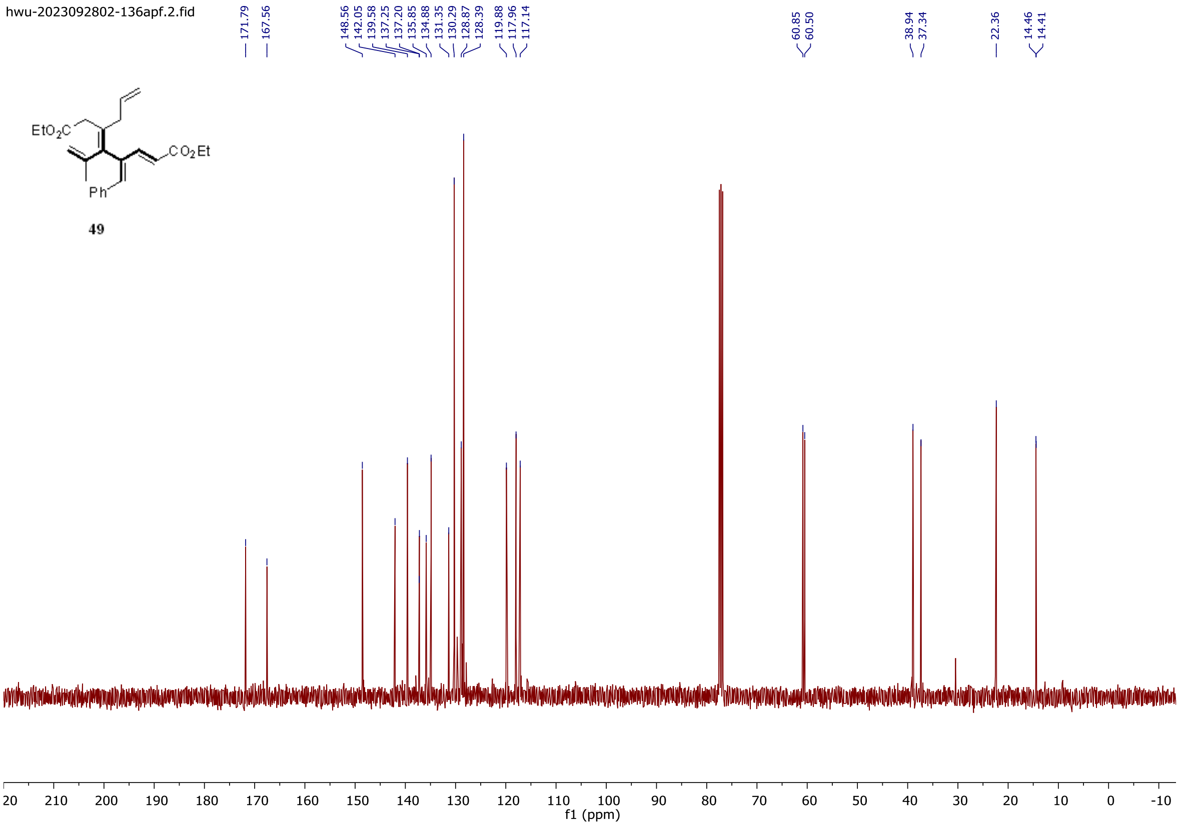

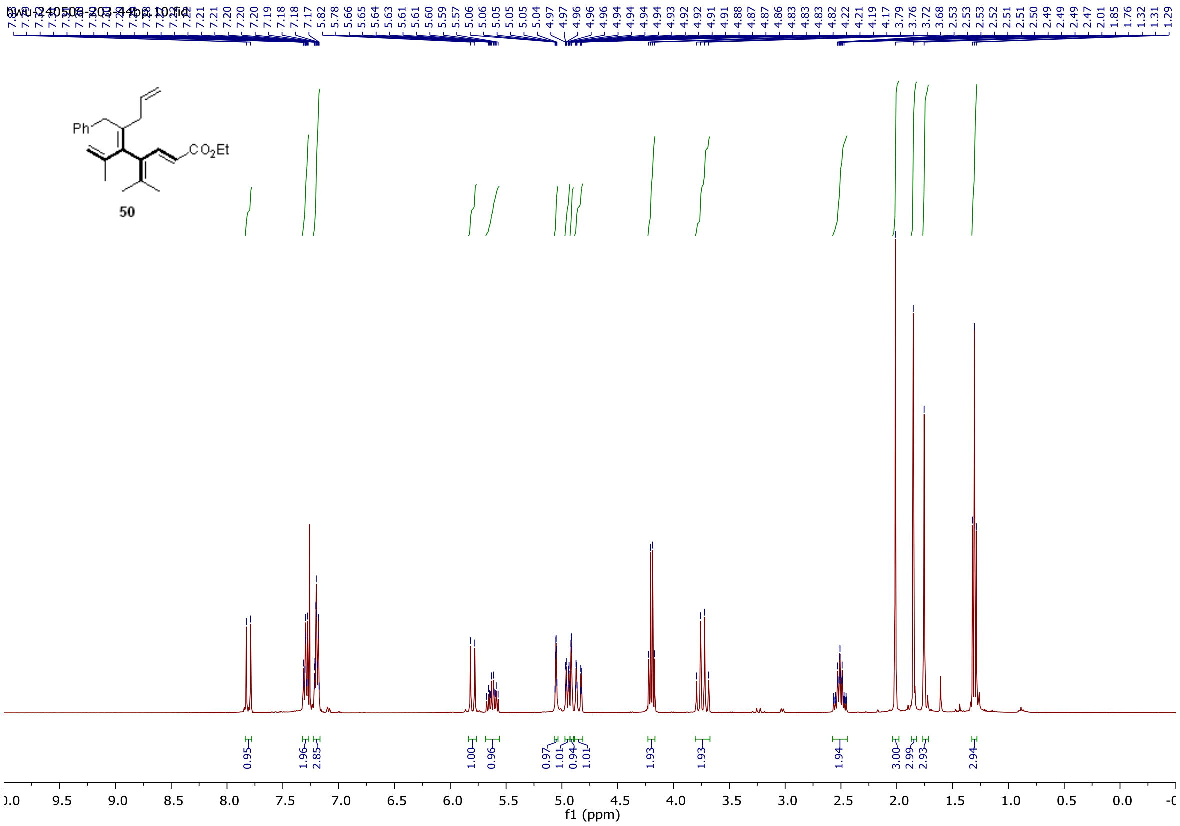
hwu-240506-203-44bp.10.fid
7.21 7.21 7.20 7.20 7.19 7.18 7.17 5.82 5.78 5.66 5.65 5.64 5.63 5.61 5.61 5.60 5.59 5.57 5.06 5.06 5.05 5.05 5.05 5.04 4.97 4.97 4.96 4.96 4.96 4.94 4.94 4.94 4.94 4.93 4.92 4.92 4.91 4.91 4.88 4.87 4.87 4.86 4.83 4.83 4.83 4.82 4.22 4.21 4.19 4.17 3.79 3.76 3.72 3.68 2.53 2.53 2.53 2.52 2.52 2.51 2.51 2.50 2.49 2.49 2.49 2.47 2.01 1.85 1.76 1.32 1.31 1.29
Ph
CO2Et
50
0.95 1.96 2.85 1.00 0.96 0.97 1.01 0.94 1.01 1.93 1.93 1.94 3.00 2.99 2.93 2.94
9.5 9.0 8.5 8.0 7.5 7.0 6.5 6.0 5.5 5.0 4.5 4.0 3.5 3.0 2.5 2.0 1.5 1.0 0.5 0.0
f1 (ppm)

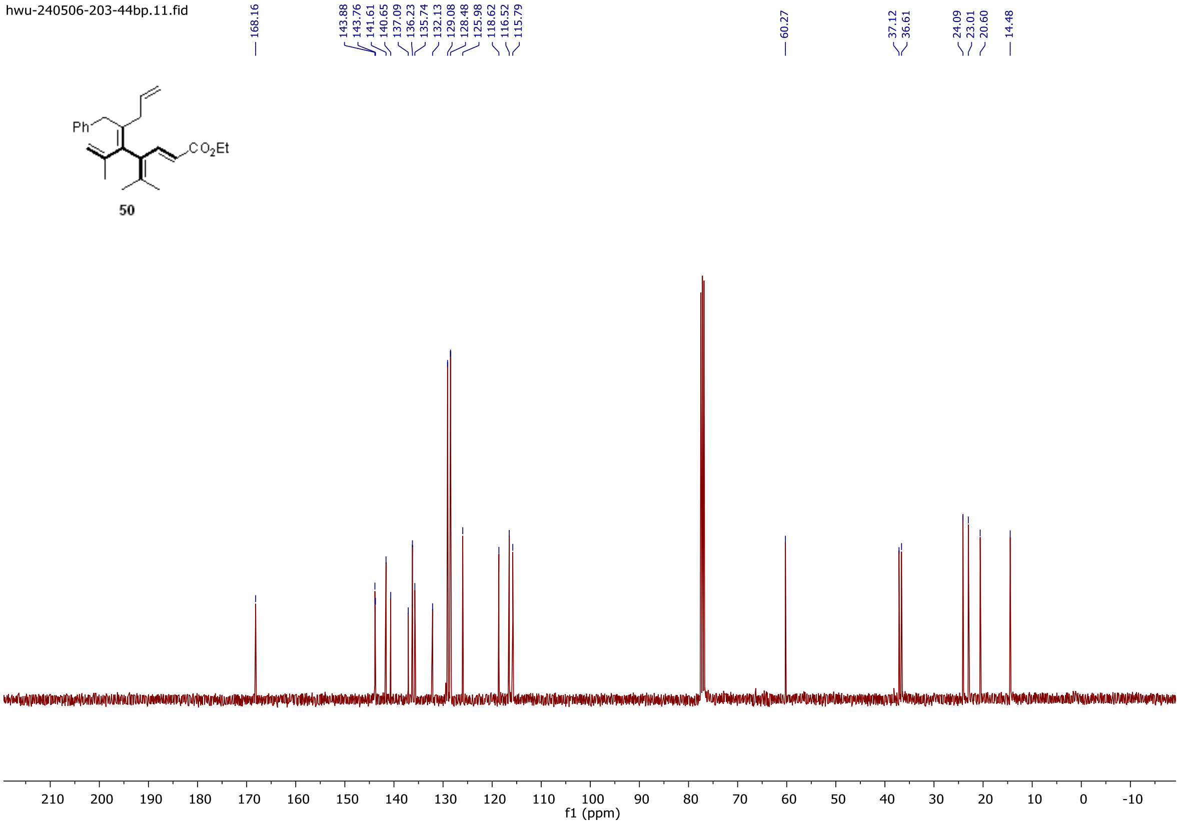
hwu-240506-203-44bp.11.fid
168.16
143.88 143.76 141.61 140.65 137.09 136.23 135.74 132.13 129.08 128.48 125.98 118.62 116.52 115.79
60.27
37.12 36.61
24.09 23.01 20.60
14.48
Ph
CO2Et
50
210 200 190 180 170 160 150 140 130 120 110 100 90 80 70 60 50 40 30 20 10 0 -10
f1 (ppm)

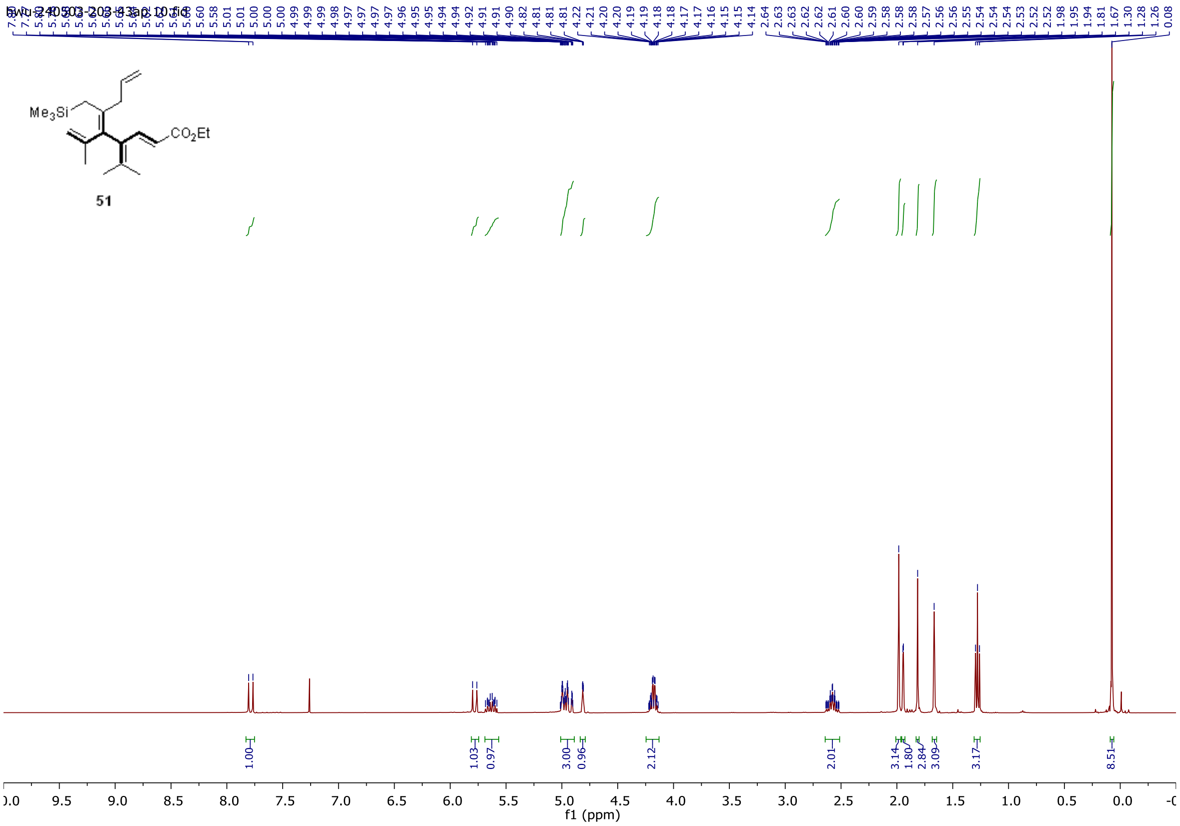
Me3Si
CO2Et
51
1.00
1.03
0.97
3.00
0.96
2.12
2.01
3.14
1.80
2.84
3.09
3.17
8.51
f1 (ppm)

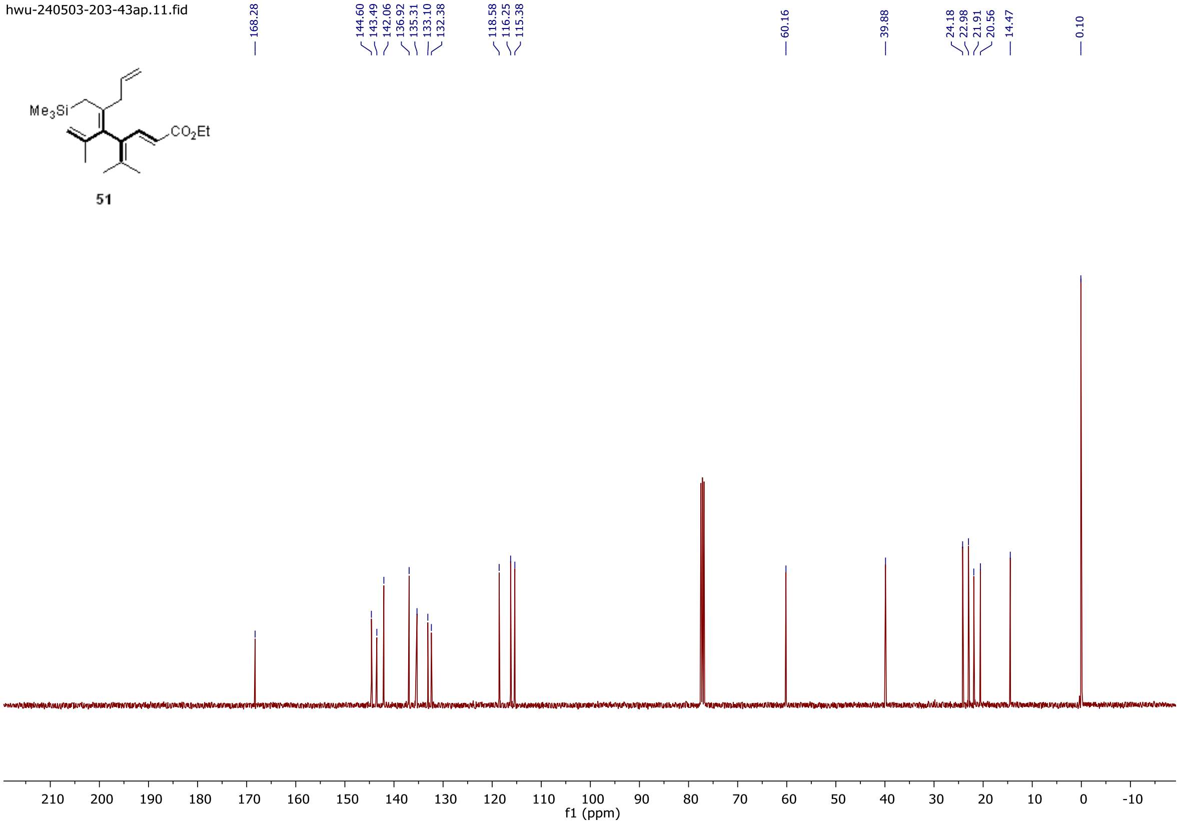
hwu-240503-203-43ap.11.fid
168.28
144.60
143.49
142.06
136.92
135.31
133.10
132.38
118.58
116.25
115.38
60.16
39.88
24.18
22.98
21.91
20.56
14.47
0.10
Me3Si
CO2Et
51
f1 (ppm)

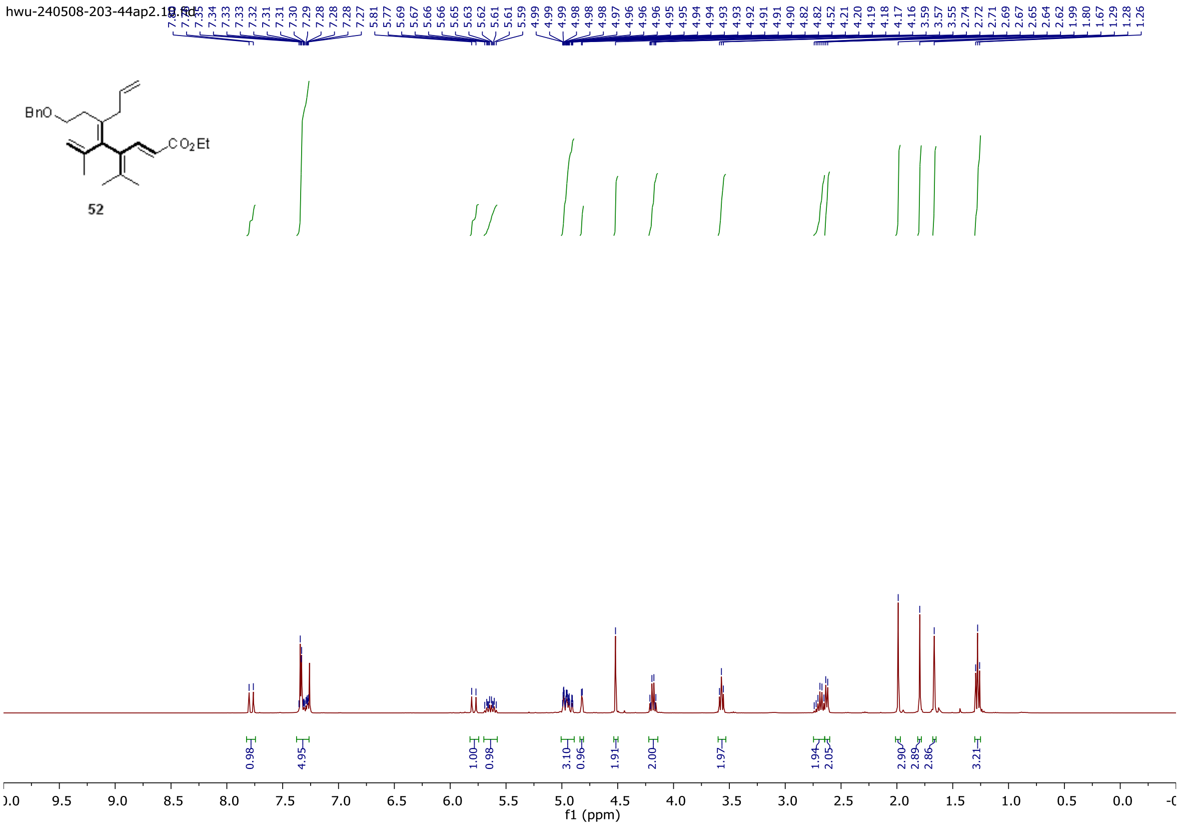

BnO
CO2Et
52
7.35 7.34 7.33 7.33 7.32 7.31 7.30 7.29 7.28 7.28 7.28 7.27 5.81 5.77 5.69 5.67 5.66 5.66 5.65 5.63 5.62 5.61 5.61 5.59 4.99 4.99 4.98 4.98 4.98 4.97 4.96 4.96 4.96 4.95 4.95 4.94 4.94 4.93 4.93 4.92 4.91 4.91 4.90 4.82 4.52 4.21 4.20 4.19 4.18 4.17 4.16 3.59 3.57 3.55 2.74 2.72 2.71 2.69 2.67 2.65 2.64 2.62 1.99 1.80 1.67 1.29 1.28 1.26
0.98 4.95 1.00 0.98 3.10 0.96 1.91 2.00 1.97 1.94 2.05 2.90 2.89 2.86 3.21
9.5 9.0 8.5 8.0 7.5 7.0 6.5 6.0 5.5 5.0 4.5 4.0 3.5 3.0 2.5 2.0 1.5 1.0 0.5 0.0
f1 (ppm)

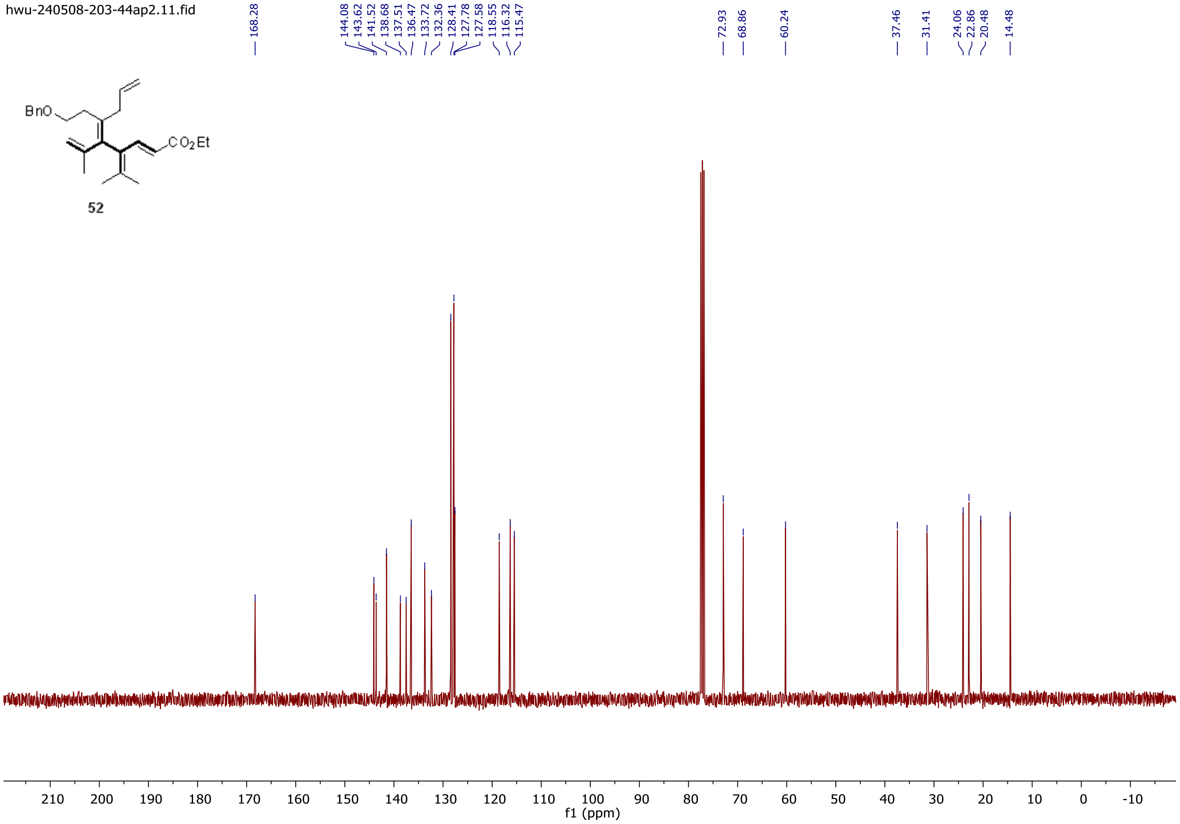

hwu-240508-203-44ap2.11.fid
168.28
144.08 143.62 141.52 138.68 137.51 136.47 133.72 132.36 128.41 127.78 127.58 118.55 116.32 115.47
72.93 68.86 60.24
37.46 31.41 24.06 22.86 20.48 14.48
BnO
CO2Et
52
210 200 190 180 170 160 150 140 130 120 110 100 90 80 70 60 50 40 30 20 10 0 -10
f1 (ppm)

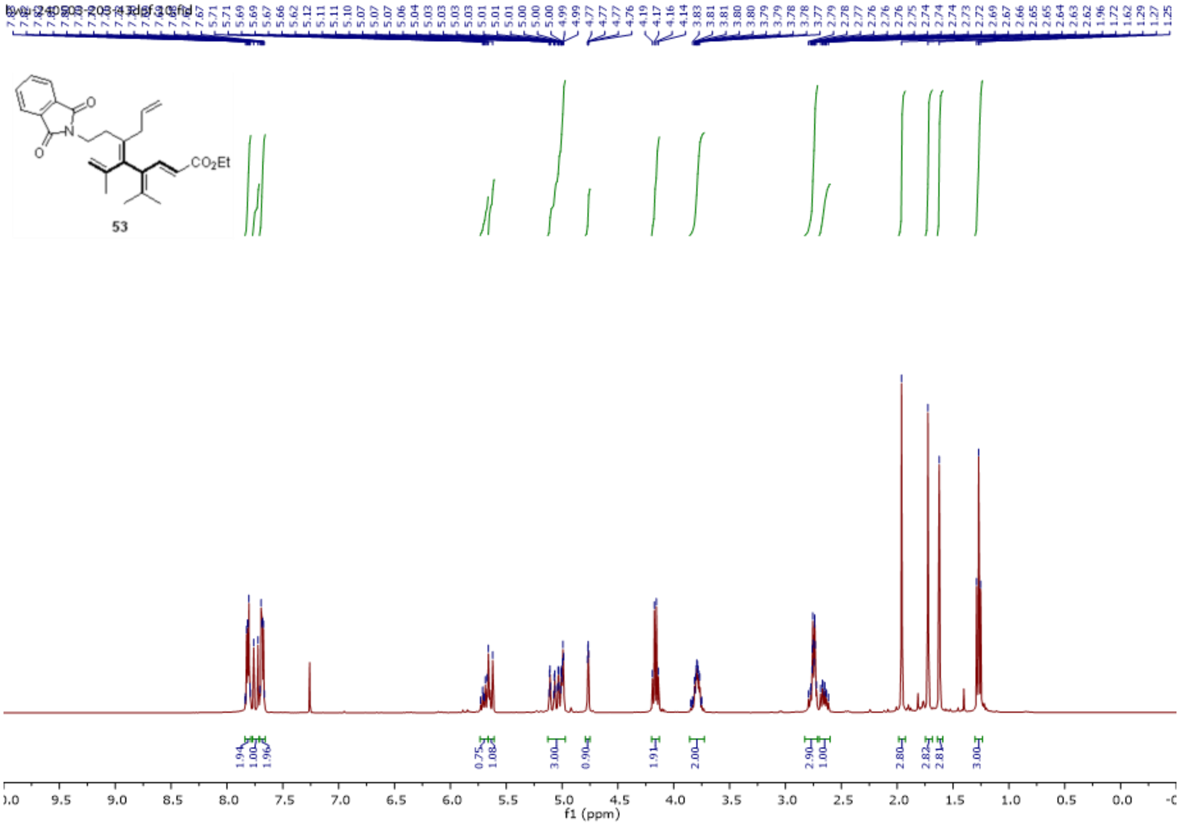
CO2Et
53
f1 (ppm)

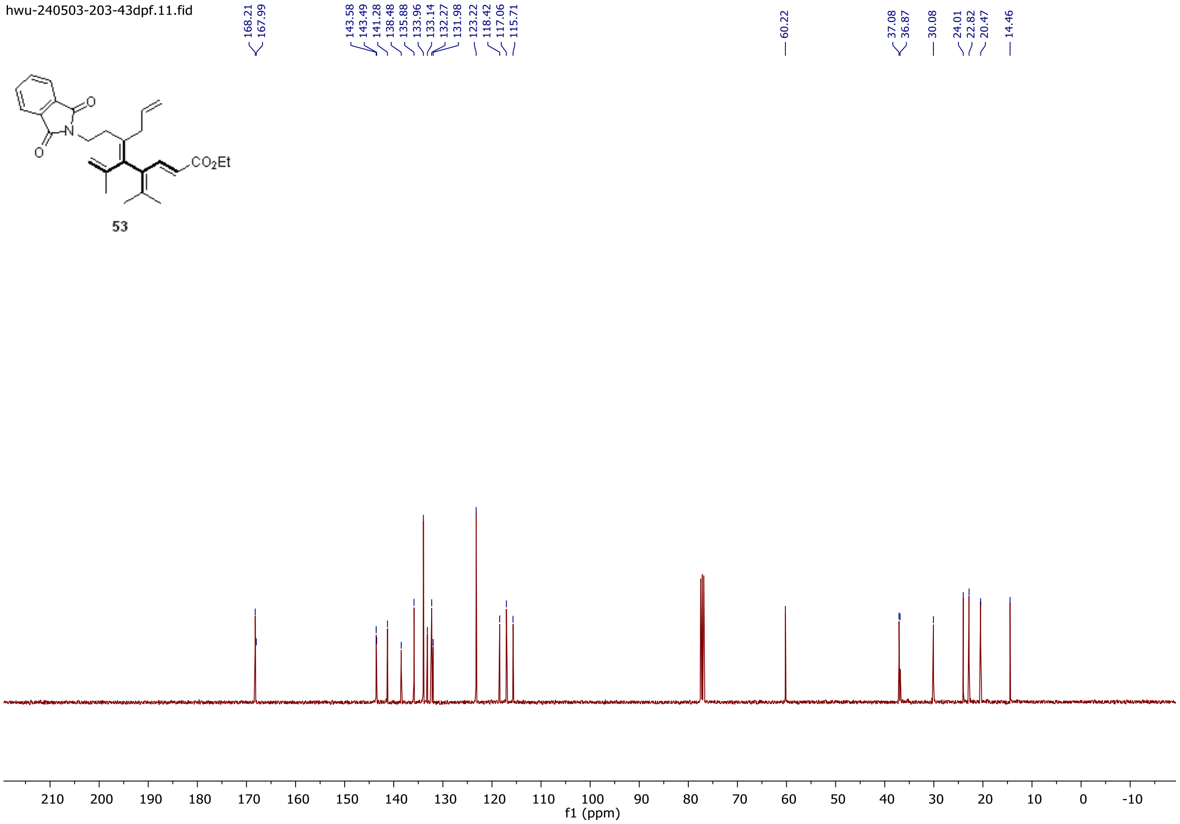
hwu-240503-203-43dpf.11.fid
168.21
167.99
143.58
143.49
141.28
138.48
135.88
133.96
133.14
132.27
131.98
123.22
118.42
117.06
115.71
60.22
37.08
36.87
30.08
24.01
22.82
20.47
14.46
CO2Et
53
f1 (ppm)

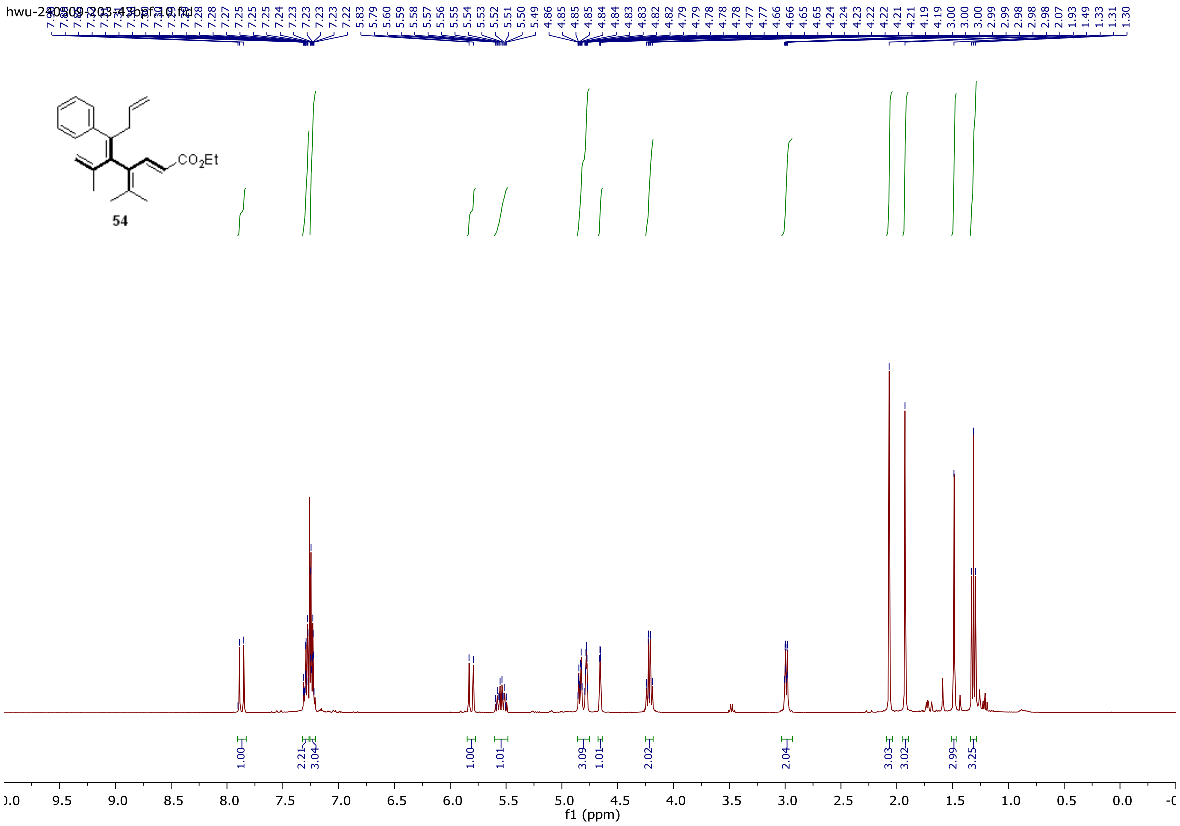
hwu-240509-203-43bpf.10.fid
CO2Et
54
f1 (ppm)

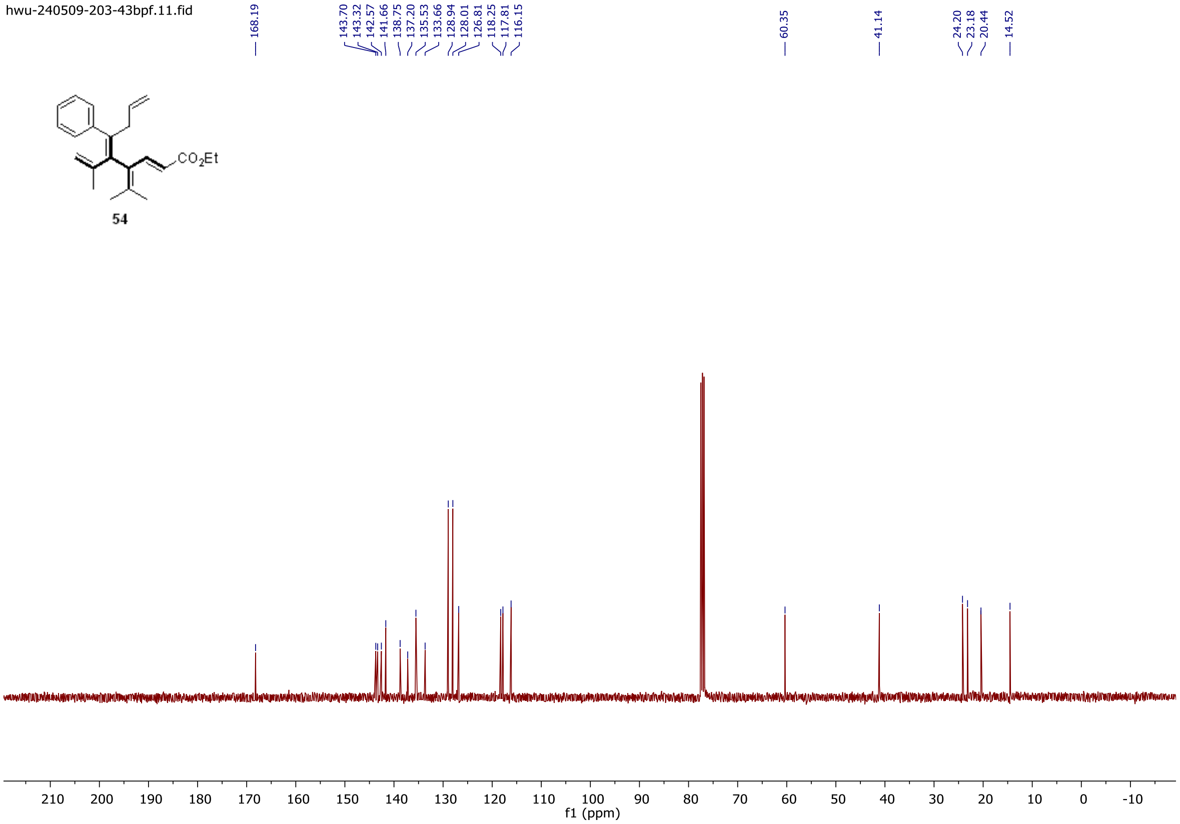
hwu-240509-203-43bpf.11.fid
168.19
143.70
143.32
142.57
141.66
138.75
137.20
135.53
133.66
128.94
128.01
126.81
118.25
117.81
116.15
60.35
41.14
24.20
23.18
20.44
14.52
CO2Et
54
f1 (ppm)

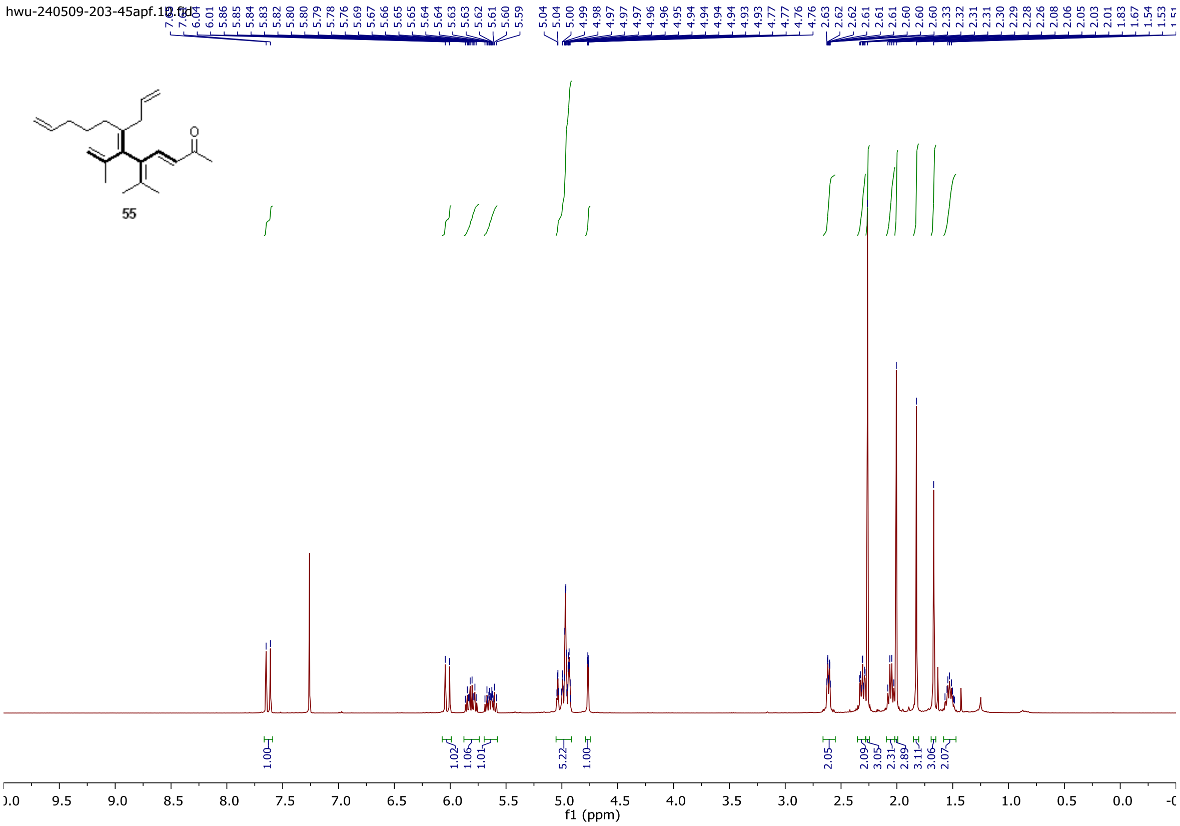
hwu-240509-203-45apf.10.fid
6.04 6.01 5.86 5.85 5.84 5.83 5.82 5.80 5.80 5.79 5.78 5.76 5.69 5.67 5.66 5.65 5.65 5.64 5.64 5.63 5.63 5.62 5.61 5.60 5.59 5.04 5.04 5.00 4.99 4.98 4.97 4.97 4.97 4.96 4.95 4.94 4.94 4.94 4.93 4.93 4.77 4.76 2.63 2.62 2.62 2.61 2.61 2.61 2.60 2.60 2.33 2.32 2.31 2.30 2.29 2.28 2.26 2.08 2.06 2.05 2.03 2.01 1.83 1.67 1.54 1.53
O
55
1.00 1.02 1.06 1.01 5.22 1.00 2.05 2.09 3.05 2.31 2.89 3.11 3.06 2.07
9.5 9.0 8.5 8.0 7.5 7.0 6.5 6.0 5.5 5.0 4.5 4.0 3.5 3.0 2.5 2.0 1.5 1.0 0.5 0.0
f1 (ppm)

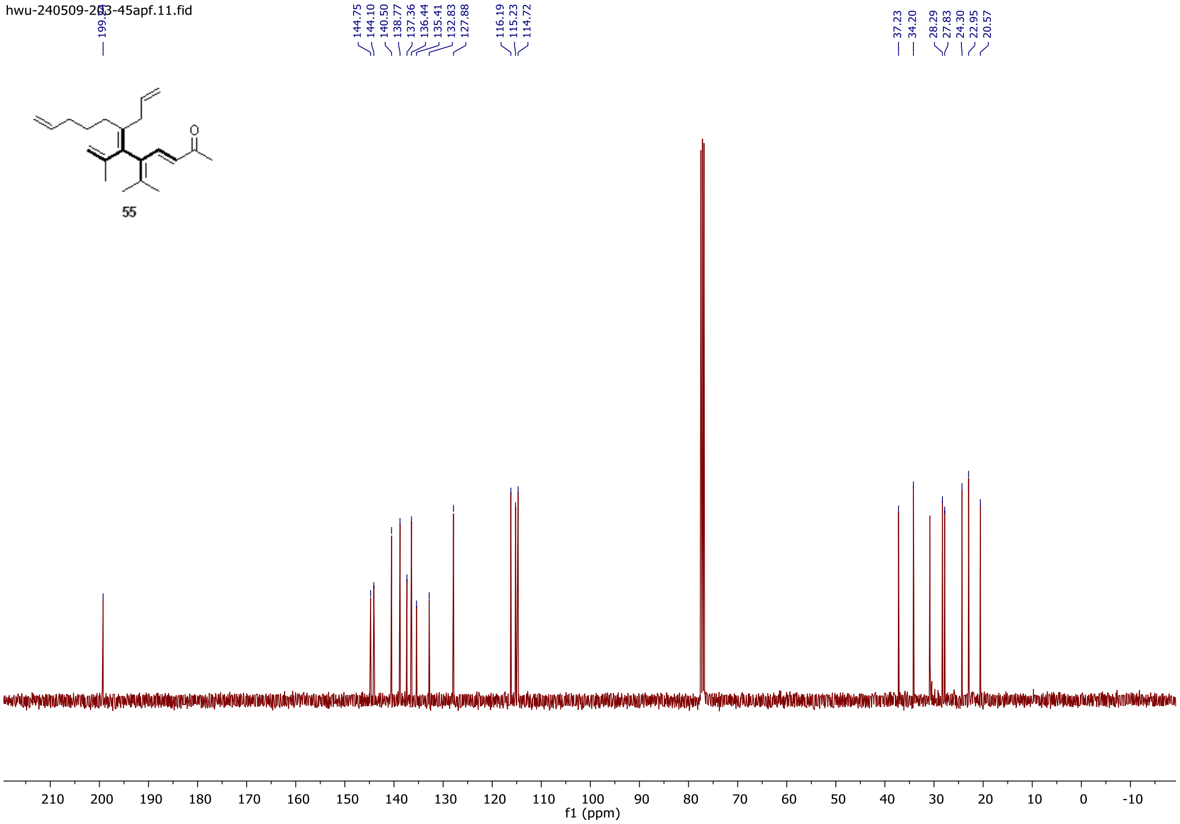
hwu-240509-203-45apf.11.fid
144.75 144.10 140.50 138.77 137.36 136.44 135.41 132.83 127.88
116.19 115.23 114.72
37.23 34.20 28.29 27.83 24.30 22.95 20.57
O
55
210 200 190 180 170 160 150 140 130 120 110 100 90 80 70 60 50 40 30 20 10 0 -10
f1 (ppm)

hwu-240509-203-45bp.10.fid

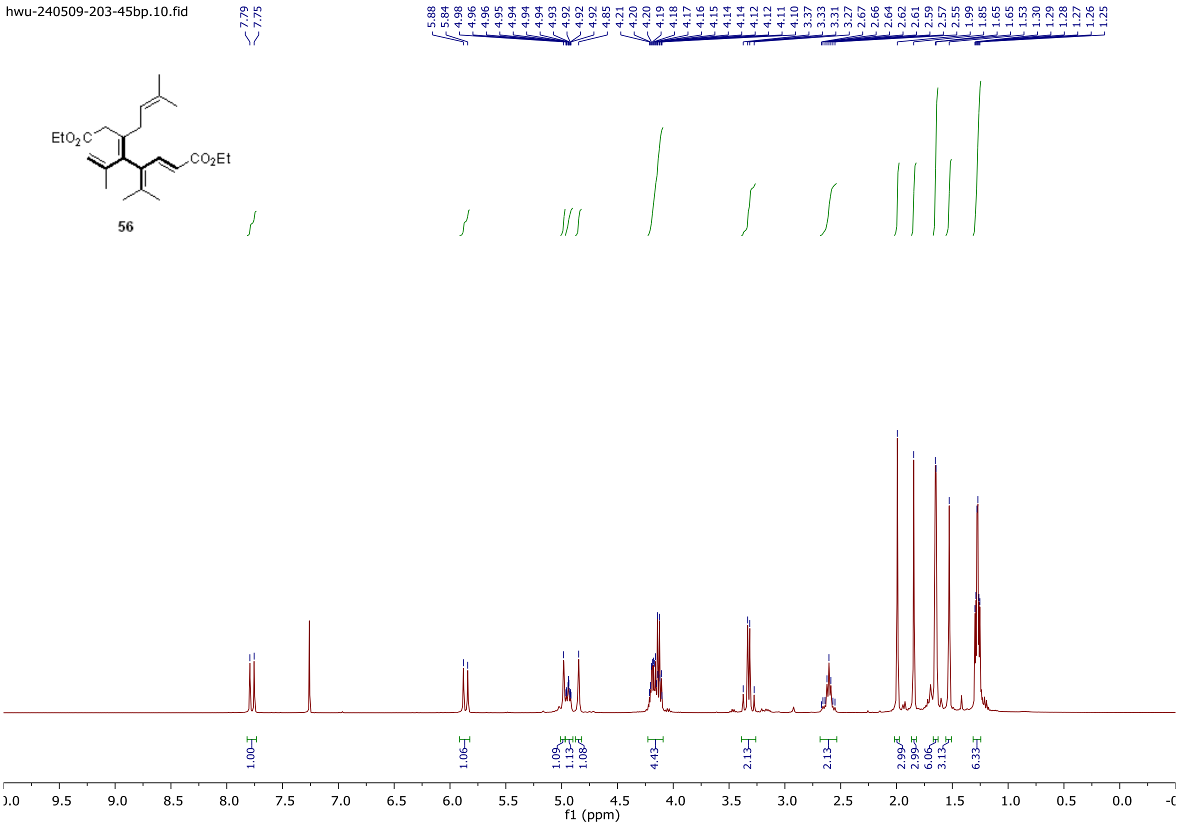

hwu-240509-203-45bp.11.fid

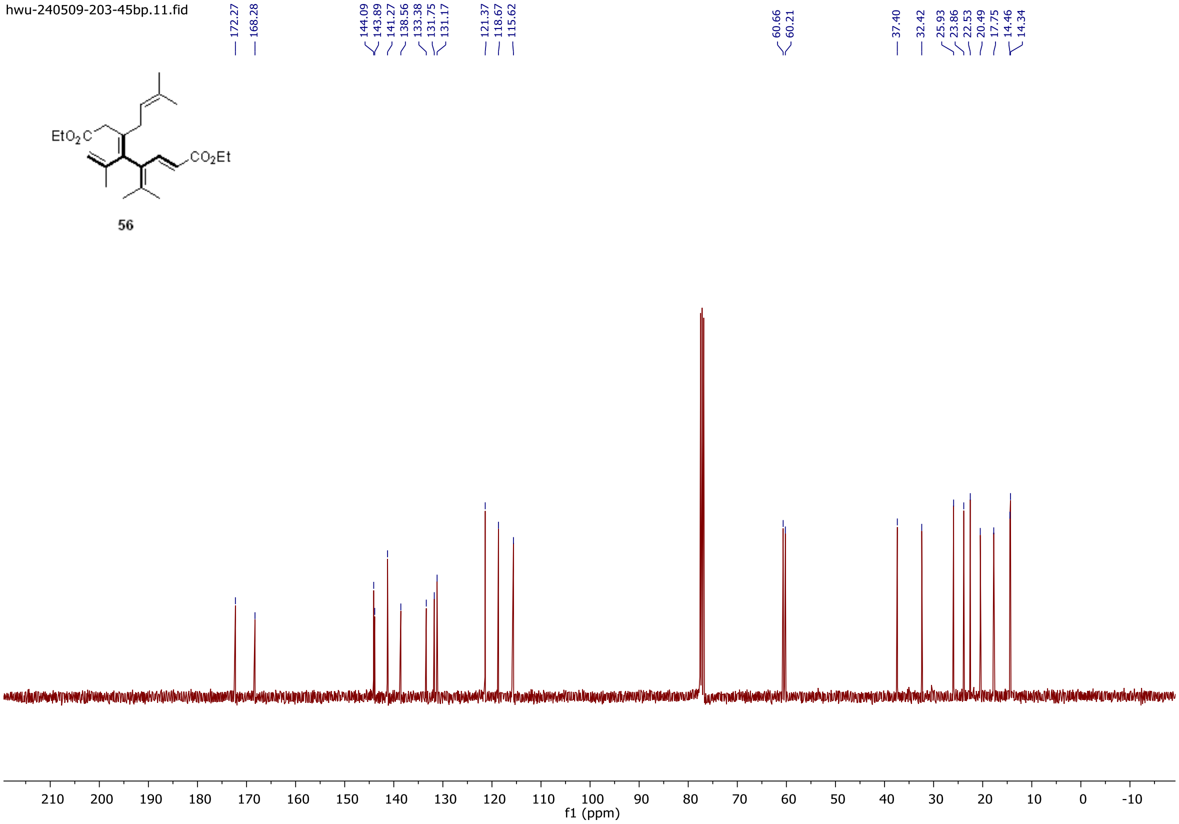

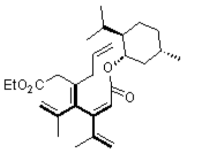

EtO2C
O
O

57

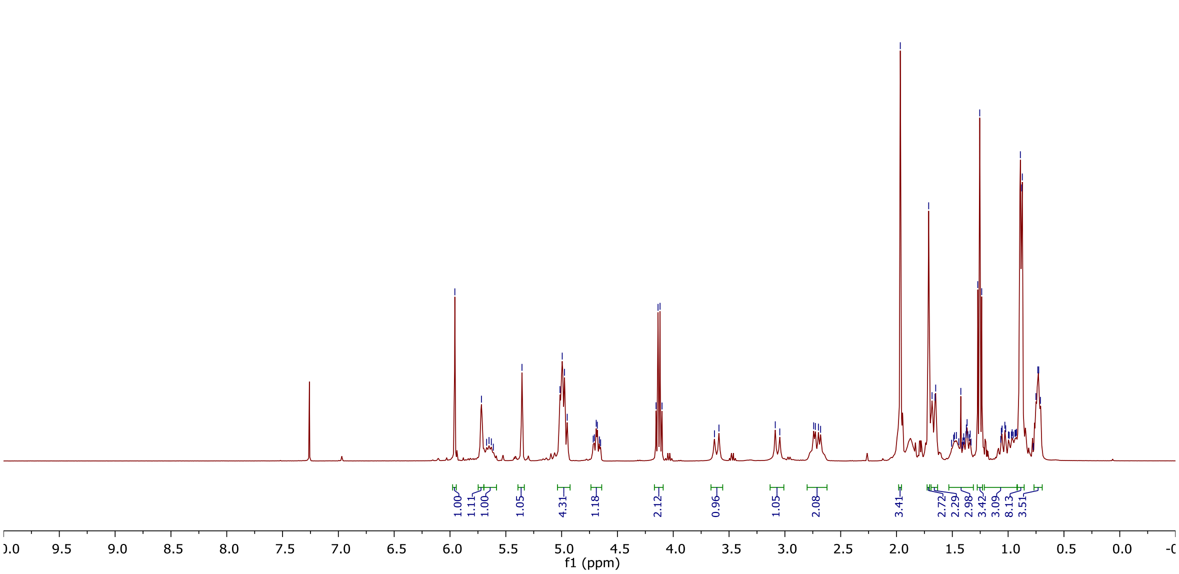

hwu-231213-202-174pf.10.fid
5.67
5.65
5.63
5.61
5.36
5.01
4.99
4.97
4.95
4.72
4.71
4.69
4.68
4.66
4.65
4.15
4.14
4.12
4.10
3.63
3.59
3.09
3.05
2.74
2.73
2.70
2.68
1.97
1.71
1.68
1.66
1.65
1.51
1.49
1.48
1.46
1.44
1.42
1.41
1.41
1.40
1.39
1.38
1.37
1.37
1.36
1.35
1.34
1.33
1.27
1.25
1.24
1.07
1.06
1.05
1.03
1.02
1.00
0.99
0.97
0.96
0.95
0.94
0.93
0.93
0.89
0.88
0.87
0.77
0.75
0.73
0.73
0.71
1.00
1.11
1.00
1.05
4.31
1.18
2.12
0.96
1.05
2.08
3.41
2.72
2.29
2.98
3.42
3.09
8.13
3.51
9.5
9.0
8.5
8.0
7.5
7.0
6.5
6.0
5.5
5.0
4.5
4.0
3.5
3.0
2.5
2.0
1.5
1.0
0.5
0.0
f1 (ppm)

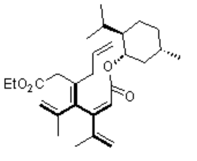

EtO2C
O
O

57

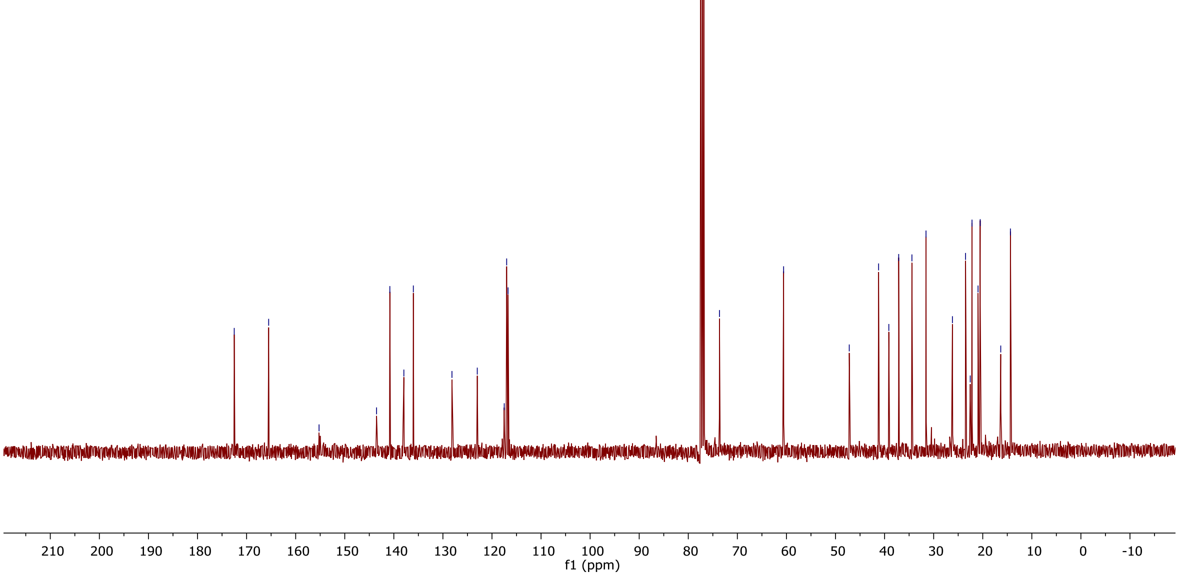

hwu-231213-202-174pf.11.fid
172.52
165.50
155.23
143.51
140.81
137.96
136.02
128.15
123.00
117.50
117.01
116.72
73.64
60.57
47.20
41.22
39.14
37.13
34.43
31.56
26.18
23.50
22.55
22.18
20.95
20.52
16.33
14.36
210
200
190
180
170
160
150
140
130
120
110
100
90
80
70
60
50
40
30
20
10
0
-10
f1 (ppm)

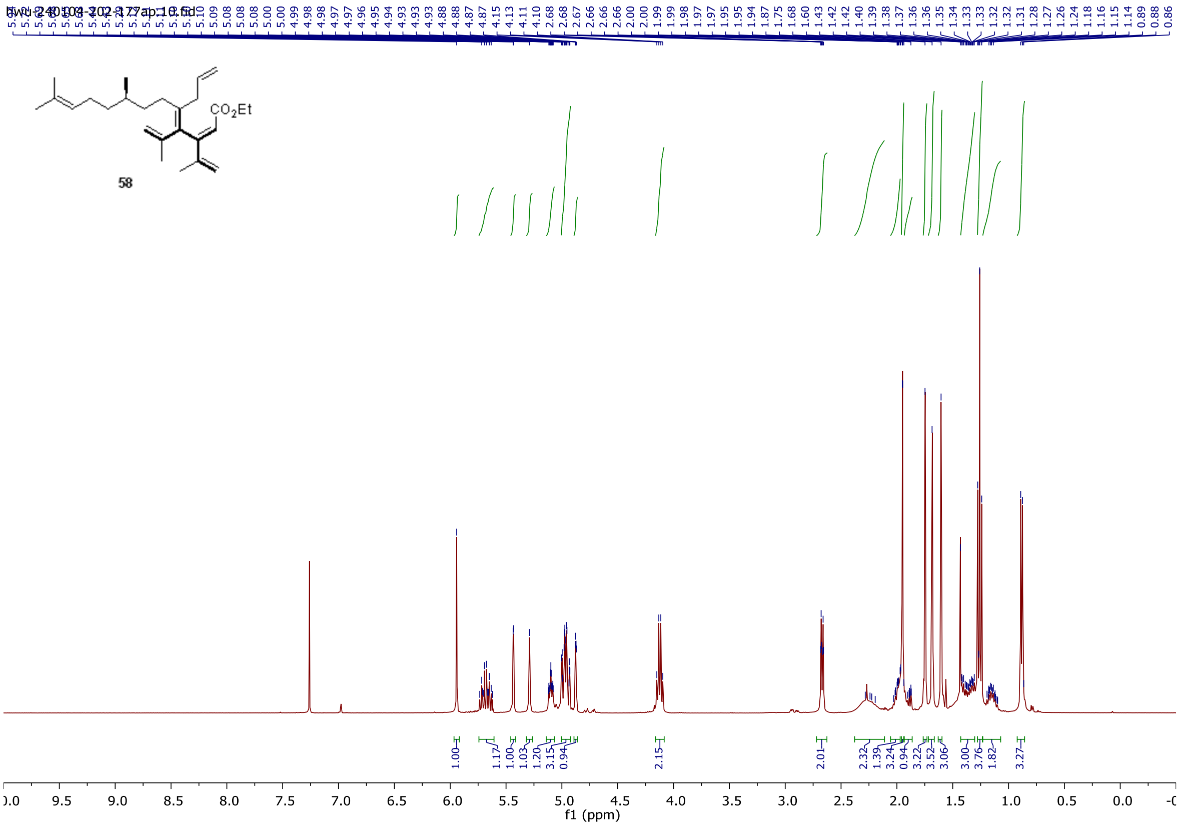
5.10 5.09 5.08 5.08 5.08 5.00 4.99 4.98 4.98 4.97 4.97 4.96 4.95 4.94 4.93 4.93 4.93 4.88 4.87 4.87 4.15 4.13 4.11 4.10 2.68 2.68 2.67 2.66 2.66 2.66 2.00 1.99 1.99 1.98 1.97 1.97 1.95 1.95 1.94 1.87 1.75 1.68 1.60 1.43 1.42 1.40 1.39 1.38 1.37 1.36 1.36 1.35 1.34 1.33 1.33 1.32 1.32 1.31 1.28 1.27 1.26 1.24 1.18 1.16 1.15 1.14 0.89 0.88 0.86
CO_2Et
58
f1 (ppm)

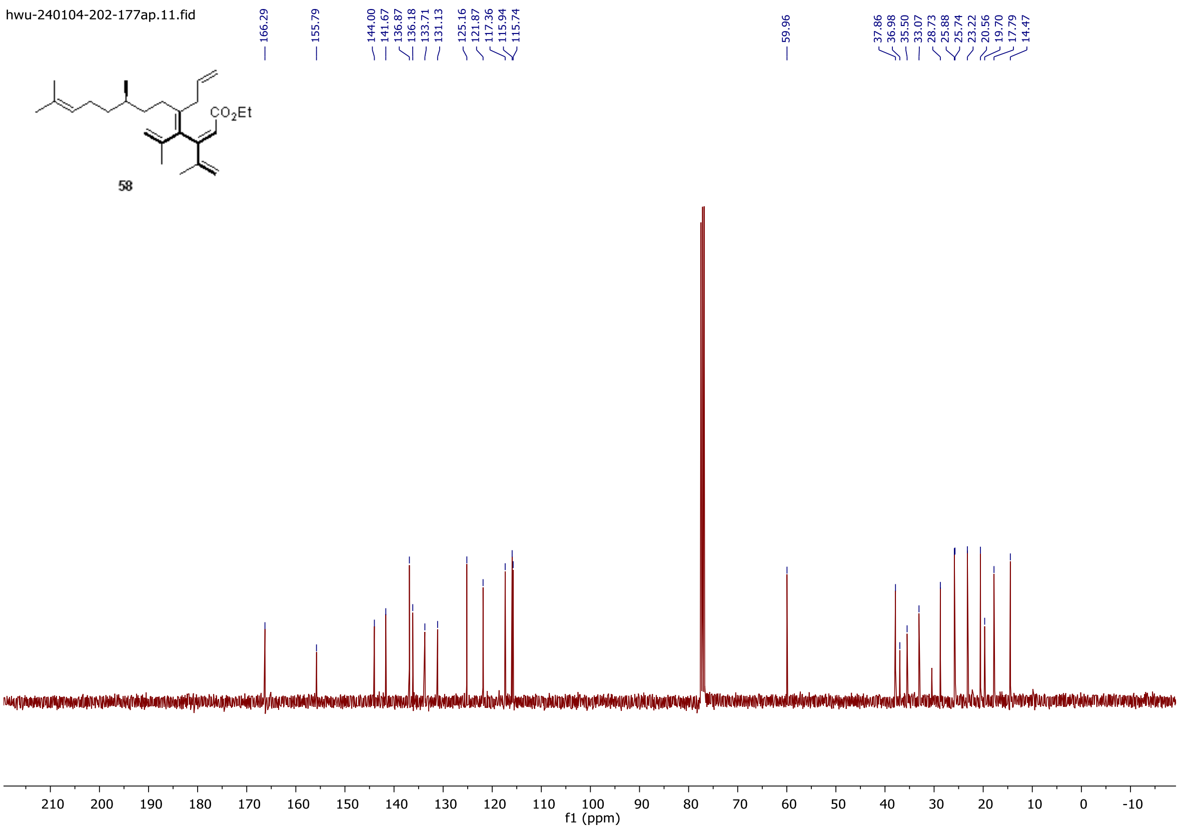
hwu-240104-202-177ap.11.fid
166.29
155.79
144.00 141.67 136.87 136.18 133.71 131.13
125.16 121.87 117.36 115.94 115.74
59.96
37.86 36.98 35.50 33.07 28.73 25.88 25.74 23.22 20.56 19.70 17.79 14.47
CO_2Et
58
f1 (ppm)

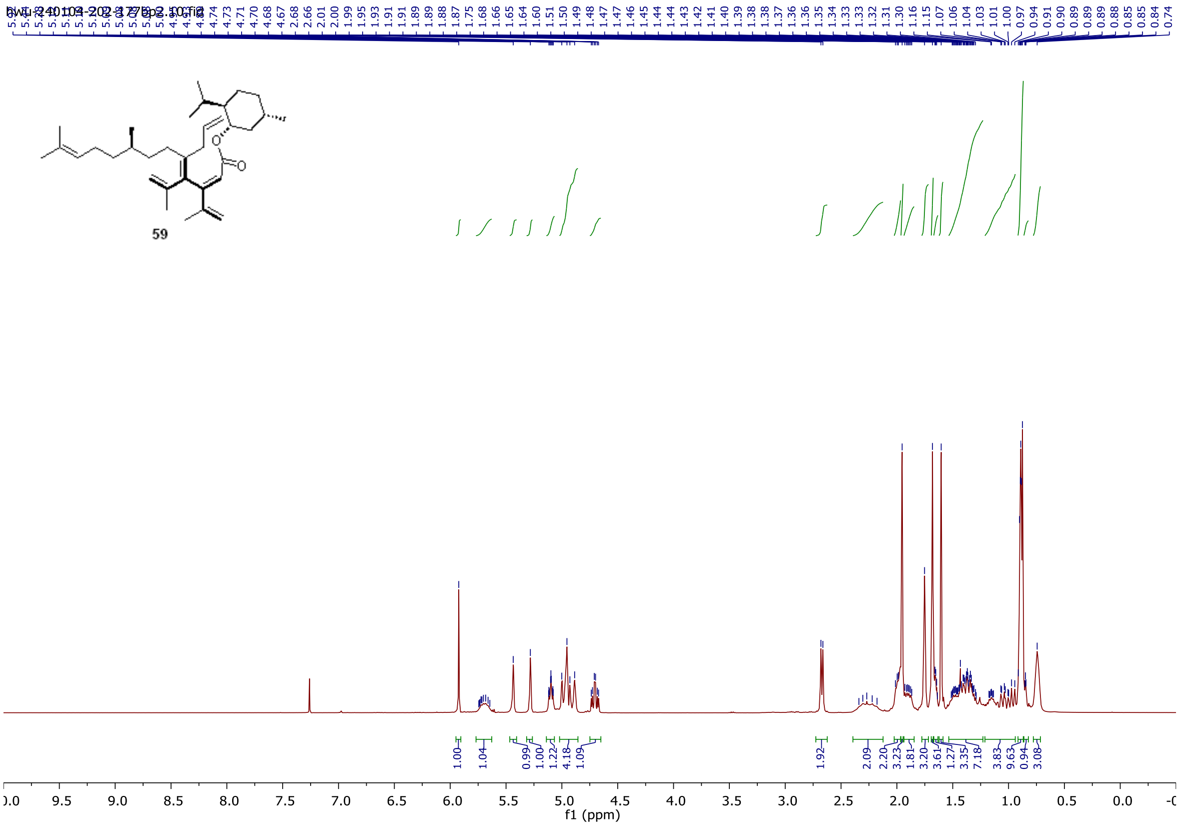
59
f1 (ppm)

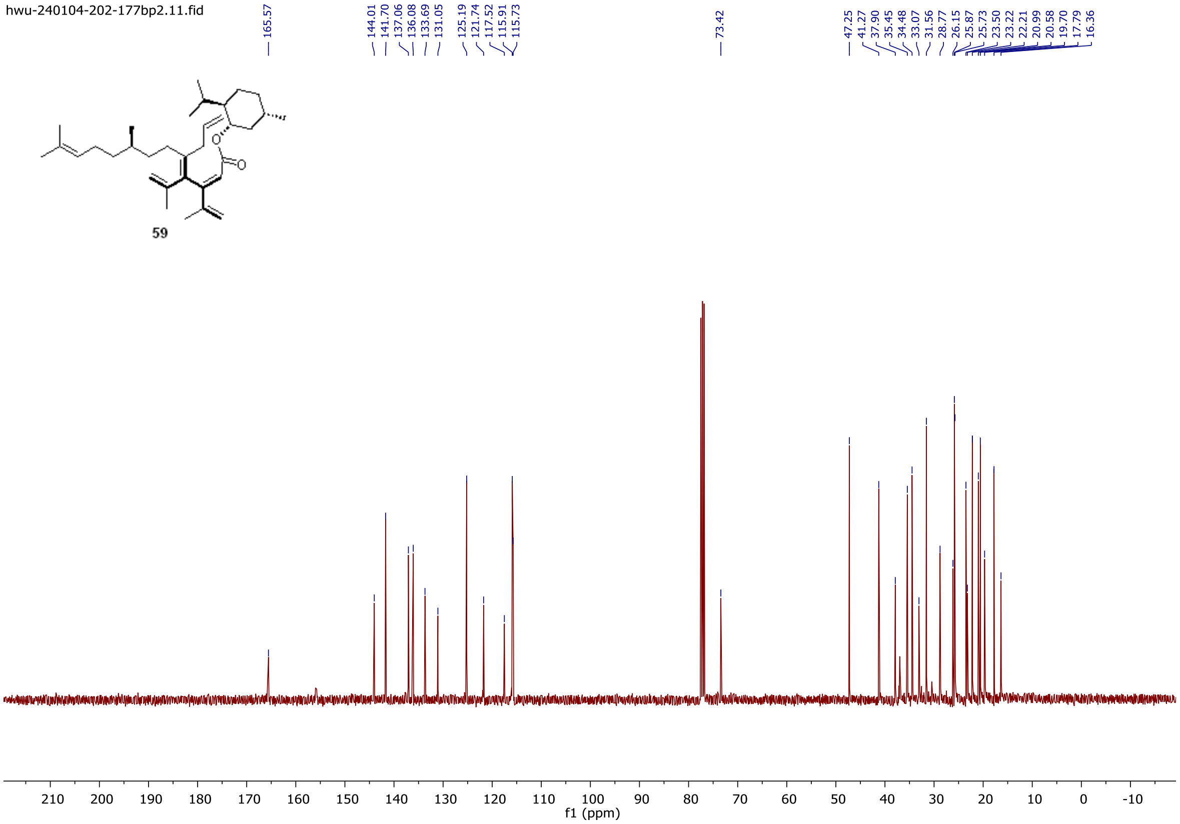
hwu-240104-202-177bp2.11.fid
165.57
144.01
141.70
137.06
136.08
133.69
131.05
125.19
121.74
117.52
115.91
115.73
73.42
47.25
41.27
37.90
35.45
34.48
33.07
31.56
28.77
26.15
25.87
25.73
23.50
23.22
22.21
20.99
20.58
19.70
17.79
16.36
59
f1 (ppm)

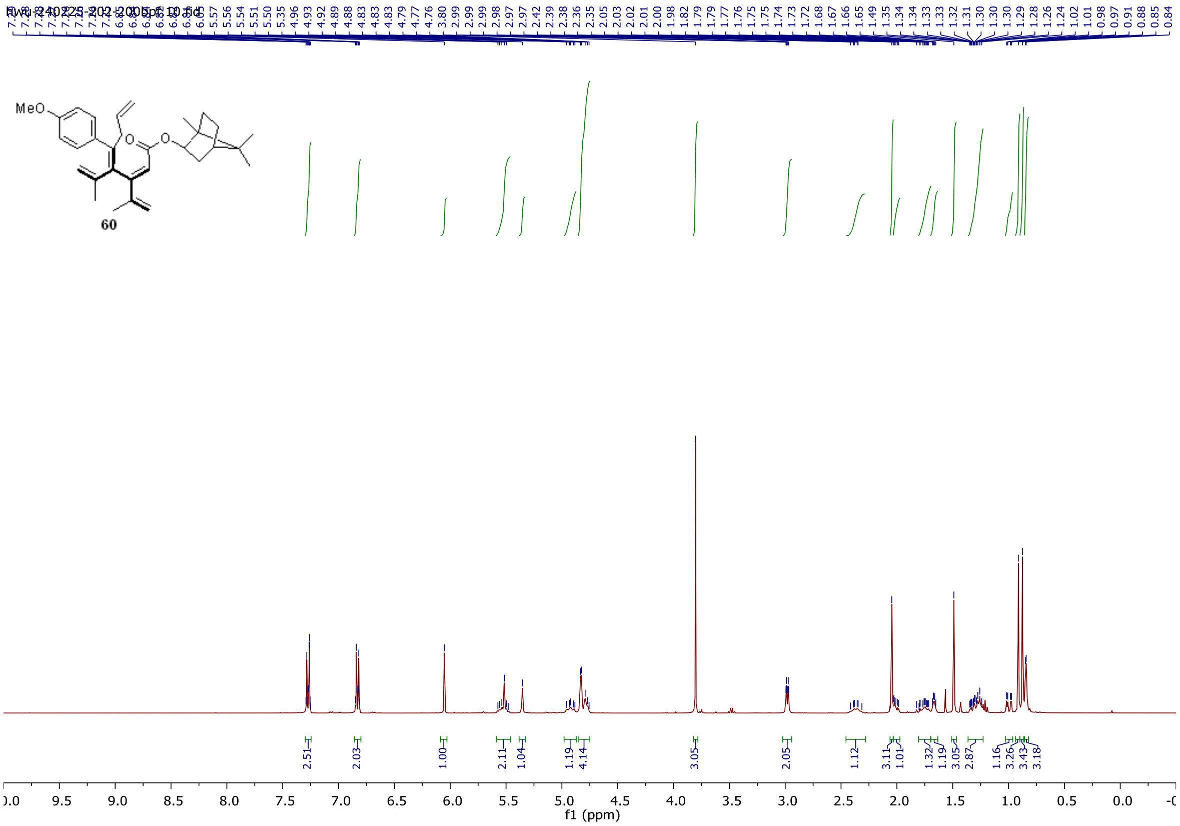
MeO
60
5.57 5.56 5.54 5.51 5.50 5.35 4.96 4.93 4.92 4.89 4.88 4.83 4.83 4.83 4.79 4.77 4.76 3.80 2.99 2.99 2.99 2.98 2.97 2.97 2.42 2.39 2.38 2.36 2.35 2.05 2.03 2.02 2.01 2.00 1.98 1.82 1.79 1.79 1.77 1.76 1.75 1.75 1.74 1.73 1.72 1.68 1.67 1.66 1.65 1.49 1.35 1.34 1.34 1.33 1.33 1.32 1.31 1.30 1.30 1.30 1.30 1.29 1.28 1.26 1.24 1.02 1.01 0.98 0.97 0.91 0.88 0.85 0.84
2.51 2.03 1.00 2.11 1.04 1.19 4.14 3.05 2.05 1.12 3.11 1.01 1.32 1.19 3.05 2.87 1.16 3.26 3.43 3.18
9.5 9.0 8.5 8.0 7.5 7.0 6.5 6.0 5.5 5.0 4.5 4.0 3.5 3.0 2.5 2.0 1.5 1.0 0.5 0.0
f1 (ppm)

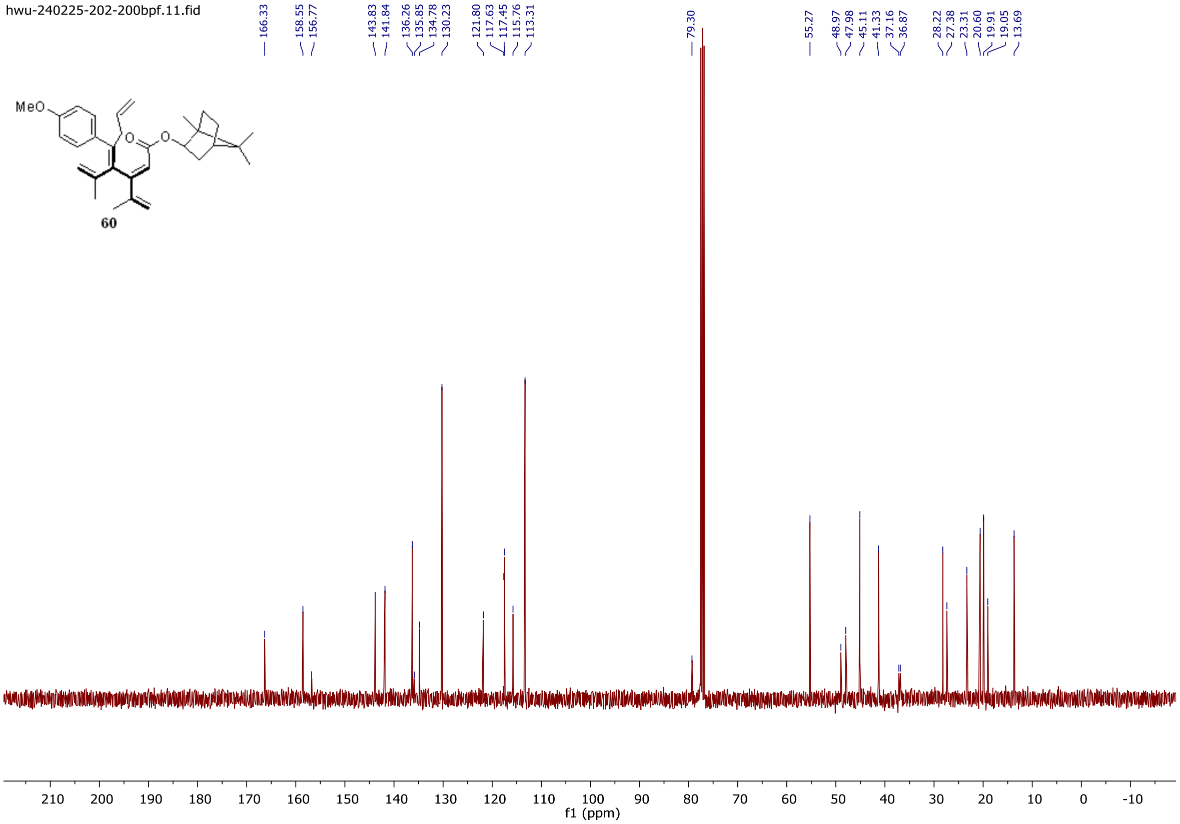
hwu-240225-202-200bpf.11.fid
166.33 158.55 156.77 143.83 141.84 136.26 135.85 134.78 130.23 121.80 117.63 117.45 115.76 113.31 79.30 55.27 48.97 47.98 45.11 41.33 37.16 36.87 28.22 27.38 23.31 20.60 19.91 19.05 13.69
MeO
60
210 200 190 180 170 160 150 140 130 120 110 100 90 80 70 60 50 40 30 20 10 0 -10
f1 (ppm)

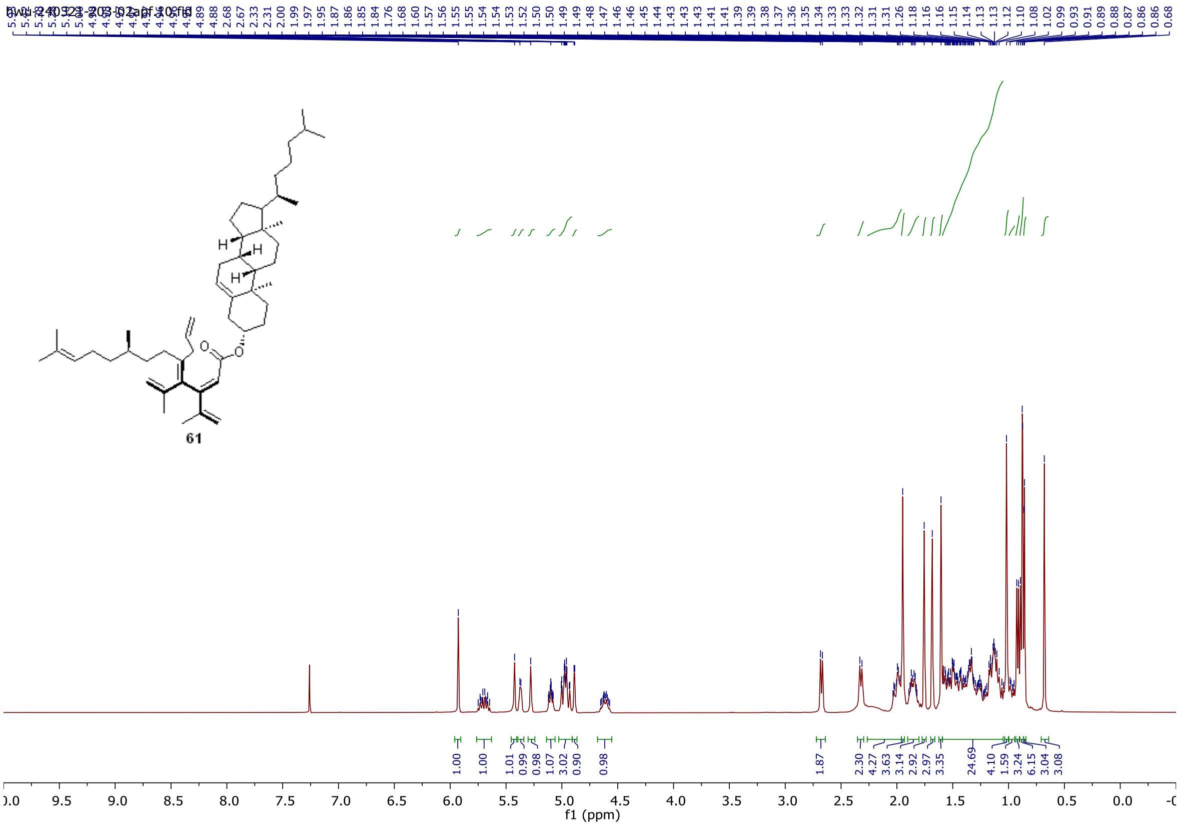
hwu-240321-203-02apf.10.fid
61
1.00
1.00
1.01
0.99
0.98
1.07
3.02
0.90
0.98
1.87
2.30
4.27
3.63
3.14
2.92
2.97
3.35
24.69
4.10
1.59
3.24
6.15
3.04
3.08
f1 (ppm)

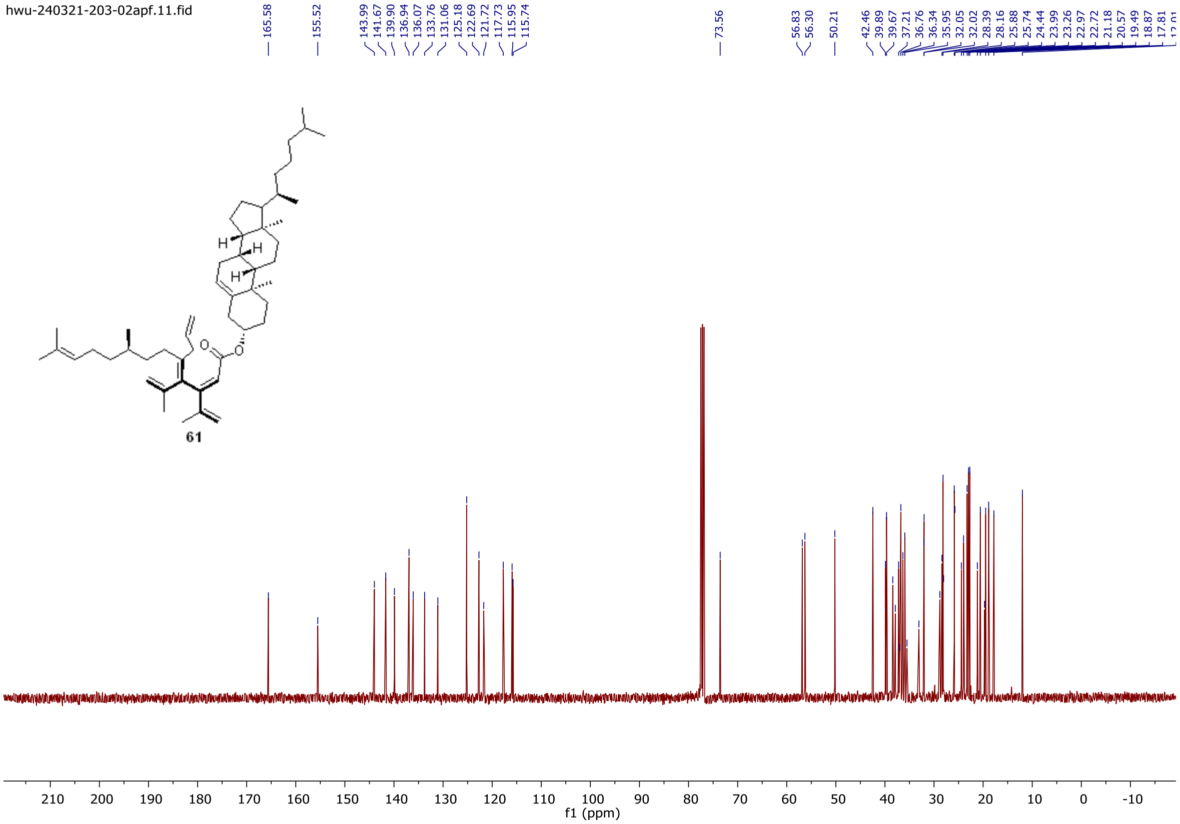
hwu-240321-203-02apf.11.fid
165.58
155.52
143.99
141.67
139.90
136.94
136.07
133.76
131.06
125.18
122.69
121.72
117.73
115.95
115.74
73.56
56.83
56.30
50.21
42.46
39.89
39.67
37.21
36.76
36.34
35.95
32.05
32.02
28.39
28.16
25.88
25.74
24.44
23.99
23.26
22.97
22.72
21.18
20.57
19.49
18.87
17.81
61
f1 (ppm)

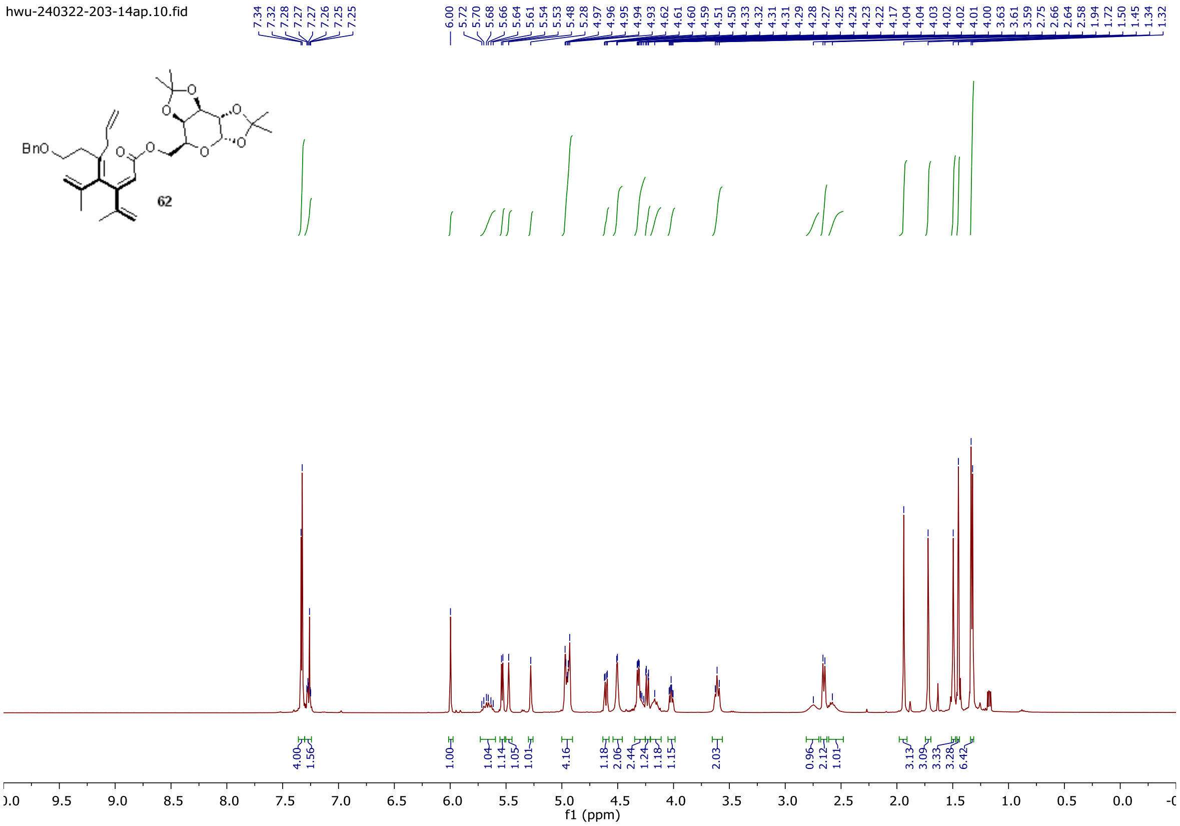

hwu-240322-203-14ap.10.fid
7.34 7.32 7.28 7.27 7.27 7.26 7.25 7.25
6.00 5.72 5.70 5.68 5.66 5.64 5.61 5.54 5.53 5.48 5.28 4.97 4.96 4.95 4.94 4.93 4.62 4.61 4.60 4.59 4.51 4.50 4.33 4.32 4.31 4.29 4.28 4.27 4.25 4.24 4.23 4.22 4.17 4.04 4.04 4.03 4.02 4.02 4.01 4.00 3.63 3.61 3.59 2.75 2.66 2.64 2.58 1.94 1.72 1.50 1.45 1.34 1.32
BnO
62
4.00 1.56 1.00 1.04 1.14 1.05 1.01 4.16 1.18 2.06 2.44 1.24 1.18 1.15 2.03 0.96 2.12 1.01 3.13 3.09 3.33 3.28 6.42
9.5 9.0 8.5 8.0 7.5 7.0 6.5 6.0 5.5 5.0 4.5 4.0 3.5 3.0 2.5 2.0 1.5 1.0 0.5 0.0
f1 (ppm)

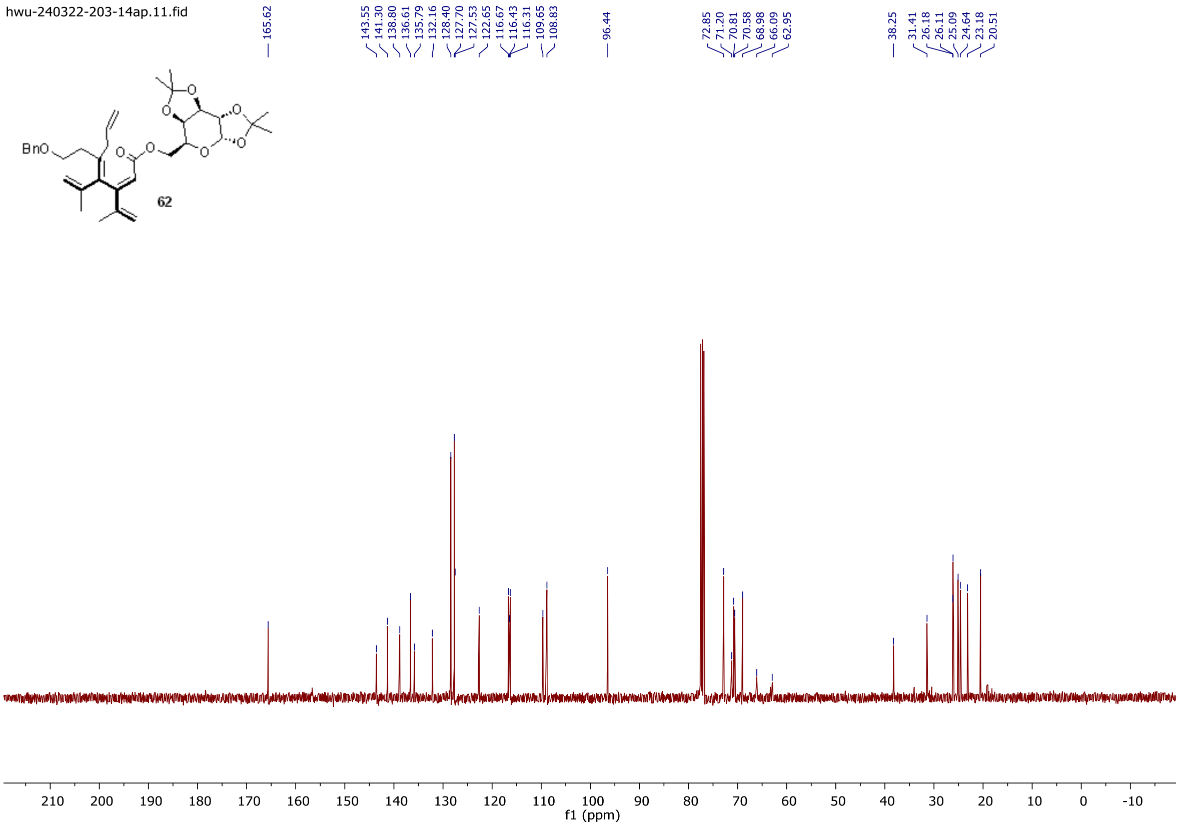

hwu-240322-203-14ap.11.fid
165.62
143.55 141.30 138.80 136.61 135.79 132.16 128.40 127.70 127.53 122.65 116.67 116.43 116.31 109.65 108.83
96.44
72.85 71.20 70.81 70.58 68.98 66.09 62.95
38.25
31.41 26.18 26.11 25.09 24.64 23.18 20.51
BnO
62
210 200 190 180 170 160 150 140 130 120 110 100 90 80 70 60 50 40 30 20 10 0 -10
f1 (ppm)

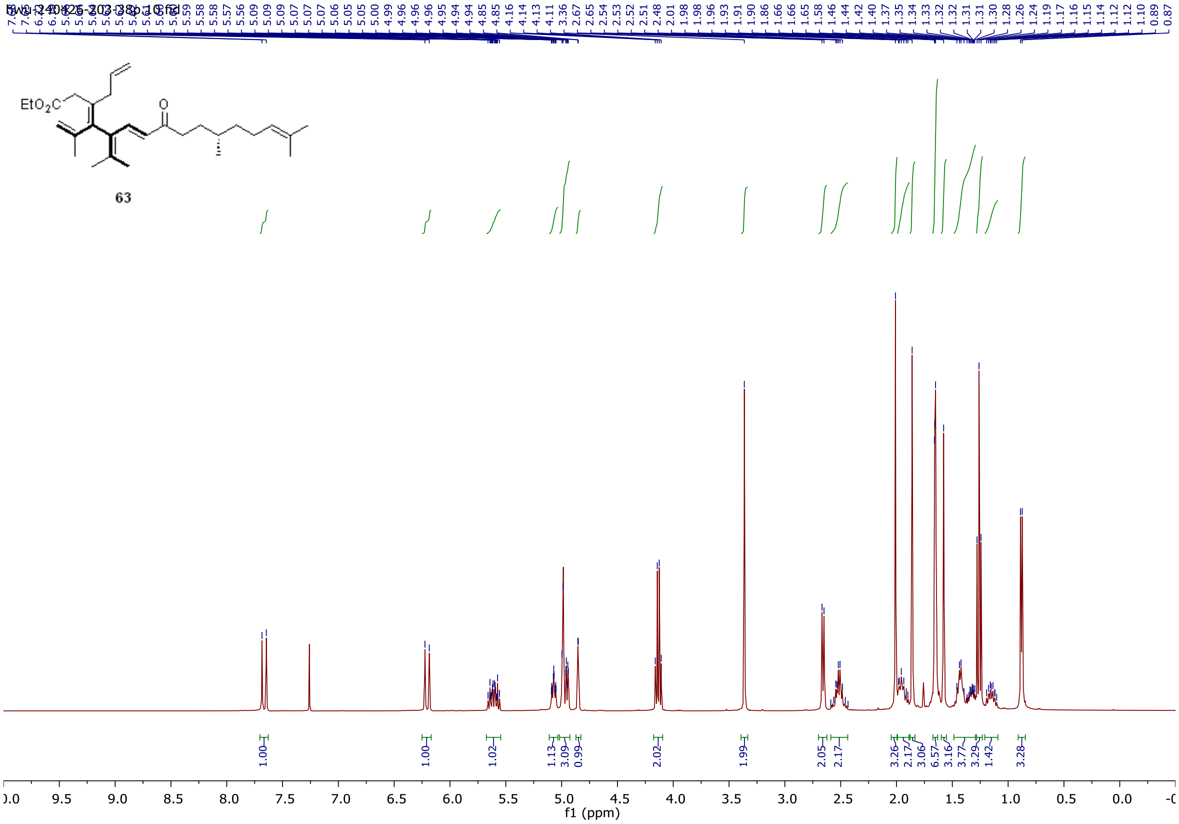
5.59 5.58 5.58 5.57 5.56 5.09 5.09 5.09 5.07 5.07 5.06 5.05 5.05 5.00 4.99 4.96 4.96 4.96 4.95 4.94 4.94 4.85 4.16 4.14 4.13 4.11 3.36 2.67 2.65 2.54 2.53 2.52 2.51 2.48 2.01 1.98 1.98 1.96 1.93 1.91 1.90 1.86 1.66 1.66 1.65 1.58 1.46 1.44 1.42 1.40 1.37 1.35 1.34 1.33 1.32 1.32 1.31 1.31 1.30 1.28 1.26 1.24 1.19 1.17 1.16 1.15 1.14 1.12 1.12 1.10 0.89 0.87
EtO2C
O
63
1.00
1.00
1.02
1.13
3.09
0.99
2.02
1.99
2.05
2.17
3.26
2.17
3.06
6.57
3.16
3.77
3.29
1.42
3.28
f1 (ppm)

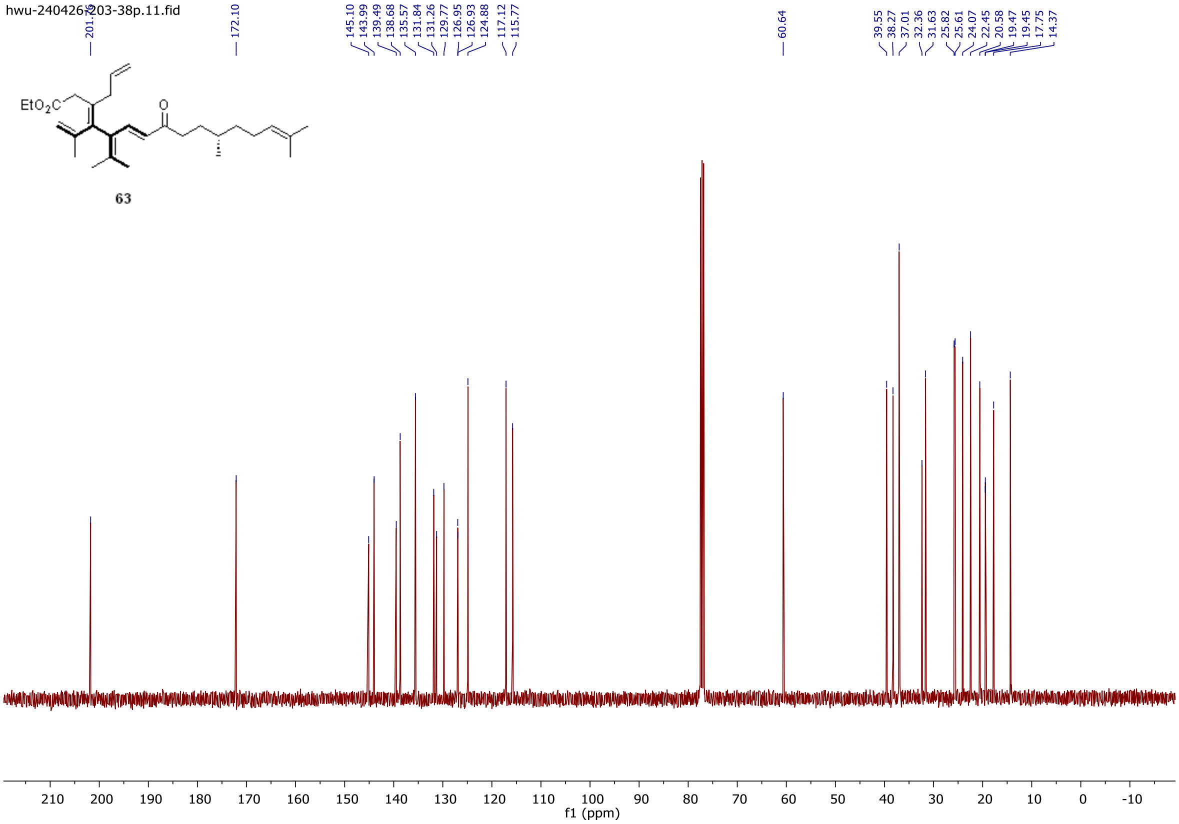
hwu-240426-203-38p.11.fid
172.10
145.10 143.99 139.49 138.68 135.57 131.84 131.26 129.77 126.95 126.93 124.88 117.12 115.77
60.64
39.55 38.27 37.01 32.36 31.63 25.82 25.61 24.07 22.45 20.58 19.47 19.45 17.75 14.37
EtO2C
O
63
f1 (ppm)

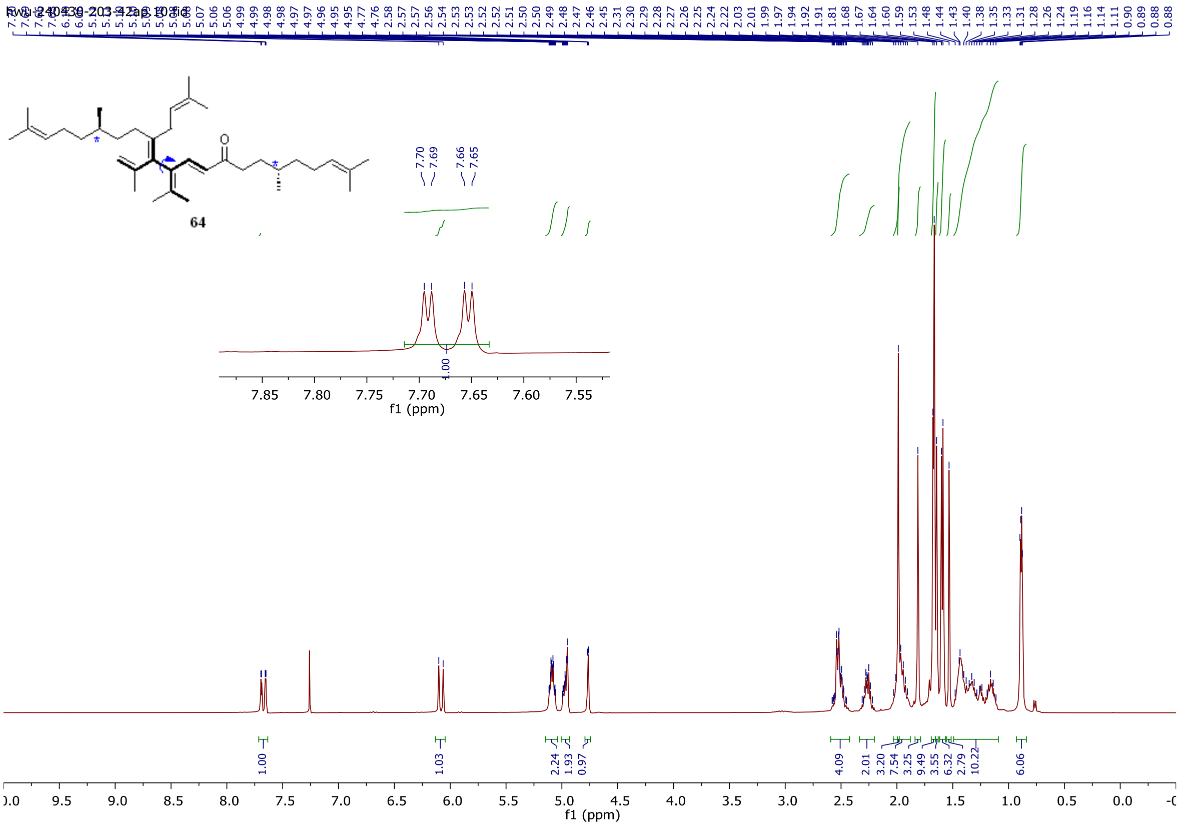

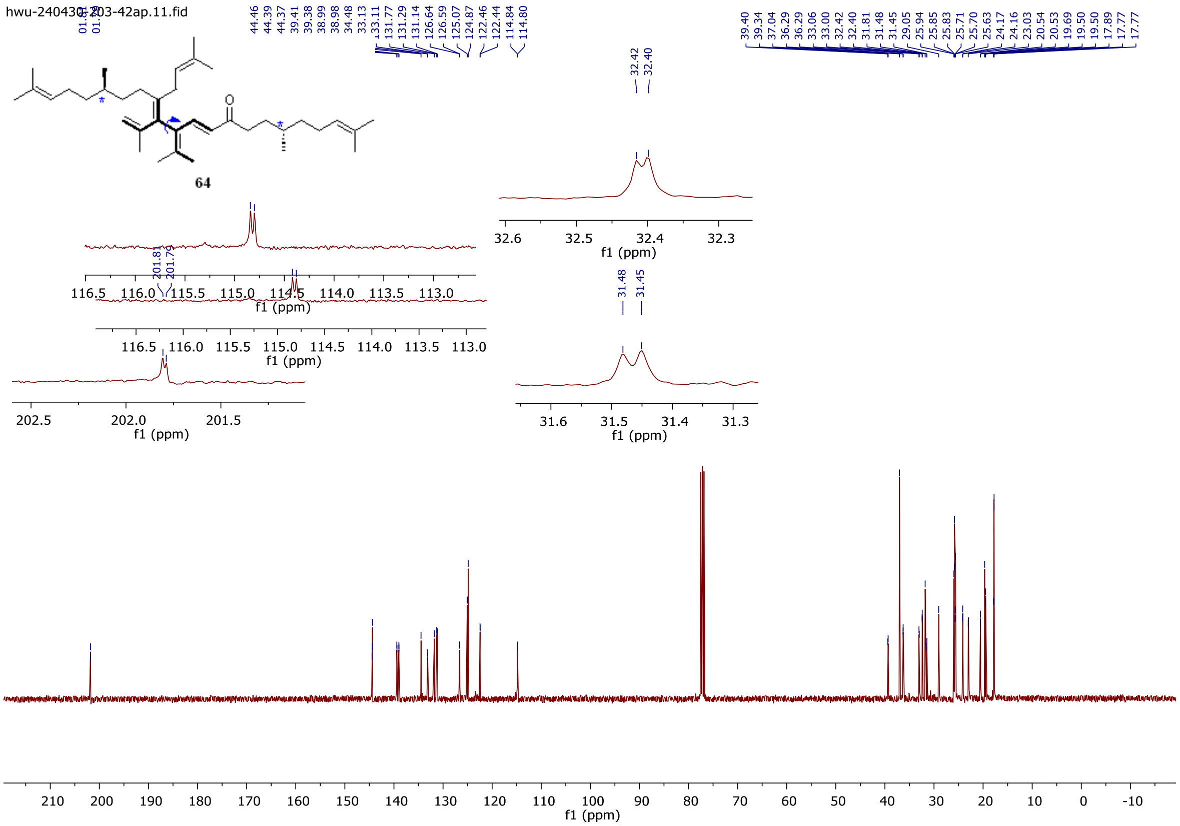

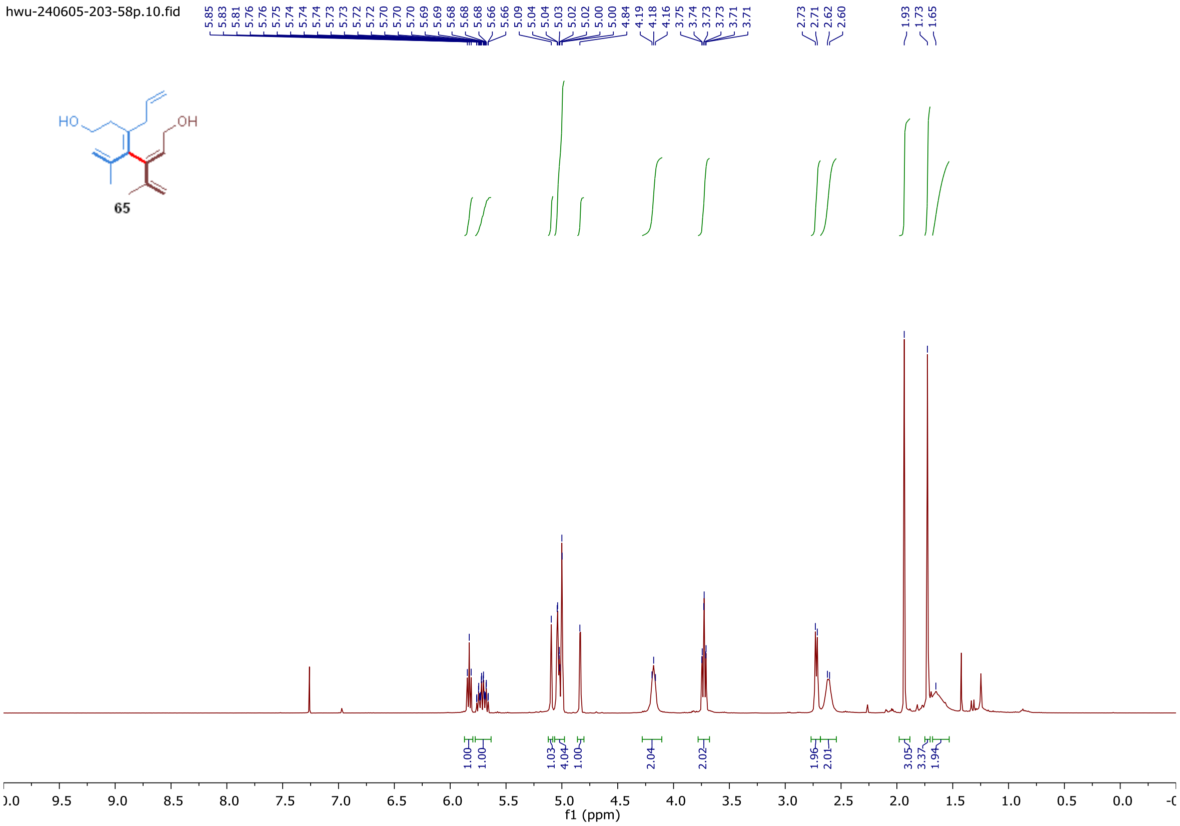
hwu-240605-203-58p.10.fid
5.85 5.83 5.81 5.76 5.75 5.74 5.74 5.74 5.73 5.73 5.72 5.72 5.70 5.70 5.70 5.69 5.69 5.68 5.68 5.68 5.66 5.66 5.09 5.04 5.04 5.03 5.02 5.02 5.00 5.00 4.84 4.19 4.18 4.16 3.75 3.74 3.74 3.73 3.73 3.71 3.71 2.73 2.71 2.62 2.60 1.93 1.73 1.65
HO
OH
65
1.00 1.00 1.03 4.04 1.00 2.04 2.02 1.96 2.01 3.05 3.37 1.94
f1 (ppm)

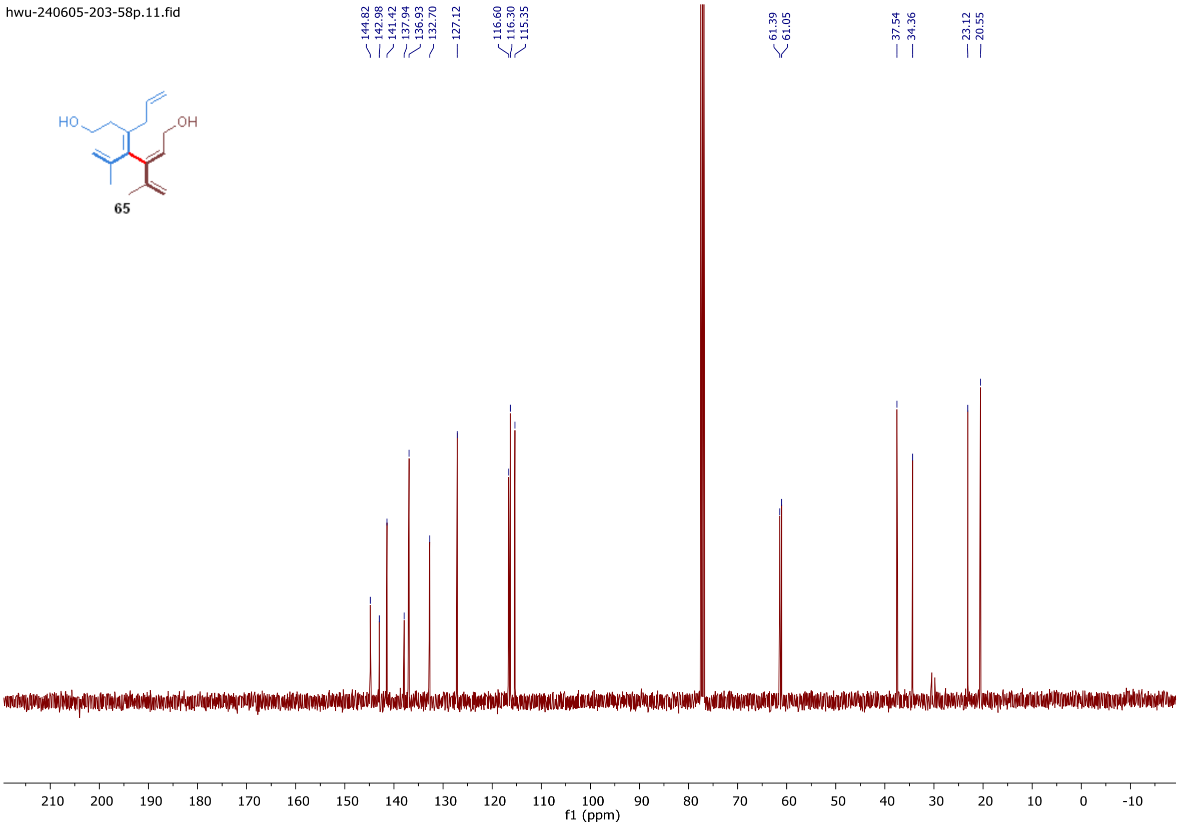
hwu-240605-203-58p.11.fid
144.82 142.98 141.42 137.94 136.93 132.70 127.12 116.60 116.30 115.35 61.39 61.05 37.54 34.36 23.12 20.55
HO
OH
65
f1 (ppm)

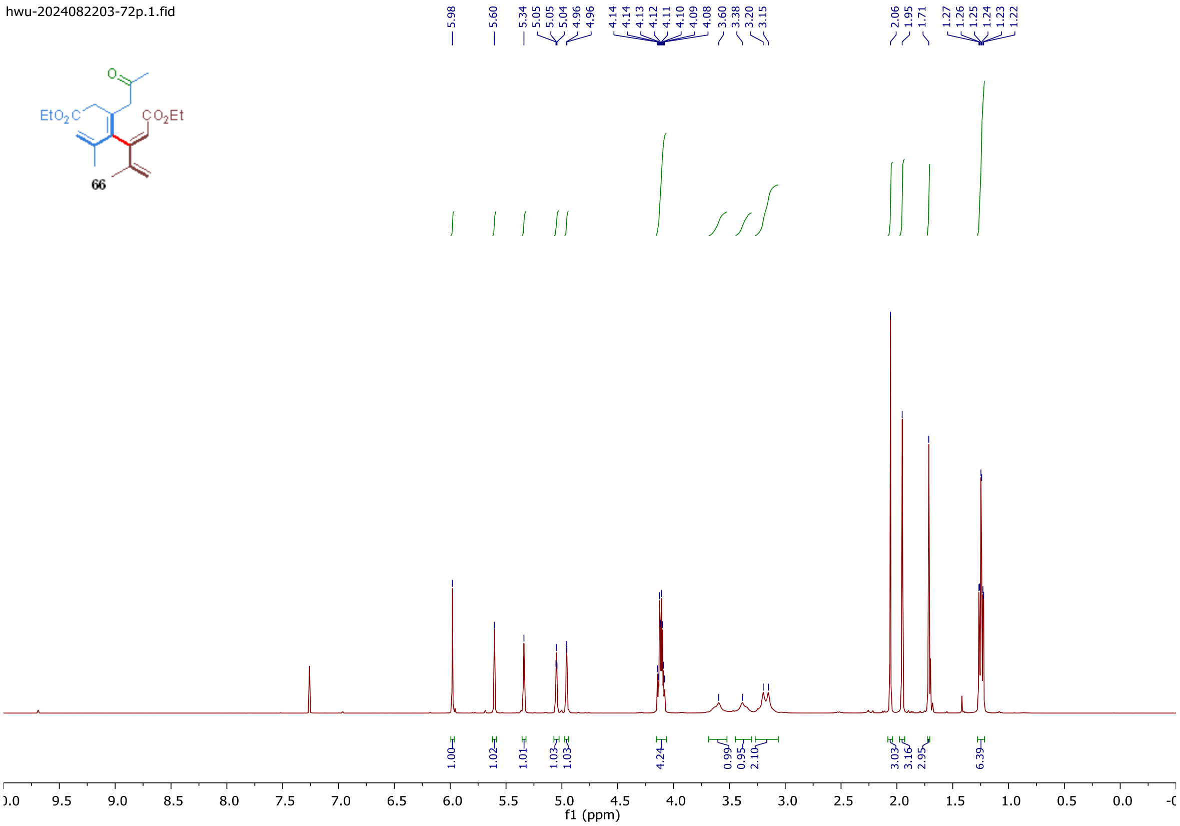
hwu-2024082203-72p.1.fid
EtO2C
O
CO2Et
66
5.98
5.60
5.34
5.05
5.05
5.04
4.96
4.96
4.14
4.14
4.13
4.12
4.11
4.11
4.10
4.09
4.08
3.60
3.38
3.20
3.15
2.06
1.95
1.71
1.27
1.26
1.25
1.24
1.23
1.22
1.00
1.02
1.01
1.03
1.03
4.24
0.99
0.95
2.10
3.03
3.16
2.95
6.39
9.5 9.0 8.5 8.0 7.5 7.0 6.5 6.0 5.5 5.0 4.5 4.0 3.5 3.0 2.5 2.0 1.5 1.0 0.5 0.0
f1 (ppm)

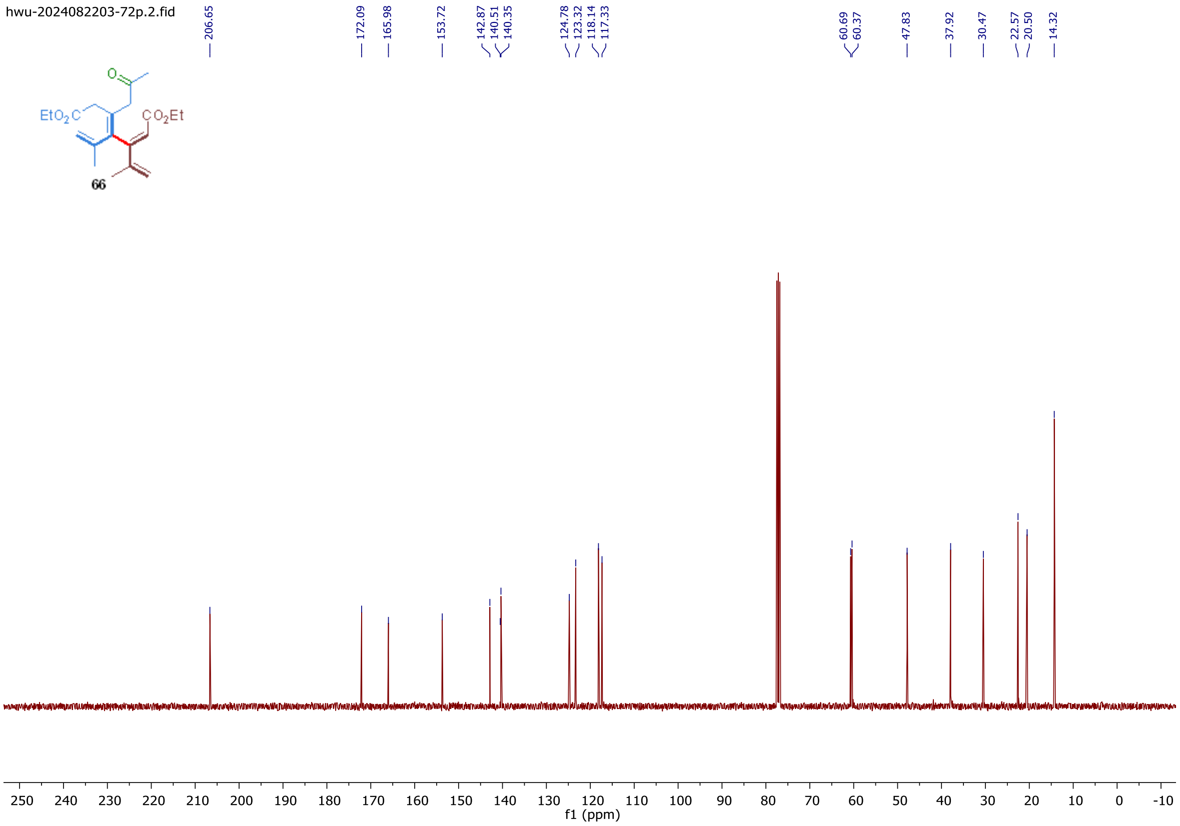
hwu-2024082203-72p.2.fid
EtO2C
O
CO2Et
66
206.65
172.09
165.98
153.72
142.87
140.51
140.35
124.78
123.32
118.14
117.33
60.69
60.37
47.83
37.92
30.47
22.57
20.50
14.32
250 240 230 220 210 200 190 180 170 160 150 140 130 120 110 100 90 80 70 60 50 40 30 20 10 0 -10
f1 (ppm)

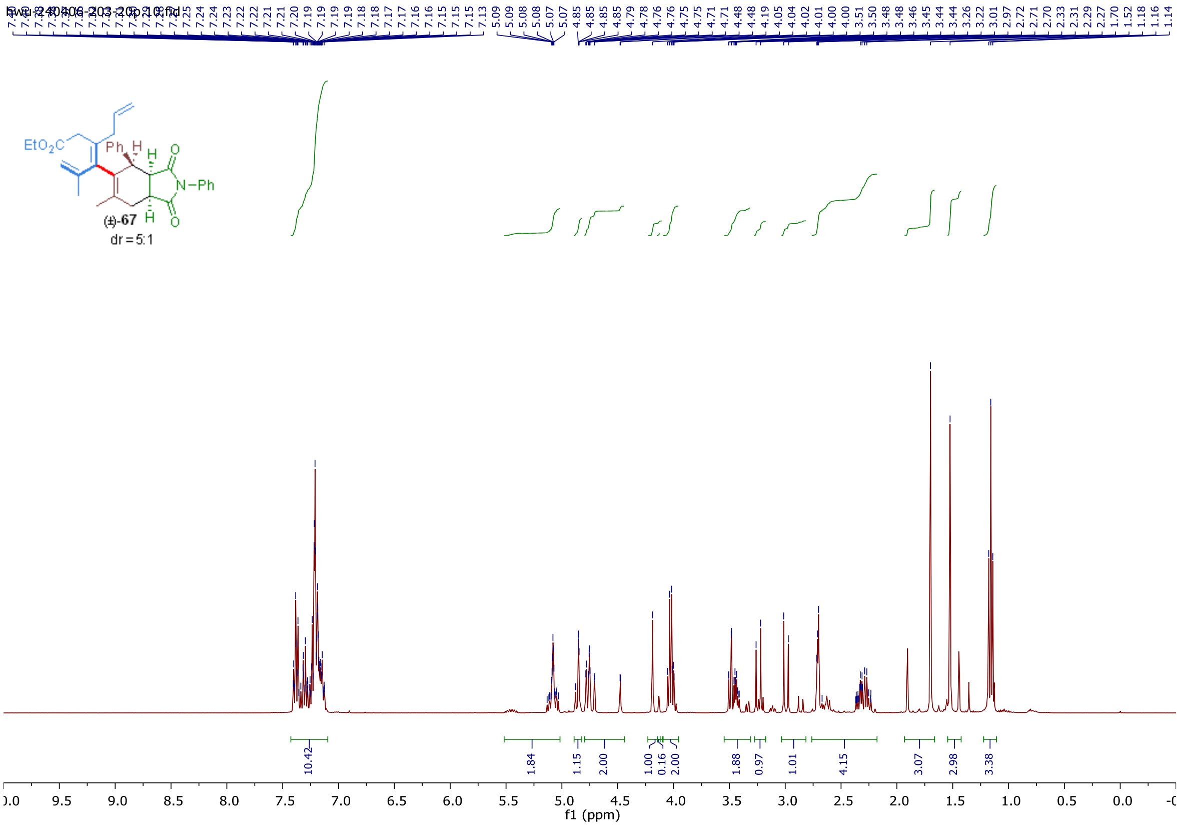
hwu-240406-203-20p.10.fid
5.09
5.09
5.08
5.08
5.07
5.07
4.85
4.85
4.85
4.79
4.78
4.76
4.76
4.75
4.75
4.71
4.71
4.48
4.48
4.19
4.05
4.04
4.02
4.01
4.00
4.00
3.51
3.50
3.48
3.48
3.46
3.45
3.44
3.44
3.26
3.22
3.01
2.97
2.72
2.71
2.70
2.33
2.31
2.29
2.27
1.70
1.52
1.18
1.16
1.14
EtO2C
Ph
H
O
N–Ph
H
O
(±)-67
dr = 5:1
10.42
1.84
1.15
2.00
1.00
0.16
2.00
1.88
0.97
1.01
4.15
3.07
2.98
3.38
f1 (ppm)

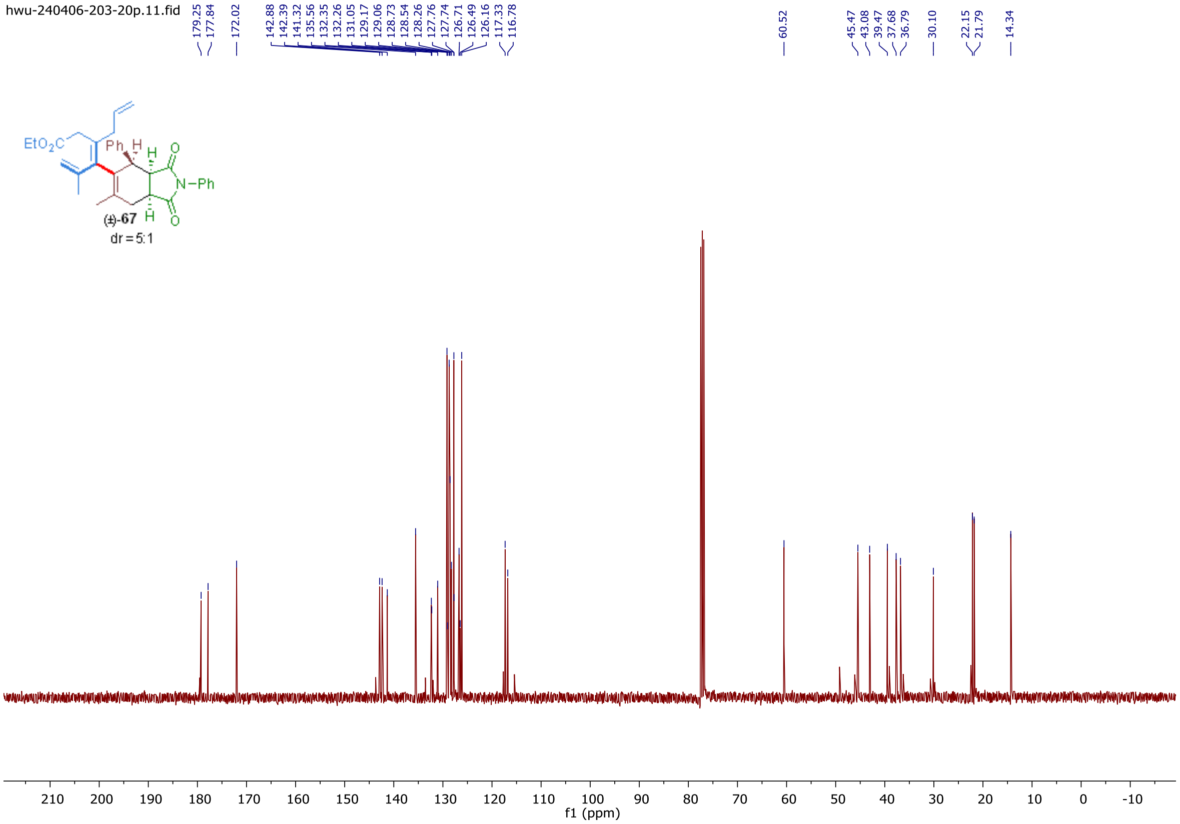
hwu-240406-203-20p.11.fid
179.25
177.84
172.02
142.88
142.39
141.32
135.56
132.35
132.26
131.05
129.17
129.06
128.73
128.54
128.26
127.76
127.74
126.71
126.49
126.16
117.33
116.78
60.52
45.47
43.08
39.47
37.68
36.79
30.10
22.15
21.79
14.34
EtO2C
Ph
H
O
N–Ph
H
O
(±)-67
dr = 5:1
f1 (ppm)

hwu-2024082703-74p1.1.fid

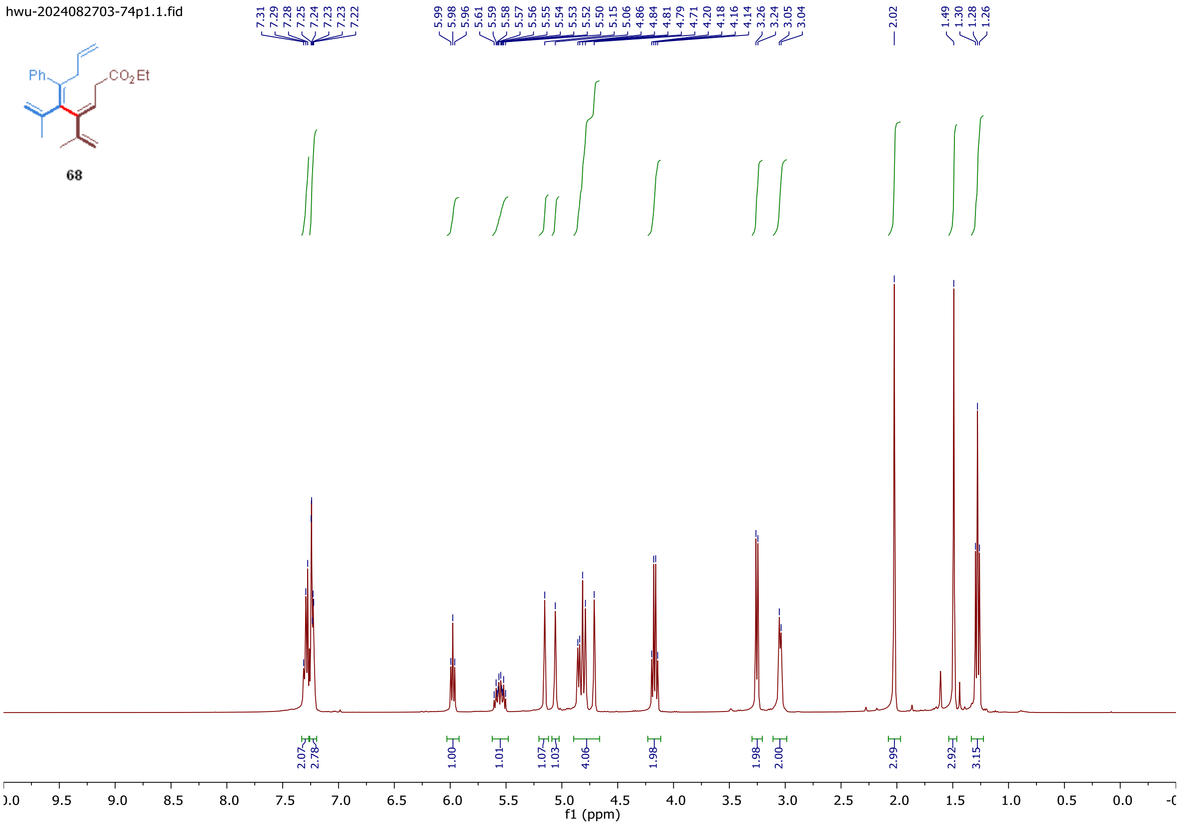

hwu-2024082703-74p1.2.fid

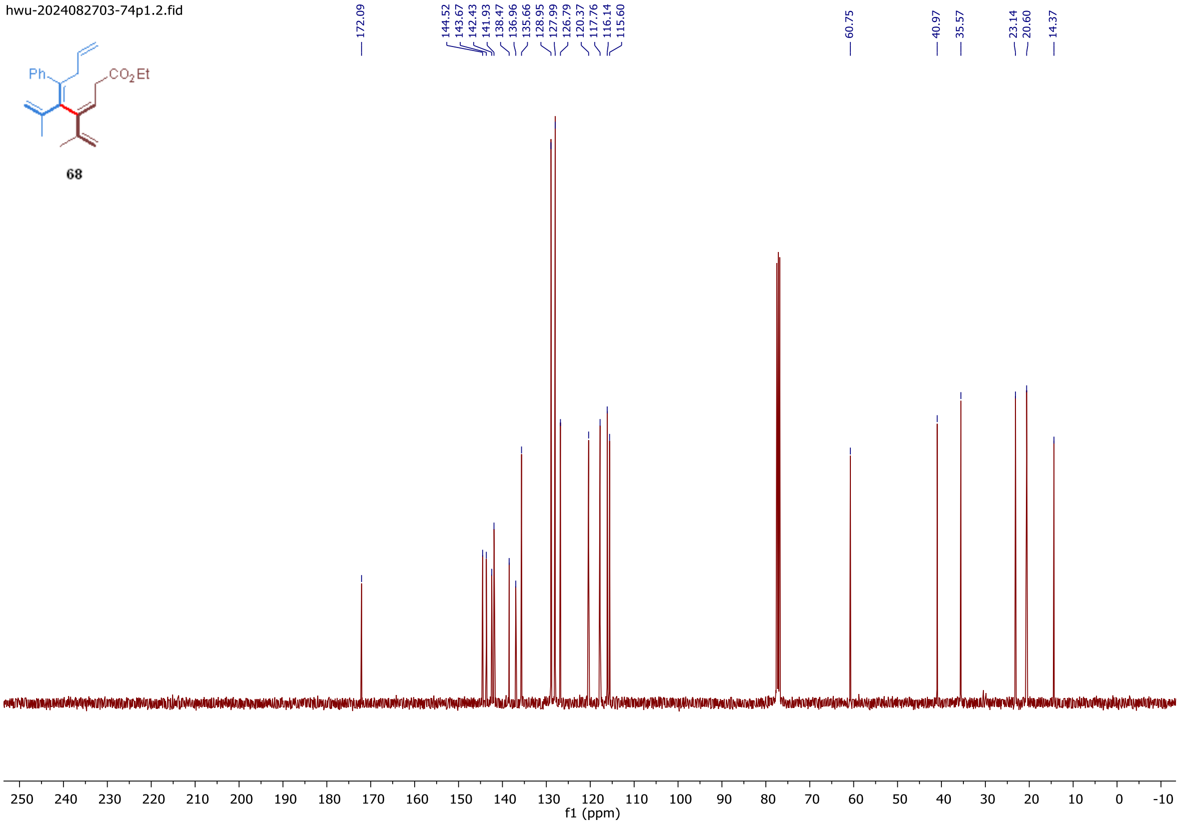

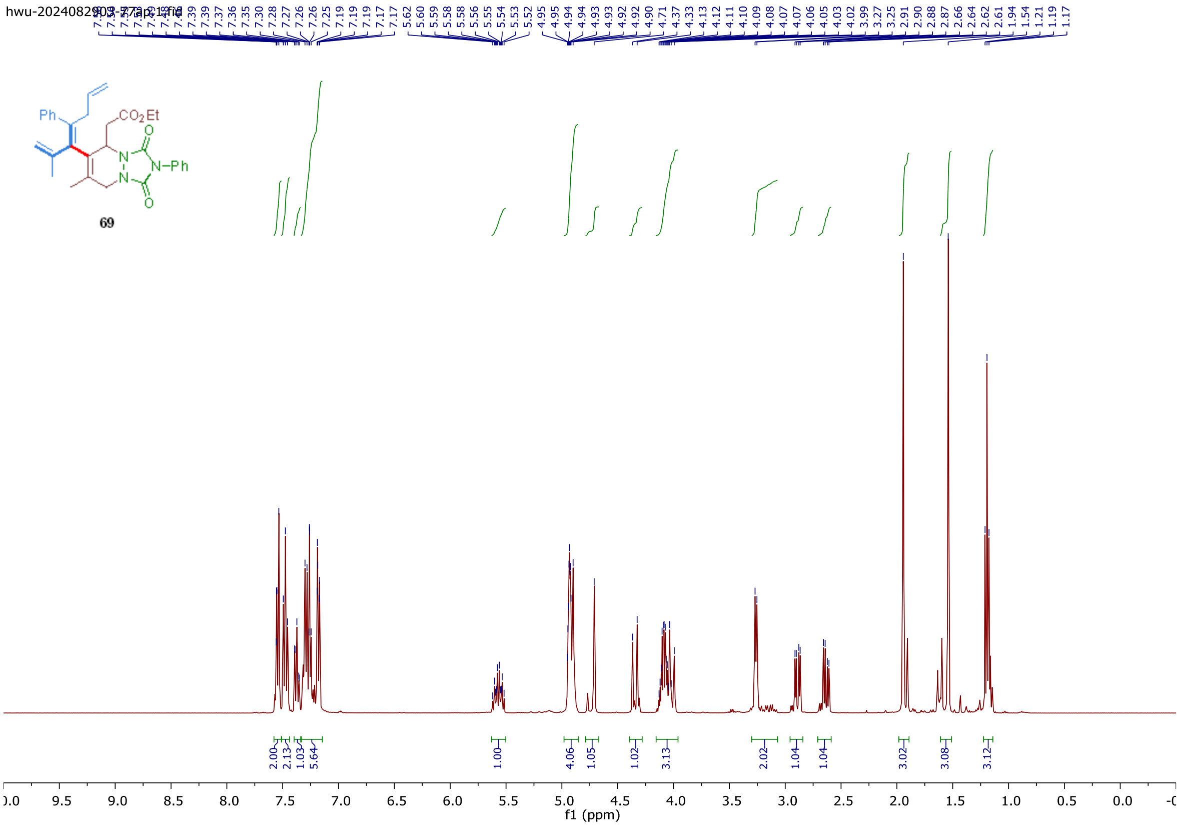
69
f1 (ppm)

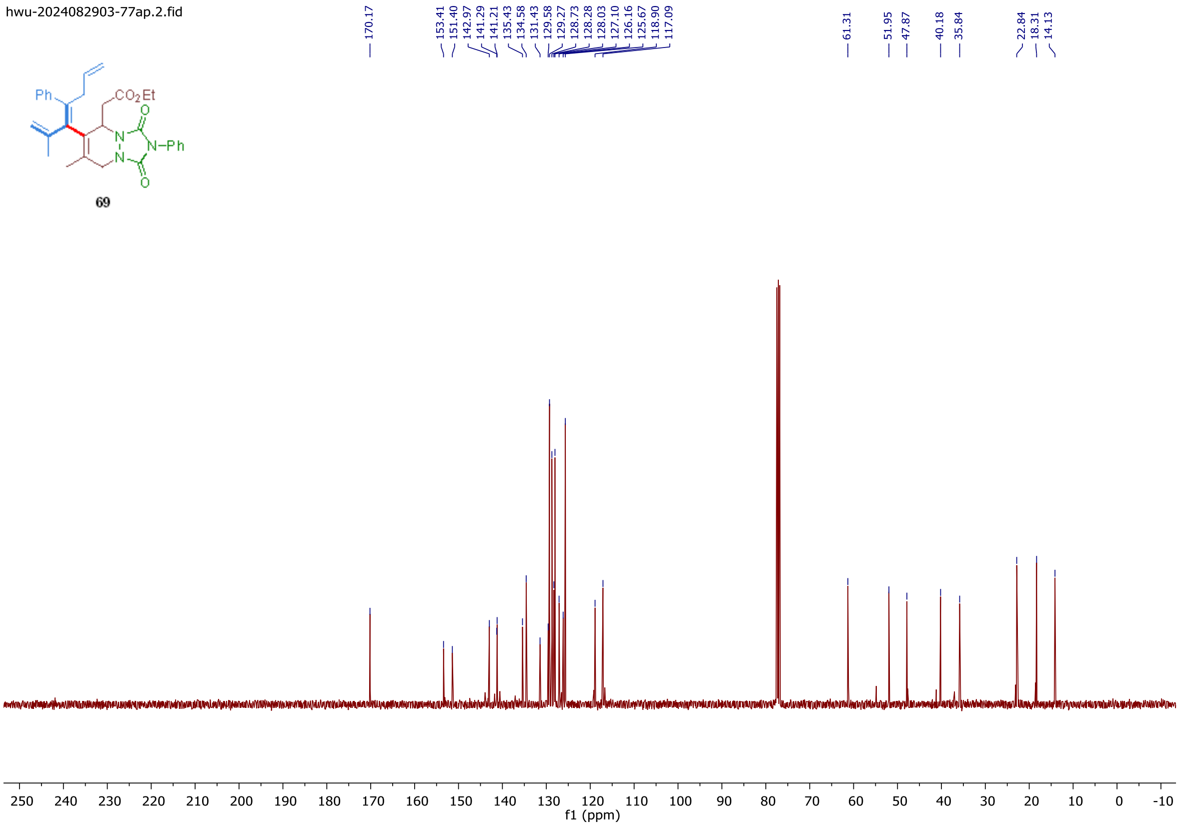
hwu-2024082903-77ap.2.fid
170.17
153.41
151.40
142.97
141.29
141.21
135.43
134.58
131.43
129.58
129.27
128.73
128.28
128.03
127.10
126.16
125.67
118.90
117.09
61.31
51.95
47.87
40.18
35.84
22.84
18.31
14.13
69
f1 (ppm)

7. *References*

(1) Zhu, C.; Yang, B.; Jiang, T.; Bäckvall, J.-E. Olefin-Directed Palladium-Catalyzed Regio- and Stereoselective Oxidative Arylation of Allenes. *Angew. Chem. Int. Ed.* **2015**, *54* (31), 9066–9069.

(2) Qiu, Y.; Yang, B.; Zhu, C.; Bäckvall, J.-E. Palladium-Catalyzed Oxidative Carbocyclization–Borylation of Enallenes to Cyclobutenes. *Angew. Chem. Int. Ed.* **2016**, *55* (22), 6520–6524.

(3) Zhang, H.; Fu, X.; Chen, J.; Wang, E.; Liu, Y.; Li, Y. Generation of Allenic/Propargylic Zirconium Complexes and Subsequent Cross-Coupling Reactions: A Facile Synthesis of Multisubstituted Allenes. *J. Org. Chem.* **2009**, *74* (24), 9351–9358.

(4) Yang, B.; Federmann, P.; Warth, V.; Ren, M.; Mu, X.; Wu, H.; Bäckvall, J.-E. Total Synthesis of Strigolactones via Palladium-Catalyzed Cascade Carbonylative Carbocyclization of Enallenes. *Org. Lett.* **2024**, *26* (22), 4637–4642.

(5) Zhu, C.; Yang, B.; Bäckvall, J.-E. Highly Selective Cascade C–C Bond Formation via Palladium- Catalyzed Oxidative Carbonylation–Carbocyclization–Carbonylation–Alkynylation of Enallenes. *J. Am. Chem. Soc.* **2015**, *137* (37), 11868–11871.

(6) Yang, B.; Qiu, Y.; Jiang, T.; Wulff, W. D.; Yin, X.; Zhu, C.; Bäckvall, J.-E. Enantioselective Palladium-Catalyzed Carbonylative Carbocyclization of Enallenes via Cross-Dehydrogenative Coupling with Terminal Alkynes: Efficient Construction of α-Chirality of Ketones. *Angew. Chem. Int. Ed.* **2017**, *56* (16), 4535–4539.

(7) Kessler, S. N.; Bäckvall, J.-E. Iron-Catalyzed Cross-Coupling of Propargyl Carboxylates and Grignard Reagents: Synthesis of Substituted Allenes. *Angew. Chem. Int. Ed.* **2016**, *55* (11), 3734–3738.

(8) Keck, G. E.; Giles, R. L.; Cee, V. J.; Wager, C. A.; Yu, T.; Kraft, M. B. Total Synthesis of Epothilones B and D: Stannane Equivalents for β-Keto Ester Dianions. *J. Org. Chem.* **2008**, *73* (24), 9675–9691.

(9) Shen, R.; Luo, B.; Yang, J.; Zhang, L.; Han, L.-B. Convenient Synthesis of Allenylphosphoryl Compounds via Cu-Catalysed Couplings of P(O)H Compounds with Propargyl Acetates. *Chem. Commun.* **2016**, *52* (38), 6451–6454.

(10) Semba, K.; Fujihara, T.; Terao, J.; Tsuji, Y. Copper-Catalyzed Borylation of α-Alkoxy Allenes with Bis(Pinacolato)Diboron: Efficient Synthesis of 2-Boryl 1,3-Butadienes. *Angew. Chem. Int. Ed.* **2013**, *52* (47), 12400–12403.

(11) Eshon, J.; Landis, C. R.; Schomaker, J. M. Regioselective Rh-Catalyzed Hydroformylation of 1,1,3-Trisubstituted Allenes Using BisDiazaPhos Ligand. *J. Org. Chem.* **2017**, *82* (18), 9270–9278.

(12) Ting, C.-M.; Hsu, Y.-L.; Liu, R.-S. Gold-Catalyzed Isomerization of Unactivated Allenes into 1,3-Dienes under Ambient Conditions. *Chem. Commun.* **2012**, *48* (52), 6577–6579.

(13) Chen, W.; Walker, J. C. L.; Oestreich, M. Metal-Free Transfer Hydroiodination of C–C Multiple Bonds. *J. Am. Chem. Soc.* **2019**, *141* (2), 1135–1140.

(14) Trost, B. M.; Pinkerton, A. B.; Seidel, M. Ruthenium-Catalyzed Two-Component Addition To Form 1,3-Dienes: Optimization, Scope, Applications, and Mechanism. *J. Am. Chem. Soc.* **2001**, *123* (50), 12466–12476.

(15) Qiu, Y.; Posevins, D.; Bäckvall, J.-E. Selective Palladium-Catalyzed Allenic C−H Bond Oxidation for the Synthesis of [3]Dendralenes. *Angew. Chem. Int. Ed.* **2017**, *56* (42), 13112–13116.

(16) Wang, C.; Zhao, W.; Li, H.; Guo, L. Solvent-Free Synthesis of Unsaturated Ketones by the Saucy–Marbet Reaction Using Simple Ammonium Ionic Liquid as a Catalyst. *Green Chem.* **2009**, *11* (6), 843–847.

(17) Hosseyni, S.; Ding, S.; Su, Y.; Akhmedov, N. G.; Shi, X. Triazole–Gold Promoted Intermolecular Propargyl Alcohol Addition to Alkyne: The Reaction Cascade toward Substituted Allenes. *Chem. Commun.* **2016**, *52* (2), 296–299.

(18) Xu, D.; Lu, Z.; Li, Z.; Ma, S. Novozym 435-Catalyzed Kinetic Resolution of β-Allenols. A Facile Route for the Preparation of Optically Active β-Allenols or Allenyl Acetates. *Tetrahedron* **2004**, *60* (51), 11879–11887.

(19) Li, S.; Hou, B.; Wang, J. Palladium-Catalyzed Oxidative Coupling of the Allenic C–H Bond with α-Diazo Esters: Synthesis of [3]Dendralenes. *J. Org. Chem.* **2021**, *86* (7), 5371–5379.

(20) Savignac, P. B., Anne; Charrier, Claude; Mathey, François. Synthesis of Dialkyl 2,3-Alkadienephosphonates by Reaction of the 1-Copper(I) Derivatives of Alkyl Alkanephosphonates with 2-Alkynyl Halides, 2-Alkynyl Benzenesulfonates, or 1-Bromoallenes. *Synthesis* **2002**, *1979* (10), 832–834.

(21) Tang, M.; Fan, C.-A.; Zhang, F.-M.; Tu, Y.-Q.; Zhang, W.-X.; Wang, A.-X. New Metal-Free One-Pot Synthesis of Substituted Allenes from Enones. *Org. Lett.* **2008**, *10* (24), 5585–5588.

(22) Wang, H.; Glorius, F. Mild Rhodium(III)-Catalyzed C-H Activation and Intermolecular Annulation with Allenes. *Angew. Chem. Int. Ed.* **2012**, *51* (29), 7318–7322.

(23) Devi, N. R.; Sultana, S.; Borah, M.; Saikia, A. K. Regio- and Diastereoselective Synthesis of Dihydropyrans and Pyranopyrans via Oxonium–Ene Reaction of β-Allenols and Aldehydes. *J. Org. Chem.* **2018**, *83* (24), 14987–14998.

(24) Devi, N. R.; Sultana, S.; Borah, M.; Saikia, A. K. Regio- and Diastereoselective Synthesis of Dihydropyrans and Pyranopyrans via Oxonium–Ene Reaction of β-Allenols and Aldehydes. *J. Org. Chem.* **2018**, *83* (24), 14987–14998.

(25) Li, Y.; Zhang, W.-S.; Yang, S.-N.; Wang, X.-Y.; Liu, Y.; Ji, D.-W.; Chen, Q.-A. Nickel-Catalyzed Unsymmetrical Bis-Allylation of Alkynes. *Angew. Chem. Int. Ed.* **2023**, *62* (17), e202300036.

(26) Fürstner, A.; Flügge, S.; Larionov, O.; Takahashi, Y.; Kubota, T.; Kobayashi, J. Total Synthesis and Biological Evaluation of Amphidinolide V and Analogues. *Chem. Eur. J.* **2009**, *15* (16), 4011–4029.

(27) Chen, C. S.; Fujimoto, Y.; Girdaukas, G.; Sih, C. J. Quantitative Analyses of Biochemical Kinetic Resolutions of Enantiomers. *J. Am. Chem. Soc.* **1982**, *104* (25), 7294–7299.
